# Supplementary material for: A community resource to mass explore the wheat grain proteome and its application to the late-maturity alpha-amylase (LMA) problem
Source: Gigascience. 2023 Nov 1;12:giad084. doi: 10.1093/gigascience/giad084 (PMC10627334; doi:10.1093/gigascience/giad084)
Supplement: giad084_Supplemental_Files [file giad084_supplemental_files.zip › Vincent_Suppl-File-SF1.pdf]

A community resource to mass explore the wheat grain proteome and its application to the LMA problem.

Vincent D. *et al.* 2023

Supplementary File SF1

Table of Contents

A community resource to mass explore the wheat grain proteome and its application to the LMA problem. 1

1. High-throughput proteomics workflow to efficiently process and analyse thousands of samples ... 2

2. Background information on statistical analyses for big data 3

2.1 Unsupervised multivariate clustering analyses (PCA, SOM, k-means, HCA) 3

2.2. Bivariate analyses (correlation and linear regression) 3

2.3. Volcano plot and heat map 4

2.4. References 4

3. MS/MS method parameters 6

4. Galaxy workflow to create a wheat non redundant FASTA database with contaminant and decoy sequences 382

5. R script to link LC-MS1 clusters to LC-MS2 clusters 392

5.1 LCMS1 data 392

5.2 LCMS2 data 392

5.3 R script 392

5.4 LCMS1-LCMS2 linked output 394

1. High-throughput proteomics workflow to efficiently process and analyse thousands of samples

Samples were packaged in small plastic and randomised by mixing them all up in a big box. The QR codes were scanned to populate an Excel spreadsheet assigning a unique number to each individual sample against sample ID. The Excel spreadsheet was readily accessible and populated on the fly by all involved using Microsoft Sharepoint from any laptop in the lab along with the SOP and other relevant documentation. Every workflow step was captured in that single Excel spreadsheet with the date, experimenter, parameters and comments where applicable. It contained 4,400 rows and 110 columns and was used as sample metadata during the statistical analyses and data correction methods.

The full content of a package was poured into a numbered 15 mL grinding jar with metal balls. Jars were arranged into two cardboard boxes to grind 200 samples per day. The samples were ground in batches of 36 samples simultaneously using a Genogrinder. Finely ground flour was transferred from the grinding jar into a microtube prelabelled with printed labels displaying the unique sample number for tracking purpose and a QR code capturing it along with the unique sample ID. Microtubes containing flour samples were stored in cardboard boxes fitting 81 tubes at -80°C until weighing and extraction. The grinding jars and metal balls were thoroughly washed to be re-used.

The thawed flour was individually and manually weighed with 1% accuracy into a prelabelled microtube. The flour was resuspended into an extraction buffer using a sonicating probe, a vortex and heating blocks in batches of 48 samples per day. Solubilised flours were spun 24 tubes at a time using two benchtop centrifuges; supernatants transferred into prelabelled microtubes. Microtubes containing protein extracts were stored in cardboard boxes fitting 81 tubes at -80°C until protein digestion.

A set of 192 frozen protein extracts were thawed overnight at 4°C and aliquoted (10 µL) into two 96-well plates for Trypsin/Lys-C digestion overnight. The IS was introduced at the end of the digestion step. Each digestion plate had a unique plate number that was tracked against all individual samples it contained. The plates were stored at -80°C until SPE.

The peptide digests were SPE-cleaned using a plate manifold, in batches of 4 plates per day (384 digests). Cleaned digests were eluted into 96-well plates bearing the same unique plate number for tracking purpose and stored at -80°C until evaporation and reconstitution.

Samples were completely evaporated using a SpeedVac fitted with a rotor accommodating 4 frozen plates at a time (384 samples/day). Dried samples were reconstituted and homogenised using a multiplate vortex shaker holding four 96-well plates. Plates with reconstituted samples were stored at -80°C until LC-MS analyses.

The Vanquish UPLC accommodated four 96-well plates simultaneously. Each sample was injected once (unless the raw data was unusable; then a duplicate was obtained) and LC-MS analysed for 43 min so that 33 samples were run daily. Enough QC, IS and blanks were prepared to last the full LC-MS run. They were injected once every 48 wheat samples, amounting at the end of the total run to 86 replicates. Data files from the blank sample were inspected throughout the run to check for carry-over peaks (data not shown). Data files from the IS sample were used throughout the run to check for retention time drift, loss of peak resolution and signal intensity, as well as low mass accuracy (data not shown). They were also used during data file processing to isolate the LC-MS peaks belonging to the spiked IS among the wheat peaks. Data files from the QC sample were reviewed throughout the run to also assess loss of peak resolution and signal intensity, as well as batch reproducibility (data not shown). Instrument maintenance such as source and heated capillary cleaning, mass calibration and LC column swapping occurred to address retention time drift, as well as loss of peak resolution, signal intensity, and mass accuracy.

2. Background information on statistical analyses for big data

2.1 Unsupervised multivariate clustering analyses (PCA, SOM, k-means, HCA)

Principal component analysis (PCA) dates from 1901 (Pearson 1901) which reduces the dimensionality of a dataset by linearly transforming the data into a new coordinate system where the variation in the data can be described with fewer dimensions than the initial data. The first two principal components are commonly used to chart the data as a scatterplot and to visually detect clusters of closely related data points. Biological studies such as transcriptomics and proteomics generating large datasets typically resort to PCA to identify trends in the multidimensional data while preserving the maximum amount of information (Jolliffe and Cadima 2016).

Invented in the 80’s Kohonen (Kohonen 1982), a self-organising map (SOM) is an unsupervised machine learning technique that generates a low-dimensional representation of a higher dimensional dataset while preserving the topological structure of the data; a two-dimensional organization of the model effectively approximates similarities of high-dimensional data features (Kohonen 2013).

Invented in the 50’s, K-means is another unsupervised machine learning method and its ease of implementation, simplicity, computational efficiency, and empirical success make it one of the most popular clustering methods (Jain 2010). In this clustering algorithm, n observations are segmented into k clusters in which each observation belongs to the cluster with the nearest mean, thereby minimising within-cluster variances. K is a key parameter that need to be prespecified. The algorithm then iteratively alternates between assigning each observation to its nearest centroid and shifting the location of the centroid to the mean of all data points until the classification remains unchanged (Bi et al. 2019, Cresta Morgado et al. 2022).

Divisive hierarchical clustering analysis (HCA) was introduced in 1999 to compare microarray data from different cell lines and tissues in order to discover colon tumour biomarkers (Alon et al. 1999). In divisive HCA, two vectors are randomly initialized and assigned each gene product using a probability function; the vectors are iteratively recalculated to form the centroids of two clusters. The same splitting process applies to each successive cluster, until each cluster consists of a single profile, and the binary tree is built from the history of the data splitting (Sherlock 2000). Two-dimensional agglomerative clustering visualised with coloured heat maps was also devised by Alon and colleagues in which not only the rows (gene products such transcripts, peptides) but also the columns (arrays, samples, stages, conditions, etc…) are rearranged (Alon et al. 1999).

2.2. Bivariate analyses (correlation and linear regression)

Formulated in 1895 (Pearson 1895), correlation analysis aims at measuring an association or linear relationship between two continuous variables, by which the change in the magnitude of one variable is associated with a change in the magnitude of the other variable, either in the same (positive correlation) or in the opposite (negative correlation) direction (Schober, Boer, and Schwarte 2018). Correlation coefficients (R2 or r) are scaled from -1 to +1, where 0 denotes no association and the relationship gets stronger towards an absolute value of 1.

Simple linear regression dates from 1805 (Legendre 1805) and determines the relationship between a quantitative outcome and a single quantitative explanatory variable (Seltman 2018), such as LMA.

2.3. Volcano plot and heat map

A volcano plot is a type of scatterplot that shows statistical significance (q-value) versus magnitude of change (effect size or fold change). It is often utilised for biomarker discovery in differential expression studies, in particular transcriptomics (Li 2012). It enables quick visual identification of gene products with large fold changes that are not only statistically significant but potentially biologically significant as well.

Heat maps offer an efficient method of visualizing complex quantitative data sets organized as matrices (Key 2012). A typical matrix is created by arranging the data such that each column contains the amount (intensity) from a single sample and each row corresponds to a single feature such as peptide. The intensities of the dependent variables in each cell are then converted into colours to create a heat map. Heat maps are flexible in that both the rows and columns can be arranged to explore a particular experimental hypothesis thus enabling a fast overview of data with a third quantitative dimension captured as coloured gradients (Pleil et al. 2011).

2.4. References

Alon, U., N. Barkai, D. A. Notterman, K. Gish, S. Ybarra, D. Mack, and A. J. Levine. 1999. "Broad patterns of gene expression revealed by clustering analysis of tumor and normal colon tissues probed by oligonucleotide arrays." *Proc Natl Acad Sci U S A* 96 (12):6745-50. doi: 10.1073/pnas.96.12.6745.

Bi, Q., K. E. Goodman, J. Kaminsky, and J. Lessler. 2019. "What is Machine Learning? A Primer for the Epidemiologist." *Am J Epidemiol* 188 (12):2222-2239. doi: 10.1093/aje/kwz189.

Cresta Morgado, P., M. Carusso, L. Alonso Alemany, and L. Acion. 2022. "Practical foundations of machine learning for addiction research. Part I. Methods and techniques." *Am J Drug Alcohol Abuse* 48 (3):260-271. doi: 10.1080/00952990.2021.1995739.

Jain, A. K. 2010. "Data clustering: 50 years beyond K-means." *Pattern Recognition Letters* 31:651-666. doi: 10.1016/j.patrec.2009.09.011.

Jolliffe, I. T., and J. Cadima. 2016. "Principal component analysis: a review and recent developments." *Philos Trans A Math Phys Eng Sci* 374 (2065):20150202. doi: 10.1098/rsta.2015.0202.

Key, M. 2012. "A tutorial in displaying mass spectrometry-based proteomic data using heat maps." *BMC Bioinformatics* 13 Suppl 16:S10. doi: 10.1186/1471-2105-13-S16-S10.

Kohonen, T. 1982. "Self-Organized Formation of Topologically Correct Feature Maps." *Biological Cybernetics* 43 (1):59-69. doi: 10.1007/bf00337288.

Kohonen, T. 2013. "Essentials of the self-organizing map." *Neural Netw* 37:52-65. doi: 10.1016/j.neunet.2012.09.018.

Legendre, A. M. 1805. *Nouvelle methodes pour la determination des orbites des cometes.* Paris: Firmin Didot.

Li, W. 2012. "Volcano plots in analyzing differential expressions with mRNA microarrays." *J Bioinform Comput Biol* 10 (6):1231003. doi: 10.1142/S0219720012310038.

Pearson, K. 1895. "Note on Regression and Inheritance in the Case of Two Parents." *Proceedings of the Royal Society of London* 58:240-242.

Pearson, K. 1901. "LIII. On lines and planes of closest fit to systems of points in space." *The London, Edinburgh, and Dublin Philosophical Magazine and Journal of Science.* 2 (11):559-572. doi: DOI: 10.1080/14786440109462720.

Pleil, J. D., M. A. Stiegel, M. C. Madden, and J. R. Sobus. 2011. "Heat map visualization of complex environmental and biomarker measurements." *Chemosphere* 84 (5):716-23. doi: 10.1016/j.chemosphere.2011.03.017.

Schober, P., C. Boer, and L. A. Schwarte. 2018. "Correlation Coefficients: Appropriate Use and Interpretation." *Anesth Analg* 126 (5):1763-1768. doi: 10.1213/ANE.0000000000002864.

Seltman, H. J. 2018. "Chapter 9: Simple linear regression." In *Experimental Design and Analysis*, 213-240. Carnegie Mellon University.

Sherlock, G. 2000. "Analysis of large-scale gene expression data." *Current Opinion in Immunology* 12 (2):201-205. doi: 10.1016/S0952-7915(99)00074-6.

3. MS/MS method parameters

Below are the parameters of the tandem methods tested. A summary of those parameters can also be found in Supplementary File SF2 as a table entitled "Summary of MS parameters across all 11 passes". Supplementary File SF2 also contains all the reject, parent and inclusion mass lists.

Pass 1 (wheat-mixed-digests_MS2_pass01_1.raw):

Creator: Orbi_30393
Last modified: 10/12/2021 by Orbi_30393
MS Run Time (min): 43.00
Sequence override of method parameters not enabled.
Divert Valve: not used during run
Contact Closure: not used during run
Syringe Pump: not used during run
MS Detector Settings:
Real-time modifications to method not enabled
Stepped collision energy not enabled
Additional Microscans:
MS2 0 0
MS3 0 0
MS4 0 0
MS5 0 0
MS6 0 0
MS7 0 0
MS8 0 0
MS9 0 0
MS10 0 0
Experiment Type: Nth Order Double Play
Tune Method: Orbitrap-tune-file_2020-03-13_HESI
Scan Event Details:
1: FTMS + p norm o(300.0-2000.0)
CV = 0.0V
2: ITMS + c norm Dep MS/MS Most intense ion from (1)
Activation Type: CID
Min. Signal Required: 3000.0
Isolation Width: 2.00
Normalized Coll. Energy: 35.0
Default Charge State: 2
Activation Q: 0.250
Activation Time: 10.000
CV = 0.0V
Scan Event 2 repeated for top 10 peaks.
Lock Masses:
Pos List Name: N/A
Source: API Source
Mass List: (none)
Neg List Name: N/A
Source: API Source
Mass List: (none)
Data Dependent Settings:
Use separate polarity settings disabled

Parent Mass List: (none)
Reject Mass List: (none)
Neutral Loss Mass List: (none)
Product Mass List: (none)
Neutral loss in top: 3
Product in top: 3
Most intense if no parent masses found not enabled
Add/subtract mass not enabled
FT master scan preview mode enabled
Charge state screening enabled
Charge state dependent ETD time not enabled
Monoisotopic precursor selection enabled
Charge state rejection enabled
Unassigned charge states : rejected
Charge state 1 : rejected
Charge state 2 : not rejected
Charge state 3 : not rejected
Charge states 4+ : not rejected
Chromatography mode is disabled
Global Data Dependent Settings:
Predict ion injection time enabled
Use global parent and reject mass lists not enabled
Exclude parent mass from data dependent selection not enabled
Exclusion mass width by mass
Exclusion mass width low: 1.50000
Exclusion mass width high: 1.50000
Parent mass width by mass
Parent mass width low: 0.50000
Parent mass width high: 0.50000
Reject mass width by mass
Reject mass width low: 0.50000
Reject mass width high: 0.50000
Zoom/UltraZoom scan mass width by mass
Zoom/UltraZoom scan mass width low: 5.00
Zoom/UltraZoom scan mass width high: 5.00
FT SIM scan mass width low: 5.00
FT SIM scan mass width high: 5.00
Neutral Loss candidates processed by decreasing intensity
Neutral Loss mass width by mass
Neutral Loss mass width low: 0.50000
Neutral Loss mass width high: 0.50000
Product candidates processed by decreasing intensity
Product mass width by mass
Product mass width low: 0.50000
Product mass width high: 0.50000
MS mass range: 300.00-2000.00
MSn mass range by mass
MSn mass range: 0.00-1000000.00
Use m/z values as masses not enabled
Analog UV data dep. not enabled
Dynamic exclusion enabled

Repeat Count: 1
Repeat Duration: 30.00
Exclusion List Size: 500
Exclusion Duration: 180.00
Exclusion mass width by mass
Exclusion mass width low: 1.50000
Exclusion mass width high: 1.50000
Expiration: disabled
Isotopic data dependence not enabled
Custom Data Dependent Settings:
Not enabled

Pass 2 (wheat-mixed-digests_MS2_pass02_1.raw):

Creator: Orbi_30393
Last modified: 10/12/2021 by Orbi_30393
MS Run Time (min): 43.00
Sequence override of method parameters not enabled.
Divert Valve: not used during run
Contact Closure: not used during run
Syringe Pump: not used during run
MS Detector Settings:
Real-time modifications to method not enabled
Stepped collision energy not enabled
Additional Microscans:
MS2 0 0
MS3 0 0
MS4 0 0
MS5 0 0
MS6 0 0
MS7 0 0
MS8 0 0
MS9 0 0
MS10 0 0
Experiment Type: Nth Order Double Play
Tune Method: Orbitrap-tune-file_2020-03-13_HESI
Scan Event Details:
1: FTMS + p norm o(300.0-2000.0)
CV = 0.0V
2: ITMS + c norm Dep MS/MS Most intense ion from (1)
Activation Type: CID
Min. Signal Required: 3000.0
Isolation Width: 2.00
Normalized Coll. Energy: 35.0
Default Charge State: 2
Activation Q: 0.250
Activation Time: 10.000
CV = 0.0V
Scan Event 2 repeated for top 10 peaks.
Lock Masses:
Pos List Name: N/A

Source: API Source
Mass List: (none)
Neg List Name: N/A
Source: API Source
Mass List: (none)
Data Dependent Settings:
Use separate polarity settings disabled
Parent Mass List: (none)
Reject Mass List:

MS Mass	Name
300.14000	exclusion0001
300.53000	exclusion0002
301.15000	exclusion0003
301.67000	exclusion0004
302.58000	exclusion0005
302.80000	exclusion0006
303.19000	exclusion0007
303.84000	exclusion0008
304.11000	exclusion0009
304.61000	exclusion0010
305.47000	exclusion0011
305.68000	exclusion0012
305.85000	exclusion0013
306.84000	exclusion0014
307.71000	exclusion0015
308.14000	exclusion0016
309.18000	exclusion0017
309.20000	exclusion0018
309.48000	exclusion0019
310.50000	exclusion0020
311.67000	exclusion0021
312.17000	exclusion0022
312.50000	exclusion0023
313.72000	exclusion0024
314.70000	exclusion0025
314.86000	exclusion0026
316.21000	exclusion0027
316.66000	exclusion0028
317.16000	exclusion0029
317.51000	exclusion0030
318.19000	exclusion0031
318.85000	exclusion0032
319.17000	exclusion0033
320.14000	exclusion0034
320.18000	exclusion0035
320.53000	exclusion0036
320.72000	exclusion0037
322.70000	exclusion0038
323.15000	exclusion0039
324.15000	exclusion0040
324.71000	exclusion0041

325.52000	exclusion0042
326.18000	exclusion0043
327.19000	exclusion0044
328.16000	exclusion0045
329.19000	exclusion0046
329.72000	exclusion0047
330.20000	exclusion0048
330.21000	exclusion0049
331.48000	exclusion0050
332.65000	exclusion0051
333.40000	exclusion0052
333.52000	exclusion0053
334.17000	exclusion0054
334.65000	exclusion0055
335.17000	exclusion0056
336.19000	exclusion0057
336.74000	exclusion0058
337.13000	exclusion0059
337.51000	exclusion0060
337.84000	exclusion0061
339.68000	exclusion0062
339.81000	exclusion0063
339.94000	exclusion0064
340.18000	exclusion0065
341.66000	exclusion0066
341.87000	exclusion0067
341.91000	exclusion0068
342.22000	exclusion0069
343.49000	exclusion0070
343.73000	exclusion0071
344.50000	exclusion0072
345.70000	exclusion0073
346.19000	exclusion0074
346.21000	exclusion0075
347.68000	exclusion0076
347.85000	exclusion0077
348.21000	exclusion0078
348.21000	exclusion0079
349.32000	exclusion0080
349.73000	exclusion0081
350.16000	exclusion0082
350.21000	exclusion0083
350.67000	exclusion0084
351.68000	exclusion0085
352.17000	exclusion0086
352.50000	exclusion0087
352.66000	exclusion0088
353.80000	exclusion0089
354.19000	exclusion0090
355.19000	exclusion0091
356.14000	exclusion0092

357.75000	exclusion0093
358.21000	exclusion0094
358.55000	exclusion0095
359.20000	exclusion0096
359.31000	exclusion0097
359.72000	exclusion0098
359.86000	exclusion0099
360.70000	exclusion0100
361.69000	exclusion0101
362.19000	exclusion0102
363.18000	exclusion0103
363.20000	exclusion0104
363.31000	exclusion0105
363.70000	exclusion0106
364.12000	exclusion0107
364.65000	exclusion0108
365.17000	exclusion0109
365.23000	exclusion0110
365.75000	exclusion0111
366.52000	exclusion0112
367.21000	exclusion0113
367.22000	exclusion0114
367.50000	exclusion0115
367.70000	exclusion0116
369.72000	exclusion0117
369.86000	exclusion0118
370.77000	exclusion0119
371.55000	exclusion0120
371.68000	exclusion0121
372.74000	exclusion0122
373.20000	exclusion0123
373.71000	exclusion0124
374.20000	exclusion0125
374.87000	exclusion0126
375.21000	exclusion0127
375.71000	exclusion0128
376.19000	exclusion0129
377.27000	exclusion0130
377.49000	exclusion0131
377.69000	exclusion0132
379.20000	exclusion0133
379.23000	exclusion0134
379.52000	exclusion0135
379.72000	exclusion0136
380.18000	exclusion0137
381.24000	exclusion0138
381.73000	exclusion0139
382.19000	exclusion0140
382.67000	exclusion0141
383.33000	exclusion0142
384.17000	exclusion0143

384.19000	exclusion0144
385.17000	exclusion0145
385.54000	exclusion0146
385.71000	exclusion0147
386.58000	exclusion0148
386.69000	exclusion0149
386.73000	exclusion0150
387.21000	exclusion0151
387.73000	exclusion0152
388.22000	exclusion0153
388.73000	exclusion0154
388.93000	exclusion0155
390.94000	exclusion0156
390.95000	exclusion0157
391.22000	exclusion0158
391.71000	exclusion0159
393.17000	exclusion0160
393.57000	exclusion0161
393.70000	exclusion0162
394.18000	exclusion0163
394.89000	exclusion0164
395.20000	exclusion0165
395.55000	exclusion0166
396.70000	exclusion0167
396.85000	exclusion0168
397.21000	exclusion0169
397.21000	exclusion0170
397.58000	exclusion0171
397.98000	exclusion0172
398.53000	exclusion0173
398.74000	exclusion0174
399.22000	exclusion0175
399.23000	exclusion0176
399.53000	exclusion0177
400.52000	exclusion0178
400.75000	exclusion0179
400.89000	exclusion0180
401.22000	exclusion0181
401.34000	exclusion0182
401.72000	exclusion0183
403.23000	exclusion0184
403.24000	exclusion0185
404.20000	exclusion0186
404.54000	exclusion0187
405.21000	exclusion0188
405.21000	exclusion0189
405.52000	exclusion0190
406.22000	exclusion0191
406.75000	exclusion0192
407.19000	exclusion0193
407.25000	exclusion0194

408.69000	exclusion0195
409.73000	exclusion0196
409.86000	exclusion0197
410.56000	exclusion0198
410.73000	exclusion0199
411.21000	exclusion0200
413.73000	exclusion0201
413.93000	exclusion0202
414.28000	exclusion0203
414.76000	exclusion0204
415.04000	exclusion0205
415.18000	exclusion0206
415.57000	exclusion0207
416.19000	exclusion0208
417.22000	exclusion0209
417.24000	exclusion0210
417.67000	exclusion0211
417.75000	exclusion0212
417.86000	exclusion0213
418.60000	exclusion0214
419.20000	exclusion0215
419.21000	exclusion0216
419.72000	exclusion0217
420.96000	exclusion0218
421.18000	exclusion0219
421.67000	exclusion0220
422.19000	exclusion0221
422.73000	exclusion0222
423.73000	exclusion0223
423.87000	exclusion0224
424.24000	exclusion0225
424.90000	exclusion0226
425.26000	exclusion0227
425.72000	exclusion0228
426.55000	exclusion0229
426.73000	exclusion0230
427.66000	exclusion0231
428.54000	exclusion0232
428.77000	exclusion0233
429.37000	exclusion0234
430.21000	exclusion0235
430.75000	exclusion0236
431.90000	exclusion0237
432.22000	exclusion0238
432.56000	exclusion0239
432.69000	exclusion0240
433.25000	exclusion0241
433.72000	exclusion0242
433.86000	exclusion0243
433.98000	exclusion0244
434.22000	exclusion0245

435.23000	exclusion0246
436.29000	exclusion0247
437.53000	exclusion0248
438.76000	exclusion0249
439.20000	exclusion0250
439.24000	exclusion0251
439.70000	exclusion0252
440.89000	exclusion0253
441.18000	exclusion0254
441.20000	exclusion0255
442.27000	exclusion0256
442.69000	exclusion0257
443.22000	exclusion0258
443.89000	exclusion0259
444.22000	exclusion0260
444.25000	exclusion0261
445.72000	exclusion0262
446.21000	exclusion0263
446.71000	exclusion0264
447.26000	exclusion0265
447.77000	exclusion0266
447.89000	exclusion0267
448.21000	exclusion0268
448.88000	exclusion0269
449.29000	exclusion0270
450.18000	exclusion0271
450.20000	exclusion0272
450.22000	exclusion0273
450.55000	exclusion0274
450.73000	exclusion0275
451.26000	exclusion0276
451.72000	exclusion0277
451.90000	exclusion0278
452.91000	exclusion0279
452.97000	exclusion0280
453.55000	exclusion0281
453.97000	exclusion0282
454.24000	exclusion0283
454.55000	exclusion0284
455.48000	exclusion0285
455.72000	exclusion0286
455.93000	exclusion0287
456.21000	exclusion0288
456.74000	exclusion0289
457.20000	exclusion0290
457.23000	exclusion0291
457.49000	exclusion0292
458.53000	exclusion0293
458.91000	exclusion0294
459.21000	exclusion0295
459.22000	exclusion0296

459.76000	exclusion0297
460.24000	exclusion0298
460.27000	exclusion0299
460.57000	exclusion0300
460.73000	exclusion0301
461.26000	exclusion0302
461.72000	exclusion0303
462.24000	exclusion0304
462.62000	exclusion0305
462.71000	exclusion0306
463.23000	exclusion0307
463.91000	exclusion0308
464.24000	exclusion0309
464.76000	exclusion0310
465.19000	exclusion0311
465.24000	exclusion0312
465.96000	exclusion0313
466.11000	exclusion0314
466.27000	exclusion0315
466.73000	exclusion0316
466.73000	exclusion0317
467.74000	exclusion0318
467.88000	exclusion0319
468.11000	exclusion0320
468.22000	exclusion0321
468.61000	exclusion0322
468.92000	exclusion0323
469.23000	exclusion0324
469.76000	exclusion0325
469.76000	exclusion0326
470.26000	exclusion0327
470.27000	exclusion0328
471.54000	exclusion0329
471.78000	exclusion0330
472.26000	exclusion0331
473.07000	exclusion0332
473.58000	exclusion0333
473.77000	exclusion0334
474.25000	exclusion0335
475.57000	exclusion0336
476.25000	exclusion0337
476.70000	exclusion0338
477.22000	exclusion0339
477.56000	exclusion0340
477.91000	exclusion0341
477.99000	exclusion0342
478.28000	exclusion0343
479.59000	exclusion0344
479.76000	exclusion0345
479.76000	exclusion0346
479.90000	exclusion0347

480.22000	exclusion0348
480.80000	exclusion0349
481.24000	exclusion0350
481.55000	exclusion0351
481.78000	exclusion0352
481.90000	exclusion0353
482.30000	exclusion0354
482.89000	exclusion0355
483.24000	exclusion0356
483.56000	exclusion0357
484.25000	exclusion0358
484.74000	exclusion0359
485.24000	exclusion0360
485.73000	exclusion0361
486.25000	exclusion0362
486.31000	exclusion0363
486.74000	exclusion0364
486.92000	exclusion0365
487.24000	exclusion0366
487.75000	exclusion0367
488.22000	exclusion0368
488.79000	exclusion0369
489.25000	exclusion0370
489.61000	exclusion0371
489.77000	exclusion0372
489.91000	exclusion0373
490.26000	exclusion0374
490.72000	exclusion0375
490.94000	exclusion0376
491.74000	exclusion0377
491.91000	exclusion0378
492.25000	exclusion0379
492.26000	exclusion0380
492.59000	exclusion0381
493.23000	exclusion0382
493.76000	exclusion0383
494.28000	exclusion0384
494.62000	exclusion0385
495.24000	exclusion0386
495.27000	exclusion0387
495.72000	exclusion0388
496.19000	exclusion0389
496.23000	exclusion0390
497.26000	exclusion0391
497.77000	exclusion0392
498.23000	exclusion0393
498.27000	exclusion0394
498.61000	exclusion0395
498.74000	exclusion0396
499.58000	exclusion0397
499.76000	exclusion0398

499.96000	exclusion0399
500.24000	exclusion0400
500.29000	exclusion0401
500.74000	exclusion0402
500.92000	exclusion0403
501.27000	exclusion0404
501.28000	exclusion0405
501.60000	exclusion0406
501.91000	exclusion0407
502.78000	exclusion0408
502.93000	exclusion0409
503.11000	exclusion0410
503.20000	exclusion0411
503.30000	exclusion0412
503.75000	exclusion0413
504.11000	exclusion0414
504.22000	exclusion0415
504.73000	exclusion0416
504.88000	exclusion0417
505.27000	exclusion0418
505.92000	exclusion0419
506.26000	exclusion0420
506.80000	exclusion0421
506.94000	exclusion0422
507.26000	exclusion0423
507.27000	exclusion0424
508.62000	exclusion0425
508.70000	exclusion0426
509.00000	exclusion0427
509.26000	exclusion0428
510.29000	exclusion0429
510.34000	exclusion0430
510.59000	exclusion0431
510.95000	exclusion0432
511.20000	exclusion0433
511.76000	exclusion0434
511.92000	exclusion0435
512.27000	exclusion0436
512.75000	exclusion0437
512.94000	exclusion0438
513.27000	exclusion0439
513.59000	exclusion0440
513.76000	exclusion0441
514.27000	exclusion0442
514.77000	exclusion0443
514.89000	exclusion0444
515.29000	exclusion0445
515.73000	exclusion0446
515.82000	exclusion0447
516.80000	exclusion0448
516.96000	exclusion0449

517.24000	exclusion0450
517.26000	exclusion0451
517.62000	exclusion0452
517.77000	exclusion0453
518.29000	exclusion0454
519.14000	exclusion0455
519.56000	exclusion0456
519.78000	exclusion0457
519.79000	exclusion0458
519.92000	exclusion0459
520.26000	exclusion0460
520.62000	exclusion0461
520.78000	exclusion0462
521.53000	exclusion0463
522.22000	exclusion0464
522.28000	exclusion0465
522.77000	exclusion0466
523.27000	exclusion0467
523.82000	exclusion0468
523.99000	exclusion0469
524.22000	exclusion0470
524.59000	exclusion0471
524.77000	exclusion0472
524.93000	exclusion0473
525.26000	exclusion0474
525.62000	exclusion0475
525.96000	exclusion0476
526.52000	exclusion0477
526.60000	exclusion0478
526.92000	exclusion0479
527.27000	exclusion0480
527.81000	exclusion0481
528.23000	exclusion0482
528.73000	exclusion0483
529.26000	exclusion0484
530.25000	exclusion0485
530.74000	exclusion0486
531.29000	exclusion0487
531.59000	exclusion0488
532.25000	exclusion0489
532.25000	exclusion0490
533.28000	exclusion0491
533.35000	exclusion0492
533.53000	exclusion0493
533.53000	exclusion0494
533.82000	exclusion0495
534.23000	exclusion0496
534.27000	exclusion0497
534.94000	exclusion0498
535.82000	exclusion0499
535.92000	exclusion0500

536.09000	exclusion0501
536.26000	exclusion0502
536.28000	exclusion0503
537.24000	exclusion0504
537.79000	exclusion0505
537.97000	exclusion0506
538.29000	exclusion0507
538.59000	exclusion0508
538.70000	exclusion0509
538.77000	exclusion0510
538.80000	exclusion0511
540.27000	exclusion0512
540.79000	exclusion0513
541.23000	exclusion0514
541.26000	exclusion0515
541.62000	exclusion0516
541.92000	exclusion0517
542.31000	exclusion0518
542.58000	exclusion0519
543.27000	exclusion0520
543.28000	exclusion0521
543.76000	exclusion0522
544.01000	exclusion0523
544.22000	exclusion0524
545.28000	exclusion0525
545.67000	exclusion0526
545.77000	exclusion0527
546.24000	exclusion0528
546.96000	exclusion0529
547.28000	exclusion0530
547.77000	exclusion0531
548.77000	exclusion0532
548.79000	exclusion0533
549.04000	exclusion0534
549.28000	exclusion0535
549.49000	exclusion0536
550.31000	exclusion0537
550.33000	exclusion0538
550.78000	exclusion0539
551.28000	exclusion0540
551.29000	exclusion0541
551.60000	exclusion0542
551.89000	exclusion0543
552.04000	exclusion0544
552.29000	exclusion0545
552.30000	exclusion0546
552.74000	exclusion0547
553.28000	exclusion0548
553.77000	exclusion0549
554.37000	exclusion0550
554.77000	exclusion0551

555.28000	exclusion0552
555.72000	exclusion0553
556.30000	exclusion0554
556.94000	exclusion0555
557.26000	exclusion0556
557.30000	exclusion0557
557.83000	exclusion0558
558.07000	exclusion0559
558.28000	exclusion0560
558.77000	exclusion0561
559.27000	exclusion0562
559.80000	exclusion0563
560.28000	exclusion0564
560.49000	exclusion0565
560.66000	exclusion0566
560.77000	exclusion0567
561.30000	exclusion0568
562.27000	exclusion0569
562.31000	exclusion0570
562.81000	exclusion0571
563.08000	exclusion0572
563.29000	exclusion0573
563.95000	exclusion0574
564.78000	exclusion0575
565.31000	exclusion0576
565.34000	exclusion0577
566.27000	exclusion0578
566.77000	exclusion0579
567.03000	exclusion0580
567.29000	exclusion0581
567.59000	exclusion0582
567.83000	exclusion0583
567.93000	exclusion0584
568.28000	exclusion0585
569.24000	exclusion0586
569.30000	exclusion0587
569.81000	exclusion0588
570.26000	exclusion0589
570.32000	exclusion0590
571.33000	exclusion0591
571.62000	exclusion0592
571.76000	exclusion0593
572.26000	exclusion0594
572.50000	exclusion0595
572.67000	exclusion0596
573.25000	exclusion0597
573.30000	exclusion0598
573.81000	exclusion0599
573.97000	exclusion0600
574.28000	exclusion0601
574.79000	exclusion0602

574.80000	exclusion0603
575.18000	exclusion0604
575.30000	exclusion0605
575.66000	exclusion0606
575.77000	exclusion0607
576.41000	exclusion0608
576.66000	exclusion0609
576.71000	exclusion0610
577.30000	exclusion0611
577.81000	exclusion0612
578.29000	exclusion0613
578.31000	exclusion0614
578.77000	exclusion0615
578.96000	exclusion0616
579.11000	exclusion0617
579.29000	exclusion0618
579.59000	exclusion0619
579.75000	exclusion0620
579.84000	exclusion0621
580.34000	exclusion0622
580.79000	exclusion0623
581.25000	exclusion0624
581.36000	exclusion0625
581.79000	exclusion0626
582.31000	exclusion0627
582.74000	exclusion0628
583.10000	exclusion0629
583.79000	exclusion0630
583.95000	exclusion0631
584.38000	exclusion0632
584.55000	exclusion0633
584.68000	exclusion0634
584.76000	exclusion0635
585.83000	exclusion0636
586.06000	exclusion0637
586.28000	exclusion0638
586.30000	exclusion0639
586.81000	exclusion0640
587.81000	exclusion0641
588.29000	exclusion0642
588.30000	exclusion0643
588.80000	exclusion0644
589.31000	exclusion0645
590.25000	exclusion0646
590.51000	exclusion0647
590.76000	exclusion0648
591.09000	exclusion0649
591.29000	exclusion0650
591.79000	exclusion0651
592.64000	exclusion0652
592.76000	exclusion0653

593.16000	exclusion0654
593.32000	exclusion0655
593.36000	exclusion0656
593.81000	exclusion0657
594.76000	exclusion0658
594.92000	exclusion0659
595.33000	exclusion0660
595.92000	exclusion0661
596.32000	exclusion0662
596.77000	exclusion0663
597.28000	exclusion0664
597.31000	exclusion0665
597.56000	exclusion0666
597.85000	exclusion0667
598.28000	exclusion0668
598.40000	exclusion0669
598.97000	exclusion0670
599.66000	exclusion0671
599.78000	exclusion0672
600.26000	exclusion0673
601.32000	exclusion0674
601.62000	exclusion0675
601.77000	exclusion0676
601.84000	exclusion0677
603.29000	exclusion0678
603.29000	exclusion0679
603.65000	exclusion0680
603.79000	exclusion0681
604.28000	exclusion0682
605.29000	exclusion0683
605.78000	exclusion0684
606.28000	exclusion0685
606.29000	exclusion0686
606.31000	exclusion0687
606.62000	exclusion0688
607.81000	exclusion0689
608.29000	exclusion0690
608.31000	exclusion0691
609.31000	exclusion0692
609.33000	exclusion0693
610.36000	exclusion0694
610.78000	exclusion0695
611.00000	exclusion0696
611.27000	exclusion0697
611.30000	exclusion0698
611.31000	exclusion0699
611.81000	exclusion0700
612.95000	exclusion0701
613.31000	exclusion0702
613.32000	exclusion0703
613.79000	exclusion0704

614.09000	exclusion0705
614.48000	exclusion0706
614.82000	exclusion0707
614.96000	exclusion0708
615.29000	exclusion0709
615.83000	exclusion0710
616.30000	exclusion0711
616.66000	exclusion0712
616.79000	exclusion0713
616.99000	exclusion0714
617.34000	exclusion0715
617.80000	exclusion0716
617.80000	exclusion0717
618.29000	exclusion0718
618.93000	exclusion0719
619.13000	exclusion0720
619.30000	exclusion0721
619.63000	exclusion0722
619.85000	exclusion0723
620.02000	exclusion0724
620.30000	exclusion0725
620.83000	exclusion0726
621.33000	exclusion0727
621.79000	exclusion0728
621.98000	exclusion0729
622.34000	exclusion0730
622.66000	exclusion0731
622.79000	exclusion0732
623.24000	exclusion0733
623.33000	exclusion0734
623.78000	exclusion0735
624.34000	exclusion0736
624.77000	exclusion0737
625.04000	exclusion0738
625.33000	exclusion0739
625.33000	exclusion0740
625.93000	exclusion0741
626.28000	exclusion0742
626.81000	exclusion0743
627.01000	exclusion0744
627.30000	exclusion0745
628.29000	exclusion0746
628.58000	exclusion0747
628.80000	exclusion0748
628.96000	exclusion0749
629.03000	exclusion0750
629.82000	exclusion0751
630.30000	exclusion0752
630.77000	exclusion0753
631.31000	exclusion0754
631.83000	exclusion0755

632.29000	exclusion0756
632.83000	exclusion0757
633.28000	exclusion0758
633.31000	exclusion0759
633.83000	exclusion0760
634.33000	exclusion0761
634.79000	exclusion0762
635.86000	exclusion0763
636.32000	exclusion0764
636.83000	exclusion0765
637.64000	exclusion0766
637.78000	exclusion0767
637.85000	exclusion0768
638.23000	exclusion0769
638.26000	exclusion0770
638.85000	exclusion0771
639.35000	exclusion0772
639.59000	exclusion0773
639.61000	exclusion0774
639.78000	exclusion0775
639.92000	exclusion0776
640.30000	exclusion0777
640.61000	exclusion0778
641.35000	exclusion0779
641.44000	exclusion0780
641.84000	exclusion0781
642.86000	exclusion0782
643.29000	exclusion0783
643.36000	exclusion0784
643.82000	exclusion0785
644.02000	exclusion0786
644.87000	exclusion0787
645.02000	exclusion0788
645.35000	exclusion0789
645.51000	exclusion0790
645.70000	exclusion0791
645.98000	exclusion0792
647.31000	exclusion0793
647.35000	exclusion0794
647.87000	exclusion0795
648.00000	exclusion0796
648.29000	exclusion0797
648.30000	exclusion0798
648.31000	exclusion0799
648.82000	exclusion0800
649.28000	exclusion0801
649.82000	exclusion0802
650.29000	exclusion0803
650.82000	exclusion0804
651.30000	exclusion0805
651.32000	exclusion0806

651.78000	exclusion0807
652.84000	exclusion0808
653.24000	exclusion0809
653.36000	exclusion0810
653.63000	exclusion0811
654.30000	exclusion0812
654.35000	exclusion0813
654.85000	exclusion0814
655.09000	exclusion0815
655.82000	exclusion0816
656.32000	exclusion0817
656.54000	exclusion0818
657.03000	exclusion0819
657.35000	exclusion0820
657.38000	exclusion0821
657.83000	exclusion0822
658.03000	exclusion0823
658.32000	exclusion0824
658.35000	exclusion0825
659.28000	exclusion0826
659.34000	exclusion0827
659.78000	exclusion0828
660.30000	exclusion0829
660.35000	exclusion0830
660.66000	exclusion0831
661.30000	exclusion0832
661.81000	exclusion0833
662.32000	exclusion0834
662.83000	exclusion0835
663.32000	exclusion0836
663.75000	exclusion0837
664.11000	exclusion0838
664.37000	exclusion0839
664.66000	exclusion0840
665.32000	exclusion0841
665.34000	exclusion0842
665.64000	exclusion0843
665.87000	exclusion0844
666.34000	exclusion0845
666.86000	exclusion0846
667.03000	exclusion0847
667.36000	exclusion0848
667.52000	exclusion0849
668.66000	exclusion0850
668.83000	exclusion0851
669.34000	exclusion0852
669.51000	exclusion0853
669.85000	exclusion0854
669.90000	exclusion0855
670.29000	exclusion0856
670.80000	exclusion0857

671.83000	exclusion0858
672.32000	exclusion0859
672.82000	exclusion0860
673.11000	exclusion0861
673.33000	exclusion0862
673.34000	exclusion0863
674.31000	exclusion0864
674.33000	exclusion0865
674.58000	exclusion0866
674.83000	exclusion0867
675.19000	exclusion0868
676.05000	exclusion0869
676.85000	exclusion0870
677.00000	exclusion0871
677.28000	exclusion0872
677.34000	exclusion0873
677.85000	exclusion0874
677.88000	exclusion0875
678.36000	exclusion0876
679.04000	exclusion0877
679.31000	exclusion0878
679.85000	exclusion0879
680.33000	exclusion0880
680.78000	exclusion0881
681.32000	exclusion0882
682.37000	exclusion0883
682.67000	exclusion0884
682.82000	exclusion0885
683.32000	exclusion0886
683.37000	exclusion0887
683.97000	exclusion0888
684.29000	exclusion0889
684.78000	exclusion0890
685.67000	exclusion0891
685.83000	exclusion0892
686.31000	exclusion0893
686.34000	exclusion0894
686.85000	exclusion0895
687.30000	exclusion0896
687.33000	exclusion0897
687.68000	exclusion0898
688.78000	exclusion0899
689.00000	exclusion0900
689.34000	exclusion0901
689.37000	exclusion0902
689.67000	exclusion0903
690.35000	exclusion0904
690.72000	exclusion0905
691.32000	exclusion0906
691.36000	exclusion0907
692.02000	exclusion0908

692.26000	exclusion0909
692.70000	exclusion0910
693.10000	exclusion0911
693.56000	exclusion0912
693.88000	exclusion0913
694.31000	exclusion0914
695.33000	exclusion0915
695.39000	exclusion0916
695.85000	exclusion0917
696.33000	exclusion0918
696.35000	exclusion0919
697.01000	exclusion0920
697.36000	exclusion0921
697.83000	exclusion0922
698.37000	exclusion0923
698.89000	exclusion0924
699.12000	exclusion0925
699.37000	exclusion0926
699.38000	exclusion0927
699.68000	exclusion0928
700.37000	exclusion0929
700.90000	exclusion0930
701.33000	exclusion0931
702.31000	exclusion0932
702.33000	exclusion0933
702.77000	exclusion0934
702.87000	exclusion0935
703.38000	exclusion0936
703.71000	exclusion0937
704.31000	exclusion0938
705.27000	exclusion0939
705.37000	exclusion0940
705.82000	exclusion0941
706.81000	exclusion0942
707.02000	exclusion0943
707.32000	exclusion0944
708.04000	exclusion0945
708.33000	exclusion0946
708.38000	exclusion0947
708.85000	exclusion0948
709.35000	exclusion0949
709.35000	exclusion0950
709.90000	exclusion0951
710.33000	exclusion0952
710.36000	exclusion0953
710.81000	exclusion0954
712.05000	exclusion0955
712.34000	exclusion0956
712.35000	exclusion0957
712.59000	exclusion0958
712.70000	exclusion0959

712.80000	exclusion0960
713.09000	exclusion0961
713.34000	exclusion0962
713.68000	exclusion0963
713.89000	exclusion0964
714.35000	exclusion0965
714.40000	exclusion0966
715.01000	exclusion0967
715.36000	exclusion0968
715.84000	exclusion0969
716.31000	exclusion0970
716.87000	exclusion0971
717.83000	exclusion0972
718.31000	exclusion0973
718.34000	exclusion0974
719.35000	exclusion0975
719.71000	exclusion0976
719.81000	exclusion0977
720.32000	exclusion0978
720.90000	exclusion0979
721.37000	exclusion0980
721.41000	exclusion0981
721.73000	exclusion0982
721.79000	exclusion0983
722.59000	exclusion0984
722.87000	exclusion0985
723.07000	exclusion0986
723.33000	exclusion0987
723.40000	exclusion0988
724.88000	exclusion0989
725.04000	exclusion0990
725.38000	exclusion0991
725.82000	exclusion0992
726.05000	exclusion0993
726.72000	exclusion0994
726.81000	exclusion0995
727.37000	exclusion0996
727.50000	exclusion0997
728.02000	exclusion0998
728.32000	exclusion0999
728.88000	exclusion1000
729.32000	exclusion1001
729.86000	exclusion1002
730.36000	exclusion1003
730.36000	exclusion1004
730.64000	exclusion1005
730.87000	exclusion1006
731.01000	exclusion1007
731.88000	exclusion1008
732.35000	exclusion1009
732.84000	exclusion1010

733.37000	exclusion1011
733.86000	exclusion1012
734.32000	exclusion1013
734.36000	exclusion1014
735.33000	exclusion1015
735.90000	exclusion1016
736.37000	exclusion1017
736.38000	exclusion1018
736.86000	exclusion1019
737.36000	exclusion1020
737.88000	exclusion1021
738.04000	exclusion1022
738.38000	exclusion1023
739.35000	exclusion1024
739.86000	exclusion1025
740.28000	exclusion1026
740.87000	exclusion1027
741.37000	exclusion1028
742.37000	exclusion1029
742.83000	exclusion1030
743.06000	exclusion1031
743.38000	exclusion1032
743.85000	exclusion1033
744.25000	exclusion1034
744.38000	exclusion1035
744.86000	exclusion1036
746.28000	exclusion1037
746.72000	exclusion1038
747.03000	exclusion1039
747.73000	exclusion1040
747.83000	exclusion1041
748.07000	exclusion1042
748.32000	exclusion1043
748.85000	exclusion1044
749.04000	exclusion1045
749.38000	exclusion1046
749.61000	exclusion1047
750.36000	exclusion1048
750.37000	exclusion1049
750.87000	exclusion1050
751.37000	exclusion1051
751.38000	exclusion1052
751.86000	exclusion1053
752.11000	exclusion1054
752.36000	exclusion1055
753.10000	exclusion1056
753.37000	exclusion1057
753.38000	exclusion1058
754.32000	exclusion1059
754.83000	exclusion1060
755.34000	exclusion1061

755.73000	exclusion1062
755.86000	exclusion1063
756.08000	exclusion1064
756.32000	exclusion1065
756.71000	exclusion1066
757.36000	exclusion1067
757.40000	exclusion1068
757.70000	exclusion1069
757.88000	exclusion1070
758.84000	exclusion1071
758.89000	exclusion1072
759.37000	exclusion1073
759.39000	exclusion1074
759.68000	exclusion1075
759.86000	exclusion1076
760.36000	exclusion1077
760.41000	exclusion1078
760.73000	exclusion1079
760.91000	exclusion1080
761.34000	exclusion1081
761.90000	exclusion1082
762.40000	exclusion1083
762.40000	exclusion1084
762.88000	exclusion1085
763.34000	exclusion1086
763.90000	exclusion1087
764.21000	exclusion1088
764.36000	exclusion1089
764.73000	exclusion1090
765.86000	exclusion1091
766.05000	exclusion1092
766.35000	exclusion1093
766.39000	exclusion1094
767.87000	exclusion1095
768.35000	exclusion1096
768.66000	exclusion1097
768.90000	exclusion1098
769.31000	exclusion1099
769.90000	exclusion1100
770.37000	exclusion1101
770.86000	exclusion1102
771.02000	exclusion1103
771.38000	exclusion1104
771.88000	exclusion1105
773.36000	exclusion1106
773.37000	exclusion1107
773.87000	exclusion1108
774.30000	exclusion1109
774.61000	exclusion1110
775.34000	exclusion1111
775.39000	exclusion1112

775.83000	exclusion1113
776.37000	exclusion1114
776.58000	exclusion1115
776.86000	exclusion1116
777.39000	exclusion1117
777.40000	exclusion1118
777.83000	exclusion1119
778.40000	exclusion1120
778.91000	exclusion1121
779.11000	exclusion1122
779.34000	exclusion1123
779.37000	exclusion1124
779.86000	exclusion1125
780.75000	exclusion1126
780.81000	exclusion1127
781.08000	exclusion1128
781.16000	exclusion1129
781.39000	exclusion1130
782.28000	exclusion1131
783.30000	exclusion1132
783.41000	exclusion1133
784.35000	exclusion1134
784.87000	exclusion1135
785.30000	exclusion1136
785.65000	exclusion1137
786.39000	exclusion1138
786.88000	exclusion1139
787.10000	exclusion1140
787.36000	exclusion1141
787.90000	exclusion1142
788.71000	exclusion1143
789.04000	exclusion1144
789.37000	exclusion1145
790.36000	exclusion1146
790.38000	exclusion1147
791.06000	exclusion1148
791.37000	exclusion1149
792.29000	exclusion1150
792.38000	exclusion1151
792.91000	exclusion1152
792.91000	exclusion1153
793.38000	exclusion1154
793.87000	exclusion1155
793.94000	exclusion1156
794.38000	exclusion1157
794.40000	exclusion1158
795.36000	exclusion1159
795.42000	exclusion1160
795.90000	exclusion1161
796.12000	exclusion1162
796.37000	exclusion1163

796.40000	exclusion1164
796.88000	exclusion1165
797.43000	exclusion1166
797.87000	exclusion1167
798.35000	exclusion1168
798.41000	exclusion1169
799.13000	exclusion1170
799.87000	exclusion1171
800.35000	exclusion1172
800.38000	exclusion1173
800.68000	exclusion1174
801.38000	exclusion1175
801.38000	exclusion1176
801.95000	exclusion1177
802.27000	exclusion1178
802.39000	exclusion1179
802.69000	exclusion1180
802.82000	exclusion1181
803.92000	exclusion1182
804.40000	exclusion1183
804.41000	exclusion1184
804.74000	exclusion1185
805.00000	exclusion1186
805.40000	exclusion1187
806.39000	exclusion1188
806.43000	exclusion1189
806.88000	exclusion1190
807.02000	exclusion1191
808.10000	exclusion1192
808.36000	exclusion1193
808.36000	exclusion1194
808.94000	exclusion1195
809.40000	exclusion1196
809.91000	exclusion1197
810.39000	exclusion1198
810.39000	exclusion1199
810.75000	exclusion1200
811.36000	exclusion1201
812.38000	exclusion1202
812.73000	exclusion1203
813.37000	exclusion1204
813.43000	exclusion1205
815.36000	exclusion1206
815.41000	exclusion1207
815.93000	exclusion1208
817.05000	exclusion1209
817.33000	exclusion1210
817.75000	exclusion1211
817.86000	exclusion1212
818.43000	exclusion1213
818.55000	exclusion1214

818.87000	exclusion1215
819.04000	exclusion1216
819.42000	exclusion1217
820.37000	exclusion1218
821.15000	exclusion1219
821.39000	exclusion1220
822.38000	exclusion1221
822.39000	exclusion1222
822.91000	exclusion1223
822.92000	exclusion1224
823.57000	exclusion1225
824.40000	exclusion1226
824.81000	exclusion1227
825.40000	exclusion1228
825.85000	exclusion1229
826.22000	exclusion1230
826.38000	exclusion1231
826.44000	exclusion1232
827.42000	exclusion1233
827.42000	exclusion1234
827.89000	exclusion1235
828.41000	exclusion1236
828.44000	exclusion1237
829.41000	exclusion1238
829.43000	exclusion1239
830.43000	exclusion1240
830.92000	exclusion1241
830.96000	exclusion1242
831.13000	exclusion1243
831.32000	exclusion1244
831.94000	exclusion1245
832.41000	exclusion1246
832.44000	exclusion1247
832.88000	exclusion1248
833.15000	exclusion1249
833.40000	exclusion1250
833.78000	exclusion1251
834.16000	exclusion1252
834.33000	exclusion1253
834.43000	exclusion1254
834.92000	exclusion1255
835.04000	exclusion1256
835.13000	exclusion1257
836.41000	exclusion1258
836.42000	exclusion1259
837.42000	exclusion1260
837.44000	exclusion1261
837.76000	exclusion1262
837.93000	exclusion1263
838.42000	exclusion1264
839.16000	exclusion1265

839.25000	exclusion1266
839.43000	exclusion1267
839.91000	exclusion1268
840.42000	exclusion1269
840.67000	exclusion1270
840.73000	exclusion1271
841.44000	exclusion1272
841.45000	exclusion1273
842.91000	exclusion1274
842.92000	exclusion1275
843.40000	exclusion1276
843.64000	exclusion1277
843.91000	exclusion1278
843.94000	exclusion1279
845.41000	exclusion1280
845.87000	exclusion1281
846.16000	exclusion1282
846.36000	exclusion1283
847.37000	exclusion1284
847.39000	exclusion1285
847.88000	exclusion1286
848.36000	exclusion1287
849.41000	exclusion1288
849.44000	exclusion1289
849.89000	exclusion1290
849.90000	exclusion1291
850.42000	exclusion1292
850.91000	exclusion1293
851.17000	exclusion1294
851.42000	exclusion1295
851.45000	exclusion1296
851.78000	exclusion1297
852.38000	exclusion1298
852.39000	exclusion1299
852.88000	exclusion1300
853.23000	exclusion1301
853.35000	exclusion1302
853.66000	exclusion1303
854.38000	exclusion1304
855.37000	exclusion1305
855.40000	exclusion1306
855.88000	exclusion1307
856.15000	exclusion1308
856.40000	exclusion1309
857.06000	exclusion1310
857.45000	exclusion1311
857.89000	exclusion1312
858.50000	exclusion1313
858.74000	exclusion1314
859.47000	exclusion1315
859.64000	exclusion1316

860.08000	exclusion1317
860.39000	exclusion1318
860.88000	exclusion1319
861.44000	exclusion1320
861.90000	exclusion1321
862.46000	exclusion1322
862.49000	exclusion1323
862.90000	exclusion1324
863.39000	exclusion1325
863.41000	exclusion1326
864.44000	exclusion1327
864.45000	exclusion1328
864.88000	exclusion1329
865.91000	exclusion1330
866.41000	exclusion1331
866.42000	exclusion1332
867.45000	exclusion1333
867.75000	exclusion1334
868.13000	exclusion1335
868.40000	exclusion1336
868.40000	exclusion1337
868.90000	exclusion1338
868.94000	exclusion1339
869.22000	exclusion1340
869.73000	exclusion1341
870.18000	exclusion1342
870.42000	exclusion1343
870.43000	exclusion1344
871.35000	exclusion1345
872.38000	exclusion1346
872.43000	exclusion1347
872.70000	exclusion1348
872.92000	exclusion1349
873.45000	exclusion1350
874.05000	exclusion1351
874.42000	exclusion1352
874.95000	exclusion1353
875.39000	exclusion1354
875.90000	exclusion1355
876.01000	exclusion1356
876.40000	exclusion1357
876.40000	exclusion1358
877.36000	exclusion1359
877.40000	exclusion1360
878.05000	exclusion1361
878.48000	exclusion1362
879.05000	exclusion1363
879.41000	exclusion1364
879.93000	exclusion1365
880.40000	exclusion1366
880.41000	exclusion1367

880.73000	exclusion1368
881.43000	exclusion1369
882.44000	exclusion1370
882.46000	exclusion1371
882.91000	exclusion1372
883.44000	exclusion1373
884.41000	exclusion1374
884.47000	exclusion1375
884.89000	exclusion1376
885.42000	exclusion1377
885.45000	exclusion1378
885.93000	exclusion1379
886.40000	exclusion1380
886.95000	exclusion1381
887.48000	exclusion1382
887.96000	exclusion1383
888.40000	exclusion1384
888.42000	exclusion1385
888.74000	exclusion1386
889.47000	exclusion1387
890.41000	exclusion1388
890.66000	exclusion1389
890.88000	exclusion1390
891.41000	exclusion1391
891.90000	exclusion1392
892.24000	exclusion1393
892.45000	exclusion1394
893.44000	exclusion1395
893.89000	exclusion1396
894.18000	exclusion1397
894.44000	exclusion1398
895.42000	exclusion1399
895.48000	exclusion1400
895.89000	exclusion1401
896.16000	exclusion1402
896.43000	exclusion1403
896.45000	exclusion1404
897.41000	exclusion1405
898.10000	exclusion1406
898.42000	exclusion1407
898.82000	exclusion1408
899.44000	exclusion1409
899.45000	exclusion1410
900.42000	exclusion1411
900.43000	exclusion1412
901.14000	exclusion1413
901.45000	exclusion1414
902.42000	exclusion1415
902.70000	exclusion1416
903.13000	exclusion1417
903.41000	exclusion1418

904.79000	exclusion1419
905.13000	exclusion1420
905.40000	exclusion1421
905.96000	exclusion1422
906.41000	exclusion1423
907.42000	exclusion1424
907.45000	exclusion1425
907.96000	exclusion1426
908.40000	exclusion1427
909.44000	exclusion1428
909.44000	exclusion1429
909.94000	exclusion1430
911.13000	exclusion1431
911.46000	exclusion1432
911.69000	exclusion1433
911.94000	exclusion1434
911.97000	exclusion1435
912.41000	exclusion1436
913.66000	exclusion1437
913.70000	exclusion1438
913.92000	exclusion1439
914.43000	exclusion1440
914.47000	exclusion1441
915.12000	exclusion1442
915.45000	exclusion1443
915.95000	exclusion1444
916.46000	exclusion1445
916.49000	exclusion1446
916.75000	exclusion1447
917.08000	exclusion1448
917.47000	exclusion1449
917.93000	exclusion1450
918.11000	exclusion1451
918.46000	exclusion1452
918.48000	exclusion1453
918.92000	exclusion1454
919.43000	exclusion1455
919.54000	exclusion1456
920.42000	exclusion1457
920.49000	exclusion1458
920.87000	exclusion1459
921.40000	exclusion1460
921.47000	exclusion1461
922.30000	exclusion1462
922.47000	exclusion1463
922.90000	exclusion1464
923.46000	exclusion1465
923.91000	exclusion1466
924.13000	exclusion1467
924.43000	exclusion1468
924.94000	exclusion1469

925.49000	exclusion1470
926.46000	exclusion1471
926.79000	exclusion1472
927.82000	exclusion1473
927.88000	exclusion1474
928.47000	exclusion1475
928.93000	exclusion1476
929.45000	exclusion1477
929.77000	exclusion1478
930.46000	exclusion1479
930.47000	exclusion1480
930.68000	exclusion1481
931.11000	exclusion1482
931.40000	exclusion1483
931.41000	exclusion1484
931.81000	exclusion1485
932.43000	exclusion1486
933.47000	exclusion1487
933.79000	exclusion1488
934.39000	exclusion1489
934.97000	exclusion1490
935.16000	exclusion1491
935.47000	exclusion1492
935.91000	exclusion1493
936.11000	exclusion1494
937.49000	exclusion1495
937.52000	exclusion1496
937.79000	exclusion1497
937.99000	exclusion1498
938.49000	exclusion1499
939.21000	exclusion1500
939.49000	exclusion1501
939.93000	exclusion1502
941.46000	exclusion1503
942.47000	exclusion1504
942.98000	exclusion1505
943.10000	exclusion1506
943.47000	exclusion1507
943.73000	exclusion1508
944.47000	exclusion1509
944.74000	exclusion1510
944.92000	exclusion1511
945.48000	exclusion1512
945.78000	exclusion1513
945.98000	exclusion1514
946.95000	exclusion1515
947.49000	exclusion1516
947.96000	exclusion1517
948.46000	exclusion1518
949.02000	exclusion1519
949.46000	exclusion1520

949.80000	exclusion1521
950.01000	exclusion1522
950.70000	exclusion1523
951.13000	exclusion1524
951.41000	exclusion1525
951.47000	exclusion1526
951.82000	exclusion1527
952.14000	exclusion1528
952.77000	exclusion1529
952.96000	exclusion1530
953.01000	exclusion1531
953.48000	exclusion1532
954.44000	exclusion1533
954.94000	exclusion1534
955.35000	exclusion1535
955.57000	exclusion1536
956.47000	exclusion1537
957.43000	exclusion1538
957.74000	exclusion1539
958.44000	exclusion1540
958.81000	exclusion1541
959.41000	exclusion1542
960.45000	exclusion1543
960.75000	exclusion1544
961.00000	exclusion1545
961.35000	exclusion1546
962.49000	exclusion1547
962.87000	exclusion1548
963.48000	exclusion1549
963.94000	exclusion1550
964.44000	exclusion1551
964.74000	exclusion1552
964.98000	exclusion1553
965.02000	exclusion1554
965.48000	exclusion1555
966.26000	exclusion1556
966.48000	exclusion1557
967.21000	exclusion1558
967.48000	exclusion1559
967.48000	exclusion1560
967.90000	exclusion1561
968.47000	exclusion1562
969.50000	exclusion1563
969.98000	exclusion1564
970.11000	exclusion1565
970.45000	exclusion1566
970.52000	exclusion1567
971.48000	exclusion1568
971.96000	exclusion1569
972.15000	exclusion1570
972.44000	exclusion1571

972.48000	exclusion1572
972.54000	exclusion1573
973.00000	exclusion1574
973.80000	exclusion1575
974.47000	exclusion1576
975.47000	exclusion1577
975.49000	exclusion1578
975.82000	exclusion1579
976.68000	exclusion1580
977.14000	exclusion1581
977.49000	exclusion1582
977.78000	exclusion1583
978.46000	exclusion1584
978.51000	exclusion1585
979.42000	exclusion1586
980.13000	exclusion1587
980.51000	exclusion1588
980.97000	exclusion1589
981.21000	exclusion1590
981.30000	exclusion1591
981.54000	exclusion1592
982.46000	exclusion1593
983.45000	exclusion1594
983.49000	exclusion1595
984.48000	exclusion1596
984.50000	exclusion1597
984.94000	exclusion1598
985.87000	exclusion1599
986.00000	exclusion1600
986.47000	exclusion1601
986.83000	exclusion1602
987.01000	exclusion1603
987.51000	exclusion1604
987.97000	exclusion1605
988.18000	exclusion1606
988.49000	exclusion1607
989.01000	exclusion1608
990.02000	exclusion1609
990.14000	exclusion1610
990.47000	exclusion1611
990.50000	exclusion1612
991.16000	exclusion1613
991.20000	exclusion1614
991.48000	exclusion1615
991.83000	exclusion1616
991.99000	exclusion1617
993.52000	exclusion1618
993.96000	exclusion1619
995.49000	exclusion1620
996.48000	exclusion1621
997.50000	exclusion1622

998.48000	exclusion1623
998.81000	exclusion1624
999.39000	exclusion1625
999.82000	exclusion1626
1000.46000	exclusion1627
1000.84000	exclusion1628
1001.50000	exclusion1629
1002.48000	exclusion1630
1003.01000	exclusion1631
1003.53000	exclusion1632
1004.48000	exclusion1633
1004.94000	exclusion1634
1005.45000	exclusion1635
1006.52000	exclusion1636
1006.98000	exclusion1637
1007.95000	exclusion1638
1008.80000	exclusion1639
1009.06000	exclusion1640
1009.92000	exclusion1641
1010.38000	exclusion1642
1011.02000	exclusion1643
1011.77000	exclusion1644
1012.48000	exclusion1645
1012.94000	exclusion1646
1014.47000	exclusion1647
1015.42000	exclusion1648
1016.01000	exclusion1649
1016.49000	exclusion1650
1017.02000	exclusion1651
1017.53000	exclusion1652
1017.91000	exclusion1653
1018.95000	exclusion1654
1019.21000	exclusion1655
1020.05000	exclusion1656
1020.49000	exclusion1657
1021.00000	exclusion1658
1021.53000	exclusion1659
1022.46000	exclusion1660
1022.82000	exclusion1661
1023.45000	exclusion1662
1024.18000	exclusion1663
1024.51000	exclusion1664
1025.42000	exclusion1665
1025.86000	exclusion1666
1026.79000	exclusion1667
1027.46000	exclusion1668
1028.02000	exclusion1669
1028.98000	exclusion1670
1030.46000	exclusion1671
1031.47000	exclusion1672
1032.46000	exclusion1673

1032.86000	exclusion1674
1033.15000	exclusion1675
1033.52000	exclusion1676
1034.02000	exclusion1677
1035.55000	exclusion1678
1036.20000	exclusion1679
1036.53000	exclusion1680
1036.77000	exclusion1681
1037.45000	exclusion1682
1038.98000	exclusion1683
1039.29000	exclusion1684
1039.50000	exclusion1685
1040.04000	exclusion1686
1040.51000	exclusion1687
1041.47000	exclusion1688
1041.77000	exclusion1689
1042.02000	exclusion1690
1042.52000	exclusion1691
1043.14000	exclusion1692
1044.17000	exclusion1693
1044.52000	exclusion1694
1044.97000	exclusion1695
1045.46000	exclusion1696
1046.50000	exclusion1697
1047.82000	exclusion1698
1048.05000	exclusion1699
1048.84000	exclusion1700
1049.47000	exclusion1701
1049.87000	exclusion1702
1050.51000	exclusion1703
1050.77000	exclusion1704
1051.47000	exclusion1705
1052.53000	exclusion1706
1053.06000	exclusion1707
1053.53000	exclusion1708
1053.84000	exclusion1709
1054.50000	exclusion1710
1055.04000	exclusion1711
1055.48000	exclusion1712
1056.04000	exclusion1713
1056.52000	exclusion1714
1057.56000	exclusion1715
1057.84000	exclusion1716
1058.07000	exclusion1717
1058.48000	exclusion1718
1059.27000	exclusion1719
1059.48000	exclusion1720
1060.03000	exclusion1721
1060.53000	exclusion1722
1061.02000	exclusion1723
1061.83000	exclusion1724

1062.24000	exclusion1725
1062.54000	exclusion1726
1063.01000	exclusion1727
1063.48000	exclusion1728
1064.48000	exclusion1729
1065.47000	exclusion1730
1067.04000	exclusion1731
1067.49000	exclusion1732
1068.04000	exclusion1733
1068.47000	exclusion1734
1070.06000	exclusion1735
1070.52000	exclusion1736
1071.15000	exclusion1737
1071.54000	exclusion1738
1072.87000	exclusion1739
1073.53000	exclusion1740
1074.04000	exclusion1741
1075.53000	exclusion1742
1076.06000	exclusion1743
1077.04000	exclusion1744
1077.54000	exclusion1745
1078.99000	exclusion1746
1079.51000	exclusion1747
1080.50000	exclusion1748
1080.85000	exclusion1749
1081.53000	exclusion1750
1082.19000	exclusion1751
1082.53000	exclusion1752
1083.58000	exclusion1753
1084.06000	exclusion1754
1084.55000	exclusion1755
1085.04000	exclusion1756
1085.53000	exclusion1757
1086.05000	exclusion1758
1086.49000	exclusion1759
1087.55000	exclusion1760
1087.88000	exclusion1761
1088.24000	exclusion1762
1088.49000	exclusion1763
1090.06000	exclusion1764
1090.53000	exclusion1765
1091.06000	exclusion1766
1091.89000	exclusion1767
1093.53000	exclusion1768
1093.87000	exclusion1769
1094.55000	exclusion1770
1095.50000	exclusion1771
1095.86000	exclusion1772
1096.12000	exclusion1773
1097.29000	exclusion1774
1097.54000	exclusion1775

1098.20000	exclusion1776
1099.53000	exclusion1777
1101.09000	exclusion1778
1102.22000	exclusion1779
1102.51000	exclusion1780
1102.88000	exclusion1781
1103.99000	exclusion1782
1104.22000	exclusion1783
1104.58000	exclusion1784
1105.54000	exclusion1785
1105.77000	exclusion1786
1106.53000	exclusion1787
1107.54000	exclusion1788
1108.58000	exclusion1789
1109.52000	exclusion1790
1109.88000	exclusion1791
1110.23000	exclusion1792
1111.56000	exclusion1793
1111.87000	exclusion1794
1112.08000	exclusion1795
1112.80000	exclusion1796
1113.00000	exclusion1797
1113.52000	exclusion1798
1114.94000	exclusion1799
1115.55000	exclusion1800
1116.08000	exclusion1801
1116.88000	exclusion1802
1117.52000	exclusion1803
1118.09000	exclusion1804
1118.58000	exclusion1805
1118.86000	exclusion1806
1119.89000	exclusion1807
1120.52000	exclusion1808
1121.05000	exclusion1809
1122.19000	exclusion1810
1122.51000	exclusion1811
1123.21000	exclusion1812
1123.56000	exclusion1813
1124.57000	exclusion1814
1125.02000	exclusion1815
1125.54000	exclusion1816
1126.04000	exclusion1817
1127.55000	exclusion1818
1127.87000	exclusion1819
1128.09000	exclusion1820
1128.53000	exclusion1821
1130.21000	exclusion1822
1130.58000	exclusion1823
1131.03000	exclusion1824
1131.57000	exclusion1825
1132.20000	exclusion1826

1132.87000	exclusion1827
1133.08000	exclusion1828
1133.62000	exclusion1829
1133.89000	exclusion1830
1134.54000	exclusion1831
1135.11000	exclusion1832
1136.04000	exclusion1833
1136.53000	exclusion1834
1137.44000	exclusion1835
1138.55000	exclusion1836
1139.18000	exclusion1837
1139.54000	exclusion1838
1141.52000	exclusion1839
1141.90000	exclusion1840
1142.51000	exclusion1841
1143.05000	exclusion1842
1143.56000	exclusion1843
1145.04000	exclusion1844
1145.53000	exclusion1845
1146.56000	exclusion1846
1147.00000	exclusion1847
1147.53000	exclusion1848
1148.07000	exclusion1849
1148.56000	exclusion1850
1149.06000	exclusion1851
1149.56000	exclusion1852
1150.56000	exclusion1853
1151.05000	exclusion1854
1151.60000	exclusion1855
1152.12000	exclusion1856
1152.55000	exclusion1857
1153.54000	exclusion1858
1154.20000	exclusion1859
1154.55000	exclusion1860
1155.08000	exclusion1861
1156.07000	exclusion1862
1156.52000	exclusion1863
1157.56000	exclusion1864
1158.23000	exclusion1865
1158.51000	exclusion1866
1159.44000	exclusion1867
1159.64000	exclusion1868
1159.91000	exclusion1869
1161.57000	exclusion1870
1162.04000	exclusion1871
1162.58000	exclusion1872
1163.03000	exclusion1873
1164.56000	exclusion1874
1165.57000	exclusion1875
1167.20000	exclusion1876
1167.52000	exclusion1877

1168.20000	exclusion1878
1168.55000	exclusion1879
1169.58000	exclusion1880
1171.09000	exclusion1881
1171.61000	exclusion1882
1173.27000	exclusion1883
1173.56000	exclusion1884
1174.54000	exclusion1885
1175.48000	exclusion1886
1176.59000	exclusion1887
1177.58000	exclusion1888
1178.09000	exclusion1889
1178.50000	exclusion1890
1179.58000	exclusion1891
1180.14000	exclusion1892
1181.56000	exclusion1893
1182.54000	exclusion1894
1182.93000	exclusion1895
1184.10000	exclusion1896
1184.55000	exclusion1897
1185.54000	exclusion1898
1187.10000	exclusion1899
1187.58000	exclusion1900
1188.57000	exclusion1901
1189.54000	exclusion1902
1189.91000	exclusion1903
1192.62000	exclusion1904
1192.89000	exclusion1905
1193.59000	exclusion1906
1195.03000	exclusion1907
1195.25000	exclusion1908
1196.25000	exclusion1909
1196.54000	exclusion1910
1197.55000	exclusion1911
1198.55000	exclusion1912
1200.23000	exclusion1913
1200.56000	exclusion1914
1200.84000	exclusion1915
1201.11000	exclusion1916
1202.57000	exclusion1917
1203.50000	exclusion1918
1204.54000	exclusion1919
1204.91000	exclusion1920
1205.63000	exclusion1921
1206.03000	exclusion1922
1206.59000	exclusion1923
1207.88000	exclusion1924
1208.87000	exclusion1925
1209.91000	exclusion1926
1210.64000	exclusion1927
1211.01000	exclusion1928

1211.60000	exclusion1929
1212.25000	exclusion1930
1213.61000	exclusion1931
1214.58000	exclusion1932
1215.65000	exclusion1933
1216.12000	exclusion1934
1217.86000	exclusion1935
1218.54000	exclusion1936
1219.58000	exclusion1937
1221.12000	exclusion1938
1223.05000	exclusion1939
1223.57000	exclusion1940
1224.08000	exclusion1941
1224.59000	exclusion1942
1225.92000	exclusion1943
1226.62000	exclusion1944
1227.13000	exclusion1945
1228.28000	exclusion1946
1228.82000	exclusion1947
1230.06000	exclusion1948
1232.05000	exclusion1949
1233.30000	exclusion1950
1234.12000	exclusion1951
1235.07000	exclusion1952
1235.58000	exclusion1953
1237.12000	exclusion1954
1241.05000	exclusion1955
1241.64000	exclusion1956
1241.87000	exclusion1957
1245.11000	exclusion1958
1245.92000	exclusion1959
1246.97000	exclusion1960
1247.36000	exclusion1961
1247.59000	exclusion1962
1248.57000	exclusion1963
1249.04000	exclusion1964
1250.60000	exclusion1965
1252.19000	exclusion1966
1252.59000	exclusion1967
1253.07000	exclusion1968
1253.59000	exclusion1969
1254.63000	exclusion1970
1256.79000	exclusion1971
1257.09000	exclusion1972
1258.57000	exclusion1973
1259.21000	exclusion1974
1260.63000	exclusion1975
1264.16000	exclusion1976
1265.08000	exclusion1977
1265.59000	exclusion1978
1265.97000	exclusion1979

1270.27000 exclusion1980
1271.18000 exclusion1981
1272.68000 exclusion1982
1275.65000 exclusion1983
1276.64000 exclusion1984
1279.02000 exclusion1985
1279.61000 exclusion1986
1283.66000 exclusion1987
1284.64000 exclusion1988
1286.66000 exclusion1989
1287.08000 exclusion1990
1288.64000 exclusion1991
1289.45000 exclusion1992
1291.08000 exclusion1993
1292.39000 exclusion1994
1293.18000 exclusion1995
1294.61000 exclusion1996
1295.33000 exclusion1997
1298.08000 exclusion1998
1300.07000 exclusion1999
1301.06000 exclusion2000

Neutral Loss Mass List: (none)
Product Mass List: (none)
Neutral loss in top: 3
Product in top: 3
Most intense if no parent masses found not enabled
Add/subtract mass not enabled
FT master scan preview mode enabled
Charge state screening enabled
Charge state dependent ETD time not enabled
Monoisotopic precursor selection enabled
Charge state rejection enabled
Unassigned charge states : rejected
Charge state 1 : rejected
Charge state 2 : not rejected
Charge state 3 : not rejected
Charge states 4+ : not rejected
Chromatography mode is disabled

Global Data Dependent Settings:
Predict ion injection time enabled
Use global parent and reject mass lists not enabled
Exclude parent mass from data dependent selection not enabled
Exclusion mass width by mass
Exclusion mass width low: 1.50000
Exclusion mass width high: 1.50000
Parent mass width by mass
Parent mass width low: 0.50000
Parent mass width high: 0.50000
Reject mass width by mass
Reject mass width low: 0.50000
Reject mass width high: 0.50000

Zoom/UltraZoom scan mass width by mass
Zoom/UltraZoom scan mass width low: 5.00
Zoom/UltraZoom scan mass width high: 5.00
FT SIM scan mass width low: 5.00
FT SIM scan mass width high: 5.00
Neutral Loss candidates processed by decreasing intensity
Neutral Loss mass width by mass
Neutral Loss mass width low: 0.50000
Neutral Loss mass width high: 0.50000
Product candidates processed by decreasing intensity
Product mass width by mass
Product mass width low: 0.50000
Product mass width high: 0.50000
MS mass range: 300.00-2000.00
MSn mass range by mass
MSn mass range: 0.00-1000000.00
Use m/z values as masses not enabled
Analog UV data dep. not enabled
Dynamic exclusion enabled
Repeat Count: 1
Repeat Duration: 30.00
Exclusion List Size: 500
Exclusion Duration: 180.00
Exclusion mass width by mass
Exclusion mass width low: 1.50000
Exclusion mass width high: 1.50000
Expiration: disabled
Isotopic data dependence not enabled
Custom Data Dependent Settings:
Not enabled

Pass 3 (wheat-mixed-digests_MS2_pass03_1.raw):

Creator: Orbi_30393
Last modified: 10/12/2021 by Orbi_30393
MS Run Time (min): 43.00
Sequence override of method parameters not enabled.
Divert Valve: not used during run
Contact Closure: not used during run
Syringe Pump: not used during run
MS Detector Settings:
Real-time modifications to method not enabled
Stepped collision energy not enabled
Additional Microscans:

MS2	0	0
MS3	0	0
MS4	0	0
MS5	0	0
MS6	0	0
MS7	0	0
MS8	0	0

MS9 0 0
MS10 0 0
Experiment Type: Nth Order Double Play
Tune Method: Orbitrap-tune-file_2020-03-13_HESI
Scan Event Details:
1: FTMS + p norm o(300.0-2000.0)
CV = 0.0V
2: ITMS + c norm Dep MS/MS Most intense ion from (1)
Activation Type: CID
Min. Signal Required: 3000.0
Isolation Width: 2.00
Normalized Coll. Energy: 35.0
Default Charge State: 2
Activation Q: 0.250
Activation Time: 10.000
CV = 0.0V
Scan Event 2 repeated for top 10 peaks.
Lock Masses:
Pos List Name: N/A
Source: API Source
Mass List: (none)
Neg List Name: N/A
Source: API Source
Mass List: (none)
Data Dependent Settings:
Use separate polarity settings disabled
Parent Mass List: (none)
Reject Mass List:

MS Mass	Name
308.66000	exclusion0001
311.10000	exclusion0002
313.17000	exclusion0003
315.47000	exclusion0004
315.70000	exclusion0005
321.24000	exclusion0006
321.66000	exclusion0007
321.68000	exclusion0008
328.69000	exclusion0009
330.83000	exclusion0010
335.68000	exclusion0011
338.67000	exclusion0012
338.69000	exclusion0013
339.17000	exclusion0014
340.69000	exclusion0015
340.83000	exclusion0016
342.84000	exclusion0017
345.13000	exclusion0018
345.15000	exclusion0019
346.71000	exclusion0020
346.85000	exclusion0021
346.93000	exclusion0022

347.17000	exclusion0023
353.18000	exclusion0024
353.19000	exclusion0025
356.69000	exclusion0026
356.87000	exclusion0027
357.19000	exclusion0028
357.23000	exclusion0029
368.22000	exclusion0030
368.86000	exclusion0031
369.16000	exclusion0032
369.26000	exclusion0033
372.22000	exclusion0034
372.23000	exclusion0035
376.69000	exclusion0036
377.68000	exclusion0037
378.22000	exclusion0038
378.22000	exclusion0039
378.52000	exclusion0040
379.20000	exclusion0041
380.70000	exclusion0042
389.89000	exclusion0043
390.19000	exclusion0044
392.25000	exclusion0045
396.18000	exclusion0046
396.20000	exclusion0047
402.23000	exclusion0048
402.44000	exclusion0049
402.53000	exclusion0050
403.22000	exclusion0051
407.76000	exclusion0052
408.19000	exclusion0053
409.20000	exclusion0054
409.23000	exclusion0055
412.75000	exclusion0056
413.23000	exclusion0057
416.71000	exclusion0058
416.71000	exclusion0059
420.22000	exclusion0060
431.27000	exclusion0061
434.72000	exclusion0062
435.73000	exclusion0063
438.20000	exclusion0064
438.24000	exclusion0065
439.24000	exclusion0066
440.21000	exclusion0067
440.26000	exclusion0068
441.21000	exclusion0069
442.25000	exclusion0070
444.76000	exclusion0071
444.87000	exclusion0072
445.24000	exclusion0073

446.23000	exclusion0074
467.23000	exclusion0075
467.23000	exclusion0076
470.78000	exclusion0077
470.91000	exclusion0078
474.76000	exclusion0079
475.22000	exclusion0080
478.78000	exclusion0081
478.90000	exclusion0082
496.74000	exclusion0083
497.49000	exclusion0084
507.80000	exclusion0085
507.88000	exclusion0086
507.99000	exclusion0087
508.26000	exclusion0088
509.76000	exclusion0089
510.28000	exclusion0090
510.76000	exclusion0091
532.77000	exclusion0092
539.30000	exclusion0093
539.72000	exclusion0094
540.27000	exclusion0095
544.76000	exclusion0096
545.25000	exclusion0097
548.77000	exclusion0098
585.26000	exclusion0099
585.28000	exclusion0100
585.28000	exclusion0101
585.65000	exclusion0102
600.78000	exclusion0103
602.84000	exclusion0104
603.28000	exclusion0105
604.79000	exclusion0106
607.28000	exclusion0107
607.30000	exclusion0108
607.30000	exclusion0109
608.32000	exclusion0110
609.85000	exclusion0111
612.32000	exclusion0112
612.33000	exclusion0113
612.35000	exclusion0114
613.32000	exclusion0115
625.93000	exclusion0606
626.28000	exclusion0607
626.81000	exclusion0608
627.01000	exclusion0609
627.30000	exclusion0610
628.29000	exclusion0611
628.58000	exclusion0612
628.80000	exclusion0613
628.96000	exclusion0614

629.03000	exclusion0615
629.82000	exclusion0616
630.30000	exclusion0617
630.77000	exclusion0618
631.31000	exclusion0619
631.83000	exclusion0620
632.29000	exclusion0621
632.83000	exclusion0622
633.28000	exclusion0623
633.31000	exclusion0624
633.83000	exclusion0625
634.33000	exclusion0626
634.79000	exclusion0627
635.29000	exclusion0116
635.32000	exclusion0117
635.86000	exclusion0118
635.86000	exclusion0628
636.32000	exclusion0629
636.83000	exclusion0630
637.64000	exclusion0631
637.78000	exclusion0632
637.85000	exclusion0633
638.23000	exclusion0634
638.26000	exclusion0635
638.85000	exclusion0636
639.35000	exclusion0637
639.59000	exclusion0638
639.61000	exclusion0639
639.78000	exclusion0640
639.92000	exclusion0641
640.30000	exclusion0642
640.61000	exclusion0643
641.35000	exclusion0644
641.44000	exclusion0645
641.84000	exclusion0646
642.35000	exclusion0119
642.86000	exclusion0647
643.29000	exclusion0648
643.36000	exclusion0649
643.82000	exclusion0650
644.02000	exclusion0651
644.87000	exclusion0652
645.02000	exclusion0653
645.35000	exclusion0654
645.51000	exclusion0655
645.70000	exclusion0656
645.98000	exclusion0657
646.80000	exclusion0120
647.03000	exclusion0121
647.31000	exclusion0658
647.35000	exclusion0659

647.87000	exclusion0660
648.00000	exclusion0661
648.29000	exclusion0662
648.30000	exclusion0663
648.31000	exclusion0664
648.82000	exclusion0665
649.28000	exclusion0666
649.82000	exclusion0667
650.29000	exclusion0668
650.82000	exclusion0669
651.30000	exclusion0670
651.32000	exclusion0671
651.78000	exclusion0672
652.30000	exclusion0122
652.32000	exclusion0123
652.84000	exclusion0673
653.24000	exclusion0674
653.32000	exclusion0124
653.35000	exclusion0125
653.36000	exclusion0675
653.63000	exclusion0676
654.30000	exclusion0677
654.35000	exclusion0678
654.85000	exclusion0679
655.09000	exclusion0680
655.82000	exclusion0681
656.32000	exclusion0682
656.54000	exclusion0683
657.03000	exclusion0684
657.35000	exclusion0685
657.38000	exclusion0686
657.83000	exclusion0687
658.03000	exclusion0688
658.32000	exclusion0689
658.35000	exclusion0690
659.28000	exclusion0691
659.34000	exclusion0692
659.78000	exclusion0693
660.30000	exclusion0694
660.35000	exclusion0695
660.66000	exclusion0696
661.30000	exclusion0697
661.81000	exclusion0698
662.32000	exclusion0699
662.83000	exclusion0700
663.32000	exclusion0701
663.75000	exclusion0702
664.11000	exclusion0703
664.37000	exclusion0704
664.66000	exclusion0705
665.32000	exclusion0706

665.34000	exclusion0707
665.64000	exclusion0708
665.87000	exclusion0709
666.34000	exclusion0710
666.86000	exclusion0711
667.03000	exclusion0712
667.36000	exclusion0713
667.52000	exclusion0714
668.66000	exclusion0715
668.83000	exclusion0716
669.34000	exclusion0717
669.51000	exclusion0718
669.85000	exclusion0719
669.90000	exclusion0720
670.29000	exclusion0721
670.80000	exclusion0722
671.30000	exclusion0126
671.53000	exclusion0127
671.83000	exclusion0128
671.83000	exclusion0723
672.32000	exclusion0724
672.82000	exclusion0725
673.11000	exclusion0726
673.33000	exclusion0727
673.34000	exclusion0728
674.31000	exclusion0729
674.33000	exclusion0730
674.58000	exclusion0731
674.83000	exclusion0732
675.19000	exclusion0733
676.05000	exclusion0734
676.85000	exclusion0735
677.00000	exclusion0736
677.28000	exclusion0737
677.34000	exclusion0738
677.85000	exclusion0739
677.88000	exclusion0740
678.36000	exclusion0741
679.04000	exclusion0742
679.31000	exclusion0743
679.85000	exclusion0744
680.33000	exclusion0745
680.78000	exclusion0746
681.32000	exclusion0747
681.83000	exclusion0129
681.84000	exclusion0130
682.37000	exclusion0748
682.67000	exclusion0749
682.82000	exclusion0750
682.85000	exclusion0131
683.32000	exclusion0751

683.37000	exclusion0752
683.97000	exclusion0753
684.29000	exclusion0754
684.78000	exclusion0755
685.67000	exclusion0756
685.83000	exclusion0757
686.31000	exclusion0758
686.34000	exclusion0759
686.85000	exclusion0760
687.30000	exclusion0761
687.33000	exclusion0762
687.68000	exclusion0763
688.27000	exclusion0132
688.78000	exclusion0764
689.00000	exclusion0765
689.34000	exclusion0766
689.37000	exclusion0767
689.67000	exclusion0768
690.35000	exclusion0769
690.72000	exclusion0770
691.32000	exclusion0771
691.36000	exclusion0772
692.02000	exclusion0773
692.26000	exclusion0774
692.70000	exclusion0775
693.10000	exclusion0776
693.56000	exclusion0777
693.88000	exclusion0778
694.31000	exclusion0779
694.81000	exclusion0133
695.33000	exclusion0780
695.39000	exclusion0781
695.85000	exclusion0782
696.33000	exclusion0783
696.35000	exclusion0784
697.01000	exclusion0785
697.36000	exclusion0786
697.83000	exclusion0787
698.37000	exclusion0788
698.89000	exclusion0789
699.12000	exclusion0790
699.37000	exclusion0791
699.38000	exclusion0792
699.68000	exclusion0793
700.37000	exclusion0794
700.90000	exclusion0795
701.33000	exclusion0796
702.31000	exclusion0797
702.33000	exclusion0798
702.77000	exclusion0799
702.87000	exclusion0800

703.38000	exclusion0801
703.71000	exclusion0802
704.31000	exclusion0803
705.27000	exclusion0804
705.37000	exclusion0805
705.82000	exclusion0806
706.81000	exclusion0807
707.02000	exclusion0808
707.32000	exclusion0809
708.04000	exclusion0810
708.33000	exclusion0811
708.38000	exclusion0812
708.85000	exclusion0813
709.35000	exclusion0814
709.35000	exclusion0815
709.90000	exclusion0816
710.33000	exclusion0817
710.36000	exclusion0818
710.81000	exclusion0819
711.35000	exclusion0134
711.69000	exclusion0135
711.89000	exclusion0136
712.05000	exclusion0820
712.34000	exclusion0821
712.35000	exclusion0822
712.38000	exclusion0137
712.39000	exclusion0138
712.59000	exclusion0823
712.70000	exclusion0824
712.80000	exclusion0825
713.09000	exclusion0826
713.34000	exclusion0827
713.68000	exclusion0828
713.89000	exclusion0829
714.35000	exclusion0830
714.40000	exclusion0831
715.01000	exclusion0832
715.36000	exclusion0833
715.84000	exclusion0834
716.31000	exclusion0835
716.87000	exclusion0836
717.83000	exclusion0837
718.31000	exclusion0838
718.34000	exclusion0839
718.85000	exclusion0139
718.85000	exclusion0140
719.35000	exclusion0840
719.71000	exclusion0841
719.81000	exclusion0842
720.32000	exclusion0843
720.90000	exclusion0844

721.37000	exclusion0845
721.41000	exclusion0846
721.73000	exclusion0847
721.79000	exclusion0848
722.59000	exclusion0849
722.87000	exclusion0850
723.07000	exclusion0851
723.33000	exclusion0852
723.40000	exclusion0853
723.93000	exclusion0141
724.34000	exclusion0142
724.37000	exclusion0143
724.68000	exclusion0144
724.88000	exclusion0854
725.04000	exclusion0855
725.37000	exclusion0145
725.38000	exclusion0856
725.82000	exclusion0857
726.05000	exclusion0858
726.72000	exclusion0859
726.81000	exclusion0860
727.37000	exclusion0861
727.50000	exclusion0862
728.02000	exclusion0863
728.32000	exclusion0864
728.88000	exclusion0865
729.32000	exclusion0866
729.86000	exclusion0867
730.36000	exclusion0868
730.36000	exclusion0869
730.64000	exclusion0870
730.87000	exclusion0871
731.01000	exclusion0872
731.88000	exclusion0873
732.35000	exclusion0874
732.84000	exclusion0875
733.37000	exclusion0876
733.86000	exclusion0877
734.32000	exclusion0878
734.36000	exclusion0879
735.33000	exclusion0880
735.90000	exclusion0881
736.37000	exclusion0882
736.38000	exclusion0883
736.86000	exclusion0884
737.36000	exclusion0885
737.88000	exclusion0886
738.04000	exclusion0887
738.38000	exclusion0888
739.35000	exclusion0889
739.86000	exclusion0890

740.28000	exclusion0891
740.87000	exclusion0892
741.37000	exclusion0893
742.37000	exclusion0894
742.83000	exclusion0895
743.06000	exclusion0896
743.38000	exclusion0897
743.85000	exclusion0898
744.25000	exclusion0899
744.38000	exclusion0900
744.86000	exclusion0901
745.36000	exclusion0146
745.90000	exclusion0147
746.28000	exclusion0902
746.72000	exclusion0903
747.03000	exclusion0904
747.73000	exclusion0905
747.83000	exclusion0906
748.07000	exclusion0907
748.32000	exclusion0908
748.85000	exclusion0909
749.04000	exclusion0910
749.38000	exclusion0911
749.61000	exclusion0912
750.36000	exclusion0913
750.37000	exclusion0914
750.87000	exclusion0915
751.37000	exclusion0916
751.38000	exclusion0917
751.86000	exclusion0918
752.11000	exclusion0919
752.36000	exclusion0920
753.10000	exclusion0921
753.37000	exclusion0922
753.38000	exclusion0923
754.32000	exclusion0924
754.83000	exclusion0925
755.34000	exclusion0926
755.73000	exclusion0927
755.86000	exclusion0928
756.08000	exclusion0929
756.32000	exclusion0930
756.71000	exclusion0931
757.36000	exclusion0932
757.40000	exclusion0933
757.70000	exclusion0934
757.88000	exclusion0935
758.84000	exclusion0936
758.89000	exclusion0937
759.37000	exclusion0938
759.39000	exclusion0939

759.68000	exclusion0940
759.86000	exclusion0941
760.36000	exclusion0942
760.41000	exclusion0943
760.73000	exclusion0944
760.91000	exclusion0945
761.34000	exclusion0946
761.90000	exclusion0947
762.40000	exclusion0948
762.40000	exclusion0949
762.88000	exclusion0950
763.34000	exclusion0951
763.90000	exclusion0952
764.21000	exclusion0953
764.36000	exclusion0954
764.73000	exclusion0955
765.34000	exclusion0148
765.36000	exclusion0149
765.86000	exclusion0956
766.05000	exclusion0957
766.35000	exclusion0958
766.36000	exclusion0150
766.39000	exclusion0959
767.16000	exclusion0151
767.37000	exclusion0152
767.37000	exclusion0153
767.86000	exclusion0154
767.87000	exclusion0960
768.35000	exclusion0961
768.66000	exclusion0962
768.90000	exclusion0963
769.31000	exclusion0964
769.90000	exclusion0965
770.37000	exclusion0966
770.86000	exclusion0967
771.02000	exclusion0968
771.38000	exclusion0969
771.88000	exclusion0970
772.39000	exclusion0155
772.39000	exclusion0156
772.85000	exclusion0157
773.36000	exclusion0971
773.37000	exclusion0972
773.38000	exclusion0158
773.87000	exclusion0973
774.30000	exclusion0974
774.61000	exclusion0975
775.34000	exclusion0976
775.39000	exclusion0977
775.83000	exclusion0978
776.37000	exclusion0979

776.58000	exclusion0980
776.86000	exclusion0981
777.39000	exclusion0982
777.40000	exclusion0983
777.83000	exclusion0984
778.40000	exclusion0985
778.91000	exclusion0986
779.11000	exclusion0987
779.34000	exclusion0988
779.37000	exclusion0989
779.86000	exclusion0990
780.75000	exclusion0991
780.81000	exclusion0992
781.08000	exclusion0993
781.16000	exclusion0994
781.39000	exclusion0995
782.28000	exclusion0996
782.79000	exclusion0159
783.30000	exclusion0997
783.41000	exclusion0998
784.35000	exclusion0999
784.87000	exclusion1000
785.30000	exclusion1001
785.65000	exclusion1002
786.39000	exclusion1003
786.88000	exclusion1004
787.10000	exclusion1005
787.36000	exclusion1006
787.90000	exclusion1007
788.71000	exclusion1008
789.04000	exclusion1009
789.37000	exclusion1010
790.36000	exclusion1011
790.38000	exclusion1012
791.06000	exclusion1013
791.37000	exclusion1014
792.29000	exclusion1015
792.38000	exclusion1016
792.91000	exclusion1017
792.91000	exclusion1018
793.38000	exclusion1019
793.87000	exclusion1020
793.94000	exclusion1021
794.38000	exclusion1022
794.40000	exclusion1023
795.36000	exclusion1024
795.42000	exclusion1025
795.90000	exclusion1026
796.12000	exclusion1027
796.37000	exclusion1028
796.40000	exclusion1029

796.88000	exclusion1030
797.43000	exclusion1031
797.87000	exclusion1032
798.35000	exclusion1033
798.41000	exclusion1034
799.13000	exclusion1035
799.87000	exclusion1036
800.35000	exclusion1037
800.38000	exclusion1038
800.68000	exclusion1039
801.38000	exclusion1040
801.38000	exclusion1041
801.95000	exclusion1042
802.27000	exclusion1043
802.39000	exclusion1044
802.69000	exclusion1045
802.82000	exclusion1046
803.37000	exclusion0160
803.92000	exclusion1047
804.40000	exclusion1048
804.41000	exclusion1049
804.74000	exclusion1050
805.00000	exclusion1051
805.40000	exclusion1052
806.39000	exclusion1053
806.43000	exclusion1054
806.88000	exclusion1055
807.02000	exclusion1056
808.10000	exclusion1057
808.36000	exclusion1058
808.36000	exclusion1059
808.94000	exclusion1060
809.40000	exclusion1061
809.91000	exclusion1062
810.39000	exclusion1063
810.39000	exclusion1064
810.75000	exclusion1065
811.36000	exclusion1066
812.36000	exclusion0161
812.38000	exclusion1067
812.73000	exclusion1068
812.86000	exclusion0162
813.37000	exclusion1069
813.43000	exclusion1070
814.36000	exclusion0163
814.41000	exclusion0164
814.74000	exclusion0165
815.36000	exclusion1071
815.41000	exclusion1072
815.93000	exclusion1073
816.44000	exclusion0166

817.05000	exclusion1074
817.33000	exclusion1075
817.75000	exclusion1076
817.86000	exclusion1077
818.43000	exclusion1078
818.55000	exclusion1079
818.87000	exclusion1080
819.04000	exclusion1081
819.42000	exclusion1082
820.37000	exclusion1083
821.15000	exclusion1084
821.39000	exclusion1085
822.38000	exclusion1086
822.39000	exclusion1087
822.91000	exclusion1088
822.92000	exclusion1089
823.57000	exclusion1090
824.40000	exclusion1091
824.81000	exclusion1092
825.40000	exclusion1093
825.85000	exclusion1094
826.22000	exclusion1095
826.38000	exclusion1096
826.44000	exclusion1097
827.42000	exclusion1098
827.42000	exclusion1099
827.89000	exclusion1100
828.41000	exclusion1101
828.44000	exclusion1102
829.41000	exclusion1103
829.43000	exclusion1104
830.43000	exclusion1105
830.92000	exclusion1106
830.96000	exclusion1107
831.13000	exclusion1108
831.32000	exclusion1109
831.94000	exclusion1110
832.41000	exclusion1111
832.44000	exclusion1112
832.88000	exclusion1113
833.15000	exclusion1114
833.40000	exclusion1115
833.78000	exclusion1116
834.16000	exclusion1117
834.33000	exclusion1118
834.43000	exclusion1119
834.92000	exclusion1120
835.04000	exclusion1121
835.13000	exclusion1122
835.72000	exclusion0167
836.06000	exclusion0168

836.40000	exclusion0169
836.41000	exclusion1123
836.42000	exclusion1124
837.42000	exclusion1125
837.44000	exclusion1126
837.76000	exclusion1127
837.93000	exclusion1128
838.42000	exclusion1129
839.16000	exclusion1130
839.25000	exclusion1131
839.43000	exclusion1132
839.91000	exclusion1133
840.42000	exclusion1134
840.67000	exclusion1135
840.73000	exclusion1136
841.44000	exclusion1137
841.45000	exclusion1138
842.11000	exclusion0170
842.14000	exclusion0171
842.38000	exclusion0172
842.39000	exclusion0173
842.91000	exclusion1139
842.92000	exclusion1140
843.40000	exclusion1141
843.64000	exclusion1142
843.91000	exclusion1143
843.94000	exclusion1144
844.44000	exclusion0174
844.58000	exclusion0175
844.89000	exclusion0176
845.37000	exclusion0177
845.41000	exclusion0178
845.41000	exclusion1145
845.87000	exclusion1146
846.16000	exclusion1147
846.36000	exclusion1148
847.37000	exclusion1149
847.39000	exclusion1150
847.88000	exclusion1151
848.36000	exclusion1152
848.91000	exclusion0179
849.37000	exclusion0180
849.41000	exclusion1153
849.44000	exclusion1154
849.89000	exclusion1155
849.90000	exclusion1156
850.42000	exclusion1157
850.91000	exclusion1158
851.17000	exclusion1159
851.42000	exclusion1160
851.45000	exclusion1161

851.78000	exclusion1162
852.38000	exclusion1163
852.39000	exclusion1164
852.88000	exclusion1165
853.23000	exclusion1166
853.35000	exclusion1167
853.66000	exclusion1168
854.38000	exclusion1169
855.37000	exclusion1170
855.40000	exclusion1171
855.88000	exclusion1172
856.15000	exclusion1173
856.40000	exclusion1174
857.06000	exclusion1175
857.45000	exclusion1176
857.89000	exclusion1177
858.50000	exclusion1178
858.74000	exclusion1179
859.47000	exclusion1180
859.64000	exclusion1181
860.08000	exclusion1182
860.39000	exclusion1183
860.88000	exclusion1184
861.44000	exclusion1185
861.90000	exclusion1186
862.46000	exclusion1187
862.49000	exclusion1188
862.90000	exclusion1189
863.39000	exclusion1190
863.41000	exclusion1191
863.92000	exclusion0181
864.42000	exclusion0182
864.44000	exclusion1192
864.45000	exclusion1193
864.88000	exclusion1194
865.40000	exclusion0183
865.91000	exclusion1195
866.41000	exclusion0184
866.41000	exclusion1196
866.42000	exclusion1197
867.43000	exclusion0185
867.45000	exclusion1198
867.75000	exclusion1199
868.13000	exclusion1200
868.40000	exclusion1201
868.40000	exclusion1202
868.90000	exclusion1203
868.94000	exclusion1204
869.22000	exclusion1205
869.73000	exclusion1206
870.18000	exclusion1207

870.42000	exclusion1208
870.43000	exclusion1209
871.35000	exclusion1210
872.38000	exclusion1211
872.43000	exclusion1212
872.70000	exclusion1213
872.92000	exclusion1214
873.45000	exclusion1215
874.05000	exclusion1216
874.42000	exclusion1217
874.95000	exclusion1218
875.39000	exclusion1219
875.90000	exclusion1220
876.01000	exclusion1221
876.40000	exclusion1222
876.40000	exclusion1223
877.36000	exclusion1224
877.40000	exclusion1225
878.05000	exclusion1226
878.48000	exclusion1227
879.05000	exclusion1228
879.41000	exclusion1229
879.93000	exclusion1230
880.40000	exclusion1231
880.41000	exclusion1232
880.73000	exclusion1233
881.43000	exclusion1234
881.94000	exclusion0186
882.44000	exclusion1235
882.46000	exclusion1236
882.91000	exclusion1237
883.44000	exclusion1238
884.41000	exclusion1239
884.47000	exclusion1240
884.89000	exclusion1241
885.42000	exclusion1242
885.45000	exclusion1243
885.93000	exclusion1244
886.40000	exclusion1245
886.95000	exclusion1246
887.48000	exclusion1247
887.96000	exclusion1248
888.40000	exclusion1249
888.42000	exclusion1250
888.74000	exclusion1251
889.47000	exclusion1252
890.41000	exclusion1253
890.66000	exclusion1254
890.88000	exclusion1255
891.41000	exclusion1256
891.90000	exclusion1257

892.24000	exclusion1258
892.45000	exclusion1259
893.44000	exclusion1260
893.89000	exclusion1261
894.18000	exclusion1262
894.44000	exclusion1263
895.42000	exclusion1264
895.48000	exclusion1265
895.89000	exclusion1266
896.16000	exclusion1267
896.43000	exclusion1268
896.45000	exclusion1269
897.41000	exclusion1270
898.10000	exclusion1271
898.42000	exclusion1272
898.82000	exclusion1273
899.44000	exclusion1274
899.45000	exclusion1275
900.42000	exclusion1276
900.43000	exclusion1277
901.14000	exclusion1278
901.45000	exclusion1279
902.42000	exclusion1280
902.70000	exclusion1281
903.13000	exclusion1282
903.41000	exclusion1283
904.43000	exclusion0187
904.44000	exclusion0188
904.79000	exclusion1284
905.13000	exclusion1285
905.40000	exclusion1286
905.96000	exclusion1287
906.41000	exclusion1288
907.42000	exclusion1289
907.45000	exclusion1290
907.96000	exclusion1291
908.40000	exclusion1292
908.93000	exclusion0189
909.43000	exclusion0190
909.44000	exclusion1293
909.44000	exclusion1294
909.94000	exclusion1295
910.44000	exclusion0191
910.44000	exclusion0192
911.13000	exclusion1296
911.46000	exclusion1297
911.69000	exclusion1298
911.94000	exclusion1299
911.97000	exclusion1300
912.41000	exclusion1301
912.91000	exclusion0193

912.99000	exclusion0194
913.45000	exclusion0195
913.66000	exclusion1302
913.70000	exclusion1303
913.92000	exclusion1304
913.93000	exclusion0196
914.43000	exclusion1305
914.47000	exclusion1306
915.12000	exclusion1307
915.45000	exclusion1308
915.95000	exclusion1309
916.46000	exclusion1310
916.49000	exclusion1311
916.75000	exclusion1312
917.08000	exclusion1313
917.47000	exclusion1314
917.93000	exclusion1315
918.11000	exclusion1316
918.46000	exclusion1317
918.48000	exclusion1318
918.92000	exclusion1319
919.43000	exclusion1320
919.54000	exclusion1321
920.42000	exclusion1322
920.49000	exclusion1323
920.87000	exclusion1324
921.40000	exclusion1325
921.47000	exclusion1326
922.30000	exclusion1327
922.47000	exclusion1328
922.90000	exclusion1329
923.46000	exclusion1330
923.91000	exclusion1331
924.13000	exclusion1332
924.43000	exclusion1333
924.94000	exclusion1334
925.49000	exclusion1335
926.46000	exclusion1336
926.79000	exclusion1337
927.48000	exclusion0197
927.82000	exclusion1338
927.88000	exclusion1339
928.47000	exclusion1340
928.93000	exclusion1341
929.45000	exclusion1342
929.77000	exclusion1343
930.46000	exclusion1344
930.47000	exclusion1345
930.68000	exclusion1346
931.11000	exclusion1347
931.40000	exclusion1348

931.41000	exclusion1349
931.81000	exclusion1350
932.43000	exclusion1351
932.94000	exclusion0198
933.14000	exclusion0199
933.47000	exclusion0200
933.47000	exclusion1352
933.79000	exclusion1353
934.39000	exclusion1354
934.97000	exclusion1355
935.16000	exclusion1356
935.47000	exclusion1357
935.91000	exclusion1358
936.11000	exclusion1359
937.12000	exclusion0201
937.46000	exclusion0202
937.49000	exclusion1360
937.52000	exclusion1361
937.79000	exclusion1362
937.99000	exclusion1363
938.49000	exclusion1364
939.21000	exclusion1365
939.49000	exclusion1366
939.93000	exclusion1367
940.44000	exclusion0203
940.48000	exclusion0204
941.09000	exclusion0205
941.46000	exclusion1368
942.47000	exclusion1369
942.48000	exclusion0206
942.98000	exclusion1370
943.10000	exclusion1371
943.47000	exclusion1372
943.73000	exclusion1373
944.47000	exclusion1374
944.74000	exclusion1375
944.92000	exclusion1376
945.48000	exclusion1377
945.78000	exclusion1378
945.98000	exclusion1379
946.95000	exclusion1380
947.49000	exclusion1381
947.96000	exclusion1382
948.46000	exclusion1383
949.02000	exclusion1384
949.46000	exclusion1385
949.80000	exclusion1386
950.01000	exclusion1387
950.70000	exclusion1388
951.13000	exclusion1389
951.41000	exclusion1390

951.47000	exclusion1391
951.82000	exclusion1392
952.14000	exclusion1393
952.77000	exclusion1394
952.96000	exclusion1395
953.01000	exclusion1396
953.48000	exclusion1397
954.44000	exclusion1398
954.94000	exclusion1399
955.35000	exclusion1400
955.57000	exclusion1401
956.47000	exclusion1402
957.43000	exclusion1403
957.74000	exclusion1404
958.44000	exclusion1405
958.81000	exclusion1406
959.41000	exclusion1407
960.41000	exclusion0207
960.45000	exclusion1408
960.75000	exclusion1409
961.00000	exclusion1410
961.35000	exclusion1411
961.98000	exclusion0208
962.49000	exclusion1412
962.87000	exclusion1413
963.48000	exclusion1414
963.94000	exclusion1415
964.44000	exclusion1416
964.74000	exclusion1417
964.98000	exclusion1418
965.02000	exclusion1419
965.48000	exclusion1420
966.26000	exclusion1421
966.48000	exclusion1422
967.21000	exclusion1423
967.48000	exclusion1424
967.48000	exclusion1425
967.90000	exclusion1426
968.47000	exclusion1427
968.97000	exclusion0209
969.50000	exclusion1428
969.98000	exclusion1429
970.11000	exclusion1430
970.45000	exclusion1431
970.52000	exclusion1432
971.48000	exclusion1433
971.96000	exclusion1434
972.15000	exclusion1435
972.44000	exclusion1436
972.48000	exclusion1437
972.54000	exclusion1438

973.00000	exclusion1439
973.80000	exclusion1440
974.47000	exclusion1441
975.47000	exclusion1442
975.49000	exclusion1443
975.82000	exclusion1444
976.68000	exclusion1445
977.14000	exclusion1446
977.49000	exclusion1447
977.78000	exclusion1448
978.46000	exclusion1449
978.51000	exclusion1450
979.42000	exclusion1451
980.13000	exclusion1452
980.51000	exclusion1453
980.97000	exclusion1454
981.21000	exclusion1455
981.30000	exclusion1456
981.54000	exclusion1457
982.46000	exclusion1458
983.45000	exclusion1459
983.49000	exclusion1460
984.48000	exclusion1461
984.50000	exclusion1462
984.94000	exclusion1463
985.87000	exclusion1464
986.00000	exclusion1465
986.47000	exclusion1466
986.83000	exclusion1467
987.01000	exclusion1468
987.51000	exclusion1469
987.97000	exclusion1470
988.18000	exclusion1471
988.49000	exclusion1472
989.01000	exclusion1473
989.52000	exclusion0210
990.02000	exclusion1474
990.14000	exclusion1475
990.47000	exclusion1476
990.50000	exclusion1477
991.16000	exclusion1478
991.20000	exclusion1479
991.48000	exclusion1480
991.83000	exclusion1481
991.99000	exclusion1482
992.50000	exclusion0211
993.02000	exclusion0212
993.50000	exclusion0213
993.52000	exclusion1483
993.96000	exclusion1484
994.47000	exclusion0214

994.98000	exclusion0215
995.46000	exclusion0216
995.49000	exclusion1485
996.48000	exclusion1486
996.98000	exclusion0217
997.50000	exclusion1487
998.48000	exclusion1488
998.81000	exclusion1489
999.39000	exclusion1490
999.82000	exclusion1491
1000.46000	exclusion1492
1000.84000	exclusion1493
1001.50000	exclusion1494
1002.48000	exclusion1495
1003.01000	exclusion1496
1003.53000	exclusion1497
1004.48000	exclusion1498
1004.94000	exclusion1499
1005.45000	exclusion1500
1005.98000	exclusion0218
1006.52000	exclusion1501
1006.98000	exclusion1502
1007.95000	exclusion1503
1008.80000	exclusion1504
1009.06000	exclusion1505
1009.92000	exclusion1506
1010.38000	exclusion1507
1011.02000	exclusion1508
1011.77000	exclusion1509
1012.48000	exclusion1510
1012.94000	exclusion1511
1013.49000	exclusion0219
1013.79000	exclusion0220
1014.47000	exclusion0221
1014.47000	exclusion1512
1015.42000	exclusion1513
1016.01000	exclusion1514
1016.49000	exclusion1515
1017.02000	exclusion1516
1017.53000	exclusion1517
1017.91000	exclusion1518
1018.41000	exclusion0222
1018.95000	exclusion1519
1019.21000	exclusion1520
1020.05000	exclusion1521
1020.49000	exclusion1522
1021.00000	exclusion1523
1021.53000	exclusion1524
1022.46000	exclusion1525
1022.82000	exclusion1526
1023.45000	exclusion1527

1024.18000	exclusion1528
1024.51000	exclusion1529
1025.42000	exclusion1530
1025.86000	exclusion1531
1026.79000	exclusion1532
1027.46000	exclusion1533
1028.02000	exclusion1534
1028.98000	exclusion1535
1029.51000	exclusion0223
1030.46000	exclusion1536
1030.53000	exclusion0224
1031.47000	exclusion1537
1032.46000	exclusion1538
1032.86000	exclusion1539
1033.15000	exclusion1540
1033.52000	exclusion1541
1034.02000	exclusion1542
1034.80000	exclusion0225
1035.54000	exclusion0226
1035.55000	exclusion1543
1036.20000	exclusion1544
1036.53000	exclusion1545
1036.77000	exclusion1546
1037.45000	exclusion1547
1038.03000	exclusion0227
1038.26000	exclusion0228
1038.46000	exclusion0229
1038.98000	exclusion1548
1039.29000	exclusion1549
1039.50000	exclusion1550
1040.04000	exclusion1551
1040.51000	exclusion1552
1041.47000	exclusion1553
1041.77000	exclusion1554
1042.02000	exclusion1555
1042.52000	exclusion1556
1043.14000	exclusion1557
1044.17000	exclusion1558
1044.52000	exclusion1559
1044.97000	exclusion1560
1045.46000	exclusion1561
1046.50000	exclusion0230
1046.50000	exclusion1562
1047.50000	exclusion0231
1047.82000	exclusion1563
1048.05000	exclusion1564
1048.84000	exclusion1565
1049.47000	exclusion1566
1049.87000	exclusion1567
1050.51000	exclusion1568
1050.77000	exclusion1569

1051.47000	exclusion1570
1052.00000	exclusion0232
1052.53000	exclusion1571
1053.06000	exclusion1572
1053.53000	exclusion1573
1053.84000	exclusion1574
1054.50000	exclusion1575
1055.04000	exclusion1576
1055.48000	exclusion1577
1056.04000	exclusion1578
1056.52000	exclusion1579
1057.55000	exclusion0233
1057.56000	exclusion1580
1057.84000	exclusion1581
1058.07000	exclusion1582
1058.48000	exclusion1583
1059.27000	exclusion1584
1059.48000	exclusion1585
1060.03000	exclusion1586
1060.53000	exclusion1587
1061.02000	exclusion1588
1061.83000	exclusion1589
1062.24000	exclusion1590
1062.54000	exclusion1591
1063.01000	exclusion1592
1063.48000	exclusion1593
1064.48000	exclusion1594
1065.47000	exclusion1595
1066.11000	exclusion0234
1066.54000	exclusion0235
1067.04000	exclusion1596
1067.49000	exclusion1597
1067.53000	exclusion0236
1068.04000	exclusion1598
1068.47000	exclusion1599
1069.03000	exclusion0237
1069.45000	exclusion0238
1070.06000	exclusion1600
1070.52000	exclusion1601
1071.15000	exclusion1602
1071.54000	exclusion1603
1072.07000	exclusion0239
1072.54000	exclusion0240
1072.84000	exclusion0241
1072.87000	exclusion1604
1073.53000	exclusion1605
1074.04000	exclusion1606
1074.55000	exclusion0242
1074.85000	exclusion0243
1075.13000	exclusion0244
1075.52000	exclusion0245

1075.53000	exclusion1607
1076.06000	exclusion1608
1077.04000	exclusion1609
1077.54000	exclusion1610
1078.99000	exclusion1611
1079.51000	exclusion1612
1080.50000	exclusion1613
1080.85000	exclusion1614
1081.53000	exclusion1615
1082.19000	exclusion1616
1082.53000	exclusion1617
1083.55000	exclusion0246
1083.58000	exclusion1618
1084.06000	exclusion1619
1084.55000	exclusion1620
1085.04000	exclusion1621
1085.53000	exclusion1622
1086.05000	exclusion1623
1086.49000	exclusion1624
1086.99000	exclusion0247
1087.55000	exclusion0248
1087.55000	exclusion1625
1087.88000	exclusion1626
1088.24000	exclusion1627
1088.49000	exclusion1628
1089.00000	exclusion0249
1089.53000	exclusion0250
1090.05000	exclusion0251
1090.06000	exclusion1629
1090.53000	exclusion1630
1091.06000	exclusion1631
1091.89000	exclusion1632
1092.50000	exclusion0252
1093.03000	exclusion0253
1093.53000	exclusion1633
1093.87000	exclusion1634
1094.55000	exclusion1635
1095.50000	exclusion1636
1095.86000	exclusion1637
1096.12000	exclusion1638
1097.16000	exclusion0254
1097.29000	exclusion1639
1097.54000	exclusion1640
1098.20000	exclusion1641
1099.29000	exclusion0255
1099.53000	exclusion1642
1100.03000	exclusion0256
1100.56000	exclusion0257
1100.81000	exclusion0258
1101.09000	exclusion0259
1101.09000	exclusion1643

1102.22000	exclusion1644
1102.51000	exclusion1645
1102.88000	exclusion1646
1103.99000	exclusion1647
1104.22000	exclusion1648
1104.58000	exclusion1649
1105.54000	exclusion1650
1105.77000	exclusion1651
1106.53000	exclusion1652
1107.54000	exclusion1653
1108.05000	exclusion0260
1108.58000	exclusion1654
1109.52000	exclusion1655
1109.88000	exclusion1656
1110.23000	exclusion1657
1110.91000	exclusion0261
1111.54000	exclusion0262
1111.56000	exclusion1658
1111.87000	exclusion1659
1112.08000	exclusion1660
1112.80000	exclusion1661
1113.00000	exclusion1662
1113.52000	exclusion1663
1114.04000	exclusion0263
1114.85000	exclusion0264
1114.94000	exclusion1664
1115.55000	exclusion1665
1116.08000	exclusion1666
1116.88000	exclusion1667
1117.52000	exclusion1668
1118.09000	exclusion1669
1118.58000	exclusion1670
1118.86000	exclusion1671
1119.89000	exclusion1672
1120.52000	exclusion1673
1121.05000	exclusion1674
1121.56000	exclusion0265
1121.88000	exclusion0266
1122.19000	exclusion1675
1122.51000	exclusion1676
1123.21000	exclusion1677
1123.56000	exclusion1678
1124.57000	exclusion1679
1125.02000	exclusion1680
1125.54000	exclusion1681
1126.04000	exclusion1682
1126.54000	exclusion0267
1127.00000	exclusion0268
1127.21000	exclusion0269
1127.55000	exclusion1683
1127.87000	exclusion1684

1128.09000	exclusion1685
1128.53000	exclusion1686
1129.58000	exclusion0270
1130.12000	exclusion0271
1130.21000	exclusion1687
1130.54000	exclusion0272
1130.58000	exclusion1688
1131.03000	exclusion1689
1131.57000	exclusion1690
1132.20000	exclusion1691
1132.87000	exclusion1692
1133.08000	exclusion1693
1133.62000	exclusion1694
1133.89000	exclusion1695
1134.54000	exclusion1696
1135.11000	exclusion1697
1136.04000	exclusion1698
1136.53000	exclusion1699
1137.44000	exclusion1700
1138.55000	exclusion0273
1138.55000	exclusion1701
1139.18000	exclusion1702
1139.54000	exclusion1703
1140.20000	exclusion0274
1140.58000	exclusion0275
1140.83000	exclusion0276
1141.52000	exclusion1704
1141.90000	exclusion1705
1142.51000	exclusion1706
1143.05000	exclusion1707
1143.56000	exclusion1708
1144.09000	exclusion0277
1144.54000	exclusion0278
1145.04000	exclusion1709
1145.51000	exclusion0279
1145.53000	exclusion1710
1146.56000	exclusion1711
1147.00000	exclusion1712
1147.53000	exclusion1713
1148.07000	exclusion1714
1148.56000	exclusion1715
1149.06000	exclusion1716
1149.56000	exclusion1717
1150.56000	exclusion0280
1150.56000	exclusion1718
1151.05000	exclusion1719
1151.60000	exclusion1720
1152.12000	exclusion1721
1152.55000	exclusion1722
1153.54000	exclusion1723
1154.20000	exclusion1724

1154.55000	exclusion1725
1155.08000	exclusion1726
1156.07000	exclusion1727
1156.52000	exclusion1728
1157.56000	exclusion1729
1158.23000	exclusion1730
1158.51000	exclusion1731
1159.44000	exclusion1732
1159.64000	exclusion1733
1159.91000	exclusion1734
1161.55000	exclusion0281
1161.57000	exclusion1735
1162.04000	exclusion1736
1162.58000	exclusion1737
1163.03000	exclusion1738
1163.58000	exclusion0282
1163.89000	exclusion0283
1164.56000	exclusion1739
1165.57000	exclusion1740
1166.27000	exclusion0284
1166.53000	exclusion0285
1166.90000	exclusion0286
1167.20000	exclusion1741
1167.21000	exclusion0287
1167.52000	exclusion1742
1168.20000	exclusion1743
1168.55000	exclusion1744
1169.58000	exclusion1745
1170.08000	exclusion0288
1170.56000	exclusion0289
1171.09000	exclusion1746
1171.61000	exclusion1747
1172.54000	exclusion0290
1173.26000	exclusion0291
1173.27000	exclusion1748
1173.56000	exclusion1749
1174.54000	exclusion1750
1175.48000	exclusion1751
1176.00000	exclusion0292
1176.58000	exclusion0293
1176.59000	exclusion1752
1177.58000	exclusion1753
1178.09000	exclusion1754
1178.50000	exclusion1755
1179.08000	exclusion0294
1179.58000	exclusion1756
1180.14000	exclusion1757
1180.94000	exclusion0295
1181.56000	exclusion1758
1181.91000	exclusion0296
1182.54000	exclusion1759

1182.93000	exclusion1760
1183.53000	exclusion0297
1184.10000	exclusion1761
1184.55000	exclusion1762
1185.54000	exclusion1763
1186.47000	exclusion0298
1187.05000	exclusion0299
1187.10000	exclusion1764
1187.58000	exclusion1765
1188.57000	exclusion1766
1189.54000	exclusion1767
1189.91000	exclusion1768
1190.58000	exclusion0300
1191.08000	exclusion0301
1192.06000	exclusion0302
1192.62000	exclusion0303
1192.62000	exclusion1769
1192.89000	exclusion1770
1193.59000	exclusion1771
1194.23000	exclusion0304
1195.03000	exclusion0305
1195.03000	exclusion1772
1195.25000	exclusion1773
1196.25000	exclusion1774
1196.54000	exclusion1775
1197.55000	exclusion0306
1197.55000	exclusion1776
1198.55000	exclusion1777
1199.06000	exclusion0307
1200.23000	exclusion1778
1200.56000	exclusion1779
1200.57000	exclusion0308
1200.84000	exclusion1780
1201.11000	exclusion1781
1202.05000	exclusion0309
1202.57000	exclusion0310
1202.57000	exclusion1782
1203.50000	exclusion1783
1204.53000	exclusion0311
1204.54000	exclusion1784
1204.91000	exclusion1785
1205.63000	exclusion1786
1206.03000	exclusion1787
1206.59000	exclusion1788
1207.23000	exclusion0312
1207.88000	exclusion1789
1208.87000	exclusion1790
1209.91000	exclusion1791
1210.64000	exclusion1792
1211.01000	exclusion1793
1211.60000	exclusion1794

1212.25000	exclusion1795
1212.76000	exclusion0313
1213.52000	exclusion0314
1213.61000	exclusion1796
1214.58000	exclusion1797
1215.65000	exclusion1798
1216.12000	exclusion1799
1217.14000	exclusion0315
1217.61000	exclusion0316
1217.86000	exclusion1800
1218.54000	exclusion1801
1219.58000	exclusion1802
1220.22000	exclusion0317
1220.58000	exclusion0318
1221.12000	exclusion1803
1222.07000	exclusion0319
1223.05000	exclusion0320
1223.05000	exclusion1804
1223.57000	exclusion1805
1224.08000	exclusion1806
1224.59000	exclusion1807
1225.09000	exclusion0321
1225.60000	exclusion0322
1225.92000	exclusion1808
1226.62000	exclusion1809
1227.13000	exclusion1810
1228.28000	exclusion1811
1228.82000	exclusion1812
1229.35000	exclusion0323
1229.56000	exclusion0324
1230.06000	exclusion1813
1230.66000	exclusion0325
1231.11000	exclusion0326
1232.05000	exclusion1814
1232.59000	exclusion0327
1233.10000	exclusion0328
1233.30000	exclusion1815
1234.12000	exclusion1816
1235.07000	exclusion1817
1235.58000	exclusion1818
1237.06000	exclusion0329
1237.12000	exclusion1819
1237.95000	exclusion0330
1240.06000	exclusion0331
1241.05000	exclusion1820
1241.64000	exclusion1821
1241.87000	exclusion1822
1243.30000	exclusion0332
1244.09000	exclusion0333
1245.11000	exclusion1823
1245.92000	exclusion1824

1246.97000	exclusion1825
1247.36000	exclusion1826
1247.59000	exclusion1827
1248.57000	exclusion1828
1249.04000	exclusion1829
1249.65000	exclusion0334
1250.60000	exclusion0335
1250.60000	exclusion1830
1252.19000	exclusion1831
1252.20000	exclusion0336
1252.59000	exclusion1832
1253.07000	exclusion1833
1253.59000	exclusion1834
1254.63000	exclusion1835
1255.14000	exclusion0337
1256.59000	exclusion0338
1256.79000	exclusion1836
1257.09000	exclusion0339
1257.09000	exclusion1837
1257.64000	exclusion0340
1258.57000	exclusion1838
1259.21000	exclusion1839
1260.13000	exclusion0341
1260.63000	exclusion1840
1260.64000	exclusion0342
1261.60000	exclusion0343
1263.27000	exclusion0344
1264.07000	exclusion0345
1264.16000	exclusion1841
1265.08000	exclusion1842
1265.59000	exclusion1843
1265.97000	exclusion1844
1266.61000	exclusion0346
1267.25000	exclusion0347
1268.19000	exclusion0348
1268.81000	exclusion0349
1269.15000	exclusion0350
1270.26000	exclusion0351
1270.27000	exclusion1845
1271.18000	exclusion1846
1272.11000	exclusion0352
1272.67000	exclusion0353
1272.68000	exclusion1847
1274.07000	exclusion0354
1275.16000	exclusion0355
1275.62000	exclusion0356
1275.65000	exclusion1848
1276.64000	exclusion1849
1277.14000	exclusion0357
1277.67000	exclusion0358
1278.46000	exclusion0359

1279.01000	exclusion0360
1279.02000	exclusion1850
1279.61000	exclusion1851
1283.66000	exclusion0361
1283.66000	exclusion1852
1284.64000	exclusion1853
1286.65000	exclusion0362
1286.66000	exclusion1854
1287.08000	exclusion1855
1287.66000	exclusion0363
1288.63000	exclusion0364
1288.64000	exclusion1856
1289.45000	exclusion1857
1291.08000	exclusion1858
1292.39000	exclusion1859
1293.18000	exclusion1860
1294.10000	exclusion0365
1294.61000	exclusion1861
1295.33000	exclusion1862
1296.64000	exclusion0366
1298.08000	exclusion1863
1298.58000	exclusion0367
1299.13000	exclusion0368
1300.07000	exclusion1864
1301.06000	exclusion1865
1301.57000	exclusion0369
1302.09000	exclusion1866
1302.10000	exclusion0370
1304.08000	exclusion0371
1304.09000	exclusion1867
1304.65000	exclusion0372
1305.69000	exclusion0373
1306.06000	exclusion0374
1307.12000	exclusion1868
1308.00000	exclusion0375
1309.07000	exclusion0376
1310.67000	exclusion0377
1311.63000	exclusion0378
1312.61000	exclusion1869
1312.67000	exclusion0379
1313.62000	exclusion0380
1313.62000	exclusion1870
1314.61000	exclusion0381
1315.13000	exclusion0382
1315.59000	exclusion1871
1315.69000	exclusion0383
1316.64000	exclusion0384
1318.03000	exclusion0385
1318.61000	exclusion0386
1319.74000	exclusion1872
1320.09000	exclusion0387

1320.66000	exclusion1873
1321.12000	exclusion0388
1321.12000	exclusion1874
1322.61000	exclusion0389
1323.60000	exclusion0390
1324.18000	exclusion1875
1324.61000	exclusion1876
1325.11000	exclusion0391
1326.10000	exclusion0392
1328.19000	exclusion0393
1328.25000	exclusion1877
1330.61000	exclusion0394
1331.19000	exclusion0395
1331.20000	exclusion1878
1331.82000	exclusion0396
1332.12000	exclusion0397
1332.19000	exclusion1879
1332.65000	exclusion0398
1333.65000	exclusion0399
1333.65000	exclusion1880
1334.06000	exclusion0400
1335.19000	exclusion0401
1335.67000	exclusion0402
1335.68000	exclusion1881
1336.17000	exclusion0403
1337.18000	exclusion1882
1337.19000	exclusion0404
1339.66000	exclusion0405
1339.68000	exclusion1883
1341.16000	exclusion0406
1341.71000	exclusion1884
1342.13000	exclusion0407
1342.13000	exclusion1885
1343.68000	exclusion0408
1344.18000	exclusion1886
1344.63000	exclusion0409
1346.20000	exclusion1887
1346.61000	exclusion1888
1346.62000	exclusion0410
1347.12000	exclusion0411
1347.63000	exclusion1889
1348.36000	exclusion0412
1348.36000	exclusion1890
1348.67000	exclusion0413
1349.10000	exclusion0414
1349.63000	exclusion0415
1351.64000	exclusion0416
1352.66000	exclusion0417
1352.66000	exclusion1891
1354.16000	exclusion0418
1354.19000	exclusion1892

1354.68000	exclusion0419
1355.21000	exclusion0420
1355.61000	exclusion0421
1355.65000	exclusion1893
1356.17000	exclusion0422
1356.66000	exclusion0423
1356.72000	exclusion1894
1357.19000	exclusion1895
1357.61000	exclusion0424
1359.64000	exclusion0425
1360.23000	exclusion0426
1361.19000	exclusion1896
1361.70000	exclusion0427
1363.66000	exclusion0428
1364.21000	exclusion0429
1365.18000	exclusion1897
1365.19000	exclusion0430
1365.69000	exclusion0431
1366.64000	exclusion0432
1367.70000	exclusion0433
1368.17000	exclusion0434
1368.17000	exclusion1898
1368.69000	exclusion1899
1368.74000	exclusion0435
1371.20000	exclusion0436
1372.19000	exclusion0437
1373.15000	exclusion0438
1374.18000	exclusion0439
1374.38000	exclusion1900
1374.68000	exclusion1901
1375.70000	exclusion0440
1376.17000	exclusion0441
1376.21000	exclusion1902
1376.68000	exclusion0442
1377.18000	exclusion1903
1377.19000	exclusion0443
1380.13000	exclusion0444
1380.13000	exclusion1904
1381.15000	exclusion0445
1381.15000	exclusion1905
1382.04000	exclusion0446
1382.71000	exclusion1906
1382.72000	exclusion0447
1383.62000	exclusion0448
1384.69000	exclusion0449
1384.69000	exclusion1907
1385.11000	exclusion1908
1385.12000	exclusion0450
1387.20000	exclusion0451
1387.20000	exclusion1909
1388.70000	exclusion0452

1389.68000	exclusion0453
1390.19000	exclusion1910
1392.67000	exclusion0454
1392.69000	exclusion1911
1395.15000	exclusion0455
1395.16000	exclusion1912
1395.68000	exclusion0456
1395.68000	exclusion1913
1396.70000	exclusion0457
1397.11000	exclusion1914
1397.12000	exclusion0458
1397.73000	exclusion0459
1398.71000	exclusion1915
1399.21000	exclusion0460
1399.69000	exclusion1916
1399.70000	exclusion0461
1402.16000	exclusion0462
1402.16000	exclusion1917
1402.79000	exclusion0463
1403.67000	exclusion0464
1404.17000	exclusion1918
1404.44000	exclusion0465
1405.23000	exclusion0466
1405.23000	exclusion1919
1406.31000	exclusion1920
1406.33000	exclusion0467
1406.66000	exclusion0468
1407.11000	exclusion0469
1407.12000	exclusion1921
1408.36000	exclusion0470
1413.19000	exclusion1922
1413.22000	exclusion0471
1413.64000	exclusion0472
1413.71000	exclusion1923
1415.02000	exclusion0473
1415.68000	exclusion0474
1415.69000	exclusion1924
1417.66000	exclusion0475
1417.68000	exclusion1925
1418.03000	exclusion0476
1419.37000	exclusion0477
1419.38000	exclusion1926
1420.34000	exclusion0478
1420.93000	exclusion1927
1421.12000	exclusion0479
1422.22000	exclusion1928
1422.70000	exclusion0480
1423.71000	exclusion0481
1424.20000	exclusion0482
1425.33000	exclusion0483
1425.68000	exclusion0484

1427.97000	exclusion0485
1428.67000	exclusion0486
1429.32000	exclusion1929
1430.20000	exclusion0487
1431.14000	exclusion1930
1432.34000	exclusion0488
1432.69000	exclusion0489
1433.21000	exclusion0490
1433.71000	exclusion1931
1435.72000	exclusion0491
1436.21000	exclusion0492
1437.23000	exclusion0493
1440.25000	exclusion0494
1440.73000	exclusion1932
1440.79000	exclusion0495
1441.23000	exclusion0496
1442.71000	exclusion0497
1442.72000	exclusion1933
1443.66000	exclusion0498
1444.21000	exclusion0499
1445.74000	exclusion0500
1445.74000	exclusion1934
1446.19000	exclusion0501
1448.17000	exclusion0502
1449.06000	exclusion0503
1450.25000	exclusion1935
1450.72000	exclusion0504
1451.24000	exclusion1936
1453.70000	exclusion0505
1454.23000	exclusion1937
1456.72000	exclusion0506
1456.74000	exclusion1938
1457.24000	exclusion1939
1458.26000	exclusion0507
1458.26000	exclusion1940
1459.21000	exclusion0508
1460.09000	exclusion0509
1460.42000	exclusion1941
1460.75000	exclusion1942
1461.23000	exclusion0510
1461.70000	exclusion0511
1461.71000	exclusion1943
1463.72000	exclusion0512
1465.73000	exclusion1944
1465.74000	exclusion0513
1467.73000	exclusion1945
1468.23000	exclusion0514
1471.31000	exclusion1946
1471.73000	exclusion0515
1473.73000	exclusion0516
1473.74000	exclusion1947

1474.23000	exclusion0517
1479.23000	exclusion0518
1479.23000	exclusion1948
1479.71000	exclusion0519
1479.72000	exclusion1949
1480.24000	exclusion0520
1481.77000	exclusion0521
1482.27000	exclusion1950
1485.74000	exclusion0522
1486.23000	exclusion1951
1487.33000	exclusion0523
1489.72000	exclusion0524
1489.73000	exclusion1952
1490.72000	exclusion1953
1490.73000	exclusion0525
1492.77000	exclusion0526
1492.77000	exclusion1954
1493.77000	exclusion0527
1497.22000	exclusion1955
1497.73000	exclusion0528
1498.24000	exclusion1956
1498.73000	exclusion0529
1504.74000	exclusion0530
1507.24000	exclusion0531
1507.24000	exclusion1957
1507.74000	exclusion0532
1509.24000	exclusion0533
1510.28000	exclusion1958
1510.29000	exclusion0534
1512.19000	exclusion0535
1512.69000	exclusion1959
1512.70000	exclusion0536
1514.74000	exclusion1960
1515.24000	exclusion0537
1518.25000	exclusion0538
1519.22000	exclusion0539
1520.79000	exclusion0540
1522.20000	exclusion0541
1524.25000	exclusion0542
1524.25000	exclusion1961
1528.30000	exclusion0543
1528.32000	exclusion1962
1532.74000	exclusion0544
1532.76000	exclusion1963
1535.75000	exclusion0545
1537.22000	exclusion0546
1537.29000	exclusion1964
1537.73000	exclusion0547
1543.74000	exclusion0548
1544.29000	exclusion1965
1553.26000	exclusion0549

1553.78000	exclusion0550
1553.80000	exclusion1966
1555.25000	exclusion0551
1559.76000	exclusion0552
1561.27000	exclusion0553
1561.75000	exclusion1967
1563.30000	exclusion1968
1564.79000	exclusion0554
1565.79000	exclusion0555
1568.27000	exclusion0556
1568.27000	exclusion1969
1569.27000	exclusion0557
1570.29000	exclusion0558
1572.26000	exclusion0559
1572.26000	exclusion1970
1574.30000	exclusion1971
1575.32000	exclusion1972
1575.80000	exclusion0560
1576.43000	exclusion0561
1579.25000	exclusion0562
1579.26000	exclusion1973
1580.48000	exclusion0563
1580.51000	exclusion1974
1583.23000	exclusion0564
1584.31000	exclusion0565
1584.32000	exclusion1975
1589.77000	exclusion0566
1589.78000	exclusion1976
1592.33000	exclusion0567
1596.28000	exclusion0568
1599.72000	exclusion0569
1635.31000	exclusion1977
1635.33000	exclusion0570
1643.75000	exclusion0571
1648.81000	exclusion0572
1654.77000	exclusion0573
1656.31000	exclusion0574
1656.32000	exclusion1978
1662.32000	exclusion0575
1662.32000	exclusion1979
1663.84000	exclusion0576
1664.33000	exclusion0577
1667.28000	exclusion1980
1667.33000	exclusion0578
1668.30000	exclusion0579
1671.31000	exclusion0580
1671.84000	exclusion1981
1673.32000	exclusion1982
1673.35000	exclusion0581
1675.31000	exclusion1983
1675.33000	exclusion0582

1677.31000	exclusion0583
1677.81000	exclusion0584
1678.32000	exclusion1984
1679.40000	exclusion0585
1679.80000	exclusion1985
1682.80000	exclusion0586
1683.30000	exclusion0587
1684.32000	exclusion0588
1684.32000	exclusion1986
1684.85000	exclusion1987
1686.39000	exclusion0589
1687.38000	exclusion1988
1687.81000	exclusion0590
1688.81000	exclusion0591
1690.39000	exclusion0592
1690.84000	exclusion1989
1698.33000	exclusion0593
1698.34000	exclusion1990
1700.37000	exclusion0594
1701.33000	exclusion1991
1701.81000	exclusion0595
1707.86000	exclusion0596
1708.36000	exclusion1992
1760.89000	exclusion0597
1760.89000	exclusion1993
1764.34000	exclusion0598
1773.89000	exclusion0599
1773.89000	exclusion1994
1775.34000	exclusion0600
1780.39000	exclusion0601
1780.89000	exclusion1995
1784.88000	exclusion1996
1784.89000	exclusion0602
1791.88000	exclusion0603
1791.88000	exclusion1997
1802.39000	exclusion0604
1809.40000	exclusion0605

Neutral Loss Mass List: (none)
Product Mass List: (none)
Neutral loss in top: 3
Product in top: 3
Most intense if no parent masses found not enabled
Add/subtract mass not enabled
FT master scan preview mode enabled
Charge state screening enabled
Charge state dependent ETD time not enabled
Monoisotopic precursor selection enabled
Charge state rejection enabled
Unassigned charge states : rejected
Charge state 1 : rejected
Charge state 2 : not rejected

Charge state 3 : not rejected
Charge states 4+ : not rejected
Chromatography mode is disabled
Global Data Dependent Settings:
Predict ion injection time enabled
Use global parent and reject mass lists not enabled
Exclude parent mass from data dependent selection not enabled
Exclusion mass width by mass
Exclusion mass width low: 1.50000
Exclusion mass width high: 1.50000
Parent mass width by mass
Parent mass width low: 0.50000
Parent mass width high: 0.50000
Reject mass width by mass
Reject mass width low: 0.50000
Reject mass width high: 0.50000
Zoom/UltraZoom scan mass width by mass
Zoom/UltraZoom scan mass width low: 5.00
Zoom/UltraZoom scan mass width high: 5.00
FT SIM scan mass width low: 5.00
FT SIM scan mass width high: 5.00
Neutral Loss candidates processed by decreasing intensity
Neutral Loss mass width by mass
Neutral Loss mass width low: 0.50000
Neutral Loss mass width high: 0.50000
Product candidates processed by decreasing intensity
Product mass width by mass
Product mass width low: 0.50000
Product mass width high: 0.50000
MS mass range: 300.00-2000.00
MSn mass range by mass
MSn mass range: 0.00-1000000.00
Use m/z values as masses not enabled
Analog UV data dep. not enabled
Dynamic exclusion enabled
Repeat Count: 1
Repeat Duration: 30.00
Exclusion List Size: 500
Exclusion Duration: 180.00
Exclusion mass width by mass
Exclusion mass width low: 1.50000
Exclusion mass width high: 1.50000
Expiration: disabled
Isotopic data dependence not enabled
Custom Data Dependent Settings:
Not enabled

Pass 4 (wheat-mixed-digests_MS2_pass04_1.raw):

Creator: Orbi_30393
Last modified: 10/12/2021 by Orbi_30393

MS Run Time (min): 43.00
Sequence override of method parameters not enabled.
Divert Valve: not used during run
Contact Closure: not used during run
Syringe Pump: not used during run
MS Detector Settings:
Real-time modifications to method not enabled
Stepped collision energy not enabled
Additional Microscans:

MS2	0	0
MS3	0	0
MS4	0	0
MS5	0	0
MS6	0	0
MS7	0	0
MS8	0	0
MS9	0	0
MS10	0	0

Experiment Type: Nth Order Double Play
Tune Method: Orbitrap-tune-file_2020-03-13_HESI
Scan Event Details:
1: FTMS + p norm o(300.0-2000.0)
CV = 0.0V
2: ITMS + c norm Dep MS/MS Most intense ion from (1)
Activation Type: CID
Min. Signal Required: 3000.0
Isolation Width: 2.00
Normalized Coll. Energy: 35.0
Default Charge State: 2
Activation Q: 0.250
Activation Time: 10.000
CV = 0.0V
Scan Event 2 repeated for top 10 peaks.
Lock Masses:
Pos List Name: N/A
Source: API Source
Mass List: (none)
Neg List Name: N/A
Source: API Source
Mass List: (none)
Data Dependent Settings:
Use separate polarity settings disabled
Parent Mass List: (none)
Reject Mass List:

MS Mass	Name
300.14000	exclusion0001
300.19000	exclusion0002
300.71000	exclusion0003
301.67000	exclusion0004
302.19000	exclusion0005
302.20000	exclusion0006

302.59000	exclusion0007
302.80000	exclusion0008
303.84000	exclusion0009
304.11000	exclusion0010
304.16000	exclusion0011
304.39000	exclusion0012
304.66000	exclusion0013
305.81000	exclusion0014
306.18000	exclusion0015
306.50000	exclusion0016
306.84000	exclusion0017
308.14000	exclusion0018
308.66000	exclusion0925
308.70000	exclusion0019
309.18000	exclusion0020
309.48000	exclusion0021
309.67000	exclusion0022
310.46000	exclusion0023
311.10000	exclusion0926
311.67000	exclusion0024
312.17000	exclusion0025
312.50000	exclusion0026
313.17000	exclusion0927
313.72000	exclusion0027
314.70000	exclusion0028
314.86000	exclusion0029
315.47000	exclusion0928
315.70000	exclusion0929
316.87000	exclusion0030
317.16000	exclusion0031
317.64000	exclusion0032
318.16000	exclusion0033
318.49000	exclusion0034
318.69000	exclusion0035
319.14000	exclusion0036
320.14000	exclusion0037
320.49000	exclusion0038
320.66000	exclusion0039
320.71000	exclusion0040
321.24000	exclusion0930
321.66000	exclusion0931
321.68000	exclusion0932
322.20000	exclusion0041
322.57000	exclusion0042
324.15000	exclusion0043
324.18000	exclusion0044
325.70000	exclusion0045
325.85000	exclusion0046
326.18000	exclusion0047
327.01000	exclusion0048
327.67000	exclusion0049

328.11000	exclusion0050
328.69000	exclusion0933
329.71000	exclusion0051
330.19000	exclusion0052
330.19000	exclusion0053
330.83000	exclusion0934
332.14000	exclusion0054
332.20000	exclusion0055
332.69000	exclusion0056
333.15000	exclusion0057
333.42000	exclusion0058
333.83000	exclusion0059
334.52000	exclusion0060
335.12000	exclusion0061
335.18000	exclusion0062
335.68000	exclusion0935
336.19000	exclusion0063
336.73000	exclusion0064
337.13000	exclusion0065
337.51000	exclusion0066
337.84000	exclusion0067
338.67000	exclusion0936
338.69000	exclusion0937
339.17000	exclusion0938
339.68000	exclusion0068
340.18000	exclusion0069
340.69000	exclusion0939
340.83000	exclusion0940
341.53000	exclusion0070
341.66000	exclusion0071
341.84000	exclusion0072
341.91000	exclusion0073
342.84000	exclusion0941
343.49000	exclusion0074
343.73000	exclusion0075
344.23000	exclusion0076
344.50000	exclusion0077
345.13000	exclusion0942
345.15000	exclusion0943
345.70000	exclusion0078
346.01000	exclusion0079
346.19000	exclusion0080
346.22000	exclusion0081
346.71000	exclusion0944
346.85000	exclusion0945
346.93000	exclusion0946
347.17000	exclusion0947
347.68000	exclusion0082
347.85000	exclusion0083
348.21000	exclusion0084
348.60000	exclusion0085

348.65000	exclusion0086
349.23000	exclusion0087
349.68000	exclusion0088
350.42000	exclusion0089
350.67000	exclusion0090
351.19000	exclusion0091
351.40000	exclusion0092
351.71000	exclusion0093
352.17000	exclusion0094
352.21000	exclusion0095
352.65000	exclusion0096
353.18000	exclusion0948
353.19000	exclusion0949
353.70000	exclusion0097
353.80000	exclusion0098
353.92000	exclusion0099
354.21000	exclusion0100
354.52000	exclusion0101
354.64000	exclusion0102
355.55000	exclusion0103
355.86000	exclusion0104
356.14000	exclusion0105
356.69000	exclusion0950
356.87000	exclusion0951
357.19000	exclusion0952
357.23000	exclusion0953
358.22000	exclusion0106
358.50000	exclusion0107
358.71000	exclusion0108
358.84000	exclusion0109
359.83000	exclusion0110
360.36000	exclusion0111
360.70000	exclusion0112
361.42000	exclusion0113
361.69000	exclusion0114
362.12000	exclusion0115
363.20000	exclusion0116
363.66000	exclusion0117
363.83000	exclusion0118
364.13000	exclusion0119
364.71000	exclusion0120
364.86000	exclusion0121
365.23000	exclusion0122
365.56000	exclusion0123
365.80000	exclusion0124
366.42000	exclusion0125
366.70000	exclusion0126
367.21000	exclusion0127
367.22000	exclusion0128
367.49000	exclusion0129
367.70000	exclusion0130

368.22000	exclusion0954
368.86000	exclusion0955
369.16000	exclusion0956
369.26000	exclusion0957
369.86000	exclusion0131
370.18000	exclusion0132
370.77000	exclusion0133
371.17000	exclusion0134
371.54000	exclusion0135
371.67000	exclusion0136
372.22000	exclusion0958
372.23000	exclusion0959
372.83000	exclusion0137
373.19000	exclusion0138
373.19000	exclusion0139
374.23000	exclusion0140
374.67000	exclusion0141
375.21000	exclusion0142
375.71000	exclusion0143
376.19000	exclusion0144
376.69000	exclusion0960
377.68000	exclusion0961
378.22000	exclusion0962
378.22000	exclusion0963
378.52000	exclusion0964
379.20000	exclusion0965
379.71000	exclusion0145
379.87000	exclusion0146
380.19000	exclusion0147
380.70000	exclusion0966
380.80000	exclusion0148
381.24000	exclusion0149
382.17000	exclusion0150
382.19000	exclusion0151
382.85000	exclusion0152
383.20000	exclusion0153
383.33000	exclusion0154
383.49000	exclusion0155
384.17000	exclusion0156
384.52000	exclusion0157
385.17000	exclusion0158
385.54000	exclusion0159
385.70000	exclusion0160
386.19000	exclusion0161
386.25000	exclusion0162
386.58000	exclusion0163
387.21000	exclusion0164
387.72000	exclusion0165
388.20000	exclusion0166
388.23000	exclusion0167
388.50000	exclusion0168

388.72000	exclusion0169
388.93000	exclusion0170
389.89000	exclusion0967
390.19000	exclusion0968
390.94000	exclusion0171
391.21000	exclusion0172
391.22000	exclusion0173
392.25000	exclusion0969
392.87000	exclusion0174
393.51000	exclusion0175
393.73000	exclusion0176
394.75000	exclusion0177
394.89000	exclusion0178
395.20000	exclusion0179
395.24000	exclusion0180
395.55000	exclusion0181
395.70000	exclusion0182
396.18000	exclusion0970
396.20000	exclusion0971
397.13000	exclusion0183
397.21000	exclusion0184
397.39000	exclusion0185
397.68000	exclusion0186
397.71000	exclusion0187
398.54000	exclusion0188
399.18000	exclusion0189
399.22000	exclusion0190
399.53000	exclusion0191
400.76000	exclusion0192
400.90000	exclusion0193
401.23000	exclusion0194
401.34000	exclusion0195
401.41000	exclusion0196
401.66000	exclusion0197
402.23000	exclusion0972
402.25000	exclusion0198
402.44000	exclusion0973
402.53000	exclusion0974
403.22000	exclusion0975
403.72000	exclusion0199
404.19000	exclusion0200
404.54000	exclusion0201
405.52000	exclusion0202
405.73000	exclusion0203
406.22000	exclusion0204
407.19000	exclusion0205
407.19000	exclusion0206
407.19000	exclusion0207
407.76000	exclusion0976
408.19000	exclusion0977
409.20000	exclusion0978

409.23000	exclusion0979
409.86000	exclusion0208
410.21000	exclusion0209
410.72000	exclusion0210
411.21000	exclusion0211
411.36000	exclusion0212
412.75000	exclusion0980
413.23000	exclusion0981
413.77000	exclusion0213
414.22000	exclusion0214
414.52000	exclusion0215
414.73000	exclusion0216
414.87000	exclusion0217
415.76000	exclusion0218
416.71000	exclusion0982
416.71000	exclusion0983
417.22000	exclusion0219
417.22000	exclusion0220
417.70000	exclusion0221
417.95000	exclusion0222
418.28000	exclusion0223
418.57000	exclusion0224
418.73000	exclusion0225
419.20000	exclusion0226
419.21000	exclusion0227
420.22000	exclusion0984
420.93000	exclusion0228
421.26000	exclusion0229
421.72000	exclusion0230
421.85000	exclusion0231
422.25000	exclusion0232
423.21000	exclusion0233
423.44000	exclusion0234
423.70000	exclusion0235
423.73000	exclusion0236
423.96000	exclusion0237
424.24000	exclusion0238
424.69000	exclusion0239
425.72000	exclusion0240
425.96000	exclusion0241
426.24000	exclusion0242
426.73000	exclusion0243
427.77000	exclusion0244
427.96000	exclusion0245
428.54000	exclusion0246
428.71000	exclusion0247
429.96000	exclusion0248
430.25000	exclusion0249
430.54000	exclusion0250
430.75000	exclusion0251
431.27000	exclusion0985

432.46000	exclusion0252
432.69000	exclusion0253
433.01000	exclusion0254
433.23000	exclusion0255
433.24000	exclusion0256
433.74000	exclusion0257
433.86000	exclusion0258
434.72000	exclusion0986
435.73000	exclusion0987
436.29000	exclusion0259
436.43000	exclusion0260
436.53000	exclusion0261
436.74000	exclusion0262
437.23000	exclusion0263
438.20000	exclusion0988
438.24000	exclusion0989
439.24000	exclusion0990
440.21000	exclusion0991
440.26000	exclusion0992
441.21000	exclusion0993
441.71000	exclusion0264
442.25000	exclusion0265
442.25000	exclusion0994
442.90000	exclusion0266
443.23000	exclusion0267
443.26000	exclusion0268
443.71000	exclusion0269
443.92000	exclusion0270
444.23000	exclusion0271
444.26000	exclusion0272
444.76000	exclusion0995
444.87000	exclusion0996
445.24000	exclusion0997
446.23000	exclusion0998
446.75000	exclusion0273
447.24000	exclusion0274
448.20000	exclusion0275
448.63000	exclusion0276
448.97000	exclusion0277
449.19000	exclusion0278
449.27000	exclusion0279
449.58000	exclusion0280
450.24000	exclusion0281
450.45000	exclusion0282
450.55000	exclusion0283
450.73000	exclusion0284
451.19000	exclusion0285
451.22000	exclusion0286
451.88000	exclusion0287
452.24000	exclusion0288
452.72000	exclusion0289

452.92000	exclusion0290
453.22000	exclusion0291
453.47000	exclusion0292
454.26000	exclusion0293
454.71000	exclusion0294
455.22000	exclusion0295
455.23000	exclusion0296
455.24000	exclusion0297
455.72000	exclusion0298
456.25000	exclusion0299
456.74000	exclusion0300
457.73000	exclusion0301
457.89000	exclusion0302
458.23000	exclusion0303
458.26000	exclusion0304
459.02000	exclusion0305
459.22000	exclusion0306
459.57000	exclusion0307
459.74000	exclusion0308
460.76000	exclusion0309
461.25000	exclusion0310
461.62000	exclusion0311
461.72000	exclusion0312
462.20000	exclusion0313
462.91000	exclusion0314
463.23000	exclusion0315
463.28000	exclusion0316
463.73000	exclusion0317
464.29000	exclusion0318
464.52000	exclusion0319
464.73000	exclusion0320
464.76000	exclusion0321
465.19000	exclusion0322
466.24000	exclusion0323
466.72000	exclusion0324
467.23000	exclusion0999
467.23000	exclusion1000
467.76000	exclusion0325
467.88000	exclusion0326
468.22000	exclusion0327
468.74000	exclusion0328
469.24000	exclusion0329
469.73000	exclusion0330
469.78000	exclusion0331
469.87000	exclusion0332
470.23000	exclusion0333
470.26000	exclusion0334
470.78000	exclusion1001
470.91000	exclusion1002
471.54000	exclusion0335
471.78000	exclusion0336

472.22000	exclusion0337
472.71000	exclusion0338
473.27000	exclusion0339
473.58000	exclusion0340
474.21000	exclusion0341
474.21000	exclusion0342
474.76000	exclusion1003
475.22000	exclusion1004
475.75000	exclusion0343
475.95000	exclusion0344
476.23000	exclusion0345
476.70000	exclusion0346
476.73000	exclusion0347
477.22000	exclusion0348
477.26000	exclusion0349
477.92000	exclusion0350
478.21000	exclusion0351
478.78000	exclusion1005
478.90000	exclusion1006
479.48000	exclusion0352
479.77000	exclusion0353
479.91000	exclusion0354
480.23000	exclusion0355
480.24000	exclusion0356
480.58000	exclusion0357
481.22000	exclusion0358
481.31000	exclusion0359
481.48000	exclusion0360
481.75000	exclusion0361
482.27000	exclusion0362
483.24000	exclusion0363
483.25000	exclusion0364
483.75000	exclusion0365
484.25000	exclusion0366
484.29000	exclusion0367
484.57000	exclusion0368
484.91000	exclusion0369
485.20000	exclusion0370
485.28000	exclusion0371
486.25000	exclusion0372
486.62000	exclusion0373
486.77000	exclusion0374
486.88000	exclusion0375
487.24000	exclusion0376
487.75000	exclusion0377
488.21000	exclusion0378
488.22000	exclusion0379
488.74000	exclusion0380
489.24000	exclusion0381
489.25000	exclusion0382
489.55000	exclusion0383

489.95000	exclusion0384
490.23000	exclusion0385
490.26000	exclusion0386
490.78000	exclusion0387
491.05000	exclusion0388
491.25000	exclusion0389
491.26000	exclusion0390
491.74000	exclusion0391
492.25000	exclusion0392
492.50000	exclusion0393
493.23000	exclusion0394
493.25000	exclusion0395
493.76000	exclusion0396
494.26000	exclusion0397
494.77000	exclusion0398
494.92000	exclusion0399
495.27000	exclusion0400
495.72000	exclusion0401
495.76000	exclusion0402
496.19000	exclusion0403
496.74000	exclusion1007
497.49000	exclusion1008
498.24000	exclusion0404
498.27000	exclusion0405
498.76000	exclusion0406
499.26000	exclusion0407
499.55000	exclusion0408
499.80000	exclusion0409
499.95000	exclusion0410
500.56000	exclusion0411
500.70000	exclusion0412
500.79000	exclusion0413
501.21000	exclusion0414
501.26000	exclusion0415
501.57000	exclusion0416
501.78000	exclusion0417
502.24000	exclusion0418
502.76000	exclusion0419
503.11000	exclusion0420
503.25000	exclusion0421
503.59000	exclusion0422
503.71000	exclusion0423
504.22000	exclusion0424
504.23000	exclusion0425
505.23000	exclusion0426
505.25000	exclusion0427
505.58000	exclusion0428
505.94000	exclusion0429
506.26000	exclusion0430
506.27000	exclusion0431
506.81000	exclusion0432

506.91000	exclusion0433
507.25000	exclusion0434
507.27000	exclusion0435
507.80000	exclusion1009
507.88000	exclusion1010
507.99000	exclusion1011
508.26000	exclusion1012
508.78000	exclusion0436
509.24000	exclusion0437
509.26000	exclusion0438
509.29000	exclusion0439
509.76000	exclusion1013
510.28000	exclusion1014
510.76000	exclusion1015
511.26000	exclusion0440
511.26000	exclusion0441
511.76000	exclusion0442
512.23000	exclusion0443
512.28000	exclusion0444
512.56000	exclusion0445
512.75000	exclusion0446
513.24000	exclusion0447
513.63000	exclusion0448
513.77000	exclusion0449
514.57000	exclusion0450
514.81000	exclusion0451
515.28000	exclusion0452
515.78000	exclusion0453
515.92000	exclusion0454
516.23000	exclusion0455
516.80000	exclusion0456
517.24000	exclusion0457
517.25000	exclusion0458
517.73000	exclusion0459
518.24000	exclusion0460
518.65000	exclusion0461
518.78000	exclusion0462
519.14000	exclusion0463
519.73000	exclusion0464
519.93000	exclusion0465
520.31000	exclusion0466
520.92000	exclusion0467
521.02000	exclusion0468
521.24000	exclusion0469
521.53000	exclusion0470
521.76000	exclusion0471
522.24000	exclusion0472
522.60000	exclusion0473
522.77000	exclusion0474
523.29000	exclusion0475
523.52000	exclusion0476

524.22000	exclusion0477
524.59000	exclusion0478
524.77000	exclusion0479
524.81000	exclusion0480
524.89000	exclusion0481
525.24000	exclusion0482
526.21000	exclusion0483
526.44000	exclusion0484
526.59000	exclusion0485
526.77000	exclusion0486
527.76000	exclusion0487
528.24000	exclusion0488
529.23000	exclusion0489
529.79000	exclusion0490
530.26000	exclusion0491
530.30000	exclusion0492
530.74000	exclusion0493
531.29000	exclusion0494
531.75000	exclusion0495
532.25000	exclusion0496
532.58000	exclusion0497
532.77000	exclusion1016
533.28000	exclusion0498
533.63000	exclusion0499
533.76000	exclusion0500
533.90000	exclusion0501
534.28000	exclusion0502
534.71000	exclusion0503
535.27000	exclusion0504
535.59000	exclusion0505
535.76000	exclusion0506
536.09000	exclusion0507
536.28000	exclusion0508
536.33000	exclusion0509
536.51000	exclusion0510
537.79000	exclusion0511
537.80000	exclusion0512
537.95000	exclusion0513
538.29000	exclusion0514
538.77000	exclusion0515
538.78000	exclusion0516
539.30000	exclusion1017
539.72000	exclusion1018
540.27000	exclusion1019
540.78000	exclusion0517
540.94000	exclusion0518
541.26000	exclusion0519
541.76000	exclusion0520
542.31000	exclusion0521
542.78000	exclusion0522
542.92000	exclusion0523

543.27000	exclusion0524
543.32000	exclusion0525
543.76000	exclusion0526
543.95000	exclusion0527
544.22000	exclusion0528
544.30000	exclusion0529
544.51000	exclusion0530
544.76000	exclusion1020
545.25000	exclusion1021
545.76000	exclusion0531
546.02000	exclusion0532
546.28000	exclusion0533
546.61000	exclusion0534
546.75000	exclusion0535
547.28000	exclusion0536
547.31000	exclusion0537
547.61000	exclusion0538
547.77000	exclusion0539
548.24000	exclusion0540
548.77000	exclusion1022
548.79000	exclusion0541
549.27000	exclusion0542
549.75000	exclusion0543
549.92000	exclusion0544
549.98000	exclusion0545
550.26000	exclusion0546
550.78000	exclusion0547
551.28000	exclusion0548
551.78000	exclusion0549
551.95000	exclusion0550
552.04000	exclusion0551
552.29000	exclusion0552
552.74000	exclusion0553
553.28000	exclusion0554
553.30000	exclusion0555
553.59000	exclusion0556
553.77000	exclusion0557
553.82000	exclusion0558
554.37000	exclusion0559
554.75000	exclusion0560
555.29000	exclusion0561
555.31000	exclusion0562
555.61000	exclusion0563
555.64000	exclusion0564
555.75000	exclusion0565
556.90000	exclusion0566
557.26000	exclusion0567
557.30000	exclusion0568
558.28000	exclusion0569
558.77000	exclusion0570
558.78000	exclusion0571

559.06000	exclusion0572
559.27000	exclusion0573
559.56000	exclusion0574
559.80000	exclusion0575
560.78000	exclusion0576
561.29000	exclusion0577
561.30000	exclusion0578
561.57000	exclusion0579
561.79000	exclusion0580
562.30000	exclusion0581
562.61000	exclusion0582
562.78000	exclusion0583
563.30000	exclusion0584
563.78000	exclusion0585
564.27000	exclusion0586
564.29000	exclusion0587
564.58000	exclusion0588
564.82000	exclusion0589
565.42000	exclusion0590
565.59000	exclusion0591
566.22000	exclusion0592
566.58000	exclusion0593
566.76000	exclusion0594
566.86000	exclusion0595
567.29000	exclusion0596
567.59000	exclusion0597
567.81000	exclusion0598
568.29000	exclusion0599
568.31000	exclusion0600
568.75000	exclusion0601
569.31000	exclusion0602
569.81000	exclusion0603
570.08000	exclusion0604
570.30000	exclusion0605
570.75000	exclusion0606
571.27000	exclusion0607
571.77000	exclusion0608
571.81000	exclusion0609
572.30000	exclusion0610
572.31000	exclusion0611
572.67000	exclusion0612
573.29000	exclusion0613
573.30000	exclusion0614
573.81000	exclusion0615
573.96000	exclusion0616
574.28000	exclusion0617
574.31000	exclusion0618
574.63000	exclusion0619
574.79000	exclusion0620
575.28000	exclusion0621
575.80000	exclusion0622

575.99000	exclusion0623
576.21000	exclusion0624
576.25000	exclusion0625
576.71000	exclusion0626
577.67000	exclusion0627
577.83000	exclusion0628
577.98000	exclusion0629
578.25000	exclusion0630
578.26000	exclusion0631
578.65000	exclusion0632
578.97000	exclusion0633
579.34000	exclusion0634
579.78000	exclusion0635
580.03000	exclusion0636
580.30000	exclusion0637
580.31000	exclusion0638
581.00000	exclusion0639
581.29000	exclusion0640
581.83000	exclusion0641
581.96000	exclusion0642
582.10000	exclusion0643
582.25000	exclusion0644
582.31000	exclusion0645
582.74000	exclusion0646
583.03000	exclusion0647
583.79000	exclusion0648
583.95000	exclusion0649
584.27000	exclusion0650
584.65000	exclusion0651
584.76000	exclusion0652
585.26000	exclusion1023
585.28000	exclusion1024
585.28000	exclusion1025
585.65000	exclusion1026
586.24000	exclusion0653
586.31000	exclusion0654
586.75000	exclusion0655
587.31000	exclusion0656
587.33000	exclusion0657
588.00000	exclusion0658
588.28000	exclusion0659
588.30000	exclusion0660
588.80000	exclusion0661
589.30000	exclusion0662
590.25000	exclusion0663
590.29000	exclusion0664
590.80000	exclusion0665
590.84000	exclusion0666
590.97000	exclusion0667
591.34000	exclusion0668
591.65000	exclusion0669

591.79000	exclusion0670
592.31000	exclusion0671
592.85000	exclusion0672
592.98000	exclusion0673
593.16000	exclusion0674
593.59000	exclusion0675
593.61000	exclusion0676
593.81000	exclusion0677
594.58000	exclusion0678
594.76000	exclusion0679
594.80000	exclusion0680
595.31000	exclusion0681
595.63000	exclusion0682
595.76000	exclusion0683
595.92000	exclusion0684
596.77000	exclusion0685
597.28000	exclusion0686
597.29000	exclusion0687
597.78000	exclusion0688
598.31000	exclusion0689
598.36000	exclusion0690
598.81000	exclusion0691
599.35000	exclusion0692
599.67000	exclusion0693
599.78000	exclusion0694
600.00000	exclusion0695
600.26000	exclusion0696
600.26000	exclusion0697
600.78000	exclusion1027
601.30000	exclusion0698
601.31000	exclusion0699
601.81000	exclusion0700
602.26000	exclusion0701
602.30000	exclusion0702
602.31000	exclusion0703
602.84000	exclusion1028
603.28000	exclusion1029
603.80000	exclusion0704
603.98000	exclusion0705
604.28000	exclusion0706
604.32000	exclusion0707
604.32000	exclusion0708
604.79000	exclusion1030
605.78000	exclusion0709
606.00000	exclusion0710
606.28000	exclusion0711
606.28000	exclusion0712
606.29000	exclusion0713
606.98000	exclusion0714
607.28000	exclusion1031
607.30000	exclusion1032

607.30000	exclusion1033
607.81000	exclusion0715
608.32000	exclusion1034
608.85000	exclusion0716
608.95000	exclusion0717
609.31000	exclusion0718
609.85000	exclusion1035
610.32000	exclusion0719
611.05000	exclusion0720
611.27000	exclusion0721
611.29000	exclusion0722
611.77000	exclusion0723
612.32000	exclusion1036
612.33000	exclusion0724
612.33000	exclusion1037
612.35000	exclusion1038
613.32000	exclusion1039
614.09000	exclusion0725
614.14000	exclusion0726
614.29000	exclusion0727
614.30000	exclusion0728
614.77000	exclusion0729
615.30000	exclusion0730
615.82000	exclusion0731
616.28000	exclusion0732
616.32000	exclusion0733
616.83000	exclusion0734
617.31000	exclusion0735
617.34000	exclusion0736
617.62000	exclusion0737
617.80000	exclusion0738
617.93000	exclusion0739
619.26000	exclusion0740
619.30000	exclusion0741
619.63000	exclusion0742
620.59000	exclusion0743
620.81000	exclusion0744
621.29000	exclusion0745
621.29000	exclusion0746
621.66000	exclusion0747
621.71000	exclusion0748
621.98000	exclusion0749
623.32000	exclusion0750
623.32000	exclusion0751
623.82000	exclusion0752
624.01000	exclusion0753
624.83000	exclusion0754
625.01000	exclusion0755
625.30000	exclusion0756
625.33000	exclusion0757
625.87000	exclusion0758

635.29000	exclusion1040
635.32000	exclusion1041
635.86000	exclusion1042
642.35000	exclusion1043
646.80000	exclusion1044
647.03000	exclusion1045
652.30000	exclusion1046
652.32000	exclusion1047
653.32000	exclusion1048
653.35000	exclusion1049
671.30000	exclusion1050
671.53000	exclusion1051
671.83000	exclusion1052
681.83000	exclusion1053
681.84000	exclusion1054
682.85000	exclusion1055
688.27000	exclusion1056
694.81000	exclusion1057
711.35000	exclusion1058
711.69000	exclusion1059
711.89000	exclusion1060
712.38000	exclusion1061
712.39000	exclusion1062
718.85000	exclusion1063
718.85000	exclusion1064
723.93000	exclusion1065
724.34000	exclusion1066
724.37000	exclusion1067
724.68000	exclusion1068
725.37000	exclusion1069
745.36000	exclusion1070
745.90000	exclusion1071
765.34000	exclusion1072
765.36000	exclusion1073
766.36000	exclusion1074
767.16000	exclusion1075
767.37000	exclusion1076
767.37000	exclusion1077
767.86000	exclusion1078
772.39000	exclusion1079
772.39000	exclusion1080
772.85000	exclusion1081
773.38000	exclusion1082
782.79000	exclusion1083
803.37000	exclusion1084
812.36000	exclusion1085
812.86000	exclusion1086
814.36000	exclusion1087
814.41000	exclusion1088
814.74000	exclusion1089
816.44000	exclusion1090

835.72000	exclusion1091
836.06000	exclusion1092
836.40000	exclusion1093
842.11000	exclusion1094
842.14000	exclusion1095
842.38000	exclusion1096
842.39000	exclusion1097
844.44000	exclusion1098
844.58000	exclusion1099
844.89000	exclusion1100
845.37000	exclusion1101
845.41000	exclusion1102
848.91000	exclusion1103
849.37000	exclusion1104
863.92000	exclusion1105
864.42000	exclusion1106
865.40000	exclusion1107
866.41000	exclusion1108
867.43000	exclusion1109
867.44000	exclusion0759
881.94000	exclusion1110
903.92000	exclusion0760
904.43000	exclusion1111
904.44000	exclusion1112
904.45000	exclusion0761
906.91000	exclusion0762
908.93000	exclusion1113
909.43000	exclusion1114
910.44000	exclusion1115
910.44000	exclusion1116
912.91000	exclusion1117
912.99000	exclusion1118
913.45000	exclusion1119
913.93000	exclusion1120
927.48000	exclusion1121
932.94000	exclusion1122
933.14000	exclusion1123
933.47000	exclusion1124
937.12000	exclusion1125
937.46000	exclusion1126
940.44000	exclusion1127
940.48000	exclusion1128
941.09000	exclusion1129
941.96000	exclusion0763
942.48000	exclusion1130
960.41000	exclusion1131
961.98000	exclusion1132
968.97000	exclusion1133
989.52000	exclusion1134
992.50000	exclusion1135
993.02000	exclusion1136

993.50000	exclusion1137
994.47000	exclusion1138
994.98000	exclusion1139
995.46000	exclusion1140
996.98000	exclusion1141
1005.98000	exclusion1142
1013.49000	exclusion1143
1013.79000	exclusion1144
1014.47000	exclusion1145
1018.41000	exclusion1146
1024.51000	exclusion1530
1025.42000	exclusion1531
1025.86000	exclusion1532
1026.79000	exclusion1533
1027.46000	exclusion1534
1028.02000	exclusion1535
1028.98000	exclusion1536
1029.51000	exclusion1147
1030.46000	exclusion1537
1030.53000	exclusion1148
1031.47000	exclusion1538
1032.46000	exclusion1539
1032.86000	exclusion1540
1033.15000	exclusion1541
1033.52000	exclusion1542
1034.02000	exclusion1543
1034.80000	exclusion1149
1035.54000	exclusion1150
1035.55000	exclusion1544
1036.20000	exclusion1545
1036.53000	exclusion1546
1036.77000	exclusion1547
1037.45000	exclusion1548
1038.03000	exclusion1151
1038.26000	exclusion1152
1038.46000	exclusion1153
1038.98000	exclusion1549
1039.29000	exclusion1550
1039.50000	exclusion1551
1040.04000	exclusion1552
1040.51000	exclusion1553
1041.47000	exclusion1554
1041.77000	exclusion1555
1042.02000	exclusion1556
1042.52000	exclusion1557
1043.14000	exclusion1558
1044.17000	exclusion1559
1044.52000	exclusion1560
1044.97000	exclusion1561
1045.46000	exclusion1562
1046.50000	exclusion0764

1046.50000	exclusion1154
1046.50000	exclusion1563
1047.50000	exclusion1155
1047.82000	exclusion1564
1048.05000	exclusion1565
1048.84000	exclusion1566
1049.47000	exclusion1567
1049.87000	exclusion1568
1050.51000	exclusion1569
1050.77000	exclusion1570
1051.47000	exclusion1571
1052.00000	exclusion1156
1052.53000	exclusion1572
1053.06000	exclusion1573
1053.53000	exclusion1574
1053.84000	exclusion1575
1054.50000	exclusion1576
1055.04000	exclusion1577
1055.48000	exclusion1578
1056.04000	exclusion1579
1056.52000	exclusion1580
1057.55000	exclusion0765
1057.55000	exclusion1157
1057.56000	exclusion1581
1057.84000	exclusion1582
1058.07000	exclusion1583
1058.48000	exclusion1584
1059.27000	exclusion1585
1059.48000	exclusion1586
1060.03000	exclusion1587
1060.53000	exclusion1588
1061.02000	exclusion1589
1061.83000	exclusion1590
1062.24000	exclusion1591
1062.54000	exclusion1592
1063.01000	exclusion1593
1063.48000	exclusion1594
1064.48000	exclusion1595
1065.47000	exclusion1596
1066.11000	exclusion1158
1066.54000	exclusion1159
1067.04000	exclusion1597
1067.49000	exclusion1598
1067.53000	exclusion1160
1068.04000	exclusion1599
1068.47000	exclusion1600
1069.03000	exclusion1161
1069.45000	exclusion1162
1070.06000	exclusion1601
1070.52000	exclusion1602
1071.15000	exclusion1603

1071.54000	exclusion1604
1072.07000	exclusion1163
1072.54000	exclusion1164
1072.84000	exclusion1165
1072.87000	exclusion1605
1073.53000	exclusion1606
1074.04000	exclusion1607
1074.55000	exclusion1166
1074.85000	exclusion1167
1075.13000	exclusion1168
1075.52000	exclusion1169
1075.53000	exclusion1608
1076.06000	exclusion1609
1077.04000	exclusion1610
1077.54000	exclusion1611
1078.07000	exclusion0766
1078.56000	exclusion0767
1078.99000	exclusion1612
1079.51000	exclusion1613
1080.50000	exclusion1614
1080.85000	exclusion1615
1081.53000	exclusion1616
1082.19000	exclusion1617
1082.53000	exclusion1618
1083.55000	exclusion1170
1083.58000	exclusion1619
1084.06000	exclusion1620
1084.55000	exclusion1621
1085.04000	exclusion1622
1085.53000	exclusion1623
1086.05000	exclusion1624
1086.49000	exclusion1625
1086.99000	exclusion1171
1087.55000	exclusion1172
1087.55000	exclusion1626
1087.88000	exclusion1627
1088.24000	exclusion1628
1088.49000	exclusion1629
1089.00000	exclusion1173
1089.53000	exclusion1174
1090.05000	exclusion1175
1090.06000	exclusion1630
1090.53000	exclusion1631
1091.06000	exclusion1632
1091.89000	exclusion1633
1092.50000	exclusion1176
1093.03000	exclusion1177
1093.53000	exclusion1634
1093.87000	exclusion1635
1094.55000	exclusion1636
1095.50000	exclusion1637

1095.86000	exclusion1638
1096.12000	exclusion1639
1097.16000	exclusion1178
1097.29000	exclusion1640
1097.54000	exclusion1641
1098.20000	exclusion1642
1099.29000	exclusion1179
1099.53000	exclusion1643
1099.54000	exclusion0768
1100.03000	exclusion1180
1100.56000	exclusion1181
1100.81000	exclusion1182
1101.09000	exclusion1183
1101.09000	exclusion1644
1102.22000	exclusion1645
1102.51000	exclusion1646
1102.88000	exclusion1647
1103.99000	exclusion1648
1104.22000	exclusion1649
1104.58000	exclusion1650
1105.54000	exclusion1651
1105.77000	exclusion1652
1106.53000	exclusion1653
1107.54000	exclusion1654
1108.05000	exclusion1184
1108.58000	exclusion1655
1109.52000	exclusion1656
1109.88000	exclusion1657
1110.23000	exclusion1658
1110.91000	exclusion1185
1111.54000	exclusion1186
1111.56000	exclusion1659
1111.87000	exclusion1660
1112.08000	exclusion1661
1112.80000	exclusion1662
1113.00000	exclusion1663
1113.52000	exclusion1664
1114.04000	exclusion1187
1114.85000	exclusion1188
1114.94000	exclusion1665
1115.55000	exclusion1666
1116.08000	exclusion1667
1116.88000	exclusion1668
1117.52000	exclusion1669
1118.09000	exclusion1670
1118.58000	exclusion1671
1118.86000	exclusion1672
1119.89000	exclusion1673
1120.52000	exclusion1674
1121.05000	exclusion1675
1121.56000	exclusion1189

1121.88000	exclusion1190
1122.19000	exclusion1676
1122.51000	exclusion1677
1123.21000	exclusion1678
1123.56000	exclusion1679
1124.57000	exclusion1680
1125.02000	exclusion1681
1125.54000	exclusion1682
1126.04000	exclusion1683
1126.54000	exclusion1191
1127.00000	exclusion1192
1127.21000	exclusion1193
1127.55000	exclusion1684
1127.87000	exclusion1685
1128.09000	exclusion1686
1128.53000	exclusion1687
1129.58000	exclusion0769
1129.58000	exclusion1194
1130.12000	exclusion1195
1130.21000	exclusion1688
1130.54000	exclusion1196
1130.58000	exclusion1689
1131.03000	exclusion1690
1131.57000	exclusion1691
1132.20000	exclusion1692
1132.87000	exclusion1693
1133.08000	exclusion1694
1133.62000	exclusion1695
1133.89000	exclusion1696
1134.54000	exclusion1697
1135.11000	exclusion1698
1136.04000	exclusion1699
1136.53000	exclusion1700
1137.44000	exclusion1701
1138.55000	exclusion0770
1138.55000	exclusion1197
1138.55000	exclusion1702
1139.18000	exclusion1703
1139.54000	exclusion1704
1140.20000	exclusion1198
1140.58000	exclusion1199
1140.83000	exclusion1200
1141.52000	exclusion1705
1141.90000	exclusion1706
1142.51000	exclusion1707
1143.05000	exclusion1708
1143.56000	exclusion1709
1144.09000	exclusion1201
1144.54000	exclusion1202
1145.04000	exclusion1710
1145.51000	exclusion1203

1145.53000	exclusion1711
1146.56000	exclusion1712
1147.00000	exclusion1713
1147.53000	exclusion1714
1148.07000	exclusion1715
1148.56000	exclusion1716
1149.06000	exclusion1717
1149.56000	exclusion1718
1150.56000	exclusion1204
1150.56000	exclusion1719
1151.05000	exclusion1720
1151.60000	exclusion1721
1152.12000	exclusion1722
1152.55000	exclusion1723
1153.54000	exclusion1724
1154.20000	exclusion1725
1154.55000	exclusion1726
1155.08000	exclusion1727
1156.07000	exclusion1728
1156.52000	exclusion1729
1157.56000	exclusion1730
1158.23000	exclusion1731
1158.51000	exclusion1732
1159.44000	exclusion1733
1159.64000	exclusion1734
1159.91000	exclusion1735
1160.55000	exclusion0771
1161.05000	exclusion0772
1161.55000	exclusion0773
1161.55000	exclusion1205
1161.57000	exclusion1736
1162.04000	exclusion1737
1162.58000	exclusion1738
1163.03000	exclusion1739
1163.58000	exclusion1206
1163.89000	exclusion1207
1164.56000	exclusion1740
1165.57000	exclusion0774
1165.57000	exclusion1741
1166.27000	exclusion1208
1166.53000	exclusion1209
1166.90000	exclusion1210
1167.20000	exclusion1742
1167.21000	exclusion1211
1167.52000	exclusion1743
1168.20000	exclusion1744
1168.55000	exclusion1745
1169.07000	exclusion0775
1169.58000	exclusion1746
1170.08000	exclusion1212
1170.56000	exclusion1213

1171.09000	exclusion1747
1171.61000	exclusion1748
1172.54000	exclusion1214
1173.26000	exclusion1215
1173.27000	exclusion1749
1173.56000	exclusion1750
1174.54000	exclusion1751
1175.48000	exclusion1752
1176.00000	exclusion1216
1176.58000	exclusion1217
1176.59000	exclusion1753
1177.58000	exclusion1754
1178.09000	exclusion1755
1178.50000	exclusion1756
1179.08000	exclusion1218
1179.58000	exclusion1757
1180.14000	exclusion1758
1180.94000	exclusion1219
1181.56000	exclusion1759
1181.91000	exclusion1220
1182.54000	exclusion1760
1182.93000	exclusion1761
1183.53000	exclusion1221
1184.10000	exclusion1762
1184.55000	exclusion1763
1185.54000	exclusion1764
1186.47000	exclusion1222
1187.05000	exclusion1223
1187.10000	exclusion1765
1187.58000	exclusion1766
1188.57000	exclusion1767
1189.54000	exclusion1768
1189.91000	exclusion1769
1190.58000	exclusion1224
1191.08000	exclusion1225
1192.06000	exclusion1226
1192.62000	exclusion1227
1192.62000	exclusion1770
1192.89000	exclusion1771
1193.59000	exclusion1772
1194.23000	exclusion1228
1195.03000	exclusion1229
1195.03000	exclusion1773
1195.25000	exclusion1774
1196.25000	exclusion1775
1196.54000	exclusion1776
1197.05000	exclusion0776
1197.55000	exclusion1230
1197.55000	exclusion1777
1198.55000	exclusion1778
1199.06000	exclusion1231

1199.57000	exclusion0777
1200.23000	exclusion0778
1200.23000	exclusion1779
1200.56000	exclusion0779
1200.56000	exclusion1780
1200.57000	exclusion1232
1200.84000	exclusion1781
1201.11000	exclusion1782
1202.05000	exclusion1233
1202.57000	exclusion1234
1202.57000	exclusion1783
1203.50000	exclusion1784
1204.53000	exclusion1235
1204.54000	exclusion1785
1204.91000	exclusion1786
1205.63000	exclusion1787
1206.03000	exclusion1788
1206.59000	exclusion1789
1207.23000	exclusion1236
1207.88000	exclusion1790
1208.87000	exclusion1791
1209.91000	exclusion1792
1210.64000	exclusion1793
1211.01000	exclusion1794
1211.60000	exclusion1795
1212.25000	exclusion1796
1212.76000	exclusion1237
1213.52000	exclusion1238
1213.61000	exclusion1797
1214.58000	exclusion1798
1215.10000	exclusion0780
1215.65000	exclusion1799
1216.12000	exclusion1800
1217.14000	exclusion1239
1217.61000	exclusion1240
1217.86000	exclusion1801
1218.54000	exclusion1802
1219.58000	exclusion1803
1220.22000	exclusion1241
1220.58000	exclusion1242
1221.12000	exclusion1804
1222.07000	exclusion1243
1223.05000	exclusion1244
1223.05000	exclusion1805
1223.57000	exclusion1806
1224.08000	exclusion1807
1224.59000	exclusion1808
1225.09000	exclusion1245
1225.60000	exclusion1246
1225.92000	exclusion1809
1226.62000	exclusion1810

1227.13000	exclusion1811
1227.66000	exclusion0781
1228.28000	exclusion1812
1228.82000	exclusion1813
1229.35000	exclusion1247
1229.56000	exclusion1248
1230.06000	exclusion1814
1230.66000	exclusion1249
1231.11000	exclusion1250
1232.05000	exclusion1815
1232.59000	exclusion1251
1233.10000	exclusion1252
1233.30000	exclusion1816
1234.12000	exclusion1817
1235.07000	exclusion1818
1235.58000	exclusion1819
1236.08000	exclusion0782
1237.06000	exclusion1253
1237.12000	exclusion1820
1237.95000	exclusion1254
1240.06000	exclusion0783
1240.06000	exclusion1255
1241.05000	exclusion1821
1241.64000	exclusion1822
1241.87000	exclusion1823
1242.58000	exclusion0784
1243.30000	exclusion1256
1244.09000	exclusion1257
1244.59000	exclusion0785
1245.11000	exclusion1824
1245.92000	exclusion1825
1246.97000	exclusion1826
1247.36000	exclusion1827
1247.59000	exclusion1828
1248.57000	exclusion1829
1249.04000	exclusion1830
1249.65000	exclusion1258
1250.60000	exclusion1259
1250.60000	exclusion1831
1252.18000	exclusion0786
1252.19000	exclusion1832
1252.20000	exclusion1260
1252.59000	exclusion1833
1253.07000	exclusion1834
1253.59000	exclusion1835
1254.63000	exclusion1836
1255.13000	exclusion0787
1255.14000	exclusion1261
1256.59000	exclusion1262
1256.79000	exclusion1837
1257.09000	exclusion1263

1257.09000	exclusion1838
1257.64000	exclusion1264
1258.57000	exclusion1839
1259.21000	exclusion1840
1260.13000	exclusion1265
1260.63000	exclusion1841
1260.64000	exclusion1266
1261.60000	exclusion1267
1262.37000	exclusion0788
1262.95000	exclusion0789
1263.27000	exclusion1268
1264.07000	exclusion1269
1264.16000	exclusion1842
1265.08000	exclusion1843
1265.59000	exclusion1844
1265.97000	exclusion1845
1266.61000	exclusion1270
1267.25000	exclusion1271
1268.19000	exclusion1272
1268.81000	exclusion1273
1269.15000	exclusion1274
1269.66000	exclusion0790
1270.26000	exclusion1275
1270.27000	exclusion1846
1271.18000	exclusion1847
1272.11000	exclusion1276
1272.67000	exclusion1277
1272.68000	exclusion1848
1274.07000	exclusion0791
1274.07000	exclusion1278
1274.65000	exclusion0792
1275.16000	exclusion1279
1275.62000	exclusion1280
1275.63000	exclusion0793
1275.65000	exclusion1849
1276.64000	exclusion1850
1277.14000	exclusion1281
1277.67000	exclusion1282
1278.46000	exclusion1283
1279.01000	exclusion1284
1279.02000	exclusion1851
1279.61000	exclusion1852
1280.64000	exclusion0794
1281.38000	exclusion0795
1283.14000	exclusion0796
1283.66000	exclusion1285
1283.66000	exclusion1853
1284.64000	exclusion1854
1285.14000	exclusion0797
1286.65000	exclusion0798
1286.65000	exclusion1286

1286.66000	exclusion1855
1287.08000	exclusion1856
1287.66000	exclusion1287
1288.63000	exclusion1288
1288.64000	exclusion1857
1289.45000	exclusion1858
1291.08000	exclusion1859
1292.39000	exclusion1860
1293.18000	exclusion1861
1294.10000	exclusion1289
1294.61000	exclusion1862
1295.33000	exclusion1863
1296.63000	exclusion0799
1296.64000	exclusion1290
1298.01000	exclusion0800
1298.08000	exclusion1864
1298.58000	exclusion1291
1299.13000	exclusion1292
1300.07000	exclusion1865
1301.06000	exclusion1866
1301.57000	exclusion1293
1302.09000	exclusion1867
1302.10000	exclusion1294
1302.70000	exclusion0801
1303.06000	exclusion0802
1304.08000	exclusion1295
1304.09000	exclusion1868
1304.65000	exclusion1296
1305.19000	exclusion0803
1305.69000	exclusion1297
1306.06000	exclusion1298
1306.58000	exclusion0804
1307.12000	exclusion1869
1308.00000	exclusion1299
1309.06000	exclusion0805
1309.07000	exclusion1300
1310.67000	exclusion1301
1311.63000	exclusion1302
1312.61000	exclusion1870
1312.67000	exclusion1303
1313.62000	exclusion1304
1313.62000	exclusion1871
1314.61000	exclusion1305
1315.13000	exclusion1306
1315.59000	exclusion1872
1315.69000	exclusion1307
1316.64000	exclusion1308
1318.03000	exclusion1309
1318.61000	exclusion1310
1319.61000	exclusion0806
1319.74000	exclusion1873

1320.09000	exclusion1311
1320.66000	exclusion1874
1321.12000	exclusion1312
1321.12000	exclusion1875
1321.67000	exclusion0807
1322.10000	exclusion0808
1322.61000	exclusion1313
1323.60000	exclusion1314
1324.18000	exclusion1876
1324.61000	exclusion1877
1325.11000	exclusion1315
1326.10000	exclusion1316
1328.19000	exclusion0809
1328.19000	exclusion1317
1328.25000	exclusion1878
1330.61000	exclusion1318
1331.19000	exclusion1319
1331.20000	exclusion1879
1331.82000	exclusion1320
1332.12000	exclusion1321
1332.19000	exclusion1880
1332.65000	exclusion1322
1333.65000	exclusion1323
1333.65000	exclusion1881
1334.06000	exclusion1324
1335.19000	exclusion0810
1335.19000	exclusion1325
1335.67000	exclusion1326
1335.68000	exclusion1882
1336.17000	exclusion1327
1337.17000	exclusion0811
1337.18000	exclusion1883
1337.19000	exclusion1328
1339.66000	exclusion1329
1339.68000	exclusion1884
1340.19000	exclusion0812
1341.16000	exclusion1330
1341.71000	exclusion1885
1342.13000	exclusion1331
1342.13000	exclusion1886
1343.18000	exclusion0813
1343.68000	exclusion1332
1344.18000	exclusion1887
1344.63000	exclusion1333
1345.24000	exclusion0814
1345.68000	exclusion0815
1346.20000	exclusion1888
1346.61000	exclusion1889
1346.62000	exclusion1334
1347.12000	exclusion1335
1347.63000	exclusion1890

1348.36000	exclusion1336
1348.36000	exclusion1891
1348.67000	exclusion1337
1349.10000	exclusion1338
1349.63000	exclusion1339
1350.14000	exclusion0816
1350.34000	exclusion0817
1351.64000	exclusion1340
1352.65000	exclusion0818
1352.66000	exclusion1341
1352.66000	exclusion1892
1353.92000	exclusion0819
1354.16000	exclusion1342
1354.19000	exclusion1893
1354.68000	exclusion1343
1355.21000	exclusion1344
1355.61000	exclusion1345
1355.65000	exclusion1894
1356.17000	exclusion1346
1356.66000	exclusion1347
1356.72000	exclusion1895
1357.19000	exclusion1896
1357.61000	exclusion1348
1359.64000	exclusion0820
1359.64000	exclusion1349
1360.23000	exclusion1350
1361.19000	exclusion1897
1361.70000	exclusion1351
1362.20000	exclusion0821
1363.64000	exclusion0822
1363.66000	exclusion1352
1364.21000	exclusion1353
1365.18000	exclusion1898
1365.19000	exclusion1354
1365.69000	exclusion1355
1366.64000	exclusion1356
1367.67000	exclusion0823
1367.70000	exclusion1357
1368.17000	exclusion1358
1368.17000	exclusion1899
1368.69000	exclusion1900
1368.74000	exclusion1359
1370.19000	exclusion0824
1370.64000	exclusion0825
1371.20000	exclusion1360
1372.19000	exclusion1361
1373.15000	exclusion1362
1374.17000	exclusion0826
1374.18000	exclusion1363
1374.38000	exclusion1901
1374.68000	exclusion1902

1375.70000	exclusion1364
1376.17000	exclusion1365
1376.21000	exclusion0827
1376.21000	exclusion1903
1376.68000	exclusion1366
1377.18000	exclusion1904
1377.19000	exclusion1367
1380.13000	exclusion1368
1380.13000	exclusion1905
1380.14000	exclusion0828
1381.15000	exclusion1369
1381.15000	exclusion1906
1382.04000	exclusion1370
1382.71000	exclusion1907
1382.72000	exclusion1371
1383.62000	exclusion1372
1384.69000	exclusion0829
1384.69000	exclusion1373
1384.69000	exclusion1908
1385.11000	exclusion1909
1385.12000	exclusion1374
1385.69000	exclusion0830
1386.71000	exclusion0831
1387.20000	exclusion1375
1387.20000	exclusion1910
1388.70000	exclusion1376
1389.68000	exclusion1377
1390.19000	exclusion1911
1392.67000	exclusion1378
1392.69000	exclusion1912
1394.63000	exclusion0832
1395.15000	exclusion1379
1395.16000	exclusion1913
1395.68000	exclusion1380
1395.68000	exclusion1914
1396.19000	exclusion0833
1396.70000	exclusion1381
1397.11000	exclusion1915
1397.12000	exclusion1382
1397.73000	exclusion1383
1398.71000	exclusion1916
1399.21000	exclusion1384
1399.69000	exclusion1917
1399.70000	exclusion1385
1400.20000	exclusion0834
1402.15000	exclusion0835
1402.16000	exclusion1386
1402.16000	exclusion1918
1402.79000	exclusion1387
1403.67000	exclusion1388
1404.17000	exclusion1919

1404.44000	exclusion1389
1405.23000	exclusion1390
1405.23000	exclusion1920
1406.31000	exclusion1921
1406.33000	exclusion1391
1406.66000	exclusion1392
1407.11000	exclusion1393
1407.12000	exclusion1922
1408.02000	exclusion0836
1408.36000	exclusion1394
1412.69000	exclusion0837
1413.19000	exclusion1923
1413.20000	exclusion0838
1413.22000	exclusion1395
1413.64000	exclusion1396
1413.71000	exclusion1924
1415.02000	exclusion1397
1415.68000	exclusion1398
1415.69000	exclusion1925
1417.65000	exclusion0839
1417.66000	exclusion1399
1417.68000	exclusion1926
1418.03000	exclusion1400
1419.37000	exclusion1401
1419.38000	exclusion1927
1420.34000	exclusion1402
1420.93000	exclusion1928
1421.12000	exclusion1403
1422.19000	exclusion0840
1422.22000	exclusion1929
1422.70000	exclusion1404
1423.71000	exclusion1405
1424.20000	exclusion1406
1424.71000	exclusion0841
1425.33000	exclusion1407
1425.68000	exclusion1408
1427.97000	exclusion1409
1428.67000	exclusion1410
1429.32000	exclusion1930
1430.20000	exclusion1411
1431.14000	exclusion1931
1432.24000	exclusion0842
1432.34000	exclusion1412
1432.69000	exclusion1413
1433.21000	exclusion1414
1433.71000	exclusion1932
1434.25000	exclusion0843
1435.72000	exclusion1415
1436.21000	exclusion1416
1437.21000	exclusion0844
1437.23000	exclusion1417

1440.24000	exclusion0845
1440.25000	exclusion1418
1440.73000	exclusion1933
1440.79000	exclusion1419
1441.23000	exclusion1420
1442.71000	exclusion1421
1442.72000	exclusion1934
1443.66000	exclusion1422
1444.21000	exclusion1423
1445.70000	exclusion0846
1445.74000	exclusion1424
1445.74000	exclusion1935
1446.19000	exclusion1425
1446.71000	exclusion0847
1448.16000	exclusion0848
1448.17000	exclusion1426
1449.06000	exclusion1427
1450.25000	exclusion1936
1450.28000	exclusion0849
1450.72000	exclusion1428
1451.24000	exclusion1937
1453.68000	exclusion0850
1453.70000	exclusion1429
1454.23000	exclusion1938
1456.72000	exclusion1430
1456.74000	exclusion1939
1457.22000	exclusion0851
1457.24000	exclusion1940
1458.26000	exclusion1431
1458.26000	exclusion1941
1458.75000	exclusion0852
1459.21000	exclusion1432
1460.09000	exclusion1433
1460.42000	exclusion1942
1460.75000	exclusion1943
1461.23000	exclusion1434
1461.70000	exclusion1435
1461.71000	exclusion1944
1462.73000	exclusion0853
1463.72000	exclusion1436
1464.70000	exclusion0854
1465.43000	exclusion0855
1465.73000	exclusion1945
1465.74000	exclusion1437
1467.71000	exclusion0856
1467.73000	exclusion1946
1468.23000	exclusion1438
1469.73000	exclusion0857
1471.30000	exclusion0858
1471.31000	exclusion1947
1471.73000	exclusion0859

1471.73000	exclusion1439
1473.73000	exclusion1440
1473.74000	exclusion1948
1474.23000	exclusion1441
1475.23000	exclusion0860
1478.29000	exclusion0861
1479.22000	exclusion0862
1479.23000	exclusion1442
1479.23000	exclusion1949
1479.71000	exclusion1443
1479.72000	exclusion1950
1480.24000	exclusion1444
1481.77000	exclusion1445
1482.27000	exclusion0863
1482.27000	exclusion1951
1485.24000	exclusion0864
1485.74000	exclusion1446
1486.23000	exclusion1952
1487.33000	exclusion1447
1489.72000	exclusion0865
1489.72000	exclusion1448
1489.73000	exclusion1953
1490.72000	exclusion1954
1490.73000	exclusion1449
1492.77000	exclusion1450
1492.77000	exclusion1955
1493.26000	exclusion0866
1493.77000	exclusion1451
1497.22000	exclusion0867
1497.22000	exclusion1956
1497.73000	exclusion1452
1498.24000	exclusion1957
1498.73000	exclusion1453
1504.74000	exclusion0868
1504.74000	exclusion1454
1507.24000	exclusion0869
1507.24000	exclusion1455
1507.24000	exclusion1958
1507.74000	exclusion1456
1507.75000	exclusion0870
1508.24000	exclusion0871
1509.23000	exclusion0872
1509.24000	exclusion1457
1509.75000	exclusion0873
1510.28000	exclusion1959
1510.29000	exclusion1458
1510.78000	exclusion0874
1512.19000	exclusion1459
1512.69000	exclusion1960
1512.70000	exclusion1460
1514.74000	exclusion1961

1515.24000	exclusion1461
1518.25000	exclusion1462
1518.73000	exclusion0875
1519.22000	exclusion1463
1520.79000	exclusion1464
1522.20000	exclusion1465
1524.25000	exclusion1466
1524.25000	exclusion1962
1524.75000	exclusion0876
1528.30000	exclusion1467
1528.32000	exclusion1963
1530.83000	exclusion0877
1532.25000	exclusion0878
1532.74000	exclusion1468
1532.76000	exclusion1964
1535.73000	exclusion0879
1535.75000	exclusion1469
1537.22000	exclusion1470
1537.29000	exclusion1965
1537.30000	exclusion0880
1537.73000	exclusion1471
1538.72000	exclusion0881
1543.22000	exclusion0882
1543.74000	exclusion1472
1544.29000	exclusion1966
1546.30000	exclusion0883
1552.75000	exclusion0884
1553.26000	exclusion1473
1553.78000	exclusion1474
1553.80000	exclusion1967
1555.25000	exclusion1475
1559.76000	exclusion1476
1561.27000	exclusion1477
1561.75000	exclusion1968
1561.76000	exclusion0885
1563.30000	exclusion1969
1564.79000	exclusion1478
1565.79000	exclusion1479
1567.76000	exclusion0886
1568.27000	exclusion1480
1568.27000	exclusion1970
1569.27000	exclusion1481
1570.29000	exclusion0887
1570.29000	exclusion1482
1572.25000	exclusion0888
1572.26000	exclusion1483
1572.26000	exclusion1971
1574.30000	exclusion1972
1574.79000	exclusion0889
1575.32000	exclusion1973
1575.80000	exclusion1484

1576.43000	exclusion1485
1578.75000	exclusion0890
1579.25000	exclusion1486
1579.26000	exclusion1974
1580.48000	exclusion1487
1580.51000	exclusion1975
1580.81000	exclusion0891
1581.78000	exclusion0892
1583.23000	exclusion1488
1584.31000	exclusion1489
1584.32000	exclusion1976
1584.81000	exclusion0893
1586.30000	exclusion0894
1589.77000	exclusion1490
1589.78000	exclusion1977
1590.28000	exclusion0895
1591.81000	exclusion0896
1592.33000	exclusion1491
1596.28000	exclusion1492
1598.84000	exclusion0897
1599.72000	exclusion1493
1635.31000	exclusion1978
1635.32000	exclusion0898
1635.33000	exclusion1494
1635.90000	exclusion0899
1643.27000	exclusion0900
1643.75000	exclusion1495
1646.30000	exclusion0901
1648.81000	exclusion1496
1649.31000	exclusion0902
1651.37000	exclusion0903
1654.77000	exclusion1497
1655.83000	exclusion0904
1656.31000	exclusion1498
1656.32000	exclusion1979
1662.32000	exclusion1499
1662.32000	exclusion1980
1663.84000	exclusion1500
1664.33000	exclusion1501
1667.28000	exclusion1981
1667.32000	exclusion0905
1667.33000	exclusion1502
1668.30000	exclusion1503
1669.87000	exclusion0906
1670.37000	exclusion0907
1671.31000	exclusion1504
1671.84000	exclusion1982
1673.31000	exclusion0908
1673.32000	exclusion1983
1673.35000	exclusion1505
1675.30000	exclusion0909

1675.31000	exclusion1984
1675.33000	exclusion1506
1677.31000	exclusion1507
1677.81000	exclusion1508
1678.32000	exclusion1985
1679.40000	exclusion1509
1679.80000	exclusion1986
1679.85000	exclusion0910
1682.33000	exclusion0911
1682.80000	exclusion1510
1683.30000	exclusion1511
1683.82000	exclusion0912
1684.32000	exclusion1512
1684.32000	exclusion1987
1684.85000	exclusion1988
1686.39000	exclusion1513
1687.38000	exclusion1989
1687.81000	exclusion1514
1688.81000	exclusion1515
1690.34000	exclusion0913
1690.39000	exclusion1516
1690.84000	exclusion1990
1698.33000	exclusion1517
1698.34000	exclusion1991
1699.86000	exclusion0914
1700.37000	exclusion1518
1701.33000	exclusion1992
1701.81000	exclusion1519
1703.85000	exclusion0915
1707.86000	exclusion0916
1707.86000	exclusion1520
1708.36000	exclusion1993
1722.42000	exclusion0917
1760.89000	exclusion0918
1760.89000	exclusion1521
1760.89000	exclusion1994
1764.34000	exclusion1522
1773.39000	exclusion0919
1773.89000	exclusion1523
1773.89000	exclusion1995
1775.34000	exclusion1524
1780.39000	exclusion1525
1780.89000	exclusion0920
1780.89000	exclusion1996
1784.88000	exclusion0921
1784.88000	exclusion1997
1784.89000	exclusion1526
1791.37000	exclusion0922
1791.88000	exclusion1527
1791.88000	exclusion1998
1801.89000	exclusion0923

1802.39000 exclusion1528
1808.89000 exclusion0924
1809.40000 exclusion1529
Neutral Loss Mass List: (none)
Product Mass List: (none)
Neutral loss in top: 3
Product in top: 3
Most intense if no parent masses found not enabled
Add/subtract mass not enabled
FT master scan preview mode enabled
Charge state screening enabled
Charge state dependent ETD time not enabled
Monoisotopic precursor selection enabled
Charge state rejection enabled
Unassigned charge states : rejected
Charge state 1 : rejected
Charge state 2 : not rejected
Charge state 3 : not rejected
Charge states 4+ : not rejected
Chromatography mode is disabled
Global Data Dependent Settings:
Predict ion injection time enabled
Use global parent and reject mass lists not enabled
Exclude parent mass from data dependent selection not enabled
Exclusion mass width by mass
Exclusion mass width low: 1.50000
Exclusion mass width high: 1.50000
Parent mass width by mass
Parent mass width low: 0.50000
Parent mass width high: 0.50000
Reject mass width by mass
Reject mass width low: 0.50000
Reject mass width high: 0.50000
Zoom/UltraZoom scan mass width by mass
Zoom/UltraZoom scan mass width low: 5.00
Zoom/UltraZoom scan mass width high: 5.00
FT SIM scan mass width low: 5.00
FT SIM scan mass width high: 5.00
Neutral Loss candidates processed by decreasing intensity
Neutral Loss mass width by mass
Neutral Loss mass width low: 0.50000
Neutral Loss mass width high: 0.50000
Product candidates processed by decreasing intensity
Product mass width by mass
Product mass width low: 0.50000
Product mass width high: 0.50000
MS mass range: 300.00-2000.00
MSn mass range by mass
MSn mass range: 0.00-1000000.00
Use m/z values as masses not enabled
Analog UV data dep. not enabled

Dynamic exclusion enabled
Repeat Count: 1
Repeat Duration: 30.00
Exclusion List Size: 500
Exclusion Duration: 180.00
Exclusion mass width by mass
Exclusion mass width low: 1.50000
Exclusion mass width high: 1.50000
Expiration: disabled
Isotopic data dependence not enabled
Custom Data Dependent Settings:
Not enabled

Pass 5 (wheat-mixed-digests_MS2_pass05_1.raw):

Creator: Orbi_30393
Last modified: 10/13/2021 by Orbi_30393
MS Run Time (min): 43.00
Sequence override of method parameters not enabled.
Divert Valve: not used during run
Contact Closure: not used during run
Syringe Pump: not used during run
MS Detector Settings:
Real-time modifications to method not enabled
Stepped collision energy not enabled
Additional Microscans:
MS2 0 0
MS3 0 0
MS4 0 0
MS5 0 0
MS6 0 0
MS7 0 0
MS8 0 0
MS9 0 0
MS10 0 0
Experiment Type: Nth Order Double Play
Tune Method: Orbitrap-tune-file_2020-03-13_HESI
Scan Event Details:
1: FTMS + p norm o(300.0-2000.0)
CV = 0.0V
2: ITMS + c norm Dep MS/MS Most intense ion from (1)
Activation Type: CID
Min. Signal Required: 500.0
Isolation Width: 2.00
Normalized Coll. Energy: 35.0
Default Charge State: 2
Activation Q: 0.250
Activation Time: 10.000
CV = 0.0V
Scan Event 2 repeated for top 10 peaks.
Lock Masses:

Pos List Name: N/A
Source: API Source
Mass List: (none)
Neg List Name: N/A
Source: API Source
Mass List: (none)

Data Dependent Settings:
Use separate polarity settings disabled
Parent Mass List: (none)
Reject Mass List: (none)
Neutral Loss Mass List: (none)
Product Mass List: (none)
Neutral loss in top: 3
Product in top: 3
Most intense if no parent masses found not enabled
Add/subtract mass not enabled
FT master scan preview mode enabled
Charge state screening enabled
Charge state dependent ETD time not enabled
Monoisotopic precursor selection enabled
Charge state rejection enabled
Unassigned charge states : rejected
Charge state 1 : rejected
Charge state 2 : not rejected
Charge state 3 : not rejected
Charge states 4+ : not rejected
Chromatography mode is disabled

Global Data Dependent Settings:
Predict ion injection time enabled
Use global parent and reject mass lists not enabled
Exclude parent mass from data dependent selection not enabled
Exclusion mass width by mass
Exclusion mass width low: 1.50000
Exclusion mass width high: 1.50000
Parent mass width by mass
Parent mass width low: 0.50000
Parent mass width high: 0.50000
Reject mass width by mass
Reject mass width low: 0.50000
Reject mass width high: 0.50000
Zoom/UltraZoom scan mass width by mass
Zoom/UltraZoom scan mass width low: 5.00
Zoom/UltraZoom scan mass width high: 5.00
FT SIM scan mass width low: 5.00
FT SIM scan mass width high: 5.00
Neutral Loss candidates processed by decreasing intensity
Neutral Loss mass width by mass
Neutral Loss mass width low: 0.50000
Neutral Loss mass width high: 0.50000
Product candidates processed by decreasing intensity
Product mass width by mass

Product mass width low: 0.50000
Product mass width high: 0.50000
MS mass range: 300.00-2000.00
MSn mass range by mass
MSn mass range: 0.00-1000000.00
Use m/z values as masses not enabled
Analog UV data dep. not enabled
Dynamic exclusion enabled
Repeat Count: 1
Repeat Duration: 30.00
Exclusion List Size: 500
Exclusion Duration: 180.00
Exclusion mass width by mass
Exclusion mass width low: 1.50000
Exclusion mass width high: 1.50000
Expiration: disabled
Isotopic data dependence not enabled
Custom Data Dependent Settings:
Not enabled

Pass 6 (wheat-mixed-digests_MS2_pass06_1.raw):

Creator: Orbi_30393
Last modified: 10/13/2021 by Orbi_30393
MS Run Time (min): 43.00
Sequence override of method parameters not enabled.
Divert Valve: not used during run
Contact Closure: not used during run
Syringe Pump: not used during run
MS Detector Settings:
Real-time modifications to method not enabled
Stepped collision energy not enabled
Additional Microscans:

MS2	0	0
MS3	0	0
MS4	0	0
MS5	0	0
MS6	0	0
MS7	0	0
MS8	0	0
MS9	0	0
MS10	0	0

Experiment Type: Nth Order Double Play
Tune Method: Orbitrap-tune-file_2020-03-13_HESI
Scan Event Details:
1: FTMS + p norm o(300.0-2000.0)
CV = 0.0V
2: ITMS + c norm Dep MS/MS Most intense ion from (1)
Activation Type: CID
Min. Signal Required: 3000.0
Isolation Width: 2.00

Normalized Coll. Energy: 35.0
Default Charge State: 2
Activation Q: 0.250
Activation Time: 10.000
CV = 0.0V
Scan Event 2 repeated for top 10 peaks.
Lock Masses:
Pos List Name: N/A
Source: API Source
Mass List: (none)
Neg List Name: N/A
Source: API Source
Mass List: (none)
Data Dependent Settings:
Use separate polarity settings disabled
Parent Mass List:

MS Mass	MS FAIMS CV	MS Normalized Collision Energy	MS2 Mass	MS2 Normalized Collision Energy	Name
300.18139		35.0			Cluster_18939
302.17822		35.0			Cluster_18998
304.10723		35.0			Cluster_19042
305.50412		35.0			Cluster_19081
307.12252		35.0			Cluster_19119
307.17583		35.0			Cluster_19131
307.17623		35.0			Cluster_19118
307.18423		35.0			Cluster_19126
307.50589		35.0			Cluster_06882
307.71572		35.0			Cluster_19142
307.84574		35.0			Cluster_06883
308.14269		35.0			Cluster_06884
308.14312		35.0			Cluster_06885
308.14934		35.0			Cluster_19160
308.64632		35.0			Cluster_19177
309.10936		35.0			Cluster_19188
309.67276		35.0			Cluster_19196
310.66456		35.0			Cluster_19221
310.86504		35.0			Cluster_19230
311.10568		35.0			Cluster_19240
312.17580		35.0			Cluster_19273
312.29236		35.0			Cluster_19281
312.52450		35.0			Cluster_19284
313.17441		35.0			Cluster_19302
313.50880		35.0			Cluster_19312
313.94655		35.0			Cluster_06933
314.11405		35.0			Cluster_19325
314.52501		35.0			Cluster_06938
314.85813		35.0			Cluster_06945
314.90987		35.0			Cluster_19353
315.17966		35.0			Cluster_19370

315.18040	35.0	Cluster_19367
315.18043	35.0	Cluster_19368
315.71422	35.0	Cluster_19400
318.85827	35.0	Cluster_06976
319.21104	35.0	Cluster_19555
319.68517	35.0	Cluster_19563
319.68541	35.0	Cluster_00847
319.68602	35.0	Cluster_00844
319.68635	35.0	Cluster_00845
320.91801	35.0	Cluster_19585
320.91883	35.0	Cluster_00861
320.91896	35.0	Cluster_00856
320.91918	35.0	Cluster_00852
321.15308	35.0	Cluster_06995
321.61792	35.0	Cluster_19601
321.72018	35.0	Cluster_19607
322.53614	35.0	Cluster_19633
323.19190	35.0	Cluster_19656
323.65030	35.0	Cluster_19672
323.68024	35.0	Cluster_19667
324.13156	35.0	Cluster_19688
324.42069	35.0	Cluster_00878
324.60316	35.0	Cluster_19711
324.66388	35.0	Cluster_19720
324.66471	35.0	Cluster_19719
325.15298	35.0	Cluster_19731
325.19669	35.0	Cluster_19741
326.61613	35.0	Cluster_19767
327.17150	35.0	Cluster_19785
327.44838	35.0	Cluster_19797
328.20600	35.0	Cluster_19817
329.00657	35.0	Cluster_19842
329.82193	35.0	Cluster_07063
330.10925	35.0	Cluster_19877
330.19915	35.0	Cluster_19884
330.90022	35.0	Cluster_19916
331.20771	35.0	Cluster_19930
331.22467	35.0	Cluster_19925
331.91322	35.0	Cluster_19943
331.92626	35.0	Cluster_00898
333.14933	35.0	Cluster_19981
333.86488	35.0	Cluster_07093
333.87455	35.0	Cluster_07090
334.51482	35.0	Cluster_20027
335.39009	35.0	Cluster_00911
335.42425	35.0	Cluster_20059
335.90453	35.0	Cluster_20079
336.18771	35.0	Cluster_07109
336.21532	35.0	Cluster_20094
336.46579	35.0	Cluster_20097
336.70926	35.0	Cluster_20102

337.19049	35.0	Cluster_20132
338.68639	35.0	Cluster_20181
338.93601	35.0	Cluster_20187
339.28949	35.0	Cluster_20198
339.49537	35.0	Cluster_07140
340.12729	35.0	Cluster_07154
341.18643	35.0	Cluster_20258
342.20057	35.0	Cluster_07171
342.22830	35.0	Cluster_20300
343.66746	35.0	Cluster_20336
344.15675	35.0	Cluster_20368
344.72229	35.0	Cluster_20384
345.15297	35.0	Cluster_07192
345.91798	35.0	Cluster_20415
346.71073	35.0	Cluster_20448
348.04600	35.0	Cluster_20480
348.64686	35.0	Cluster_00973
348.69149	35.0	Cluster_20506
349.24306	35.0	Cluster_07239
349.63790	35.0	Cluster_20543
350.11421	35.0	Cluster_20551
350.15978	35.0	Cluster_20553
351.23268	35.0	Cluster_20627
351.44817	35.0	Cluster_07249
351.70979	35.0	Cluster_20644
352.17409	35.0	Cluster_07256
352.65949	35.0	Cluster_20669
352.68040	35.0	Cluster_20674
354.69171	35.0	Cluster_20769
356.18535	35.0	Cluster_20822
356.21147	35.0	Cluster_20827
356.21666	35.0	Cluster_20830
356.54633	35.0	Cluster_07307
356.64829	35.0	Cluster_20835
356.86733	35.0	Cluster_07310
357.15015	35.0	Cluster_07312
357.63894	35.0	Cluster_20878
357.81560	35.0	Cluster_07316
357.83203	35.0	Cluster_20894
359.46536	35.0	Cluster_01042
359.87192	35.0	Cluster_07329
360.20680	35.0	Cluster_20980
360.53233	35.0	Cluster_07338
360.91407	35.0	Cluster_21012
361.42083	35.0	Cluster_01055
361.90202	35.0	Cluster_07352
362.22641	35.0	Cluster_21071
362.69593	35.0	Cluster_21078
363.10528	35.0	Cluster_21089
363.17084	35.0	Cluster_21090
363.18642	35.0	Cluster_21093

363.24378	35.0	Cluster_21098
364.15928	35.0	Cluster_21135
364.53487	35.0	Cluster_07386
365.21954	35.0	Cluster_21200
365.74467	35.0	Cluster_21233
365.86270	35.0	Cluster_07398
366.66719	35.0	Cluster_21282
367.19893	35.0	Cluster_21312
367.23906	35.0	Cluster_07411
367.54820	35.0	Cluster_21323
367.67913	35.0	Cluster_21329
368.18275	35.0	Cluster_21363
368.19973	35.0	Cluster_21366
368.20426	35.0	Cluster_21358
368.20533	35.0	Cluster_21369
368.35175	35.0	Cluster_21372
368.66636	35.0	Cluster_21380
368.68342	35.0	Cluster_21381
368.69841	35.0	Cluster_21379
369.18125	35.0	Cluster_21406
369.18336	35.0	Cluster_21407
369.68028	35.0	Cluster_21424
369.68403	35.0	Cluster_21443
370.08367	35.0	Cluster_21453
370.19785	35.0	Cluster_21468
370.25193	35.0	Cluster_21469
370.54002	35.0	Cluster_21470
370.73721	35.0	Cluster_21476
371.17419	35.0	Cluster_21493
371.27007	35.0	Cluster_21509
371.45807	35.0	Cluster_21511
371.60677	35.0	Cluster_21514
372.22058	35.0	Cluster_21545
372.67345	35.0	Cluster_21575
372.70722	35.0	Cluster_21577
373.21517	35.0	Cluster_21597
373.21690	35.0	Cluster_21590
374.70173	35.0	Cluster_21660
374.72356	35.0	Cluster_21659
375.17267	35.0	Cluster_21676
376.18825	35.0	Cluster_07499
376.19398	35.0	Cluster_21713
376.24488	35.0	Cluster_21722
377.20229	35.0	Cluster_21745
377.23336	35.0	Cluster_21758
377.69338	35.0	Cluster_21776
377.71834	35.0	Cluster_21774
378.18100	35.0	Cluster_21788
380.45221	35.0	Cluster_21886
380.72076	35.0	Cluster_21888
381.23578	35.0	Cluster_21923

381.72765	35.0	Cluster_21933
382.19501	35.0	Cluster_07559
382.19970	35.0	Cluster_21959
382.73846	35.0	Cluster_21985
383.16199	35.0	Cluster_07572
383.70026	35.0	Cluster_22026
383.89718	35.0	Cluster_07580
384.20618	35.0	Cluster_22055
384.20882	35.0	Cluster_22063
384.22734	35.0	Cluster_22048
384.23699	35.0	Cluster_22058
384.23964	35.0	Cluster_22056
384.95294	35.0	Cluster_22080
386.22103	35.0	Cluster_22144
386.36180	35.0	Cluster_22148
387.15735	35.0	Cluster_22178
387.92865	35.0	Cluster_22226
388.21127	35.0	Cluster_22239
390.07893	35.0	Cluster_22287
391.83529	35.0	Cluster_07662
391.88959	35.0	Cluster_07660
392.19049	35.0	Cluster_01189
393.16093	35.0	Cluster_22393
393.35115	35.0	Cluster_22422
393.35150	35.0	Cluster_22418
394.12425	35.0	Cluster_22482
394.23632	35.0	Cluster_22487
395.18490	35.0	Cluster_07695
395.22912	35.0	Cluster_22537
395.23140	35.0	Cluster_22535
396.19889	35.0	Cluster_07708
396.67333	35.0	Cluster_22609
396.88504	35.0	Cluster_22610
396.90372	35.0	Cluster_01219
397.21106	35.0	Cluster_07728
397.21183	35.0	Cluster_07721
397.21517	35.0	Cluster_07729
397.21518	35.0	Cluster_07727
398.54590	35.0	Cluster_07741
400.68518	35.0	Cluster_22777
400.73920	35.0	Cluster_22787
400.76739	35.0	Cluster_22792
401.12569	35.0	Cluster_22796
402.47822	35.0	Cluster_22868
403.21431	35.0	Cluster_22897
403.22681	35.0	Cluster_22898
403.47621	35.0	Cluster_22910
403.50556	35.0	Cluster_01262
403.54121	35.0	Cluster_07797
403.71289	35.0	Cluster_22924
405.20527	35.0	Cluster_22990

405.95533	35.0	Cluster_01279
406.19169	35.0	Cluster_23017
406.25032	35.0	Cluster_23028
406.25211	35.0	Cluster_23025
407.19440	35.0	Cluster_01299
407.23778	35.0	Cluster_23063
407.69970	35.0	Cluster_23073
408.23545	35.0	Cluster_07858
408.54132	35.0	Cluster_07863
409.12920	35.0	Cluster_23141
409.13151	35.0	Cluster_23142
409.21581	35.0	Cluster_07870
409.22568	35.0	Cluster_23153
409.43498	35.0	Cluster_01310
409.57292	35.0	Cluster_23167
409.73324	35.0	Cluster_23180
410.21306	35.0	Cluster_23192
410.43506	35.0	Cluster_23203
410.69545	35.0	Cluster_23208
410.88152	35.0	Cluster_23217
411.29757	35.0	Cluster_23239
411.46011	35.0	Cluster_01319
411.46179	35.0	Cluster_23246
411.87723	35.0	Cluster_07897
412.22721	35.0	Cluster_23277
412.36399	35.0	Cluster_07901
412.44074	35.0	Cluster_23284
412.99218	35.0	Cluster_23308
413.16510	35.0	Cluster_01330
413.42901	35.0	Cluster_23332
413.70849	35.0	Cluster_01337
413.72640	35.0	Cluster_23344
413.76861	35.0	Cluster_23346
414.75386	35.0	Cluster_23381
415.19319	35.0	Cluster_23407
416.76401	35.0	Cluster_23476
416.96144	35.0	Cluster_23484
417.24118	35.0	Cluster_23495
417.70707	35.0	Cluster_23504
417.93206	35.0	Cluster_07965
418.27534	35.0	Cluster_23531
418.68587	35.0	Cluster_23550
418.73063	35.0	Cluster_23554
419.16928	35.0	Cluster_23561
420.19295	35.0	Cluster_23617
420.48611	35.0	Cluster_23629
420.99622	35.0	Cluster_23649
421.17949	35.0	Cluster_07996
421.25549	35.0	Cluster_23656
421.74177	35.0	Cluster_23691
422.24188	35.0	Cluster_08011

422.75222	35.0	Cluster_23720
423.24596	35.0	Cluster_23732
423.25021	35.0	Cluster_23734
423.26452	35.0	Cluster_23744
423.71445	35.0	Cluster_23753
423.71797	35.0	Cluster_23757
423.86317	35.0	Cluster_08030
424.21310	35.0	Cluster_23791
424.24130	35.0	Cluster_08033
424.71578	35.0	Cluster_23830
425.21353	35.0	Cluster_23847
425.22563	35.0	Cluster_23845
425.23397	35.0	Cluster_08046
425.75390	35.0	Cluster_23890
425.75537	35.0	Cluster_23891
425.86680	35.0	Cluster_08056
426.21748	35.0	Cluster_23928
426.25210	35.0	Cluster_23935
426.85610	35.0	Cluster_23956
426.96516	35.0	Cluster_23959
427.45165	35.0	Cluster_01418
427.73954	35.0	Cluster_23990
427.77055	35.0	Cluster_23996
427.77629	35.0	Cluster_23986
428.20390	35.0	Cluster_01423
428.26325	35.0	Cluster_24023
428.27627	35.0	Cluster_24030
428.73429	35.0	Cluster_24052
429.17967	35.0	Cluster_24066
429.56584	35.0	Cluster_08120
430.22949	35.0	Cluster_08134
431.27472	35.0	Cluster_24169
431.55978	35.0	Cluster_08150
432.46095	35.0	Cluster_24230
433.16955	35.0	Cluster_24257
433.24369	35.0	Cluster_24263
433.28448	35.0	Cluster_24266
434.76872	35.0	Cluster_24329
435.22149	35.0	Cluster_24369
435.22203	35.0	Cluster_24359
435.90332	35.0	Cluster_08208
436.27565	35.0	Cluster_24432
436.47569	35.0	Cluster_01491
436.56693	35.0	Cluster_24436
436.56866	35.0	Cluster_01492
436.72309	35.0	Cluster_24448
436.77680	35.0	Cluster_24452
436.78365	35.0	Cluster_24453
437.24974	35.0	Cluster_08221
438.52285	35.0	Cluster_24544
438.73222	35.0	Cluster_24563

438.90950	35.0	Cluster_24583
439.25435	35.0	Cluster_24589
439.25668	35.0	Cluster_24587
439.76705	35.0	Cluster_24627
439.90281	35.0	Cluster_08267
440.21796	35.0	Cluster_24645
440.73012	35.0	Cluster_24657
440.95090	35.0	Cluster_24671
441.06270	35.0	Cluster_24673
441.14804	35.0	Cluster_08284
441.19838	35.0	Cluster_08283
441.65909	35.0	Cluster_24701
442.22077	35.0	Cluster_08299
442.48368	35.0	Cluster_24747
442.73721	35.0	Cluster_24752
442.89667	35.0	Cluster_24764
443.22990	35.0	Cluster_01524
443.29019	35.0	Cluster_24795
443.72248	35.0	Cluster_01529
444.56992	35.0	Cluster_24864
444.62609	35.0	Cluster_24865
444.80902	35.0	Cluster_24880
444.98546	35.0	Cluster_24883
444.98560	35.0	Cluster_24882
444.98721	35.0	Cluster_01544
445.55386	35.0	Cluster_24900
445.66341	35.0	Cluster_01549
445.70794	35.0	Cluster_24905
445.72605	35.0	Cluster_24909
446.30454	35.0	Cluster_24942
446.55756	35.0	Cluster_24946
446.74957	35.0	Cluster_01557
447.19331	35.0	Cluster_24975
447.29041	35.0	Cluster_24988
447.29684	35.0	Cluster_24980
447.47895	35.0	Cluster_24992
447.76941	35.0	Cluster_25002
448.21671	35.0	Cluster_08397
448.23477	35.0	Cluster_25021
448.57791	35.0	Cluster_08400
449.27084	35.0	Cluster_25066
449.95854	35.0	Cluster_25103
450.27739	35.0	Cluster_25114
450.56658	35.0	Cluster_25130
451.77488	35.0	Cluster_25180
452.01254	35.0	Cluster_25192
452.75396	35.0	Cluster_25235
453.70221	35.0	Cluster_01604
454.19131	35.0	Cluster_08500
454.58119	35.0	Cluster_08511
454.70208	35.0	Cluster_25319

455.20057	35.0	Cluster_08518
455.47836	35.0	Cluster_01621
455.81190	35.0	Cluster_25364
455.92600	35.0	Cluster_08527
456.79120	35.0	Cluster_25411
457.23466	35.0	Cluster_25428
457.23627	35.0	Cluster_25426
457.26552	35.0	Cluster_25430
457.95445	35.0	Cluster_08555
458.78823	35.0	Cluster_25504
458.79020	35.0	Cluster_25505
459.99743	35.0	Cluster_25551
461.16305	35.0	Cluster_08588
461.24082	35.0	Cluster_08590
461.26795	35.0	Cluster_25609
461.26968	35.0	Cluster_25607
461.45638	35.0	Cluster_25615
461.55248	35.0	Cluster_25617
461.57128	35.0	Cluster_08594
461.67636	35.0	Cluster_25619
461.72794	35.0	Cluster_25620
461.92659	35.0	Cluster_08602
462.24726	35.0	Cluster_25635
462.52195	35.0	Cluster_08614
462.71081	35.0	Cluster_25647
462.71773	35.0	Cluster_25648
463.22978	35.0	Cluster_25667
463.28631	35.0	Cluster_25669
463.51536	35.0	Cluster_01676
463.74334	35.0	Cluster_25680
463.98229	35.0	Cluster_25697
464.22084	35.0	Cluster_08632
464.97394	35.0	Cluster_25759
464.97417	35.0	Cluster_25764
464.97567	35.0	Cluster_25761
465.24645	35.0	Cluster_25784
465.27096	35.0	Cluster_25773
465.27897	35.0	Cluster_25781
466.75077	35.0	Cluster_25838
466.90772	35.0	Cluster_25848
467.57948	35.0	Cluster_08687
468.74197	35.0	Cluster_25932
468.75878	35.0	Cluster_25925
468.93506	35.0	Cluster_08710
469.20920	35.0	Cluster_08717
469.24421	35.0	Cluster_25946
469.25453	35.0	Cluster_08719
469.25778	35.0	Cluster_08720
469.48106	35.0	Cluster_01715
469.78252	35.0	Cluster_25970
470.23518	35.0	Cluster_25990

470.23714	35.0	Cluster_25992
470.24168	35.0	Cluster_08729
470.31187	35.0	Cluster_25999
470.73989	35.0	Cluster_01726
470.95744	35.0	Cluster_26025
471.25377	35.0	Cluster_01737
471.78022	35.0	Cluster_26056
471.85656	35.0	Cluster_26069
472.28933	35.0	Cluster_26090
473.00613	35.0	Cluster_26120
473.25522	35.0	Cluster_26141
473.26784	35.0	Cluster_26146
473.50841	35.0	Cluster_26151
473.61584	35.0	Cluster_08782
474.25392	35.0	Cluster_01752
474.74844	35.0	Cluster_26188
474.82517	35.0	Cluster_26201
475.20902	35.0	Cluster_26205
475.25075	35.0	Cluster_26223
475.91606	35.0	Cluster_08825
476.77033	35.0	Cluster_26267
477.91699	35.0	Cluster_08866
478.21889	35.0	Cluster_26309
478.50171	35.0	Cluster_26327
478.51894	35.0	Cluster_08877
478.79293	35.0	Cluster_26336
479.23947	35.0	Cluster_26383
479.24655	35.0	Cluster_26380
479.24822	35.0	Cluster_26381
479.32696	35.0	Cluster_26391
479.48057	35.0	Cluster_26410
479.98975	35.0	Cluster_26444
480.19347	35.0	Cluster_26456
480.23128	35.0	Cluster_26461
480.87318	35.0	Cluster_08912
481.21585	35.0	Cluster_26502
481.22448	35.0	Cluster_26493
481.55886	35.0	Cluster_08922
482.56792	35.0	Cluster_26551
482.75063	35.0	Cluster_26575
482.75333	35.0	Cluster_26573
483.26706	35.0	Cluster_01822
483.28102	35.0	Cluster_26592
483.51623	35.0	Cluster_26609
483.77330	35.0	Cluster_26614
484.26988	35.0	Cluster_26656
484.56376	35.0	Cluster_26659
484.74531	35.0	Cluster_26674
486.05408	35.0	Cluster_26738
486.30822	35.0	Cluster_26750
486.77412	35.0	Cluster_26773

487.78232	35.0	Cluster_26821
488.49865	35.0	Cluster_01848
488.59040	35.0	Cluster_09031
488.59139	35.0	Cluster_09030
488.99668	35.0	Cluster_26865
488.99712	35.0	Cluster_26866
489.27346	35.0	Cluster_09043
489.31443	35.0	Cluster_26892
489.62012	35.0	Cluster_09044
489.95299	35.0	Cluster_09049
490.92353	35.0	Cluster_26947
491.21912	35.0	Cluster_26962
491.24758	35.0	Cluster_26961
491.29815	35.0	Cluster_09076
491.79438	35.0	Cluster_26978
491.79691	35.0	Cluster_26976
491.95912	35.0	Cluster_09085
492.23402	35.0	Cluster_26996
492.25131	35.0	Cluster_01878
492.26447	35.0	Cluster_27001
492.56112	35.0	Cluster_27005
492.62984	35.0	Cluster_00178
493.20484	35.0	Cluster_27036
493.70557	35.0	Cluster_27072
493.77046	35.0	Cluster_27075
493.97846	35.0	Cluster_27098
494.24345	35.0	Cluster_09119
494.26012	35.0	Cluster_27100
494.30737	35.0	Cluster_27115
494.58050	35.0	Cluster_09124
495.18779	35.0	Cluster_27149
495.24938	35.0	Cluster_27147
495.25575	35.0	Cluster_09135
495.49799	35.0	Cluster_27166
495.90563	35.0	Cluster_09146
496.76962	35.0	Cluster_27229
497.24142	35.0	Cluster_27241
497.56231	35.0	Cluster_09183
497.74856	35.0	Cluster_01911
497.75069	35.0	Cluster_01921
497.78784	35.0	Cluster_27268
498.25766	35.0	Cluster_09195
498.26429	35.0	Cluster_27284
498.28088	35.0	Cluster_27285
498.50412	35.0	Cluster_27299
498.60981	35.0	Cluster_09200
498.77805	35.0	Cluster_27303
498.78258	35.0	Cluster_27312
499.29594	35.0	Cluster_27338
499.50829	35.0	Cluster_01942
499.70562	35.0	Cluster_27342

500.74414	35.0	Cluster_01949
501.76450	35.0	Cluster_27459
502.78120	35.0	Cluster_27488
502.81600	35.0	Cluster_27498
502.81913	35.0	Cluster_27495
503.24700	35.0	Cluster_27512
503.25273	35.0	Cluster_27520
503.50051	35.0	Cluster_27526
504.28071	35.0	Cluster_27541
504.29921	35.0	Cluster_27556
505.25105	35.0	Cluster_09295
505.28005	35.0	Cluster_27584
505.28920	35.0	Cluster_27589
505.29389	35.0	Cluster_27576
505.71880	35.0	Cluster_27594
505.72781	35.0	Cluster_01992
505.77895	35.0	Cluster_27595
506.20698	35.0	Cluster_09317
506.23976	35.0	Cluster_09316
506.25029	35.0	Cluster_09318
506.25580	35.0	Cluster_09321
506.72657	35.0	Cluster_27611
507.27444	35.0	Cluster_27625
507.32513	35.0	Cluster_09337
507.55637	35.0	Cluster_27647
507.56368	35.0	Cluster_09340
507.73798	35.0	Cluster_02005
507.80290	35.0	Cluster_27650
507.80497	35.0	Cluster_27658
508.29129	35.0	Cluster_27676
508.75919	35.0	Cluster_27690
509.57744	35.0	Cluster_09372
510.02497	35.0	Cluster_02027
510.24236	35.0	Cluster_27767
510.26739	35.0	Cluster_27773
510.60055	35.0	Cluster_09393
510.62285	35.0	Cluster_09390
510.80102	35.0	Cluster_27809
511.00698	35.0	Cluster_27816
511.20283	35.0	Cluster_27818
511.23726	35.0	Cluster_09403
511.67620	35.0	Cluster_27837
512.25496	35.0	Cluster_27867
512.27120	35.0	Cluster_27865
512.27809	35.0	Cluster_27872
513.21850	35.0	Cluster_27907
513.28698	35.0	Cluster_09437
513.30292	35.0	Cluster_27916
513.82143	35.0	Cluster_27928
514.23162	35.0	Cluster_27944
514.29844	35.0	Cluster_27956

514.72678	35.0	Cluster_27962
515.03858	35.0	Cluster_02052
515.42879	35.0	Cluster_28005
515.90242	35.0	Cluster_09478
516.23238	35.0	Cluster_28027
516.24464	35.0	Cluster_28041
516.78373	35.0	Cluster_28064
516.78653	35.0	Cluster_28060
518.59799	35.0	Cluster_09517
518.63157	35.0	Cluster_09519
518.68185	35.0	Cluster_28149
518.75080	35.0	Cluster_28148
519.29071	35.0	Cluster_28177
519.44164	35.0	Cluster_28179
519.52561	35.0	Cluster_28182
520.72991	35.0	Cluster_28238
521.03117	35.0	Cluster_02096
521.72792	35.0	Cluster_28263
522.21350	35.0	Cluster_02103
522.25391	35.0	Cluster_28293
522.28500	35.0	Cluster_09575
523.26552	35.0	Cluster_28334
523.29131	35.0	Cluster_09594
523.83465	35.0	Cluster_28370
523.99562	35.0	Cluster_09605
524.75967	35.0	Cluster_28399
524.76802	35.0	Cluster_28395
524.82869	35.0	Cluster_28404
524.96550	35.0	Cluster_09628
525.01312	35.0	Cluster_28407
525.24753	35.0	Cluster_28422
525.26257	35.0	Cluster_28419
525.31479	35.0	Cluster_28430
525.74833	35.0	Cluster_02122
525.78681	35.0	Cluster_28443
526.21218	35.0	Cluster_09659
526.28131	35.0	Cluster_28458
526.30647	35.0	Cluster_09666
526.55381	35.0	Cluster_28469
526.77481	35.0	Cluster_28471
526.92737	35.0	Cluster_09676
527.31121	35.0	Cluster_28488
527.40923	35.0	Cluster_28492
527.73779	35.0	Cluster_28503
527.80726	35.0	Cluster_28514
527.95744	35.0	Cluster_09698
527.96172	35.0	Cluster_09696
527.97250	35.0	Cluster_09700
528.26935	35.0	Cluster_00184
528.30280	35.0	Cluster_09702
528.30501	35.0	Cluster_28550

528.32878	35.0	Cluster_28540
528.46671	35.0	Cluster_28558
528.49276	35.0	Cluster_28556
529.27669	35.0	Cluster_28578
529.46801	35.0	Cluster_02143
530.02188	35.0	Cluster_28624
530.29494	35.0	Cluster_28649
530.30380	35.0	Cluster_28641
530.78294	35.0	Cluster_28675
531.19942	35.0	Cluster_09748
531.25743	35.0	Cluster_28699
531.26425	35.0	Cluster_28687
532.96674	35.0	Cluster_09773
533.26423	35.0	Cluster_28757
533.26639	35.0	Cluster_28756
533.28262	35.0	Cluster_28768
533.34161	35.0	Cluster_28774
533.75392	35.0	Cluster_28791
533.77168	35.0	Cluster_28786
533.91745	35.0	Cluster_09785
533.92278	35.0	Cluster_09791
534.53000	35.0	Cluster_28822
534.75817	35.0	Cluster_28839
535.32665	35.0	Cluster_09813
537.26984	35.0	Cluster_28925
537.29559	35.0	Cluster_28936
537.30451	35.0	Cluster_02195
537.52975	35.0	Cluster_02196
537.79197	35.0	Cluster_28941
537.91326	35.0	Cluster_09869
538.52689	35.0	Cluster_28962
538.79448	35.0	Cluster_28978
539.73333	35.0	Cluster_02212
539.87213	35.0	Cluster_29005
539.97028	35.0	Cluster_09906
539.98204	35.0	Cluster_09905
540.02231	35.0	Cluster_09899
540.26271	35.0	Cluster_29033
540.58133	35.0	Cluster_09919
540.93901	35.0	Cluster_09927
541.04334	35.0	Cluster_29059
541.12109	35.0	Cluster_29068
541.26220	35.0	Cluster_29080
541.79336	35.0	Cluster_29114
542.33816	35.0	Cluster_29137
542.52109	35.0	Cluster_02234
542.52124	35.0	Cluster_02233
542.67811	35.0	Cluster_29147
542.80738	35.0	Cluster_29176
542.81002	35.0	Cluster_29175
543.23404	35.0	Cluster_02240

543.26414	35.0	Cluster_09960
543.27696	35.0	Cluster_29192
543.48293	35.0	Cluster_02239
543.97154	35.0	Cluster_09968
544.61757	35.0	Cluster_09977
544.75729	35.0	Cluster_29233
545.24926	35.0	Cluster_29272
545.53016	35.0	Cluster_02260
545.53044	35.0	Cluster_29276
545.76869	35.0	Cluster_29280
545.77989	35.0	Cluster_29284
545.82018	35.0	Cluster_29288
546.78186	35.0	Cluster_29325
546.78372	35.0	Cluster_29324
546.83805	35.0	Cluster_29334
547.62967	35.0	Cluster_10018
548.62278	35.0	Cluster_10038
548.63524	35.0	Cluster_10037
548.98839	35.0	Cluster_29415
549.26773	35.0	Cluster_29422
549.98566	35.0	Cluster_10058
550.04160	35.0	Cluster_29449
550.28949	35.0	Cluster_10064
550.79143	35.0	Cluster_29466
551.79855	35.0	Cluster_29517
552.72294	35.0	Cluster_29549
552.79997	35.0	Cluster_29559
552.81760	35.0	Cluster_29561
553.30563	35.0	Cluster_10111
553.51882	35.0	Cluster_02298
553.54594	35.0	Cluster_29579
554.25070	35.0	Cluster_02300
554.75239	35.0	Cluster_29631
555.28796	35.0	Cluster_10147
555.29621	35.0	Cluster_29653
555.65390	35.0	Cluster_10160
555.65807	35.0	Cluster_10161
556.27635	35.0	Cluster_29690
557.01747	35.0	Cluster_29723
557.30706	35.0	Cluster_29740
557.31245	35.0	Cluster_10191
557.83386	35.0	Cluster_29760
559.54108	35.0	Cluster_02335
559.55624	35.0	Cluster_29814
559.70118	35.0	Cluster_29812
559.79273	35.0	Cluster_29826
560.23774	35.0	Cluster_29837
560.25365	35.0	Cluster_10240
560.34293	35.0	Cluster_29841
560.61957	35.0	Cluster_10242
561.03156	35.0	Cluster_29867

561.05796	35.0	Cluster_29871
561.28991	35.0	Cluster_29882
561.30986	35.0	Cluster_29873
561.55399	35.0	Cluster_29893
561.79208	35.0	Cluster_29903
562.19999	35.0	Cluster_29911
562.80554	35.0	Cluster_02348
562.81178	35.0	Cluster_29927
563.05489	35.0	Cluster_29945
563.06345	35.0	Cluster_29939
563.06864	35.0	Cluster_29942
563.79657	35.0	Cluster_02357
565.58903	35.0	Cluster_10320
565.81858	35.0	Cluster_30054
566.29562	35.0	Cluster_30075
566.29597	35.0	Cluster_30076
566.90411	35.0	Cluster_30092
567.32070	35.0	Cluster_10347
568.29705	35.0	Cluster_10359
568.75120	35.0	Cluster_30170
568.79681	35.0	Cluster_30176
569.53416	35.0	Cluster_30199
571.81791	35.0	Cluster_30271
571.87618	35.0	Cluster_10410
571.98268	35.0	Cluster_10418
572.32261	35.0	Cluster_30285
574.26044	35.0	Cluster_10456
574.28417	35.0	Cluster_10460
574.47512	35.0	Cluster_00219
576.51907	35.0	Cluster_30411
576.53992	35.0	Cluster_30412
577.47477	35.0	Cluster_00221
577.74768	35.0	Cluster_30445
577.78800	35.0	Cluster_30435
578.81497	35.0	Cluster_30477
579.78136	35.0	Cluster_30511
580.28827	35.0	Cluster_30554
580.52520	35.0	Cluster_02465
580.85968	35.0	Cluster_02466
581.79816	35.0	Cluster_02475
581.89041	35.0	Cluster_10562
582.06200	35.0	Cluster_30614
582.30425	35.0	Cluster_30634
582.35907	35.0	Cluster_30651
584.75963	35.0	Cluster_30741
584.99621	35.0	Cluster_10603
586.03148	35.0	Cluster_30798
586.05382	35.0	Cluster_02506
586.06372	35.0	Cluster_30797
586.34272	35.0	Cluster_30803
586.65752	35.0	Cluster_10632

586.66077	35.0	Cluster_10633
587.81633	35.0	Cluster_30865
587.99851	35.0	Cluster_10651
588.27868	35.0	Cluster_30879
588.29050	35.0	Cluster_30880
588.31813	35.0	Cluster_10661
588.32128	35.0	Cluster_10664
588.79784	35.0	Cluster_30889
588.80006	35.0	Cluster_30887
589.09090	35.0	Cluster_02521
589.31162	35.0	Cluster_30920
589.31328	35.0	Cluster_30912
589.34271	35.0	Cluster_30916
590.28217	35.0	Cluster_30964
590.29951	35.0	Cluster_30953
590.49205	35.0	Cluster_02529
590.87542	35.0	Cluster_30993
590.87542	35.0	Cluster_30994
590.99826	35.0	Cluster_30999
591.64593	35.0	Cluster_10702
591.77316	35.0	Cluster_31010
591.77316	35.0	Cluster_31015
592.03205	35.0	Cluster_31028
592.37681	35.0	Cluster_31036
592.90589	35.0	Cluster_31063
592.97018	35.0	Cluster_10720
593.06175	35.0	Cluster_02547
593.29794	35.0	Cluster_31068
593.99147	35.0	Cluster_10739
594.81425	35.0	Cluster_31119
595.07225	35.0	Cluster_02556
595.32870	35.0	Cluster_10766
595.57788	35.0	Cluster_31139
597.33731	35.0	Cluster_31195
597.35703	35.0	Cluster_31199
598.32380	35.0	Cluster_02578
598.62702	35.0	Cluster_10823
599.81361	35.0	Cluster_02581
599.84927	35.0	Cluster_31286
601.76195	35.0	Cluster_02594
601.85727	35.0	Cluster_31366
601.86414	35.0	Cluster_31365
602.31203	35.0	Cluster_31391
603.53345	35.0	Cluster_31419
603.60560	35.0	Cluster_31422
603.94866	35.0	Cluster_31432
604.36908	35.0	Cluster_31449
605.67389	35.0	Cluster_10910
606.36332	35.0	Cluster_31521
606.62627	35.0	Cluster_10929
606.63827	35.0	Cluster_10932

606.99602	35.0	Cluster_10938
607.81374	35.0	Cluster_31552
607.86967	35.0	Cluster_31561
608.00218	35.0	Cluster_10951
608.32379	35.0	Cluster_31587
608.37907	35.0	Cluster_31579
609.53174	35.0	Cluster_31620
609.54399	35.0	Cluster_31621
610.58909	35.0	Cluster_31662
610.81214	35.0	Cluster_31672
611.77453	35.0	Cluster_31712
611.95982	35.0	Cluster_11001
612.30404	35.0	Cluster_31723
613.33866	35.0	Cluster_31764
613.84388	35.0	Cluster_31788
614.30736	35.0	Cluster_31801
614.32829	35.0	Cluster_31804
614.58419	35.0	Cluster_31817
615.31368	35.0	Cluster_31835
615.36301	35.0	Cluster_31840
615.65857	35.0	Cluster_11061
616.34935	35.0	Cluster_31871
616.54862	35.0	Cluster_31873
616.81547	35.0	Cluster_31888
617.81621	35.0	Cluster_31926
617.99735	35.0	Cluster_11092
619.23737	35.0	Cluster_31977
619.23825	35.0	Cluster_31978
619.33872	35.0	Cluster_31982
619.63456	35.0	Cluster_11118
619.65655	35.0	Cluster_11120
619.78725	35.0	Cluster_31996
620.32652	35.0	Cluster_32018
620.38097	35.0	Cluster_32024
620.83282	35.0	Cluster_32031
621.29918	35.0	Cluster_32049
621.39040	35.0	Cluster_32052
622.79607	35.0	Cluster_32093
623.30190	35.0	Cluster_32111
623.64928	35.0	Cluster_11191
623.83853	35.0	Cluster_32135
624.98651	35.0	Cluster_11209
625.85548	35.0	Cluster_32203
625.97156	35.0	Cluster_11220
626.01878	35.0	Cluster_02740
626.31307	35.0	Cluster_11232
626.38225	35.0	Cluster_32224
627.11200	35.0	Cluster_02743
627.38196	35.0	Cluster_32271
627.62311	35.0	Cluster_11256
628.00286	35.0	Cluster_11263

628.33999	35.0	Cluster_11261
628.55578	35.0	Cluster_32312
628.84969	35.0	Cluster_32316
630.09806	35.0	Cluster_02750
630.86016	35.0	Cluster_32381
632.72081	35.0	Cluster_32443
632.80122	35.0	Cluster_32448
634.29435	35.0	Cluster_11358
634.31779	35.0	Cluster_32515
634.35247	35.0	Cluster_11350
635.82948	35.0	Cluster_32562
636.99289	35.0	Cluster_11392
637.33236	35.0	Cluster_32612
637.62019	35.0	Cluster_11403
638.31773	35.0	Cluster_11412
638.34363	35.0	Cluster_32654
639.31559	35.0	Cluster_32685
639.68269	35.0	Cluster_11437
639.80812	35.0	Cluster_32706
640.82008	35.0	Cluster_32751
640.83128	35.0	Cluster_32746
641.04752	35.0	Cluster_32764
642.81785	35.0	Cluster_32830
643.08841	35.0	Cluster_32841
645.09355	35.0	Cluster_02837
645.31567	35.0	Cluster_00252
645.84840	35.0	Cluster_32926
646.05937	35.0	Cluster_02847
646.37710	35.0	Cluster_32949
646.95918	35.0	Cluster_11539
648.39547	35.0	Cluster_33012
649.07347	35.0	Cluster_33032
649.25206	35.0	Cluster_33036
649.56122	35.0	Cluster_02868
649.76473	35.0	Cluster_33053
649.86734	35.0	Cluster_33068
651.06179	35.0	Cluster_33115
651.66012	35.0	Cluster_11603
654.75497	35.0	Cluster_33236
655.81688	35.0	Cluster_33275
658.66615	35.0	Cluster_33366
658.79165	35.0	Cluster_33375
659.02086	35.0	Cluster_11706
659.37949	35.0	Cluster_11709
659.54833	35.0	Cluster_33395
659.62589	35.0	Cluster_02907
661.33894	35.0	Cluster_11734
661.79061	35.0	Cluster_33460
662.11757	35.0	Cluster_02928
662.69475	35.0	Cluster_11762
662.82543	35.0	Cluster_02934

662.94506	35.0	Cluster_00261
663.08145	35.0	Cluster_33501
663.25841	35.0	Cluster_33503
663.38606	35.0	Cluster_33507
663.60178	35.0	Cluster_02940
664.37085	35.0	Cluster_33540
664.81843	35.0	Cluster_33548
665.35530	35.0	Cluster_33569
665.82233	35.0	Cluster_02959
665.84730	35.0	Cluster_33575
666.34744	35.0	Cluster_02964
666.37680	35.0	Cluster_33591
667.81938	35.0	Cluster_33644
667.84739	35.0	Cluster_33646
667.89684	35.0	Cluster_33648
667.97367	35.0	Cluster_11845
667.98825	35.0	Cluster_11843
668.32263	35.0	Cluster_11851
668.33838	35.0	Cluster_33655
668.65012	35.0	Cluster_11854
669.09668	35.0	Cluster_02977
669.11109	35.0	Cluster_02976
669.16846	35.0	Cluster_00267
669.32694	35.0	Cluster_33680
669.69725	35.0	Cluster_00270
669.85195	35.0	Cluster_02980
670.38121	35.0	Cluster_33722
670.38953	35.0	Cluster_33710
670.38953	35.0	Cluster_33726
670.81349	35.0	Cluster_33737
671.10547	35.0	Cluster_02987
671.36374	35.0	Cluster_33756
671.86108	35.0	Cluster_33771
672.38383	35.0	Cluster_11909
673.69262	35.0	Cluster_11928
673.84601	35.0	Cluster_33828
674.37802	35.0	Cluster_33841
675.28713	35.0	Cluster_11947
675.63966	35.0	Cluster_03025
675.86022	35.0	Cluster_33867
676.04126	35.0	Cluster_33882
676.06023	35.0	Cluster_33875
676.82567	35.0	Cluster_33894
677.38049	35.0	Cluster_33924
677.66310	35.0	Cluster_11983
678.05380	35.0	Cluster_11985
678.07682	35.0	Cluster_03041
678.10788	35.0	Cluster_33950
678.61132	35.0	Cluster_03048
679.35854	35.0	Cluster_12002
680.39313	35.0	Cluster_12012

680.68664	35.0	Cluster_12015
680.79621	35.0	Cluster_34046
680.80240	35.0	Cluster_34051
681.68356	35.0	Cluster_12028
683.17868	35.0	Cluster_34121
683.50325	35.0	Cluster_00281
683.61484	35.0	Cluster_12059
684.33882	35.0	Cluster_34147
684.61364	35.0	Cluster_12066
684.78469	35.0	Cluster_34165
684.81441	35.0	Cluster_34164
684.82466	35.0	Cluster_34171
685.08807	35.0	Cluster_03103
685.08939	35.0	Cluster_03095
685.08962	35.0	Cluster_34186
685.33558	35.0	Cluster_34202
685.34195	35.0	Cluster_12078
685.84087	35.0	Cluster_34213
687.37344	35.0	Cluster_34250
687.56541	35.0	Cluster_03119
687.79947	35.0	Cluster_34262
687.80190	35.0	Cluster_34258
687.88397	35.0	Cluster_03131
687.88623	35.0	Cluster_03124
688.01588	35.0	Cluster_12113
688.27721	35.0	Cluster_34272
688.34085	35.0	Cluster_34279
688.38593	35.0	Cluster_34282
688.84881	35.0	Cluster_34292
688.85810	35.0	Cluster_03136
688.85987	35.0	Cluster_34290
690.32779	35.0	Cluster_03148
690.82663	35.0	Cluster_34322
691.07637	35.0	Cluster_34336
691.10745	35.0	Cluster_34337
691.81429	35.0	Cluster_03161
692.58945	35.0	Cluster_03180
692.59083	35.0	Cluster_03181
692.59314	35.0	Cluster_03176
692.59958	35.0	Cluster_03182
693.01929	35.0	Cluster_12161
693.09427	35.0	Cluster_34407
693.99699	35.0	Cluster_12168
694.06755	35.0	Cluster_34421
694.67508	35.0	Cluster_12172
695.04142	35.0	Cluster_12177
695.33781	35.0	Cluster_03206
695.61408	35.0	Cluster_34466
696.02156	35.0	Cluster_12198
696.03203	35.0	Cluster_12194
696.31580	35.0	Cluster_34489

696.36747	35.0	Cluster_34481
696.99121	35.0	Cluster_12209
697.02744	35.0	Cluster_12212
697.40157	35.0	Cluster_34514
698.56565	35.0	Cluster_03224
698.56567	35.0	Cluster_03225
698.69069	35.0	Cluster_12232
699.09398	35.0	Cluster_34549
699.34183	35.0	Cluster_12236
699.70254	35.0	Cluster_12244
699.90969	35.0	Cluster_34574
700.06571	35.0	Cluster_03240
700.31327	35.0	Cluster_34581
700.87788	35.0	Cluster_34610
701.80476	35.0	Cluster_34627
702.28565	35.0	Cluster_34650
702.29475	35.0	Cluster_34647
702.62288	35.0	Cluster_12286
702.93375	35.0	Cluster_34673
703.31422	35.0	Cluster_34685
703.60021	35.0	Cluster_34686
704.21072	35.0	Cluster_34700
704.32933	35.0	Cluster_34707
704.35627	35.0	Cluster_12313
704.78009	35.0	Cluster_34721
704.85837	35.0	Cluster_34731
704.88607	35.0	Cluster_34735
705.02263	35.0	Cluster_12328
705.34861	35.0	Cluster_12324
705.41349	35.0	Cluster_34748
705.70698	35.0	Cluster_12329
706.56252	35.0	Cluster_00307
706.62232	35.0	Cluster_03289
706.62925	35.0	Cluster_03297
706.63126	35.0	Cluster_03300
706.76398	35.0	Cluster_00306
706.86361	35.0	Cluster_03301
708.60835	35.0	Cluster_34818
708.87437	35.0	Cluster_34828
709.28860	35.0	Cluster_34838
709.34977	35.0	Cluster_12378
709.66787	35.0	Cluster_34855
709.81635	35.0	Cluster_34851
710.60612	35.0	Cluster_34899
710.90108	35.0	Cluster_34904
711.83266	35.0	Cluster_03342
711.83663	35.0	Cluster_03347
711.86301	35.0	Cluster_34922
713.27764	35.0	Cluster_34969
713.29080	35.0	Cluster_03356
713.36980	35.0	Cluster_34978

713.86227	35.0	Cluster_03365
713.91951	35.0	Cluster_34998
714.36691	35.0	Cluster_35003
714.82840	35.0	Cluster_35015
714.83014	35.0	Cluster_35026
715.18884	35.0	Cluster_35027
717.16182	35.0	Cluster_35084
717.26810	35.0	Cluster_35093
719.01137	35.0	Cluster_12500
719.31062	35.0	Cluster_35143
719.61357	35.0	Cluster_03408
719.62148	35.0	Cluster_03410
719.86761	35.0	Cluster_35165
720.05737	35.0	Cluster_12523
721.42197	35.0	Cluster_35196
721.86747	35.0	Cluster_03429
721.92070	35.0	Cluster_35214
722.42456	35.0	Cluster_35223
722.60635	35.0	Cluster_03438
722.61381	35.0	Cluster_03439
722.70199	35.0	Cluster_12561
723.69044	35.0	Cluster_35246
723.73145	35.0	Cluster_00325
724.01495	35.0	Cluster_12583
724.63248	35.0	Cluster_35271
727.04057	35.0	Cluster_12624
727.11444	35.0	Cluster_03480
727.21257	35.0	Cluster_35312
727.72402	35.0	Cluster_35325
728.02187	35.0	Cluster_12644
729.13426	35.0	Cluster_35352
729.99899	35.0	Cluster_12666
731.01313	35.0	Cluster_12681
731.86730	35.0	Cluster_35428
732.35032	35.0	Cluster_12702
732.63776	35.0	Cluster_03534
733.11661	35.0	Cluster_03543
733.11760	35.0	Cluster_35455
733.11909	35.0	Cluster_03544
733.12735	35.0	Cluster_03548
733.36842	35.0	Cluster_03556
733.36858	35.0	Cluster_03555
735.35959	35.0	Cluster_35520
736.22851	35.0	Cluster_35536
736.53601	35.0	Cluster_35546
736.85330	35.0	Cluster_03578
737.11647	35.0	Cluster_03579
737.33266	35.0	Cluster_35583
737.69447	35.0	Cluster_12767
738.10935	35.0	Cluster_03586
738.30853	35.0	Cluster_35618

738.33760	35.0	Cluster_12777
738.37561	35.0	Cluster_35615
738.88226	35.0	Cluster_35625
739.32896	35.0	Cluster_12785
740.02039	35.0	Cluster_12796
740.69260	35.0	Cluster_12808
742.33310	35.0	Cluster_35695
742.39069	35.0	Cluster_35694
742.68914	35.0	Cluster_12829
743.35586	35.0	Cluster_35729
744.07867	35.0	Cluster_12846
744.35590	35.0	Cluster_35745
744.42886	35.0	Cluster_35754
744.87374	35.0	Cluster_35760
745.71379	35.0	Cluster_12858
745.87885	35.0	Cluster_35786
746.06574	35.0	Cluster_12867
746.39046	35.0	Cluster_12876
748.06008	35.0	Cluster_12907
748.86103	35.0	Cluster_03654
749.38170	35.0	Cluster_35861
749.39351	35.0	Cluster_35865
749.43777	35.0	Cluster_35870
750.39152	35.0	Cluster_35881
751.69588	35.0	Cluster_12965
751.80275	35.0	Cluster_35915
753.38453	35.0	Cluster_35960
753.78417	35.0	Cluster_35982
754.58916	35.0	Cluster_03681
754.73608	35.0	Cluster_13017
754.80036	35.0	Cluster_35999
755.85015	35.0	Cluster_03685
755.87117	35.0	Cluster_36038
756.39066	35.0	Cluster_36047
756.42287	35.0	Cluster_36046
756.59788	35.0	Cluster_03689
757.07652	35.0	Cluster_13067
757.30855	35.0	Cluster_36071
758.22619	35.0	Cluster_36095
758.73364	35.0	Cluster_36107
759.06890	35.0	Cluster_13093
759.37381	35.0	Cluster_36124
761.02441	35.0	Cluster_13116
761.45335	35.0	Cluster_13117
761.64099	35.0	Cluster_36196
762.05458	35.0	Cluster_13131
762.07253	35.0	Cluster_13132
762.10790	35.0	Cluster_36203
762.14821	35.0	Cluster_03718
762.28681	35.0	Cluster_36205
762.33840	35.0	Cluster_36213

762.34180	35.0	Cluster_13133
762.38228	35.0	Cluster_13130
764.06043	35.0	Cluster_13156
766.08420	35.0	Cluster_03757
766.32954	35.0	Cluster_13190
766.87855	35.0	Cluster_36304
767.16279	35.0	Cluster_03766
768.06607	35.0	Cluster_13209
769.12088	35.0	Cluster_36363
769.40797	35.0	Cluster_36377
769.46550	35.0	Cluster_36384
769.72586	35.0	Cluster_13232
770.69391	35.0	Cluster_13238
771.07220	35.0	Cluster_13246
771.56399	35.0	Cluster_00342
771.88334	35.0	Cluster_03784
771.93573	35.0	Cluster_36427
773.13438	35.0	Cluster_13272
773.87560	35.0	Cluster_03789
774.04780	35.0	Cluster_13292
774.17570	35.0	Cluster_36483
774.37503	35.0	Cluster_13304
774.85555	35.0	Cluster_36496
775.66717	35.0	Cluster_13318
775.87209	35.0	Cluster_36521
777.70948	35.0	Cluster_13338
777.83832	35.0	Cluster_36578
777.84116	35.0	Cluster_36575
778.37272	35.0	Cluster_36591
780.30002	35.0	Cluster_36632
780.74770	35.0	Cluster_13383
781.40025	35.0	Cluster_36661
782.03197	35.0	Cluster_13403
783.12152	35.0	Cluster_03835
783.88454	35.0	Cluster_36719
784.06281	35.0	Cluster_13433
784.14461	35.0	Cluster_03845
784.88961	35.0	Cluster_36733
784.95613	35.0	Cluster_36736
785.02199	35.0	Cluster_13443
785.09844	35.0	Cluster_36737
785.41303	35.0	Cluster_36755
785.61996	35.0	Cluster_03854
785.87805	35.0	Cluster_03853
786.60068	35.0	Cluster_03865
786.82147	35.0	Cluster_36782
787.05983	35.0	Cluster_13459
787.40128	35.0	Cluster_03872
788.36857	35.0	Cluster_36828
788.38543	35.0	Cluster_36836
788.41338	35.0	Cluster_13472

788.70878	35.0	Cluster_13473
788.86008	35.0	Cluster_36852
788.86793	35.0	Cluster_36840
789.11768	35.0	Cluster_03883
789.19890	35.0	Cluster_00345
789.38869	35.0	Cluster_36865
789.61728	35.0	Cluster_03891
789.83948	35.0	Cluster_36869
789.85099	35.0	Cluster_36877
789.86668	35.0	Cluster_36866
790.03748	35.0	Cluster_13497
790.07355	35.0	Cluster_13501
790.34222	35.0	Cluster_36880
791.39206	35.0	Cluster_03901
791.45763	35.0	Cluster_36916
792.14125	35.0	Cluster_36929
792.68615	35.0	Cluster_13537
792.80105	35.0	Cluster_36946
792.96916	35.0	Cluster_13541
793.45015	35.0	Cluster_36966
793.95164	35.0	Cluster_36976
794.13239	35.0	Cluster_36978
794.42894	35.0	Cluster_36983
795.05070	35.0	Cluster_13574
795.77104	35.0	Cluster_37009
796.39992	35.0	Cluster_37032
796.40163	35.0	Cluster_37036
796.88868	35.0	Cluster_03956
796.98818	35.0	Cluster_37058
797.13475	35.0	Cluster_03957
798.35794	35.0	Cluster_13632
798.91924	35.0	Cluster_37110
799.16011	35.0	Cluster_03969
799.90299	35.0	Cluster_37123
799.92816	35.0	Cluster_37119
800.38300	35.0	Cluster_37139
800.39257	35.0	Cluster_37138
800.89786	35.0	Cluster_37148
800.90112	35.0	Cluster_00030
800.91677	35.0	Cluster_03988
800.91942	35.0	Cluster_37153
801.46098	35.0	Cluster_37165
801.70986	35.0	Cluster_13663
801.73389	35.0	Cluster_13658
802.04041	35.0	Cluster_13662
802.36221	35.0	Cluster_03998
802.38466	35.0	Cluster_37185
802.41035	35.0	Cluster_13666
802.42282	35.0	Cluster_37192
802.74372	35.0	Cluster_13681
802.91353	35.0	Cluster_04000

804.33872	35.0	Cluster_13691
804.41270	35.0	Cluster_37237
804.44409	35.0	Cluster_13695
804.90323	35.0	Cluster_37264
804.92086	35.0	Cluster_37265
805.64630	35.0	Cluster_04018
806.00995	35.0	Cluster_37294
806.43863	35.0	Cluster_37309
806.92500	35.0	Cluster_37317
807.14878	35.0	Cluster_04029
807.73159	35.0	Cluster_13735
807.87638	35.0	Cluster_04031
807.89009	35.0	Cluster_37340
807.89822	35.0	Cluster_37345
808.39507	35.0	Cluster_37359
808.84749	35.0	Cluster_37370
808.89580	35.0	Cluster_37373
808.92878	35.0	Cluster_04036
809.05025	35.0	Cluster_13760
809.73734	35.0	Cluster_13764
809.78549	35.0	Cluster_13767
811.43019	35.0	Cluster_37431
811.74034	35.0	Cluster_00036
811.85919	35.0	Cluster_37446
812.10995	35.0	Cluster_13797
812.41776	35.0	Cluster_37457
812.65606	35.0	Cluster_04058
812.85184	35.0	Cluster_37467
813.72689	35.0	Cluster_13823
813.74559	35.0	Cluster_13819
814.00285	35.0	Cluster_37491
814.42062	35.0	Cluster_37495
814.57404	35.0	Cluster_37500
814.74378	35.0	Cluster_13828
814.89596	35.0	Cluster_04074
815.16227	35.0	Cluster_04078
815.68634	35.0	Cluster_13842
815.80355	35.0	Cluster_00353
815.87690	35.0	Cluster_37524
815.87690	35.0	Cluster_37536
815.93641	35.0	Cluster_37532
816.70520	35.0	Cluster_04090
818.38020	35.0	Cluster_37581
818.87672	35.0	Cluster_37589
819.16928	35.0	Cluster_04097
820.08348	35.0	Cluster_13916
820.84883	35.0	Cluster_37627
821.66013	35.0	Cluster_13928
822.07133	35.0	Cluster_13931
823.39219	35.0	Cluster_04123
826.90824	35.0	Cluster_37758

827.36936	35.0	Cluster_37773
827.77245	35.0	Cluster_13990
828.79852	35.0	Cluster_14000
829.43734	35.0	Cluster_37818
829.90674	35.0	Cluster_04169
830.07611	35.0	Cluster_14011
830.40984	35.0	Cluster_37831
831.04351	35.0	Cluster_14024
831.16303	35.0	Cluster_04175
831.37479	35.0	Cluster_37854
832.06644	35.0	Cluster_14030
832.16480	35.0	Cluster_04195
832.16812	35.0	Cluster_04194
833.89799	35.0	Cluster_37898
834.49238	35.0	Cluster_37906
834.79922	35.0	Cluster_14057
834.92208	35.0	Cluster_37924
836.67277	35.0	Cluster_04230
836.75775	35.0	Cluster_14083
836.87714	35.0	Cluster_04232
836.96388	35.0	Cluster_37964
838.42552	35.0	Cluster_37988
839.42310	35.0	Cluster_38013
839.42443	35.0	Cluster_38016
840.38600	35.0	Cluster_38045
840.42961	35.0	Cluster_38043
840.65991	35.0	Cluster_04260
840.93222	35.0	Cluster_38057
842.15556	35.0	Cluster_04278
842.42996	35.0	Cluster_38083
843.17212	35.0	Cluster_38098
843.91630	35.0	Cluster_38116
843.92674	35.0	Cluster_04290
844.40997	35.0	Cluster_04296
845.41720	35.0	Cluster_38145
845.69271	35.0	Cluster_04313
846.16906	35.0	Cluster_04326
846.64883	35.0	Cluster_38171
846.91185	35.0	Cluster_38178
847.87944	35.0	Cluster_38203
847.97354	35.0	Cluster_38213
848.09241	35.0	Cluster_14192
848.42873	35.0	Cluster_14196
848.44349	35.0	Cluster_14193
849.05340	35.0	Cluster_14199
849.66179	35.0	Cluster_04366
849.89533	35.0	Cluster_04373
850.70279	35.0	Cluster_38261
850.93488	35.0	Cluster_04378
851.45581	35.0	Cluster_38292
851.93495	35.0	Cluster_38300

852.36811	35.0	Cluster_38309
852.39296	35.0	Cluster_04398
852.87918	35.0	Cluster_38320
853.91600	35.0	Cluster_38358
853.91840	35.0	Cluster_38362
854.86483	35.0	Cluster_38381
854.89483	35.0	Cluster_38383
855.42613	35.0	Cluster_38394
856.19549	35.0	Cluster_38404
856.42831	35.0	Cluster_14249
857.47441	35.0	Cluster_38452
858.90187	35.0	Cluster_04463
859.48300	35.0	Cluster_38507
859.90011	35.0	Cluster_38518
859.90579	35.0	Cluster_04476
859.94338	35.0	Cluster_38512
860.40068	35.0	Cluster_38529
860.43330	35.0	Cluster_38536
860.86555	35.0	Cluster_00368
861.37862	35.0	Cluster_38563
862.39101	35.0	Cluster_04505
862.89909	35.0	Cluster_04512
862.94643	35.0	Cluster_04515
863.41282	35.0	Cluster_38604
863.91763	35.0	Cluster_38614
863.93693	35.0	Cluster_38615
864.91738	35.0	Cluster_38635
865.65650	35.0	Cluster_04549
865.94266	35.0	Cluster_38656
866.50122	35.0	Cluster_14332
866.64805	35.0	Cluster_04564
866.90274	35.0	Cluster_38675
867.15163	35.0	Cluster_04567
867.42398	35.0	Cluster_14342
867.94135	35.0	Cluster_38687
868.95585	35.0	Cluster_38713
869.39554	35.0	Cluster_38720
869.40591	35.0	Cluster_04599
869.65175	35.0	Cluster_04607
871.15725	35.0	Cluster_04624
871.38291	35.0	Cluster_04626
871.44686	35.0	Cluster_38770
871.48385	35.0	Cluster_14389
871.93492	35.0	Cluster_38790
871.99104	35.0	Cluster_38783
872.21335	35.0	Cluster_38793
872.42942	35.0	Cluster_38804
872.93760	35.0	Cluster_38810
873.02168	35.0	Cluster_38812
873.92713	35.0	Cluster_38826
874.05293	35.0	Cluster_38841

874.91378	35.0	Cluster_04666
874.98369	35.0	Cluster_38855
875.77964	35.0	Cluster_14433
876.13511	35.0	Cluster_04697
876.75188	35.0	Cluster_14443
876.75497	35.0	Cluster_14439
876.75613	35.0	Cluster_14442
876.80258	35.0	Cluster_14444
877.39658	35.0	Cluster_04709
877.44878	35.0	Cluster_14450
877.88623	35.0	Cluster_04717
877.91326	35.0	Cluster_04713
878.14699	35.0	Cluster_04718
879.16542	35.0	Cluster_14474
879.16633	35.0	Cluster_14473
879.82195	35.0	Cluster_14482
879.82480	35.0	Cluster_14476
879.90195	35.0	Cluster_04734
881.79493	35.0	Cluster_14500
881.97445	35.0	Cluster_38983
882.02414	35.0	Cluster_39002
882.08171	35.0	Cluster_14501
882.66243	35.0	Cluster_04748
882.91454	35.0	Cluster_39015
883.40859	35.0	Cluster_04754
883.43072	35.0	Cluster_14515
883.77664	35.0	Cluster_14526
884.44669	35.0	Cluster_04756
885.13034	35.0	Cluster_14552
885.68403	35.0	Cluster_04765
885.69766	35.0	Cluster_04767
885.97171	35.0	Cluster_39076
885.99322	35.0	Cluster_39073
886.12034	35.0	Cluster_14560
887.13762	35.0	Cluster_14569
887.51949	35.0	Cluster_39107
888.09975	35.0	Cluster_14576
888.48577	35.0	Cluster_39122
889.43944	35.0	Cluster_39147
889.95457	35.0	Cluster_39156
892.45399	35.0	Cluster_39210
893.42606	35.0	Cluster_39231
894.93787	35.0	Cluster_39259
894.99352	35.0	Cluster_04854
895.80879	35.0	Cluster_14625
896.76001	35.0	Cluster_14640
896.77212	35.0	Cluster_14637
897.39803	35.0	Cluster_39296
897.48380	35.0	Cluster_14652
897.94487	35.0	Cluster_39306
897.95625	35.0	Cluster_39315

899.10766	35.0	Cluster_14671
899.12716	35.0	Cluster_14674
899.46375	35.0	Cluster_39340
899.92997	35.0	Cluster_39346
900.44745	35.0	Cluster_04893
900.93632	35.0	Cluster_39368
900.96315	35.0	Cluster_39369
901.00269	35.0	Cluster_39384
902.56355	35.0	Cluster_39414
902.56355	35.0	Cluster_39415
902.80635	35.0	Cluster_14691
902.83968	35.0	Cluster_14697
903.09960	35.0	Cluster_14690
903.43465	35.0	Cluster_39426
903.95125	35.0	Cluster_04920
904.40367	35.0	Cluster_39437
904.45901	35.0	Cluster_39443
904.93200	35.0	Cluster_39455
906.97714	35.0	Cluster_39478
907.38470	35.0	Cluster_39484
907.49299	35.0	Cluster_39483
907.96227	35.0	Cluster_39490
907.99865	35.0	Cluster_39496
908.68258	35.0	Cluster_04964
908.85062	35.0	Cluster_00383
908.94704	35.0	Cluster_39504
909.97831	35.0	Cluster_39519
910.12391	35.0	Cluster_39518
910.12524	35.0	Cluster_14768
911.98028	35.0	Cluster_39560
912.42574	35.0	Cluster_14806
912.80213	35.0	Cluster_14805
912.92380	35.0	Cluster_39580
912.92797	35.0	Cluster_39581
912.95093	35.0	Cluster_39585
914.26396	35.0	Cluster_00389
914.45343	35.0	Cluster_14816
915.11024	35.0	Cluster_14822
916.09860	35.0	Cluster_14835
916.44537	35.0	Cluster_39639
916.94625	35.0	Cluster_39657
917.97343	35.0	Cluster_39674
918.44980	35.0	Cluster_39693
918.80128	35.0	Cluster_14871
919.26354	35.0	Cluster_39707
919.47261	35.0	Cluster_14874
920.10752	35.0	Cluster_14882
920.28060	35.0	Cluster_39719
920.62551	35.0	Cluster_00053
920.77045	35.0	Cluster_14885
922.44243	35.0	Cluster_14905

922.96183	35.0	Cluster_39769
923.09415	35.0	Cluster_14931
923.94294	35.0	Cluster_39783
923.98960	35.0	Cluster_39784
924.43536	35.0	Cluster_14953
924.92883	35.0	Cluster_39798
924.96027	35.0	Cluster_39797
926.01701	35.0	Cluster_39810
926.13223	35.0	Cluster_14978
926.45313	35.0	Cluster_14969
926.46599	35.0	Cluster_14973
926.49297	35.0	Cluster_39814
926.49478	35.0	Cluster_14979
927.43778	35.0	Cluster_39836
927.92586	35.0	Cluster_39851
928.44165	35.0	Cluster_39864
928.47669	35.0	Cluster_39862
930.81778	35.0	Cluster_15028
930.92703	35.0	Cluster_05035
930.97959	35.0	Cluster_39916
930.98681	35.0	Cluster_39912
931.41413	35.0	Cluster_39913
931.75879	35.0	Cluster_15046
931.80684	35.0	Cluster_15048
931.89565	35.0	Cluster_39924
932.63768	35.0	Cluster_15062
932.75835	35.0	Cluster_15059
933.92763	35.0	Cluster_39962
934.94244	35.0	Cluster_39977
936.16095	35.0	Cluster_15109
936.48194	35.0	Cluster_39994
936.97952	35.0	Cluster_40008
937.48268	35.0	Cluster_15142
937.71270	35.0	Cluster_40015
937.73608	35.0	Cluster_40014
937.94932	35.0	Cluster_05054
938.37752	35.0	Cluster_40030
938.37914	35.0	Cluster_40031
938.80176	35.0	Cluster_15159
938.94420	35.0	Cluster_40033
938.96404	35.0	Cluster_05057
938.96620	35.0	Cluster_40042
939.15327	35.0	Cluster_15165
939.68688	35.0	Cluster_05058
941.11983	35.0	Cluster_15193
941.98683	35.0	Cluster_40092
942.14657	35.0	Cluster_15209
942.48402	35.0	Cluster_40107
943.92893	35.0	Cluster_40133
943.96400	35.0	Cluster_40118
944.17376	35.0	Cluster_05071

945.38704	35.0	Cluster_15242
945.69833	35.0	Cluster_00409
946.02003	35.0	Cluster_05076
946.96633	35.0	Cluster_05081
946.97997	35.0	Cluster_40166
947.13555	35.0	Cluster_15263
947.51320	35.0	Cluster_15268
948.12920	35.0	Cluster_15278
949.44613	35.0	Cluster_40193
949.47546	35.0	Cluster_15306
949.48378	35.0	Cluster_15297
949.79869	35.0	Cluster_40191
951.98931	35.0	Cluster_40222
952.14699	35.0	Cluster_15339
952.88349	35.0	Cluster_15346
952.89465	35.0	Cluster_00415
954.41272	35.0	Cluster_15371
954.78930	35.0	Cluster_15383
954.80409	35.0	Cluster_15390
954.80629	35.0	Cluster_15384
954.82575	35.0	Cluster_15380
955.47325	35.0	Cluster_15404
955.49162	35.0	Cluster_40268
956.15976	35.0	Cluster_15421
956.44311	35.0	Cluster_15422
956.47325	35.0	Cluster_40279
956.47921	35.0	Cluster_15426
956.80746	35.0	Cluster_15424
956.81074	35.0	Cluster_15425
957.42854	35.0	Cluster_40288
958.70795	35.0	Cluster_05141
960.16356	35.0	Cluster_15469
960.83092	35.0	Cluster_15476
961.21712	35.0	Cluster_40324
961.71471	35.0	Cluster_05152
962.78316	35.0	Cluster_15504
963.14215	35.0	Cluster_15508
964.47449	35.0	Cluster_40369
964.71819	35.0	Cluster_40371
964.98137	35.0	Cluster_05166
965.80056	35.0	Cluster_15544
965.82093	35.0	Cluster_15540
966.92472	35.0	Cluster_40391
968.00687	35.0	Cluster_05185
968.51104	35.0	Cluster_15580
971.14245	35.0	Cluster_15612
971.59682	35.0	Cluster_40462
971.99294	35.0	Cluster_40453
972.00212	35.0	Cluster_40459
972.26876	35.0	Cluster_05201
972.45860	35.0	Cluster_15627

972.48455	35.0	Cluster_15634
973.26231	35.0	Cluster_40484
975.16800	35.0	Cluster_15670
977.10216	35.0	Cluster_15692
977.15613	35.0	Cluster_15689
978.75156	35.0	Cluster_05224
979.02301	35.0	Cluster_40541
979.49673	35.0	Cluster_15713
980.13256	35.0	Cluster_15723
981.41397	35.0	Cluster_40580
981.44889	35.0	Cluster_15750
981.45712	35.0	Cluster_15741
981.45924	35.0	Cluster_15747
982.13387	35.0	Cluster_15752
983.47704	35.0	Cluster_40610
984.15760	35.0	Cluster_15784
985.95900	35.0	Cluster_40633
986.82341	35.0	Cluster_15809
987.47846	35.0	Cluster_40647
988.49825	35.0	Cluster_15833
988.94776	35.0	Cluster_40676
990.42795	35.0	Cluster_40698
990.48099	35.0	Cluster_15858
991.15972	35.0	Cluster_15864
991.47567	35.0	Cluster_15867
991.97924	35.0	Cluster_05270
992.48241	35.0	Cluster_40726
992.74985	35.0	Cluster_05272
992.82102	35.0	Cluster_15885
993.01345	35.0	Cluster_40733
994.14517	35.0	Cluster_15902
996.00812	35.0	Cluster_40774
996.70093	35.0	Cluster_40783
996.86309	35.0	Cluster_15945
997.76759	35.0	Cluster_05282
997.96271	35.0	Cluster_40814
998.87343	35.0	Cluster_15967
998.87590	35.0	Cluster_15968
999.99081	35.0	Cluster_40861
1001.02474	35.0	Cluster_40870
1003.12198	35.0	Cluster_16013
1003.14396	35.0	Cluster_16019
1003.15815	35.0	Cluster_16017
1003.53342	35.0	Cluster_40926
1006.19509	35.0	Cluster_16058
1007.01591	35.0	Cluster_40963
1007.54887	35.0	Cluster_40979
1007.80918	35.0	Cluster_16072
1007.96099	35.0	Cluster_40980
1008.19150	35.0	Cluster_16075
1009.53477	35.0	Cluster_41016

1009.82372	35.0	Cluster_16100
1012.00753	35.0	Cluster_05327
1013.18893	35.0	Cluster_16139
1013.46270	35.0	Cluster_16144
1014.19342	35.0	Cluster_16147
1014.50348	35.0	Cluster_41066
1015.75807	35.0	Cluster_05350
1015.79694	35.0	Cluster_16185
1015.91838	35.0	Cluster_41088
1016.17395	35.0	Cluster_16182
1016.48392	35.0	Cluster_16187
1016.78489	35.0	Cluster_05351
1017.91335	35.0	Cluster_41107
1018.01928	35.0	Cluster_41119
1018.03014	35.0	Cluster_41116
1018.15185	35.0	Cluster_16199
1018.27963	35.0	Cluster_41121
1022.17658	35.0	Cluster_16227
1022.18316	35.0	Cluster_16229
1022.49717	35.0	Cluster_16231
1023.01121	35.0	Cluster_41172
1023.18114	35.0	Cluster_16239
1023.19682	35.0	Cluster_16242
1024.50414	35.0	Cluster_16256
1024.92184	35.0	Cluster_41190
1026.15295	35.0	Cluster_16265
1027.88031	35.0	Cluster_16291
1028.00760	35.0	Cluster_05398
1028.51822	35.0	Cluster_16304
1028.55896	35.0	Cluster_41243
1028.82739	35.0	Cluster_16303
1030.05344	35.0	Cluster_41261
1030.51311	35.0	Cluster_16316
1030.83122	35.0	Cluster_16315
1031.52220	35.0	Cluster_41282
1031.95890	35.0	Cluster_41279
1032.06454	35.0	Cluster_41290
1034.50686	35.0	Cluster_41326
1035.51947	35.0	Cluster_16366
1035.52766	35.0	Cluster_16368
1035.88293	35.0	Cluster_16378
1038.04978	35.0	Cluster_41356
1038.52523	35.0	Cluster_41369
1039.11544	35.0	Cluster_41372
1039.52126	35.0	Cluster_41374
1039.55516	35.0	Cluster_41383
1041.01058	35.0	Cluster_41401
1041.85584	35.0	Cluster_16451
1042.51021	35.0	Cluster_41413
1042.53113	35.0	Cluster_16458
1045.27743	35.0	Cluster_05445

1046.18482	35.0	Cluster_16494
1046.22057	35.0	Cluster_16495
1046.52458	35.0	Cluster_16503
1048.00284	35.0	Cluster_41465
1048.97425	35.0	Cluster_41478
1050.51799	35.0	Cluster_16549
1052.08579	35.0	Cluster_41500
1053.27199	35.0	Cluster_41517
1053.47816	35.0	Cluster_41529
1053.51090	35.0	Cluster_16579
1053.87018	35.0	Cluster_16587
1054.86259	35.0	Cluster_16590
1054.86458	35.0	Cluster_16593
1055.04277	35.0	Cluster_41552
1055.21811	35.0	Cluster_16595
1055.52224	35.0	Cluster_16604
1056.84213	35.0	Cluster_16627
1056.84310	35.0	Cluster_16621
1056.86273	35.0	Cluster_16631
1056.86682	35.0	Cluster_16628
1056.98835	35.0	Cluster_41572
1057.48459	35.0	Cluster_41573
1057.99718	35.0	Cluster_41576
1058.07318	35.0	Cluster_41590
1058.82604	35.0	Cluster_16656
1059.48627	35.0	Cluster_41600
1061.05662	35.0	Cluster_05509
1062.01230	35.0	Cluster_41632
1062.51278	35.0	Cluster_41641
1062.53761	35.0	Cluster_41640
1062.94023	35.0	Cluster_41638
1063.51637	35.0	Cluster_16710
1064.19367	35.0	Cluster_16719
1064.20364	35.0	Cluster_16713
1064.26120	35.0	Cluster_41669
1064.52753	35.0	Cluster_41674
1064.82145	35.0	Cluster_16722
1065.19208	35.0	Cluster_16729
1065.54336	35.0	Cluster_16738
1065.55172	35.0	Cluster_16733
1065.88246	35.0	Cluster_16740
1065.95736	35.0	Cluster_00582
1066.21797	35.0	Cluster_16744
1066.84581	35.0	Cluster_16751
1068.74086	35.0	Cluster_41735
1068.74952	35.0	Cluster_41734
1069.06383	35.0	Cluster_41739
1069.87675	35.0	Cluster_16777
1070.03189	35.0	Cluster_41745
1070.11798	35.0	Cluster_41751
1070.86551	35.0	Cluster_16790

1073.53750	35.0	Cluster_41789
1074.52560	35.0	Cluster_16830
1075.19675	35.0	Cluster_16840
1077.55035	35.0	Cluster_16862
1077.55228	35.0	Cluster_16863
1079.08133	35.0	Cluster_41847
1079.58373	35.0	Cluster_41846
1080.99907	35.0	Cluster_41855
1081.04145	35.0	Cluster_41859
1083.50260	35.0	Cluster_05592
1083.60866	35.0	Cluster_41894
1085.05222	35.0	Cluster_41919
1086.49351	35.0	Cluster_41934
1087.03798	35.0	Cluster_41945
1089.06441	35.0	Cluster_41967
1093.87964	35.0	Cluster_17034
1098.22930	35.0	Cluster_17071
1098.80067	35.0	Cluster_42074
1098.88169	35.0	Cluster_17084
1099.13859	35.0	Cluster_00634
1103.12066	35.0	Cluster_42104
1103.50753	35.0	Cluster_42106
1103.54965	35.0	Cluster_00644
1105.53482	35.0	Cluster_17143
1105.55729	35.0	Cluster_17144
1108.17135	35.0	Cluster_17167
1108.90668	35.0	Cluster_17181
1111.53648	35.0	Cluster_17220
1113.59218	35.0	Cluster_17234
1113.60048	35.0	Cluster_17235
1114.57480	35.0	Cluster_17251
1116.59135	35.0	Cluster_42204
1117.22661	35.0	Cluster_17271
1118.01598	35.0	Cluster_42221
1118.08766	35.0	Cluster_42224
1118.87403	35.0	Cluster_17290
1121.01097	35.0	Cluster_42253
1121.03337	35.0	Cluster_42254
1122.55016	35.0	Cluster_17335
1122.86660	35.0	Cluster_17332
1123.53963	35.0	Cluster_17344
1123.97198	35.0	Cluster_42271
1124.00796	35.0	Cluster_42274
1124.51758	35.0	Cluster_42279
1124.52443	35.0	Cluster_05696
1124.52521	35.0	Cluster_17361
1125.56818	35.0	Cluster_42283
1127.87407	35.0	Cluster_17396
1129.16094	35.0	Cluster_17421
1129.57408	35.0	Cluster_42318
1130.53740	35.0	Cluster_42327

1132.22675	35.0	Cluster_17466
1136.05741	35.0	Cluster_42359
1137.19776	35.0	Cluster_17532
1137.21620	35.0	Cluster_17536
1137.22189	35.0	Cluster_17533
1137.22952	35.0	Cluster_17529
1138.87058	35.0	Cluster_17553
1139.87279	35.0	Cluster_17566
1139.92314	35.0	Cluster_17563
1140.20245	35.0	Cluster_17575
1141.54454	35.0	Cluster_17592
1141.77200	35.0	Cluster_00673
1141.89056	35.0	Cluster_17593
1143.03536	35.0	Cluster_42405
1143.25402	35.0	Cluster_17604
1143.54127	35.0	Cluster_17610
1144.55115	35.0	Cluster_17624
1144.55511	35.0	Cluster_17632
1144.86522	35.0	Cluster_17636
1145.57649	35.0	Cluster_05763
1147.10296	35.0	Cluster_42440
1147.85107	35.0	Cluster_17678
1150.20248	35.0	Cluster_17718
1151.04837	35.0	Cluster_42474
1151.06782	35.0	Cluster_42470
1152.20245	35.0	Cluster_17734
1152.54176	35.0	Cluster_17735
1152.57782	35.0	Cluster_17744
1153.03400	35.0	Cluster_05790
1153.87912	35.0	Cluster_17753
1153.88976	35.0	Cluster_17763
1155.08158	35.0	Cluster_42492
1155.24348	35.0	Cluster_17775
1155.59409	35.0	Cluster_17779
1155.89824	35.0	Cluster_17801
1156.88520	35.0	Cluster_17793
1161.58506	35.0	Cluster_42549
1163.55909	35.0	Cluster_17884
1166.21448	35.0	Cluster_17894
1168.08892	35.0	Cluster_42606
1168.58026	35.0	Cluster_05832
1172.61481	35.0	Cluster_17968
1173.06914	35.0	Cluster_42645
1176.56192	35.0	Cluster_42661
1177.58775	35.0	Cluster_42668
1177.59766	35.0	Cluster_42670
1178.92015	35.0	Cluster_18022
1182.12534	35.0	Cluster_42707
1182.58890	35.0	Cluster_18037
1183.23440	35.0	Cluster_18040
1183.54983	35.0	Cluster_18043

1184.10037	35.0	Cluster_42721
1187.11937	35.0	Cluster_05925
1187.49288	35.0	Cluster_42749
1187.51857	35.0	Cluster_42746
1187.59216	35.0	Cluster_18075
1187.92731	35.0	Cluster_18077
1187.92845	35.0	Cluster_18076
1190.56370	35.0	Cluster_18091
1192.59067	35.0	Cluster_18107
1195.88339	35.0	Cluster_18133
1198.06720	35.0	Cluster_42794
1198.63811	35.0	Cluster_42803
1200.25888	35.0	Cluster_18168
1200.52867	35.0	Cluster_18173
1200.56319	35.0	Cluster_18177
1202.23828	35.0	Cluster_18187
1202.23877	35.0	Cluster_18194
1202.24327	35.0	Cluster_18188
1204.59157	35.0	Cluster_18204
1205.52768	35.0	Cluster_42840
1206.89327	35.0	Cluster_18228
1208.38799	35.0	Cluster_42881
1208.56000	35.0	Cluster_18237
1209.25140	35.0	Cluster_18244
1214.21516	35.0	Cluster_18272
1219.90840	35.0	Cluster_18297
1221.61327	35.0	Cluster_42961
1222.91304	35.0	Cluster_18319
1224.59870	35.0	Cluster_42983
1229.56319	35.0	Cluster_43017
1230.62662	35.0	Cluster_43026
1232.60076	35.0	Cluster_43040
1233.11912	35.0	Cluster_43048
1235.08055	35.0	Cluster_43061
1235.57065	35.0	Cluster_06173
1242.32292	35.0	Cluster_18386
1242.56480	35.0	Cluster_18385
1244.94841	35.0	Cluster_18392
1248.07817	35.0	Cluster_43132
1248.07817	35.0	Cluster_43134
1249.82369	35.0	Cluster_06249
1256.58258	35.0	Cluster_06280
1256.84647	35.0	Cluster_43180
1265.60966	35.0	Cluster_43219
1267.20414	35.0	Cluster_43224
1281.84230	35.0	Cluster_06351
1287.13293	35.0	Cluster_43302
1297.34487	35.0	Cluster_18563
1297.57738	35.0	Cluster_43344
1303.91742	35.0	Cluster_06419
1309.16699	35.0	Cluster_06433

1323.96017	35.0	Cluster_06474
1324.59971	35.0	Cluster_43461
1333.16281	35.0	Cluster_43496
1343.21645	35.0	Cluster_43538
1353.42347	35.0	Cluster_06570
1356.69696	35.0	Cluster_43600
1356.70119	35.0	Cluster_43599
1358.65593	35.0	Cluster_06587
1358.66525	35.0	Cluster_43610
1371.69886	35.0	Cluster_43665
1372.75349	35.0	Cluster_43667
1375.22316	35.0	Cluster_43678
1382.71602	35.0	Cluster_43695
1383.64043	35.0	Cluster_43699
1404.23437	35.0	Cluster_43777
1429.02179	35.0	Cluster_18795
1442.73652	35.0	Cluster_43921
1445.24833	35.0	Cluster_43930
1464.40330	35.0	Cluster_18820
1470.73553	35.0	Cluster_43998
1473.26210	35.0	Cluster_44004
1477.30369	35.0	Cluster_44015
1478.73704	35.0	Cluster_44019
1530.32978	35.0	Cluster_44125
1564.32207	35.0	Cluster_44184
1571.75675	35.0	Cluster_44194
1616.85500	35.0	Cluster_44250
1655.34019	35.0	Cluster_44282
1689.84084	35.0	Cluster_44356
1700.84338	35.0	Cluster_44377
1739.94490	35.0	Cluster_44403

Reject Mass List: (none)
Neutral Loss Mass List: (none)
Product Mass List: (none)
Neutral loss in top: 3
Product in top: 3
Most intense if no parent masses found not enabled
Add/subtract mass not enabled
FT master scan preview mode enabled
Charge state screening enabled
Charge state dependent ETD time not enabled
Monoisotopic precursor selection enabled
Charge state rejection enabled
Unassigned charge states : rejected
Charge state 1 : rejected
Charge state 2 : not rejected
Charge state 3 : not rejected
Charge states 4+ : not rejected
Chromatography mode is disabled
Global Data Dependent Settings:
Predict ion injection time enabled

Use global parent and reject mass lists not enabled
Exclude parent mass from data dependent selection not enabled
Exclusion mass width by mass
Exclusion mass width low: 1.50000
Exclusion mass width high: 1.50000
Parent mass width by mass
Parent mass width low: 0.50000
Parent mass width high: 0.50000
Reject mass width by mass
Reject mass width low: 0.50000
Reject mass width high: 0.50000
Zoom/UltraZoom scan mass width by mass
Zoom/UltraZoom scan mass width low: 5.00
Zoom/UltraZoom scan mass width high: 5.00
FT SIM scan mass width low: 5.00
FT SIM scan mass width high: 5.00
Neutral Loss candidates processed by decreasing intensity
Neutral Loss mass width by mass
Neutral Loss mass width low: 0.50000
Neutral Loss mass width high: 0.50000
Product candidates processed by decreasing intensity
Product mass width by mass
Product mass width low: 0.50000
Product mass width high: 0.50000
MS mass range: 300.00-2000.00
MSn mass range by mass
MSn mass range: 0.00-1000000.00
Use m/z values as masses not enabled
Analog UV data dep. not enabled
Dynamic exclusion enabled
Repeat Count: 1
Repeat Duration: 30.00
Exclusion List Size: 500
Exclusion Duration: 180.00
Exclusion mass width by mass
Exclusion mass width low: 1.50000
Exclusion mass width high: 1.50000
Expiration: disabled
Isotopic data dependence not enabled
Custom Data Dependent Settings:
Not enabled

Pass 7 (wheat-mixed-digests_FTMS1-ITMS2-only_inclusion-RT-01.raw):

Creator: Orbi_30393
Last modified: 10/20/2021 by Orbi_30393
MS Run Time (min): 43.00
Sequence override of method parameters not enabled.
Divert Valve: not used during run
Contact Closure: not used during run
Syringe Pump: not used during run

MS Detector Settings:
Real-time modifications to method not enabled
Stepped collision energy not enabled
Additional Microscans:

MS2	0	0
MS3	0	0
MS4	0	0
MS5	0	0
MS6	0	0
MS7	0	0
MS8	0	0
MS9	0	0
MS10	0	0

Segment 1 Information
Duration (min): 43.00
Number of Scan Events: 2
Tune Method: Orbitrap-tune-file_2020-03-13_HESI
Scan Event Details:
1: FTMS + p norm res=15000 o(300.0-2000.0)
CV = 0.0V
2: ITMS + c norm ·(list)->o(auto range)
Using global MS/MS mass lists
MS/MS: AT CID CE 35.0% Q 0.250 Time 10.000 IsoW 1.0
CV = 0.0V
Lock Masses:
Pos List Name: N/A
Source: API Source
Mass List: (none)
Neg List Name: N/A
Source: API Source
Mass List: (none)
Global Non-Data Dependent Settings:
(+) Global MS/MS Masses:

MS Mass	Start (min)	End (min)	MS FAIMS CV	MS Normalized Collision Energy	Last Mass	Name
300.13000	27.890	28.060		35.0		Cluster_06844
300.14000	1.590	2.170		35.0		Cluster_18928
300.71000	7.520	8.290		35.0		Cluster_18955
301.50000	1.580	2.120		35.0		Cluster_06851
301.65000	11.650	12.150		35.0		Cluster_18984
302.64000	1.240	1.560		35.0		Cluster_19016
302.65000	1.560	2.040		35.0		Cluster_19017
303.99000	1.650	2.110		35.0		Cluster_19040
304.11000	17.480	17.640		35.0		Cluster_19042
304.14000	1.280	1.580		35.0		Cluster_19049
304.16000	1.560	2.030		35.0		Cluster_19050
304.20000	7.520	8.260		35.0		Cluster_19052
304.49000	7.390	7.980		35.0		Cluster_06863
304.64000	1.270	1.570		35.0		Cluster_19062

306.13000	7.630	8.420	35.0	Cluster_06873
306.67000	11.740	12.080	35.0	Cluster_19109
307.12000	19.890	20.520	35.0	Cluster_19119
307.13000	7.640	8.390	35.0	Cluster_06880
308.65000	1.480	1.910	35.0	Cluster_19177
308.66000	7.530	8.280	35.0	Cluster_19179
309.53000	7.580	8.060	35.0	Cluster_06901
310.58000	17.390	18.040	35.0	Cluster_19217
310.65000	7.630	7.900	35.0	Cluster_19222
310.66000	1.580	2.020	35.0	Cluster_19221
311.17000	7.570	8.330	35.0	Cluster_19246
311.68000	7.770	8.930	35.0	Cluster_19257
313.65000	1.240	1.630	35.0	Cluster_19315
314.82000	1.460	1.880	35.0	Cluster_06941
315.65000	1.540	2.000	35.0	Cluster_19384
316.69000	11.690	12.070	35.0	Cluster_19442
317.06000	11.640	11.860	35.0	Cluster_19459
317.16000	1.470	2.010	35.0	Cluster_19466
317.64000	14.650	15.060	35.0	Cluster_19490
318.64000	1.530	1.960	35.0	Cluster_19532
318.85000	7.830	9.360	35.0	Cluster_06973
319.17000	1.260	1.540	35.0	Cluster_19552
319.67000	1.530	2.040	35.0	Cluster_19560
319.94000	7.730	8.090	35.0	Cluster_00850
320.49000	1.510	2.130	35.0	Cluster_06993
320.80000	11.560	12.030	35.0	Cluster_06994
321.15000	1.560	1.880	35.0	Cluster_06995
321.17000	14.840	15.780	35.0	Cluster_00869
321.52000	7.600	8.110	35.0	Cluster_19594
322.15000	19.880	20.020	35.0	Cluster_19619
322.17000	1.490	1.910	35.0	Cluster_19617
322.50000	1.590	2.130	35.0	Cluster_06998
323.65000	1.230	1.590	35.0	Cluster_19672
323.69000	19.910	20.420	35.0	Cluster_19677
323.83000	7.450	7.800	35.0	Cluster_19683
324.42000	11.820	12.680	35.0	Cluster_00878
324.60000	11.610	11.830	35.0	Cluster_19711
324.65000	1.530	1.960	35.0	Cluster_19713
324.83000	11.690	12.010	35.0	Cluster_07017
325.14000	7.400	7.970	35.0	Cluster_19730
325.82000	1.570	2.110	35.0	Cluster_07023
325.85000	11.630	12.670	35.0	Cluster_07028
326.17000	7.780	8.310	35.0	Cluster_19753
326.40000	1.640	2.050	35.0	Cluster_00887
326.90000	7.400	7.730	35.0	Cluster_00888
327.21000	14.670	15.080	35.0	Cluster_19791
327.96000	7.700	8.060	35.0	Cluster_07043
328.48000	1.240	1.570	35.0	Cluster_07051
329.18000	1.520	2.030	35.0	Cluster_19855
329.82000	11.770	12.160	35.0	Cluster_07063
329.93000	1.230	1.630	35.0	Cluster_19873

330.18000	14.850	15.020	35.0	Cluster_19886
330.90000	1.650	2.040	35.0	Cluster_19916
331.17000	1.560	2.070	35.0	Cluster_19926
331.20000	7.320	7.700	35.0	Cluster_19928
331.21000	7.550	7.760	35.0	Cluster_19930
331.22000	17.390	17.790	35.0	Cluster_19925
331.66000	1.240	1.580	35.0	Cluster_19936
332.14000	1.450	1.820	35.0	Cluster_19948
333.47000	1.650	2.010	35.0	Cluster_07081
333.82000	17.380	17.810	35.0	Cluster_07087
333.87000	7.640	8.170	35.0	Cluster_07090
335.12000	7.740	8.570	35.0	Cluster_20034
335.83000	1.230	1.640	35.0	Cluster_07104
336.19000	7.470	7.920	35.0	Cluster_07109
336.23000	11.790	12.070	35.0	Cluster_20093
336.91000	7.390	7.630	35.0	Cluster_00920
337.19000	19.960	20.100	35.0	Cluster_20132
337.21000	11.720	12.080	35.0	Cluster_20126
337.83000	11.850	12.060	35.0	Cluster_07126
338.11000	7.370	7.610	35.0	Cluster_20150
338.67000	7.620	8.030	35.0	Cluster_20172
339.16000	1.450	1.910	35.0	Cluster_20192
339.47000	1.440	2.030	35.0	Cluster_07139
339.81000	14.710	15.090	35.0	Cluster_07150
339.94000	7.660	8.070	35.0	Cluster_00929
340.18000	19.890	20.250	35.0	Cluster_20223
340.26000	22.630	22.970	35.0	Cluster_20229
341.15000	1.270	1.550	35.0	Cluster_20257
341.16000	1.650	2.020	35.0	Cluster_20261
342.16000	1.620	2.020	35.0	Cluster_07167
343.11000	7.800	8.560	35.0	Cluster_20316
343.18000	7.750	8.420	35.0	Cluster_20318
343.66000	1.250	1.550	35.0	Cluster_20344
345.19000	14.850	15.320	35.0	Cluster_20390
345.67000	1.450	1.780	35.0	Cluster_20402
346.15000	17.420	18.290	35.0	Cluster_07199
346.67000	7.760	8.250	35.0	Cluster_20444
347.20000	7.310	7.800	35.0	Cluster_20457
347.71000	7.760	8.410	35.0	Cluster_20471
348.44000	19.970	20.260	35.0	Cluster_20495
348.87000	7.420	7.900	35.0	Cluster_07234
348.92000	14.640	14.870	35.0	Cluster_00975
348.93000	14.860	15.120	35.0	Cluster_00976
349.20000	7.770	8.090	35.0	Cluster_20517
349.21000	11.780	12.250	35.0	Cluster_20520
349.64000	11.600	11.900	35.0	Cluster_20543
349.73000	11.610	11.990	35.0	Cluster_20549
350.42000	14.810	15.540	35.0	Cluster_00982
351.18000	1.240	1.560	35.0	Cluster_20611
351.19000	17.520	17.990	35.0	Cluster_20622
352.66000	11.800	12.220	35.0	Cluster_20669

353.17000	14.630	15.020	35.0	Cluster_01002
353.21000	7.750	8.140	35.0	Cluster_20705
353.66000	1.540	2.210	35.0	Cluster_01003
353.80000	19.900	20.390	35.0	Cluster_07278
353.87000	7.510	7.740	35.0	Cluster_20720
353.91000	1.610	2.100	35.0	Cluster_01004
354.16000	1.610	2.040	35.0	Cluster_20732
354.20000	11.790	12.210	35.0	Cluster_20739
354.40000	7.680	8.720	35.0	Cluster_01009
354.42000	11.860	12.480	35.0	Cluster_01011
355.14000	1.230	1.620	35.0	Cluster_20781
355.16000	1.450	1.850	35.0	Cluster_20782
355.22000	7.500	7.920	35.0	Cluster_20787
355.36000	1.330	1.600	35.0	Cluster_20789
355.70000	1.440	2.070	35.0	Cluster_20796
356.24000	14.870	15.320	35.0	Cluster_20824
356.55000	14.880	16.740	35.0	Cluster_07307
358.42000	1.590	2.110	35.0	Cluster_20933
359.13000	19.930	20.340	35.0	Cluster_07326
359.17000	11.670	12.360	35.0	Cluster_00118
359.37000	11.560	12.150	35.0	Cluster_20960
360.16000	1.260	1.450	35.0	Cluster_20986
360.42000	11.840	12.330	35.0	Cluster_01047
360.83000	7.800	8.000	35.0	Cluster_21008
361.18000	11.800	12.350	35.0	Cluster_21024
361.42000	7.620	8.490	35.0	Cluster_01055
361.66000	1.430	2.060	35.0	Cluster_21038
361.67000	11.800	12.310	35.0	Cluster_21036
361.69000	7.390	7.940	35.0	Cluster_21039
361.84000	7.750	8.230	35.0	Cluster_07347
362.10000	1.550	1.980	35.0	Cluster_21048
362.42000	17.390	17.620	35.0	Cluster_01059
362.43000	7.800	8.230	35.0	Cluster_21075
362.60000	1.520	2.040	35.0	Cluster_21077
362.65000	1.280	1.550	35.0	Cluster_21076
363.11000	1.580	2.030	35.0	Cluster_21089
363.19000	11.880	12.080	35.0	Cluster_21093
363.20000	7.390	8.120	35.0	Cluster_21094
363.77000	17.520	17.890	35.0	Cluster_21121
364.16000	11.580	12.110	35.0	Cluster_21135
364.50000	1.550	1.900	35.0	Cluster_21161
364.65000	19.910	20.370	35.0	Cluster_21162
365.24000	17.500	17.690	35.0	Cluster_21221
365.79000	11.730	12.200	35.0	Cluster_07396
365.90000	1.310	1.630	35.0	Cluster_21241
366.27000	7.710	8.040	35.0	Cluster_21267
366.74000	11.800	12.060	35.0	Cluster_21285
367.22000	14.610	15.000	35.0	Cluster_21306
367.50000	11.840	12.340	35.0	Cluster_07416
367.53000	11.850	12.350	35.0	Cluster_21324
368.16000	11.830	12.240	35.0	Cluster_21368

368.20000	7.600	8.110	35.0	Cluster_21366
368.47000	7.520	8.860	35.0	Cluster_07428
369.67000	1.240	1.570	35.0	Cluster_21437
370.08000	1.590	1.940	35.0	Cluster_21453
370.50000	11.840	12.340	35.0	Cluster_07445
370.74000	22.670	23.120	35.0	Cluster_21476
370.93000	14.750	14.960	35.0	Cluster_21486
371.18000	1.570	1.930	35.0	Cluster_07449
371.24000	11.560	11.900	35.0	Cluster_21501
371.71000	17.530	17.830	35.0	Cluster_21517
371.89000	1.270	1.470	35.0	Cluster_21531
372.17000	7.580	7.970	35.0	Cluster_21537
372.23000	11.560	12.260	35.0	Cluster_07464
372.67000	20.000	20.220	35.0	Cluster_21575
372.69000	1.240	1.640	35.0	Cluster_21572
373.12000	1.600	1.800	35.0	Cluster_21589
373.14000	20.040	20.290	35.0	Cluster_21585
373.16000	14.870	15.050	35.0	Cluster_07470
373.19000	7.420	8.060	35.0	Cluster_07471
373.22000	22.670	23.130	35.0	Cluster_21597
373.90000	14.600	14.750	35.0	Cluster_21628
374.19000	7.480	8.090	35.0	Cluster_07480
374.40000	1.250	1.670	35.0	Cluster_21650
374.68000	14.750	15.180	35.0	Cluster_21656
375.15000	1.650	2.000	35.0	Cluster_21668
375.89000	17.460	17.840	35.0	Cluster_07496
377.48000	14.870	15.440	35.0	Cluster_07512
377.77000	14.780	14.930	35.0	Cluster_21780
378.17000	11.710	12.170	35.0	Cluster_01122
378.18000	1.230	1.600	35.0	Cluster_21788
378.20000	11.840	12.330	35.0	Cluster_07523
378.67000	14.750	14.950	35.0	Cluster_01125
379.26000	14.750	15.010	35.0	Cluster_21841
380.45000	7.770	8.430	35.0	Cluster_21886
380.68000	14.810	15.050	35.0	Cluster_21891
381.22000	17.270	17.840	35.0	Cluster_07550
381.70000	7.800	8.490	35.0	Cluster_01141
382.18000	1.540	1.730	35.0	Cluster_21953
382.66000	14.630	15.040	35.0	Cluster_01144
383.63000	19.940	20.250	35.0	Cluster_22024
383.68000	7.470	8.030	35.0	Cluster_22025
384.16000	14.730	15.060	35.0	Cluster_01146
384.21000	22.650	22.920	35.0	Cluster_22055
384.22000	7.530	7.780	35.0	Cluster_22065
385.52000	22.480	22.910	35.0	Cluster_07597
385.54000	31.410	31.800	35.0	Cluster_07596
385.86000	27.960	28.270	35.0	Cluster_22123
386.66000	20.010	20.720	35.0	Cluster_22155
386.68000	14.620	14.820	35.0	Cluster_22158
386.90000	14.610	14.880	35.0	Cluster_22172
387.11000	11.820	12.000	35.0	Cluster_22177

387.63000	17.350	17.770	35.0	Cluster_22217
387.93000	7.700	8.280	35.0	Cluster_22226
388.23000	11.660	12.060	35.0	Cluster_22233
388.50000	11.630	12.080	35.0	Cluster_07621
388.64000	14.810	15.110	35.0	Cluster_22244
388.67000	19.930	20.200	35.0	Cluster_22245
388.83000	22.620	22.930	35.0	Cluster_07623
389.25000	17.510	18.000	35.0	Cluster_22269
389.51000	14.810	15.110	35.0	Cluster_07627
389.91000	14.830	14.940	35.0	Cluster_22284
390.71000	14.680	15.090	35.0	Cluster_22316
390.87000	31.450	31.800	35.0	Cluster_07651
390.88000	7.800	9.140	35.0	Cluster_07646
391.50000	1.450	2.120	35.0	Cluster_07654
391.65000	19.880	20.230	35.0	Cluster_22334
391.75000	11.860	12.080	35.0	Cluster_22344
391.82000	7.470	7.980	35.0	Cluster_07658
392.22000	14.780	15.190	35.0	Cluster_07665
392.25000	7.750	8.230	35.0	Cluster_22358
392.73000	7.380	7.780	35.0	Cluster_22381
392.77000	11.560	11.910	35.0	Cluster_22377
393.19000	14.820	15.160	35.0	Cluster_01195
393.53000	11.840	12.050	35.0	Cluster_07674
393.69000	14.830	15.060	35.0	Cluster_01197
393.71000	1.590	2.000	35.0	Cluster_22428
393.98000	17.500	17.660	35.0	Cluster_22479
394.21000	25.240	25.400	35.0	Cluster_22490
394.71000	14.870	15.050	35.0	Cluster_22522
394.72000	11.850	11.980	35.0	Cluster_22521
395.89000	11.840	12.350	35.0	Cluster_07703
396.23000	19.970	20.450	35.0	Cluster_22589
396.30000	22.510	22.690	35.0	Cluster_22595
397.39000	25.090	26.350	35.0	Cluster_22628
397.67000	1.530	1.910	35.0	Cluster_22634
397.72000	7.580	8.070	35.0	Cluster_22641
397.90000	14.840	15.260	35.0	Cluster_07736
398.18000	17.310	17.770	35.0	Cluster_01226
398.44000	17.280	17.770	35.0	Cluster_01230
400.24000	14.690	14.900	35.0	Cluster_22747
400.68000	22.560	22.770	35.0	Cluster_22780
400.90000	20.030	20.230	35.0	Cluster_07771
401.70000	7.350	7.800	35.0	Cluster_22833
402.32000	7.400	7.580	35.0	Cluster_22866
402.48000	11.860	12.560	35.0	Cluster_22868
402.72000	11.830	11.990	35.0	Cluster_22878
403.44000	1.560	1.910	35.0	Cluster_01260
403.54000	28.070	28.440	35.0	Cluster_07797
404.47000	14.760	15.050	35.0	Cluster_01272
405.88000	25.070	25.350	35.0	Cluster_07831
406.22000	22.600	23.040	35.0	Cluster_23023
406.68000	1.270	1.540	35.0	Cluster_01285

406.73000	20.010	20.210	35.0	Cluster_23046
406.95000	1.450	2.070	35.0	Cluster_01293
407.17000	7.360	7.710	35.0	Cluster_23059
407.25000	11.740	12.110	35.0	Cluster_23066
407.67000	11.640	12.000	35.0	Cluster_01302
407.75000	14.850	15.150	35.0	Cluster_23083
407.93000	1.570	2.110	35.0	Cluster_01303
408.22000	14.810	14.990	35.0	Cluster_23104
408.54000	11.800	12.160	35.0	Cluster_07863
408.66000	17.290	17.790	35.0	Cluster_01305
408.91000	7.750	8.310	35.0	Cluster_07864
408.92000	20.020	20.430	35.0	Cluster_01307
409.19000	1.560	1.970	35.0	Cluster_07867
409.25000	11.580	11.910	35.0	Cluster_23160
409.69000	1.240	1.530	35.0	Cluster_23170
410.53000	17.390	17.810	35.0	Cluster_07882
411.72000	11.630	12.000	35.0	Cluster_23258
412.67000	1.440	1.970	35.0	Cluster_23299
412.68000	11.850	12.330	35.0	Cluster_01326
413.23000	11.660	11.900	35.0	Cluster_23320
413.87000	1.590	2.080	35.0	Cluster_07913
413.92000	19.920	20.450	35.0	Cluster_07916
413.94000	17.500	17.750	35.0	Cluster_23350
413.98000	11.740	12.090	35.0	Cluster_23354
414.71000	1.530	2.160	35.0	Cluster_23379
414.85000	1.610	2.210	35.0	Cluster_23397
414.87000	14.710	14.970	35.0	Cluster_07924
415.22000	1.230	1.480	35.0	Cluster_23422
415.73000	22.500	23.100	35.0	Cluster_23434
416.00000	14.750	15.180	35.0	Cluster_23448
416.22000	7.630	7.950	35.0	Cluster_23463
416.76000	7.470	7.800	35.0	Cluster_23476
416.88000	1.230	1.600	35.0	Cluster_23482
416.94000	17.320	17.820	35.0	Cluster_01353
418.25000	17.280	17.520	35.0	Cluster_23534
418.47000	7.650	7.880	35.0	Cluster_23549
419.18000	17.330	17.880	35.0	Cluster_01360
419.21000	7.510	7.930	35.0	Cluster_07978
419.88000	1.270	1.430	35.0	Cluster_23600
420.19000	11.580	11.880	35.0	Cluster_23617
420.23000	14.640	15.400	35.0	Cluster_23612
420.71000	7.310	8.340	35.0	Cluster_23632
421.56000	7.310	8.090	35.0	Cluster_08005
422.12000	1.580	2.070	35.0	Cluster_08008
422.55000	31.510	31.860	35.0	Cluster_08014
422.58000	25.320	25.840	35.0	Cluster_08015
423.14000	11.570	12.000	35.0	Cluster_23727
423.21000	14.870	15.370	35.0	Cluster_08022
423.24000	11.740	12.070	35.0	Cluster_23740
423.44000	17.530	18.010	35.0	Cluster_01387
423.71000	27.930	28.180	35.0	Cluster_23753

423.74000	22.510	23.020	35.0	Cluster_23766
424.18000	7.300	7.920	35.0	Cluster_23787
424.51000	11.730	12.470	35.0	Cluster_23811
424.68000	11.820	12.260	35.0	Cluster_23813
425.18000	7.350	7.860	35.0	Cluster_23850
425.46000	11.660	11.990	35.0	Cluster_23865
425.87000	7.600	8.080	35.0	Cluster_08056
425.88000	1.340	1.550	35.0	Cluster_23895
426.14000	7.470	7.920	35.0	Cluster_23901
426.24000	25.190	25.440	35.0	Cluster_23924
426.44000	7.530	7.670	35.0	Cluster_23942
426.88000	11.830	12.340	35.0	Cluster_08077
427.51000	17.320	17.760	35.0	Cluster_08094
428.56000	14.860	15.120	35.0	Cluster_08107
428.72000	7.840	8.690	35.0	Cluster_24045
428.79000	17.280	17.930	35.0	Cluster_24059
429.24000	7.630	7.910	35.0	Cluster_24074
429.57000	22.600	23.060	35.0	Cluster_08120
429.72000	14.590	14.880	35.0	Cluster_24100
429.76000	7.420	7.660	35.0	Cluster_24093
429.85000	22.580	22.890	35.0	Cluster_08124
429.95000	17.400	17.830	35.0	Cluster_01432
430.73000	27.890	28.160	35.0	Cluster_24136
431.21000	1.610	1.860	35.0	Cluster_24172
431.41000	17.290	17.520	35.0	Cluster_24179
431.74000	14.710	14.950	35.0	Cluster_24194
432.16000	11.860	12.140	35.0	Cluster_24207
432.21000	11.630	11.940	35.0	Cluster_24215
432.87000	14.770	14.990	35.0	Cluster_08164
433.96000	1.640	1.960	35.0	Cluster_24289
434.79000	17.290	17.460	35.0	Cluster_24347
434.97000	17.370	17.820	35.0	Cluster_24350
435.17000	11.710	11.980	35.0	Cluster_24357
435.24000	14.610	15.010	35.0	Cluster_08201
435.37000	25.040	25.410	35.0	Cluster_24383
435.70000	1.590	2.060	35.0	Cluster_01481
435.97000	17.270	18.110	35.0	Cluster_01487
436.48000	22.610	22.960	35.0	Cluster_01491
437.37000	7.350	7.700	35.0	Cluster_24491
437.46000	14.860	15.410	35.0	Cluster_01497
438.22000	7.510	8.020	35.0	Cluster_24532
438.30000	20.000	20.340	35.0	Cluster_24543
438.96000	17.260	17.670	35.0	Cluster_01503
438.97000	22.600	22.960	35.0	Cluster_01504
439.82000	7.670	8.890	35.0	Cluster_24632
440.21000	14.830	15.150	35.0	Cluster_01512
440.56000	7.700	7.920	35.0	Cluster_24655
440.74000	7.450	7.710	35.0	Cluster_24659
440.86000	1.610	2.060	35.0	Cluster_08277
441.21000	14.690	14.880	35.0	Cluster_24682
441.24000	25.130	25.470	35.0	Cluster_01516

441.73000	7.710	7.920	35.0	Cluster_24703
441.87000	11.740	12.340	35.0	Cluster_08293
442.00000	14.790	14.990	35.0	Cluster_24716
442.28000	14.810	15.170	35.0	Cluster_24730
442.57000	14.840	15.230	35.0	Cluster_08303
442.77000	11.790	12.030	35.0	Cluster_24758
442.89000	25.110	25.560	35.0	Cluster_24763
442.98000	11.680	12.030	35.0	Cluster_24761
443.12000	1.590	2.030	35.0	Cluster_24771
443.22000	17.370	17.550	35.0	Cluster_24782
443.28000	22.540	22.660	35.0	Cluster_24785
443.29000	22.640	22.950	35.0	Cluster_24795
443.89000	7.480	8.970	35.0	Cluster_08322
443.92000	11.750	12.130	35.0	Cluster_08324
444.72000	1.250	1.570	35.0	Cluster_24866
445.18000	14.620	14.980	35.0	Cluster_08343
445.55000	11.740	12.010	35.0	Cluster_24900
446.27000	14.850	15.060	35.0	Cluster_24941
446.73000	11.850	12.030	35.0	Cluster_24959
446.91000	19.900	20.460	35.0	Cluster_08369
447.17000	7.690	8.410	35.0	Cluster_24974
447.25000	22.590	23.160	35.0	Cluster_08376
447.28000	7.590	7.950	35.0	Cluster_24982
447.29000	7.410	7.710	35.0	Cluster_24988
447.60000	11.750	12.350	35.0	Cluster_08380
448.30000	11.860	12.310	35.0	Cluster_25022
448.79000	7.770	8.190	35.0	Cluster_25051
448.97000	11.580	12.360	35.0	Cluster_01573
449.25000	17.320	18.580	35.0	Cluster_08416
449.74000	14.850	15.190	35.0	Cluster_25092
449.84000	1.260	1.540	35.0	Cluster_25102
450.23000	14.590	14.860	35.0	Cluster_25125
450.93000	17.440	18.090	35.0	Cluster_08435
451.14000	7.390	7.860	35.0	Cluster_25153
451.17000	1.580	1.900	35.0	Cluster_25168
451.57000	20.060	20.280	35.0	Cluster_08446
452.13000	1.600	1.910	35.0	Cluster_25195
452.26000	14.770	15.110	35.0	Cluster_08462
452.75000	22.580	23.210	35.0	Cluster_25235
453.14000	1.450	1.910	35.0	Cluster_25244
453.24000	11.780	12.210	35.0	Cluster_25248
454.68000	28.050	28.170	35.0	Cluster_25320
454.70000	1.440	1.890	35.0	Cluster_25319
454.74000	11.700	12.160	35.0	Cluster_25325
455.15000	7.410	7.710	35.0	Cluster_25343
455.18000	17.430	17.890	35.0	Cluster_01617
455.20000	25.200	25.350	35.0	Cluster_08518
456.24000	7.470	7.700	35.0	Cluster_25393
456.26000	20.060	20.430	35.0	Cluster_08536
456.57000	11.860	12.310	35.0	Cluster_08539
456.75000	14.690	14.920	35.0	Cluster_25404

456.78000	11.630	12.040	35.0	Cluster_25413
457.20000	22.620	22.950	35.0	Cluster_25422
457.75000	7.570	8.250	35.0	Cluster_25448
457.76000	19.950	20.130	35.0	Cluster_25454
458.69000	7.650	8.440	35.0	Cluster_25493
458.76000	11.740	12.070	35.0	Cluster_25500
459.22000	7.770	8.510	35.0	Cluster_08566
459.25000	7.430	7.750	35.0	Cluster_25514
459.78000	11.640	12.160	35.0	Cluster_25540
459.90000	22.680	23.130	35.0	Cluster_08580
460.27000	14.580	14.840	35.0	Cluster_25572
460.77000	7.570	7.800	35.0	Cluster_25581
460.97000	19.890	20.150	35.0	Cluster_01663
461.60000	17.530	17.830	35.0	Cluster_08592
461.70000	7.800	8.280	35.0	Cluster_25618
461.73000	11.600	12.100	35.0	Cluster_25620
462.22000	14.740	15.170	35.0	Cluster_08608
462.50000	11.680	12.120	35.0	Cluster_25643
463.61000	17.260	17.520	35.0	Cluster_25677
463.73000	7.690	7.930	35.0	Cluster_25692
464.22000	20.010	20.270	35.0	Cluster_08632
464.30000	22.630	23.030	35.0	Cluster_25714
464.55000	22.620	23.550	35.0	Cluster_08640
465.77000	7.690	7.940	35.0	Cluster_25807
465.95000	17.270	17.670	35.0	Cluster_08659
465.99000	11.720	12.350	35.0	Cluster_01692
466.27000	14.870	15.280	35.0	Cluster_25822
466.90000	14.760	15.170	35.0	Cluster_08673
467.18000	7.360	7.770	35.0	Cluster_08677
467.25000	7.800	8.810	35.0	Cluster_08678
467.30000	25.180	25.510	35.0	Cluster_25858
467.67000	25.110	25.470	35.0	Cluster_00144
467.72000	17.470	17.920	35.0	Cluster_25877
468.89000	17.410	17.840	35.0	Cluster_08711
469.26000	31.370	31.710	35.0	Cluster_08720
469.74000	11.690	11.980	35.0	Cluster_25967
469.88000	14.730	15.000	35.0	Cluster_08724
470.43000	19.910	20.270	35.0	Cluster_26002
470.56000	7.620	7.940	35.0	Cluster_00010
470.92000	11.610	12.050	35.0	Cluster_08745
471.00000	11.820	12.340	35.0	Cluster_01731
471.22000	1.510	2.030	35.0	Cluster_08748
471.25000	22.510	22.710	35.0	Cluster_01737
471.26000	22.690	22.990	35.0	Cluster_01738
471.48000	17.440	17.680	35.0	Cluster_26044
471.74000	14.730	14.930	35.0	Cluster_26063
471.84000	7.370	7.650	35.0	Cluster_26052
472.23000	22.680	22.910	35.0	Cluster_26081
472.24000	11.690	12.530	35.0	Cluster_08764
472.72000	11.740	12.060	35.0	Cluster_26107
472.76000	11.600	11.750	35.0	Cluster_26106

472.77000	14.810	15.350	35.0	Cluster_26113
473.27000	11.610	11.880	35.0	Cluster_26146
473.77000	17.300	17.560	35.0	Cluster_26157
474.54000	17.320	17.750	35.0	Cluster_08802
474.58000	14.870	15.300	35.0	Cluster_08800
474.85000	7.400	7.630	35.0	Cluster_08806
475.58000	19.890	20.200	35.0	Cluster_08820
475.78000	20.000	20.420	35.0	Cluster_26234
476.21000	1.250	1.470	35.0	Cluster_26244
476.23000	7.750	8.120	35.0	Cluster_08830
476.28000	14.840	15.180	35.0	Cluster_08832
477.07000	19.920	20.380	35.0	Cluster_26275
477.20000	22.680	23.010	35.0	Cluster_08843
477.27000	17.480	17.750	35.0	Cluster_08840
477.55000	14.840	15.510	35.0	Cluster_08851
477.61000	22.510	22.910	35.0	Cluster_08856
477.93000	19.960	20.240	35.0	Cluster_08861
478.22000	1.490	1.880	35.0	Cluster_26309
478.25000	7.330	7.580	35.0	Cluster_26313
478.76000	11.590	11.940	35.0	Cluster_26329
478.77000	14.670	14.890	35.0	Cluster_26332
478.93000	25.120	26.580	35.0	Cluster_08880
478.99000	7.590	7.870	35.0	Cluster_01780
479.57000	7.510	8.630	35.0	Cluster_08888
479.71000	17.270	17.470	35.0	Cluster_26429
479.76000	19.940	20.380	35.0	Cluster_26423
480.22000	11.730	12.180	35.0	Cluster_08900
480.70000	11.660	11.900	35.0	Cluster_26476
481.22000	1.550	1.900	35.0	Cluster_26493
481.27000	14.680	15.340	35.0	Cluster_26504
481.28000	27.810	28.270	35.0	Cluster_08920
481.56000	7.600	8.560	35.0	Cluster_08922
481.58000	11.570	12.170	35.0	Cluster_08924
481.72000	17.290	17.460	35.0	Cluster_26523
481.73000	19.870	20.200	35.0	Cluster_01804
481.79000	11.560	11.750	35.0	Cluster_26518
482.20000	7.630	8.370	35.0	Cluster_01810
482.28000	7.540	7.710	35.0	Cluster_26547
482.49000	19.890	20.100	35.0	Cluster_26549
483.24000	1.580	2.000	35.0	Cluster_26594
483.30000	22.610	22.980	35.0	Cluster_26603
483.76000	7.690	7.970	35.0	Cluster_26625
483.84000	1.270	1.660	35.0	Cluster_26627
484.09000	17.390	17.580	35.0	Cluster_26635
484.73000	19.950	20.210	35.0	Cluster_26685
484.76000	11.760	12.370	35.0	Cluster_26681
485.06000	25.120	25.450	35.0	Cluster_00166
485.54000	11.860	12.200	35.0	Cluster_08981
488.29000	17.460	17.920	35.0	Cluster_26837
488.61000	27.970	28.270	35.0	Cluster_09032
488.79000	19.960	20.480	35.0	Cluster_26859

488.95000	19.910	20.540	35.0	Cluster_09038
489.74000	19.910	20.150	35.0	Cluster_26904
489.93000	11.670	12.140	35.0	Cluster_09048
490.50000	11.570	12.530	35.0	Cluster_26931
490.70000	1.610	2.010	35.0	Cluster_26935
490.77000	14.620	14.740	35.0	Cluster_26939
492.55000	14.810	14.940	35.0	Cluster_09097
492.60000	14.680	14.860	35.0	Cluster_09099
493.27000	17.370	17.580	35.0	Cluster_27041
494.26000	7.650	7.870	35.0	Cluster_27100
495.19000	19.900	20.310	35.0	Cluster_27149
495.71000	1.570	1.910	35.0	Cluster_27170
495.95000	22.630	22.900	35.0	Cluster_09160
496.24000	19.960	20.280	35.0	Cluster_01895
496.95000	31.350	31.660	35.0	Cluster_09176
497.35000	1.270	1.610	35.0	Cluster_27258
497.74000	7.370	7.780	35.0	Cluster_27265
497.80000	17.360	17.690	35.0	Cluster_27275
497.94000	22.570	23.390	35.0	Cluster_09191
498.01000	25.130	25.440	35.0	Cluster_01929
498.50000	11.610	11.980	35.0	Cluster_27299
498.73000	7.580	7.760	35.0	Cluster_27302
499.23000	14.690	15.160	35.0	Cluster_09211
499.33000	22.660	23.010	35.0	Cluster_27324
499.76000	22.660	22.870	35.0	Cluster_01941
499.92000	19.990	20.350	35.0	Cluster_09218
499.96000	27.850	28.580	35.0	Cluster_09219
500.02000	1.280	1.610	35.0	Cluster_27355
500.63000	22.660	23.320	35.0	Cluster_09231
500.70000	7.470	8.230	35.0	Cluster_27401
500.96000	1.250	1.490	35.0	Cluster_01956
501.22000	14.600	15.020	35.0	Cluster_27436
501.59000	19.900	20.410	35.0	Cluster_09244
501.76000	25.270	25.430	35.0	Cluster_27459
502.21000	14.840	15.400	35.0	Cluster_01970
502.22000	19.940	20.310	35.0	Cluster_27473
502.59000	11.820	12.250	35.0	Cluster_09268
502.76000	7.580	8.030	35.0	Cluster_27485
503.25000	7.500	7.680	35.0	Cluster_27512
503.28000	27.860	28.290	35.0	Cluster_27518
504.21000	17.260	17.490	35.0	Cluster_27547
504.56000	14.800	15.010	35.0	Cluster_09288
504.98000	25.280	25.740	35.0	Cluster_01984
505.01000	17.500	18.120	35.0	Cluster_27567
505.24000	19.920	20.380	35.0	Cluster_09298
505.52000	11.640	11.980	35.0	Cluster_01990
505.60000	14.600	14.880	35.0	Cluster_09305
505.79000	14.760	15.190	35.0	Cluster_27593
506.73000	1.450	1.900	35.0	Cluster_27611
506.74000	22.540	22.870	35.0	Cluster_27621
506.76000	11.670	11.950	35.0	Cluster_27614

507.25000	11.830	11.980	35.0	Cluster_27638
507.97000	14.640	15.020	35.0	Cluster_09350
508.62000	20.060	20.640	35.0	Cluster_09366
508.70000	1.600	2.010	35.0	Cluster_27686
508.71000	31.670	31.910	35.0	Cluster_27692
509.20000	14.750	15.200	35.0	Cluster_27715
509.26000	17.360	17.720	35.0	Cluster_27718
509.87000	19.870	20.150	35.0	Cluster_09377
509.91000	11.720	12.580	35.0	Cluster_09383
511.23000	19.900	20.500	35.0	Cluster_27820
511.79000	25.080	25.960	35.0	Cluster_27852
511.91000	7.750	8.370	35.0	Cluster_09420
512.25000	22.680	22.900	35.0	Cluster_27867
512.27000	17.380	17.700	35.0	Cluster_27865
513.09000	7.390	7.620	35.0	Cluster_27898
513.77000	14.700	15.030	35.0	Cluster_27929
514.72000	7.380	7.820	35.0	Cluster_27964
514.74000	11.660	11.800	35.0	Cluster_27971
515.25000	11.820	12.340	35.0	Cluster_02053
515.48000	14.690	15.030	35.0	Cluster_02061
515.90000	17.500	17.700	35.0	Cluster_09478
516.74000	7.410	8.010	35.0	Cluster_28062
517.92000	17.270	17.770	35.0	Cluster_09503
518.00000	7.400	8.010	35.0	Cluster_28115
518.26000	7.480	8.140	35.0	Cluster_09508
518.31000	22.490	22.790	35.0	Cluster_28130
518.51000	11.730	11.950	35.0	Cluster_28138
518.59000	25.120	25.590	35.0	Cluster_09521
518.78000	11.860	12.260	35.0	Cluster_28152
519.59000	19.910	20.490	35.0	Cluster_09531
519.81000	11.710	11.900	35.0	Cluster_28200
520.04000	11.820	12.330	35.0	Cluster_28202
520.51000	19.930	20.220	35.0	Cluster_28225
520.80000	19.990	20.360	35.0	Cluster_28240
520.81000	14.720	14.890	35.0	Cluster_28232
521.02000	27.920	28.760	35.0	Cluster_02095
521.26000	25.120	25.760	35.0	Cluster_09553
521.66000	14.750	14.990	35.0	Cluster_28271
522.22000	19.900	20.320	35.0	Cluster_28286
522.45000	17.530	17.930	35.0	Cluster_02102
522.50000	14.850	15.550	35.0	Cluster_28303
522.62000	22.580	23.260	35.0	Cluster_09582
522.75000	14.580	14.890	35.0	Cluster_28315
523.22000	1.540	1.970	35.0	Cluster_28350
523.27000	22.580	22.760	35.0	Cluster_28334
523.35000	7.710	8.070	35.0	Cluster_28353
523.92000	27.830	28.280	35.0	Cluster_09603
523.97000	19.890	20.330	35.0	Cluster_28374
524.28000	7.440	7.750	35.0	Cluster_28386
526.18000	27.810	27.980	35.0	Cluster_28467
526.20000	19.930	20.490	35.0	Cluster_09656

526.93000	17.300	17.490	35.0	Cluster_09676
526.95000	17.420	17.830	35.0	Cluster_09677
527.76000	14.630	14.940	35.0	Cluster_28507
528.23000	25.180	25.480	35.0	Cluster_28536
528.27000	31.350	31.880	35.0	Cluster_00184
528.32000	11.860	12.150	35.0	Cluster_28539
528.36000	1.420	1.660	35.0	Cluster_28552
528.97000	25.130	25.350	35.0	Cluster_09710
529.25000	1.250	1.600	35.0	Cluster_09712
529.79000	27.930	28.250	35.0	Cluster_28615
530.20000	19.970	20.370	35.0	Cluster_28629
530.26000	28.010	28.680	35.0	Cluster_02153
530.51000	28.000	28.590	35.0	Cluster_28657
530.81000	19.940	20.120	35.0	Cluster_28677
530.94000	17.380	17.810	35.0	Cluster_09741
531.75000	11.600	11.860	35.0	Cluster_28707
531.77000	11.850	12.330	35.0	Cluster_28708
532.24000	19.900	20.560	35.0	Cluster_09763
532.27000	19.900	20.070	35.0	Cluster_09766
532.62000	27.970	28.560	35.0	Cluster_09768
532.97000	17.500	18.660	35.0	Cluster_09773
533.15000	1.420	1.860	35.0	Cluster_28752
533.16000	1.570	1.940	35.0	Cluster_28753
533.19000	27.910	28.680	35.0	Cluster_09774
533.32000	31.520	34.610	35.0	Cluster_09778
533.70000	27.990	28.510	35.0	Cluster_28783
533.82000	25.050	25.470	35.0	Cluster_28799
533.91000	25.110	25.630	35.0	Cluster_09792
534.28000	7.780	8.110	35.0	Cluster_28807
534.59000	17.310	17.790	35.0	Cluster_09803
535.32000	31.570	34.610	35.0	Cluster_09812
535.80000	14.830	15.050	35.0	Cluster_28867
535.94000	22.660	23.170	35.0	Cluster_09822
536.06000	14.680	14.880	35.0	Cluster_28878
536.26000	17.340	17.850	35.0	Cluster_09823
536.27000	11.740	12.330	35.0	Cluster_28888
536.48000	1.270	1.570	35.0	Cluster_28899
536.60000	28.030	28.230	35.0	Cluster_09834
537.23000	19.940	20.380	35.0	Cluster_09849
537.56000	20.040	20.430	35.0	Cluster_09853
537.69000	20.000	20.310	35.0	Cluster_28943
537.91000	17.280	17.830	35.0	Cluster_09869
538.84000	27.940	29.330	35.0	Cluster_28980
538.97000	25.250	26.160	35.0	Cluster_09886
538.98000	25.040	25.270	35.0	Cluster_09885
539.01000	14.600	14.840	35.0	Cluster_28985
539.28000	11.840	12.000	35.0	Cluster_28993
539.52000	7.750	8.260	35.0	Cluster_29001
540.02000	7.730	8.260	35.0	Cluster_09899
540.27000	20.030	20.690	35.0	Cluster_29031
540.79000	11.560	12.000	35.0	Cluster_29052

541.34000	11.580	11.860	35.0	Cluster_29085
541.44000	19.890	20.040	35.0	Cluster_29098
541.82000	11.830	12.010	35.0	Cluster_29115
542.26000	31.550	31.980	35.0	Cluster_02236
542.28000	17.530	17.810	35.0	Cluster_02229
542.52000	19.870	20.080	35.0	Cluster_02233
542.77000	11.860	12.200	35.0	Cluster_29162
543.27000	22.630	23.250	35.0	Cluster_29193
544.95000	11.820	12.320	35.0	Cluster_09982
545.77000	7.360	7.830	35.0	Cluster_29280
545.92000	17.420	18.000	35.0	Cluster_09992
546.31000	14.620	14.810	35.0	Cluster_29312
547.32000	17.390	17.760	35.0	Cluster_29345
548.27000	14.730	15.170	35.0	Cluster_29389
548.46000	25.260	25.750	35.0	Cluster_00194
548.61000	1.570	2.000	35.0	Cluster_10035
548.64000	22.690	23.540	35.0	Cluster_10037
549.25000	27.830	28.270	35.0	Cluster_10044
549.28000	7.590	7.870	35.0	Cluster_29418
549.90000	11.780	12.150	35.0	Cluster_10052
549.99000	25.100	25.460	35.0	Cluster_10058
550.22000	7.560	7.770	35.0	Cluster_29451
550.31000	22.610	23.060	35.0	Cluster_29461
550.55000	17.370	17.630	35.0	Cluster_10069
550.61000	14.840	15.150	35.0	Cluster_10068
550.78000	7.640	7.920	35.0	Cluster_29465
550.84000	17.510	17.830	35.0	Cluster_29476
551.01000	25.090	25.830	35.0	Cluster_29483
551.29000	11.610	11.870	35.0	Cluster_29499
551.57000	7.610	8.130	35.0	Cluster_10082
551.97000	17.510	17.780	35.0	Cluster_10089
552.36000	19.970	20.430	35.0	Cluster_29545
552.74000	7.590	7.850	35.0	Cluster_29553
552.75000	1.570	2.190	35.0	Cluster_29548
552.96000	25.250	25.480	35.0	Cluster_10104
553.55000	19.990	20.610	35.0	Cluster_29579
553.63000	11.870	12.380	35.0	Cluster_10116
554.58000	27.910	28.110	35.0	Cluster_10130
554.60000	17.510	17.920	35.0	Cluster_10136
554.97000	22.600	23.080	35.0	Cluster_10144
556.25000	14.750	15.130	35.0	Cluster_29692
556.68000	27.880	28.460	35.0	Cluster_10177
556.96000	14.720	14.880	35.0	Cluster_10184
557.73000	14.790	15.330	35.0	Cluster_29750
558.23000	17.360	17.700	35.0	Cluster_10209
558.65000	14.880	15.260	35.0	Cluster_10215
558.81000	22.580	23.130	35.0	Cluster_29792
559.29000	7.590	7.940	35.0	Cluster_29811
560.35000	28.000	28.210	35.0	Cluster_29845
560.65000	19.890	20.480	35.0	Cluster_10245
560.83000	25.190	25.430	35.0	Cluster_29862

561.28000	28.030	28.230	35.0	Cluster_29879
561.64000	31.590	32.110	35.0	Cluster_10262
561.70000	25.100	26.430	35.0	Cluster_29892
561.81000	17.510	17.830	35.0	Cluster_29901
561.98000	22.680	25.390	35.0	Cluster_10266
562.07000	7.430	7.710	35.0	Cluster_29906
562.29000	19.900	20.370	35.0	Cluster_29915
563.32000	19.870	20.460	35.0	Cluster_10288
563.50000	22.690	23.220	35.0	Cluster_02355
563.75000	14.750	15.200	35.0	Cluster_29975
563.76000	22.530	22.950	35.0	Cluster_29965
564.78000	14.710	15.270	35.0	Cluster_30003
564.79000	20.010	20.400	35.0	Cluster_02366
565.56000	19.960	20.140	35.0	Cluster_30039
565.60000	25.100	25.530	35.0	Cluster_10318
566.33000	11.630	12.010	35.0	Cluster_30080
566.74000	20.060	20.350	35.0	Cluster_02376
566.79000	7.390	7.780	35.0	Cluster_30088
566.90000	22.620	22.840	35.0	Cluster_30092
566.98000	22.640	23.320	35.0	Cluster_10333
567.07000	17.470	17.720	35.0	Cluster_30105
567.24000	14.640	14.920	35.0	Cluster_30110
567.30000	28.010	28.530	35.0	Cluster_30123
567.31000	11.850	12.010	35.0	Cluster_30114
568.56000	25.160	25.510	35.0	Cluster_02384
568.73000	14.840	15.260	35.0	Cluster_30166
568.79000	31.530	31.820	35.0	Cluster_30177
568.80000	17.460	17.930	35.0	Cluster_30176
568.85000	28.010	28.520	35.0	Cluster_30178
569.06000	25.130	25.340	35.0	Cluster_02386
569.57000	25.040	25.390	35.0	Cluster_02390
569.82000	17.530	17.910	35.0	Cluster_30204
570.30000	17.480	18.020	35.0	Cluster_02396
570.36000	22.500	22.780	35.0	Cluster_30219
570.73000	20.060	20.310	35.0	Cluster_02397
570.77000	7.460	7.770	35.0	Cluster_30236
571.22000	17.470	17.700	35.0	Cluster_30252
571.34000	27.880	28.210	35.0	Cluster_30256
571.64000	11.820	12.560	35.0	Cluster_10409
571.77000	7.480	8.050	35.0	Cluster_30264
572.13000	22.500	23.000	35.0	Cluster_00213
572.30000	17.340	18.190	35.0	Cluster_10420
572.79000	14.660	14.850	35.0	Cluster_30301
573.35000	22.660	22.940	35.0	Cluster_30317
574.26000	11.830	12.020	35.0	Cluster_10456
574.80000	11.580	11.830	35.0	Cluster_30355
575.25000	31.530	33.580	35.0	Cluster_02433
575.33000	22.510	22.760	35.0	Cluster_02435
576.55000	17.430	17.830	35.0	Cluster_02439
576.77000	14.720	14.850	35.0	Cluster_30417
576.98000	11.830	12.410	35.0	Cluster_10494

577.30000	19.960	20.490	35.0	Cluster_10500
577.81000	31.450	31.790	35.0	Cluster_30446
578.32000	17.430	17.920	35.0	Cluster_30460
578.55000	19.870	20.210	35.0	Cluster_02454
578.86000	22.490	22.830	35.0	Cluster_30486
578.98000	14.820	15.290	35.0	Cluster_10523
579.33000	11.750	12.130	35.0	Cluster_30494
579.55000	25.260	25.600	35.0	Cluster_02460
579.61000	7.580	8.870	35.0	Cluster_10531
579.63000	22.500	23.120	35.0	Cluster_10533
579.77000	11.700	12.170	35.0	Cluster_30512
579.81000	17.460	17.700	35.0	Cluster_30517
579.83000	14.670	15.090	35.0	Cluster_30515
580.36000	17.280	17.480	35.0	Cluster_30555
580.86000	25.310	25.670	35.0	Cluster_02466
581.28000	20.030	20.280	35.0	Cluster_02468
581.31000	7.630	7.950	35.0	Cluster_30577
581.96000	11.650	12.310	35.0	Cluster_10563
582.31000	7.770	8.190	35.0	Cluster_30648
582.54000	17.320	17.540	35.0	Cluster_02478
582.65000	25.140	25.550	35.0	Cluster_10574
582.70000	22.490	23.000	35.0	Cluster_00226
582.74000	1.640	2.170	35.0	Cluster_30654
584.05000	19.980	20.140	35.0	Cluster_30718
584.31000	14.750	15.070	35.0	Cluster_30724
584.54000	25.270	25.550	35.0	Cluster_30733
585.02000	14.690	15.040	35.0	Cluster_30763
585.29000	25.160	25.780	35.0	Cluster_10612
585.35000	31.700	32.250	35.0	Cluster_02501
585.80000	31.510	31.730	35.0	Cluster_30791
585.84000	27.950	28.760	35.0	Cluster_30792
586.62000	14.830	15.010	35.0	Cluster_10630
586.75000	1.640	2.010	35.0	Cluster_30815
587.30000	14.680	14.880	35.0	Cluster_10642
587.33000	11.630	11.810	35.0	Cluster_30848
587.34000	17.340	17.720	35.0	Cluster_30837
587.65000	14.830	15.170	35.0	Cluster_10646
588.69000	11.810	12.000	35.0	Cluster_30892
588.82000	19.980	20.330	35.0	Cluster_30900
589.55000	20.030	20.200	35.0	Cluster_30932
589.74000	7.420	7.770	35.0	Cluster_30933
589.96000	20.020	20.580	35.0	Cluster_10677
590.22000	17.360	17.460	35.0	Cluster_30952
590.30000	17.400	17.930	35.0	Cluster_30953
591.04000	20.040	20.300	35.0	Cluster_30998
591.44000	7.460	7.700	35.0	Cluster_31008
591.52000	25.310	25.570	35.0	Cluster_02538
591.66000	17.410	17.790	35.0	Cluster_10703
591.81000	31.510	31.730	35.0	Cluster_31027
592.01000	22.470	22.920	35.0	Cluster_10706
592.79000	7.750	8.200	35.0	Cluster_31056

592.82000	28.040	28.460	35.0	Cluster_31060
593.06000	25.120	25.660	35.0	Cluster_02547
593.07000	25.320	25.710	35.0	Cluster_02548
593.33000	22.540	22.890	35.0	Cluster_10724
593.34000	11.740	12.200	35.0	Cluster_31073
593.93000	17.370	17.520	35.0	Cluster_10743
594.32000	27.980	28.390	35.0	Cluster_10753
595.59000	17.510	17.810	35.0	Cluster_31146
595.62000	17.380	17.730	35.0	Cluster_10769
596.75000	7.800	8.600	35.0	Cluster_31177
597.67000	31.700	32.040	35.0	Cluster_10808
597.81000	22.500	22.900	35.0	Cluster_31212
597.82000	17.410	17.830	35.0	Cluster_31208
598.29000	14.840	15.150	35.0	Cluster_10814
598.36000	27.860	28.450	35.0	Cluster_10818
599.36000	19.990	20.260	35.0	Cluster_31267
599.66000	31.720	32.150	35.0	Cluster_10840
599.95000	19.900	20.430	35.0	Cluster_10848
600.81000	7.740	8.050	35.0	Cluster_31316
600.97000	11.800	12.190	35.0	Cluster_10858
601.36000	22.680	23.100	35.0	Cluster_31339
602.32000	27.800	28.110	35.0	Cluster_31390
602.83000	20.070	20.220	35.0	Cluster_31406
603.30000	11.680	12.200	35.0	Cluster_31409
603.51000	7.800	7.990	35.0	Cluster_02598
603.53000	19.920	20.190	35.0	Cluster_31419
603.96000	11.700	12.340	35.0	Cluster_10888
604.80000	17.520	17.930	35.0	Cluster_31463
605.31000	11.820	12.480	35.0	Cluster_31473
605.67000	25.090	25.450	35.0	Cluster_10910
606.07000	31.390	31.590	35.0	Cluster_31501
606.27000	7.750	7.930	35.0	Cluster_31514
606.87000	25.100	25.350	35.0	Cluster_31531
606.89000	25.280	25.620	35.0	Cluster_10941
607.32000	14.800	15.120	35.0	Cluster_31536
608.09000	22.520	22.950	35.0	Cluster_02626
608.32000	31.350	31.690	35.0	Cluster_31587
608.49000	19.940	20.290	35.0	Cluster_31590
608.78000	14.760	15.080	35.0	Cluster_31595
608.79000	1.520	1.980	35.0	Cluster_31601
609.06000	17.530	17.740	35.0	Cluster_31605
609.81000	7.750	8.000	35.0	Cluster_31625
610.31000	31.440	31.740	35.0	Cluster_31655
611.34000	22.690	22.940	35.0	Cluster_31685
612.32000	14.860	14.970	35.0	Cluster_11010
613.83000	27.990	28.170	35.0	Cluster_31794
614.06000	14.760	14.970	35.0	Cluster_31796
614.07000	17.250	17.600	35.0	Cluster_02670
614.19000	1.610	1.960	35.0	Cluster_31797
614.26000	17.420	18.050	35.0	Cluster_11035
615.83000	31.340	31.550	35.0	Cluster_31852

616.08000	31.590	31.820	35.0	Cluster_02680
616.32000	28.000	28.760	35.0	Cluster_11075
616.55000	14.620	14.950	35.0	Cluster_31873
616.83000	14.850	14.940	35.0	Cluster_31882
617.02000	25.040	25.250	35.0	Cluster_31892
617.35000	31.420	32.000	35.0	Cluster_31911
617.93000	31.430	31.700	35.0	Cluster_31935
618.05000	11.870	12.540	35.0	Cluster_02693
618.79000	22.510	22.750	35.0	Cluster_31952
619.29000	27.910	28.450	35.0	Cluster_02701
619.33000	11.880	12.150	35.0	Cluster_31985
619.62000	14.870	15.340	35.0	Cluster_11116
620.53000	25.290	25.590	35.0	Cluster_32028
620.60000	17.420	17.850	35.0	Cluster_11128
621.30000	7.510	7.700	35.0	Cluster_32049
621.36000	31.580	31.760	35.0	Cluster_32053
621.39000	25.140	25.350	35.0	Cluster_32052
621.79000	22.560	22.900	35.0	Cluster_32057
621.98000	27.860	28.620	35.0	Cluster_11154
622.78000	14.670	14.880	35.0	Cluster_32095
622.98000	19.870	20.320	35.0	Cluster_11175
623.28000	11.660	12.090	35.0	Cluster_32112
623.36000	14.850	15.070	35.0	Cluster_32126
623.65000	28.070	28.400	35.0	Cluster_11191
623.98000	20.050	20.290	35.0	Cluster_11189
624.20000	1.600	1.870	35.0	Cluster_32153
624.28000	7.840	8.040	35.0	Cluster_32154
624.63000	28.070	28.390	35.0	Cluster_11202
625.04000	19.980	20.630	35.0	Cluster_02732
625.32000	11.570	12.160	35.0	Cluster_11203
625.58000	27.880	28.190	35.0	Cluster_02735
626.00000	20.030	20.250	35.0	Cluster_11225
626.07000	27.840	28.080	35.0	Cluster_32208
626.27000	25.120	25.590	35.0	Cluster_32218
626.28000	7.550	8.420	35.0	Cluster_32212
626.83000	11.680	11.830	35.0	Cluster_32244
627.30000	31.710	32.020	35.0	Cluster_32266
627.31000	17.250	17.550	35.0	Cluster_32263
627.50000	1.390	1.660	35.0	Cluster_32275
627.79000	17.480	17.610	35.0	Cluster_32283
628.07000	7.800	8.180	35.0	Cluster_32292
628.86000	1.450	1.770	35.0	Cluster_32321
629.63000	11.830	12.310	35.0	Cluster_11289
629.83000	14.580	15.190	35.0	Cluster_32344
629.96000	7.690	8.530	35.0	Cluster_11292
630.84000	11.630	11.990	35.0	Cluster_32375
631.37000	17.410	17.660	35.0	Cluster_11312
631.80000	14.840	15.180	35.0	Cluster_32410
632.05000	28.070	28.630	35.0	Cluster_11324
632.35000	31.350	31.640	35.0	Cluster_11329
632.78000	17.370	17.650	35.0	Cluster_32445

632.84000	11.640	11.850	35.0	Cluster_32450
633.00000	22.660	23.130	35.0	Cluster_11339
633.32000	14.850	15.230	35.0	Cluster_32465
633.36000	25.310	25.480	35.0	Cluster_32472
635.07000	22.620	23.020	35.0	Cluster_02772
635.82000	25.280	25.600	35.0	Cluster_02775
636.32000	31.510	31.820	35.0	Cluster_32584
636.84000	14.840	15.110	35.0	Cluster_32596
637.62000	7.690	8.090	35.0	Cluster_11403
637.83000	17.450	17.890	35.0	Cluster_32632
638.35000	22.570	22.950	35.0	Cluster_32655
638.36000	27.860	28.070	35.0	Cluster_11416
638.39000	20.000	20.130	35.0	Cluster_32659
638.79000	7.640	7.920	35.0	Cluster_32669
638.85000	17.450	17.760	35.0	Cluster_32673
639.85000	11.870	12.150	35.0	Cluster_32724
639.99000	11.800	12.120	35.0	Cluster_11446
640.32000	19.880	20.330	35.0	Cluster_32732
641.64000	19.900	20.110	35.0	Cluster_11472
641.84000	14.670	15.030	35.0	Cluster_32794
642.02000	25.230	25.570	35.0	Cluster_11476
643.07000	25.200	25.430	35.0	Cluster_32838
643.83000	11.760	12.250	35.0	Cluster_32863
644.00000	31.400	31.630	35.0	Cluster_11510
644.30000	7.630	8.960	35.0	Cluster_02836
644.36000	27.970	28.160	35.0	Cluster_32878
644.37000	25.080	25.430	35.0	Cluster_11511
645.00000	31.710	31.970	35.0	Cluster_11529
645.85000	14.780	14.940	35.0	Cluster_32926
646.30000	7.730	8.000	35.0	Cluster_32945
648.34000	27.840	28.040	35.0	Cluster_33005
648.67000	20.000	20.410	35.0	Cluster_11566
648.88000	17.540	17.890	35.0	Cluster_33029
649.01000	22.600	23.360	35.0	Cluster_11574
649.77000	7.630	7.950	35.0	Cluster_33054
650.31000	17.480	17.840	35.0	Cluster_33085
651.01000	22.660	23.440	35.0	Cluster_11595
651.08000	22.660	23.610	35.0	Cluster_33114
651.32000	25.070	25.170	35.0	Cluster_33124
651.36000	25.060	26.830	35.0	Cluster_11600
651.86000	31.600	32.050	35.0	Cluster_33158
651.99000	22.630	23.030	35.0	Cluster_11614
652.06000	22.590	23.040	35.0	Cluster_33159
652.35000	17.450	17.730	35.0	Cluster_33162
652.86000	11.860	12.170	35.0	Cluster_33187
653.33000	22.490	23.010	35.0	Cluster_33199
653.57000	14.650	14.940	35.0	Cluster_33207
653.60000	31.520	31.800	35.0	Cluster_02883
654.05000	22.470	22.620	35.0	Cluster_33221
654.68000	14.810	15.140	35.0	Cluster_11655
654.78000	7.650	7.850	35.0	Cluster_33237

655.99000	14.600	14.720	35.0	Cluster_11675
656.63000	14.820	15.370	35.0	Cluster_11676
657.90000	22.660	22.950	35.0	Cluster_33331
657.95000	22.660	22.890	35.0	Cluster_33350
658.07000	17.500	17.770	35.0	Cluster_33340
658.66000	14.810	15.150	35.0	Cluster_11699
659.28000	7.340	7.640	35.0	Cluster_33386
659.60000	14.800	15.150	35.0	Cluster_33396
659.63000	25.250	25.590	35.0	Cluster_02907
659.98000	7.810	8.090	35.0	Cluster_11725
660.33000	17.390	17.640	35.0	Cluster_33416
660.46000	17.280	17.490	35.0	Cluster_33423
660.84000	14.710	14.980	35.0	Cluster_33430
661.01000	27.910	28.720	35.0	Cluster_11741
661.31000	7.510	8.260	35.0	Cluster_33436
662.02000	22.620	23.480	35.0	Cluster_11748
662.12000	25.110	25.570	35.0	Cluster_02928
662.30000	11.760	12.160	35.0	Cluster_33472
663.37000	22.610	23.380	35.0	Cluster_33509
663.55000	14.680	15.020	35.0	Cluster_02941
663.60000	31.540	33.600	35.0	Cluster_02940
665.32000	11.790	12.000	35.0	Cluster_33570
666.35000	25.220	25.970	35.0	Cluster_02964
666.68000	31.630	31.810	35.0	Cluster_11814
666.95000	22.560	22.710	35.0	Cluster_33617
667.82000	11.770	12.000	35.0	Cluster_33644
668.33000	14.580	14.830	35.0	Cluster_33651
668.35000	22.530	22.820	35.0	Cluster_11848
669.10000	31.550	31.990	35.0	Cluster_02977
669.33000	7.730	7.920	35.0	Cluster_33680
669.78000	11.880	12.330	35.0	Cluster_33695
670.01000	20.010	20.600	35.0	Cluster_11876
670.40000	11.810	12.090	35.0	Cluster_33725
670.80000	1.580	2.170	35.0	Cluster_33734
670.82000	14.840	15.230	35.0	Cluster_33742
670.87000	31.500	31.700	35.0	Cluster_02986
671.44000	22.680	23.230	35.0	Cluster_33760
671.82000	14.870	15.080	35.0	Cluster_33770
671.97000	19.880	20.710	35.0	Cluster_11894
672.35000	17.390	17.900	35.0	Cluster_33781
672.81000	22.560	22.940	35.0	Cluster_33789
673.09000	31.550	31.860	35.0	Cluster_03005
673.37000	17.280	17.620	35.0	Cluster_33802
673.62000	31.510	31.800	35.0	Cluster_33824
673.65000	11.600	11.860	35.0	Cluster_11927
674.11000	31.240	31.630	35.0	Cluster_03014
674.32000	14.640	15.510	35.0	Cluster_11933
674.95000	31.550	32.110	35.0	Cluster_00274
675.37000	22.640	23.000	35.0	Cluster_33861
675.60000	31.330	31.510	35.0	Cluster_33864
675.64000	25.070	25.330	35.0	Cluster_03025

675.84000	25.240	25.960	35.0	Cluster_03026
676.00000	27.960	28.390	35.0	Cluster_11956
676.32000	17.510	17.840	35.0	Cluster_11959
677.20000	1.550	1.800	35.0	Cluster_33905
677.89000	17.290	17.370	35.0	Cluster_33946
678.81000	14.610	14.810	35.0	Cluster_33981
678.84000	19.900	20.130	35.0	Cluster_33980
678.85000	25.320	25.840	35.0	Cluster_03050
679.38000	31.570	31.860	35.0	Cluster_33996
679.79000	7.500	7.750	35.0	Cluster_34007
679.86000	22.690	23.500	35.0	Cluster_34018
680.08000	25.280	25.680	35.0	Cluster_03060
680.09000	19.920	20.480	35.0	Cluster_34023
680.87000	14.720	15.220	35.0	Cluster_34054
681.08000	25.320	25.720	35.0	Cluster_03066
681.32000	17.470	17.820	35.0	Cluster_12020
681.35000	11.710	11.930	35.0	Cluster_34074
682.09000	25.270	25.680	35.0	Cluster_34089
682.31000	1.380	1.610	35.0	Cluster_34106
682.38000	31.630	31.920	35.0	Cluster_03074
683.61000	17.540	17.710	35.0	Cluster_12059
683.81000	14.650	15.140	35.0	Cluster_34134
683.99000	11.820	12.300	35.0	Cluster_12062
684.08000	31.210	31.860	35.0	Cluster_03088
684.82000	17.510	17.670	35.0	Cluster_34171
684.83000	20.060	20.260	35.0	Cluster_34174
685.09000	31.700	32.020	35.0	Cluster_03095
685.10000	27.910	28.210	35.0	Cluster_03102
685.35000	25.210	25.420	35.0	Cluster_34201
686.04000	19.890	20.200	35.0	Cluster_12086
686.50000	27.930	28.200	35.0	Cluster_00285
686.75000	17.440	17.750	35.0	Cluster_00286
687.66000	27.960	28.390	35.0	Cluster_34255
689.34000	11.570	11.990	35.0	Cluster_34298
689.36000	14.790	15.320	35.0	Cluster_34295
689.57000	31.350	31.700	35.0	Cluster_03142
690.82000	7.740	8.350	35.0	Cluster_34324
690.84000	14.610	15.040	35.0	Cluster_34327
691.59000	22.500	22.700	35.0	Cluster_03162
691.79000	7.360	7.590	35.0	Cluster_34357
691.83000	25.240	25.490	35.0	Cluster_34369
692.26000	7.370	7.970	35.0	Cluster_34373
692.32000	19.900	20.660	35.0	Cluster_03167
692.35000	22.680	22.990	35.0	Cluster_03168
692.60000	31.670	32.670	35.0	Cluster_03182
692.84000	22.550	22.680	35.0	Cluster_34402
693.56000	31.300	31.830	35.0	Cluster_03192
693.60000	19.950	20.630	35.0	Cluster_34413
693.88000	14.790	15.050	35.0	Cluster_34418
694.36000	11.580	11.990	35.0	Cluster_34424
694.84000	22.670	22.890	35.0	Cluster_34436

694.87000	14.830	15.400	35.0	Cluster_34443
695.01000	19.930	20.930	35.0	Cluster_12183
695.36000	11.820	12.170	35.0	Cluster_34458
695.70000	25.120	25.630	35.0	Cluster_12190
695.87000	14.660	14.860	35.0	Cluster_34468
696.32000	7.450	7.550	35.0	Cluster_34489
696.34000	1.370	1.810	35.0	Cluster_03210
696.39000	17.310	17.590	35.0	Cluster_34483
696.58000	25.070	25.430	35.0	Cluster_03211
697.31000	31.260	31.680	35.0	Cluster_03215
697.37000	31.590	31.830	35.0	Cluster_34515
697.85000	14.600	15.110	35.0	Cluster_34520
697.87000	17.490	17.790	35.0	Cluster_34523
698.82000	17.530	17.830	35.0	Cluster_34541
698.83000	11.730	12.010	35.0	Cluster_34546
699.70000	19.920	20.280	35.0	Cluster_12244
699.85000	14.630	14.990	35.0	Cluster_03235
700.26000	1.650	1.780	35.0	Cluster_34580
700.36000	22.500	22.890	35.0	Cluster_34589
700.65000	22.540	22.880	35.0	Cluster_34590
700.85000	11.860	12.200	35.0	Cluster_34594
701.36000	14.670	14.980	35.0	Cluster_34614
702.34000	25.110	25.300	35.0	Cluster_34653
702.62000	14.690	14.940	35.0	Cluster_12286
703.04000	19.990	20.450	35.0	Cluster_12291
703.05000	31.270	31.700	35.0	Cluster_03266
703.33000	17.280	17.830	35.0	Cluster_34677
703.69000	25.310	25.600	35.0	Cluster_12310
703.91000	22.540	22.890	35.0	Cluster_03274
704.17000	22.580	22.900	35.0	Cluster_34698
704.21000	1.560	1.920	35.0	Cluster_34700
704.31000	7.790	7.920	35.0	Cluster_34706
704.81000	7.820	8.870	35.0	Cluster_34728
704.85000	28.070	28.260	35.0	Cluster_34734
705.04000	31.460	33.040	35.0	Cluster_12319
705.37000	19.890	20.240	35.0	Cluster_34742
705.83000	14.660	15.040	35.0	Cluster_34756
705.88000	11.630	12.130	35.0	Cluster_34757
706.86000	22.650	22.880	35.0	Cluster_03301
707.02000	25.090	25.400	35.0	Cluster_12346
707.80000	7.820	8.290	35.0	Cluster_34791
708.51000	11.650	12.330	35.0	Cluster_00309
709.28000	7.800	8.070	35.0	Cluster_34840
709.39000	17.500	17.840	35.0	Cluster_34846
710.88000	25.290	25.490	35.0	Cluster_03335
711.06000	22.690	23.590	35.0	Cluster_12397
711.85000	11.860	12.330	35.0	Cluster_34925
712.39000	22.630	22.910	35.0	Cluster_34948
712.84000	11.860	12.340	35.0	Cluster_34967
713.08000	28.070	28.340	35.0	Cluster_03351
713.29000	14.660	14.920	35.0	Cluster_03356

713.38000	7.720	8.060	35.0	Cluster_12431
713.72000	31.630	33.460	35.0	Cluster_12437
713.77000	7.320	7.800	35.0	Cluster_34987
713.92000	14.850	15.200	35.0	Cluster_34998
714.32000	17.390	17.720	35.0	Cluster_35004
714.36000	19.930	20.350	35.0	Cluster_35010
715.38000	19.960	20.320	35.0	Cluster_35041
715.54000	11.860	12.010	35.0	Cluster_00320
715.71000	14.660	14.920	35.0	Cluster_12456
715.85000	17.470	17.620	35.0	Cluster_35046
715.99000	31.390	31.720	35.0	Cluster_12465
716.39000	19.880	20.360	35.0	Cluster_35061
717.36000	31.540	32.090	35.0	Cluster_12478
717.90000	25.120	25.550	35.0	Cluster_35102
718.36000	19.940	20.380	35.0	Cluster_35122
718.85000	7.680	8.400	35.0	Cluster_35127
718.89000	17.510	17.800	35.0	Cluster_35137
718.91000	27.800	28.330	35.0	Cluster_35135
718.96000	28.020	28.340	35.0	Cluster_12511
719.39000	19.980	20.260	35.0	Cluster_35145
719.83000	11.610	12.340	35.0	Cluster_35161
719.85000	25.100	25.670	35.0	Cluster_03411
719.98000	19.940	20.400	35.0	Cluster_12519
720.12000	25.110	25.240	35.0	Cluster_35168
720.13000	28.000	28.180	35.0	Cluster_35170
720.35000	17.530	17.700	35.0	Cluster_03417
720.58000	17.300	17.590	35.0	Cluster_03421
720.83000	7.750	8.350	35.0	Cluster_35178
721.32000	19.890	20.270	35.0	Cluster_35195
721.41000	17.260	17.540	35.0	Cluster_35191
721.72000	22.640	22.890	35.0	Cluster_12548
721.88000	22.670	23.060	35.0	Cluster_35209
721.90000	25.050	25.270	35.0	Cluster_35213
722.02000	31.660	32.060	35.0	Cluster_12553
722.09000	28.070	28.500	35.0	Cluster_03436
722.34000	25.130	26.370	35.0	Cluster_03433
722.68000	31.370	32.020	35.0	Cluster_12565
723.40000	19.960	20.360	35.0	Cluster_12568
723.42000	22.560	22.940	35.0	Cluster_12570
723.95000	27.910	28.520	35.0	Cluster_00326
724.04000	31.210	31.650	35.0	Cluster_12575
724.90000	22.470	22.960	35.0	Cluster_03465
725.06000	27.970	28.270	35.0	Cluster_12593
725.32000	7.780	8.500	35.0	Cluster_03469
725.41000	17.370	17.560	35.0	Cluster_35286
725.42000	31.360	31.600	35.0	Cluster_35293
727.38000	25.090	25.480	35.0	Cluster_35317
728.10000	25.290	25.660	35.0	Cluster_03487
728.13000	22.560	22.730	35.0	Cluster_03484
728.42000	25.140	25.320	35.0	Cluster_35338
728.98000	31.520	31.690	35.0	Cluster_12653

729.15000	31.210	31.440	35.0	Cluster_03497
729.84000	17.500	17.710	35.0	Cluster_35373
729.89000	22.500	22.710	35.0	Cluster_03507
730.91000	22.680	22.870	35.0	Cluster_35408
731.10000	28.060	28.490	35.0	Cluster_35410
731.83000	25.260	25.650	35.0	Cluster_03518
732.11000	17.410	17.790	35.0	Cluster_03522
732.64000	31.490	31.690	35.0	Cluster_03534
733.03000	17.410	17.820	35.0	Cluster_12708
733.13000	25.140	25.560	35.0	Cluster_03548
733.83000	11.880	12.390	35.0	Cluster_35471
733.85000	14.650	15.150	35.0	Cluster_35474
734.58000	7.790	8.040	35.0	Cluster_35493
735.04000	20.040	20.350	35.0	Cluster_12729
735.74000	17.500	17.780	35.0	Cluster_12741
736.37000	11.800	12.200	35.0	Cluster_35541
737.04000	27.800	28.310	35.0	Cluster_12754
737.16000	22.680	22.850	35.0	Cluster_35560
737.82000	7.630	8.050	35.0	Cluster_35593
738.39000	19.880	20.480	35.0	Cluster_35611
738.63000	11.820	12.200	35.0	Cluster_03590
739.38000	27.950	28.260	35.0	Cluster_12791
739.39000	27.810	27.990	35.0	Cluster_12789
739.84000	1.420	1.740	35.0	Cluster_35645
740.37000	14.720	15.210	35.0	Cluster_35657
740.38000	22.620	22.940	35.0	Cluster_35661
740.93000	27.860	28.230	35.0	Cluster_35676
741.85000	17.470	17.680	35.0	Cluster_35683
742.37000	11.870	12.390	35.0	Cluster_35697
742.67000	7.750	8.360	35.0	Cluster_12831
742.84000	14.750	15.520	35.0	Cluster_35707
744.37000	25.210	25.740	35.0	Cluster_35750
745.38000	25.200	25.420	35.0	Cluster_35777
745.68000	22.680	23.580	35.0	Cluster_12857
746.06000	22.680	22.970	35.0	Cluster_12863
746.15000	28.030	28.180	35.0	Cluster_35792
746.30000	7.780	8.080	35.0	Cluster_35797
747.12000	11.800	12.230	35.0	Cluster_35817
747.37000	11.710	12.180	35.0	Cluster_35818
748.00000	22.610	22.950	35.0	Cluster_12903
748.86000	27.800	28.150	35.0	Cluster_03654
748.88000	19.970	20.640	35.0	Cluster_35857
749.07000	19.960	20.200	35.0	Cluster_12920
749.69000	17.250	17.550	35.0	Cluster_12929
749.98000	19.860	20.090	35.0	Cluster_12936
750.01000	22.600	23.500	35.0	Cluster_12941
750.38000	14.680	14.960	35.0	Cluster_35883
750.44000	22.600	22.990	35.0	Cluster_35886
750.94000	22.590	23.000	35.0	Cluster_35903
751.06000	28.050	28.290	35.0	Cluster_12961
751.15000	25.250	25.670	35.0	Cluster_03662

751.36000	14.640	15.100	35.0	Cluster_35908
752.37000	11.840	12.380	35.0	Cluster_35932
753.05000	31.680	31.970	35.0	Cluster_12988
753.06000	25.170	25.500	35.0	Cluster_12984
753.39000	22.670	22.900	35.0	Cluster_35964
753.91000	27.920	28.160	35.0	Cluster_35981
754.06000	20.050	20.510	35.0	Cluster_13009
754.90000	14.660	15.500	35.0	Cluster_36004
755.06000	25.260	25.460	35.0	Cluster_13021
755.30000	20.050	20.270	35.0	Cluster_13019
755.33000	22.630	23.190	35.0	Cluster_13020
755.42000	19.910	20.150	35.0	Cluster_36013
755.75000	17.360	17.740	35.0	Cluster_13034
755.92000	19.960	20.150	35.0	Cluster_36043
756.13000	25.270	25.620	35.0	Cluster_03688
757.31000	14.830	14.950	35.0	Cluster_36071
757.73000	27.890	28.230	35.0	Cluster_13076
758.41000	25.080	25.600	35.0	Cluster_36102
758.70000	14.580	14.990	35.0	Cluster_13088
759.63000	22.600	22.980	35.0	Cluster_03704
759.90000	14.610	14.870	35.0	Cluster_36129
759.91000	17.320	17.630	35.0	Cluster_36132
760.35000	14.800	15.030	35.0	Cluster_36146
761.05000	17.490	17.830	35.0	Cluster_00024
761.41000	17.280	17.520	35.0	Cluster_36182
762.38000	27.960	28.200	35.0	Cluster_13130
763.03000	7.560	9.080	35.0	Cluster_13141
763.08000	17.400	17.840	35.0	Cluster_13137
763.35000	25.210	26.120	35.0	Cluster_03726
763.71000	31.480	31.660	35.0	Cluster_13147
764.09000	31.660	32.060	35.0	Cluster_13148
764.36000	11.800	12.080	35.0	Cluster_36253
765.15000	31.680	31.980	35.0	Cluster_03741
765.79000	11.610	11.860	35.0	Cluster_36274
765.80000	11.860	12.080	35.0	Cluster_36275
766.37000	11.600	11.750	35.0	Cluster_36292
766.55000	17.450	18.040	35.0	Cluster_00339
767.70000	27.940	28.270	35.0	Cluster_13203
767.85000	11.790	12.130	35.0	Cluster_36325
768.07000	17.280	17.780	35.0	Cluster_13209
768.08000	31.360	31.640	35.0	Cluster_13205
768.38000	7.480	7.660	35.0	Cluster_36351
769.33000	22.640	22.980	35.0	Cluster_36370
769.59000	19.930	20.190	35.0	Cluster_36368
769.82000	19.960	20.600	35.0	Cluster_36385
772.41000	22.650	23.180	35.0	Cluster_36441
772.74000	25.070	25.310	35.0	Cluster_13265
772.80000	31.680	32.590	35.0	Cluster_13273
772.88000	14.580	14.710	35.0	Cluster_36459
772.92000	14.670	14.950	35.0	Cluster_36460
773.08000	19.950	20.220	35.0	Cluster_03785

773.09000	20.050	20.330	35.0	Cluster_03786
773.13000	31.430	31.710	35.0	Cluster_13272
773.35000	14.740	15.130	35.0	Cluster_13283
773.41000	27.880	28.470	35.0	Cluster_13286
774.12000	20.050	20.350	35.0	Cluster_36481
774.73000	17.470	18.010	35.0	Cluster_13306
775.06000	11.690	12.150	35.0	Cluster_13312
775.07000	19.940	20.600	35.0	Cluster_13311
775.42000	27.800	28.440	35.0	Cluster_36510
776.37000	22.480	22.750	35.0	Cluster_03803
777.15000	27.810	28.220	35.0	Cluster_03806
777.35000	11.810	12.190	35.0	Cluster_36561
777.38000	25.120	25.450	35.0	Cluster_36567
777.39000	14.650	14.840	35.0	Cluster_36564
778.37000	17.250	17.390	35.0	Cluster_36591
778.77000	25.180	25.660	35.0	Cluster_13350
778.87000	17.380	17.700	35.0	Cluster_36605
780.73000	27.980	28.270	35.0	Cluster_13379
780.81000	19.910	20.480	35.0	Cluster_36636
780.88000	31.460	32.210	35.0	Cluster_03821
781.40000	31.410	31.620	35.0	Cluster_36661
782.03000	11.610	12.170	35.0	Cluster_13403
783.13000	22.500	22.860	35.0	Cluster_03834
783.46000	25.110	25.420	35.0	Cluster_36708
783.72000	19.910	20.190	35.0	Cluster_13422
784.06000	27.900	28.250	35.0	Cluster_13433
784.89000	1.480	1.760	35.0	Cluster_36733
784.93000	25.120	25.800	35.0	Cluster_36732
785.45000	19.890	20.320	35.0	Cluster_36744
785.83000	22.660	22.780	35.0	Cluster_36763
787.06000	28.010	28.200	35.0	Cluster_13459
787.12000	22.530	22.900	35.0	Cluster_03868
787.35000	19.870	20.400	35.0	Cluster_03870
788.87000	22.600	22.810	35.0	Cluster_36840
789.06000	31.630	32.000	35.0	Cluster_13483
790.87000	31.560	32.090	35.0	Cluster_36906
791.12000	22.590	22.780	35.0	Cluster_36909
791.38000	25.170	25.540	35.0	Cluster_36914
791.90000	27.890	28.500	35.0	Cluster_36919
792.38000	31.390	31.540	35.0	Cluster_36933
792.64000	17.450	17.820	35.0	Cluster_36941
792.86000	22.690	23.220	35.0	Cluster_03914
792.87000	31.460	31.670	35.0	Cluster_36948
792.97000	14.580	14.860	35.0	Cluster_13541
793.78000	31.320	31.590	35.0	Cluster_13547
794.36000	31.670	32.080	35.0	Cluster_36984
794.37000	19.940	20.210	35.0	Cluster_36982
794.45000	1.420	1.790	35.0	Cluster_36985
794.65000	17.390	17.780	35.0	Cluster_36986
795.07000	25.160	25.360	35.0	Cluster_13571
795.42000	22.670	22.790	35.0	Cluster_37004

795.44000	1.400	1.800	35.0	Cluster_37000
795.68000	14.590	14.880	35.0	Cluster_13585
795.87000	31.560	32.170	35.0	Cluster_03947
795.90000	28.060	28.270	35.0	Cluster_37017
795.96000	14.760	15.070	35.0	Cluster_37020
796.41000	31.450	31.680	35.0	Cluster_13605
796.72000	19.940	20.100	35.0	Cluster_13597
796.91000	22.500	22.720	35.0	Cluster_37044
797.86000	19.930	20.440	35.0	Cluster_37077
797.87000	7.430	7.730	35.0	Cluster_37073
797.88000	7.310	7.440	35.0	Cluster_37072
797.89000	22.500	22.690	35.0	Cluster_37068
799.38000	22.500	23.460	35.0	Cluster_37115
799.40000	14.770	15.530	35.0	Cluster_37112
799.89000	31.620	32.180	35.0	Cluster_03975
800.35000	31.530	31.630	35.0	Cluster_37146
800.41000	19.890	20.120	35.0	Cluster_13643
800.84000	31.290	31.550	35.0	Cluster_37149
800.86000	31.650	32.090	35.0	Cluster_03991
801.06000	11.650	12.070	35.0	Cluster_00029
801.37000	17.480	17.810	35.0	Cluster_03995
801.42000	25.180	25.660	35.0	Cluster_37163
801.44000	31.270	31.660	35.0	Cluster_13660
802.43000	20.050	20.550	35.0	Cluster_37188
802.74000	27.900	28.080	35.0	Cluster_13681
803.41000	25.130	25.600	35.0	Cluster_13684
803.88000	17.370	17.770	35.0	Cluster_37226
804.43000	22.650	22.870	35.0	Cluster_37243
804.91000	31.690	32.140	35.0	Cluster_04015
805.16000	14.600	14.940	35.0	Cluster_37269
805.39000	27.930	28.290	35.0	Cluster_37283
805.94000	31.440	31.630	35.0	Cluster_37284
806.39000	14.640	14.860	35.0	Cluster_37304
807.01000	19.980	20.440	35.0	Cluster_13723
807.46000	19.920	20.080	35.0	Cluster_37330
807.92000	27.870	28.030	35.0	Cluster_37352
808.85000	19.960	20.450	35.0	Cluster_37370
808.91000	31.470	31.730	35.0	Cluster_37377
809.40000	22.660	23.070	35.0	Cluster_04037
809.91000	14.850	15.310	35.0	Cluster_37401
810.39000	19.920	20.390	35.0	Cluster_37407
810.44000	22.630	23.110	35.0	Cluster_13774
811.74000	22.480	22.860	35.0	Cluster_00036
811.86000	20.040	20.560	35.0	Cluster_37446
811.91000	14.870	15.290	35.0	Cluster_37441
812.39000	22.630	22.830	35.0	Cluster_37458
812.41000	27.930	28.400	35.0	Cluster_13796
813.82000	11.790	12.190	35.0	Cluster_00352
813.90000	17.360	17.730	35.0	Cluster_37483
814.10000	25.250	25.470	35.0	Cluster_13827
814.16000	27.890	28.190	35.0	Cluster_37492

814.17000	25.120	25.320	35.0	Cluster_04067
814.90000	22.680	23.030	35.0	Cluster_04074
815.06000	20.000	20.430	35.0	Cluster_13832
815.79000	31.570	31.790	35.0	Cluster_13844
815.92000	31.670	31.980	35.0	Cluster_37530
816.38000	11.830	12.230	35.0	Cluster_37540
816.43000	17.290	17.640	35.0	Cluster_04088
816.72000	25.320	25.810	35.0	Cluster_13857
817.34000	17.250	17.390	35.0	Cluster_37561
817.88000	17.360	17.810	35.0	Cluster_37570
818.42000	14.620	15.040	35.0	Cluster_13883
819.05000	25.150	26.050	35.0	Cluster_13897
819.42000	27.910	28.360	35.0	Cluster_13906
820.17000	31.380	32.010	35.0	Cluster_04104
821.23000	31.570	34.850	35.0	Cluster_04111
821.38000	17.280	17.680	35.0	Cluster_37637
822.06000	19.930	20.470	35.0	Cluster_13934
823.42000	17.480	17.870	35.0	Cluster_37669
823.91000	19.880	20.220	35.0	Cluster_37674
823.94000	31.630	31.990	35.0	Cluster_37688
825.32000	17.260	17.390	35.0	Cluster_37717
825.39000	31.380	31.780	35.0	Cluster_04137
826.38000	25.130	25.960	35.0	Cluster_13981
826.86000	7.610	8.080	35.0	Cluster_37765
827.04000	25.110	25.810	35.0	Cluster_13986
827.06000	25.130	25.350	35.0	Cluster_13988
827.09000	27.900	28.200	35.0	Cluster_13989
827.88000	17.410	17.760	35.0	Cluster_37794
828.80000	31.260	31.860	35.0	Cluster_14000
828.98000	11.690	11.970	35.0	Cluster_14004
829.16000	28.030	28.450	35.0	Cluster_04165
829.36000	17.310	17.660	35.0	Cluster_37821
829.43000	1.390	1.780	35.0	Cluster_37825
829.91000	31.400	31.980	35.0	Cluster_04169
830.74000	25.120	25.400	35.0	Cluster_14020
830.89000	17.440	17.890	35.0	Cluster_00042
831.45000	22.630	22.880	35.0	Cluster_37853
832.41000	19.980	20.380	35.0	Cluster_37873
832.68000	27.950	28.150	35.0	Cluster_37879
833.49000	25.150	25.550	35.0	Cluster_37894
833.99000	25.080	25.180	35.0	Cluster_37902
834.10000	27.890	28.190	35.0	Cluster_14054
834.94000	17.250	17.590	35.0	Cluster_37918
835.05000	11.600	12.000	35.0	Cluster_14062
836.39000	25.120	25.370	35.0	Cluster_14076
836.42000	28.040	28.340	35.0	Cluster_14077
836.76000	7.520	8.200	35.0	Cluster_14083
836.86000	17.310	17.800	35.0	Cluster_37959
836.94000	31.630	32.340	35.0	Cluster_04234
837.41000	31.230	31.570	35.0	Cluster_37970
837.95000	25.170	25.490	35.0	Cluster_37976

839.10000	31.510	32.010	35.0	Cluster_14102
840.66000	31.220	31.560	35.0	Cluster_04260
840.78000	25.110	25.290	35.0	Cluster_14117
841.08000	22.570	22.840	35.0	Cluster_14128
841.10000	19.960	20.190	35.0	Cluster_14126
841.67000	25.300	25.610	35.0	Cluster_00364
841.76000	27.960	28.220	35.0	Cluster_38066
842.73000	25.140	25.740	35.0	Cluster_14140
843.87000	17.390	17.890	35.0	Cluster_38114
844.18000	31.530	32.150	35.0	Cluster_04298
844.44000	25.120	25.510	35.0	Cluster_14157
846.98000	1.510	1.880	35.0	Cluster_38180
847.48000	1.390	1.840	35.0	Cluster_38200
847.75000	19.880	20.160	35.0	Cluster_14185
847.88000	11.560	11.920	35.0	Cluster_38203
848.40000	27.880	28.510	35.0	Cluster_04350
848.91000	31.520	32.250	35.0	Cluster_04352
849.46000	25.150	25.670	35.0	Cluster_38235
849.64000	27.920	28.360	35.0	Cluster_04364
849.97000	22.620	23.510	35.0	Cluster_38245
849.99000	25.120	25.700	35.0	Cluster_38248
850.33000	14.660	14.780	35.0	Cluster_38250
850.40000	19.990	20.460	35.0	Cluster_04376
850.76000	19.870	20.160	35.0	Cluster_14213
851.36000	11.750	12.340	35.0	Cluster_14215
851.77000	31.300	31.540	35.0	Cluster_38298
852.03000	22.650	23.290	35.0	Cluster_38307
852.91000	22.610	23.310	35.0	Cluster_04400
852.93000	31.510	32.160	35.0	Cluster_04401
853.45000	20.010	20.160	35.0	Cluster_38334
854.44000	20.050	20.750	35.0	Cluster_38377
854.47000	22.680	23.080	35.0	Cluster_38375
854.88000	22.650	22.930	35.0	Cluster_38385
855.41000	27.900	28.200	35.0	Cluster_38392
855.73000	17.250	17.700	35.0	Cluster_14240
856.43000	25.110	25.490	35.0	Cluster_14249
856.91000	31.520	32.060	35.0	Cluster_04444
857.06000	17.480	17.920	35.0	Cluster_14257
857.89000	14.810	15.530	35.0	Cluster_38459
857.91000	27.910	28.720	35.0	Cluster_04449
858.07000	11.840	12.350	35.0	Cluster_14269
858.77000	25.070	25.380	35.0	Cluster_14275
859.45000	17.420	18.280	35.0	Cluster_38500
859.47000	7.790	8.140	35.0	Cluster_38497
860.47000	31.240	31.540	35.0	Cluster_14284
861.39000	27.870	28.200	35.0	Cluster_04493
862.39000	22.610	23.110	35.0	Cluster_04505
862.49000	25.140	25.580	35.0	Cluster_38591
863.05000	14.600	15.530	35.0	Cluster_14302
863.76000	22.540	23.030	35.0	Cluster_14307
863.94000	25.070	25.410	35.0	Cluster_38615

864.07000	14.650	14.990	35.0	Cluster_14314
865.03000	17.390	17.520	35.0	Cluster_38646
865.75000	25.320	25.950	35.0	Cluster_14326
865.92000	7.380	8.040	35.0	Cluster_38659
865.93000	25.050	25.440	35.0	Cluster_04555
866.41000	25.100	25.470	35.0	Cluster_14334
869.07000	25.170	26.260	35.0	Cluster_14353
869.28000	1.610	1.970	35.0	Cluster_38719
869.41000	17.420	17.720	35.0	Cluster_04599
869.78000	25.260	25.530	35.0	Cluster_14363
871.17000	14.840	14.960	35.0	Cluster_38758
871.27000	1.620	1.910	35.0	Cluster_38759
871.44000	14.800	15.060	35.0	Cluster_38769
871.47000	31.540	31.790	35.0	Cluster_14388
872.27000	1.620	1.840	35.0	Cluster_38802
874.09000	25.150	25.530	35.0	Cluster_14414
874.26000	1.450	1.800	35.0	Cluster_38844
874.78000	17.480	17.850	35.0	Cluster_14424
875.28000	1.610	1.910	35.0	Cluster_38860
875.66000	14.760	15.120	35.0	Cluster_04698
876.08000	22.620	23.800	35.0	Cluster_14437
876.53000	22.590	22.860	35.0	Cluster_38895
877.38000	31.440	31.940	35.0	Cluster_14453
877.44000	25.130	25.990	35.0	Cluster_38909
878.48000	22.630	22.810	35.0	Cluster_38928
880.46000	25.120	25.320	35.0	Cluster_14483
881.96000	31.500	31.750	35.0	Cluster_38993
882.44000	14.860	15.200	35.0	Cluster_39008
882.94000	22.620	22.950	35.0	Cluster_39018
884.40000	14.610	15.200	35.0	Cluster_14528
884.66000	17.330	17.790	35.0	Cluster_39047
885.12000	25.300	25.650	35.0	Cluster_14544
885.68000	22.680	22.970	35.0	Cluster_04765
885.95000	28.020	28.260	35.0	Cluster_39078
886.43000	19.990	20.220	35.0	Cluster_39095
886.96000	17.420	18.080	35.0	Cluster_39099
887.52000	22.650	22.880	35.0	Cluster_39107
887.68000	27.940	28.230	35.0	Cluster_04781
887.74000	19.860	20.230	35.0	Cluster_14571
888.13000	25.110	25.970	35.0	Cluster_14577
888.40000	22.640	23.100	35.0	Cluster_14578
888.91000	14.830	15.120	35.0	Cluster_39135
888.92000	22.520	22.680	35.0	Cluster_04786
889.11000	20.050	20.460	35.0	Cluster_14581
889.44000	1.430	1.770	35.0	Cluster_39147
890.17000	11.610	11.980	35.0	Cluster_04796
890.38000	17.520	18.070	35.0	Cluster_39161
890.39000	17.270	17.520	35.0	Cluster_39160
890.46000	22.600	22.960	35.0	Cluster_39173
890.99000	22.610	23.040	35.0	Cluster_39180
891.46000	25.300	25.500	35.0	Cluster_39186

891.81000	31.560	31.880	35.0	Cluster_14598
892.21000	14.840	15.360	35.0	Cluster_04824
893.43000	27.950	28.230	35.0	Cluster_39231
893.78000	19.930	20.280	35.0	Cluster_14615
894.12000	22.690	23.160	35.0	Cluster_14617
894.71000	27.820	28.230	35.0	Cluster_04845
895.21000	14.810	15.350	35.0	Cluster_04851
895.45000	25.300	25.980	35.0	Cluster_14622
895.81000	27.910	28.270	35.0	Cluster_14625
896.15000	31.390	31.880	35.0	Cluster_14634
897.04000	27.950	28.390	35.0	Cluster_39292
898.77000	22.550	22.980	35.0	Cluster_14664
899.43000	19.930	20.330	35.0	Cluster_39337
899.51000	1.420	1.800	35.0	Cluster_39345
899.96000	22.500	22.910	35.0	Cluster_04891
900.94000	14.600	15.030	35.0	Cluster_39368
900.97000	19.860	20.300	35.0	Cluster_39380
900.99000	1.400	1.920	35.0	Cluster_39377
902.46000	17.480	17.700	35.0	Cluster_39412
904.46000	31.430	31.860	35.0	Cluster_39443
905.43000	11.850	12.310	35.0	Cluster_39461
906.77000	31.320	31.760	35.0	Cluster_14739
907.80000	19.890	20.410	35.0	Cluster_14749
908.46000	19.880	20.220	35.0	Cluster_39500
908.48000	25.100	25.280	35.0	Cluster_14756
908.94000	25.120	25.750	35.0	Cluster_04958
908.95000	31.420	31.720	35.0	Cluster_39504
909.44000	20.020	20.410	35.0	Cluster_14760
909.50000	31.530	31.860	35.0	Cluster_14765
909.89000	17.400	17.650	35.0	Cluster_39520
910.11000	25.230	26.320	35.0	Cluster_14769
910.19000	27.860	28.480	35.0	Cluster_04966
911.96000	19.920	20.160	35.0	Cluster_39564
914.56000	27.860	28.530	35.0	Cluster_39615
915.13000	22.590	23.080	35.0	Cluster_14823
915.93000	27.880	28.130	35.0	Cluster_39626
916.44000	17.290	17.670	35.0	Cluster_39640
916.49000	19.900	20.670	35.0	Cluster_39633
917.45000	25.240	25.410	35.0	Cluster_14854
917.97000	31.670	31.940	35.0	Cluster_39674
918.66000	31.620	32.200	35.0	Cluster_39698
920.45000	19.920	20.220	35.0	Cluster_39721
920.76000	25.120	25.460	35.0	Cluster_14891
920.79000	1.550	1.670	35.0	Cluster_39725
920.88000	17.500	18.010	35.0	Cluster_39726
920.95000	22.500	22.690	35.0	Cluster_39731
922.42000	22.630	22.920	35.0	Cluster_14907
922.98000	25.090	25.510	35.0	Cluster_39772
923.30000	28.040	28.540	35.0	Cluster_14924
923.45000	25.290	25.740	35.0	Cluster_14932
923.92000	7.790	8.320	35.0	Cluster_39782

924.49000	28.040	28.700	35.0	Cluster_39788
925.11000	25.090	25.380	35.0	Cluster_14961
926.42000	31.250	31.670	35.0	Cluster_14975
927.48000	31.560	31.800	35.0	Cluster_39834
928.47000	19.990	20.340	35.0	Cluster_39858
928.96000	28.060	28.490	35.0	Cluster_39861
929.12000	31.300	31.890	35.0	Cluster_15009
929.43000	25.120	25.330	35.0	Cluster_39880
929.90000	17.420	17.750	35.0	Cluster_39897
931.73000	31.290	31.680	35.0	Cluster_15043
931.90000	14.590	15.170	35.0	Cluster_39924
932.64000	28.020	30.050	35.0	Cluster_15062
932.95000	20.060	20.220	35.0	Cluster_39941
933.14000	31.530	32.260	35.0	Cluster_15072
933.33000	25.110	25.530	35.0	Cluster_05038
934.00000	1.390	1.810	35.0	Cluster_39963
934.13000	25.040	25.480	35.0	Cluster_15084
934.43000	22.600	23.410	35.0	Cluster_15083
934.78000	27.990	28.710	35.0	Cluster_15089
935.78000	31.660	32.620	35.0	Cluster_15100
936.45000	20.050	20.400	35.0	Cluster_15116
936.80000	19.860	20.120	35.0	Cluster_15129
937.00000	17.430	17.980	35.0	Cluster_39997
937.44000	27.840	28.110	35.0	Cluster_15144
937.49000	31.290	31.910	35.0	Cluster_05051
938.07000	14.680	15.560	35.0	Cluster_15154
938.13000	28.060	28.400	35.0	Cluster_15156
938.21000	22.680	22.870	35.0	Cluster_15160
939.69000	20.000	20.360	35.0	Cluster_05058
940.46000	31.580	32.160	35.0	Cluster_15180
941.72000	17.260	17.790	35.0	Cluster_40098
942.00000	22.540	23.200	35.0	Cluster_40094
943.45000	31.330	31.630	35.0	Cluster_15229
945.13000	27.970	28.380	35.0	Cluster_15244
945.39000	14.670	14.920	35.0	Cluster_15242
945.72000	22.620	23.600	35.0	Cluster_05075
945.73000	19.890	20.380	35.0	Cluster_05078
945.78000	31.650	32.180	35.0	Cluster_15250
945.98000	25.310	25.950	35.0	Cluster_40152
947.16000	25.310	25.490	35.0	Cluster_15264
947.22000	31.420	31.860	35.0	Cluster_05084
947.79000	31.690	31.920	35.0	Cluster_15273
948.19000	27.930	28.220	35.0	Cluster_15277
948.52000	31.340	31.750	35.0	Cluster_40180
949.14000	31.350	31.630	35.0	Cluster_15301
949.44000	20.060	20.260	35.0	Cluster_40190
949.46000	31.610	31.940	35.0	Cluster_15302
950.44000	28.070	28.420	35.0	Cluster_15314
951.17000	22.650	22.880	35.0	Cluster_15324
951.96000	7.510	8.760	35.0	Cluster_40227
952.14000	28.060	28.500	35.0	Cluster_15337

952.15000	31.590	31.830	35.0	Cluster_15339
952.44000	31.610	32.570	35.0	Cluster_15342
952.53000	1.380	1.840	35.0	Cluster_40233
953.98000	19.900	20.100	35.0	Cluster_40255
954.82000	25.130	25.390	35.0	Cluster_15379
954.99000	19.910	20.370	35.0	Cluster_40262
955.13000	17.260	17.690	35.0	Cluster_15394
955.47000	25.300	25.650	35.0	Cluster_15404
956.48000	22.670	22.990	35.0	Cluster_15426
957.07000	11.840	12.000	35.0	Cluster_40290
957.48000	22.550	22.750	35.0	Cluster_40291
960.15000	27.830	28.270	35.0	Cluster_15462
960.98000	25.150	25.570	35.0	Cluster_40317
961.48000	25.120	25.340	35.0	Cluster_15478
962.14000	22.620	22.830	35.0	Cluster_15497
962.38000	17.250	17.410	35.0	Cluster_40344
962.79000	25.120	25.790	35.0	Cluster_15505
964.13000	25.130	25.570	35.0	Cluster_15523
964.43000	17.440	17.700	35.0	Cluster_40365
965.29000	1.640	1.850	35.0	Cluster_40377
966.15000	27.940	28.380	35.0	Cluster_15549
966.49000	17.440	17.820	35.0	Cluster_40387
966.50000	31.570	32.070	35.0	Cluster_05172
966.85000	25.130	25.470	35.0	Cluster_15552
967.00000	31.630	32.160	35.0	Cluster_40392
967.56000	22.680	23.030	35.0	Cluster_15565
968.03000	17.400	17.670	35.0	Cluster_40402
968.25000	25.220	25.360	35.0	Cluster_05187
971.97000	19.900	20.000	35.0	Cluster_40451
972.01000	19.890	20.440	35.0	Cluster_40467
972.46000	17.480	17.700	35.0	Cluster_15627
972.49000	22.660	22.870	35.0	Cluster_15626
973.51000	22.670	23.170	35.0	Cluster_40476
974.47000	28.050	28.200	35.0	Cluster_15651
974.98000	17.380	17.710	35.0	Cluster_40495
975.82000	17.380	17.850	35.0	Cluster_15672
975.98000	31.650	32.000	35.0	Cluster_05210
976.00000	22.650	23.000	35.0	Cluster_05212
976.03000	25.160	25.450	35.0	Cluster_40504
977.14000	27.990	28.200	35.0	Cluster_15696
977.62000	31.460	31.650	35.0	Cluster_40522
979.45000	22.690	23.050	35.0	Cluster_40546
979.80000	28.060	28.190	35.0	Cluster_15720
979.83000	31.360	32.110	35.0	Cluster_15721
979.98000	7.510	7.660	35.0	Cluster_40568
980.00000	17.300	17.710	35.0	Cluster_40562
980.50000	19.910	20.120	35.0	Cluster_40565
980.70000	14.830	15.050	35.0	Cluster_40578
981.45000	28.050	28.230	35.0	Cluster_15750
983.25000	20.060	20.280	35.0	Cluster_05237
983.95000	14.870	15.210	35.0	Cluster_40617

985.14000	22.650	22.870	35.0	Cluster_40628
986.02000	25.190	25.490	35.0	Cluster_40630
986.52000	1.390	1.800	35.0	Cluster_40639
986.83000	25.160	25.490	35.0	Cluster_15810
987.24000	19.890	20.200	35.0	Cluster_40654
987.80000	22.650	23.040	35.0	Cluster_15825
988.44000	17.480	17.670	35.0	Cluster_15835
991.93000	14.670	15.150	35.0	Cluster_40717
993.50000	22.650	22.790	35.0	Cluster_15895
997.19000	31.660	31.950	35.0	Cluster_15954
1000.06000	25.320	25.610	35.0	Cluster_40851
1001.03000	28.010	28.330	35.0	Cluster_40878
1001.16000	27.820	28.010	35.0	Cluster_15996
1001.49000	22.660	22.960	35.0	Cluster_40884
1003.52000	11.730	11.910	35.0	Cluster_05294
1004.00000	20.000	20.330	35.0	Cluster_40937
1004.31000	22.610	22.830	35.0	Cluster_05296
1004.57000	1.420	1.800	35.0	Cluster_40940
1007.81000	31.270	31.580	35.0	Cluster_16072
1008.78000	27.810	28.060	35.0	Cluster_16080
1012.26000	27.990	29.000	35.0	Cluster_05329
1012.27000	27.980	28.720	35.0	Cluster_41044
1012.93000	22.490	22.610	35.0	Cluster_41054
1013.01000	27.790	28.710	35.0	Cluster_05341
1013.47000	31.580	31.950	35.0	Cluster_16138
1013.49000	31.460	31.600	35.0	Cluster_16137
1013.76000	25.100	25.600	35.0	Cluster_05344
1013.90000	27.810	27.940	35.0	Cluster_41056
1015.92000	31.650	31.810	35.0	Cluster_41088
1016.78000	11.730	12.300	35.0	Cluster_05351
1017.27000	31.660	31.940	35.0	Cluster_41099
1019.25000	25.060	25.510	35.0	Cluster_05360
1020.15000	19.920	20.310	35.0	Cluster_16210
1022.50000	31.700	32.310	35.0	Cluster_16231
1023.82000	1.650	1.960	35.0	Cluster_41185
1024.48000	17.530	17.820	35.0	Cluster_16254
1024.50000	22.590	22.900	35.0	Cluster_16256
1024.93000	17.520	17.710	35.0	Cluster_41195
1025.46000	19.880	20.050	35.0	Cluster_16264
1025.81000	31.690	32.100	35.0	Cluster_41211
1026.83000	31.710	32.080	35.0	Cluster_16274
1027.13000	19.980	20.230	35.0	Cluster_16285
1027.14000	19.940	20.060	35.0	Cluster_16284
1027.88000	28.020	28.430	35.0	Cluster_16291
1028.18000	31.580	32.300	35.0	Cluster_16295
1028.84000	22.590	23.480	35.0	Cluster_16300
1029.99000	25.120	25.250	35.0	Cluster_41262
1031.20000	31.260	31.520	35.0	Cluster_16323
1032.82000	25.220	25.760	35.0	Cluster_16335
1033.20000	31.260	31.480	35.0	Cluster_16342
1036.52000	22.500	23.060	35.0	Cluster_16382

1037.50000	17.460	17.740	35.0	Cluster_16400
1039.05000	1.390	1.810	35.0	Cluster_41371
1039.52000	27.970	28.620	35.0	Cluster_41374
1039.81000	27.920	28.190	35.0	Cluster_16429
1040.83000	25.120	25.310	35.0	Cluster_05437
1041.01000	28.030	28.270	35.0	Cluster_41401
1042.17000	31.590	31.910	35.0	Cluster_16457
1044.49000	27.920	28.280	35.0	Cluster_41432
1046.13000	25.260	25.540	35.0	Cluster_16488
1046.22000	17.430	17.580	35.0	Cluster_16495
1047.16000	19.910	20.550	35.0	Cluster_16507
1047.83000	27.840	28.260	35.0	Cluster_41470
1053.50000	31.540	32.190	35.0	Cluster_16581
1053.98000	28.040	28.260	35.0	Cluster_41534
1054.99000	7.750	8.370	35.0	Cluster_05476
1055.22000	31.420	31.670	35.0	Cluster_16595
1055.50000	31.640	31.900	35.0	Cluster_16606
1056.52000	22.510	22.750	35.0	Cluster_16616
1057.06000	31.490	32.650	35.0	Cluster_41567
1057.33000	25.040	25.360	35.0	Cluster_05486
1057.34000	25.310	25.570	35.0	Cluster_05487
1057.58000	1.390	1.800	35.0	Cluster_41574
1057.99000	11.640	11.940	35.0	Cluster_41578
1058.02000	27.910	28.270	35.0	Cluster_41582
1058.81000	31.600	31.910	35.0	Cluster_16658
1058.85000	22.580	22.790	35.0	Cluster_16649
1058.96000	27.840	27.920	35.0	Cluster_41595
1058.97000	28.040	28.300	35.0	Cluster_41597
1059.01000	27.920	28.050	35.0	Cluster_41596
1060.32000	1.650	1.860	35.0	Cluster_41612
1060.75000	7.790	8.260	35.0	Cluster_05502
1060.82000	31.630	31.910	35.0	Cluster_16679
1061.26000	27.800	28.300	35.0	Cluster_05510
1061.33000	25.060	25.320	35.0	Cluster_05506
1061.82000	1.640	1.910	35.0	Cluster_41625
1062.83000	31.620	31.910	35.0	Cluster_16700
1063.27000	27.790	28.260	35.0	Cluster_05516
1063.82000	1.630	1.910	35.0	Cluster_41655
1063.99000	17.430	17.700	35.0	Cluster_41663
1064.72000	7.500	7.790	35.0	Cluster_05521
1064.73000	7.770	8.180	35.0	Cluster_41675
1064.99000	17.450	17.700	35.0	Cluster_41673
1066.26000	27.830	28.220	35.0	Cluster_41702
1067.52000	17.470	17.670	35.0	Cluster_41715
1068.05000	31.450	32.110	35.0	Cluster_41719
1068.75000	25.120	25.410	35.0	Cluster_41734
1069.51000	27.810	28.180	35.0	Cluster_41748
1070.16000	31.560	31.910	35.0	Cluster_16781
1070.51000	27.810	28.230	35.0	Cluster_05546
1072.55000	31.660	32.210	35.0	Cluster_16804
1072.76000	27.820	28.190	35.0	Cluster_05555

1074.19000	25.140	25.380	35.0	Cluster_16831
1074.51000	27.900	28.260	35.0	Cluster_41810
1075.53000	31.540	31.840	35.0	Cluster_41816
1076.75000	27.800	28.170	35.0	Cluster_05574
1078.01000	27.800	28.160	35.0	Cluster_05578
1079.49000	27.900	28.160	35.0	Cluster_05582
1080.75000	19.910	20.820	35.0	Cluster_00593
1082.00000	27.820	28.190	35.0	Cluster_05588
1083.50000	27.920	28.110	35.0	Cluster_05592
1086.59000	27.920	28.290	35.0	Cluster_41940
1091.58000	1.390	1.800	35.0	Cluster_41998
1092.90000	31.350	32.070	35.0	Cluster_17025
1093.21000	31.350	32.010	35.0	Cluster_17031
1093.55000	31.340	32.060	35.0	Cluster_17035
1093.58000	19.960	20.180	35.0	Cluster_42024
1093.99000	20.020	20.190	35.0	Cluster_42029
1094.92000	22.480	22.800	35.0	Cluster_00625
1096.55000	22.660	23.350	35.0	Cluster_42051
1100.23000	31.350	31.820	35.0	Cluster_17091
1100.56000	31.360	31.640	35.0	Cluster_17097
1101.96000	31.560	32.670	35.0	Cluster_17105
1102.54000	27.940	28.260	35.0	Cluster_17111
1103.12000	22.610	22.840	35.0	Cluster_42104
1108.23000	27.990	28.440	35.0	Cluster_17176
1108.37000	27.990	28.540	35.0	Cluster_42148
1108.88000	27.940	28.220	35.0	Cluster_17175
1109.86000	28.030	28.260	35.0	Cluster_17199
1113.50000	19.920	20.940	35.0	Cluster_42184
1113.53000	25.230	25.610	35.0	Cluster_17241
1115.18000	20.010	20.940	35.0	Cluster_17258
1115.77000	28.030	28.340	35.0	Cluster_00660
1117.22000	31.210	31.520	35.0	Cluster_17276
1118.21000	25.320	25.650	35.0	Cluster_17277
1118.22000	27.860	28.580	35.0	Cluster_17280
1119.87000	1.650	1.840	35.0	Cluster_42234
1121.54000	25.110	25.550	35.0	Cluster_17322
1123.04000	25.040	25.380	35.0	Cluster_42270
1123.97000	19.950	20.110	35.0	Cluster_42271
1124.25000	31.510	32.350	35.0	Cluster_17359
1125.57000	1.430	1.760	35.0	Cluster_42283
1126.07000	25.070	26.000	35.0	Cluster_42291
1127.88000	31.490	31.930	35.0	Cluster_17398
1128.57000	27.990	28.730	35.0	Cluster_17409
1129.21000	31.220	31.570	35.0	Cluster_17418
1129.55000	31.540	31.870	35.0	Cluster_17428
1129.90000	31.220	31.560	35.0	Cluster_17433
1130.23000	27.880	28.550	35.0	Cluster_17434
1131.58000	31.370	32.260	35.0	Cluster_17448
1133.13000	17.390	17.710	35.0	Cluster_42337
1133.20000	31.230	31.590	35.0	Cluster_17479
1133.22000	31.550	31.950	35.0	Cluster_17484

1134.90000	31.510	32.360	35.0	Cluster_17503
1135.23000	31.430	31.940	35.0	Cluster_17509
1135.51000	22.610	22.830	35.0	Cluster_17513
1135.54000	31.210	31.470	35.0	Cluster_17519
1135.55000	27.920	28.160	35.0	Cluster_17517
1136.11000	25.210	25.510	35.0	Cluster_42363
1136.88000	22.560	23.520	35.0	Cluster_17525
1136.89000	31.550	32.160	35.0	Cluster_17538
1138.13000	31.240	31.470	35.0	Cluster_42377
1140.22000	27.830	28.300	35.0	Cluster_17567
1143.18000	22.670	23.020	35.0	Cluster_00674
1143.56000	27.910	28.730	35.0	Cluster_17607
1143.60000	1.410	1.860	35.0	Cluster_42409
1144.20000	22.650	23.260	35.0	Cluster_17626
1144.24000	31.500	32.060	35.0	Cluster_17620
1145.21000	27.830	28.220	35.0	Cluster_17645
1146.57000	22.680	23.030	35.0	Cluster_00680
1149.29000	31.270	31.440	35.0	Cluster_42461
1151.05000	27.960	28.350	35.0	Cluster_42474
1152.54000	28.010	28.390	35.0	Cluster_17735
1152.88000	28.010	28.180	35.0	Cluster_17750
1153.03000	22.500	22.700	35.0	Cluster_05790
1153.76000	19.970	20.360	35.0	Cluster_00695
1154.35000	1.610	1.810	35.0	Cluster_42485
1155.34000	1.640	1.840	35.0	Cluster_42501
1156.84000	1.600	1.850	35.0	Cluster_42511
1158.84000	1.650	1.860	35.0	Cluster_42519
1159.61000	27.920	28.320	35.0	Cluster_42529
1160.34000	1.650	1.840	35.0	Cluster_42534
1162.11000	20.010	20.380	35.0	Cluster_42551
1162.64000	1.410	1.800	35.0	Cluster_42555
1163.09000	25.260	26.480	35.0	Cluster_05817
1166.08000	25.300	25.730	35.0	Cluster_05827
1166.14000	25.310	25.480	35.0	Cluster_42592
1166.21000	20.060	20.770	35.0	Cluster_17894
1167.19000	25.060	25.610	35.0	Cluster_17908
1167.57000	28.030	28.490	35.0	Cluster_17921
1168.21000	17.500	17.810	35.0	Cluster_17931
1168.33000	25.190	25.800	35.0	Cluster_42608
1168.58000	25.190	25.720	35.0	Cluster_05832
1173.07000	27.970	28.150	35.0	Cluster_42645
1180.80000	27.990	28.420	35.0	Cluster_05888
1183.23000	27.940	28.290	35.0	Cluster_18040
1187.59000	31.420	31.800	35.0	Cluster_18075
1190.56000	27.950	28.230	35.0	Cluster_18091
1192.81000	27.930	28.170	35.0	Cluster_42765
1192.92000	25.320	26.100	35.0	Cluster_18117
1195.88000	27.950	28.200	35.0	Cluster_18133
1196.63000	1.390	1.800	35.0	Cluster_42787
1197.59000	25.080	25.680	35.0	Cluster_18145
1197.90000	27.950	28.180	35.0	Cluster_18140

1198.64000	22.610	22.860	35.0	Cluster_42803
1199.32000	22.530	22.720	35.0	Cluster_05982
1199.57000	31.670	32.590	35.0	Cluster_18161
1200.26000	25.090	25.780	35.0	Cluster_18168
1200.56000	25.320	25.570	35.0	Cluster_18177
1201.89000	11.730	11.870	35.0	Cluster_18192
1203.44000	1.500	1.680	35.0	Cluster_42830
1206.62000	22.520	22.840	35.0	Cluster_42853
1208.56000	31.640	32.560	35.0	Cluster_18237
1209.51000	19.980	20.450	35.0	Cluster_42887
1213.12000	14.580	14.970	35.0	Cluster_42910
1215.15000	1.390	1.800	35.0	Cluster_42924
1218.11000	27.880	28.380	35.0	Cluster_42950
1219.09000	31.570	31.720	35.0	Cluster_42956
1221.25000	19.960	20.370	35.0	Cluster_18299
1222.10000	19.970	20.690	35.0	Cluster_42968
1224.08000	25.090	25.700	35.0	Cluster_42977
1224.60000	25.150	25.340	35.0	Cluster_42983
1225.59000	25.280	25.630	35.0	Cluster_42994
1228.07000	25.130	25.960	35.0	Cluster_43008
1228.11000	22.540	22.780	35.0	Cluster_43007
1230.63000	1.450	1.740	35.0	Cluster_43026
1232.57000	25.160	25.320	35.0	Cluster_43045
1234.58000	25.160	25.330	35.0	Cluster_43065
1238.57000	25.240	25.570	35.0	Cluster_43079
1245.28000	19.970	20.630	35.0	Cluster_18390
1249.16000	1.380	1.790	35.0	Cluster_43135
1251.87000	1.650	1.850	35.0	Cluster_43149
1253.87000	1.650	1.850	35.0	Cluster_43162
1255.86000	1.600	1.910	35.0	Cluster_43176
1257.08000	22.640	22.820	35.0	Cluster_06282
1264.27000	17.430	18.580	35.0	Cluster_18463
1267.20000	1.430	1.790	35.0	Cluster_43224
1267.68000	1.420	1.800	35.0	Cluster_43228
1274.29000	31.230	31.620	35.0	Cluster_18502
1284.15000	22.660	23.000	35.0	Cluster_43287
1300.69000	31.520	31.710	35.0	Cluster_43366
1300.70000	31.390	31.530	35.0	Cluster_43360
1300.73000	31.690	31.890	35.0	Cluster_43361
1300.89000	1.430	1.820	35.0	Cluster_43362
1301.68000	1.410	1.690	35.0	Cluster_43365
1320.21000	1.390	1.780	35.0	Cluster_43449
1329.73000	25.160	25.710	35.0	Cluster_43484
1330.45000	27.860	28.160	35.0	Cluster_06499
1331.69000	25.220	25.950	35.0	Cluster_43492
1334.68000	31.300	31.730	35.0	Cluster_43505
1349.89000	1.650	1.840	35.0	Cluster_43562
1353.71000	25.040	25.230	35.0	Cluster_43578
1354.21000	25.210	25.480	35.0	Cluster_43579
1354.66000	27.960	28.180	35.0	Cluster_06571
1361.03000	27.810	27.940	35.0	Cluster_18681

1367.16000	31.300	31.700	35.0	Cluster_43645
1372.75000	1.410	1.790	35.0	Cluster_43667
1376.18000	19.930	20.320	35.0	Cluster_43680
1376.34000	27.820	27.970	35.0	Cluster_18707
1383.67000	28.020	28.220	35.0	Cluster_18716
1394.65000	27.850	28.080	35.0	Cluster_43739
1396.44000	28.030	28.270	35.0	Cluster_18732
1402.12000	25.310	25.610	35.0	Cluster_18741
1406.24000	1.420	1.740	35.0	Cluster_43793
1407.03000	27.830	28.260	35.0	Cluster_18752
1414.35000	27.850	28.190	35.0	Cluster_18768
1417.37000	27.810	28.230	35.0	Cluster_18772
1420.01000	27.880	28.160	35.0	Cluster_18783
1422.96000	22.600	23.030	35.0	Cluster_43843
1423.47000	22.650	23.080	35.0	Cluster_43844
1424.69000	27.830	28.170	35.0	Cluster_18789
1424.79000	1.450	1.800	35.0	Cluster_43851
1425.27000	1.420	1.800	35.0	Cluster_43853
1427.03000	27.920	28.150	35.0	Cluster_18794
1428.82000	22.560	22.720	35.0	Cluster_43862
1429.02000	27.940	28.120	35.0	Cluster_18795
1430.01000	27.860	28.130	35.0	Cluster_18796
1431.68000	27.860	28.120	35.0	Cluster_18799
1437.03000	27.910	28.120	35.0	Cluster_18803
1439.35000	27.920	28.100	35.0	Cluster_18807
1459.27000	1.420	1.790	35.0	Cluster_43959
1477.30000	1.420	1.800	35.0	Cluster_44015
1489.22000	31.260	31.580	35.0	Cluster_44043
1492.94000	1.440	1.810	35.0	Cluster_44054
1495.14000	1.520	1.720	35.0	Cluster_44065
1511.80000	1.420	1.800	35.0	Cluster_44102
1518.72000	19.930	20.060	35.0	Cluster_44112
1530.33000	1.420	1.800	35.0	Cluster_44125
1560.76000	31.560	31.910	35.0	Cluster_44175
1564.32000	1.420	1.780	35.0	Cluster_44184
1571.76000	31.610	31.910	35.0	Cluster_44194
1582.85000	1.420	1.730	35.0	Cluster_44218
1616.85000	1.440	1.780	35.0	Cluster_44250
1633.97000	1.560	1.810	35.0	Cluster_44257
1635.40000	1.430	1.720	35.0	Cluster_44260
1676.82000	27.900	28.270	35.0	Cluster_44320
1683.84000	27.900	28.150	35.0	Cluster_44341
1687.92000	1.440	1.800	35.0	Cluster_44353
1739.94000	1.490	1.800	35.0	Cluster_44403
1792.99000	1.520	1.790	35.0	Cluster_44425

(-) Global MS/MS Masses:
(none)
Custom Data Dependent Settings:
Not enabled

Pass 8 (wheat-mixed-digests_MS2_TO-USE-inclusion-01_1.raw):

Creator: Orbi_30393
Last modified: 10/19/2021 by Orbi_30393
MS Run Time (min): 43.00
Sequence override of method parameters not enabled.
Divert Valve: not used during run
Contact Closure: not used during run
Syringe Pump: not used during run
MS Detector Settings:
Real-time modifications to method not enabled
Stepped collision energy not enabled
Additional Microscans:

MS2	0	0
MS3	0	0
MS4	0	0
MS5	0	0
MS6	0	0
MS7	0	0
MS8	0	0
MS9	0	0
MS10	0	0

Segment 1 Information
Duration (min): 43.00
Number of Scan Events: 2
Tune Method: Orbitrap-tune-file_2020-03-13_HESI
Scan Event Details:
1: FTMS + p norm res=15000 o(300.0-2000.0)
CV = 0.0V
2: ITMS + c norm Dep MS/MS Most intense ion from (1)
Activation Type: CID
Min. Signal Required: 500.0
Isolation Width: 2.00
Normalized Coll. Energy: 35.0
Default Charge State: 2
Activation Q: 0.250
Activation Time: 10.000
CV = 0.0V
Lock Masses:
Pos List Name: N/A
Source: API Source
Mass List: (none)
Neg List Name: N/A
Source: API Source
Mass List: (none)
Data Dependent Settings:
Use separate polarity settings disabled
Parent Mass List:

MS Mass	MS FAIMS CV	MS Normalized Collision Energy	MS2 Mass	MS2 Normalized Collision Energy	Name

300.13000	35.0	Cluster_06844
300.14000	35.0	Cluster_18928
300.71000	35.0	Cluster_18955
301.50000	35.0	Cluster_06851
301.65000	35.0	Cluster_18984
302.64000	35.0	Cluster_19016
302.65000	35.0	Cluster_19017
303.99000	35.0	Cluster_19040
304.11000	35.0	Cluster_19042
304.14000	35.0	Cluster_19049
304.16000	35.0	Cluster_19050
304.20000	35.0	Cluster_19052
304.49000	35.0	Cluster_06863
304.64000	35.0	Cluster_19062
306.13000	35.0	Cluster_06873
306.67000	35.0	Cluster_19109
307.12000	35.0	Cluster_19119
307.13000	35.0	Cluster_06880
308.65000	35.0	Cluster_19177
308.66000	35.0	Cluster_19179
309.53000	35.0	Cluster_06901
310.58000	35.0	Cluster_19217
310.65000	35.0	Cluster_19222
310.66000	35.0	Cluster_19221
311.17000	35.0	Cluster_19246
311.68000	35.0	Cluster_19257
313.65000	35.0	Cluster_19315
314.82000	35.0	Cluster_06941
315.65000	35.0	Cluster_19384
316.69000	35.0	Cluster_19442
317.06000	35.0	Cluster_19459
317.16000	35.0	Cluster_19466
317.64000	35.0	Cluster_19490
318.64000	35.0	Cluster_19532
318.85000	35.0	Cluster_06973
319.17000	35.0	Cluster_19552
319.67000	35.0	Cluster_19560
319.94000	35.0	Cluster_00850
320.49000	35.0	Cluster_06993
320.80000	35.0	Cluster_06994
321.15000	35.0	Cluster_06995
321.17000	35.0	Cluster_00869
321.52000	35.0	Cluster_19594
322.15000	35.0	Cluster_19619
322.17000	35.0	Cluster_19617
322.50000	35.0	Cluster_06998
323.65000	35.0	Cluster_19672
323.69000	35.0	Cluster_19677
323.83000	35.0	Cluster_19683
324.42000	35.0	Cluster_00878
324.60000	35.0	Cluster_19711

324.65000	35.0	Cluster_19713
324.83000	35.0	Cluster_07017
325.14000	35.0	Cluster_19730
325.82000	35.0	Cluster_07023
325.85000	35.0	Cluster_07028
326.17000	35.0	Cluster_19753
326.40000	35.0	Cluster_00887
326.90000	35.0	Cluster_00888
327.21000	35.0	Cluster_19791
327.96000	35.0	Cluster_07043
328.48000	35.0	Cluster_07051
329.18000	35.0	Cluster_19855
329.82000	35.0	Cluster_07063
329.93000	35.0	Cluster_19873
330.18000	35.0	Cluster_19886
330.90000	35.0	Cluster_19916
331.17000	35.0	Cluster_19926
331.20000	35.0	Cluster_19928
331.21000	35.0	Cluster_19930
331.22000	35.0	Cluster_19925
331.66000	35.0	Cluster_19936
332.14000	35.0	Cluster_19948
333.47000	35.0	Cluster_07081
333.82000	35.0	Cluster_07087
333.87000	35.0	Cluster_07090
335.12000	35.0	Cluster_20034
335.83000	35.0	Cluster_07104
336.19000	35.0	Cluster_07109
336.23000	35.0	Cluster_20093
336.91000	35.0	Cluster_00920
337.19000	35.0	Cluster_20132
337.21000	35.0	Cluster_20126
337.83000	35.0	Cluster_07126
338.11000	35.0	Cluster_20150
338.67000	35.0	Cluster_20172
339.16000	35.0	Cluster_20192
339.47000	35.0	Cluster_07139
339.81000	35.0	Cluster_07150
339.94000	35.0	Cluster_00929
340.18000	35.0	Cluster_20223
340.26000	35.0	Cluster_20229
341.15000	35.0	Cluster_20257
341.16000	35.0	Cluster_20261
342.16000	35.0	Cluster_07167
343.11000	35.0	Cluster_20316
343.18000	35.0	Cluster_20318
343.66000	35.0	Cluster_20344
345.19000	35.0	Cluster_20390
345.67000	35.0	Cluster_20402
346.15000	35.0	Cluster_07199
346.67000	35.0	Cluster_20444

347.20000	35.0	Cluster_20457
347.71000	35.0	Cluster_20471
348.44000	35.0	Cluster_20495
348.87000	35.0	Cluster_07234
348.92000	35.0	Cluster_00975
348.93000	35.0	Cluster_00976
349.20000	35.0	Cluster_20517
349.21000	35.0	Cluster_20520
349.64000	35.0	Cluster_20543
349.73000	35.0	Cluster_20549
350.42000	35.0	Cluster_00982
351.18000	35.0	Cluster_20611
351.19000	35.0	Cluster_20622
352.66000	35.0	Cluster_20669
353.17000	35.0	Cluster_01002
353.21000	35.0	Cluster_20705
353.66000	35.0	Cluster_01003
353.80000	35.0	Cluster_07278
353.87000	35.0	Cluster_20720
353.91000	35.0	Cluster_01004
354.16000	35.0	Cluster_20732
354.20000	35.0	Cluster_20739
354.40000	35.0	Cluster_01009
354.42000	35.0	Cluster_01011
355.14000	35.0	Cluster_20781
355.16000	35.0	Cluster_20782
355.22000	35.0	Cluster_20787
355.36000	35.0	Cluster_20789
355.70000	35.0	Cluster_20796
356.24000	35.0	Cluster_20824
356.55000	35.0	Cluster_07307
358.42000	35.0	Cluster_20933
359.13000	35.0	Cluster_07326
359.17000	35.0	Cluster_00118
359.37000	35.0	Cluster_20960
360.16000	35.0	Cluster_20986
360.42000	35.0	Cluster_01047
360.83000	35.0	Cluster_21008
361.18000	35.0	Cluster_21024
361.42000	35.0	Cluster_01055
361.66000	35.0	Cluster_21038
361.67000	35.0	Cluster_21036
361.69000	35.0	Cluster_21039
361.84000	35.0	Cluster_07347
362.10000	35.0	Cluster_21048
362.42000	35.0	Cluster_01059
362.43000	35.0	Cluster_21075
362.60000	35.0	Cluster_21077
362.65000	35.0	Cluster_21076
363.11000	35.0	Cluster_21089
363.19000	35.0	Cluster_21093

363.20000	35.0	Cluster_21094
363.77000	35.0	Cluster_21121
364.16000	35.0	Cluster_21135
364.50000	35.0	Cluster_21161
364.65000	35.0	Cluster_21162
365.24000	35.0	Cluster_21221
365.79000	35.0	Cluster_07396
365.90000	35.0	Cluster_21241
366.27000	35.0	Cluster_21267
366.74000	35.0	Cluster_21285
367.22000	35.0	Cluster_21306
367.50000	35.0	Cluster_07416
367.53000	35.0	Cluster_21324
368.16000	35.0	Cluster_21368
368.20000	35.0	Cluster_21366
368.47000	35.0	Cluster_07428
369.67000	35.0	Cluster_21437
370.08000	35.0	Cluster_21453
370.50000	35.0	Cluster_07445
370.74000	35.0	Cluster_21476
370.93000	35.0	Cluster_21486
371.18000	35.0	Cluster_07449
371.24000	35.0	Cluster_21501
371.71000	35.0	Cluster_21517
371.89000	35.0	Cluster_21531
372.17000	35.0	Cluster_21537
372.23000	35.0	Cluster_07464
372.67000	35.0	Cluster_21575
372.69000	35.0	Cluster_21572
373.12000	35.0	Cluster_21589
373.14000	35.0	Cluster_21585
373.16000	35.0	Cluster_07470
373.19000	35.0	Cluster_07471
373.22000	35.0	Cluster_21597
373.90000	35.0	Cluster_21628
374.19000	35.0	Cluster_07480
374.40000	35.0	Cluster_21650
374.68000	35.0	Cluster_21656
375.15000	35.0	Cluster_21668
375.89000	35.0	Cluster_07496
377.48000	35.0	Cluster_07512
377.77000	35.0	Cluster_21780
378.17000	35.0	Cluster_01122
378.18000	35.0	Cluster_21788
378.20000	35.0	Cluster_07523
378.67000	35.0	Cluster_01125
379.26000	35.0	Cluster_21841
380.45000	35.0	Cluster_21886
380.68000	35.0	Cluster_21891
381.22000	35.0	Cluster_07550
381.70000	35.0	Cluster_01141

382.18000	35.0	Cluster_21953
382.66000	35.0	Cluster_01144
383.63000	35.0	Cluster_22024
383.68000	35.0	Cluster_22025
384.16000	35.0	Cluster_01146
384.21000	35.0	Cluster_22055
384.22000	35.0	Cluster_22065
385.52000	35.0	Cluster_07597
385.54000	35.0	Cluster_07596
385.86000	35.0	Cluster_22123
386.66000	35.0	Cluster_22155
386.68000	35.0	Cluster_22158
386.90000	35.0	Cluster_22172
387.11000	35.0	Cluster_22177
387.63000	35.0	Cluster_22217
387.93000	35.0	Cluster_22226
388.23000	35.0	Cluster_22233
388.50000	35.0	Cluster_07621
388.64000	35.0	Cluster_22244
388.67000	35.0	Cluster_22245
388.83000	35.0	Cluster_07623
389.25000	35.0	Cluster_22269
389.51000	35.0	Cluster_07627
389.91000	35.0	Cluster_22284
390.71000	35.0	Cluster_22316
390.87000	35.0	Cluster_07651
390.88000	35.0	Cluster_07646
391.50000	35.0	Cluster_07654
391.65000	35.0	Cluster_22334
391.75000	35.0	Cluster_22344
391.82000	35.0	Cluster_07658
392.22000	35.0	Cluster_07665
392.25000	35.0	Cluster_22358
392.73000	35.0	Cluster_22381
392.77000	35.0	Cluster_22377
393.19000	35.0	Cluster_01195
393.53000	35.0	Cluster_07674
393.69000	35.0	Cluster_01197
393.71000	35.0	Cluster_22428
393.98000	35.0	Cluster_22479
394.21000	35.0	Cluster_22490
394.71000	35.0	Cluster_22522
394.72000	35.0	Cluster_22521
395.89000	35.0	Cluster_07703
396.23000	35.0	Cluster_22589
396.30000	35.0	Cluster_22595
397.39000	35.0	Cluster_22628
397.67000	35.0	Cluster_22634
397.72000	35.0	Cluster_22641
397.90000	35.0	Cluster_07736
398.18000	35.0	Cluster_01226

398.44000	35.0	Cluster_01230
400.24000	35.0	Cluster_22747
400.68000	35.0	Cluster_22780
400.90000	35.0	Cluster_07771
401.70000	35.0	Cluster_22833
402.32000	35.0	Cluster_22866
402.48000	35.0	Cluster_22868
402.72000	35.0	Cluster_22878
403.44000	35.0	Cluster_01260
403.54000	35.0	Cluster_07797
404.47000	35.0	Cluster_01272
405.88000	35.0	Cluster_07831
406.22000	35.0	Cluster_23023
406.68000	35.0	Cluster_01285
406.73000	35.0	Cluster_23046
406.95000	35.0	Cluster_01293
407.17000	35.0	Cluster_23059
407.25000	35.0	Cluster_23066
407.67000	35.0	Cluster_01302
407.75000	35.0	Cluster_23083
407.93000	35.0	Cluster_01303
408.22000	35.0	Cluster_23104
408.54000	35.0	Cluster_07863
408.66000	35.0	Cluster_01305
408.91000	35.0	Cluster_07864
408.92000	35.0	Cluster_01307
409.19000	35.0	Cluster_07867
409.25000	35.0	Cluster_23160
409.69000	35.0	Cluster_23170
410.53000	35.0	Cluster_07882
411.72000	35.0	Cluster_23258
412.67000	35.0	Cluster_23299
412.68000	35.0	Cluster_01326
413.23000	35.0	Cluster_23320
413.87000	35.0	Cluster_07913
413.92000	35.0	Cluster_07916
413.94000	35.0	Cluster_23350
413.98000	35.0	Cluster_23354
414.71000	35.0	Cluster_23379
414.85000	35.0	Cluster_23397
414.87000	35.0	Cluster_07924
415.22000	35.0	Cluster_23422
415.73000	35.0	Cluster_23434
416.00000	35.0	Cluster_23448
416.22000	35.0	Cluster_23463
416.76000	35.0	Cluster_23476
416.88000	35.0	Cluster_23482
416.94000	35.0	Cluster_01353
418.25000	35.0	Cluster_23534
418.47000	35.0	Cluster_23549
419.18000	35.0	Cluster_01360

419.21000	35.0	Cluster_07978
419.88000	35.0	Cluster_23600
420.19000	35.0	Cluster_23617
420.23000	35.0	Cluster_23612
420.71000	35.0	Cluster_23632
421.56000	35.0	Cluster_08005
422.12000	35.0	Cluster_08008
422.55000	35.0	Cluster_08014
422.58000	35.0	Cluster_08015
423.14000	35.0	Cluster_23727
423.21000	35.0	Cluster_08022
423.24000	35.0	Cluster_23740
423.44000	35.0	Cluster_01387
423.71000	35.0	Cluster_23753
423.74000	35.0	Cluster_23766
424.18000	35.0	Cluster_23787
424.51000	35.0	Cluster_23811
424.68000	35.0	Cluster_23813
425.18000	35.0	Cluster_23850
425.46000	35.0	Cluster_23865
425.87000	35.0	Cluster_08056
425.88000	35.0	Cluster_23895
426.14000	35.0	Cluster_23901
426.24000	35.0	Cluster_23924
426.44000	35.0	Cluster_23942
426.88000	35.0	Cluster_08077
427.51000	35.0	Cluster_08094
428.56000	35.0	Cluster_08107
428.72000	35.0	Cluster_24045
428.79000	35.0	Cluster_24059
429.24000	35.0	Cluster_24074
429.57000	35.0	Cluster_08120
429.72000	35.0	Cluster_24100
429.76000	35.0	Cluster_24093
429.85000	35.0	Cluster_08124
429.95000	35.0	Cluster_01432
430.73000	35.0	Cluster_24136
431.21000	35.0	Cluster_24172
431.41000	35.0	Cluster_24179
431.74000	35.0	Cluster_24194
432.16000	35.0	Cluster_24207
432.21000	35.0	Cluster_24215
432.87000	35.0	Cluster_08164
433.96000	35.0	Cluster_24289
434.79000	35.0	Cluster_24347
434.97000	35.0	Cluster_24350
435.17000	35.0	Cluster_24357
435.24000	35.0	Cluster_08201
435.37000	35.0	Cluster_24383
435.70000	35.0	Cluster_01481
435.97000	35.0	Cluster_01487

436.48000	35.0	Cluster_01491
437.37000	35.0	Cluster_24491
437.46000	35.0	Cluster_01497
438.22000	35.0	Cluster_24532
438.30000	35.0	Cluster_24543
438.96000	35.0	Cluster_01503
438.97000	35.0	Cluster_01504
439.82000	35.0	Cluster_24632
440.21000	35.0	Cluster_01512
440.56000	35.0	Cluster_24655
440.74000	35.0	Cluster_24659
440.86000	35.0	Cluster_08277
441.21000	35.0	Cluster_24682
441.24000	35.0	Cluster_01516
441.73000	35.0	Cluster_24703
441.87000	35.0	Cluster_08293
442.00000	35.0	Cluster_24716
442.28000	35.0	Cluster_24730
442.57000	35.0	Cluster_08303
442.77000	35.0	Cluster_24758
442.89000	35.0	Cluster_24763
442.98000	35.0	Cluster_24761
443.12000	35.0	Cluster_24771
443.22000	35.0	Cluster_24782
443.28000	35.0	Cluster_24785
443.29000	35.0	Cluster_24795
443.89000	35.0	Cluster_08322
443.92000	35.0	Cluster_08324
444.72000	35.0	Cluster_24866
445.18000	35.0	Cluster_08343
445.55000	35.0	Cluster_24900
446.27000	35.0	Cluster_24941
446.73000	35.0	Cluster_24959
446.91000	35.0	Cluster_08369
447.17000	35.0	Cluster_24974
447.25000	35.0	Cluster_08376
447.28000	35.0	Cluster_24982
447.29000	35.0	Cluster_24988
447.60000	35.0	Cluster_08380
448.30000	35.0	Cluster_25022
448.79000	35.0	Cluster_25051
448.97000	35.0	Cluster_01573
449.25000	35.0	Cluster_08416
449.74000	35.0	Cluster_25092
449.84000	35.0	Cluster_25102
450.23000	35.0	Cluster_25125
450.93000	35.0	Cluster_08435
451.14000	35.0	Cluster_25153
451.17000	35.0	Cluster_25168
451.57000	35.0	Cluster_08446
452.13000	35.0	Cluster_25195

452.26000	35.0	Cluster_08462
452.75000	35.0	Cluster_25235
453.14000	35.0	Cluster_25244
453.24000	35.0	Cluster_25248
454.68000	35.0	Cluster_25320
454.70000	35.0	Cluster_25319
454.74000	35.0	Cluster_25325
455.15000	35.0	Cluster_25343
455.18000	35.0	Cluster_01617
455.20000	35.0	Cluster_08518
456.24000	35.0	Cluster_25393
456.26000	35.0	Cluster_08536
456.57000	35.0	Cluster_08539
456.75000	35.0	Cluster_25404
456.78000	35.0	Cluster_25413
457.20000	35.0	Cluster_25422
457.75000	35.0	Cluster_25448
457.76000	35.0	Cluster_25454
458.69000	35.0	Cluster_25493
458.76000	35.0	Cluster_25500
459.22000	35.0	Cluster_08566
459.25000	35.0	Cluster_25514
459.78000	35.0	Cluster_25540
459.90000	35.0	Cluster_08580
460.27000	35.0	Cluster_25572
460.77000	35.0	Cluster_25581
460.97000	35.0	Cluster_01663
461.60000	35.0	Cluster_08592
461.70000	35.0	Cluster_25618
461.73000	35.0	Cluster_25620
462.22000	35.0	Cluster_08608
462.50000	35.0	Cluster_25643
463.61000	35.0	Cluster_25677
463.73000	35.0	Cluster_25692
464.22000	35.0	Cluster_08632
464.30000	35.0	Cluster_25714
464.55000	35.0	Cluster_08640
465.77000	35.0	Cluster_25807
465.95000	35.0	Cluster_08659
465.99000	35.0	Cluster_01692
466.27000	35.0	Cluster_25822
466.90000	35.0	Cluster_08673
467.18000	35.0	Cluster_08677
467.25000	35.0	Cluster_08678
467.30000	35.0	Cluster_25858
467.67000	35.0	Cluster_00144
467.72000	35.0	Cluster_25877
468.89000	35.0	Cluster_08711
469.26000	35.0	Cluster_08720
469.74000	35.0	Cluster_25967
469.88000	35.0	Cluster_08724

470.43000	35.0	Cluster_26002
470.56000	35.0	Cluster_00010
470.92000	35.0	Cluster_08745
471.00000	35.0	Cluster_01731
471.22000	35.0	Cluster_08748
471.25000	35.0	Cluster_01737
471.26000	35.0	Cluster_01738
471.48000	35.0	Cluster_26044
471.74000	35.0	Cluster_26063
471.84000	35.0	Cluster_26052
472.23000	35.0	Cluster_26081
472.24000	35.0	Cluster_08764
472.72000	35.0	Cluster_26107
472.76000	35.0	Cluster_26106
472.77000	35.0	Cluster_26113
473.27000	35.0	Cluster_26146
473.77000	35.0	Cluster_26157
474.54000	35.0	Cluster_08802
474.58000	35.0	Cluster_08800
474.85000	35.0	Cluster_08806
475.58000	35.0	Cluster_08820
475.78000	35.0	Cluster_26234
476.21000	35.0	Cluster_26244
476.23000	35.0	Cluster_08830
476.28000	35.0	Cluster_08832
477.07000	35.0	Cluster_26275
477.20000	35.0	Cluster_08843
477.27000	35.0	Cluster_08840
477.55000	35.0	Cluster_08851
477.61000	35.0	Cluster_08856
477.93000	35.0	Cluster_08861
478.22000	35.0	Cluster_26309
478.25000	35.0	Cluster_26313
478.76000	35.0	Cluster_26329
478.77000	35.0	Cluster_26332
478.93000	35.0	Cluster_08880
478.99000	35.0	Cluster_01780
479.57000	35.0	Cluster_08888
479.71000	35.0	Cluster_26429
479.76000	35.0	Cluster_26423
480.22000	35.0	Cluster_08900
480.70000	35.0	Cluster_26476
481.22000	35.0	Cluster_26493
481.27000	35.0	Cluster_26504
481.28000	35.0	Cluster_08920
481.56000	35.0	Cluster_08922
481.58000	35.0	Cluster_08924
481.72000	35.0	Cluster_26523
481.73000	35.0	Cluster_01804
481.79000	35.0	Cluster_26518
482.20000	35.0	Cluster_01810

482.28000	35.0	Cluster_26547
482.49000	35.0	Cluster_26549
483.24000	35.0	Cluster_26594
483.30000	35.0	Cluster_26603
483.76000	35.0	Cluster_26625
483.84000	35.0	Cluster_26627
484.09000	35.0	Cluster_26635
484.73000	35.0	Cluster_26685
484.76000	35.0	Cluster_26681
485.06000	35.0	Cluster_00166
485.54000	35.0	Cluster_08981
488.29000	35.0	Cluster_26837
488.61000	35.0	Cluster_09032
488.79000	35.0	Cluster_26859
488.95000	35.0	Cluster_09038
489.74000	35.0	Cluster_26904
489.93000	35.0	Cluster_09048
490.50000	35.0	Cluster_26931
490.70000	35.0	Cluster_26935
490.77000	35.0	Cluster_26939
492.55000	35.0	Cluster_09097
492.60000	35.0	Cluster_09099
493.27000	35.0	Cluster_27041
494.26000	35.0	Cluster_27100
495.19000	35.0	Cluster_27149
495.71000	35.0	Cluster_27170
495.95000	35.0	Cluster_09160
496.24000	35.0	Cluster_01895
496.95000	35.0	Cluster_09176
497.35000	35.0	Cluster_27258
497.74000	35.0	Cluster_27265
497.80000	35.0	Cluster_27275
497.94000	35.0	Cluster_09191
498.01000	35.0	Cluster_01929
498.50000	35.0	Cluster_27299
498.73000	35.0	Cluster_27302
499.23000	35.0	Cluster_09211
499.33000	35.0	Cluster_27324
499.76000	35.0	Cluster_01941
499.92000	35.0	Cluster_09218
499.96000	35.0	Cluster_09219
500.02000	35.0	Cluster_27355
500.63000	35.0	Cluster_09231
500.70000	35.0	Cluster_27401
500.96000	35.0	Cluster_01956
501.22000	35.0	Cluster_27436
501.59000	35.0	Cluster_09244
501.76000	35.0	Cluster_27459
502.21000	35.0	Cluster_01970
502.22000	35.0	Cluster_27473
502.59000	35.0	Cluster_09268

502.76000	35.0	Cluster_27485
503.25000	35.0	Cluster_27512
503.28000	35.0	Cluster_27518
504.21000	35.0	Cluster_27547
504.56000	35.0	Cluster_09288
504.98000	35.0	Cluster_01984
505.01000	35.0	Cluster_27567
505.24000	35.0	Cluster_09298
505.52000	35.0	Cluster_01990
505.60000	35.0	Cluster_09305
505.79000	35.0	Cluster_27593
506.73000	35.0	Cluster_27611
506.74000	35.0	Cluster_27621
506.76000	35.0	Cluster_27614
507.25000	35.0	Cluster_27638
507.97000	35.0	Cluster_09350
508.62000	35.0	Cluster_09366
508.70000	35.0	Cluster_27686
508.71000	35.0	Cluster_27692
509.20000	35.0	Cluster_27715
509.26000	35.0	Cluster_27718
509.87000	35.0	Cluster_09377
509.91000	35.0	Cluster_09383
511.23000	35.0	Cluster_27820
511.79000	35.0	Cluster_27852
511.91000	35.0	Cluster_09420
512.25000	35.0	Cluster_27867
512.27000	35.0	Cluster_27865
513.09000	35.0	Cluster_27898
513.77000	35.0	Cluster_27929
514.72000	35.0	Cluster_27964
514.74000	35.0	Cluster_27971
515.25000	35.0	Cluster_02053
515.48000	35.0	Cluster_02061
515.90000	35.0	Cluster_09478
516.74000	35.0	Cluster_28062
517.92000	35.0	Cluster_09503
518.00000	35.0	Cluster_28115
518.26000	35.0	Cluster_09508
518.31000	35.0	Cluster_28130
518.51000	35.0	Cluster_28138
518.59000	35.0	Cluster_09521
518.78000	35.0	Cluster_28152
519.59000	35.0	Cluster_09531
519.81000	35.0	Cluster_28200
520.04000	35.0	Cluster_28202
520.51000	35.0	Cluster_28225
520.80000	35.0	Cluster_28240
520.81000	35.0	Cluster_28232
521.02000	35.0	Cluster_02095
521.26000	35.0	Cluster_09553

521.66000	35.0	Cluster_28271
522.22000	35.0	Cluster_28286
522.45000	35.0	Cluster_02102
522.50000	35.0	Cluster_28303
522.62000	35.0	Cluster_09582
522.75000	35.0	Cluster_28315
523.22000	35.0	Cluster_28350
523.27000	35.0	Cluster_28334
523.35000	35.0	Cluster_28353
523.92000	35.0	Cluster_09603
523.97000	35.0	Cluster_28374
524.28000	35.0	Cluster_28386
526.18000	35.0	Cluster_28467
526.20000	35.0	Cluster_09656
526.93000	35.0	Cluster_09676
526.95000	35.0	Cluster_09677
527.76000	35.0	Cluster_28507
528.23000	35.0	Cluster_28536
528.27000	35.0	Cluster_00184
528.32000	35.0	Cluster_28539
528.36000	35.0	Cluster_28552
528.97000	35.0	Cluster_09710
529.25000	35.0	Cluster_09712
529.79000	35.0	Cluster_28615
530.20000	35.0	Cluster_28629
530.26000	35.0	Cluster_02153
530.51000	35.0	Cluster_28657
530.81000	35.0	Cluster_28677
530.94000	35.0	Cluster_09741
531.75000	35.0	Cluster_28707
531.77000	35.0	Cluster_28708
532.24000	35.0	Cluster_09763
532.27000	35.0	Cluster_09766
532.62000	35.0	Cluster_09768
532.97000	35.0	Cluster_09773
533.15000	35.0	Cluster_28752
533.16000	35.0	Cluster_28753
533.19000	35.0	Cluster_09774
533.32000	35.0	Cluster_09778
533.70000	35.0	Cluster_28783
533.82000	35.0	Cluster_28799
533.91000	35.0	Cluster_09792
534.28000	35.0	Cluster_28807
534.59000	35.0	Cluster_09803
535.32000	35.0	Cluster_09812
535.80000	35.0	Cluster_28867
535.94000	35.0	Cluster_09822
536.06000	35.0	Cluster_28878
536.26000	35.0	Cluster_09823
536.27000	35.0	Cluster_28888
536.48000	35.0	Cluster_28899

536.60000	35.0	Cluster_09834
537.23000	35.0	Cluster_09849
537.56000	35.0	Cluster_09853
537.69000	35.0	Cluster_28943
537.91000	35.0	Cluster_09869
538.84000	35.0	Cluster_28980
538.97000	35.0	Cluster_09886
538.98000	35.0	Cluster_09885
539.01000	35.0	Cluster_28985
539.28000	35.0	Cluster_28993
539.52000	35.0	Cluster_29001
540.02000	35.0	Cluster_09899
540.27000	35.0	Cluster_29031
540.79000	35.0	Cluster_29052
541.34000	35.0	Cluster_29085
541.44000	35.0	Cluster_29098
541.82000	35.0	Cluster_29115
542.26000	35.0	Cluster_02236
542.28000	35.0	Cluster_02229
542.52000	35.0	Cluster_02233
542.77000	35.0	Cluster_29162
543.27000	35.0	Cluster_29193
544.95000	35.0	Cluster_09982
545.77000	35.0	Cluster_29280
545.92000	35.0	Cluster_09992
546.31000	35.0	Cluster_29312
547.32000	35.0	Cluster_29345
548.27000	35.0	Cluster_29389
548.46000	35.0	Cluster_00194
548.61000	35.0	Cluster_10035
548.64000	35.0	Cluster_10037
549.25000	35.0	Cluster_10044
549.28000	35.0	Cluster_29418
549.90000	35.0	Cluster_10052
549.99000	35.0	Cluster_10058
550.22000	35.0	Cluster_29451
550.31000	35.0	Cluster_29461
550.55000	35.0	Cluster_10069
550.61000	35.0	Cluster_10068
550.78000	35.0	Cluster_29465
550.84000	35.0	Cluster_29476
551.01000	35.0	Cluster_29483
551.29000	35.0	Cluster_29499
551.57000	35.0	Cluster_10082
551.97000	35.0	Cluster_10089
552.36000	35.0	Cluster_29545
552.74000	35.0	Cluster_29553
552.75000	35.0	Cluster_29548
552.96000	35.0	Cluster_10104
553.55000	35.0	Cluster_29579
553.63000	35.0	Cluster_10116

554.58000	35.0	Cluster_10130
554.60000	35.0	Cluster_10136
554.97000	35.0	Cluster_10144
556.25000	35.0	Cluster_29692
556.68000	35.0	Cluster_10177
556.96000	35.0	Cluster_10184
557.73000	35.0	Cluster_29750
558.23000	35.0	Cluster_10209
558.65000	35.0	Cluster_10215
558.81000	35.0	Cluster_29792
559.29000	35.0	Cluster_29811
560.35000	35.0	Cluster_29845
560.65000	35.0	Cluster_10245
560.83000	35.0	Cluster_29862
561.28000	35.0	Cluster_29879
561.64000	35.0	Cluster_10262
561.70000	35.0	Cluster_29892
561.81000	35.0	Cluster_29901
561.98000	35.0	Cluster_10266
562.07000	35.0	Cluster_29906
562.29000	35.0	Cluster_29915
563.32000	35.0	Cluster_10288
563.50000	35.0	Cluster_02355
563.75000	35.0	Cluster_29975
563.76000	35.0	Cluster_29965
564.78000	35.0	Cluster_30003
564.79000	35.0	Cluster_02366
565.56000	35.0	Cluster_30039
565.60000	35.0	Cluster_10318
566.33000	35.0	Cluster_30080
566.74000	35.0	Cluster_02376
566.79000	35.0	Cluster_30088
566.90000	35.0	Cluster_30092
566.98000	35.0	Cluster_10333
567.07000	35.0	Cluster_30105
567.24000	35.0	Cluster_30110
567.30000	35.0	Cluster_30123
567.31000	35.0	Cluster_30114
568.56000	35.0	Cluster_02384
568.73000	35.0	Cluster_30166
568.79000	35.0	Cluster_30177
568.80000	35.0	Cluster_30176
568.85000	35.0	Cluster_30178
569.06000	35.0	Cluster_02386
569.57000	35.0	Cluster_02390
569.82000	35.0	Cluster_30204
570.30000	35.0	Cluster_02396
570.36000	35.0	Cluster_30219
570.73000	35.0	Cluster_02397
570.77000	35.0	Cluster_30236
571.22000	35.0	Cluster_30252

571.34000	35.0	Cluster_30256
571.64000	35.0	Cluster_10409
571.77000	35.0	Cluster_30264
572.13000	35.0	Cluster_00213
572.30000	35.0	Cluster_10420
572.79000	35.0	Cluster_30301
573.35000	35.0	Cluster_30317
574.26000	35.0	Cluster_10456
574.80000	35.0	Cluster_30355
575.25000	35.0	Cluster_02433
575.33000	35.0	Cluster_02435
576.55000	35.0	Cluster_02439
576.77000	35.0	Cluster_30417
576.98000	35.0	Cluster_10494
577.30000	35.0	Cluster_10500
577.81000	35.0	Cluster_30446
578.32000	35.0	Cluster_30460
578.55000	35.0	Cluster_02454
578.86000	35.0	Cluster_30486
578.98000	35.0	Cluster_10523
579.33000	35.0	Cluster_30494
579.55000	35.0	Cluster_02460
579.61000	35.0	Cluster_10531
579.63000	35.0	Cluster_10533
579.77000	35.0	Cluster_30512
579.81000	35.0	Cluster_30517
579.83000	35.0	Cluster_30515
580.36000	35.0	Cluster_30555
580.86000	35.0	Cluster_02466
581.28000	35.0	Cluster_02468
581.31000	35.0	Cluster_30577
581.96000	35.0	Cluster_10563
582.31000	35.0	Cluster_30648
582.54000	35.0	Cluster_02478
582.65000	35.0	Cluster_10574
582.70000	35.0	Cluster_00226
582.74000	35.0	Cluster_30654
584.05000	35.0	Cluster_30718
584.31000	35.0	Cluster_30724
584.54000	35.0	Cluster_30733
585.02000	35.0	Cluster_30763
585.29000	35.0	Cluster_10612
585.35000	35.0	Cluster_02501
585.80000	35.0	Cluster_30791
585.84000	35.0	Cluster_30792
586.62000	35.0	Cluster_10630
586.75000	35.0	Cluster_30815
587.30000	35.0	Cluster_10642
587.33000	35.0	Cluster_30848
587.34000	35.0	Cluster_30837
587.65000	35.0	Cluster_10646

588.69000	35.0	Cluster_30892
588.82000	35.0	Cluster_30900
589.55000	35.0	Cluster_30932
589.74000	35.0	Cluster_30933
589.96000	35.0	Cluster_10677
590.22000	35.0	Cluster_30952
590.30000	35.0	Cluster_30953
591.04000	35.0	Cluster_30998
591.44000	35.0	Cluster_31008
591.52000	35.0	Cluster_02538
591.66000	35.0	Cluster_10703
591.81000	35.0	Cluster_31027
592.01000	35.0	Cluster_10706
592.79000	35.0	Cluster_31056
592.82000	35.0	Cluster_31060
592.91000	35.0	Cluster_31063
593.06000	35.0	Cluster_02547
593.07000	35.0	Cluster_02548
593.33000	35.0	Cluster_10724
593.34000	35.0	Cluster_31073
593.93000	35.0	Cluster_10743
594.32000	35.0	Cluster_10753
595.59000	35.0	Cluster_31146
595.62000	35.0	Cluster_10769
596.75000	35.0	Cluster_31177
597.67000	35.0	Cluster_10808
597.81000	35.0	Cluster_31212
597.82000	35.0	Cluster_31208
598.29000	35.0	Cluster_10814
598.36000	35.0	Cluster_10818
599.36000	35.0	Cluster_31267
599.66000	35.0	Cluster_10840
599.95000	35.0	Cluster_10848
600.81000	35.0	Cluster_31316
600.97000	35.0	Cluster_10858
601.36000	35.0	Cluster_31339
602.32000	35.0	Cluster_31390
602.83000	35.0	Cluster_31406
603.30000	35.0	Cluster_31409
603.51000	35.0	Cluster_02598
603.53000	35.0	Cluster_31419
603.96000	35.0	Cluster_10888
604.80000	35.0	Cluster_31463
605.31000	35.0	Cluster_31473
605.67000	35.0	Cluster_10910
606.07000	35.0	Cluster_31501
606.27000	35.0	Cluster_31514
606.87000	35.0	Cluster_31531
606.89000	35.0	Cluster_10941
607.32000	35.0	Cluster_31536
608.09000	35.0	Cluster_02626

608.32000	35.0	Cluster_31587
608.49000	35.0	Cluster_31590
608.78000	35.0	Cluster_31595
608.79000	35.0	Cluster_31601
609.06000	35.0	Cluster_31605
609.81000	35.0	Cluster_31625
610.31000	35.0	Cluster_31655
611.34000	35.0	Cluster_31685
612.32000	35.0	Cluster_11010
613.83000	35.0	Cluster_31794
614.06000	35.0	Cluster_31796
614.07000	35.0	Cluster_02670
614.19000	35.0	Cluster_31797
614.26000	35.0	Cluster_11035
615.83000	35.0	Cluster_31852
616.08000	35.0	Cluster_02680
616.32000	35.0	Cluster_11075
616.55000	35.0	Cluster_31873
616.83000	35.0	Cluster_31882
617.02000	35.0	Cluster_31892
617.35000	35.0	Cluster_31911
617.93000	35.0	Cluster_31935
618.05000	35.0	Cluster_02693
618.79000	35.0	Cluster_31952
619.29000	35.0	Cluster_02701
619.33000	35.0	Cluster_31985
619.62000	35.0	Cluster_11116
620.53000	35.0	Cluster_32028
620.60000	35.0	Cluster_11128
621.30000	35.0	Cluster_32049
621.36000	35.0	Cluster_32053
621.39000	35.0	Cluster_32052
621.79000	35.0	Cluster_32057
621.98000	35.0	Cluster_11154
622.78000	35.0	Cluster_32095
622.98000	35.0	Cluster_11175
623.28000	35.0	Cluster_32112
623.36000	35.0	Cluster_32126
623.65000	35.0	Cluster_11191
623.98000	35.0	Cluster_11189
624.20000	35.0	Cluster_32153
624.28000	35.0	Cluster_32154
624.63000	35.0	Cluster_11202
625.04000	35.0	Cluster_02732
625.32000	35.0	Cluster_11203
625.58000	35.0	Cluster_02735
626.00000	35.0	Cluster_11225
626.07000	35.0	Cluster_32208
626.27000	35.0	Cluster_32218
626.28000	35.0	Cluster_32212
626.83000	35.0	Cluster_32244

627.30000	35.0	Cluster_32266
627.31000	35.0	Cluster_32263
627.50000	35.0	Cluster_32275
627.79000	35.0	Cluster_32283
628.07000	35.0	Cluster_32292
628.86000	35.0	Cluster_32321
629.63000	35.0	Cluster_11289
629.83000	35.0	Cluster_32344
629.96000	35.0	Cluster_11292
630.84000	35.0	Cluster_32375
631.37000	35.0	Cluster_11312
631.80000	35.0	Cluster_32410
632.05000	35.0	Cluster_11324
632.35000	35.0	Cluster_11329
632.78000	35.0	Cluster_32445
632.84000	35.0	Cluster_32450
633.00000	35.0	Cluster_11339
633.32000	35.0	Cluster_32465
633.36000	35.0	Cluster_32472
635.07000	35.0	Cluster_02772
635.82000	35.0	Cluster_02775
636.32000	35.0	Cluster_32584
636.84000	35.0	Cluster_32596
637.62000	35.0	Cluster_11403
637.83000	35.0	Cluster_32632
638.35000	35.0	Cluster_32655
638.36000	35.0	Cluster_11416
638.39000	35.0	Cluster_32659
638.79000	35.0	Cluster_32669
638.85000	35.0	Cluster_32673
639.85000	35.0	Cluster_32724
639.99000	35.0	Cluster_11446
640.32000	35.0	Cluster_32732
641.64000	35.0	Cluster_11472
641.84000	35.0	Cluster_32794
642.02000	35.0	Cluster_11476
643.07000	35.0	Cluster_32838
643.83000	35.0	Cluster_32863
644.00000	35.0	Cluster_11510
644.30000	35.0	Cluster_02836
644.36000	35.0	Cluster_32878
644.37000	35.0	Cluster_11511
645.00000	35.0	Cluster_11529
645.85000	35.0	Cluster_32926
646.30000	35.0	Cluster_32945
648.34000	35.0	Cluster_33005
648.67000	35.0	Cluster_11566
648.88000	35.0	Cluster_33029
649.01000	35.0	Cluster_11574
649.77000	35.0	Cluster_33054
650.31000	35.0	Cluster_33085

651.01000	35.0	Cluster_11595
651.08000	35.0	Cluster_33114
651.32000	35.0	Cluster_33124
651.36000	35.0	Cluster_11600
651.86000	35.0	Cluster_33158
651.99000	35.0	Cluster_11614
652.06000	35.0	Cluster_33159
652.35000	35.0	Cluster_33162
652.86000	35.0	Cluster_33187
653.33000	35.0	Cluster_33199
653.57000	35.0	Cluster_33207
653.60000	35.0	Cluster_02883
654.05000	35.0	Cluster_33221
654.68000	35.0	Cluster_11655
654.78000	35.0	Cluster_33237
655.99000	35.0	Cluster_11675
656.63000	35.0	Cluster_11676
657.90000	35.0	Cluster_33331
657.95000	35.0	Cluster_33350
658.07000	35.0	Cluster_33340
658.66000	35.0	Cluster_11699
659.28000	35.0	Cluster_33386
659.60000	35.0	Cluster_33396
659.63000	35.0	Cluster_02907
659.98000	35.0	Cluster_11725
660.33000	35.0	Cluster_33416
660.46000	35.0	Cluster_33423
660.84000	35.0	Cluster_33430
661.01000	35.0	Cluster_11741
661.31000	35.0	Cluster_33436
662.02000	35.0	Cluster_11748
662.12000	35.0	Cluster_02928
662.30000	35.0	Cluster_33472
663.37000	35.0	Cluster_33509
663.55000	35.0	Cluster_02941
663.60000	35.0	Cluster_02940
665.32000	35.0	Cluster_33570
666.35000	35.0	Cluster_02964
666.68000	35.0	Cluster_11814
666.95000	35.0	Cluster_33617
667.82000	35.0	Cluster_33644
668.33000	35.0	Cluster_33651
668.35000	35.0	Cluster_11848
669.10000	35.0	Cluster_02977
669.33000	35.0	Cluster_33680
669.78000	35.0	Cluster_33695
670.01000	35.0	Cluster_11876
670.40000	35.0	Cluster_33725
670.80000	35.0	Cluster_33734
670.82000	35.0	Cluster_33742
670.87000	35.0	Cluster_02986

671.44000	35.0	Cluster_33760
671.82000	35.0	Cluster_33770
671.97000	35.0	Cluster_11894
672.35000	35.0	Cluster_33781
672.81000	35.0	Cluster_33789
673.09000	35.0	Cluster_03005
673.37000	35.0	Cluster_33802
673.62000	35.0	Cluster_33824
673.65000	35.0	Cluster_11927
674.11000	35.0	Cluster_03014
674.32000	35.0	Cluster_11933
674.95000	35.0	Cluster_00274
675.37000	35.0	Cluster_33861
675.60000	35.0	Cluster_33864
675.64000	35.0	Cluster_03025
675.84000	35.0	Cluster_03026
676.00000	35.0	Cluster_11956
676.32000	35.0	Cluster_11959
677.20000	35.0	Cluster_33905
677.89000	35.0	Cluster_33946
678.81000	35.0	Cluster_33981
678.84000	35.0	Cluster_33980
678.85000	35.0	Cluster_03050
679.38000	35.0	Cluster_33996
679.79000	35.0	Cluster_34007
679.86000	35.0	Cluster_34018
680.08000	35.0	Cluster_03060
680.09000	35.0	Cluster_34023
680.87000	35.0	Cluster_34054
681.08000	35.0	Cluster_03066
681.32000	35.0	Cluster_12020
681.35000	35.0	Cluster_34074
682.09000	35.0	Cluster_34089
682.31000	35.0	Cluster_34106
682.38000	35.0	Cluster_03074
683.61000	35.0	Cluster_12059
683.81000	35.0	Cluster_34134
683.99000	35.0	Cluster_12062
684.08000	35.0	Cluster_03088
684.82000	35.0	Cluster_34171
684.83000	35.0	Cluster_34174
685.09000	35.0	Cluster_03095
685.10000	35.0	Cluster_03102
685.35000	35.0	Cluster_34201
686.04000	35.0	Cluster_12086
686.50000	35.0	Cluster_00285
686.75000	35.0	Cluster_00286
687.66000	35.0	Cluster_34255
689.34000	35.0	Cluster_34298
689.36000	35.0	Cluster_34295
689.57000	35.0	Cluster_03142

690.82000	35.0	Cluster_34324
690.84000	35.0	Cluster_34327
691.59000	35.0	Cluster_03162
691.79000	35.0	Cluster_34357
691.83000	35.0	Cluster_34369
692.26000	35.0	Cluster_34373
692.32000	35.0	Cluster_03167
692.35000	35.0	Cluster_03168
692.60000	35.0	Cluster_03182
692.84000	35.0	Cluster_34402
693.56000	35.0	Cluster_03192
693.60000	35.0	Cluster_34413
693.88000	35.0	Cluster_34418
694.36000	35.0	Cluster_34424
694.84000	35.0	Cluster_34436
694.87000	35.0	Cluster_34443
695.01000	35.0	Cluster_12183
695.36000	35.0	Cluster_34458
695.70000	35.0	Cluster_12190
695.87000	35.0	Cluster_34468
696.32000	35.0	Cluster_34489
696.34000	35.0	Cluster_03210
696.39000	35.0	Cluster_34483
696.58000	35.0	Cluster_03211
697.31000	35.0	Cluster_03215
697.37000	35.0	Cluster_34515
697.85000	35.0	Cluster_34520
697.87000	35.0	Cluster_34523
698.82000	35.0	Cluster_34541
698.83000	35.0	Cluster_34546
699.70000	35.0	Cluster_12244
699.85000	35.0	Cluster_03235
700.26000	35.0	Cluster_34580
700.36000	35.0	Cluster_34589
700.65000	35.0	Cluster_34590
700.85000	35.0	Cluster_34594
701.36000	35.0	Cluster_34614
702.34000	35.0	Cluster_34653
702.62000	35.0	Cluster_12286
703.04000	35.0	Cluster_12291
703.05000	35.0	Cluster_03266
703.33000	35.0	Cluster_34677
703.69000	35.0	Cluster_12310
703.91000	35.0	Cluster_03274
704.17000	35.0	Cluster_34698
704.21000	35.0	Cluster_34700
704.31000	35.0	Cluster_34706
704.81000	35.0	Cluster_34728
704.85000	35.0	Cluster_34734
705.04000	35.0	Cluster_12319
705.37000	35.0	Cluster_34742

705.83000	35.0	Cluster_34756
705.88000	35.0	Cluster_34757
706.86000	35.0	Cluster_03301
707.02000	35.0	Cluster_12346
707.80000	35.0	Cluster_34791
708.51000	35.0	Cluster_00309
709.28000	35.0	Cluster_34840
709.39000	35.0	Cluster_34846
710.88000	35.0	Cluster_03335
711.06000	35.0	Cluster_12397
711.85000	35.0	Cluster_34925
712.39000	35.0	Cluster_34948
712.84000	35.0	Cluster_34967
713.08000	35.0	Cluster_03351
713.29000	35.0	Cluster_03356
713.38000	35.0	Cluster_12431
713.72000	35.0	Cluster_12437
713.77000	35.0	Cluster_34987
713.92000	35.0	Cluster_34998
714.32000	35.0	Cluster_35004
714.36000	35.0	Cluster_35010
715.38000	35.0	Cluster_35041
715.54000	35.0	Cluster_00320
715.71000	35.0	Cluster_12456
715.85000	35.0	Cluster_35046
715.99000	35.0	Cluster_12465
716.39000	35.0	Cluster_35061
717.36000	35.0	Cluster_12478
717.90000	35.0	Cluster_35102
718.36000	35.0	Cluster_35122
718.85000	35.0	Cluster_35127
718.89000	35.0	Cluster_35137
718.91000	35.0	Cluster_35135
718.96000	35.0	Cluster_12511
719.39000	35.0	Cluster_35145
719.83000	35.0	Cluster_35161
719.85000	35.0	Cluster_03411
719.98000	35.0	Cluster_12519
720.12000	35.0	Cluster_35168
720.13000	35.0	Cluster_35170
720.35000	35.0	Cluster_03417
720.58000	35.0	Cluster_03421
720.83000	35.0	Cluster_35178
721.32000	35.0	Cluster_35195
721.41000	35.0	Cluster_35191
721.72000	35.0	Cluster_12548
721.88000	35.0	Cluster_35209
721.90000	35.0	Cluster_35213
722.02000	35.0	Cluster_12553
722.09000	35.0	Cluster_03436
722.34000	35.0	Cluster_03433

722.68000	35.0	Cluster_12565
723.40000	35.0	Cluster_12568
723.42000	35.0	Cluster_12570
723.95000	35.0	Cluster_00326
724.04000	35.0	Cluster_12575
724.90000	35.0	Cluster_03465
725.06000	35.0	Cluster_12593
725.32000	35.0	Cluster_03469
725.41000	35.0	Cluster_35286
725.42000	35.0	Cluster_35293
727.38000	35.0	Cluster_35317
728.10000	35.0	Cluster_03487
728.13000	35.0	Cluster_03484
728.42000	35.0	Cluster_35338
728.98000	35.0	Cluster_12653
729.15000	35.0	Cluster_03497
729.84000	35.0	Cluster_35373
729.89000	35.0	Cluster_03507
730.91000	35.0	Cluster_35408
731.10000	35.0	Cluster_35410
731.83000	35.0	Cluster_03518
732.11000	35.0	Cluster_03522
732.64000	35.0	Cluster_03534
733.03000	35.0	Cluster_12708
733.13000	35.0	Cluster_03548
733.83000	35.0	Cluster_35471
733.85000	35.0	Cluster_35474
734.58000	35.0	Cluster_35493
735.04000	35.0	Cluster_12729
735.74000	35.0	Cluster_12741
736.37000	35.0	Cluster_35541
737.04000	35.0	Cluster_12754
737.16000	35.0	Cluster_35560
737.82000	35.0	Cluster_35593
738.39000	35.0	Cluster_35611
738.63000	35.0	Cluster_03590
739.38000	35.0	Cluster_12791
739.39000	35.0	Cluster_12789
739.84000	35.0	Cluster_35645
740.37000	35.0	Cluster_35657
740.38000	35.0	Cluster_35661
740.93000	35.0	Cluster_35676
741.85000	35.0	Cluster_35683
742.37000	35.0	Cluster_35697
742.67000	35.0	Cluster_12831
742.84000	35.0	Cluster_35707
744.37000	35.0	Cluster_35750
745.38000	35.0	Cluster_35777
745.68000	35.0	Cluster_12857
746.06000	35.0	Cluster_12863
746.15000	35.0	Cluster_35792

746.30000	35.0	Cluster_35797
747.12000	35.0	Cluster_35817
747.37000	35.0	Cluster_35818
748.00000	35.0	Cluster_12903
748.86000	35.0	Cluster_03654
748.88000	35.0	Cluster_35857
749.07000	35.0	Cluster_12920
749.69000	35.0	Cluster_12929
749.98000	35.0	Cluster_12936
750.01000	35.0	Cluster_12941
750.38000	35.0	Cluster_35883
750.44000	35.0	Cluster_35886
750.94000	35.0	Cluster_35903
751.06000	35.0	Cluster_12961
751.15000	35.0	Cluster_03662
751.36000	35.0	Cluster_35908
752.37000	35.0	Cluster_35932
753.05000	35.0	Cluster_12988
753.06000	35.0	Cluster_12984
753.39000	35.0	Cluster_35964
753.91000	35.0	Cluster_35981
754.06000	35.0	Cluster_13009
754.90000	35.0	Cluster_36004
755.06000	35.0	Cluster_13021
755.30000	35.0	Cluster_13019
755.33000	35.0	Cluster_13020
755.42000	35.0	Cluster_36013
755.75000	35.0	Cluster_13034
755.92000	35.0	Cluster_36043
756.13000	35.0	Cluster_03688
757.31000	35.0	Cluster_36071
757.73000	35.0	Cluster_13076
758.41000	35.0	Cluster_36102
758.70000	35.0	Cluster_13088
759.63000	35.0	Cluster_03704
759.90000	35.0	Cluster_36129
759.91000	35.0	Cluster_36132
760.35000	35.0	Cluster_36146
761.05000	35.0	Cluster_00024
761.41000	35.0	Cluster_36182
762.38000	35.0	Cluster_13130
763.03000	35.0	Cluster_13141
763.08000	35.0	Cluster_13137
763.35000	35.0	Cluster_03726
763.71000	35.0	Cluster_13147
764.09000	35.0	Cluster_13148
764.36000	35.0	Cluster_36253
765.15000	35.0	Cluster_03741
765.79000	35.0	Cluster_36274
765.80000	35.0	Cluster_36275
766.37000	35.0	Cluster_36292

766.55000	35.0	Cluster_00339
767.70000	35.0	Cluster_13203
767.85000	35.0	Cluster_36325
768.07000	35.0	Cluster_13209
768.08000	35.0	Cluster_13205
768.38000	35.0	Cluster_36351
769.33000	35.0	Cluster_36370
769.59000	35.0	Cluster_36368
769.82000	35.0	Cluster_36385
772.41000	35.0	Cluster_36441
772.74000	35.0	Cluster_13265
772.80000	35.0	Cluster_13273
772.88000	35.0	Cluster_36459
772.92000	35.0	Cluster_36460
773.08000	35.0	Cluster_03785
773.09000	35.0	Cluster_03786
773.13000	35.0	Cluster_13272
773.35000	35.0	Cluster_13283
773.41000	35.0	Cluster_13286
774.12000	35.0	Cluster_36481
774.73000	35.0	Cluster_13306
775.06000	35.0	Cluster_13312
775.07000	35.0	Cluster_13311
775.42000	35.0	Cluster_36510
776.37000	35.0	Cluster_03803
777.15000	35.0	Cluster_03806
777.35000	35.0	Cluster_36561
777.38000	35.0	Cluster_36567
777.39000	35.0	Cluster_36564
778.37000	35.0	Cluster_36591
778.77000	35.0	Cluster_13350
778.87000	35.0	Cluster_36605
780.73000	35.0	Cluster_13379
780.81000	35.0	Cluster_36636
780.88000	35.0	Cluster_03821
781.40000	35.0	Cluster_36661
782.03000	35.0	Cluster_13403
783.13000	35.0	Cluster_03834
783.46000	35.0	Cluster_36708
783.72000	35.0	Cluster_13422
784.06000	35.0	Cluster_13433
784.89000	35.0	Cluster_36733
784.93000	35.0	Cluster_36732
785.45000	35.0	Cluster_36744
785.83000	35.0	Cluster_36763
787.06000	35.0	Cluster_13459
787.12000	35.0	Cluster_03868
787.35000	35.0	Cluster_03870
788.87000	35.0	Cluster_36840
789.06000	35.0	Cluster_13483
790.87000	35.0	Cluster_36906

791.12000	35.0	Cluster_36909
791.38000	35.0	Cluster_36914
791.90000	35.0	Cluster_36919
792.38000	35.0	Cluster_36933
792.64000	35.0	Cluster_36941
792.86000	35.0	Cluster_03914
792.87000	35.0	Cluster_36948
792.97000	35.0	Cluster_13541
793.78000	35.0	Cluster_13547
794.36000	35.0	Cluster_36984
794.37000	35.0	Cluster_36982
794.45000	35.0	Cluster_36985
794.65000	35.0	Cluster_36986
795.07000	35.0	Cluster_13571
795.42000	35.0	Cluster_37004
795.44000	35.0	Cluster_37000
795.68000	35.0	Cluster_13585
795.87000	35.0	Cluster_03947
795.90000	35.0	Cluster_37017
795.96000	35.0	Cluster_37020
796.41000	35.0	Cluster_13605
796.72000	35.0	Cluster_13597
796.91000	35.0	Cluster_37044
797.86000	35.0	Cluster_37077
797.87000	35.0	Cluster_37073
797.88000	35.0	Cluster_37072
797.89000	35.0	Cluster_37068
799.38000	35.0	Cluster_37115
799.40000	35.0	Cluster_37112
799.89000	35.0	Cluster_03975
800.35000	35.0	Cluster_37146
800.41000	35.0	Cluster_13643
800.84000	35.0	Cluster_37149
800.86000	35.0	Cluster_03991
801.06000	35.0	Cluster_00029
801.37000	35.0	Cluster_03995
801.42000	35.0	Cluster_37163
801.44000	35.0	Cluster_13660
802.43000	35.0	Cluster_37188
802.74000	35.0	Cluster_13681
803.41000	35.0	Cluster_13684
803.88000	35.0	Cluster_37226
804.43000	35.0	Cluster_37243
804.91000	35.0	Cluster_04015
805.16000	35.0	Cluster_37269
805.39000	35.0	Cluster_37283
805.94000	35.0	Cluster_37284
806.39000	35.0	Cluster_37304
807.01000	35.0	Cluster_13723
807.46000	35.0	Cluster_37330
807.92000	35.0	Cluster_37352

808.85000	35.0	Cluster_37370
808.91000	35.0	Cluster_37377
809.40000	35.0	Cluster_04037
809.91000	35.0	Cluster_37401
810.39000	35.0	Cluster_37407
810.44000	35.0	Cluster_13774
811.74000	35.0	Cluster_00036
811.86000	35.0	Cluster_37446
811.91000	35.0	Cluster_37441
812.39000	35.0	Cluster_37458
812.41000	35.0	Cluster_13796
813.82000	35.0	Cluster_00352
813.90000	35.0	Cluster_37483
814.10000	35.0	Cluster_13827
814.16000	35.0	Cluster_37492
814.17000	35.0	Cluster_04067
814.90000	35.0	Cluster_04074
815.06000	35.0	Cluster_13832
815.79000	35.0	Cluster_13844
815.92000	35.0	Cluster_37530
816.38000	35.0	Cluster_37540
816.43000	35.0	Cluster_04088
816.72000	35.0	Cluster_13857
817.34000	35.0	Cluster_37561
817.88000	35.0	Cluster_37570
818.42000	35.0	Cluster_13883
819.05000	35.0	Cluster_13897
819.42000	35.0	Cluster_13906
820.17000	35.0	Cluster_04104
821.23000	35.0	Cluster_04111
821.38000	35.0	Cluster_37637
822.06000	35.0	Cluster_13934
823.42000	35.0	Cluster_37669
823.91000	35.0	Cluster_37674
823.94000	35.0	Cluster_37688
825.32000	35.0	Cluster_37717
825.39000	35.0	Cluster_04137
826.38000	35.0	Cluster_13981
826.86000	35.0	Cluster_37765
827.04000	35.0	Cluster_13986
827.06000	35.0	Cluster_13988
827.09000	35.0	Cluster_13989
827.88000	35.0	Cluster_37794
828.80000	35.0	Cluster_14000
828.98000	35.0	Cluster_14004
829.16000	35.0	Cluster_04165
829.36000	35.0	Cluster_37821
829.43000	35.0	Cluster_37825
829.91000	35.0	Cluster_04169
830.74000	35.0	Cluster_14020
830.89000	35.0	Cluster_00042

831.45000	35.0	Cluster_37853
832.41000	35.0	Cluster_37873
832.68000	35.0	Cluster_37879
833.49000	35.0	Cluster_37894
833.99000	35.0	Cluster_37902
834.10000	35.0	Cluster_14054
834.94000	35.0	Cluster_37918
835.05000	35.0	Cluster_14062
836.39000	35.0	Cluster_14076
836.42000	35.0	Cluster_14077
836.76000	35.0	Cluster_14083
836.86000	35.0	Cluster_37959
836.94000	35.0	Cluster_04234
837.41000	35.0	Cluster_37970
837.95000	35.0	Cluster_37976
839.10000	35.0	Cluster_14102
840.66000	35.0	Cluster_04260
840.78000	35.0	Cluster_14117
841.08000	35.0	Cluster_14128
841.10000	35.0	Cluster_14126
841.67000	35.0	Cluster_00364
841.76000	35.0	Cluster_38066
842.73000	35.0	Cluster_14140
843.87000	35.0	Cluster_38114
844.18000	35.0	Cluster_04298
844.44000	35.0	Cluster_14157
846.98000	35.0	Cluster_38180
847.48000	35.0	Cluster_38200
847.75000	35.0	Cluster_14185
847.88000	35.0	Cluster_38203
848.40000	35.0	Cluster_04350
848.91000	35.0	Cluster_04352
849.46000	35.0	Cluster_38235
849.64000	35.0	Cluster_04364
849.97000	35.0	Cluster_38245
849.99000	35.0	Cluster_38248
850.33000	35.0	Cluster_38250
850.40000	35.0	Cluster_04376
850.76000	35.0	Cluster_14213
851.36000	35.0	Cluster_14215
851.77000	35.0	Cluster_38298
852.03000	35.0	Cluster_38307
852.91000	35.0	Cluster_04400
852.93000	35.0	Cluster_04401
853.45000	35.0	Cluster_38334
854.44000	35.0	Cluster_38377
854.47000	35.0	Cluster_38375
854.88000	35.0	Cluster_38385
855.41000	35.0	Cluster_38392
855.73000	35.0	Cluster_14240
856.43000	35.0	Cluster_14249

856.91000	35.0	Cluster_04444
857.06000	35.0	Cluster_14257
857.89000	35.0	Cluster_38459
857.91000	35.0	Cluster_04449
858.07000	35.0	Cluster_14269
858.77000	35.0	Cluster_14275
859.45000	35.0	Cluster_38500
859.47000	35.0	Cluster_38497
860.47000	35.0	Cluster_14284
861.39000	35.0	Cluster_04493
862.39000	35.0	Cluster_04505
862.49000	35.0	Cluster_38591
863.05000	35.0	Cluster_14302
863.76000	35.0	Cluster_14307
863.94000	35.0	Cluster_38615
864.07000	35.0	Cluster_14314
865.03000	35.0	Cluster_38646
865.75000	35.0	Cluster_14326
865.92000	35.0	Cluster_38659
865.93000	35.0	Cluster_04555
866.41000	35.0	Cluster_14334
869.07000	35.0	Cluster_14353
869.28000	35.0	Cluster_38719
869.41000	35.0	Cluster_04599
869.78000	35.0	Cluster_14363
871.17000	35.0	Cluster_38758
871.27000	35.0	Cluster_38759
871.44000	35.0	Cluster_38769
871.47000	35.0	Cluster_14388
872.27000	35.0	Cluster_38802
874.09000	35.0	Cluster_14414
874.26000	35.0	Cluster_38844
874.78000	35.0	Cluster_14424
875.28000	35.0	Cluster_38860
875.66000	35.0	Cluster_04698
876.08000	35.0	Cluster_14437
876.53000	35.0	Cluster_38895
877.38000	35.0	Cluster_14453
877.44000	35.0	Cluster_38909
878.48000	35.0	Cluster_38928
880.46000	35.0	Cluster_14483
881.96000	35.0	Cluster_38993
882.44000	35.0	Cluster_39008
882.94000	35.0	Cluster_39018
884.40000	35.0	Cluster_14528
884.66000	35.0	Cluster_39047
885.12000	35.0	Cluster_14544
885.68000	35.0	Cluster_04765
885.95000	35.0	Cluster_39078
886.43000	35.0	Cluster_39095
886.96000	35.0	Cluster_39099

887.52000	35.0	Cluster_39107
887.68000	35.0	Cluster_04781
887.74000	35.0	Cluster_14571
888.13000	35.0	Cluster_14577
888.40000	35.0	Cluster_14578
888.91000	35.0	Cluster_39135
888.92000	35.0	Cluster_04786
889.11000	35.0	Cluster_14581
889.44000	35.0	Cluster_39147
890.17000	35.0	Cluster_04796
890.38000	35.0	Cluster_39161
890.39000	35.0	Cluster_39160
890.46000	35.0	Cluster_39173
890.99000	35.0	Cluster_39180
891.46000	35.0	Cluster_39186
891.81000	35.0	Cluster_14598
892.21000	35.0	Cluster_04824
893.43000	35.0	Cluster_39231
893.78000	35.0	Cluster_14615
894.12000	35.0	Cluster_14617
894.71000	35.0	Cluster_04845
895.21000	35.0	Cluster_04851
895.45000	35.0	Cluster_14622
895.81000	35.0	Cluster_14625
896.15000	35.0	Cluster_14634
897.04000	35.0	Cluster_39292
898.77000	35.0	Cluster_14664
899.43000	35.0	Cluster_39337
899.51000	35.0	Cluster_39345
899.96000	35.0	Cluster_04891
900.94000	35.0	Cluster_39368
900.97000	35.0	Cluster_39380
900.99000	35.0	Cluster_39377
902.46000	35.0	Cluster_39412
904.46000	35.0	Cluster_39443
905.43000	35.0	Cluster_39461
906.77000	35.0	Cluster_14739
907.80000	35.0	Cluster_14749
908.46000	35.0	Cluster_39500
908.48000	35.0	Cluster_14756
908.94000	35.0	Cluster_04958
908.95000	35.0	Cluster_39504
909.44000	35.0	Cluster_14760
909.50000	35.0	Cluster_14765
909.89000	35.0	Cluster_39520
910.11000	35.0	Cluster_14769
910.19000	35.0	Cluster_04966
911.96000	35.0	Cluster_39564
914.56000	35.0	Cluster_39615
915.13000	35.0	Cluster_14823
915.93000	35.0	Cluster_39626

916.44000	35.0	Cluster_39640
916.49000	35.0	Cluster_39633
917.45000	35.0	Cluster_14854
917.97000	35.0	Cluster_39674
918.66000	35.0	Cluster_39698
920.45000	35.0	Cluster_39721
920.76000	35.0	Cluster_14891
920.79000	35.0	Cluster_39725
920.88000	35.0	Cluster_39726
920.95000	35.0	Cluster_39731
922.42000	35.0	Cluster_14907
922.98000	35.0	Cluster_39772
923.30000	35.0	Cluster_14924
923.45000	35.0	Cluster_14932
923.92000	35.0	Cluster_39782
924.49000	35.0	Cluster_39788
925.11000	35.0	Cluster_14961
926.42000	35.0	Cluster_14975
927.48000	35.0	Cluster_39834
928.47000	35.0	Cluster_39858
928.96000	35.0	Cluster_39861
929.12000	35.0	Cluster_15009
929.43000	35.0	Cluster_39880
929.90000	35.0	Cluster_39897
930.47000	35.0	Cluster_15020
931.73000	35.0	Cluster_15043
931.90000	35.0	Cluster_39924
932.64000	35.0	Cluster_15062
932.95000	35.0	Cluster_39941
933.14000	35.0	Cluster_15072
933.33000	35.0	Cluster_05038
934.00000	35.0	Cluster_39963
934.13000	35.0	Cluster_15084
934.43000	35.0	Cluster_15083
934.78000	35.0	Cluster_15089
935.78000	35.0	Cluster_15100
936.45000	35.0	Cluster_15116
936.80000	35.0	Cluster_15129
937.00000	35.0	Cluster_39997
937.44000	35.0	Cluster_15144
937.49000	35.0	Cluster_05051
938.07000	35.0	Cluster_15154
938.13000	35.0	Cluster_15156
938.21000	35.0	Cluster_15160
939.69000	35.0	Cluster_05058
940.46000	35.0	Cluster_15180
941.72000	35.0	Cluster_40098
942.00000	35.0	Cluster_40094
943.45000	35.0	Cluster_15229
945.13000	35.0	Cluster_15244
945.39000	35.0	Cluster_15242

945.72000	35.0	Cluster_05075
945.73000	35.0	Cluster_05078
945.78000	35.0	Cluster_15250
945.98000	35.0	Cluster_40152
947.16000	35.0	Cluster_15264
947.22000	35.0	Cluster_05084
947.79000	35.0	Cluster_15273
948.19000	35.0	Cluster_15277
948.52000	35.0	Cluster_40180
949.14000	35.0	Cluster_15301
949.44000	35.0	Cluster_40190
949.46000	35.0	Cluster_15302
950.44000	35.0	Cluster_15314
951.17000	35.0	Cluster_15324
951.96000	35.0	Cluster_40227
952.14000	35.0	Cluster_15337
952.15000	35.0	Cluster_15339
952.44000	35.0	Cluster_15342
952.53000	35.0	Cluster_40233
953.98000	35.0	Cluster_40255
954.82000	35.0	Cluster_15379
954.99000	35.0	Cluster_40262
955.13000	35.0	Cluster_15394
955.47000	35.0	Cluster_15404
956.48000	35.0	Cluster_15426
957.07000	35.0	Cluster_40290
957.48000	35.0	Cluster_40291
960.15000	35.0	Cluster_15462
960.98000	35.0	Cluster_40317
961.48000	35.0	Cluster_15478
962.14000	35.0	Cluster_15497
962.38000	35.0	Cluster_40344
962.79000	35.0	Cluster_15505
964.13000	35.0	Cluster_15523
964.43000	35.0	Cluster_40365
965.29000	35.0	Cluster_40377
966.15000	35.0	Cluster_15549
966.49000	35.0	Cluster_40387
966.50000	35.0	Cluster_05172
966.85000	35.0	Cluster_15552
967.00000	35.0	Cluster_40392
967.56000	35.0	Cluster_15565
968.03000	35.0	Cluster_40402
968.25000	35.0	Cluster_05187
971.97000	35.0	Cluster_40451
972.01000	35.0	Cluster_40467
972.46000	35.0	Cluster_15627
972.49000	35.0	Cluster_15626
973.51000	35.0	Cluster_40476
974.47000	35.0	Cluster_15651
974.98000	35.0	Cluster_40495

975.82000	35.0	Cluster_15672
975.98000	35.0	Cluster_05210
976.00000	35.0	Cluster_05212
976.03000	35.0	Cluster_40504
977.14000	35.0	Cluster_15696
977.62000	35.0	Cluster_40522
979.45000	35.0	Cluster_40546
979.80000	35.0	Cluster_15720
979.83000	35.0	Cluster_15721
979.98000	35.0	Cluster_40568
980.00000	35.0	Cluster_40562
980.50000	35.0	Cluster_40565
980.70000	35.0	Cluster_40578
981.45000	35.0	Cluster_15750
983.25000	35.0	Cluster_05237
983.95000	35.0	Cluster_40617
985.14000	35.0	Cluster_40628
986.02000	35.0	Cluster_40630
986.52000	35.0	Cluster_40639
986.83000	35.0	Cluster_15810
987.24000	35.0	Cluster_40654
987.80000	35.0	Cluster_15825
988.44000	35.0	Cluster_15835
991.93000	35.0	Cluster_40717
993.50000	35.0	Cluster_15895
997.19000	35.0	Cluster_15954
1000.06000	35.0	Cluster_40851
1001.03000	35.0	Cluster_40878
1001.16000	35.0	Cluster_15996
1001.49000	35.0	Cluster_40884
1003.52000	35.0	Cluster_05294
1004.00000	35.0	Cluster_40937
1004.31000	35.0	Cluster_05296
1004.57000	35.0	Cluster_40940
1007.81000	35.0	Cluster_16072
1008.78000	35.0	Cluster_16080
1012.26000	35.0	Cluster_05329
1012.27000	35.0	Cluster_41044
1012.93000	35.0	Cluster_41054
1013.01000	35.0	Cluster_05341
1013.47000	35.0	Cluster_16138
1013.49000	35.0	Cluster_16137
1013.76000	35.0	Cluster_05344
1013.90000	35.0	Cluster_41056
1015.92000	35.0	Cluster_41088
1016.78000	35.0	Cluster_05351
1017.27000	35.0	Cluster_41099
1019.25000	35.0	Cluster_05360
1020.15000	35.0	Cluster_16210
1022.50000	35.0	Cluster_16231
1023.82000	35.0	Cluster_41185

1024.48000	35.0	Cluster_16254
1024.50000	35.0	Cluster_16256
1024.93000	35.0	Cluster_41195
1025.46000	35.0	Cluster_16264
1025.81000	35.0	Cluster_41211
1026.83000	35.0	Cluster_16274
1027.13000	35.0	Cluster_16285
1027.14000	35.0	Cluster_16284
1027.88000	35.0	Cluster_16291
1028.18000	35.0	Cluster_16295
1028.84000	35.0	Cluster_16300
1029.99000	35.0	Cluster_41262
1031.20000	35.0	Cluster_16323
1032.82000	35.0	Cluster_16335
1033.20000	35.0	Cluster_16342
1036.52000	35.0	Cluster_16382
1037.50000	35.0	Cluster_16400
1039.05000	35.0	Cluster_41371
1039.52000	35.0	Cluster_41374
1039.81000	35.0	Cluster_16429
1040.83000	35.0	Cluster_05437
1041.01000	35.0	Cluster_41401
1042.17000	35.0	Cluster_16457
1044.49000	35.0	Cluster_41432
1046.13000	35.0	Cluster_16488
1046.22000	35.0	Cluster_16495
1047.16000	35.0	Cluster_16507
1047.83000	35.0	Cluster_41470
1053.50000	35.0	Cluster_16581
1053.98000	35.0	Cluster_41534
1054.99000	35.0	Cluster_05476
1055.22000	35.0	Cluster_16595
1055.50000	35.0	Cluster_16606
1056.52000	35.0	Cluster_16616
1057.06000	35.0	Cluster_41567
1057.33000	35.0	Cluster_05486
1057.34000	35.0	Cluster_05487
1057.58000	35.0	Cluster_41574
1057.99000	35.0	Cluster_41578
1058.02000	35.0	Cluster_41582
1058.81000	35.0	Cluster_16658
1058.85000	35.0	Cluster_16649
1058.96000	35.0	Cluster_41595
1058.97000	35.0	Cluster_41597
1059.01000	35.0	Cluster_41596
1060.32000	35.0	Cluster_41612
1060.75000	35.0	Cluster_05502
1060.82000	35.0	Cluster_16679
1061.26000	35.0	Cluster_05510
1061.33000	35.0	Cluster_05506
1061.82000	35.0	Cluster_41625

1062.83000	35.0	Cluster_16700
1063.27000	35.0	Cluster_05516
1063.82000	35.0	Cluster_41655
1063.99000	35.0	Cluster_41663
1064.72000	35.0	Cluster_05521
1064.73000	35.0	Cluster_41675
1064.99000	35.0	Cluster_41673
1066.26000	35.0	Cluster_41702
1067.52000	35.0	Cluster_41715
1068.05000	35.0	Cluster_41719
1068.75000	35.0	Cluster_41734
1069.51000	35.0	Cluster_41748
1070.16000	35.0	Cluster_16781
1070.51000	35.0	Cluster_05546
1072.55000	35.0	Cluster_16804
1072.76000	35.0	Cluster_05555
1074.19000	35.0	Cluster_16831
1074.51000	35.0	Cluster_41810
1075.53000	35.0	Cluster_41816
1076.75000	35.0	Cluster_05574
1078.01000	35.0	Cluster_05578
1079.49000	35.0	Cluster_05582
1080.75000	35.0	Cluster_00593
1082.00000	35.0	Cluster_05588
1083.50000	35.0	Cluster_05592
1086.59000	35.0	Cluster_41940
1091.58000	35.0	Cluster_41998
1092.90000	35.0	Cluster_17025
1093.21000	35.0	Cluster_17031
1093.55000	35.0	Cluster_17035
1093.58000	35.0	Cluster_42024
1093.99000	35.0	Cluster_42029
1094.92000	35.0	Cluster_00625
1096.55000	35.0	Cluster_42051
1100.23000	35.0	Cluster_17091
1100.56000	35.0	Cluster_17097
1101.96000	35.0	Cluster_17105
1102.54000	35.0	Cluster_17111
1103.12000	35.0	Cluster_42104
1108.23000	35.0	Cluster_17176
1108.37000	35.0	Cluster_42148
1108.88000	35.0	Cluster_17175
1109.86000	35.0	Cluster_17199
1113.50000	35.0	Cluster_42184
1113.53000	35.0	Cluster_17241
1115.18000	35.0	Cluster_17258
1115.77000	35.0	Cluster_00660
1117.22000	35.0	Cluster_17276
1118.21000	35.0	Cluster_17277
1118.22000	35.0	Cluster_17280
1119.87000	35.0	Cluster_42234

1121.54000	35.0	Cluster_17322
1123.04000	35.0	Cluster_42270
1123.97000	35.0	Cluster_42271
1124.25000	35.0	Cluster_17359
1125.57000	35.0	Cluster_42283
1126.07000	35.0	Cluster_42291
1127.88000	35.0	Cluster_17398
1128.57000	35.0	Cluster_17409
1129.21000	35.0	Cluster_17418
1129.55000	35.0	Cluster_17428
1129.90000	35.0	Cluster_17433
1130.23000	35.0	Cluster_17434
1131.58000	35.0	Cluster_17448
1133.13000	35.0	Cluster_42337
1133.20000	35.0	Cluster_17479
1133.22000	35.0	Cluster_17484
1134.90000	35.0	Cluster_17503
1135.23000	35.0	Cluster_17509
1135.51000	35.0	Cluster_17513
1135.54000	35.0	Cluster_17519
1135.55000	35.0	Cluster_17517
1136.11000	35.0	Cluster_42363
1136.88000	35.0	Cluster_17525
1136.89000	35.0	Cluster_17538
1138.13000	35.0	Cluster_42377
1140.22000	35.0	Cluster_17567
1143.18000	35.0	Cluster_00674
1143.56000	35.0	Cluster_17607
1143.60000	35.0	Cluster_42409
1144.20000	35.0	Cluster_17626
1144.24000	35.0	Cluster_17620
1145.21000	35.0	Cluster_17645
1146.57000	35.0	Cluster_00680
1149.29000	35.0	Cluster_42461
1151.05000	35.0	Cluster_42474
1152.54000	35.0	Cluster_17735
1152.88000	35.0	Cluster_17750
1153.03000	35.0	Cluster_05790
1153.76000	35.0	Cluster_00695
1154.35000	35.0	Cluster_42485
1155.34000	35.0	Cluster_42501
1156.84000	35.0	Cluster_42511
1158.84000	35.0	Cluster_42519
1159.61000	35.0	Cluster_42529
1160.34000	35.0	Cluster_42534
1162.11000	35.0	Cluster_42551
1162.64000	35.0	Cluster_42555
1163.09000	35.0	Cluster_05817
1166.08000	35.0	Cluster_05827
1166.14000	35.0	Cluster_42592
1166.21000	35.0	Cluster_17894

1167.19000	35.0	Cluster_17908
1167.57000	35.0	Cluster_17921
1168.21000	35.0	Cluster_17931
1168.33000	35.0	Cluster_42608
1168.58000	35.0	Cluster_05832
1173.07000	35.0	Cluster_42645
1180.80000	35.0	Cluster_05888
1183.23000	35.0	Cluster_18040
1187.59000	35.0	Cluster_18075
1190.56000	35.0	Cluster_18091
1192.81000	35.0	Cluster_42765
1192.92000	35.0	Cluster_18117
1195.88000	35.0	Cluster_18133
1196.63000	35.0	Cluster_42787
1197.59000	35.0	Cluster_18145
1197.90000	35.0	Cluster_18140
1198.64000	35.0	Cluster_42803
1199.32000	35.0	Cluster_05982
1199.57000	35.0	Cluster_18161
1200.26000	35.0	Cluster_18168
1200.56000	35.0	Cluster_18177
1201.89000	35.0	Cluster_18192
1203.44000	35.0	Cluster_42830
1206.62000	35.0	Cluster_42853
1208.56000	35.0	Cluster_18237
1209.51000	35.0	Cluster_42887
1213.12000	35.0	Cluster_42910
1215.15000	35.0	Cluster_42924
1218.11000	35.0	Cluster_42950
1219.09000	35.0	Cluster_42956
1221.25000	35.0	Cluster_18299
1222.10000	35.0	Cluster_42968
1224.08000	35.0	Cluster_42977
1224.60000	35.0	Cluster_42983
1225.59000	35.0	Cluster_42994
1228.07000	35.0	Cluster_43008
1228.11000	35.0	Cluster_43007
1230.63000	35.0	Cluster_43026
1232.57000	35.0	Cluster_43045
1234.58000	35.0	Cluster_43065
1238.57000	35.0	Cluster_43079
1245.28000	35.0	Cluster_18390
1249.16000	35.0	Cluster_43135
1251.87000	35.0	Cluster_43149
1253.87000	35.0	Cluster_43162
1255.86000	35.0	Cluster_43176
1257.08000	35.0	Cluster_06282
1264.27000	35.0	Cluster_18463
1267.20000	35.0	Cluster_43224
1267.68000	35.0	Cluster_43228
1274.29000	35.0	Cluster_18502

1284.15000	35.0	Cluster_43287
1300.69000	35.0	Cluster_43366
1300.70000	35.0	Cluster_43360
1300.73000	35.0	Cluster_43361
1300.89000	35.0	Cluster_43362
1301.68000	35.0	Cluster_43365
1320.21000	35.0	Cluster_43449
1329.73000	35.0	Cluster_43484
1330.45000	35.0	Cluster_06499
1331.69000	35.0	Cluster_43492
1334.68000	35.0	Cluster_43505
1349.89000	35.0	Cluster_43562
1353.71000	35.0	Cluster_43578
1354.21000	35.0	Cluster_43579
1354.66000	35.0	Cluster_06571
1361.03000	35.0	Cluster_18681
1367.16000	35.0	Cluster_43645
1372.75000	35.0	Cluster_43667
1376.18000	35.0	Cluster_43680
1376.34000	35.0	Cluster_18707
1383.67000	35.0	Cluster_18716
1394.65000	35.0	Cluster_43739
1396.44000	35.0	Cluster_18732
1402.12000	35.0	Cluster_18741
1406.24000	35.0	Cluster_43793
1407.03000	35.0	Cluster_18752
1414.35000	35.0	Cluster_18768
1417.37000	35.0	Cluster_18772
1420.01000	35.0	Cluster_18783
1422.96000	35.0	Cluster_43843
1423.47000	35.0	Cluster_43844
1424.69000	35.0	Cluster_18789
1424.79000	35.0	Cluster_43851
1425.27000	35.0	Cluster_43853
1427.03000	35.0	Cluster_18794
1428.82000	35.0	Cluster_43862
1429.02000	35.0	Cluster_18795
1430.01000	35.0	Cluster_18796
1431.68000	35.0	Cluster_18799
1437.03000	35.0	Cluster_18803
1439.35000	35.0	Cluster_18807
1459.27000	35.0	Cluster_43959
1477.30000	35.0	Cluster_44015
1489.22000	35.0	Cluster_44043
1492.94000	35.0	Cluster_44054
1495.14000	35.0	Cluster_44065
1511.80000	35.0	Cluster_44102
1518.72000	35.0	Cluster_44112
1530.33000	35.0	Cluster_44125
1560.76000	35.0	Cluster_44175
1564.32000	35.0	Cluster_44184

1571.76000	35.0	Cluster_44194
1582.85000	35.0	Cluster_44218
1616.85000	35.0	Cluster_44250
1633.97000	35.0	Cluster_44257
1635.40000	35.0	Cluster_44260
1676.82000	35.0	Cluster_44320
1683.84000	35.0	Cluster_44341
1687.92000	35.0	Cluster_44353
1739.94000	35.0	Cluster_44403
1792.99000	35.0	Cluster_44425

Reject Mass List: (none)
Neutral Loss Mass List: (none)
Product Mass List: (none)
Neutral loss in top: 3
Product in top: 3
Most intense if no parent masses found enabled
Add/subtract mass not enabled
FT master scan preview mode enabled
Charge state screening enabled
Charge state dependent ETD time not enabled
Monoisotopic precursor selection enabled
Charge state rejection enabled
Unassigned charge states : rejected
Charge state 1 : rejected
Charge state 2 : not rejected
Charge state 3 : not rejected
Charge states 4+ : not rejected
Chromatography mode is disabled

Global Data Dependent Settings:
Predict ion injection time enabled
Use global parent and reject mass lists not enabled
Exclude parent mass from data dependent selection not enabled
Exclusion mass width by mass
Exclusion mass width low: 0.50000
Exclusion mass width high: 0.50000
Parent mass width by mass
Parent mass width low: 0.50000
Parent mass width high: 0.50000
Reject mass width by mass
Reject mass width low: 0.50000
Reject mass width high: 0.50000
Zoom/UltraZoom scan mass width by mass
Zoom/UltraZoom scan mass width low: 5.00
Zoom/UltraZoom scan mass width high: 5.00
FT SIM scan mass width low: 5.00
FT SIM scan mass width high: 5.00
Neutral Loss candidates processed by decreasing intensity
Neutral Loss mass width by mass
Neutral Loss mass width low: 0.50000

Neutral Loss mass width high: 0.50000
Product candidates processed by decreasing intensity
Product mass width by mass
Product mass width low: 0.50000
Product mass width high: 0.50000
MS mass range: 0.00-1000000.00
MSn mass range by mass
MSn mass range: 0.00-1000000.00
Use m/z values as masses not enabled
Analog UV data dep. not enabled
Dynamic exclusion not enabled
Isotopic data dependence not enabled
Mass Tags data dependence not enabled
Custom Data Dependent Settings:
Not enabled

Pass 9 (wheat-mixed-digests_MS2_TO-USE-inclusion-01_2.raw):

Creator: Orbi_30393
Last modified: 10/19/2021 by Orbi_30393
MS Run Time (min): 43.00
Sequence override of method parameters not enabled.
Divert Valve: not used during run
Contact Closure: not used during run
Syringe Pump: not used during run
MS Detector Settings:
Real-time modifications to method not enabled
Stepped collision energy not enabled
Additional Microscans:

MS2	0	0
MS3	0	0
MS4	0	0
MS5	0	0
MS6	0	0
MS7	0	0
MS8	0	0
MS9	0	0
MS10	0	0

Segment 1 Information
Duration (min): 43.00
Number of Scan Events: 2
Tune Method: Orbitrap-tune-file_2020-03-13_HESI
Scan Event Details:
1: FTMS + p norm res=15000 o(300.0-2000.0)
CV = 0.0V
2: ITMS + c norm Dep MS/MS Most intense ion from (1)
Activation Type: CID
Min. Signal Required: 500.0
Isolation Width: 1.00
Normalized Coll. Energy: 35.0
Default Charge State: 2

Activation Q: 0.250
Activation Time: 10.000
CV = 0.0V

Lock Masses:
Pos List Name: N/A
Source: API Source
Mass List: (none)
Neg List Name: N/A
Source: API Source
Mass List: (none)

Data Dependent Settings:
Use separate polarity settings disabled
Parent Mass List:

MS Mass	MS FAIMS CV	MS Normalized Collision Energy	MS2 Mass	MS2 Normalized Collision Energy	Name
300.13000		35.0			Cluster_06844
300.14000		35.0			Cluster_18928
300.71000		35.0			Cluster_18955
301.50000		35.0			Cluster_06851
301.65000		35.0			Cluster_18984
302.64000		35.0			Cluster_19016
302.65000		35.0			Cluster_19017
303.99000		35.0			Cluster_19040
304.11000		35.0			Cluster_19042
304.14000		35.0			Cluster_19049
304.16000		35.0			Cluster_19050
304.20000		35.0			Cluster_19052
304.49000		35.0			Cluster_06863
304.64000		35.0			Cluster_19062
306.13000		35.0			Cluster_06873
306.67000		35.0			Cluster_19109
307.12000		35.0			Cluster_19119
307.13000		35.0			Cluster_06880
308.65000		35.0			Cluster_19177
308.66000		35.0			Cluster_19179
309.53000		35.0			Cluster_06901
310.58000		35.0			Cluster_19217
310.65000		35.0			Cluster_19222
310.66000		35.0			Cluster_19221
311.17000		35.0			Cluster_19246
311.68000		35.0			Cluster_19257
313.65000		35.0			Cluster_19315
314.82000		35.0			Cluster_06941
315.65000		35.0			Cluster_19384
316.69000		35.0			Cluster_19442
317.06000		35.0			Cluster_19459
317.16000		35.0			Cluster_19466

317.64000	35.0	Cluster_19490
318.64000	35.0	Cluster_19532
318.85000	35.0	Cluster_06973
319.17000	35.0	Cluster_19552
319.67000	35.0	Cluster_19560
319.94000	35.0	Cluster_00850
320.49000	35.0	Cluster_06993
320.80000	35.0	Cluster_06994
321.15000	35.0	Cluster_06995
321.17000	35.0	Cluster_00869
321.52000	35.0	Cluster_19594
322.15000	35.0	Cluster_19619
322.17000	35.0	Cluster_19617
322.50000	35.0	Cluster_06998
323.65000	35.0	Cluster_19672
323.69000	35.0	Cluster_19677
323.83000	35.0	Cluster_19683
324.42000	35.0	Cluster_00878
324.60000	35.0	Cluster_19711
324.65000	35.0	Cluster_19713
324.83000	35.0	Cluster_07017
325.14000	35.0	Cluster_19730
325.82000	35.0	Cluster_07023
325.85000	35.0	Cluster_07028
326.17000	35.0	Cluster_19753
326.40000	35.0	Cluster_00887
326.90000	35.0	Cluster_00888
327.21000	35.0	Cluster_19791
327.96000	35.0	Cluster_07043
328.48000	35.0	Cluster_07051
329.18000	35.0	Cluster_19855
329.82000	35.0	Cluster_07063
329.93000	35.0	Cluster_19873
330.18000	35.0	Cluster_19886
330.90000	35.0	Cluster_19916
331.17000	35.0	Cluster_19926
331.20000	35.0	Cluster_19928
331.21000	35.0	Cluster_19930
331.22000	35.0	Cluster_19925
331.66000	35.0	Cluster_19936
332.14000	35.0	Cluster_19948
333.47000	35.0	Cluster_07081
333.82000	35.0	Cluster_07087
333.87000	35.0	Cluster_07090
335.12000	35.0	Cluster_20034
335.83000	35.0	Cluster_07104
336.19000	35.0	Cluster_07109
336.23000	35.0	Cluster_20093
336.91000	35.0	Cluster_00920
337.19000	35.0	Cluster_20132
337.21000	35.0	Cluster_20126

337.83000	35.0	Cluster_07126
338.11000	35.0	Cluster_20150
338.67000	35.0	Cluster_20172
339.16000	35.0	Cluster_20192
339.47000	35.0	Cluster_07139
339.81000	35.0	Cluster_07150
339.94000	35.0	Cluster_00929
340.18000	35.0	Cluster_20223
340.26000	35.0	Cluster_20229
341.15000	35.0	Cluster_20257
341.16000	35.0	Cluster_20261
342.16000	35.0	Cluster_07167
343.11000	35.0	Cluster_20316
343.18000	35.0	Cluster_20318
343.66000	35.0	Cluster_20344
345.19000	35.0	Cluster_20390
345.67000	35.0	Cluster_20402
346.15000	35.0	Cluster_07199
346.67000	35.0	Cluster_20444
347.20000	35.0	Cluster_20457
347.71000	35.0	Cluster_20471
348.44000	35.0	Cluster_20495
348.87000	35.0	Cluster_07234
348.92000	35.0	Cluster_00975
348.93000	35.0	Cluster_00976
349.20000	35.0	Cluster_20517
349.21000	35.0	Cluster_20520
349.64000	35.0	Cluster_20543
349.73000	35.0	Cluster_20549
350.42000	35.0	Cluster_00982
351.18000	35.0	Cluster_20611
351.19000	35.0	Cluster_20622
352.66000	35.0	Cluster_20669
353.17000	35.0	Cluster_01002
353.21000	35.0	Cluster_20705
353.66000	35.0	Cluster_01003
353.80000	35.0	Cluster_07278
353.87000	35.0	Cluster_20720
353.91000	35.0	Cluster_01004
354.16000	35.0	Cluster_20732
354.20000	35.0	Cluster_20739
354.40000	35.0	Cluster_01009
354.42000	35.0	Cluster_01011
355.14000	35.0	Cluster_20781
355.16000	35.0	Cluster_20782
355.22000	35.0	Cluster_20787
355.36000	35.0	Cluster_20789
355.70000	35.0	Cluster_20796
356.24000	35.0	Cluster_20824
356.55000	35.0	Cluster_07307
358.42000	35.0	Cluster_20933

359.13000	35.0	Cluster_07326
359.17000	35.0	Cluster_00118
359.37000	35.0	Cluster_20960
360.16000	35.0	Cluster_20986
360.42000	35.0	Cluster_01047
360.83000	35.0	Cluster_21008
361.18000	35.0	Cluster_21024
361.42000	35.0	Cluster_01055
361.66000	35.0	Cluster_21038
361.67000	35.0	Cluster_21036
361.69000	35.0	Cluster_21039
361.84000	35.0	Cluster_07347
362.10000	35.0	Cluster_21048
362.42000	35.0	Cluster_01059
362.43000	35.0	Cluster_21075
362.60000	35.0	Cluster_21077
362.65000	35.0	Cluster_21076
363.11000	35.0	Cluster_21089
363.19000	35.0	Cluster_21093
363.20000	35.0	Cluster_21094
363.77000	35.0	Cluster_21121
364.16000	35.0	Cluster_21135
364.50000	35.0	Cluster_21161
364.65000	35.0	Cluster_21162
365.24000	35.0	Cluster_21221
365.79000	35.0	Cluster_07396
365.90000	35.0	Cluster_21241
366.27000	35.0	Cluster_21267
366.74000	35.0	Cluster_21285
367.22000	35.0	Cluster_21306
367.50000	35.0	Cluster_07416
367.53000	35.0	Cluster_21324
368.16000	35.0	Cluster_21368
368.20000	35.0	Cluster_21366
368.47000	35.0	Cluster_07428
369.67000	35.0	Cluster_21437
370.08000	35.0	Cluster_21453
370.50000	35.0	Cluster_07445
370.74000	35.0	Cluster_21476
370.93000	35.0	Cluster_21486
371.18000	35.0	Cluster_07449
371.24000	35.0	Cluster_21501
371.71000	35.0	Cluster_21517
371.89000	35.0	Cluster_21531
372.17000	35.0	Cluster_21537
372.23000	35.0	Cluster_07464
372.67000	35.0	Cluster_21575
372.69000	35.0	Cluster_21572
373.12000	35.0	Cluster_21589
373.14000	35.0	Cluster_21585
373.16000	35.0	Cluster_07470

373.19000	35.0	Cluster_07471
373.22000	35.0	Cluster_21597
373.90000	35.0	Cluster_21628
374.19000	35.0	Cluster_07480
374.40000	35.0	Cluster_21650
374.68000	35.0	Cluster_21656
375.15000	35.0	Cluster_21668
375.89000	35.0	Cluster_07496
377.48000	35.0	Cluster_07512
377.77000	35.0	Cluster_21780
378.17000	35.0	Cluster_01122
378.18000	35.0	Cluster_21788
378.20000	35.0	Cluster_07523
378.67000	35.0	Cluster_01125
379.26000	35.0	Cluster_21841
380.45000	35.0	Cluster_21886
380.68000	35.0	Cluster_21891
381.22000	35.0	Cluster_07550
381.70000	35.0	Cluster_01141
382.18000	35.0	Cluster_21953
382.66000	35.0	Cluster_01144
383.63000	35.0	Cluster_22024
383.68000	35.0	Cluster_22025
384.16000	35.0	Cluster_01146
384.21000	35.0	Cluster_22055
384.22000	35.0	Cluster_22065
385.52000	35.0	Cluster_07597
385.54000	35.0	Cluster_07596
385.86000	35.0	Cluster_22123
386.66000	35.0	Cluster_22155
386.68000	35.0	Cluster_22158
386.90000	35.0	Cluster_22172
387.11000	35.0	Cluster_22177
387.63000	35.0	Cluster_22217
387.93000	35.0	Cluster_22226
388.23000	35.0	Cluster_22233
388.50000	35.0	Cluster_07621
388.64000	35.0	Cluster_22244
388.67000	35.0	Cluster_22245
388.83000	35.0	Cluster_07623
389.25000	35.0	Cluster_22269
389.51000	35.0	Cluster_07627
389.91000	35.0	Cluster_22284
390.71000	35.0	Cluster_22316
390.87000	35.0	Cluster_07651
390.88000	35.0	Cluster_07646
391.50000	35.0	Cluster_07654
391.65000	35.0	Cluster_22334
391.75000	35.0	Cluster_22344
391.82000	35.0	Cluster_07658
392.22000	35.0	Cluster_07665

392.25000	35.0	Cluster_22358
392.73000	35.0	Cluster_22381
392.77000	35.0	Cluster_22377
393.19000	35.0	Cluster_01195
393.53000	35.0	Cluster_07674
393.69000	35.0	Cluster_01197
393.71000	35.0	Cluster_22428
393.98000	35.0	Cluster_22479
394.21000	35.0	Cluster_22490
394.71000	35.0	Cluster_22522
394.72000	35.0	Cluster_22521
395.89000	35.0	Cluster_07703
396.23000	35.0	Cluster_22589
396.30000	35.0	Cluster_22595
397.39000	35.0	Cluster_22628
397.67000	35.0	Cluster_22634
397.72000	35.0	Cluster_22641
397.90000	35.0	Cluster_07736
398.18000	35.0	Cluster_01226
398.44000	35.0	Cluster_01230
400.24000	35.0	Cluster_22747
400.68000	35.0	Cluster_22780
400.90000	35.0	Cluster_07771
401.70000	35.0	Cluster_22833
402.32000	35.0	Cluster_22866
402.48000	35.0	Cluster_22868
402.72000	35.0	Cluster_22878
403.44000	35.0	Cluster_01260
403.54000	35.0	Cluster_07797
404.47000	35.0	Cluster_01272
405.88000	35.0	Cluster_07831
406.22000	35.0	Cluster_23023
406.68000	35.0	Cluster_01285
406.73000	35.0	Cluster_23046
406.95000	35.0	Cluster_01293
407.17000	35.0	Cluster_23059
407.25000	35.0	Cluster_23066
407.67000	35.0	Cluster_01302
407.75000	35.0	Cluster_23083
407.93000	35.0	Cluster_01303
408.22000	35.0	Cluster_23104
408.54000	35.0	Cluster_07863
408.66000	35.0	Cluster_01305
408.91000	35.0	Cluster_07864
408.92000	35.0	Cluster_01307
409.19000	35.0	Cluster_07867
409.25000	35.0	Cluster_23160
409.69000	35.0	Cluster_23170
410.53000	35.0	Cluster_07882
411.72000	35.0	Cluster_23258
412.67000	35.0	Cluster_23299

412.68000	35.0	Cluster_01326
413.23000	35.0	Cluster_23320
413.87000	35.0	Cluster_07913
413.92000	35.0	Cluster_07916
413.94000	35.0	Cluster_23350
413.98000	35.0	Cluster_23354
414.71000	35.0	Cluster_23379
414.85000	35.0	Cluster_23397
414.87000	35.0	Cluster_07924
415.22000	35.0	Cluster_23422
415.73000	35.0	Cluster_23434
416.00000	35.0	Cluster_23448
416.22000	35.0	Cluster_23463
416.76000	35.0	Cluster_23476
416.88000	35.0	Cluster_23482
416.94000	35.0	Cluster_01353
418.25000	35.0	Cluster_23534
418.47000	35.0	Cluster_23549
419.18000	35.0	Cluster_01360
419.21000	35.0	Cluster_07978
419.88000	35.0	Cluster_23600
420.19000	35.0	Cluster_23617
420.23000	35.0	Cluster_23612
420.71000	35.0	Cluster_23632
421.56000	35.0	Cluster_08005
422.12000	35.0	Cluster_08008
422.55000	35.0	Cluster_08014
422.58000	35.0	Cluster_08015
423.14000	35.0	Cluster_23727
423.21000	35.0	Cluster_08022
423.24000	35.0	Cluster_23740
423.44000	35.0	Cluster_01387
423.71000	35.0	Cluster_23753
423.74000	35.0	Cluster_23766
424.18000	35.0	Cluster_23787
424.51000	35.0	Cluster_23811
424.68000	35.0	Cluster_23813
425.18000	35.0	Cluster_23850
425.46000	35.0	Cluster_23865
425.87000	35.0	Cluster_08056
425.88000	35.0	Cluster_23895
426.14000	35.0	Cluster_23901
426.24000	35.0	Cluster_23924
426.44000	35.0	Cluster_23942
426.88000	35.0	Cluster_08077
427.51000	35.0	Cluster_08094
428.56000	35.0	Cluster_08107
428.72000	35.0	Cluster_24045
428.79000	35.0	Cluster_24059
429.24000	35.0	Cluster_24074
429.57000	35.0	Cluster_08120

429.72000	35.0	Cluster_24100
429.76000	35.0	Cluster_24093
429.85000	35.0	Cluster_08124
429.95000	35.0	Cluster_01432
430.73000	35.0	Cluster_24136
431.21000	35.0	Cluster_24172
431.41000	35.0	Cluster_24179
431.74000	35.0	Cluster_24194
432.16000	35.0	Cluster_24207
432.21000	35.0	Cluster_24215
432.87000	35.0	Cluster_08164
433.96000	35.0	Cluster_24289
434.79000	35.0	Cluster_24347
434.97000	35.0	Cluster_24350
435.17000	35.0	Cluster_24357
435.24000	35.0	Cluster_08201
435.37000	35.0	Cluster_24383
435.70000	35.0	Cluster_01481
435.97000	35.0	Cluster_01487
436.48000	35.0	Cluster_01491
437.37000	35.0	Cluster_24491
437.46000	35.0	Cluster_01497
438.22000	35.0	Cluster_24532
438.30000	35.0	Cluster_24543
438.96000	35.0	Cluster_01503
438.97000	35.0	Cluster_01504
439.82000	35.0	Cluster_24632
440.21000	35.0	Cluster_01512
440.56000	35.0	Cluster_24655
440.74000	35.0	Cluster_24659
440.86000	35.0	Cluster_08277
441.21000	35.0	Cluster_24682
441.24000	35.0	Cluster_01516
441.73000	35.0	Cluster_24703
441.87000	35.0	Cluster_08293
442.00000	35.0	Cluster_24716
442.28000	35.0	Cluster_24730
442.57000	35.0	Cluster_08303
442.77000	35.0	Cluster_24758
442.89000	35.0	Cluster_24763
442.98000	35.0	Cluster_24761
443.12000	35.0	Cluster_24771
443.22000	35.0	Cluster_24782
443.28000	35.0	Cluster_24785
443.29000	35.0	Cluster_24795
443.89000	35.0	Cluster_08322
443.92000	35.0	Cluster_08324
444.72000	35.0	Cluster_24866
445.18000	35.0	Cluster_08343
445.55000	35.0	Cluster_24900
446.27000	35.0	Cluster_24941

446.73000	35.0	Cluster_24959
446.91000	35.0	Cluster_08369
447.17000	35.0	Cluster_24974
447.25000	35.0	Cluster_08376
447.28000	35.0	Cluster_24982
447.29000	35.0	Cluster_24988
447.60000	35.0	Cluster_08380
448.30000	35.0	Cluster_25022
448.79000	35.0	Cluster_25051
448.97000	35.0	Cluster_01573
449.25000	35.0	Cluster_08416
449.74000	35.0	Cluster_25092
449.84000	35.0	Cluster_25102
450.23000	35.0	Cluster_25125
450.93000	35.0	Cluster_08435
451.14000	35.0	Cluster_25153
451.17000	35.0	Cluster_25168
451.57000	35.0	Cluster_08446
452.13000	35.0	Cluster_25195
452.26000	35.0	Cluster_08462
452.75000	35.0	Cluster_25235
453.14000	35.0	Cluster_25244
453.24000	35.0	Cluster_25248
454.68000	35.0	Cluster_25320
454.70000	35.0	Cluster_25319
454.74000	35.0	Cluster_25325
455.15000	35.0	Cluster_25343
455.18000	35.0	Cluster_01617
455.20000	35.0	Cluster_08518
456.24000	35.0	Cluster_25393
456.26000	35.0	Cluster_08536
456.57000	35.0	Cluster_08539
456.75000	35.0	Cluster_25404
456.78000	35.0	Cluster_25413
457.20000	35.0	Cluster_25422
457.75000	35.0	Cluster_25448
457.76000	35.0	Cluster_25454
458.69000	35.0	Cluster_25493
458.76000	35.0	Cluster_25500
459.22000	35.0	Cluster_08566
459.25000	35.0	Cluster_25514
459.78000	35.0	Cluster_25540
459.90000	35.0	Cluster_08580
460.27000	35.0	Cluster_25572
460.77000	35.0	Cluster_25581
460.97000	35.0	Cluster_01663
461.60000	35.0	Cluster_08592
461.70000	35.0	Cluster_25618
461.73000	35.0	Cluster_25620
462.22000	35.0	Cluster_08608
462.50000	35.0	Cluster_25643

463.61000	35.0	Cluster_25677
463.73000	35.0	Cluster_25692
464.22000	35.0	Cluster_08632
464.30000	35.0	Cluster_25714
464.55000	35.0	Cluster_08640
465.77000	35.0	Cluster_25807
465.95000	35.0	Cluster_08659
465.99000	35.0	Cluster_01692
466.27000	35.0	Cluster_25822
466.90000	35.0	Cluster_08673
467.18000	35.0	Cluster_08677
467.25000	35.0	Cluster_08678
467.30000	35.0	Cluster_25858
467.67000	35.0	Cluster_00144
467.72000	35.0	Cluster_25877
468.89000	35.0	Cluster_08711
469.26000	35.0	Cluster_08720
469.74000	35.0	Cluster_25967
469.88000	35.0	Cluster_08724
470.43000	35.0	Cluster_26002
470.56000	35.0	Cluster_00010
470.92000	35.0	Cluster_08745
471.00000	35.0	Cluster_01731
471.22000	35.0	Cluster_08748
471.25000	35.0	Cluster_01737
471.26000	35.0	Cluster_01738
471.48000	35.0	Cluster_26044
471.74000	35.0	Cluster_26063
471.84000	35.0	Cluster_26052
472.23000	35.0	Cluster_26081
472.24000	35.0	Cluster_08764
472.72000	35.0	Cluster_26107
472.76000	35.0	Cluster_26106
472.77000	35.0	Cluster_26113
473.27000	35.0	Cluster_26146
473.77000	35.0	Cluster_26157
474.54000	35.0	Cluster_08802
474.58000	35.0	Cluster_08800
474.85000	35.0	Cluster_08806
475.58000	35.0	Cluster_08820
475.78000	35.0	Cluster_26234
476.21000	35.0	Cluster_26244
476.23000	35.0	Cluster_08830
476.28000	35.0	Cluster_08832
477.07000	35.0	Cluster_26275
477.20000	35.0	Cluster_08843
477.27000	35.0	Cluster_08840
477.55000	35.0	Cluster_08851
477.61000	35.0	Cluster_08856
477.93000	35.0	Cluster_08861
478.22000	35.0	Cluster_26309

478.25000	35.0	Cluster_26313
478.76000	35.0	Cluster_26329
478.77000	35.0	Cluster_26332
478.93000	35.0	Cluster_08880
478.99000	35.0	Cluster_01780
479.57000	35.0	Cluster_08888
479.71000	35.0	Cluster_26429
479.76000	35.0	Cluster_26423
480.22000	35.0	Cluster_08900
480.70000	35.0	Cluster_26476
481.22000	35.0	Cluster_26493
481.27000	35.0	Cluster_26504
481.28000	35.0	Cluster_08920
481.56000	35.0	Cluster_08922
481.58000	35.0	Cluster_08924
481.72000	35.0	Cluster_26523
481.73000	35.0	Cluster_01804
481.79000	35.0	Cluster_26518
482.20000	35.0	Cluster_01810
482.28000	35.0	Cluster_26547
482.49000	35.0	Cluster_26549
483.24000	35.0	Cluster_26594
483.30000	35.0	Cluster_26603
483.76000	35.0	Cluster_26625
483.84000	35.0	Cluster_26627
484.09000	35.0	Cluster_26635
484.73000	35.0	Cluster_26685
484.76000	35.0	Cluster_26681
485.06000	35.0	Cluster_00166
485.54000	35.0	Cluster_08981
488.29000	35.0	Cluster_26837
488.61000	35.0	Cluster_09032
488.79000	35.0	Cluster_26859
488.95000	35.0	Cluster_09038
489.74000	35.0	Cluster_26904
489.93000	35.0	Cluster_09048
490.50000	35.0	Cluster_26931
490.70000	35.0	Cluster_26935
490.77000	35.0	Cluster_26939
492.55000	35.0	Cluster_09097
492.60000	35.0	Cluster_09099
493.27000	35.0	Cluster_27041
494.26000	35.0	Cluster_27100
495.19000	35.0	Cluster_27149
495.71000	35.0	Cluster_27170
495.95000	35.0	Cluster_09160
496.24000	35.0	Cluster_01895
496.95000	35.0	Cluster_09176
497.35000	35.0	Cluster_27258
497.74000	35.0	Cluster_27265
497.80000	35.0	Cluster_27275

497.94000	35.0	Cluster_09191
498.01000	35.0	Cluster_01929
498.50000	35.0	Cluster_27299
498.73000	35.0	Cluster_27302
499.23000	35.0	Cluster_09211
499.33000	35.0	Cluster_27324
499.76000	35.0	Cluster_01941
499.92000	35.0	Cluster_09218
499.96000	35.0	Cluster_09219
500.02000	35.0	Cluster_27355
500.63000	35.0	Cluster_09231
500.70000	35.0	Cluster_27401
500.96000	35.0	Cluster_01956
501.22000	35.0	Cluster_27436
501.59000	35.0	Cluster_09244
501.76000	35.0	Cluster_27459
502.21000	35.0	Cluster_01970
502.22000	35.0	Cluster_27473
502.59000	35.0	Cluster_09268
502.76000	35.0	Cluster_27485
503.25000	35.0	Cluster_27512
503.28000	35.0	Cluster_27518
504.21000	35.0	Cluster_27547
504.56000	35.0	Cluster_09288
504.98000	35.0	Cluster_01984
505.01000	35.0	Cluster_27567
505.24000	35.0	Cluster_09298
505.52000	35.0	Cluster_01990
505.60000	35.0	Cluster_09305
505.79000	35.0	Cluster_27593
506.73000	35.0	Cluster_27611
506.74000	35.0	Cluster_27621
506.76000	35.0	Cluster_27614
507.25000	35.0	Cluster_27638
507.97000	35.0	Cluster_09350
508.62000	35.0	Cluster_09366
508.70000	35.0	Cluster_27686
508.71000	35.0	Cluster_27692
509.20000	35.0	Cluster_27715
509.26000	35.0	Cluster_27718
509.87000	35.0	Cluster_09377
509.91000	35.0	Cluster_09383
511.23000	35.0	Cluster_27820
511.79000	35.0	Cluster_27852
511.91000	35.0	Cluster_09420
512.25000	35.0	Cluster_27867
512.27000	35.0	Cluster_27865
513.09000	35.0	Cluster_27898
513.77000	35.0	Cluster_27929
514.72000	35.0	Cluster_27964
514.74000	35.0	Cluster_27971

515.25000	35.0	Cluster_02053
515.48000	35.0	Cluster_02061
515.90000	35.0	Cluster_09478
516.74000	35.0	Cluster_28062
517.92000	35.0	Cluster_09503
518.00000	35.0	Cluster_28115
518.26000	35.0	Cluster_09508
518.31000	35.0	Cluster_28130
518.51000	35.0	Cluster_28138
518.59000	35.0	Cluster_09521
518.78000	35.0	Cluster_28152
519.59000	35.0	Cluster_09531
519.81000	35.0	Cluster_28200
520.04000	35.0	Cluster_28202
520.51000	35.0	Cluster_28225
520.80000	35.0	Cluster_28240
520.81000	35.0	Cluster_28232
521.02000	35.0	Cluster_02095
521.26000	35.0	Cluster_09553
521.66000	35.0	Cluster_28271
522.22000	35.0	Cluster_28286
522.45000	35.0	Cluster_02102
522.50000	35.0	Cluster_28303
522.62000	35.0	Cluster_09582
522.75000	35.0	Cluster_28315
523.22000	35.0	Cluster_28350
523.27000	35.0	Cluster_28334
523.35000	35.0	Cluster_28353
523.92000	35.0	Cluster_09603
523.97000	35.0	Cluster_28374
524.28000	35.0	Cluster_28386
526.18000	35.0	Cluster_28467
526.20000	35.0	Cluster_09656
526.93000	35.0	Cluster_09676
526.95000	35.0	Cluster_09677
527.76000	35.0	Cluster_28507
528.23000	35.0	Cluster_28536
528.27000	35.0	Cluster_00184
528.32000	35.0	Cluster_28539
528.36000	35.0	Cluster_28552
528.97000	35.0	Cluster_09710
529.25000	35.0	Cluster_09712
529.79000	35.0	Cluster_28615
530.20000	35.0	Cluster_28629
530.26000	35.0	Cluster_02153
530.51000	35.0	Cluster_28657
530.81000	35.0	Cluster_28677
530.94000	35.0	Cluster_09741
531.75000	35.0	Cluster_28707
531.77000	35.0	Cluster_28708
532.24000	35.0	Cluster_09763

532.27000	35.0	Cluster_09766
532.62000	35.0	Cluster_09768
532.97000	35.0	Cluster_09773
533.15000	35.0	Cluster_28752
533.16000	35.0	Cluster_28753
533.19000	35.0	Cluster_09774
533.32000	35.0	Cluster_09778
533.70000	35.0	Cluster_28783
533.82000	35.0	Cluster_28799
533.91000	35.0	Cluster_09792
534.28000	35.0	Cluster_28807
534.59000	35.0	Cluster_09803
535.32000	35.0	Cluster_09812
535.80000	35.0	Cluster_28867
535.94000	35.0	Cluster_09822
536.06000	35.0	Cluster_28878
536.26000	35.0	Cluster_09823
536.27000	35.0	Cluster_28888
536.48000	35.0	Cluster_28899
536.60000	35.0	Cluster_09834
537.23000	35.0	Cluster_09849
537.56000	35.0	Cluster_09853
537.69000	35.0	Cluster_28943
537.91000	35.0	Cluster_09869
538.84000	35.0	Cluster_28980
538.97000	35.0	Cluster_09886
538.98000	35.0	Cluster_09885
539.01000	35.0	Cluster_28985
539.28000	35.0	Cluster_28993
539.52000	35.0	Cluster_29001
540.02000	35.0	Cluster_09899
540.27000	35.0	Cluster_29031
540.79000	35.0	Cluster_29052
541.34000	35.0	Cluster_29085
541.44000	35.0	Cluster_29098
541.82000	35.0	Cluster_29115
542.26000	35.0	Cluster_02236
542.28000	35.0	Cluster_02229
542.52000	35.0	Cluster_02233
542.77000	35.0	Cluster_29162
543.27000	35.0	Cluster_29193
544.95000	35.0	Cluster_09982
545.77000	35.0	Cluster_29280
545.92000	35.0	Cluster_09992
546.31000	35.0	Cluster_29312
547.32000	35.0	Cluster_29345
548.27000	35.0	Cluster_29389
548.46000	35.0	Cluster_00194
548.61000	35.0	Cluster_10035
548.64000	35.0	Cluster_10037
549.25000	35.0	Cluster_10044

549.28000	35.0	Cluster_29418
549.90000	35.0	Cluster_10052
549.99000	35.0	Cluster_10058
550.22000	35.0	Cluster_29451
550.31000	35.0	Cluster_29461
550.55000	35.0	Cluster_10069
550.61000	35.0	Cluster_10068
550.78000	35.0	Cluster_29465
550.84000	35.0	Cluster_29476
551.01000	35.0	Cluster_29483
551.29000	35.0	Cluster_29499
551.57000	35.0	Cluster_10082
551.97000	35.0	Cluster_10089
552.36000	35.0	Cluster_29545
552.74000	35.0	Cluster_29553
552.75000	35.0	Cluster_29548
552.96000	35.0	Cluster_10104
553.55000	35.0	Cluster_29579
553.63000	35.0	Cluster_10116
554.58000	35.0	Cluster_10130
554.60000	35.0	Cluster_10136
554.97000	35.0	Cluster_10144
556.25000	35.0	Cluster_29692
556.68000	35.0	Cluster_10177
556.96000	35.0	Cluster_10184
557.73000	35.0	Cluster_29750
558.23000	35.0	Cluster_10209
558.65000	35.0	Cluster_10215
558.81000	35.0	Cluster_29792
559.29000	35.0	Cluster_29811
560.35000	35.0	Cluster_29845
560.65000	35.0	Cluster_10245
560.83000	35.0	Cluster_29862
561.28000	35.0	Cluster_29879
561.64000	35.0	Cluster_10262
561.70000	35.0	Cluster_29892
561.81000	35.0	Cluster_29901
561.98000	35.0	Cluster_10266
562.07000	35.0	Cluster_29906
562.29000	35.0	Cluster_29915
563.32000	35.0	Cluster_10288
563.50000	35.0	Cluster_02355
563.75000	35.0	Cluster_29975
563.76000	35.0	Cluster_29965
564.78000	35.0	Cluster_30003
564.79000	35.0	Cluster_02366
565.56000	35.0	Cluster_30039
565.60000	35.0	Cluster_10318
566.33000	35.0	Cluster_30080
566.74000	35.0	Cluster_02376
566.79000	35.0	Cluster_30088

566.90000	35.0	Cluster_30092
566.98000	35.0	Cluster_10333
567.07000	35.0	Cluster_30105
567.24000	35.0	Cluster_30110
567.30000	35.0	Cluster_30123
567.31000	35.0	Cluster_30114
568.56000	35.0	Cluster_02384
568.73000	35.0	Cluster_30166
568.79000	35.0	Cluster_30177
568.80000	35.0	Cluster_30176
568.85000	35.0	Cluster_30178
569.06000	35.0	Cluster_02386
569.57000	35.0	Cluster_02390
569.82000	35.0	Cluster_30204
570.30000	35.0	Cluster_02396
570.36000	35.0	Cluster_30219
570.73000	35.0	Cluster_02397
570.77000	35.0	Cluster_30236
571.22000	35.0	Cluster_30252
571.34000	35.0	Cluster_30256
571.64000	35.0	Cluster_10409
571.77000	35.0	Cluster_30264
572.13000	35.0	Cluster_00213
572.30000	35.0	Cluster_10420
572.79000	35.0	Cluster_30301
573.35000	35.0	Cluster_30317
574.26000	35.0	Cluster_10456
574.80000	35.0	Cluster_30355
575.25000	35.0	Cluster_02433
575.33000	35.0	Cluster_02435
576.55000	35.0	Cluster_02439
576.77000	35.0	Cluster_30417
576.98000	35.0	Cluster_10494
577.30000	35.0	Cluster_10500
577.81000	35.0	Cluster_30446
578.32000	35.0	Cluster_30460
578.55000	35.0	Cluster_02454
578.86000	35.0	Cluster_30486
578.98000	35.0	Cluster_10523
579.33000	35.0	Cluster_30494
579.55000	35.0	Cluster_02460
579.61000	35.0	Cluster_10531
579.63000	35.0	Cluster_10533
579.77000	35.0	Cluster_30512
579.81000	35.0	Cluster_30517
579.83000	35.0	Cluster_30515
580.36000	35.0	Cluster_30555
580.86000	35.0	Cluster_02466
581.28000	35.0	Cluster_02468
581.31000	35.0	Cluster_30577
581.96000	35.0	Cluster_10563

582.31000	35.0	Cluster_30648
582.54000	35.0	Cluster_02478
582.65000	35.0	Cluster_10574
582.70000	35.0	Cluster_00226
582.74000	35.0	Cluster_30654
584.05000	35.0	Cluster_30718
584.31000	35.0	Cluster_30724
584.54000	35.0	Cluster_30733
585.02000	35.0	Cluster_30763
585.29000	35.0	Cluster_10612
585.35000	35.0	Cluster_02501
585.80000	35.0	Cluster_30791
585.84000	35.0	Cluster_30792
586.62000	35.0	Cluster_10630
586.75000	35.0	Cluster_30815
587.30000	35.0	Cluster_10642
587.33000	35.0	Cluster_30848
587.34000	35.0	Cluster_30837
587.65000	35.0	Cluster_10646
588.69000	35.0	Cluster_30892
588.82000	35.0	Cluster_30900
589.55000	35.0	Cluster_30932
589.74000	35.0	Cluster_30933
589.96000	35.0	Cluster_10677
590.22000	35.0	Cluster_30952
590.30000	35.0	Cluster_30953
591.04000	35.0	Cluster_30998
591.44000	35.0	Cluster_31008
591.52000	35.0	Cluster_02538
591.66000	35.0	Cluster_10703
591.81000	35.0	Cluster_31027
592.01000	35.0	Cluster_10706
592.79000	35.0	Cluster_31056
592.82000	35.0	Cluster_31060
592.91000	35.0	Cluster_31063
593.06000	35.0	Cluster_02547
593.07000	35.0	Cluster_02548
593.33000	35.0	Cluster_10724
593.34000	35.0	Cluster_31073
593.93000	35.0	Cluster_10743
594.32000	35.0	Cluster_10753
595.59000	35.0	Cluster_31146
595.62000	35.0	Cluster_10769
596.75000	35.0	Cluster_31177
597.67000	35.0	Cluster_10808
597.81000	35.0	Cluster_31212
597.82000	35.0	Cluster_31208
598.29000	35.0	Cluster_10814
598.36000	35.0	Cluster_10818
599.36000	35.0	Cluster_31267
599.66000	35.0	Cluster_10840

599.95000	35.0	Cluster_10848
600.81000	35.0	Cluster_31316
600.97000	35.0	Cluster_10858
601.36000	35.0	Cluster_31339
602.32000	35.0	Cluster_31390
602.83000	35.0	Cluster_31406
603.30000	35.0	Cluster_31409
603.51000	35.0	Cluster_02598
603.53000	35.0	Cluster_31419
603.96000	35.0	Cluster_10888
604.80000	35.0	Cluster_31463
605.31000	35.0	Cluster_31473
605.67000	35.0	Cluster_10910
606.07000	35.0	Cluster_31501
606.27000	35.0	Cluster_31514
606.87000	35.0	Cluster_31531
606.89000	35.0	Cluster_10941
607.32000	35.0	Cluster_31536
608.09000	35.0	Cluster_02626
608.32000	35.0	Cluster_31587
608.49000	35.0	Cluster_31590
608.78000	35.0	Cluster_31595
608.79000	35.0	Cluster_31601
609.06000	35.0	Cluster_31605
609.81000	35.0	Cluster_31625
610.31000	35.0	Cluster_31655
611.34000	35.0	Cluster_31685
612.32000	35.0	Cluster_11010
613.83000	35.0	Cluster_31794
614.06000	35.0	Cluster_31796
614.07000	35.0	Cluster_02670
614.19000	35.0	Cluster_31797
614.26000	35.0	Cluster_11035
615.83000	35.0	Cluster_31852
616.08000	35.0	Cluster_02680
616.32000	35.0	Cluster_11075
616.55000	35.0	Cluster_31873
616.83000	35.0	Cluster_31882
617.02000	35.0	Cluster_31892
617.35000	35.0	Cluster_31911
617.93000	35.0	Cluster_31935
618.05000	35.0	Cluster_02693
618.79000	35.0	Cluster_31952
619.29000	35.0	Cluster_02701
619.33000	35.0	Cluster_31985
619.62000	35.0	Cluster_11116
620.53000	35.0	Cluster_32028
620.60000	35.0	Cluster_11128
621.30000	35.0	Cluster_32049
621.36000	35.0	Cluster_32053
621.39000	35.0	Cluster_32052

621.79000	35.0	Cluster_32057
621.98000	35.0	Cluster_11154
622.78000	35.0	Cluster_32095
622.98000	35.0	Cluster_11175
623.28000	35.0	Cluster_32112
623.36000	35.0	Cluster_32126
623.65000	35.0	Cluster_11191
623.98000	35.0	Cluster_11189
624.20000	35.0	Cluster_32153
624.28000	35.0	Cluster_32154
624.63000	35.0	Cluster_11202
625.04000	35.0	Cluster_02732
625.32000	35.0	Cluster_11203
625.58000	35.0	Cluster_02735
626.00000	35.0	Cluster_11225
626.07000	35.0	Cluster_32208
626.27000	35.0	Cluster_32218
626.28000	35.0	Cluster_32212
626.83000	35.0	Cluster_32244
627.30000	35.0	Cluster_32266
627.31000	35.0	Cluster_32263
627.50000	35.0	Cluster_32275
627.79000	35.0	Cluster_32283
628.07000	35.0	Cluster_32292
628.86000	35.0	Cluster_32321
629.63000	35.0	Cluster_11289
629.83000	35.0	Cluster_32344
629.96000	35.0	Cluster_11292
630.84000	35.0	Cluster_32375
631.37000	35.0	Cluster_11312
631.80000	35.0	Cluster_32410
632.05000	35.0	Cluster_11324
632.35000	35.0	Cluster_11329
632.78000	35.0	Cluster_32445
632.84000	35.0	Cluster_32450
633.00000	35.0	Cluster_11339
633.32000	35.0	Cluster_32465
633.36000	35.0	Cluster_32472
635.07000	35.0	Cluster_02772
635.82000	35.0	Cluster_02775
636.32000	35.0	Cluster_32584
636.84000	35.0	Cluster_32596
637.62000	35.0	Cluster_11403
637.83000	35.0	Cluster_32632
638.35000	35.0	Cluster_32655
638.36000	35.0	Cluster_11416
638.39000	35.0	Cluster_32659
638.79000	35.0	Cluster_32669
638.85000	35.0	Cluster_32673
639.85000	35.0	Cluster_32724
639.99000	35.0	Cluster_11446

640.32000	35.0	Cluster_32732
641.64000	35.0	Cluster_11472
641.84000	35.0	Cluster_32794
642.02000	35.0	Cluster_11476
643.07000	35.0	Cluster_32838
643.83000	35.0	Cluster_32863
644.00000	35.0	Cluster_11510
644.30000	35.0	Cluster_02836
644.36000	35.0	Cluster_32878
644.37000	35.0	Cluster_11511
645.00000	35.0	Cluster_11529
645.85000	35.0	Cluster_32926
646.30000	35.0	Cluster_32945
648.34000	35.0	Cluster_33005
648.67000	35.0	Cluster_11566
648.88000	35.0	Cluster_33029
649.01000	35.0	Cluster_11574
649.77000	35.0	Cluster_33054
650.31000	35.0	Cluster_33085
651.01000	35.0	Cluster_11595
651.08000	35.0	Cluster_33114
651.32000	35.0	Cluster_33124
651.36000	35.0	Cluster_11600
651.86000	35.0	Cluster_33158
651.99000	35.0	Cluster_11614
652.06000	35.0	Cluster_33159
652.35000	35.0	Cluster_33162
652.86000	35.0	Cluster_33187
653.33000	35.0	Cluster_33199
653.57000	35.0	Cluster_33207
653.60000	35.0	Cluster_02883
654.05000	35.0	Cluster_33221
654.68000	35.0	Cluster_11655
654.78000	35.0	Cluster_33237
655.99000	35.0	Cluster_11675
656.63000	35.0	Cluster_11676
657.90000	35.0	Cluster_33331
657.95000	35.0	Cluster_33350
658.07000	35.0	Cluster_33340
658.66000	35.0	Cluster_11699
659.28000	35.0	Cluster_33386
659.60000	35.0	Cluster_33396
659.63000	35.0	Cluster_02907
659.98000	35.0	Cluster_11725
660.33000	35.0	Cluster_33416
660.46000	35.0	Cluster_33423
660.84000	35.0	Cluster_33430
661.01000	35.0	Cluster_11741
661.31000	35.0	Cluster_33436
662.02000	35.0	Cluster_11748
662.12000	35.0	Cluster_02928

662.30000	35.0	Cluster_33472
663.37000	35.0	Cluster_33509
663.55000	35.0	Cluster_02941
663.60000	35.0	Cluster_02940
665.32000	35.0	Cluster_33570
666.35000	35.0	Cluster_02964
666.68000	35.0	Cluster_11814
666.95000	35.0	Cluster_33617
667.82000	35.0	Cluster_33644
668.33000	35.0	Cluster_33651
668.35000	35.0	Cluster_11848
669.10000	35.0	Cluster_02977
669.33000	35.0	Cluster_33680
669.78000	35.0	Cluster_33695
670.01000	35.0	Cluster_11876
670.40000	35.0	Cluster_33725
670.80000	35.0	Cluster_33734
670.82000	35.0	Cluster_33742
670.87000	35.0	Cluster_02986
671.44000	35.0	Cluster_33760
671.82000	35.0	Cluster_33770
671.97000	35.0	Cluster_11894
672.35000	35.0	Cluster_33781
672.81000	35.0	Cluster_33789
673.09000	35.0	Cluster_03005
673.37000	35.0	Cluster_33802
673.62000	35.0	Cluster_33824
673.65000	35.0	Cluster_11927
674.11000	35.0	Cluster_03014
674.32000	35.0	Cluster_11933
674.95000	35.0	Cluster_00274
675.37000	35.0	Cluster_33861
675.60000	35.0	Cluster_33864
675.64000	35.0	Cluster_03025
675.84000	35.0	Cluster_03026
676.00000	35.0	Cluster_11956
676.32000	35.0	Cluster_11959
677.20000	35.0	Cluster_33905
677.89000	35.0	Cluster_33946
678.81000	35.0	Cluster_33981
678.84000	35.0	Cluster_33980
678.85000	35.0	Cluster_03050
679.38000	35.0	Cluster_33996
679.79000	35.0	Cluster_34007
679.86000	35.0	Cluster_34018
680.08000	35.0	Cluster_03060
680.09000	35.0	Cluster_34023
680.87000	35.0	Cluster_34054
681.08000	35.0	Cluster_03066
681.32000	35.0	Cluster_12020
681.35000	35.0	Cluster_34074

682.09000	35.0	Cluster_34089
682.31000	35.0	Cluster_34106
682.38000	35.0	Cluster_03074
683.61000	35.0	Cluster_12059
683.81000	35.0	Cluster_34134
683.99000	35.0	Cluster_12062
684.08000	35.0	Cluster_03088
684.82000	35.0	Cluster_34171
684.83000	35.0	Cluster_34174
685.09000	35.0	Cluster_03095
685.10000	35.0	Cluster_03102
685.35000	35.0	Cluster_34201
686.04000	35.0	Cluster_12086
686.50000	35.0	Cluster_00285
686.75000	35.0	Cluster_00286
687.66000	35.0	Cluster_34255
689.34000	35.0	Cluster_34298
689.36000	35.0	Cluster_34295
689.57000	35.0	Cluster_03142
690.82000	35.0	Cluster_34324
690.84000	35.0	Cluster_34327
691.59000	35.0	Cluster_03162
691.79000	35.0	Cluster_34357
691.83000	35.0	Cluster_34369
692.26000	35.0	Cluster_34373
692.32000	35.0	Cluster_03167
692.35000	35.0	Cluster_03168
692.60000	35.0	Cluster_03182
692.84000	35.0	Cluster_34402
693.56000	35.0	Cluster_03192
693.60000	35.0	Cluster_34413
693.88000	35.0	Cluster_34418
694.36000	35.0	Cluster_34424
694.84000	35.0	Cluster_34436
694.87000	35.0	Cluster_34443
695.01000	35.0	Cluster_12183
695.36000	35.0	Cluster_34458
695.70000	35.0	Cluster_12190
695.87000	35.0	Cluster_34468
696.32000	35.0	Cluster_34489
696.34000	35.0	Cluster_03210
696.39000	35.0	Cluster_34483
696.58000	35.0	Cluster_03211
697.31000	35.0	Cluster_03215
697.37000	35.0	Cluster_34515
697.85000	35.0	Cluster_34520
697.87000	35.0	Cluster_34523
698.82000	35.0	Cluster_34541
698.83000	35.0	Cluster_34546
699.70000	35.0	Cluster_12244
699.85000	35.0	Cluster_03235

700.26000	35.0	Cluster_34580
700.36000	35.0	Cluster_34589
700.65000	35.0	Cluster_34590
700.85000	35.0	Cluster_34594
701.36000	35.0	Cluster_34614
702.34000	35.0	Cluster_34653
702.62000	35.0	Cluster_12286
703.04000	35.0	Cluster_12291
703.05000	35.0	Cluster_03266
703.33000	35.0	Cluster_34677
703.69000	35.0	Cluster_12310
703.91000	35.0	Cluster_03274
704.17000	35.0	Cluster_34698
704.21000	35.0	Cluster_34700
704.31000	35.0	Cluster_34706
704.81000	35.0	Cluster_34728
704.85000	35.0	Cluster_34734
705.04000	35.0	Cluster_12319
705.37000	35.0	Cluster_34742
705.83000	35.0	Cluster_34756
705.88000	35.0	Cluster_34757
706.86000	35.0	Cluster_03301
707.02000	35.0	Cluster_12346
707.80000	35.0	Cluster_34791
708.51000	35.0	Cluster_00309
709.28000	35.0	Cluster_34840
709.39000	35.0	Cluster_34846
710.88000	35.0	Cluster_03335
711.06000	35.0	Cluster_12397
711.85000	35.0	Cluster_34925
712.39000	35.0	Cluster_34948
712.84000	35.0	Cluster_34967
713.08000	35.0	Cluster_03351
713.29000	35.0	Cluster_03356
713.38000	35.0	Cluster_12431
713.72000	35.0	Cluster_12437
713.77000	35.0	Cluster_34987
713.92000	35.0	Cluster_34998
714.32000	35.0	Cluster_35004
714.36000	35.0	Cluster_35010
715.38000	35.0	Cluster_35041
715.54000	35.0	Cluster_00320
715.71000	35.0	Cluster_12456
715.85000	35.0	Cluster_35046
715.99000	35.0	Cluster_12465
716.39000	35.0	Cluster_35061
717.36000	35.0	Cluster_12478
717.90000	35.0	Cluster_35102
718.36000	35.0	Cluster_35122
718.85000	35.0	Cluster_35127
718.89000	35.0	Cluster_35137

718.91000	35.0	Cluster_35135
718.96000	35.0	Cluster_12511
719.39000	35.0	Cluster_35145
719.83000	35.0	Cluster_35161
719.85000	35.0	Cluster_03411
719.98000	35.0	Cluster_12519
720.12000	35.0	Cluster_35168
720.13000	35.0	Cluster_35170
720.35000	35.0	Cluster_03417
720.58000	35.0	Cluster_03421
720.83000	35.0	Cluster_35178
721.32000	35.0	Cluster_35195
721.41000	35.0	Cluster_35191
721.72000	35.0	Cluster_12548
721.88000	35.0	Cluster_35209
721.90000	35.0	Cluster_35213
722.02000	35.0	Cluster_12553
722.09000	35.0	Cluster_03436
722.34000	35.0	Cluster_03433
722.68000	35.0	Cluster_12565
723.40000	35.0	Cluster_12568
723.42000	35.0	Cluster_12570
723.95000	35.0	Cluster_00326
724.04000	35.0	Cluster_12575
724.90000	35.0	Cluster_03465
725.06000	35.0	Cluster_12593
725.32000	35.0	Cluster_03469
725.41000	35.0	Cluster_35286
725.42000	35.0	Cluster_35293
727.38000	35.0	Cluster_35317
728.10000	35.0	Cluster_03487
728.13000	35.0	Cluster_03484
728.42000	35.0	Cluster_35338
728.98000	35.0	Cluster_12653
729.15000	35.0	Cluster_03497
729.84000	35.0	Cluster_35373
729.89000	35.0	Cluster_03507
730.91000	35.0	Cluster_35408
731.10000	35.0	Cluster_35410
731.83000	35.0	Cluster_03518
732.11000	35.0	Cluster_03522
732.64000	35.0	Cluster_03534
733.03000	35.0	Cluster_12708
733.13000	35.0	Cluster_03548
733.83000	35.0	Cluster_35471
733.85000	35.0	Cluster_35474
734.58000	35.0	Cluster_35493
735.04000	35.0	Cluster_12729
735.74000	35.0	Cluster_12741
736.37000	35.0	Cluster_35541
737.04000	35.0	Cluster_12754

737.16000	35.0	Cluster_35560
737.82000	35.0	Cluster_35593
738.39000	35.0	Cluster_35611
738.63000	35.0	Cluster_03590
739.38000	35.0	Cluster_12791
739.39000	35.0	Cluster_12789
739.84000	35.0	Cluster_35645
740.37000	35.0	Cluster_35657
740.38000	35.0	Cluster_35661
740.93000	35.0	Cluster_35676
741.85000	35.0	Cluster_35683
742.37000	35.0	Cluster_35697
742.67000	35.0	Cluster_12831
742.84000	35.0	Cluster_35707
744.37000	35.0	Cluster_35750
745.38000	35.0	Cluster_35777
745.68000	35.0	Cluster_12857
746.06000	35.0	Cluster_12863
746.15000	35.0	Cluster_35792
746.30000	35.0	Cluster_35797
747.12000	35.0	Cluster_35817
747.37000	35.0	Cluster_35818
748.00000	35.0	Cluster_12903
748.86000	35.0	Cluster_03654
748.88000	35.0	Cluster_35857
749.07000	35.0	Cluster_12920
749.69000	35.0	Cluster_12929
749.98000	35.0	Cluster_12936
750.01000	35.0	Cluster_12941
750.38000	35.0	Cluster_35883
750.44000	35.0	Cluster_35886
750.94000	35.0	Cluster_35903
751.06000	35.0	Cluster_12961
751.15000	35.0	Cluster_03662
751.36000	35.0	Cluster_35908
752.37000	35.0	Cluster_35932
753.05000	35.0	Cluster_12988
753.06000	35.0	Cluster_12984
753.39000	35.0	Cluster_35964
753.91000	35.0	Cluster_35981
754.06000	35.0	Cluster_13009
754.90000	35.0	Cluster_36004
755.06000	35.0	Cluster_13021
755.30000	35.0	Cluster_13019
755.33000	35.0	Cluster_13020
755.42000	35.0	Cluster_36013
755.75000	35.0	Cluster_13034
755.92000	35.0	Cluster_36043
756.13000	35.0	Cluster_03688
757.31000	35.0	Cluster_36071
757.73000	35.0	Cluster_13076

758.41000	35.0	Cluster_36102
758.70000	35.0	Cluster_13088
759.63000	35.0	Cluster_03704
759.90000	35.0	Cluster_36129
759.91000	35.0	Cluster_36132
760.35000	35.0	Cluster_36146
761.05000	35.0	Cluster_00024
761.41000	35.0	Cluster_36182
762.38000	35.0	Cluster_13130
763.03000	35.0	Cluster_13141
763.08000	35.0	Cluster_13137
763.35000	35.0	Cluster_03726
763.71000	35.0	Cluster_13147
764.09000	35.0	Cluster_13148
764.36000	35.0	Cluster_36253
765.15000	35.0	Cluster_03741
765.79000	35.0	Cluster_36274
765.80000	35.0	Cluster_36275
766.37000	35.0	Cluster_36292
766.55000	35.0	Cluster_00339
767.70000	35.0	Cluster_13203
767.85000	35.0	Cluster_36325
768.07000	35.0	Cluster_13209
768.08000	35.0	Cluster_13205
768.38000	35.0	Cluster_36351
769.33000	35.0	Cluster_36370
769.59000	35.0	Cluster_36368
769.82000	35.0	Cluster_36385
772.41000	35.0	Cluster_36441
772.74000	35.0	Cluster_13265
772.80000	35.0	Cluster_13273
772.88000	35.0	Cluster_36459
772.92000	35.0	Cluster_36460
773.08000	35.0	Cluster_03785
773.09000	35.0	Cluster_03786
773.13000	35.0	Cluster_13272
773.35000	35.0	Cluster_13283
773.41000	35.0	Cluster_13286
774.12000	35.0	Cluster_36481
774.73000	35.0	Cluster_13306
775.06000	35.0	Cluster_13312
775.07000	35.0	Cluster_13311
775.42000	35.0	Cluster_36510
776.37000	35.0	Cluster_03803
777.15000	35.0	Cluster_03806
777.35000	35.0	Cluster_36561
777.38000	35.0	Cluster_36567
777.39000	35.0	Cluster_36564
778.37000	35.0	Cluster_36591
778.77000	35.0	Cluster_13350
778.87000	35.0	Cluster_36605

780.73000	35.0	Cluster_13379
780.81000	35.0	Cluster_36636
780.88000	35.0	Cluster_03821
781.40000	35.0	Cluster_36661
782.03000	35.0	Cluster_13403
783.13000	35.0	Cluster_03834
783.46000	35.0	Cluster_36708
783.72000	35.0	Cluster_13422
784.06000	35.0	Cluster_13433
784.89000	35.0	Cluster_36733
784.93000	35.0	Cluster_36732
785.45000	35.0	Cluster_36744
785.83000	35.0	Cluster_36763
787.06000	35.0	Cluster_13459
787.12000	35.0	Cluster_03868
787.35000	35.0	Cluster_03870
788.87000	35.0	Cluster_36840
789.06000	35.0	Cluster_13483
790.87000	35.0	Cluster_36906
791.12000	35.0	Cluster_36909
791.38000	35.0	Cluster_36914
791.90000	35.0	Cluster_36919
792.38000	35.0	Cluster_36933
792.64000	35.0	Cluster_36941
792.86000	35.0	Cluster_03914
792.87000	35.0	Cluster_36948
792.97000	35.0	Cluster_13541
793.78000	35.0	Cluster_13547
794.36000	35.0	Cluster_36984
794.37000	35.0	Cluster_36982
794.45000	35.0	Cluster_36985
794.65000	35.0	Cluster_36986
795.07000	35.0	Cluster_13571
795.42000	35.0	Cluster_37004
795.44000	35.0	Cluster_37000
795.68000	35.0	Cluster_13585
795.87000	35.0	Cluster_03947
795.90000	35.0	Cluster_37017
795.96000	35.0	Cluster_37020
796.41000	35.0	Cluster_13605
796.72000	35.0	Cluster_13597
796.91000	35.0	Cluster_37044
797.86000	35.0	Cluster_37077
797.87000	35.0	Cluster_37073
797.88000	35.0	Cluster_37072
797.89000	35.0	Cluster_37068
799.38000	35.0	Cluster_37115
799.40000	35.0	Cluster_37112
799.89000	35.0	Cluster_03975
800.35000	35.0	Cluster_37146
800.41000	35.0	Cluster_13643

800.84000	35.0	Cluster_37149
800.86000	35.0	Cluster_03991
801.06000	35.0	Cluster_00029
801.37000	35.0	Cluster_03995
801.42000	35.0	Cluster_37163
801.44000	35.0	Cluster_13660
802.43000	35.0	Cluster_37188
802.74000	35.0	Cluster_13681
803.41000	35.0	Cluster_13684
803.88000	35.0	Cluster_37226
804.43000	35.0	Cluster_37243
804.91000	35.0	Cluster_04015
805.16000	35.0	Cluster_37269
805.39000	35.0	Cluster_37283
805.94000	35.0	Cluster_37284
806.39000	35.0	Cluster_37304
807.01000	35.0	Cluster_13723
807.46000	35.0	Cluster_37330
807.92000	35.0	Cluster_37352
808.85000	35.0	Cluster_37370
808.91000	35.0	Cluster_37377
809.40000	35.0	Cluster_04037
809.91000	35.0	Cluster_37401
810.39000	35.0	Cluster_37407
810.44000	35.0	Cluster_13774
811.74000	35.0	Cluster_00036
811.86000	35.0	Cluster_37446
811.91000	35.0	Cluster_37441
812.39000	35.0	Cluster_37458
812.41000	35.0	Cluster_13796
813.82000	35.0	Cluster_00352
813.90000	35.0	Cluster_37483
814.10000	35.0	Cluster_13827
814.16000	35.0	Cluster_37492
814.17000	35.0	Cluster_04067
814.90000	35.0	Cluster_04074
815.06000	35.0	Cluster_13832
815.79000	35.0	Cluster_13844
815.92000	35.0	Cluster_37530
816.38000	35.0	Cluster_37540
816.43000	35.0	Cluster_04088
816.72000	35.0	Cluster_13857
817.34000	35.0	Cluster_37561
817.88000	35.0	Cluster_37570
818.42000	35.0	Cluster_13883
819.05000	35.0	Cluster_13897
819.42000	35.0	Cluster_13906
820.17000	35.0	Cluster_04104
821.23000	35.0	Cluster_04111
821.38000	35.0	Cluster_37637
822.06000	35.0	Cluster_13934

823.42000	35.0	Cluster_37669
823.91000	35.0	Cluster_37674
823.94000	35.0	Cluster_37688
825.32000	35.0	Cluster_37717
825.39000	35.0	Cluster_04137
826.38000	35.0	Cluster_13981
826.86000	35.0	Cluster_37765
827.04000	35.0	Cluster_13986
827.06000	35.0	Cluster_13988
827.09000	35.0	Cluster_13989
827.88000	35.0	Cluster_37794
828.80000	35.0	Cluster_14000
828.98000	35.0	Cluster_14004
829.16000	35.0	Cluster_04165
829.36000	35.0	Cluster_37821
829.43000	35.0	Cluster_37825
829.91000	35.0	Cluster_04169
830.74000	35.0	Cluster_14020
830.89000	35.0	Cluster_00042
831.45000	35.0	Cluster_37853
832.41000	35.0	Cluster_37873
832.68000	35.0	Cluster_37879
833.49000	35.0	Cluster_37894
833.99000	35.0	Cluster_37902
834.10000	35.0	Cluster_14054
834.94000	35.0	Cluster_37918
835.05000	35.0	Cluster_14062
836.39000	35.0	Cluster_14076
836.42000	35.0	Cluster_14077
836.76000	35.0	Cluster_14083
836.86000	35.0	Cluster_37959
836.94000	35.0	Cluster_04234
837.41000	35.0	Cluster_37970
837.95000	35.0	Cluster_37976
839.10000	35.0	Cluster_14102
840.66000	35.0	Cluster_04260
840.78000	35.0	Cluster_14117
841.08000	35.0	Cluster_14128
841.10000	35.0	Cluster_14126
841.67000	35.0	Cluster_00364
841.76000	35.0	Cluster_38066
842.73000	35.0	Cluster_14140
843.87000	35.0	Cluster_38114
844.18000	35.0	Cluster_04298
844.44000	35.0	Cluster_14157
846.98000	35.0	Cluster_38180
847.48000	35.0	Cluster_38200
847.75000	35.0	Cluster_14185
847.88000	35.0	Cluster_38203
848.40000	35.0	Cluster_04350
848.91000	35.0	Cluster_04352

849.46000	35.0	Cluster_38235
849.64000	35.0	Cluster_04364
849.97000	35.0	Cluster_38245
849.99000	35.0	Cluster_38248
850.33000	35.0	Cluster_38250
850.40000	35.0	Cluster_04376
850.76000	35.0	Cluster_14213
851.36000	35.0	Cluster_14215
851.77000	35.0	Cluster_38298
852.03000	35.0	Cluster_38307
852.91000	35.0	Cluster_04400
852.93000	35.0	Cluster_04401
853.45000	35.0	Cluster_38334
854.44000	35.0	Cluster_38377
854.47000	35.0	Cluster_38375
854.88000	35.0	Cluster_38385
855.41000	35.0	Cluster_38392
855.73000	35.0	Cluster_14240
856.43000	35.0	Cluster_14249
856.91000	35.0	Cluster_04444
857.06000	35.0	Cluster_14257
857.89000	35.0	Cluster_38459
857.91000	35.0	Cluster_04449
858.07000	35.0	Cluster_14269
858.77000	35.0	Cluster_14275
859.45000	35.0	Cluster_38500
859.47000	35.0	Cluster_38497
860.47000	35.0	Cluster_14284
861.39000	35.0	Cluster_04493
862.39000	35.0	Cluster_04505
862.49000	35.0	Cluster_38591
863.05000	35.0	Cluster_14302
863.76000	35.0	Cluster_14307
863.94000	35.0	Cluster_38615
864.07000	35.0	Cluster_14314
865.03000	35.0	Cluster_38646
865.75000	35.0	Cluster_14326
865.92000	35.0	Cluster_38659
865.93000	35.0	Cluster_04555
866.41000	35.0	Cluster_14334
869.07000	35.0	Cluster_14353
869.28000	35.0	Cluster_38719
869.41000	35.0	Cluster_04599
869.78000	35.0	Cluster_14363
871.17000	35.0	Cluster_38758
871.27000	35.0	Cluster_38759
871.44000	35.0	Cluster_38769
871.47000	35.0	Cluster_14388
872.27000	35.0	Cluster_38802
874.09000	35.0	Cluster_14414
874.26000	35.0	Cluster_38844

874.78000	35.0	Cluster_14424
875.28000	35.0	Cluster_38860
875.66000	35.0	Cluster_04698
876.08000	35.0	Cluster_14437
876.53000	35.0	Cluster_38895
877.38000	35.0	Cluster_14453
877.44000	35.0	Cluster_38909
878.48000	35.0	Cluster_38928
880.46000	35.0	Cluster_14483
881.96000	35.0	Cluster_38993
882.44000	35.0	Cluster_39008
882.94000	35.0	Cluster_39018
884.40000	35.0	Cluster_14528
884.66000	35.0	Cluster_39047
885.12000	35.0	Cluster_14544
885.68000	35.0	Cluster_04765
885.95000	35.0	Cluster_39078
886.43000	35.0	Cluster_39095
886.96000	35.0	Cluster_39099
887.52000	35.0	Cluster_39107
887.68000	35.0	Cluster_04781
887.74000	35.0	Cluster_14571
888.13000	35.0	Cluster_14577
888.40000	35.0	Cluster_14578
888.91000	35.0	Cluster_39135
888.92000	35.0	Cluster_04786
889.11000	35.0	Cluster_14581
889.44000	35.0	Cluster_39147
890.17000	35.0	Cluster_04796
890.38000	35.0	Cluster_39161
890.39000	35.0	Cluster_39160
890.46000	35.0	Cluster_39173
890.99000	35.0	Cluster_39180
891.46000	35.0	Cluster_39186
891.81000	35.0	Cluster_14598
892.21000	35.0	Cluster_04824
893.43000	35.0	Cluster_39231
893.78000	35.0	Cluster_14615
894.12000	35.0	Cluster_14617
894.71000	35.0	Cluster_04845
895.21000	35.0	Cluster_04851
895.45000	35.0	Cluster_14622
895.81000	35.0	Cluster_14625
896.15000	35.0	Cluster_14634
897.04000	35.0	Cluster_39292
898.77000	35.0	Cluster_14664
899.43000	35.0	Cluster_39337
899.51000	35.0	Cluster_39345
899.96000	35.0	Cluster_04891
900.94000	35.0	Cluster_39368
900.97000	35.0	Cluster_39380

900.99000	35.0	Cluster_39377
902.46000	35.0	Cluster_39412
904.46000	35.0	Cluster_39443
905.43000	35.0	Cluster_39461
906.77000	35.0	Cluster_14739
907.80000	35.0	Cluster_14749
908.46000	35.0	Cluster_39500
908.48000	35.0	Cluster_14756
908.94000	35.0	Cluster_04958
908.95000	35.0	Cluster_39504
909.44000	35.0	Cluster_14760
909.50000	35.0	Cluster_14765
909.89000	35.0	Cluster_39520
910.11000	35.0	Cluster_14769
910.19000	35.0	Cluster_04966
911.96000	35.0	Cluster_39564
914.56000	35.0	Cluster_39615
915.13000	35.0	Cluster_14823
915.93000	35.0	Cluster_39626
916.44000	35.0	Cluster_39640
916.49000	35.0	Cluster_39633
917.45000	35.0	Cluster_14854
917.97000	35.0	Cluster_39674
918.66000	35.0	Cluster_39698
920.45000	35.0	Cluster_39721
920.76000	35.0	Cluster_14891
920.79000	35.0	Cluster_39725
920.88000	35.0	Cluster_39726
920.95000	35.0	Cluster_39731
922.42000	35.0	Cluster_14907
922.98000	35.0	Cluster_39772
923.30000	35.0	Cluster_14924
923.45000	35.0	Cluster_14932
923.92000	35.0	Cluster_39782
924.49000	35.0	Cluster_39788
925.11000	35.0	Cluster_14961
926.42000	35.0	Cluster_14975
927.48000	35.0	Cluster_39834
928.47000	35.0	Cluster_39858
928.96000	35.0	Cluster_39861
929.12000	35.0	Cluster_15009
929.43000	35.0	Cluster_39880
929.90000	35.0	Cluster_39897
930.47000	35.0	Cluster_15020
931.73000	35.0	Cluster_15043
931.90000	35.0	Cluster_39924
932.64000	35.0	Cluster_15062
932.95000	35.0	Cluster_39941
933.14000	35.0	Cluster_15072
933.33000	35.0	Cluster_05038
934.00000	35.0	Cluster_39963

934.13000	35.0	Cluster_15084
934.43000	35.0	Cluster_15083
934.78000	35.0	Cluster_15089
935.78000	35.0	Cluster_15100
936.45000	35.0	Cluster_15116
936.80000	35.0	Cluster_15129
937.00000	35.0	Cluster_39997
937.44000	35.0	Cluster_15144
937.49000	35.0	Cluster_05051
938.07000	35.0	Cluster_15154
938.13000	35.0	Cluster_15156
938.21000	35.0	Cluster_15160
939.69000	35.0	Cluster_05058
940.46000	35.0	Cluster_15180
941.72000	35.0	Cluster_40098
942.00000	35.0	Cluster_40094
943.45000	35.0	Cluster_15229
945.13000	35.0	Cluster_15244
945.39000	35.0	Cluster_15242
945.72000	35.0	Cluster_05075
945.73000	35.0	Cluster_05078
945.78000	35.0	Cluster_15250
945.98000	35.0	Cluster_40152
947.16000	35.0	Cluster_15264
947.22000	35.0	Cluster_05084
947.79000	35.0	Cluster_15273
948.19000	35.0	Cluster_15277
948.52000	35.0	Cluster_40180
949.14000	35.0	Cluster_15301
949.44000	35.0	Cluster_40190
949.46000	35.0	Cluster_15302
950.44000	35.0	Cluster_15314
951.17000	35.0	Cluster_15324
951.96000	35.0	Cluster_40227
952.14000	35.0	Cluster_15337
952.15000	35.0	Cluster_15339
952.44000	35.0	Cluster_15342
952.53000	35.0	Cluster_40233
953.98000	35.0	Cluster_40255
954.82000	35.0	Cluster_15379
954.99000	35.0	Cluster_40262
955.13000	35.0	Cluster_15394
955.47000	35.0	Cluster_15404
956.48000	35.0	Cluster_15426
957.07000	35.0	Cluster_40290
957.48000	35.0	Cluster_40291
960.15000	35.0	Cluster_15462
960.98000	35.0	Cluster_40317
961.48000	35.0	Cluster_15478
962.14000	35.0	Cluster_15497
962.38000	35.0	Cluster_40344

962.79000	35.0	Cluster_15505
964.13000	35.0	Cluster_15523
964.43000	35.0	Cluster_40365
965.29000	35.0	Cluster_40377
966.15000	35.0	Cluster_15549
966.49000	35.0	Cluster_40387
966.50000	35.0	Cluster_05172
966.85000	35.0	Cluster_15552
967.00000	35.0	Cluster_40392
967.56000	35.0	Cluster_15565
968.03000	35.0	Cluster_40402
968.25000	35.0	Cluster_05187
971.97000	35.0	Cluster_40451
972.01000	35.0	Cluster_40467
972.46000	35.0	Cluster_15627
972.49000	35.0	Cluster_15626
973.51000	35.0	Cluster_40476
974.47000	35.0	Cluster_15651
974.98000	35.0	Cluster_40495
975.82000	35.0	Cluster_15672
975.98000	35.0	Cluster_05210
976.00000	35.0	Cluster_05212
976.03000	35.0	Cluster_40504
977.14000	35.0	Cluster_15696
977.62000	35.0	Cluster_40522
979.45000	35.0	Cluster_40546
979.80000	35.0	Cluster_15720
979.83000	35.0	Cluster_15721
979.98000	35.0	Cluster_40568
980.00000	35.0	Cluster_40562
980.50000	35.0	Cluster_40565
980.70000	35.0	Cluster_40578
981.45000	35.0	Cluster_15750
983.25000	35.0	Cluster_05237
983.95000	35.0	Cluster_40617
985.14000	35.0	Cluster_40628
986.02000	35.0	Cluster_40630
986.52000	35.0	Cluster_40639
986.83000	35.0	Cluster_15810
987.24000	35.0	Cluster_40654
987.80000	35.0	Cluster_15825
988.44000	35.0	Cluster_15835
991.93000	35.0	Cluster_40717
993.50000	35.0	Cluster_15895
997.19000	35.0	Cluster_15954
1000.06000	35.0	Cluster_40851
1001.03000	35.0	Cluster_40878
1001.16000	35.0	Cluster_15996
1001.49000	35.0	Cluster_40884
1003.52000	35.0	Cluster_05294
1004.00000	35.0	Cluster_40937

1004.31000	35.0	Cluster_05296
1004.57000	35.0	Cluster_40940
1007.81000	35.0	Cluster_16072
1008.78000	35.0	Cluster_16080
1012.26000	35.0	Cluster_05329
1012.27000	35.0	Cluster_41044
1012.93000	35.0	Cluster_41054
1013.01000	35.0	Cluster_05341
1013.47000	35.0	Cluster_16138
1013.49000	35.0	Cluster_16137
1013.76000	35.0	Cluster_05344
1013.90000	35.0	Cluster_41056
1015.92000	35.0	Cluster_41088
1016.78000	35.0	Cluster_05351
1017.27000	35.0	Cluster_41099
1019.25000	35.0	Cluster_05360
1020.15000	35.0	Cluster_16210
1022.50000	35.0	Cluster_16231
1023.82000	35.0	Cluster_41185
1024.48000	35.0	Cluster_16254
1024.50000	35.0	Cluster_16256
1024.93000	35.0	Cluster_41195
1025.46000	35.0	Cluster_16264
1025.81000	35.0	Cluster_41211
1026.83000	35.0	Cluster_16274
1027.13000	35.0	Cluster_16285
1027.14000	35.0	Cluster_16284
1027.88000	35.0	Cluster_16291
1028.18000	35.0	Cluster_16295
1028.84000	35.0	Cluster_16300
1029.99000	35.0	Cluster_41262
1031.20000	35.0	Cluster_16323
1032.82000	35.0	Cluster_16335
1033.20000	35.0	Cluster_16342
1036.52000	35.0	Cluster_16382
1037.50000	35.0	Cluster_16400
1039.05000	35.0	Cluster_41371
1039.52000	35.0	Cluster_41374
1039.81000	35.0	Cluster_16429
1040.83000	35.0	Cluster_05437
1041.01000	35.0	Cluster_41401
1042.17000	35.0	Cluster_16457
1044.49000	35.0	Cluster_41432
1046.13000	35.0	Cluster_16488
1046.22000	35.0	Cluster_16495
1047.16000	35.0	Cluster_16507
1047.83000	35.0	Cluster_41470
1053.50000	35.0	Cluster_16581
1053.98000	35.0	Cluster_41534
1054.99000	35.0	Cluster_05476
1055.22000	35.0	Cluster_16595

1055.50000	35.0	Cluster_16606
1056.52000	35.0	Cluster_16616
1057.06000	35.0	Cluster_41567
1057.33000	35.0	Cluster_05486
1057.34000	35.0	Cluster_05487
1057.58000	35.0	Cluster_41574
1057.99000	35.0	Cluster_41578
1058.02000	35.0	Cluster_41582
1058.81000	35.0	Cluster_16658
1058.85000	35.0	Cluster_16649
1058.96000	35.0	Cluster_41595
1058.97000	35.0	Cluster_41597
1059.01000	35.0	Cluster_41596
1060.32000	35.0	Cluster_41612
1060.75000	35.0	Cluster_05502
1060.82000	35.0	Cluster_16679
1061.26000	35.0	Cluster_05510
1061.33000	35.0	Cluster_05506
1061.82000	35.0	Cluster_41625
1062.83000	35.0	Cluster_16700
1063.27000	35.0	Cluster_05516
1063.82000	35.0	Cluster_41655
1063.99000	35.0	Cluster_41663
1064.72000	35.0	Cluster_05521
1064.73000	35.0	Cluster_41675
1064.99000	35.0	Cluster_41673
1066.26000	35.0	Cluster_41702
1067.52000	35.0	Cluster_41715
1068.05000	35.0	Cluster_41719
1068.75000	35.0	Cluster_41734
1069.51000	35.0	Cluster_41748
1070.16000	35.0	Cluster_16781
1070.51000	35.0	Cluster_05546
1072.55000	35.0	Cluster_16804
1072.76000	35.0	Cluster_05555
1074.19000	35.0	Cluster_16831
1074.51000	35.0	Cluster_41810
1075.53000	35.0	Cluster_41816
1076.75000	35.0	Cluster_05574
1078.01000	35.0	Cluster_05578
1079.49000	35.0	Cluster_05582
1080.75000	35.0	Cluster_00593
1082.00000	35.0	Cluster_05588
1083.50000	35.0	Cluster_05592
1086.59000	35.0	Cluster_41940
1091.58000	35.0	Cluster_41998
1092.90000	35.0	Cluster_17025
1093.21000	35.0	Cluster_17031
1093.55000	35.0	Cluster_17035
1093.58000	35.0	Cluster_42024
1093.99000	35.0	Cluster_42029

1094.92000	35.0	Cluster_00625
1096.55000	35.0	Cluster_42051
1100.23000	35.0	Cluster_17091
1100.56000	35.0	Cluster_17097
1101.96000	35.0	Cluster_17105
1102.54000	35.0	Cluster_17111
1103.12000	35.0	Cluster_42104
1108.23000	35.0	Cluster_17176
1108.37000	35.0	Cluster_42148
1108.88000	35.0	Cluster_17175
1109.86000	35.0	Cluster_17199
1113.50000	35.0	Cluster_42184
1113.53000	35.0	Cluster_17241
1115.18000	35.0	Cluster_17258
1115.77000	35.0	Cluster_00660
1117.22000	35.0	Cluster_17276
1118.21000	35.0	Cluster_17277
1118.22000	35.0	Cluster_17280
1119.87000	35.0	Cluster_42234
1121.54000	35.0	Cluster_17322
1123.04000	35.0	Cluster_42270
1123.97000	35.0	Cluster_42271
1124.25000	35.0	Cluster_17359
1125.57000	35.0	Cluster_42283
1126.07000	35.0	Cluster_42291
1127.88000	35.0	Cluster_17398
1128.57000	35.0	Cluster_17409
1129.21000	35.0	Cluster_17418
1129.55000	35.0	Cluster_17428
1129.90000	35.0	Cluster_17433
1130.23000	35.0	Cluster_17434
1131.58000	35.0	Cluster_17448
1133.13000	35.0	Cluster_42337
1133.20000	35.0	Cluster_17479
1133.22000	35.0	Cluster_17484
1134.90000	35.0	Cluster_17503
1135.23000	35.0	Cluster_17509
1135.51000	35.0	Cluster_17513
1135.54000	35.0	Cluster_17519
1135.55000	35.0	Cluster_17517
1136.11000	35.0	Cluster_42363
1136.88000	35.0	Cluster_17525
1136.89000	35.0	Cluster_17538
1138.13000	35.0	Cluster_42377
1140.22000	35.0	Cluster_17567
1143.18000	35.0	Cluster_00674
1143.56000	35.0	Cluster_17607
1143.60000	35.0	Cluster_42409
1144.20000	35.0	Cluster_17626
1144.24000	35.0	Cluster_17620
1145.21000	35.0	Cluster_17645

1146.57000	35.0	Cluster_00680
1149.29000	35.0	Cluster_42461
1151.05000	35.0	Cluster_42474
1152.54000	35.0	Cluster_17735
1152.88000	35.0	Cluster_17750
1153.03000	35.0	Cluster_05790
1153.76000	35.0	Cluster_00695
1154.35000	35.0	Cluster_42485
1155.34000	35.0	Cluster_42501
1156.84000	35.0	Cluster_42511
1158.84000	35.0	Cluster_42519
1159.61000	35.0	Cluster_42529
1160.34000	35.0	Cluster_42534
1162.11000	35.0	Cluster_42551
1162.64000	35.0	Cluster_42555
1163.09000	35.0	Cluster_05817
1166.08000	35.0	Cluster_05827
1166.14000	35.0	Cluster_42592
1166.21000	35.0	Cluster_17894
1167.19000	35.0	Cluster_17908
1167.57000	35.0	Cluster_17921
1168.21000	35.0	Cluster_17931
1168.33000	35.0	Cluster_42608
1168.58000	35.0	Cluster_05832
1173.07000	35.0	Cluster_42645
1180.80000	35.0	Cluster_05888
1183.23000	35.0	Cluster_18040
1187.59000	35.0	Cluster_18075
1190.56000	35.0	Cluster_18091
1192.81000	35.0	Cluster_42765
1192.92000	35.0	Cluster_18117
1195.88000	35.0	Cluster_18133
1196.63000	35.0	Cluster_42787
1197.59000	35.0	Cluster_18145
1197.90000	35.0	Cluster_18140
1198.64000	35.0	Cluster_42803
1199.32000	35.0	Cluster_05982
1199.57000	35.0	Cluster_18161
1200.26000	35.0	Cluster_18168
1200.56000	35.0	Cluster_18177
1201.89000	35.0	Cluster_18192
1203.44000	35.0	Cluster_42830
1206.62000	35.0	Cluster_42853
1208.56000	35.0	Cluster_18237
1209.51000	35.0	Cluster_42887
1213.12000	35.0	Cluster_42910
1215.15000	35.0	Cluster_42924
1218.11000	35.0	Cluster_42950
1219.09000	35.0	Cluster_42956
1221.25000	35.0	Cluster_18299
1222.10000	35.0	Cluster_42968

1224.08000	35.0	Cluster_42977
1224.60000	35.0	Cluster_42983
1225.59000	35.0	Cluster_42994
1228.07000	35.0	Cluster_43008
1228.11000	35.0	Cluster_43007
1230.63000	35.0	Cluster_43026
1232.57000	35.0	Cluster_43045
1234.58000	35.0	Cluster_43065
1238.57000	35.0	Cluster_43079
1245.28000	35.0	Cluster_18390
1249.16000	35.0	Cluster_43135
1251.87000	35.0	Cluster_43149
1253.87000	35.0	Cluster_43162
1255.86000	35.0	Cluster_43176
1257.08000	35.0	Cluster_06282
1264.27000	35.0	Cluster_18463
1267.20000	35.0	Cluster_43224
1267.68000	35.0	Cluster_43228
1274.29000	35.0	Cluster_18502
1284.15000	35.0	Cluster_43287
1300.69000	35.0	Cluster_43366
1300.70000	35.0	Cluster_43360
1300.73000	35.0	Cluster_43361
1300.89000	35.0	Cluster_43362
1301.68000	35.0	Cluster_43365
1320.21000	35.0	Cluster_43449
1329.73000	35.0	Cluster_43484
1330.45000	35.0	Cluster_06499
1331.69000	35.0	Cluster_43492
1334.68000	35.0	Cluster_43505
1349.89000	35.0	Cluster_43562
1353.71000	35.0	Cluster_43578
1354.21000	35.0	Cluster_43579
1354.66000	35.0	Cluster_06571
1361.03000	35.0	Cluster_18681
1367.16000	35.0	Cluster_43645
1372.75000	35.0	Cluster_43667
1376.18000	35.0	Cluster_43680
1376.34000	35.0	Cluster_18707
1383.67000	35.0	Cluster_18716
1394.65000	35.0	Cluster_43739
1396.44000	35.0	Cluster_18732
1402.12000	35.0	Cluster_18741
1406.24000	35.0	Cluster_43793
1407.03000	35.0	Cluster_18752
1414.35000	35.0	Cluster_18768
1417.37000	35.0	Cluster_18772
1420.01000	35.0	Cluster_18783
1422.96000	35.0	Cluster_43843
1423.47000	35.0	Cluster_43844
1424.69000	35.0	Cluster_18789

1424.79000	35.0	Cluster_43851
1425.27000	35.0	Cluster_43853
1427.03000	35.0	Cluster_18794
1428.82000	35.0	Cluster_43862
1429.02000	35.0	Cluster_18795
1430.01000	35.0	Cluster_18796
1431.68000	35.0	Cluster_18799
1437.03000	35.0	Cluster_18803
1439.35000	35.0	Cluster_18807
1459.27000	35.0	Cluster_43959
1477.30000	35.0	Cluster_44015
1489.22000	35.0	Cluster_44043
1492.94000	35.0	Cluster_44054
1495.14000	35.0	Cluster_44065
1511.80000	35.0	Cluster_44102
1518.72000	35.0	Cluster_44112
1530.33000	35.0	Cluster_44125
1560.76000	35.0	Cluster_44175
1564.32000	35.0	Cluster_44184
1571.76000	35.0	Cluster_44194
1582.85000	35.0	Cluster_44218
1616.85000	35.0	Cluster_44250
1633.97000	35.0	Cluster_44257
1635.40000	35.0	Cluster_44260
1676.82000	35.0	Cluster_44320
1683.84000	35.0	Cluster_44341
1687.92000	35.0	Cluster_44353
1739.94000	35.0	Cluster_44403
1792.99000	35.0	Cluster_44425

Reject Mass List: (none)
Neutral Loss Mass List: (none)
Product Mass List: (none)
Neutral loss in top: 3
Product in top: 3
Most intense if no parent masses found enabled
Add/subtract mass not enabled
FT master scan preview mode enabled
Charge state screening enabled
Charge state dependent ETD time not enabled
Monoisotopic precursor selection enabled
Charge state rejection enabled
Unassigned charge states : rejected
Charge state 1 : rejected
Charge state 2 : not rejected
Charge state 3 : not rejected
Charge states 4+ : not rejected
Chromatography mode is disabled

Global Data Dependent Settings:
Predict ion injection time enabled
Use global parent and reject mass lists not enabled

Exclude parent mass from data dependent selection not enabled
Exclusion mass width by mass
Exclusion mass width low: 0.50000
Exclusion mass width high: 0.50000
Parent mass width by mass
Parent mass width low: 0.50000
Parent mass width high: 0.50000
Reject mass width by mass
Reject mass width low: 0.50000
Reject mass width high: 0.50000
Zoom/UltraZoom scan mass width by mass
Zoom/UltraZoom scan mass width low: 5.00
Zoom/UltraZoom scan mass width high: 5.00
FT SIM scan mass width low: 5.00
FT SIM scan mass width high: 5.00
Neutral Loss candidates processed by decreasing intensity
Neutral Loss mass width by mass
Neutral Loss mass width low: 0.50000
Neutral Loss mass width high: 0.50000
Product candidates processed by decreasing intensity
Product mass width by mass
Product mass width low: 0.50000
Product mass width high: 0.50000
MS mass range: 0.00-1000000.00
MSn mass range by mass
MSn mass range: 0.00-1000000.00
Use m/z values as masses not enabled
Analog UV data dep. not enabled
Dynamic exclusion not enabled
Isotopic data dependence not enabled
Mass Tags data dependence not enabled

Custom Data Dependent Settings:
Not enabled

Pass 10 (wheat-mixed-digests_MS2_TO-USE-inclusion-01_3.raw):

Creator: Orbi_30393
Last modified: 10/19/2021 by Orbi_30393
MS Run Time (min): 43.00
Sequence override of method parameters not enabled.
Divert Valve: not used during run
Contact Closure: not used during run
Syringe Pump: not used during run
MS Detector Settings:
Real-time modifications to method not enabled
Stepped collision energy not enabled
Additional Microscans:

MS2	0	0
MS3	0	0
MS4	0	0
MS5	0	0

MS6 0 0
MS7 0 0
MS8 0 0
MS9 0 0
MS10 0 0
Segment 1 Information
Duration (min): 43.00
Number of Scan Events: 2
Tune Method: Orbitrap-tune-file_2020-03-13_HESI

Scan Event Details:
1: FTMS + p norm res=15000 o(300.0-2000.0)
CV = 0.0V
2: ITMS + c norm Dep MS/MS Most intense ion from (1)
Activation Type: CID
Min. Signal Required: 500.0
Isolation Width: 0.50
Normalized Coll. Energy: 35.0
Default Charge State: 2
Activation Q: 0.250
Activation Time: 10.000
CV = 0.0V

Lock Masses:
Pos List Name: N/A
Source: API Source
Mass List: (none)
Neg List Name: N/A
Source: API Source
Mass List: (none)

Data Dependent Settings:
Use separate polarity settings disabled
Parent Mass List:

MS Mass	MS FAIMS CV	MS Normalized Collision Energy	MS2 Mass	MS2 Normalized Collision Energy	Name
300.13000		35.0			Cluster_06844
300.14000		35.0			Cluster_18928
300.71000		35.0			Cluster_18955
301.50000		35.0			Cluster_06851
301.65000		35.0			Cluster_18984
302.64000		35.0			Cluster_19016
302.65000		35.0			Cluster_19017
303.99000		35.0			Cluster_19040
304.11000		35.0			Cluster_19042
304.14000		35.0			Cluster_19049
304.16000		35.0			Cluster_19050
304.20000		35.0			Cluster_19052
304.49000		35.0			Cluster_06863

304.64000	35.0	Cluster_19062
306.13000	35.0	Cluster_06873
306.67000	35.0	Cluster_19109
307.12000	35.0	Cluster_19119
307.13000	35.0	Cluster_06880
308.65000	35.0	Cluster_19177
308.66000	35.0	Cluster_19179
309.53000	35.0	Cluster_06901
310.58000	35.0	Cluster_19217
310.65000	35.0	Cluster_19222
310.66000	35.0	Cluster_19221
311.17000	35.0	Cluster_19246
311.68000	35.0	Cluster_19257
313.65000	35.0	Cluster_19315
314.82000	35.0	Cluster_06941
315.65000	35.0	Cluster_19384
316.69000	35.0	Cluster_19442
317.06000	35.0	Cluster_19459
317.16000	35.0	Cluster_19466
317.64000	35.0	Cluster_19490
318.64000	35.0	Cluster_19532
318.85000	35.0	Cluster_06973
319.17000	35.0	Cluster_19552
319.67000	35.0	Cluster_19560
319.94000	35.0	Cluster_00850
320.49000	35.0	Cluster_06993
320.80000	35.0	Cluster_06994
321.15000	35.0	Cluster_06995
321.17000	35.0	Cluster_00869
321.52000	35.0	Cluster_19594
322.15000	35.0	Cluster_19619
322.17000	35.0	Cluster_19617
322.50000	35.0	Cluster_06998
323.65000	35.0	Cluster_19672
323.69000	35.0	Cluster_19677
323.83000	35.0	Cluster_19683
324.42000	35.0	Cluster_00878
324.60000	35.0	Cluster_19711
324.65000	35.0	Cluster_19713
324.83000	35.0	Cluster_07017
325.14000	35.0	Cluster_19730
325.82000	35.0	Cluster_07023
325.85000	35.0	Cluster_07028
326.17000	35.0	Cluster_19753
326.40000	35.0	Cluster_00887
326.90000	35.0	Cluster_00888
327.21000	35.0	Cluster_19791
327.96000	35.0	Cluster_07043
328.48000	35.0	Cluster_07051
329.18000	35.0	Cluster_19855
329.82000	35.0	Cluster_07063

329.93000	35.0	Cluster_19873
330.18000	35.0	Cluster_19886
330.90000	35.0	Cluster_19916
331.17000	35.0	Cluster_19926
331.20000	35.0	Cluster_19928
331.21000	35.0	Cluster_19930
331.22000	35.0	Cluster_19925
331.66000	35.0	Cluster_19936
332.14000	35.0	Cluster_19948
333.47000	35.0	Cluster_07081
333.82000	35.0	Cluster_07087
333.87000	35.0	Cluster_07090
335.12000	35.0	Cluster_20034
335.83000	35.0	Cluster_07104
336.19000	35.0	Cluster_07109
336.23000	35.0	Cluster_20093
336.91000	35.0	Cluster_00920
337.19000	35.0	Cluster_20132
337.21000	35.0	Cluster_20126
337.83000	35.0	Cluster_07126
338.11000	35.0	Cluster_20150
338.67000	35.0	Cluster_20172
339.16000	35.0	Cluster_20192
339.47000	35.0	Cluster_07139
339.81000	35.0	Cluster_07150
339.94000	35.0	Cluster_00929
340.18000	35.0	Cluster_20223
340.26000	35.0	Cluster_20229
341.15000	35.0	Cluster_20257
341.16000	35.0	Cluster_20261
342.16000	35.0	Cluster_07167
343.11000	35.0	Cluster_20316
343.18000	35.0	Cluster_20318
343.66000	35.0	Cluster_20344
345.19000	35.0	Cluster_20390
345.67000	35.0	Cluster_20402
346.15000	35.0	Cluster_07199
346.67000	35.0	Cluster_20444
347.20000	35.0	Cluster_20457
347.71000	35.0	Cluster_20471
348.44000	35.0	Cluster_20495
348.87000	35.0	Cluster_07234
348.92000	35.0	Cluster_00975
348.93000	35.0	Cluster_00976
349.20000	35.0	Cluster_20517
349.21000	35.0	Cluster_20520
349.64000	35.0	Cluster_20543
349.73000	35.0	Cluster_20549
350.42000	35.0	Cluster_00982
351.18000	35.0	Cluster_20611
351.19000	35.0	Cluster_20622

352.66000	35.0	Cluster_20669
353.17000	35.0	Cluster_01002
353.21000	35.0	Cluster_20705
353.66000	35.0	Cluster_01003
353.80000	35.0	Cluster_07278
353.87000	35.0	Cluster_20720
353.91000	35.0	Cluster_01004
354.16000	35.0	Cluster_20732
354.20000	35.0	Cluster_20739
354.40000	35.0	Cluster_01009
354.42000	35.0	Cluster_01011
355.14000	35.0	Cluster_20781
355.16000	35.0	Cluster_20782
355.22000	35.0	Cluster_20787
355.36000	35.0	Cluster_20789
355.70000	35.0	Cluster_20796
356.24000	35.0	Cluster_20824
356.55000	35.0	Cluster_07307
358.42000	35.0	Cluster_20933
359.13000	35.0	Cluster_07326
359.17000	35.0	Cluster_00118
359.37000	35.0	Cluster_20960
360.16000	35.0	Cluster_20986
360.42000	35.0	Cluster_01047
360.83000	35.0	Cluster_21008
361.18000	35.0	Cluster_21024
361.42000	35.0	Cluster_01055
361.66000	35.0	Cluster_21038
361.67000	35.0	Cluster_21036
361.69000	35.0	Cluster_21039
361.84000	35.0	Cluster_07347
362.10000	35.0	Cluster_21048
362.42000	35.0	Cluster_01059
362.43000	35.0	Cluster_21075
362.60000	35.0	Cluster_21077
362.65000	35.0	Cluster_21076
363.11000	35.0	Cluster_21089
363.19000	35.0	Cluster_21093
363.20000	35.0	Cluster_21094
363.77000	35.0	Cluster_21121
364.16000	35.0	Cluster_21135
364.50000	35.0	Cluster_21161
364.65000	35.0	Cluster_21162
365.24000	35.0	Cluster_21221
365.79000	35.0	Cluster_07396
365.90000	35.0	Cluster_21241
366.27000	35.0	Cluster_21267
366.74000	35.0	Cluster_21285
367.22000	35.0	Cluster_21306
367.50000	35.0	Cluster_07416
367.53000	35.0	Cluster_21324

368.16000	35.0	Cluster_21368
368.20000	35.0	Cluster_21366
368.47000	35.0	Cluster_07428
369.67000	35.0	Cluster_21437
370.08000	35.0	Cluster_21453
370.50000	35.0	Cluster_07445
370.74000	35.0	Cluster_21476
370.93000	35.0	Cluster_21486
371.18000	35.0	Cluster_07449
371.24000	35.0	Cluster_21501
371.71000	35.0	Cluster_21517
371.89000	35.0	Cluster_21531
372.17000	35.0	Cluster_21537
372.23000	35.0	Cluster_07464
372.67000	35.0	Cluster_21575
372.69000	35.0	Cluster_21572
373.12000	35.0	Cluster_21589
373.14000	35.0	Cluster_21585
373.16000	35.0	Cluster_07470
373.19000	35.0	Cluster_07471
373.22000	35.0	Cluster_21597
373.90000	35.0	Cluster_21628
374.19000	35.0	Cluster_07480
374.40000	35.0	Cluster_21650
374.68000	35.0	Cluster_21656
375.15000	35.0	Cluster_21668
375.89000	35.0	Cluster_07496
377.48000	35.0	Cluster_07512
377.77000	35.0	Cluster_21780
378.17000	35.0	Cluster_01122
378.18000	35.0	Cluster_21788
378.20000	35.0	Cluster_07523
378.67000	35.0	Cluster_01125
379.26000	35.0	Cluster_21841
380.45000	35.0	Cluster_21886
380.68000	35.0	Cluster_21891
381.22000	35.0	Cluster_07550
381.70000	35.0	Cluster_01141
382.18000	35.0	Cluster_21953
382.66000	35.0	Cluster_01144
383.63000	35.0	Cluster_22024
383.68000	35.0	Cluster_22025
384.16000	35.0	Cluster_01146
384.21000	35.0	Cluster_22055
384.22000	35.0	Cluster_22065
385.52000	35.0	Cluster_07597
385.54000	35.0	Cluster_07596
385.86000	35.0	Cluster_22123
386.66000	35.0	Cluster_22155
386.68000	35.0	Cluster_22158
386.90000	35.0	Cluster_22172

387.11000	35.0	Cluster_22177
387.63000	35.0	Cluster_22217
387.93000	35.0	Cluster_22226
388.23000	35.0	Cluster_22233
388.50000	35.0	Cluster_07621
388.64000	35.0	Cluster_22244
388.67000	35.0	Cluster_22245
388.83000	35.0	Cluster_07623
389.25000	35.0	Cluster_22269
389.51000	35.0	Cluster_07627
389.91000	35.0	Cluster_22284
390.71000	35.0	Cluster_22316
390.87000	35.0	Cluster_07651
390.88000	35.0	Cluster_07646
391.50000	35.0	Cluster_07654
391.65000	35.0	Cluster_22334
391.75000	35.0	Cluster_22344
391.82000	35.0	Cluster_07658
392.22000	35.0	Cluster_07665
392.25000	35.0	Cluster_22358
392.73000	35.0	Cluster_22381
392.77000	35.0	Cluster_22377
393.19000	35.0	Cluster_01195
393.53000	35.0	Cluster_07674
393.69000	35.0	Cluster_01197
393.71000	35.0	Cluster_22428
393.98000	35.0	Cluster_22479
394.21000	35.0	Cluster_22490
394.71000	35.0	Cluster_22522
394.72000	35.0	Cluster_22521
395.89000	35.0	Cluster_07703
396.23000	35.0	Cluster_22589
396.30000	35.0	Cluster_22595
397.39000	35.0	Cluster_22628
397.67000	35.0	Cluster_22634
397.72000	35.0	Cluster_22641
397.90000	35.0	Cluster_07736
398.18000	35.0	Cluster_01226
398.44000	35.0	Cluster_01230
400.24000	35.0	Cluster_22747
400.68000	35.0	Cluster_22780
400.90000	35.0	Cluster_07771
401.70000	35.0	Cluster_22833
402.32000	35.0	Cluster_22866
402.48000	35.0	Cluster_22868
402.72000	35.0	Cluster_22878
403.44000	35.0	Cluster_01260
403.54000	35.0	Cluster_07797
404.47000	35.0	Cluster_01272
405.88000	35.0	Cluster_07831
406.22000	35.0	Cluster_23023

406.68000	35.0	Cluster_01285
406.73000	35.0	Cluster_23046
406.95000	35.0	Cluster_01293
407.17000	35.0	Cluster_23059
407.25000	35.0	Cluster_23066
407.67000	35.0	Cluster_01302
407.75000	35.0	Cluster_23083
407.93000	35.0	Cluster_01303
408.22000	35.0	Cluster_23104
408.54000	35.0	Cluster_07863
408.66000	35.0	Cluster_01305
408.91000	35.0	Cluster_07864
408.92000	35.0	Cluster_01307
409.19000	35.0	Cluster_07867
409.25000	35.0	Cluster_23160
409.69000	35.0	Cluster_23170
410.53000	35.0	Cluster_07882
411.72000	35.0	Cluster_23258
412.67000	35.0	Cluster_23299
412.68000	35.0	Cluster_01326
413.23000	35.0	Cluster_23320
413.87000	35.0	Cluster_07913
413.92000	35.0	Cluster_07916
413.94000	35.0	Cluster_23350
413.98000	35.0	Cluster_23354
414.71000	35.0	Cluster_23379
414.85000	35.0	Cluster_23397
414.87000	35.0	Cluster_07924
415.22000	35.0	Cluster_23422
415.73000	35.0	Cluster_23434
416.00000	35.0	Cluster_23448
416.22000	35.0	Cluster_23463
416.76000	35.0	Cluster_23476
416.88000	35.0	Cluster_23482
416.94000	35.0	Cluster_01353
418.25000	35.0	Cluster_23534
418.47000	35.0	Cluster_23549
419.18000	35.0	Cluster_01360
419.21000	35.0	Cluster_07978
419.88000	35.0	Cluster_23600
420.19000	35.0	Cluster_23617
420.23000	35.0	Cluster_23612
420.71000	35.0	Cluster_23632
421.56000	35.0	Cluster_08005
422.12000	35.0	Cluster_08008
422.55000	35.0	Cluster_08014
422.58000	35.0	Cluster_08015
423.14000	35.0	Cluster_23727
423.21000	35.0	Cluster_08022
423.24000	35.0	Cluster_23740
423.44000	35.0	Cluster_01387

423.71000	35.0	Cluster_23753
423.74000	35.0	Cluster_23766
424.18000	35.0	Cluster_23787
424.51000	35.0	Cluster_23811
424.68000	35.0	Cluster_23813
425.18000	35.0	Cluster_23850
425.46000	35.0	Cluster_23865
425.87000	35.0	Cluster_08056
425.88000	35.0	Cluster_23895
426.14000	35.0	Cluster_23901
426.24000	35.0	Cluster_23924
426.44000	35.0	Cluster_23942
426.88000	35.0	Cluster_08077
427.51000	35.0	Cluster_08094
428.56000	35.0	Cluster_08107
428.72000	35.0	Cluster_24045
428.79000	35.0	Cluster_24059
429.24000	35.0	Cluster_24074
429.57000	35.0	Cluster_08120
429.72000	35.0	Cluster_24100
429.76000	35.0	Cluster_24093
429.85000	35.0	Cluster_08124
429.95000	35.0	Cluster_01432
430.73000	35.0	Cluster_24136
431.21000	35.0	Cluster_24172
431.41000	35.0	Cluster_24179
431.74000	35.0	Cluster_24194
432.16000	35.0	Cluster_24207
432.21000	35.0	Cluster_24215
432.87000	35.0	Cluster_08164
433.96000	35.0	Cluster_24289
434.79000	35.0	Cluster_24347
434.97000	35.0	Cluster_24350
435.17000	35.0	Cluster_24357
435.24000	35.0	Cluster_08201
435.37000	35.0	Cluster_24383
435.70000	35.0	Cluster_01481
435.97000	35.0	Cluster_01487
436.48000	35.0	Cluster_01491
437.37000	35.0	Cluster_24491
437.46000	35.0	Cluster_01497
438.22000	35.0	Cluster_24532
438.30000	35.0	Cluster_24543
438.96000	35.0	Cluster_01503
438.97000	35.0	Cluster_01504
439.82000	35.0	Cluster_24632
440.21000	35.0	Cluster_01512
440.56000	35.0	Cluster_24655
440.74000	35.0	Cluster_24659
440.86000	35.0	Cluster_08277
441.21000	35.0	Cluster_24682

441.24000	35.0	Cluster_01516
441.73000	35.0	Cluster_24703
441.87000	35.0	Cluster_08293
442.00000	35.0	Cluster_24716
442.28000	35.0	Cluster_24730
442.57000	35.0	Cluster_08303
442.77000	35.0	Cluster_24758
442.89000	35.0	Cluster_24763
442.98000	35.0	Cluster_24761
443.12000	35.0	Cluster_24771
443.22000	35.0	Cluster_24782
443.28000	35.0	Cluster_24785
443.29000	35.0	Cluster_24795
443.89000	35.0	Cluster_08322
443.92000	35.0	Cluster_08324
444.72000	35.0	Cluster_24866
445.18000	35.0	Cluster_08343
445.55000	35.0	Cluster_24900
446.27000	35.0	Cluster_24941
446.73000	35.0	Cluster_24959
446.91000	35.0	Cluster_08369
447.17000	35.0	Cluster_24974
447.25000	35.0	Cluster_08376
447.28000	35.0	Cluster_24982
447.29000	35.0	Cluster_24988
447.60000	35.0	Cluster_08380
448.30000	35.0	Cluster_25022
448.79000	35.0	Cluster_25051
448.97000	35.0	Cluster_01573
449.25000	35.0	Cluster_08416
449.74000	35.0	Cluster_25092
449.84000	35.0	Cluster_25102
450.23000	35.0	Cluster_25125
450.93000	35.0	Cluster_08435
451.14000	35.0	Cluster_25153
451.17000	35.0	Cluster_25168
451.57000	35.0	Cluster_08446
452.13000	35.0	Cluster_25195
452.26000	35.0	Cluster_08462
452.75000	35.0	Cluster_25235
453.14000	35.0	Cluster_25244
453.24000	35.0	Cluster_25248
454.68000	35.0	Cluster_25320
454.70000	35.0	Cluster_25319
454.74000	35.0	Cluster_25325
455.15000	35.0	Cluster_25343
455.18000	35.0	Cluster_01617
455.20000	35.0	Cluster_08518
456.24000	35.0	Cluster_25393
456.26000	35.0	Cluster_08536
456.57000	35.0	Cluster_08539

456.75000	35.0	Cluster_25404
456.78000	35.0	Cluster_25413
457.20000	35.0	Cluster_25422
457.75000	35.0	Cluster_25448
457.76000	35.0	Cluster_25454
458.69000	35.0	Cluster_25493
458.76000	35.0	Cluster_25500
459.22000	35.0	Cluster_08566
459.25000	35.0	Cluster_25514
459.78000	35.0	Cluster_25540
459.90000	35.0	Cluster_08580
460.27000	35.0	Cluster_25572
460.77000	35.0	Cluster_25581
460.97000	35.0	Cluster_01663
461.60000	35.0	Cluster_08592
461.70000	35.0	Cluster_25618
461.73000	35.0	Cluster_25620
462.22000	35.0	Cluster_08608
462.50000	35.0	Cluster_25643
463.61000	35.0	Cluster_25677
463.73000	35.0	Cluster_25692
464.22000	35.0	Cluster_08632
464.30000	35.0	Cluster_25714
464.55000	35.0	Cluster_08640
465.77000	35.0	Cluster_25807
465.95000	35.0	Cluster_08659
465.99000	35.0	Cluster_01692
466.27000	35.0	Cluster_25822
466.90000	35.0	Cluster_08673
467.18000	35.0	Cluster_08677
467.25000	35.0	Cluster_08678
467.30000	35.0	Cluster_25858
467.67000	35.0	Cluster_00144
467.72000	35.0	Cluster_25877
468.89000	35.0	Cluster_08711
469.26000	35.0	Cluster_08720
469.74000	35.0	Cluster_25967
469.88000	35.0	Cluster_08724
470.43000	35.0	Cluster_26002
470.56000	35.0	Cluster_00010
470.92000	35.0	Cluster_08745
471.00000	35.0	Cluster_01731
471.22000	35.0	Cluster_08748
471.25000	35.0	Cluster_01737
471.26000	35.0	Cluster_01738
471.48000	35.0	Cluster_26044
471.74000	35.0	Cluster_26063
471.84000	35.0	Cluster_26052
472.23000	35.0	Cluster_26081
472.24000	35.0	Cluster_08764
472.72000	35.0	Cluster_26107

472.76000	35.0	Cluster_26106
472.77000	35.0	Cluster_26113
473.27000	35.0	Cluster_26146
473.77000	35.0	Cluster_26157
474.54000	35.0	Cluster_08802
474.58000	35.0	Cluster_08800
474.85000	35.0	Cluster_08806
475.58000	35.0	Cluster_08820
475.78000	35.0	Cluster_26234
476.21000	35.0	Cluster_26244
476.23000	35.0	Cluster_08830
476.28000	35.0	Cluster_08832
477.07000	35.0	Cluster_26275
477.20000	35.0	Cluster_08843
477.27000	35.0	Cluster_08840
477.55000	35.0	Cluster_08851
477.61000	35.0	Cluster_08856
477.93000	35.0	Cluster_08861
478.22000	35.0	Cluster_26309
478.25000	35.0	Cluster_26313
478.76000	35.0	Cluster_26329
478.77000	35.0	Cluster_26332
478.93000	35.0	Cluster_08880
478.99000	35.0	Cluster_01780
479.57000	35.0	Cluster_08888
479.71000	35.0	Cluster_26429
479.76000	35.0	Cluster_26423
480.22000	35.0	Cluster_08900
480.70000	35.0	Cluster_26476
481.22000	35.0	Cluster_26493
481.27000	35.0	Cluster_26504
481.28000	35.0	Cluster_08920
481.56000	35.0	Cluster_08922
481.58000	35.0	Cluster_08924
481.72000	35.0	Cluster_26523
481.73000	35.0	Cluster_01804
481.79000	35.0	Cluster_26518
482.20000	35.0	Cluster_01810
482.28000	35.0	Cluster_26547
482.49000	35.0	Cluster_26549
483.24000	35.0	Cluster_26594
483.30000	35.0	Cluster_26603
483.76000	35.0	Cluster_26625
483.84000	35.0	Cluster_26627
484.09000	35.0	Cluster_26635
484.73000	35.0	Cluster_26685
484.76000	35.0	Cluster_26681
485.06000	35.0	Cluster_00166
485.54000	35.0	Cluster_08981
488.29000	35.0	Cluster_26837
488.61000	35.0	Cluster_09032

488.79000	35.0	Cluster_26859
488.95000	35.0	Cluster_09038
489.74000	35.0	Cluster_26904
489.93000	35.0	Cluster_09048
490.50000	35.0	Cluster_26931
490.70000	35.0	Cluster_26935
490.77000	35.0	Cluster_26939
492.55000	35.0	Cluster_09097
492.60000	35.0	Cluster_09099
493.27000	35.0	Cluster_27041
494.26000	35.0	Cluster_27100
495.19000	35.0	Cluster_27149
495.71000	35.0	Cluster_27170
495.95000	35.0	Cluster_09160
496.24000	35.0	Cluster_01895
496.95000	35.0	Cluster_09176
497.35000	35.0	Cluster_27258
497.74000	35.0	Cluster_27265
497.80000	35.0	Cluster_27275
497.94000	35.0	Cluster_09191
498.01000	35.0	Cluster_01929
498.50000	35.0	Cluster_27299
498.73000	35.0	Cluster_27302
499.23000	35.0	Cluster_09211
499.33000	35.0	Cluster_27324
499.76000	35.0	Cluster_01941
499.92000	35.0	Cluster_09218
499.96000	35.0	Cluster_09219
500.02000	35.0	Cluster_27355
500.63000	35.0	Cluster_09231
500.70000	35.0	Cluster_27401
500.96000	35.0	Cluster_01956
501.22000	35.0	Cluster_27436
501.59000	35.0	Cluster_09244
501.76000	35.0	Cluster_27459
502.21000	35.0	Cluster_01970
502.22000	35.0	Cluster_27473
502.59000	35.0	Cluster_09268
502.76000	35.0	Cluster_27485
503.25000	35.0	Cluster_27512
503.28000	35.0	Cluster_27518
504.21000	35.0	Cluster_27547
504.56000	35.0	Cluster_09288
504.98000	35.0	Cluster_01984
505.01000	35.0	Cluster_27567
505.24000	35.0	Cluster_09298
505.52000	35.0	Cluster_01990
505.60000	35.0	Cluster_09305
505.79000	35.0	Cluster_27593
506.73000	35.0	Cluster_27611
506.74000	35.0	Cluster_27621

506.76000	35.0	Cluster_27614
507.25000	35.0	Cluster_27638
507.97000	35.0	Cluster_09350
508.62000	35.0	Cluster_09366
508.70000	35.0	Cluster_27686
508.71000	35.0	Cluster_27692
509.20000	35.0	Cluster_27715
509.26000	35.0	Cluster_27718
509.87000	35.0	Cluster_09377
509.91000	35.0	Cluster_09383
511.23000	35.0	Cluster_27820
511.79000	35.0	Cluster_27852
511.91000	35.0	Cluster_09420
512.25000	35.0	Cluster_27867
512.27000	35.0	Cluster_27865
513.09000	35.0	Cluster_27898
513.77000	35.0	Cluster_27929
514.72000	35.0	Cluster_27964
514.74000	35.0	Cluster_27971
515.25000	35.0	Cluster_02053
515.48000	35.0	Cluster_02061
515.90000	35.0	Cluster_09478
516.74000	35.0	Cluster_28062
517.92000	35.0	Cluster_09503
518.00000	35.0	Cluster_28115
518.26000	35.0	Cluster_09508
518.31000	35.0	Cluster_28130
518.51000	35.0	Cluster_28138
518.59000	35.0	Cluster_09521
518.78000	35.0	Cluster_28152
519.59000	35.0	Cluster_09531
519.81000	35.0	Cluster_28200
520.04000	35.0	Cluster_28202
520.51000	35.0	Cluster_28225
520.80000	35.0	Cluster_28240
520.81000	35.0	Cluster_28232
521.02000	35.0	Cluster_02095
521.26000	35.0	Cluster_09553
521.66000	35.0	Cluster_28271
522.22000	35.0	Cluster_28286
522.45000	35.0	Cluster_02102
522.50000	35.0	Cluster_28303
522.62000	35.0	Cluster_09582
522.75000	35.0	Cluster_28315
523.22000	35.0	Cluster_28350
523.27000	35.0	Cluster_28334
523.35000	35.0	Cluster_28353
523.92000	35.0	Cluster_09603
523.97000	35.0	Cluster_28374
524.28000	35.0	Cluster_28386
526.18000	35.0	Cluster_28467

526.20000	35.0	Cluster_09656
526.93000	35.0	Cluster_09676
526.95000	35.0	Cluster_09677
527.76000	35.0	Cluster_28507
528.23000	35.0	Cluster_28536
528.27000	35.0	Cluster_00184
528.32000	35.0	Cluster_28539
528.36000	35.0	Cluster_28552
528.97000	35.0	Cluster_09710
529.25000	35.0	Cluster_09712
529.79000	35.0	Cluster_28615
530.20000	35.0	Cluster_28629
530.26000	35.0	Cluster_02153
530.51000	35.0	Cluster_28657
530.81000	35.0	Cluster_28677
530.94000	35.0	Cluster_09741
531.75000	35.0	Cluster_28707
531.77000	35.0	Cluster_28708
532.24000	35.0	Cluster_09763
532.27000	35.0	Cluster_09766
532.62000	35.0	Cluster_09768
532.97000	35.0	Cluster_09773
533.15000	35.0	Cluster_28752
533.16000	35.0	Cluster_28753
533.19000	35.0	Cluster_09774
533.32000	35.0	Cluster_09778
533.70000	35.0	Cluster_28783
533.82000	35.0	Cluster_28799
533.91000	35.0	Cluster_09792
534.28000	35.0	Cluster_28807
534.59000	35.0	Cluster_09803
535.32000	35.0	Cluster_09812
535.80000	35.0	Cluster_28867
535.94000	35.0	Cluster_09822
536.06000	35.0	Cluster_28878
536.26000	35.0	Cluster_09823
536.27000	35.0	Cluster_28888
536.48000	35.0	Cluster_28899
536.60000	35.0	Cluster_09834
537.23000	35.0	Cluster_09849
537.56000	35.0	Cluster_09853
537.69000	35.0	Cluster_28943
537.91000	35.0	Cluster_09869
538.84000	35.0	Cluster_28980
538.97000	35.0	Cluster_09886
538.98000	35.0	Cluster_09885
539.01000	35.0	Cluster_28985
539.28000	35.0	Cluster_28993
539.52000	35.0	Cluster_29001
540.02000	35.0	Cluster_09899
540.27000	35.0	Cluster_29031

540.79000	35.0	Cluster_29052
541.34000	35.0	Cluster_29085
541.44000	35.0	Cluster_29098
541.82000	35.0	Cluster_29115
542.26000	35.0	Cluster_02236
542.28000	35.0	Cluster_02229
542.52000	35.0	Cluster_02233
542.77000	35.0	Cluster_29162
543.27000	35.0	Cluster_29193
544.95000	35.0	Cluster_09982
545.77000	35.0	Cluster_29280
545.92000	35.0	Cluster_09992
546.31000	35.0	Cluster_29312
547.32000	35.0	Cluster_29345
548.27000	35.0	Cluster_29389
548.46000	35.0	Cluster_00194
548.61000	35.0	Cluster_10035
548.64000	35.0	Cluster_10037
549.25000	35.0	Cluster_10044
549.28000	35.0	Cluster_29418
549.90000	35.0	Cluster_10052
549.99000	35.0	Cluster_10058
550.22000	35.0	Cluster_29451
550.31000	35.0	Cluster_29461
550.55000	35.0	Cluster_10069
550.61000	35.0	Cluster_10068
550.78000	35.0	Cluster_29465
550.84000	35.0	Cluster_29476
551.01000	35.0	Cluster_29483
551.29000	35.0	Cluster_29499
551.57000	35.0	Cluster_10082
551.97000	35.0	Cluster_10089
552.36000	35.0	Cluster_29545
552.74000	35.0	Cluster_29553
552.75000	35.0	Cluster_29548
552.96000	35.0	Cluster_10104
553.55000	35.0	Cluster_29579
553.63000	35.0	Cluster_10116
554.58000	35.0	Cluster_10130
554.60000	35.0	Cluster_10136
554.97000	35.0	Cluster_10144
556.25000	35.0	Cluster_29692
556.68000	35.0	Cluster_10177
556.96000	35.0	Cluster_10184
557.73000	35.0	Cluster_29750
558.23000	35.0	Cluster_10209
558.65000	35.0	Cluster_10215
558.81000	35.0	Cluster_29792
559.29000	35.0	Cluster_29811
560.35000	35.0	Cluster_29845
560.65000	35.0	Cluster_10245

560.83000	35.0	Cluster_29862
561.28000	35.0	Cluster_29879
561.64000	35.0	Cluster_10262
561.70000	35.0	Cluster_29892
561.81000	35.0	Cluster_29901
561.98000	35.0	Cluster_10266
562.07000	35.0	Cluster_29906
562.29000	35.0	Cluster_29915
563.32000	35.0	Cluster_10288
563.50000	35.0	Cluster_02355
563.75000	35.0	Cluster_29975
563.76000	35.0	Cluster_29965
564.78000	35.0	Cluster_30003
564.79000	35.0	Cluster_02366
565.56000	35.0	Cluster_30039
565.60000	35.0	Cluster_10318
566.33000	35.0	Cluster_30080
566.74000	35.0	Cluster_02376
566.79000	35.0	Cluster_30088
566.90000	35.0	Cluster_30092
566.98000	35.0	Cluster_10333
567.07000	35.0	Cluster_30105
567.24000	35.0	Cluster_30110
567.30000	35.0	Cluster_30123
567.31000	35.0	Cluster_30114
568.56000	35.0	Cluster_02384
568.73000	35.0	Cluster_30166
568.79000	35.0	Cluster_30177
568.80000	35.0	Cluster_30176
568.85000	35.0	Cluster_30178
569.06000	35.0	Cluster_02386
569.57000	35.0	Cluster_02390
569.82000	35.0	Cluster_30204
570.30000	35.0	Cluster_02396
570.36000	35.0	Cluster_30219
570.73000	35.0	Cluster_02397
570.77000	35.0	Cluster_30236
571.22000	35.0	Cluster_30252
571.34000	35.0	Cluster_30256
571.64000	35.0	Cluster_10409
571.77000	35.0	Cluster_30264
572.13000	35.0	Cluster_00213
572.30000	35.0	Cluster_10420
572.79000	35.0	Cluster_30301
573.35000	35.0	Cluster_30317
574.26000	35.0	Cluster_10456
574.80000	35.0	Cluster_30355
575.25000	35.0	Cluster_02433
575.33000	35.0	Cluster_02435
576.55000	35.0	Cluster_02439
576.77000	35.0	Cluster_30417

576.98000	35.0	Cluster_10494
577.30000	35.0	Cluster_10500
577.81000	35.0	Cluster_30446
578.32000	35.0	Cluster_30460
578.55000	35.0	Cluster_02454
578.86000	35.0	Cluster_30486
578.98000	35.0	Cluster_10523
579.33000	35.0	Cluster_30494
579.55000	35.0	Cluster_02460
579.61000	35.0	Cluster_10531
579.63000	35.0	Cluster_10533
579.77000	35.0	Cluster_30512
579.81000	35.0	Cluster_30517
579.83000	35.0	Cluster_30515
580.36000	35.0	Cluster_30555
580.86000	35.0	Cluster_02466
581.28000	35.0	Cluster_02468
581.31000	35.0	Cluster_30577
581.96000	35.0	Cluster_10563
582.31000	35.0	Cluster_30648
582.54000	35.0	Cluster_02478
582.65000	35.0	Cluster_10574
582.70000	35.0	Cluster_00226
582.74000	35.0	Cluster_30654
584.05000	35.0	Cluster_30718
584.31000	35.0	Cluster_30724
584.54000	35.0	Cluster_30733
585.02000	35.0	Cluster_30763
585.29000	35.0	Cluster_10612
585.35000	35.0	Cluster_02501
585.80000	35.0	Cluster_30791
585.84000	35.0	Cluster_30792
586.62000	35.0	Cluster_10630
586.75000	35.0	Cluster_30815
587.30000	35.0	Cluster_10642
587.33000	35.0	Cluster_30848
587.34000	35.0	Cluster_30837
587.65000	35.0	Cluster_10646
588.69000	35.0	Cluster_30892
588.82000	35.0	Cluster_30900
589.55000	35.0	Cluster_30932
589.74000	35.0	Cluster_30933
589.96000	35.0	Cluster_10677
590.22000	35.0	Cluster_30952
590.30000	35.0	Cluster_30953
591.04000	35.0	Cluster_30998
591.44000	35.0	Cluster_31008
591.52000	35.0	Cluster_02538
591.66000	35.0	Cluster_10703
591.81000	35.0	Cluster_31027
592.01000	35.0	Cluster_10706

592.79000	35.0	Cluster_31056
592.82000	35.0	Cluster_31060
592.91000	35.0	Cluster_31063
593.06000	35.0	Cluster_02547
593.07000	35.0	Cluster_02548
593.33000	35.0	Cluster_10724
593.34000	35.0	Cluster_31073
593.93000	35.0	Cluster_10743
594.32000	35.0	Cluster_10753
595.59000	35.0	Cluster_31146
595.62000	35.0	Cluster_10769
596.75000	35.0	Cluster_31177
597.67000	35.0	Cluster_10808
597.81000	35.0	Cluster_31212
597.82000	35.0	Cluster_31208
598.29000	35.0	Cluster_10814
598.36000	35.0	Cluster_10818
599.36000	35.0	Cluster_31267
599.66000	35.0	Cluster_10840
599.95000	35.0	Cluster_10848
600.81000	35.0	Cluster_31316
600.97000	35.0	Cluster_10858
601.36000	35.0	Cluster_31339
602.32000	35.0	Cluster_31390
602.83000	35.0	Cluster_31406
603.30000	35.0	Cluster_31409
603.51000	35.0	Cluster_02598
603.53000	35.0	Cluster_31419
603.96000	35.0	Cluster_10888
604.80000	35.0	Cluster_31463
605.31000	35.0	Cluster_31473
605.67000	35.0	Cluster_10910
606.07000	35.0	Cluster_31501
606.27000	35.0	Cluster_31514
606.87000	35.0	Cluster_31531
606.89000	35.0	Cluster_10941
607.32000	35.0	Cluster_31536
608.09000	35.0	Cluster_02626
608.32000	35.0	Cluster_31587
608.49000	35.0	Cluster_31590
608.78000	35.0	Cluster_31595
608.79000	35.0	Cluster_31601
609.06000	35.0	Cluster_31605
609.81000	35.0	Cluster_31625
610.31000	35.0	Cluster_31655
611.34000	35.0	Cluster_31685
612.32000	35.0	Cluster_11010
613.83000	35.0	Cluster_31794
614.06000	35.0	Cluster_31796
614.07000	35.0	Cluster_02670
614.19000	35.0	Cluster_31797

614.26000	35.0	Cluster_11035
615.83000	35.0	Cluster_31852
616.08000	35.0	Cluster_02680
616.32000	35.0	Cluster_11075
616.55000	35.0	Cluster_31873
616.83000	35.0	Cluster_31882
617.02000	35.0	Cluster_31892
617.35000	35.0	Cluster_31911
617.93000	35.0	Cluster_31935
618.05000	35.0	Cluster_02693
618.79000	35.0	Cluster_31952
619.29000	35.0	Cluster_02701
619.33000	35.0	Cluster_31985
619.62000	35.0	Cluster_11116
620.53000	35.0	Cluster_32028
620.60000	35.0	Cluster_11128
621.30000	35.0	Cluster_32049
621.36000	35.0	Cluster_32053
621.39000	35.0	Cluster_32052
621.79000	35.0	Cluster_32057
621.98000	35.0	Cluster_11154
622.78000	35.0	Cluster_32095
622.98000	35.0	Cluster_11175
623.28000	35.0	Cluster_32112
623.36000	35.0	Cluster_32126
623.65000	35.0	Cluster_11191
623.98000	35.0	Cluster_11189
624.20000	35.0	Cluster_32153
624.28000	35.0	Cluster_32154
624.63000	35.0	Cluster_11202
625.04000	35.0	Cluster_02732
625.32000	35.0	Cluster_11203
625.58000	35.0	Cluster_02735
626.00000	35.0	Cluster_11225
626.07000	35.0	Cluster_32208
626.27000	35.0	Cluster_32218
626.28000	35.0	Cluster_32212
626.83000	35.0	Cluster_32244
627.30000	35.0	Cluster_32266
627.31000	35.0	Cluster_32263
627.50000	35.0	Cluster_32275
627.79000	35.0	Cluster_32283
628.07000	35.0	Cluster_32292
628.86000	35.0	Cluster_32321
629.63000	35.0	Cluster_11289
629.83000	35.0	Cluster_32344
629.96000	35.0	Cluster_11292
630.84000	35.0	Cluster_32375
631.37000	35.0	Cluster_11312
631.80000	35.0	Cluster_32410
632.05000	35.0	Cluster_11324

632.35000	35.0	Cluster_11329
632.78000	35.0	Cluster_32445
632.84000	35.0	Cluster_32450
633.00000	35.0	Cluster_11339
633.32000	35.0	Cluster_32465
633.36000	35.0	Cluster_32472
635.07000	35.0	Cluster_02772
635.82000	35.0	Cluster_02775
636.32000	35.0	Cluster_32584
636.84000	35.0	Cluster_32596
637.62000	35.0	Cluster_11403
637.83000	35.0	Cluster_32632
638.35000	35.0	Cluster_32655
638.36000	35.0	Cluster_11416
638.39000	35.0	Cluster_32659
638.79000	35.0	Cluster_32669
638.85000	35.0	Cluster_32673
639.85000	35.0	Cluster_32724
639.99000	35.0	Cluster_11446
640.32000	35.0	Cluster_32732
641.64000	35.0	Cluster_11472
641.84000	35.0	Cluster_32794
642.02000	35.0	Cluster_11476
643.07000	35.0	Cluster_32838
643.83000	35.0	Cluster_32863
644.00000	35.0	Cluster_11510
644.30000	35.0	Cluster_02836
644.36000	35.0	Cluster_32878
644.37000	35.0	Cluster_11511
645.00000	35.0	Cluster_11529
645.85000	35.0	Cluster_32926
646.30000	35.0	Cluster_32945
648.34000	35.0	Cluster_33005
648.67000	35.0	Cluster_11566
648.88000	35.0	Cluster_33029
649.01000	35.0	Cluster_11574
649.77000	35.0	Cluster_33054
650.31000	35.0	Cluster_33085
651.01000	35.0	Cluster_11595
651.08000	35.0	Cluster_33114
651.32000	35.0	Cluster_33124
651.36000	35.0	Cluster_11600
651.86000	35.0	Cluster_33158
651.99000	35.0	Cluster_11614
652.06000	35.0	Cluster_33159
652.35000	35.0	Cluster_33162
652.86000	35.0	Cluster_33187
653.33000	35.0	Cluster_33199
653.57000	35.0	Cluster_33207
653.60000	35.0	Cluster_02883
654.05000	35.0	Cluster_33221

654.68000	35.0	Cluster_11655
654.78000	35.0	Cluster_33237
655.99000	35.0	Cluster_11675
656.63000	35.0	Cluster_11676
657.90000	35.0	Cluster_33331
657.95000	35.0	Cluster_33350
658.07000	35.0	Cluster_33340
658.66000	35.0	Cluster_11699
659.28000	35.0	Cluster_33386
659.60000	35.0	Cluster_33396
659.63000	35.0	Cluster_02907
659.98000	35.0	Cluster_11725
660.33000	35.0	Cluster_33416
660.46000	35.0	Cluster_33423
660.84000	35.0	Cluster_33430
661.01000	35.0	Cluster_11741
661.31000	35.0	Cluster_33436
662.02000	35.0	Cluster_11748
662.12000	35.0	Cluster_02928
662.30000	35.0	Cluster_33472
663.37000	35.0	Cluster_33509
663.55000	35.0	Cluster_02941
663.60000	35.0	Cluster_02940
665.32000	35.0	Cluster_33570
666.35000	35.0	Cluster_02964
666.68000	35.0	Cluster_11814
666.95000	35.0	Cluster_33617
667.82000	35.0	Cluster_33644
668.33000	35.0	Cluster_33651
668.35000	35.0	Cluster_11848
669.10000	35.0	Cluster_02977
669.33000	35.0	Cluster_33680
669.78000	35.0	Cluster_33695
670.01000	35.0	Cluster_11876
670.40000	35.0	Cluster_33725
670.80000	35.0	Cluster_33734
670.82000	35.0	Cluster_33742
670.87000	35.0	Cluster_02986
671.44000	35.0	Cluster_33760
671.82000	35.0	Cluster_33770
671.97000	35.0	Cluster_11894
672.35000	35.0	Cluster_33781
672.81000	35.0	Cluster_33789
673.09000	35.0	Cluster_03005
673.37000	35.0	Cluster_33802
673.62000	35.0	Cluster_33824
673.65000	35.0	Cluster_11927
674.11000	35.0	Cluster_03014
674.32000	35.0	Cluster_11933
674.95000	35.0	Cluster_00274
675.37000	35.0	Cluster_33861

675.60000	35.0	Cluster_33864
675.64000	35.0	Cluster_03025
675.84000	35.0	Cluster_03026
676.00000	35.0	Cluster_11956
676.32000	35.0	Cluster_11959
677.20000	35.0	Cluster_33905
677.89000	35.0	Cluster_33946
678.81000	35.0	Cluster_33981
678.84000	35.0	Cluster_33980
678.85000	35.0	Cluster_03050
679.38000	35.0	Cluster_33996
679.79000	35.0	Cluster_34007
679.86000	35.0	Cluster_34018
680.08000	35.0	Cluster_03060
680.09000	35.0	Cluster_34023
680.87000	35.0	Cluster_34054
681.08000	35.0	Cluster_03066
681.32000	35.0	Cluster_12020
681.35000	35.0	Cluster_34074
682.09000	35.0	Cluster_34089
682.31000	35.0	Cluster_34106
682.38000	35.0	Cluster_03074
683.61000	35.0	Cluster_12059
683.81000	35.0	Cluster_34134
683.99000	35.0	Cluster_12062
684.08000	35.0	Cluster_03088
684.82000	35.0	Cluster_34171
684.83000	35.0	Cluster_34174
685.09000	35.0	Cluster_03095
685.10000	35.0	Cluster_03102
685.35000	35.0	Cluster_34201
686.04000	35.0	Cluster_12086
686.50000	35.0	Cluster_00285
686.75000	35.0	Cluster_00286
687.66000	35.0	Cluster_34255
689.34000	35.0	Cluster_34298
689.36000	35.0	Cluster_34295
689.57000	35.0	Cluster_03142
690.82000	35.0	Cluster_34324
690.84000	35.0	Cluster_34327
691.59000	35.0	Cluster_03162
691.79000	35.0	Cluster_34357
691.83000	35.0	Cluster_34369
692.26000	35.0	Cluster_34373
692.32000	35.0	Cluster_03167
692.35000	35.0	Cluster_03168
692.60000	35.0	Cluster_03182
692.84000	35.0	Cluster_34402
693.56000	35.0	Cluster_03192
693.60000	35.0	Cluster_34413
693.88000	35.0	Cluster_34418

694.36000	35.0	Cluster_34424
694.84000	35.0	Cluster_34436
694.87000	35.0	Cluster_34443
695.01000	35.0	Cluster_12183
695.36000	35.0	Cluster_34458
695.70000	35.0	Cluster_12190
695.87000	35.0	Cluster_34468
696.32000	35.0	Cluster_34489
696.34000	35.0	Cluster_03210
696.39000	35.0	Cluster_34483
696.58000	35.0	Cluster_03211
697.31000	35.0	Cluster_03215
697.37000	35.0	Cluster_34515
697.85000	35.0	Cluster_34520
697.87000	35.0	Cluster_34523
698.82000	35.0	Cluster_34541
698.83000	35.0	Cluster_34546
699.70000	35.0	Cluster_12244
699.85000	35.0	Cluster_03235
700.26000	35.0	Cluster_34580
700.36000	35.0	Cluster_34589
700.65000	35.0	Cluster_34590
700.85000	35.0	Cluster_34594
701.36000	35.0	Cluster_34614
702.34000	35.0	Cluster_34653
702.62000	35.0	Cluster_12286
703.04000	35.0	Cluster_12291
703.05000	35.0	Cluster_03266
703.33000	35.0	Cluster_34677
703.69000	35.0	Cluster_12310
703.91000	35.0	Cluster_03274
704.17000	35.0	Cluster_34698
704.21000	35.0	Cluster_34700
704.31000	35.0	Cluster_34706
704.81000	35.0	Cluster_34728
704.85000	35.0	Cluster_34734
705.04000	35.0	Cluster_12319
705.37000	35.0	Cluster_34742
705.83000	35.0	Cluster_34756
705.88000	35.0	Cluster_34757
706.86000	35.0	Cluster_03301
707.02000	35.0	Cluster_12346
707.80000	35.0	Cluster_34791
708.51000	35.0	Cluster_00309
709.28000	35.0	Cluster_34840
709.39000	35.0	Cluster_34846
710.88000	35.0	Cluster_03335
711.06000	35.0	Cluster_12397
711.85000	35.0	Cluster_34925
712.39000	35.0	Cluster_34948
712.84000	35.0	Cluster_34967

713.08000	35.0	Cluster_03351
713.29000	35.0	Cluster_03356
713.38000	35.0	Cluster_12431
713.72000	35.0	Cluster_12437
713.77000	35.0	Cluster_34987
713.92000	35.0	Cluster_34998
714.32000	35.0	Cluster_35004
714.36000	35.0	Cluster_35010
715.38000	35.0	Cluster_35041
715.54000	35.0	Cluster_00320
715.71000	35.0	Cluster_12456
715.85000	35.0	Cluster_35046
715.99000	35.0	Cluster_12465
716.39000	35.0	Cluster_35061
717.36000	35.0	Cluster_12478
717.90000	35.0	Cluster_35102
718.36000	35.0	Cluster_35122
718.85000	35.0	Cluster_35127
718.89000	35.0	Cluster_35137
718.91000	35.0	Cluster_35135
718.96000	35.0	Cluster_12511
719.39000	35.0	Cluster_35145
719.83000	35.0	Cluster_35161
719.85000	35.0	Cluster_03411
719.98000	35.0	Cluster_12519
720.12000	35.0	Cluster_35168
720.13000	35.0	Cluster_35170
720.35000	35.0	Cluster_03417
720.58000	35.0	Cluster_03421
720.83000	35.0	Cluster_35178
721.32000	35.0	Cluster_35195
721.41000	35.0	Cluster_35191
721.72000	35.0	Cluster_12548
721.88000	35.0	Cluster_35209
721.90000	35.0	Cluster_35213
722.02000	35.0	Cluster_12553
722.09000	35.0	Cluster_03436
722.34000	35.0	Cluster_03433
722.68000	35.0	Cluster_12565
723.40000	35.0	Cluster_12568
723.42000	35.0	Cluster_12570
723.95000	35.0	Cluster_00326
724.04000	35.0	Cluster_12575
724.90000	35.0	Cluster_03465
725.06000	35.0	Cluster_12593
725.32000	35.0	Cluster_03469
725.41000	35.0	Cluster_35286
725.42000	35.0	Cluster_35293
727.38000	35.0	Cluster_35317
728.10000	35.0	Cluster_03487
728.13000	35.0	Cluster_03484

728.42000	35.0	Cluster_35338
728.98000	35.0	Cluster_12653
729.15000	35.0	Cluster_03497
729.84000	35.0	Cluster_35373
729.89000	35.0	Cluster_03507
730.91000	35.0	Cluster_35408
731.10000	35.0	Cluster_35410
731.83000	35.0	Cluster_03518
732.11000	35.0	Cluster_03522
732.64000	35.0	Cluster_03534
733.03000	35.0	Cluster_12708
733.13000	35.0	Cluster_03548
733.83000	35.0	Cluster_35471
733.85000	35.0	Cluster_35474
734.58000	35.0	Cluster_35493
735.04000	35.0	Cluster_12729
735.74000	35.0	Cluster_12741
736.37000	35.0	Cluster_35541
737.04000	35.0	Cluster_12754
737.16000	35.0	Cluster_35560
737.82000	35.0	Cluster_35593
738.39000	35.0	Cluster_35611
738.63000	35.0	Cluster_03590
739.38000	35.0	Cluster_12791
739.39000	35.0	Cluster_12789
739.84000	35.0	Cluster_35645
740.37000	35.0	Cluster_35657
740.38000	35.0	Cluster_35661
740.93000	35.0	Cluster_35676
741.85000	35.0	Cluster_35683
742.37000	35.0	Cluster_35697
742.67000	35.0	Cluster_12831
742.84000	35.0	Cluster_35707
744.37000	35.0	Cluster_35750
745.38000	35.0	Cluster_35777
745.68000	35.0	Cluster_12857
746.06000	35.0	Cluster_12863
746.15000	35.0	Cluster_35792
746.30000	35.0	Cluster_35797
747.12000	35.0	Cluster_35817
747.37000	35.0	Cluster_35818
748.00000	35.0	Cluster_12903
748.86000	35.0	Cluster_03654
748.88000	35.0	Cluster_35857
749.07000	35.0	Cluster_12920
749.69000	35.0	Cluster_12929
749.98000	35.0	Cluster_12936
750.01000	35.0	Cluster_12941
750.38000	35.0	Cluster_35883
750.44000	35.0	Cluster_35886
750.94000	35.0	Cluster_35903

751.06000	35.0	Cluster_12961
751.15000	35.0	Cluster_03662
751.36000	35.0	Cluster_35908
752.37000	35.0	Cluster_35932
753.05000	35.0	Cluster_12988
753.06000	35.0	Cluster_12984
753.39000	35.0	Cluster_35964
753.91000	35.0	Cluster_35981
754.06000	35.0	Cluster_13009
754.90000	35.0	Cluster_36004
755.06000	35.0	Cluster_13021
755.30000	35.0	Cluster_13019
755.33000	35.0	Cluster_13020
755.42000	35.0	Cluster_36013
755.75000	35.0	Cluster_13034
755.92000	35.0	Cluster_36043
756.13000	35.0	Cluster_03688
757.31000	35.0	Cluster_36071
757.73000	35.0	Cluster_13076
758.41000	35.0	Cluster_36102
758.70000	35.0	Cluster_13088
759.63000	35.0	Cluster_03704
759.90000	35.0	Cluster_36129
759.91000	35.0	Cluster_36132
760.35000	35.0	Cluster_36146
761.05000	35.0	Cluster_00024
761.41000	35.0	Cluster_36182
762.38000	35.0	Cluster_13130
763.03000	35.0	Cluster_13141
763.08000	35.0	Cluster_13137
763.35000	35.0	Cluster_03726
763.71000	35.0	Cluster_13147
764.09000	35.0	Cluster_13148
764.36000	35.0	Cluster_36253
765.15000	35.0	Cluster_03741
765.79000	35.0	Cluster_36274
765.80000	35.0	Cluster_36275
766.37000	35.0	Cluster_36292
766.55000	35.0	Cluster_00339
767.70000	35.0	Cluster_13203
767.85000	35.0	Cluster_36325
768.07000	35.0	Cluster_13209
768.08000	35.0	Cluster_13205
768.38000	35.0	Cluster_36351
769.33000	35.0	Cluster_36370
769.59000	35.0	Cluster_36368
769.82000	35.0	Cluster_36385
772.41000	35.0	Cluster_36441
772.74000	35.0	Cluster_13265
772.80000	35.0	Cluster_13273
772.88000	35.0	Cluster_36459

772.92000	35.0	Cluster_36460
773.08000	35.0	Cluster_03785
773.09000	35.0	Cluster_03786
773.13000	35.0	Cluster_13272
773.35000	35.0	Cluster_13283
773.41000	35.0	Cluster_13286
774.12000	35.0	Cluster_36481
774.73000	35.0	Cluster_13306
775.06000	35.0	Cluster_13312
775.07000	35.0	Cluster_13311
775.42000	35.0	Cluster_36510
776.37000	35.0	Cluster_03803
777.15000	35.0	Cluster_03806
777.35000	35.0	Cluster_36561
777.38000	35.0	Cluster_36567
777.39000	35.0	Cluster_36564
778.37000	35.0	Cluster_36591
778.77000	35.0	Cluster_13350
778.87000	35.0	Cluster_36605
780.73000	35.0	Cluster_13379
780.81000	35.0	Cluster_36636
780.88000	35.0	Cluster_03821
781.40000	35.0	Cluster_36661
782.03000	35.0	Cluster_13403
783.13000	35.0	Cluster_03834
783.46000	35.0	Cluster_36708
783.72000	35.0	Cluster_13422
784.06000	35.0	Cluster_13433
784.89000	35.0	Cluster_36733
784.93000	35.0	Cluster_36732
785.45000	35.0	Cluster_36744
785.83000	35.0	Cluster_36763
787.06000	35.0	Cluster_13459
787.12000	35.0	Cluster_03868
787.35000	35.0	Cluster_03870
788.87000	35.0	Cluster_36840
789.06000	35.0	Cluster_13483
790.87000	35.0	Cluster_36906
791.12000	35.0	Cluster_36909
791.38000	35.0	Cluster_36914
791.90000	35.0	Cluster_36919
792.38000	35.0	Cluster_36933
792.64000	35.0	Cluster_36941
792.86000	35.0	Cluster_03914
792.87000	35.0	Cluster_36948
792.97000	35.0	Cluster_13541
793.78000	35.0	Cluster_13547
794.36000	35.0	Cluster_36984
794.37000	35.0	Cluster_36982
794.45000	35.0	Cluster_36985
794.65000	35.0	Cluster_36986

795.07000	35.0	Cluster_13571
795.42000	35.0	Cluster_37004
795.44000	35.0	Cluster_37000
795.68000	35.0	Cluster_13585
795.87000	35.0	Cluster_03947
795.90000	35.0	Cluster_37017
795.96000	35.0	Cluster_37020
796.41000	35.0	Cluster_13605
796.72000	35.0	Cluster_13597
796.91000	35.0	Cluster_37044
797.86000	35.0	Cluster_37077
797.87000	35.0	Cluster_37073
797.88000	35.0	Cluster_37072
797.89000	35.0	Cluster_37068
799.38000	35.0	Cluster_37115
799.40000	35.0	Cluster_37112
799.89000	35.0	Cluster_03975
800.35000	35.0	Cluster_37146
800.41000	35.0	Cluster_13643
800.84000	35.0	Cluster_37149
800.86000	35.0	Cluster_03991
801.06000	35.0	Cluster_00029
801.37000	35.0	Cluster_03995
801.42000	35.0	Cluster_37163
801.44000	35.0	Cluster_13660
802.43000	35.0	Cluster_37188
802.74000	35.0	Cluster_13681
803.41000	35.0	Cluster_13684
803.88000	35.0	Cluster_37226
804.43000	35.0	Cluster_37243
804.91000	35.0	Cluster_04015
805.16000	35.0	Cluster_37269
805.39000	35.0	Cluster_37283
805.94000	35.0	Cluster_37284
806.39000	35.0	Cluster_37304
807.01000	35.0	Cluster_13723
807.46000	35.0	Cluster_37330
807.92000	35.0	Cluster_37352
808.85000	35.0	Cluster_37370
808.91000	35.0	Cluster_37377
809.40000	35.0	Cluster_04037
809.91000	35.0	Cluster_37401
810.39000	35.0	Cluster_37407
810.44000	35.0	Cluster_13774
811.74000	35.0	Cluster_00036
811.86000	35.0	Cluster_37446
811.91000	35.0	Cluster_37441
812.39000	35.0	Cluster_37458
812.41000	35.0	Cluster_13796
813.82000	35.0	Cluster_00352
813.90000	35.0	Cluster_37483

814.10000	35.0	Cluster_13827
814.16000	35.0	Cluster_37492
814.17000	35.0	Cluster_04067
814.90000	35.0	Cluster_04074
815.06000	35.0	Cluster_13832
815.79000	35.0	Cluster_13844
815.92000	35.0	Cluster_37530
816.38000	35.0	Cluster_37540
816.43000	35.0	Cluster_04088
816.72000	35.0	Cluster_13857
817.34000	35.0	Cluster_37561
817.88000	35.0	Cluster_37570
818.42000	35.0	Cluster_13883
819.05000	35.0	Cluster_13897
819.42000	35.0	Cluster_13906
820.17000	35.0	Cluster_04104
821.23000	35.0	Cluster_04111
821.38000	35.0	Cluster_37637
822.06000	35.0	Cluster_13934
823.42000	35.0	Cluster_37669
823.91000	35.0	Cluster_37674
823.94000	35.0	Cluster_37688
825.32000	35.0	Cluster_37717
825.39000	35.0	Cluster_04137
826.38000	35.0	Cluster_13981
826.86000	35.0	Cluster_37765
827.04000	35.0	Cluster_13986
827.06000	35.0	Cluster_13988
827.09000	35.0	Cluster_13989
827.88000	35.0	Cluster_37794
828.80000	35.0	Cluster_14000
828.98000	35.0	Cluster_14004
829.16000	35.0	Cluster_04165
829.36000	35.0	Cluster_37821
829.43000	35.0	Cluster_37825
829.91000	35.0	Cluster_04169
830.74000	35.0	Cluster_14020
830.89000	35.0	Cluster_00042
831.45000	35.0	Cluster_37853
832.41000	35.0	Cluster_37873
832.68000	35.0	Cluster_37879
833.49000	35.0	Cluster_37894
833.99000	35.0	Cluster_37902
834.10000	35.0	Cluster_14054
834.94000	35.0	Cluster_37918
835.05000	35.0	Cluster_14062
836.39000	35.0	Cluster_14076
836.42000	35.0	Cluster_14077
836.76000	35.0	Cluster_14083
836.86000	35.0	Cluster_37959
836.94000	35.0	Cluster_04234

837.41000	35.0	Cluster_37970
837.95000	35.0	Cluster_37976
839.10000	35.0	Cluster_14102
840.66000	35.0	Cluster_04260
840.78000	35.0	Cluster_14117
841.08000	35.0	Cluster_14128
841.10000	35.0	Cluster_14126
841.67000	35.0	Cluster_00364
841.76000	35.0	Cluster_38066
842.73000	35.0	Cluster_14140
843.87000	35.0	Cluster_38114
844.18000	35.0	Cluster_04298
844.44000	35.0	Cluster_14157
846.98000	35.0	Cluster_38180
847.48000	35.0	Cluster_38200
847.75000	35.0	Cluster_14185
847.88000	35.0	Cluster_38203
848.40000	35.0	Cluster_04350
848.91000	35.0	Cluster_04352
849.46000	35.0	Cluster_38235
849.64000	35.0	Cluster_04364
849.97000	35.0	Cluster_38245
849.99000	35.0	Cluster_38248
850.33000	35.0	Cluster_38250
850.40000	35.0	Cluster_04376
850.76000	35.0	Cluster_14213
851.36000	35.0	Cluster_14215
851.77000	35.0	Cluster_38298
852.03000	35.0	Cluster_38307
852.91000	35.0	Cluster_04400
852.93000	35.0	Cluster_04401
853.45000	35.0	Cluster_38334
854.44000	35.0	Cluster_38377
854.47000	35.0	Cluster_38375
854.88000	35.0	Cluster_38385
855.41000	35.0	Cluster_38392
855.73000	35.0	Cluster_14240
856.43000	35.0	Cluster_14249
856.91000	35.0	Cluster_04444
857.06000	35.0	Cluster_14257
857.89000	35.0	Cluster_38459
857.91000	35.0	Cluster_04449
858.07000	35.0	Cluster_14269
858.77000	35.0	Cluster_14275
859.45000	35.0	Cluster_38500
859.47000	35.0	Cluster_38497
860.47000	35.0	Cluster_14284
861.39000	35.0	Cluster_04493
862.39000	35.0	Cluster_04505
862.49000	35.0	Cluster_38591
863.05000	35.0	Cluster_14302

863.76000	35.0	Cluster_14307
863.94000	35.0	Cluster_38615
864.07000	35.0	Cluster_14314
865.03000	35.0	Cluster_38646
865.75000	35.0	Cluster_14326
865.92000	35.0	Cluster_38659
865.93000	35.0	Cluster_04555
866.41000	35.0	Cluster_14334
869.07000	35.0	Cluster_14353
869.28000	35.0	Cluster_38719
869.41000	35.0	Cluster_04599
869.78000	35.0	Cluster_14363
871.17000	35.0	Cluster_38758
871.27000	35.0	Cluster_38759
871.44000	35.0	Cluster_38769
871.47000	35.0	Cluster_14388
872.27000	35.0	Cluster_38802
874.09000	35.0	Cluster_14414
874.26000	35.0	Cluster_38844
874.78000	35.0	Cluster_14424
875.28000	35.0	Cluster_38860
875.66000	35.0	Cluster_04698
876.08000	35.0	Cluster_14437
876.53000	35.0	Cluster_38895
877.38000	35.0	Cluster_14453
877.44000	35.0	Cluster_38909
878.48000	35.0	Cluster_38928
880.46000	35.0	Cluster_14483
881.96000	35.0	Cluster_38993
882.44000	35.0	Cluster_39008
882.94000	35.0	Cluster_39018
884.40000	35.0	Cluster_14528
884.66000	35.0	Cluster_39047
885.12000	35.0	Cluster_14544
885.68000	35.0	Cluster_04765
885.95000	35.0	Cluster_39078
886.43000	35.0	Cluster_39095
886.96000	35.0	Cluster_39099
887.52000	35.0	Cluster_39107
887.68000	35.0	Cluster_04781
887.74000	35.0	Cluster_14571
888.13000	35.0	Cluster_14577
888.40000	35.0	Cluster_14578
888.91000	35.0	Cluster_39135
888.92000	35.0	Cluster_04786
889.11000	35.0	Cluster_14581
889.44000	35.0	Cluster_39147
890.17000	35.0	Cluster_04796
890.38000	35.0	Cluster_39161
890.39000	35.0	Cluster_39160
890.46000	35.0	Cluster_39173

890.99000	35.0	Cluster_39180
891.46000	35.0	Cluster_39186
891.81000	35.0	Cluster_14598
892.21000	35.0	Cluster_04824
893.43000	35.0	Cluster_39231
893.78000	35.0	Cluster_14615
894.12000	35.0	Cluster_14617
894.71000	35.0	Cluster_04845
895.21000	35.0	Cluster_04851
895.45000	35.0	Cluster_14622
895.81000	35.0	Cluster_14625
896.15000	35.0	Cluster_14634
897.04000	35.0	Cluster_39292
898.77000	35.0	Cluster_14664
899.43000	35.0	Cluster_39337
899.51000	35.0	Cluster_39345
899.96000	35.0	Cluster_04891
900.94000	35.0	Cluster_39368
900.97000	35.0	Cluster_39380
900.99000	35.0	Cluster_39377
902.46000	35.0	Cluster_39412
904.46000	35.0	Cluster_39443
905.43000	35.0	Cluster_39461
906.77000	35.0	Cluster_14739
907.80000	35.0	Cluster_14749
908.46000	35.0	Cluster_39500
908.48000	35.0	Cluster_14756
908.94000	35.0	Cluster_04958
908.95000	35.0	Cluster_39504
909.44000	35.0	Cluster_14760
909.50000	35.0	Cluster_14765
909.89000	35.0	Cluster_39520
910.11000	35.0	Cluster_14769
910.19000	35.0	Cluster_04966
911.96000	35.0	Cluster_39564
914.56000	35.0	Cluster_39615
915.13000	35.0	Cluster_14823
915.93000	35.0	Cluster_39626
916.44000	35.0	Cluster_39640
916.49000	35.0	Cluster_39633
917.45000	35.0	Cluster_14854
917.97000	35.0	Cluster_39674
918.66000	35.0	Cluster_39698
920.45000	35.0	Cluster_39721
920.76000	35.0	Cluster_14891
920.79000	35.0	Cluster_39725
920.88000	35.0	Cluster_39726
920.95000	35.0	Cluster_39731
922.42000	35.0	Cluster_14907
922.98000	35.0	Cluster_39772
923.30000	35.0	Cluster_14924

923.45000	35.0	Cluster_14932
923.92000	35.0	Cluster_39782
924.49000	35.0	Cluster_39788
925.11000	35.0	Cluster_14961
926.42000	35.0	Cluster_14975
927.48000	35.0	Cluster_39834
928.47000	35.0	Cluster_39858
928.96000	35.0	Cluster_39861
929.12000	35.0	Cluster_15009
929.43000	35.0	Cluster_39880
929.90000	35.0	Cluster_39897
930.47000	35.0	Cluster_15020
931.73000	35.0	Cluster_15043
931.90000	35.0	Cluster_39924
932.64000	35.0	Cluster_15062
932.95000	35.0	Cluster_39941
933.14000	35.0	Cluster_15072
933.33000	35.0	Cluster_05038
934.00000	35.0	Cluster_39963
934.13000	35.0	Cluster_15084
934.43000	35.0	Cluster_15083
934.78000	35.0	Cluster_15089
935.78000	35.0	Cluster_15100
936.45000	35.0	Cluster_15116
936.80000	35.0	Cluster_15129
937.00000	35.0	Cluster_39997
937.44000	35.0	Cluster_15144
937.49000	35.0	Cluster_05051
938.07000	35.0	Cluster_15154
938.13000	35.0	Cluster_15156
938.21000	35.0	Cluster_15160
939.69000	35.0	Cluster_05058
940.46000	35.0	Cluster_15180
941.72000	35.0	Cluster_40098
942.00000	35.0	Cluster_40094
943.45000	35.0	Cluster_15229
945.13000	35.0	Cluster_15244
945.39000	35.0	Cluster_15242
945.72000	35.0	Cluster_05075
945.73000	35.0	Cluster_05078
945.78000	35.0	Cluster_15250
945.98000	35.0	Cluster_40152
947.16000	35.0	Cluster_15264
947.22000	35.0	Cluster_05084
947.79000	35.0	Cluster_15273
948.19000	35.0	Cluster_15277
948.52000	35.0	Cluster_40180
949.14000	35.0	Cluster_15301
949.44000	35.0	Cluster_40190
949.46000	35.0	Cluster_15302
950.44000	35.0	Cluster_15314

951.17000	35.0	Cluster_15324
951.96000	35.0	Cluster_40227
952.14000	35.0	Cluster_15337
952.15000	35.0	Cluster_15339
952.44000	35.0	Cluster_15342
952.53000	35.0	Cluster_40233
953.98000	35.0	Cluster_40255
954.82000	35.0	Cluster_15379
954.99000	35.0	Cluster_40262
955.13000	35.0	Cluster_15394
955.47000	35.0	Cluster_15404
956.48000	35.0	Cluster_15426
957.07000	35.0	Cluster_40290
957.48000	35.0	Cluster_40291
960.15000	35.0	Cluster_15462
960.98000	35.0	Cluster_40317
961.48000	35.0	Cluster_15478
962.14000	35.0	Cluster_15497
962.38000	35.0	Cluster_40344
962.79000	35.0	Cluster_15505
964.13000	35.0	Cluster_15523
964.43000	35.0	Cluster_40365
965.29000	35.0	Cluster_40377
966.15000	35.0	Cluster_15549
966.49000	35.0	Cluster_40387
966.50000	35.0	Cluster_05172
966.85000	35.0	Cluster_15552
967.00000	35.0	Cluster_40392
967.56000	35.0	Cluster_15565
968.03000	35.0	Cluster_40402
968.25000	35.0	Cluster_05187
971.97000	35.0	Cluster_40451
972.01000	35.0	Cluster_40467
972.46000	35.0	Cluster_15627
972.49000	35.0	Cluster_15626
973.51000	35.0	Cluster_40476
974.47000	35.0	Cluster_15651
974.98000	35.0	Cluster_40495
975.82000	35.0	Cluster_15672
975.98000	35.0	Cluster_05210
976.00000	35.0	Cluster_05212
976.03000	35.0	Cluster_40504
977.14000	35.0	Cluster_15696
977.62000	35.0	Cluster_40522
979.45000	35.0	Cluster_40546
979.80000	35.0	Cluster_15720
979.83000	35.0	Cluster_15721
979.98000	35.0	Cluster_40568
980.00000	35.0	Cluster_40562
980.50000	35.0	Cluster_40565
980.70000	35.0	Cluster_40578

981.45000	35.0	Cluster_15750
983.25000	35.0	Cluster_05237
983.95000	35.0	Cluster_40617
985.14000	35.0	Cluster_40628
986.02000	35.0	Cluster_40630
986.52000	35.0	Cluster_40639
986.83000	35.0	Cluster_15810
987.24000	35.0	Cluster_40654
987.80000	35.0	Cluster_15825
988.44000	35.0	Cluster_15835
991.93000	35.0	Cluster_40717
993.50000	35.0	Cluster_15895
997.19000	35.0	Cluster_15954
1000.06000	35.0	Cluster_40851
1001.03000	35.0	Cluster_40878
1001.16000	35.0	Cluster_15996
1001.49000	35.0	Cluster_40884
1003.52000	35.0	Cluster_05294
1004.00000	35.0	Cluster_40937
1004.31000	35.0	Cluster_05296
1004.57000	35.0	Cluster_40940
1007.81000	35.0	Cluster_16072
1008.78000	35.0	Cluster_16080
1012.26000	35.0	Cluster_05329
1012.27000	35.0	Cluster_41044
1012.93000	35.0	Cluster_41054
1013.01000	35.0	Cluster_05341
1013.47000	35.0	Cluster_16138
1013.49000	35.0	Cluster_16137
1013.76000	35.0	Cluster_05344
1013.90000	35.0	Cluster_41056
1015.92000	35.0	Cluster_41088
1016.78000	35.0	Cluster_05351
1017.27000	35.0	Cluster_41099
1019.25000	35.0	Cluster_05360
1020.15000	35.0	Cluster_16210
1022.50000	35.0	Cluster_16231
1023.82000	35.0	Cluster_41185
1024.48000	35.0	Cluster_16254
1024.50000	35.0	Cluster_16256
1024.93000	35.0	Cluster_41195
1025.46000	35.0	Cluster_16264
1025.81000	35.0	Cluster_41211
1026.83000	35.0	Cluster_16274
1027.13000	35.0	Cluster_16285
1027.14000	35.0	Cluster_16284
1027.88000	35.0	Cluster_16291
1028.18000	35.0	Cluster_16295
1028.84000	35.0	Cluster_16300
1029.99000	35.0	Cluster_41262
1031.20000	35.0	Cluster_16323

1032.82000	35.0	Cluster_16335
1033.20000	35.0	Cluster_16342
1036.52000	35.0	Cluster_16382
1037.50000	35.0	Cluster_16400
1039.05000	35.0	Cluster_41371
1039.52000	35.0	Cluster_41374
1039.81000	35.0	Cluster_16429
1040.83000	35.0	Cluster_05437
1041.01000	35.0	Cluster_41401
1042.17000	35.0	Cluster_16457
1044.49000	35.0	Cluster_41432
1046.13000	35.0	Cluster_16488
1046.22000	35.0	Cluster_16495
1047.16000	35.0	Cluster_16507
1047.83000	35.0	Cluster_41470
1053.50000	35.0	Cluster_16581
1053.98000	35.0	Cluster_41534
1054.99000	35.0	Cluster_05476
1055.22000	35.0	Cluster_16595
1055.50000	35.0	Cluster_16606
1056.52000	35.0	Cluster_16616
1057.06000	35.0	Cluster_41567
1057.33000	35.0	Cluster_05486
1057.34000	35.0	Cluster_05487
1057.58000	35.0	Cluster_41574
1057.99000	35.0	Cluster_41578
1058.02000	35.0	Cluster_41582
1058.81000	35.0	Cluster_16658
1058.85000	35.0	Cluster_16649
1058.96000	35.0	Cluster_41595
1058.97000	35.0	Cluster_41597
1059.01000	35.0	Cluster_41596
1060.32000	35.0	Cluster_41612
1060.75000	35.0	Cluster_05502
1060.82000	35.0	Cluster_16679
1061.26000	35.0	Cluster_05510
1061.33000	35.0	Cluster_05506
1061.82000	35.0	Cluster_41625
1062.83000	35.0	Cluster_16700
1063.27000	35.0	Cluster_05516
1063.82000	35.0	Cluster_41655
1063.99000	35.0	Cluster_41663
1064.72000	35.0	Cluster_05521
1064.73000	35.0	Cluster_41675
1064.99000	35.0	Cluster_41673
1066.26000	35.0	Cluster_41702
1067.52000	35.0	Cluster_41715
1068.05000	35.0	Cluster_41719
1068.75000	35.0	Cluster_41734
1069.51000	35.0	Cluster_41748
1070.16000	35.0	Cluster_16781

1070.51000	35.0	Cluster_05546
1072.55000	35.0	Cluster_16804
1072.76000	35.0	Cluster_05555
1074.19000	35.0	Cluster_16831
1074.51000	35.0	Cluster_41810
1075.53000	35.0	Cluster_41816
1076.75000	35.0	Cluster_05574
1078.01000	35.0	Cluster_05578
1079.49000	35.0	Cluster_05582
1080.75000	35.0	Cluster_00593
1082.00000	35.0	Cluster_05588
1083.50000	35.0	Cluster_05592
1086.59000	35.0	Cluster_41940
1091.58000	35.0	Cluster_41998
1092.90000	35.0	Cluster_17025
1093.21000	35.0	Cluster_17031
1093.55000	35.0	Cluster_17035
1093.58000	35.0	Cluster_42024
1093.99000	35.0	Cluster_42029
1094.92000	35.0	Cluster_00625
1096.55000	35.0	Cluster_42051
1100.23000	35.0	Cluster_17091
1100.56000	35.0	Cluster_17097
1101.96000	35.0	Cluster_17105
1102.54000	35.0	Cluster_17111
1103.12000	35.0	Cluster_42104
1108.23000	35.0	Cluster_17176
1108.37000	35.0	Cluster_42148
1108.88000	35.0	Cluster_17175
1109.86000	35.0	Cluster_17199
1113.50000	35.0	Cluster_42184
1113.53000	35.0	Cluster_17241
1115.18000	35.0	Cluster_17258
1115.77000	35.0	Cluster_00660
1117.22000	35.0	Cluster_17276
1118.21000	35.0	Cluster_17277
1118.22000	35.0	Cluster_17280
1119.87000	35.0	Cluster_42234
1121.54000	35.0	Cluster_17322
1123.04000	35.0	Cluster_42270
1123.97000	35.0	Cluster_42271
1124.25000	35.0	Cluster_17359
1125.57000	35.0	Cluster_42283
1126.07000	35.0	Cluster_42291
1127.88000	35.0	Cluster_17398
1128.57000	35.0	Cluster_17409
1129.21000	35.0	Cluster_17418
1129.55000	35.0	Cluster_17428
1129.90000	35.0	Cluster_17433
1130.23000	35.0	Cluster_17434
1131.58000	35.0	Cluster_17448

1133.13000	35.0	Cluster_42337
1133.20000	35.0	Cluster_17479
1133.22000	35.0	Cluster_17484
1134.90000	35.0	Cluster_17503
1135.23000	35.0	Cluster_17509
1135.51000	35.0	Cluster_17513
1135.54000	35.0	Cluster_17519
1135.55000	35.0	Cluster_17517
1136.11000	35.0	Cluster_42363
1136.88000	35.0	Cluster_17525
1136.89000	35.0	Cluster_17538
1138.13000	35.0	Cluster_42377
1140.22000	35.0	Cluster_17567
1143.18000	35.0	Cluster_00674
1143.56000	35.0	Cluster_17607
1143.60000	35.0	Cluster_42409
1144.20000	35.0	Cluster_17626
1144.24000	35.0	Cluster_17620
1145.21000	35.0	Cluster_17645
1146.57000	35.0	Cluster_00680
1149.29000	35.0	Cluster_42461
1151.05000	35.0	Cluster_42474
1152.54000	35.0	Cluster_17735
1152.88000	35.0	Cluster_17750
1153.03000	35.0	Cluster_05790
1153.76000	35.0	Cluster_00695
1154.35000	35.0	Cluster_42485
1155.34000	35.0	Cluster_42501
1156.84000	35.0	Cluster_42511
1158.84000	35.0	Cluster_42519
1159.61000	35.0	Cluster_42529
1160.34000	35.0	Cluster_42534
1162.11000	35.0	Cluster_42551
1162.64000	35.0	Cluster_42555
1163.09000	35.0	Cluster_05817
1166.08000	35.0	Cluster_05827
1166.14000	35.0	Cluster_42592
1166.21000	35.0	Cluster_17894
1167.19000	35.0	Cluster_17908
1167.57000	35.0	Cluster_17921
1168.21000	35.0	Cluster_17931
1168.33000	35.0	Cluster_42608
1168.58000	35.0	Cluster_05832
1173.07000	35.0	Cluster_42645
1180.80000	35.0	Cluster_05888
1183.23000	35.0	Cluster_18040
1187.59000	35.0	Cluster_18075
1190.56000	35.0	Cluster_18091
1192.81000	35.0	Cluster_42765
1192.92000	35.0	Cluster_18117
1195.88000	35.0	Cluster_18133

1196.63000	35.0	Cluster_42787
1197.59000	35.0	Cluster_18145
1197.90000	35.0	Cluster_18140
1198.64000	35.0	Cluster_42803
1199.32000	35.0	Cluster_05982
1199.57000	35.0	Cluster_18161
1200.26000	35.0	Cluster_18168
1200.56000	35.0	Cluster_18177
1201.89000	35.0	Cluster_18192
1203.44000	35.0	Cluster_42830
1206.62000	35.0	Cluster_42853
1208.56000	35.0	Cluster_18237
1209.51000	35.0	Cluster_42887
1213.12000	35.0	Cluster_42910
1215.15000	35.0	Cluster_42924
1218.11000	35.0	Cluster_42950
1219.09000	35.0	Cluster_42956
1221.25000	35.0	Cluster_18299
1222.10000	35.0	Cluster_42968
1224.08000	35.0	Cluster_42977
1224.60000	35.0	Cluster_42983
1225.59000	35.0	Cluster_42994
1228.07000	35.0	Cluster_43008
1228.11000	35.0	Cluster_43007
1230.63000	35.0	Cluster_43026
1232.57000	35.0	Cluster_43045
1234.58000	35.0	Cluster_43065
1238.57000	35.0	Cluster_43079
1245.28000	35.0	Cluster_18390
1249.16000	35.0	Cluster_43135
1251.87000	35.0	Cluster_43149
1253.87000	35.0	Cluster_43162
1255.86000	35.0	Cluster_43176
1257.08000	35.0	Cluster_06282
1264.27000	35.0	Cluster_18463
1267.20000	35.0	Cluster_43224
1267.68000	35.0	Cluster_43228
1274.29000	35.0	Cluster_18502
1284.15000	35.0	Cluster_43287
1300.69000	35.0	Cluster_43366
1300.70000	35.0	Cluster_43360
1300.73000	35.0	Cluster_43361
1300.89000	35.0	Cluster_43362
1301.68000	35.0	Cluster_43365
1320.21000	35.0	Cluster_43449
1329.73000	35.0	Cluster_43484
1330.45000	35.0	Cluster_06499
1331.69000	35.0	Cluster_43492
1334.68000	35.0	Cluster_43505
1349.89000	35.0	Cluster_43562
1353.71000	35.0	Cluster_43578

1354.21000	35.0	Cluster_43579
1354.66000	35.0	Cluster_06571
1361.03000	35.0	Cluster_18681
1367.16000	35.0	Cluster_43645
1372.75000	35.0	Cluster_43667
1376.18000	35.0	Cluster_43680
1376.34000	35.0	Cluster_18707
1383.67000	35.0	Cluster_18716
1394.65000	35.0	Cluster_43739
1396.44000	35.0	Cluster_18732
1402.12000	35.0	Cluster_18741
1406.24000	35.0	Cluster_43793
1407.03000	35.0	Cluster_18752
1414.35000	35.0	Cluster_18768
1417.37000	35.0	Cluster_18772
1420.01000	35.0	Cluster_18783
1422.96000	35.0	Cluster_43843
1423.47000	35.0	Cluster_43844
1424.69000	35.0	Cluster_18789
1424.79000	35.0	Cluster_43851
1425.27000	35.0	Cluster_43853
1427.03000	35.0	Cluster_18794
1428.82000	35.0	Cluster_43862
1429.02000	35.0	Cluster_18795
1430.01000	35.0	Cluster_18796
1431.68000	35.0	Cluster_18799
1437.03000	35.0	Cluster_18803
1439.35000	35.0	Cluster_18807
1459.27000	35.0	Cluster_43959
1477.30000	35.0	Cluster_44015
1489.22000	35.0	Cluster_44043
1492.94000	35.0	Cluster_44054
1495.14000	35.0	Cluster_44065
1511.80000	35.0	Cluster_44102
1518.72000	35.0	Cluster_44112
1530.33000	35.0	Cluster_44125
1560.76000	35.0	Cluster_44175
1564.32000	35.0	Cluster_44184
1571.76000	35.0	Cluster_44194
1582.85000	35.0	Cluster_44218
1616.85000	35.0	Cluster_44250
1633.97000	35.0	Cluster_44257
1635.40000	35.0	Cluster_44260
1676.82000	35.0	Cluster_44320
1683.84000	35.0	Cluster_44341
1687.92000	35.0	Cluster_44353
1739.94000	35.0	Cluster_44403
1792.99000	35.0	Cluster_44425

Reject Mass List: (none)
Neutral Loss Mass List: (none)
Product Mass List: (none)

Neutral loss in top: 3
Product in top: 3
Most intense if no parent masses found enabled
Add/subtract mass not enabled
FT master scan preview mode enabled
Charge state screening enabled
Charge state dependent ETD time not enabled
Monoisotopic precursor selection enabled
Charge state rejection enabled
Unassigned charge states : rejected
Charge state 1 : rejected
Charge state 2 : not rejected
Charge state 3 : not rejected
Charge states 4+ : not rejected
Chromatography mode is disabled

Global Data Dependent Settings:
Predict ion injection time enabled
Use global parent and reject mass lists not enabled
Exclude parent mass from data dependent selection not enabled
Exclusion mass width by mass
Exclusion mass width low: 0.50000
Exclusion mass width high: 0.50000
Parent mass width by mass
Parent mass width low: 0.50000
Parent mass width high: 0.50000
Reject mass width by mass
Reject mass width low: 0.50000
Reject mass width high: 0.50000
Zoom/UltraZoom scan mass width by mass
Zoom/UltraZoom scan mass width low: 5.00
Zoom/UltraZoom scan mass width high: 5.00
FT SIM scan mass width low: 5.00
FT SIM scan mass width high: 5.00
Neutral Loss candidates processed by decreasing intensity
Neutral Loss mass width by mass
Neutral Loss mass width low: 0.50000
Neutral Loss mass width high: 0.50000
Product candidates processed by decreasing intensity
Product mass width by mass
Product mass width low: 0.50000
Product mass width high: 0.50000
MS mass range: 0.00-1000000.00
MSn mass range by mass
MSn mass range: 0.00-1000000.00
Use m/z values as masses not enabled
Analog UV data dep. not enabled
Dynamic exclusion not enabled
Isotopic data dependence not enabled
Mass Tags data dependence not enabled
Custom Data Dependent Settings:

Not enabled

Pass 11 (wheat-mixed-digests_MS2_TO-USE-inclusion-01_4.raw):

Creator: Orbi_30393
Last modified: 10/19/2021 by Orbi_30393
MS Run Time (min): 43.00
Sequence override of method parameters not enabled.
Divert Valve: not used during run
Contact Closure: not used during run
Syringe Pump: not used during run
MS Detector Settings:
Real-time modifications to method not enabled
Stepped collision energy not enabled
Additional Microscans:

MS2	0	0
MS3	0	0
MS4	0	0
MS5	0	0
MS6	0	0
MS7	0	0
MS8	0	0
MS9	0	0
MS10	0	0

Segment 1 Information

Duration (min): 43.00
Number of Scan Events: 2
Tune Method: Orbitrap-tune-file_2020-03-13_HESI

Scan Event Details:
1: FTMS + p norm res=15000 o(300.0-2000.0)
CV = 0.0V
2: ITMS + c norm Dep MS/MS Most intense ion from (1)
Activation Type: CID
Min. Signal Required: 500.0
Isolation Width: 0.20
Normalized Coll. Energy: 35.0
Default Charge State: 2
Activation Q: 0.250
Activation Time: 10.000
CV = 0.0V

Lock Masses:
Pos List Name: N/A
Source: API Source
Mass List: (none)
Neg List Name: N/A
Source: API Source
Mass List: (none)

Data Dependent Settings:

Use separate polarity settings disabled

Parent Mass List:

MS Mass	MS FAIMS CV	MS Normalized Collision Energy	MS2 Mass	MS2 Normalized Collision Energy	Name
300.13000		35.0			Cluster_06844
300.14000		35.0			Cluster_18928
300.71000		35.0			Cluster_18955
301.50000		35.0			Cluster_06851
301.65000		35.0			Cluster_18984
302.64000		35.0			Cluster_19016
302.65000		35.0			Cluster_19017
303.99000		35.0			Cluster_19040
304.11000		35.0			Cluster_19042
304.14000		35.0			Cluster_19049
304.16000		35.0			Cluster_19050
304.20000		35.0			Cluster_19052
304.49000		35.0			Cluster_06863
304.64000		35.0			Cluster_19062
306.13000		35.0			Cluster_06873
306.67000		35.0			Cluster_19109
307.12000		35.0			Cluster_19119
307.13000		35.0			Cluster_06880
308.65000		35.0			Cluster_19177
308.66000		35.0			Cluster_19179
309.53000		35.0			Cluster_06901
310.58000		35.0			Cluster_19217
310.65000		35.0			Cluster_19222
310.66000		35.0			Cluster_19221
311.17000		35.0			Cluster_19246
311.68000		35.0			Cluster_19257
313.65000		35.0			Cluster_19315
314.82000		35.0			Cluster_06941
315.65000		35.0			Cluster_19384
316.69000		35.0			Cluster_19442
317.06000		35.0			Cluster_19459
317.16000		35.0			Cluster_19466
317.64000		35.0			Cluster_19490
318.64000		35.0			Cluster_19532
318.85000		35.0			Cluster_06973
319.17000		35.0			Cluster_19552
319.67000		35.0			Cluster_19560
319.94000		35.0			Cluster_00850
320.49000		35.0			Cluster_06993
320.80000		35.0			Cluster_06994
321.15000		35.0			Cluster_06995
321.17000		35.0			Cluster_00869
321.52000		35.0			Cluster_19594

322.15000	35.0	Cluster_19619
322.17000	35.0	Cluster_19617
322.50000	35.0	Cluster_06998
323.65000	35.0	Cluster_19672
323.69000	35.0	Cluster_19677
323.83000	35.0	Cluster_19683
324.42000	35.0	Cluster_00878
324.60000	35.0	Cluster_19711
324.65000	35.0	Cluster_19713
324.83000	35.0	Cluster_07017
325.14000	35.0	Cluster_19730
325.82000	35.0	Cluster_07023
325.85000	35.0	Cluster_07028
326.17000	35.0	Cluster_19753
326.40000	35.0	Cluster_00887
326.90000	35.0	Cluster_00888
327.21000	35.0	Cluster_19791
327.96000	35.0	Cluster_07043
328.48000	35.0	Cluster_07051
329.18000	35.0	Cluster_19855
329.82000	35.0	Cluster_07063
329.93000	35.0	Cluster_19873
330.18000	35.0	Cluster_19886
330.90000	35.0	Cluster_19916
331.17000	35.0	Cluster_19926
331.20000	35.0	Cluster_19928
331.21000	35.0	Cluster_19930
331.22000	35.0	Cluster_19925
331.66000	35.0	Cluster_19936
332.14000	35.0	Cluster_19948
333.47000	35.0	Cluster_07081
333.82000	35.0	Cluster_07087
333.87000	35.0	Cluster_07090
335.12000	35.0	Cluster_20034
335.83000	35.0	Cluster_07104
336.19000	35.0	Cluster_07109
336.23000	35.0	Cluster_20093
336.91000	35.0	Cluster_00920
337.19000	35.0	Cluster_20132
337.21000	35.0	Cluster_20126
337.83000	35.0	Cluster_07126
338.11000	35.0	Cluster_20150
338.67000	35.0	Cluster_20172
339.16000	35.0	Cluster_20192
339.47000	35.0	Cluster_07139
339.81000	35.0	Cluster_07150
339.94000	35.0	Cluster_00929
340.18000	35.0	Cluster_20223
340.26000	35.0	Cluster_20229
341.15000	35.0	Cluster_20257
341.16000	35.0	Cluster_20261

342.16000	35.0	Cluster_07167
343.11000	35.0	Cluster_20316
343.18000	35.0	Cluster_20318
343.66000	35.0	Cluster_20344
345.19000	35.0	Cluster_20390
345.67000	35.0	Cluster_20402
346.15000	35.0	Cluster_07199
346.67000	35.0	Cluster_20444
347.20000	35.0	Cluster_20457
347.71000	35.0	Cluster_20471
348.44000	35.0	Cluster_20495
348.87000	35.0	Cluster_07234
348.92000	35.0	Cluster_00975
348.93000	35.0	Cluster_00976
349.20000	35.0	Cluster_20517
349.21000	35.0	Cluster_20520
349.64000	35.0	Cluster_20543
349.73000	35.0	Cluster_20549
350.42000	35.0	Cluster_00982
351.18000	35.0	Cluster_20611
351.19000	35.0	Cluster_20622
352.66000	35.0	Cluster_20669
353.17000	35.0	Cluster_01002
353.21000	35.0	Cluster_20705
353.66000	35.0	Cluster_01003
353.80000	35.0	Cluster_07278
353.87000	35.0	Cluster_20720
353.91000	35.0	Cluster_01004
354.16000	35.0	Cluster_20732
354.20000	35.0	Cluster_20739
354.40000	35.0	Cluster_01009
354.42000	35.0	Cluster_01011
355.14000	35.0	Cluster_20781
355.16000	35.0	Cluster_20782
355.22000	35.0	Cluster_20787
355.36000	35.0	Cluster_20789
355.70000	35.0	Cluster_20796
356.24000	35.0	Cluster_20824
356.55000	35.0	Cluster_07307
358.42000	35.0	Cluster_20933
359.13000	35.0	Cluster_07326
359.17000	35.0	Cluster_00118
359.37000	35.0	Cluster_20960
360.16000	35.0	Cluster_20986
360.42000	35.0	Cluster_01047
360.83000	35.0	Cluster_21008
361.18000	35.0	Cluster_21024
361.42000	35.0	Cluster_01055
361.66000	35.0	Cluster_21038
361.67000	35.0	Cluster_21036
361.69000	35.0	Cluster_21039

361.84000	35.0	Cluster_07347
362.10000	35.0	Cluster_21048
362.42000	35.0	Cluster_01059
362.43000	35.0	Cluster_21075
362.60000	35.0	Cluster_21077
362.65000	35.0	Cluster_21076
363.11000	35.0	Cluster_21089
363.19000	35.0	Cluster_21093
363.20000	35.0	Cluster_21094
363.77000	35.0	Cluster_21121
364.16000	35.0	Cluster_21135
364.50000	35.0	Cluster_21161
364.65000	35.0	Cluster_21162
365.24000	35.0	Cluster_21221
365.79000	35.0	Cluster_07396
365.90000	35.0	Cluster_21241
366.27000	35.0	Cluster_21267
366.74000	35.0	Cluster_21285
367.22000	35.0	Cluster_21306
367.50000	35.0	Cluster_07416
367.53000	35.0	Cluster_21324
368.16000	35.0	Cluster_21368
368.20000	35.0	Cluster_21366
368.47000	35.0	Cluster_07428
369.67000	35.0	Cluster_21437
370.08000	35.0	Cluster_21453
370.50000	35.0	Cluster_07445
370.74000	35.0	Cluster_21476
370.93000	35.0	Cluster_21486
371.18000	35.0	Cluster_07449
371.24000	35.0	Cluster_21501
371.71000	35.0	Cluster_21517
371.89000	35.0	Cluster_21531
372.17000	35.0	Cluster_21537
372.23000	35.0	Cluster_07464
372.67000	35.0	Cluster_21575
372.69000	35.0	Cluster_21572
373.12000	35.0	Cluster_21589
373.14000	35.0	Cluster_21585
373.16000	35.0	Cluster_07470
373.19000	35.0	Cluster_07471
373.22000	35.0	Cluster_21597
373.90000	35.0	Cluster_21628
374.19000	35.0	Cluster_07480
374.40000	35.0	Cluster_21650
374.68000	35.0	Cluster_21656
375.15000	35.0	Cluster_21668
375.89000	35.0	Cluster_07496
377.48000	35.0	Cluster_07512
377.77000	35.0	Cluster_21780
378.17000	35.0	Cluster_01122

378.18000	35.0	Cluster_21788
378.20000	35.0	Cluster_07523
378.67000	35.0	Cluster_01125
379.26000	35.0	Cluster_21841
380.45000	35.0	Cluster_21886
380.68000	35.0	Cluster_21891
381.22000	35.0	Cluster_07550
381.70000	35.0	Cluster_01141
382.18000	35.0	Cluster_21953
382.66000	35.0	Cluster_01144
383.63000	35.0	Cluster_22024
383.68000	35.0	Cluster_22025
384.16000	35.0	Cluster_01146
384.21000	35.0	Cluster_22055
384.22000	35.0	Cluster_22065
385.52000	35.0	Cluster_07597
385.54000	35.0	Cluster_07596
385.86000	35.0	Cluster_22123
386.66000	35.0	Cluster_22155
386.68000	35.0	Cluster_22158
386.90000	35.0	Cluster_22172
387.11000	35.0	Cluster_22177
387.63000	35.0	Cluster_22217
387.93000	35.0	Cluster_22226
388.23000	35.0	Cluster_22233
388.50000	35.0	Cluster_07621
388.64000	35.0	Cluster_22244
388.67000	35.0	Cluster_22245
388.83000	35.0	Cluster_07623
389.25000	35.0	Cluster_22269
389.51000	35.0	Cluster_07627
389.91000	35.0	Cluster_22284
390.71000	35.0	Cluster_22316
390.87000	35.0	Cluster_07651
390.88000	35.0	Cluster_07646
391.50000	35.0	Cluster_07654
391.65000	35.0	Cluster_22334
391.75000	35.0	Cluster_22344
391.82000	35.0	Cluster_07658
392.22000	35.0	Cluster_07665
392.25000	35.0	Cluster_22358
392.73000	35.0	Cluster_22381
392.77000	35.0	Cluster_22377
393.19000	35.0	Cluster_01195
393.53000	35.0	Cluster_07674
393.69000	35.0	Cluster_01197
393.71000	35.0	Cluster_22428
393.98000	35.0	Cluster_22479
394.21000	35.0	Cluster_22490
394.71000	35.0	Cluster_22522
394.72000	35.0	Cluster_22521

395.89000	35.0	Cluster_07703
396.23000	35.0	Cluster_22589
396.30000	35.0	Cluster_22595
397.39000	35.0	Cluster_22628
397.67000	35.0	Cluster_22634
397.72000	35.0	Cluster_22641
397.90000	35.0	Cluster_07736
398.18000	35.0	Cluster_01226
398.44000	35.0	Cluster_01230
400.24000	35.0	Cluster_22747
400.68000	35.0	Cluster_22780
400.90000	35.0	Cluster_07771
401.70000	35.0	Cluster_22833
402.32000	35.0	Cluster_22866
402.48000	35.0	Cluster_22868
402.72000	35.0	Cluster_22878
403.44000	35.0	Cluster_01260
403.54000	35.0	Cluster_07797
404.47000	35.0	Cluster_01272
405.88000	35.0	Cluster_07831
406.22000	35.0	Cluster_23023
406.68000	35.0	Cluster_01285
406.73000	35.0	Cluster_23046
406.95000	35.0	Cluster_01293
407.17000	35.0	Cluster_23059
407.25000	35.0	Cluster_23066
407.67000	35.0	Cluster_01302
407.75000	35.0	Cluster_23083
407.93000	35.0	Cluster_01303
408.22000	35.0	Cluster_23104
408.54000	35.0	Cluster_07863
408.66000	35.0	Cluster_01305
408.91000	35.0	Cluster_07864
408.92000	35.0	Cluster_01307
409.19000	35.0	Cluster_07867
409.25000	35.0	Cluster_23160
409.69000	35.0	Cluster_23170
410.53000	35.0	Cluster_07882
411.72000	35.0	Cluster_23258
412.67000	35.0	Cluster_23299
412.68000	35.0	Cluster_01326
413.23000	35.0	Cluster_23320
413.87000	35.0	Cluster_07913
413.92000	35.0	Cluster_07916
413.94000	35.0	Cluster_23350
413.98000	35.0	Cluster_23354
414.71000	35.0	Cluster_23379
414.85000	35.0	Cluster_23397
414.87000	35.0	Cluster_07924
415.22000	35.0	Cluster_23422
415.73000	35.0	Cluster_23434

416.00000	35.0	Cluster_23448
416.22000	35.0	Cluster_23463
416.76000	35.0	Cluster_23476
416.88000	35.0	Cluster_23482
416.94000	35.0	Cluster_01353
418.25000	35.0	Cluster_23534
418.47000	35.0	Cluster_23549
419.18000	35.0	Cluster_01360
419.21000	35.0	Cluster_07978
419.88000	35.0	Cluster_23600
420.19000	35.0	Cluster_23617
420.23000	35.0	Cluster_23612
420.71000	35.0	Cluster_23632
421.56000	35.0	Cluster_08005
422.12000	35.0	Cluster_08008
422.55000	35.0	Cluster_08014
422.58000	35.0	Cluster_08015
423.14000	35.0	Cluster_23727
423.21000	35.0	Cluster_08022
423.24000	35.0	Cluster_23740
423.44000	35.0	Cluster_01387
423.71000	35.0	Cluster_23753
423.74000	35.0	Cluster_23766
424.18000	35.0	Cluster_23787
424.51000	35.0	Cluster_23811
424.68000	35.0	Cluster_23813
425.18000	35.0	Cluster_23850
425.46000	35.0	Cluster_23865
425.87000	35.0	Cluster_08056
425.88000	35.0	Cluster_23895
426.14000	35.0	Cluster_23901
426.24000	35.0	Cluster_23924
426.44000	35.0	Cluster_23942
426.88000	35.0	Cluster_08077
427.51000	35.0	Cluster_08094
428.56000	35.0	Cluster_08107
428.72000	35.0	Cluster_24045
428.79000	35.0	Cluster_24059
429.24000	35.0	Cluster_24074
429.57000	35.0	Cluster_08120
429.72000	35.0	Cluster_24100
429.76000	35.0	Cluster_24093
429.85000	35.0	Cluster_08124
429.95000	35.0	Cluster_01432
430.73000	35.0	Cluster_24136
431.21000	35.0	Cluster_24172
431.41000	35.0	Cluster_24179
431.74000	35.0	Cluster_24194
432.16000	35.0	Cluster_24207
432.21000	35.0	Cluster_24215
432.87000	35.0	Cluster_08164

433.96000	35.0	Cluster_24289
434.79000	35.0	Cluster_24347
434.97000	35.0	Cluster_24350
435.17000	35.0	Cluster_24357
435.24000	35.0	Cluster_08201
435.37000	35.0	Cluster_24383
435.70000	35.0	Cluster_01481
435.97000	35.0	Cluster_01487
436.48000	35.0	Cluster_01491
437.37000	35.0	Cluster_24491
437.46000	35.0	Cluster_01497
438.22000	35.0	Cluster_24532
438.30000	35.0	Cluster_24543
438.96000	35.0	Cluster_01503
438.97000	35.0	Cluster_01504
439.82000	35.0	Cluster_24632
440.21000	35.0	Cluster_01512
440.56000	35.0	Cluster_24655
440.74000	35.0	Cluster_24659
440.86000	35.0	Cluster_08277
441.21000	35.0	Cluster_24682
441.24000	35.0	Cluster_01516
441.73000	35.0	Cluster_24703
441.87000	35.0	Cluster_08293
442.00000	35.0	Cluster_24716
442.28000	35.0	Cluster_24730
442.57000	35.0	Cluster_08303
442.77000	35.0	Cluster_24758
442.89000	35.0	Cluster_24763
442.98000	35.0	Cluster_24761
443.12000	35.0	Cluster_24771
443.22000	35.0	Cluster_24782
443.28000	35.0	Cluster_24785
443.29000	35.0	Cluster_24795
443.89000	35.0	Cluster_08322
443.92000	35.0	Cluster_08324
444.72000	35.0	Cluster_24866
445.18000	35.0	Cluster_08343
445.55000	35.0	Cluster_24900
446.27000	35.0	Cluster_24941
446.73000	35.0	Cluster_24959
446.91000	35.0	Cluster_08369
447.17000	35.0	Cluster_24974
447.25000	35.0	Cluster_08376
447.28000	35.0	Cluster_24982
447.29000	35.0	Cluster_24988
447.60000	35.0	Cluster_08380
448.30000	35.0	Cluster_25022
448.79000	35.0	Cluster_25051
448.97000	35.0	Cluster_01573
449.25000	35.0	Cluster_08416

449.74000	35.0	Cluster_25092
449.84000	35.0	Cluster_25102
450.23000	35.0	Cluster_25125
450.93000	35.0	Cluster_08435
451.14000	35.0	Cluster_25153
451.17000	35.0	Cluster_25168
451.57000	35.0	Cluster_08446
452.13000	35.0	Cluster_25195
452.26000	35.0	Cluster_08462
452.75000	35.0	Cluster_25235
453.14000	35.0	Cluster_25244
453.24000	35.0	Cluster_25248
454.68000	35.0	Cluster_25320
454.70000	35.0	Cluster_25319
454.74000	35.0	Cluster_25325
455.15000	35.0	Cluster_25343
455.18000	35.0	Cluster_01617
455.20000	35.0	Cluster_08518
456.24000	35.0	Cluster_25393
456.26000	35.0	Cluster_08536
456.57000	35.0	Cluster_08539
456.75000	35.0	Cluster_25404
456.78000	35.0	Cluster_25413
457.20000	35.0	Cluster_25422
457.75000	35.0	Cluster_25448
457.76000	35.0	Cluster_25454
458.69000	35.0	Cluster_25493
458.76000	35.0	Cluster_25500
459.22000	35.0	Cluster_08566
459.25000	35.0	Cluster_25514
459.78000	35.0	Cluster_25540
459.90000	35.0	Cluster_08580
460.27000	35.0	Cluster_25572
460.77000	35.0	Cluster_25581
460.97000	35.0	Cluster_01663
461.60000	35.0	Cluster_08592
461.70000	35.0	Cluster_25618
461.73000	35.0	Cluster_25620
462.22000	35.0	Cluster_08608
462.50000	35.0	Cluster_25643
463.61000	35.0	Cluster_25677
463.73000	35.0	Cluster_25692
464.22000	35.0	Cluster_08632
464.30000	35.0	Cluster_25714
464.55000	35.0	Cluster_08640
465.77000	35.0	Cluster_25807
465.95000	35.0	Cluster_08659
465.99000	35.0	Cluster_01692
466.27000	35.0	Cluster_25822
466.90000	35.0	Cluster_08673
467.18000	35.0	Cluster_08677

467.25000	35.0	Cluster_08678
467.30000	35.0	Cluster_25858
467.67000	35.0	Cluster_00144
467.72000	35.0	Cluster_25877
468.89000	35.0	Cluster_08711
469.26000	35.0	Cluster_08720
469.74000	35.0	Cluster_25967
469.88000	35.0	Cluster_08724
470.43000	35.0	Cluster_26002
470.56000	35.0	Cluster_00010
470.92000	35.0	Cluster_08745
471.00000	35.0	Cluster_01731
471.22000	35.0	Cluster_08748
471.25000	35.0	Cluster_01737
471.26000	35.0	Cluster_01738
471.48000	35.0	Cluster_26044
471.74000	35.0	Cluster_26063
471.84000	35.0	Cluster_26052
472.23000	35.0	Cluster_26081
472.24000	35.0	Cluster_08764
472.72000	35.0	Cluster_26107
472.76000	35.0	Cluster_26106
472.77000	35.0	Cluster_26113
473.27000	35.0	Cluster_26146
473.77000	35.0	Cluster_26157
474.54000	35.0	Cluster_08802
474.58000	35.0	Cluster_08800
474.85000	35.0	Cluster_08806
475.58000	35.0	Cluster_08820
475.78000	35.0	Cluster_26234
476.21000	35.0	Cluster_26244
476.23000	35.0	Cluster_08830
476.28000	35.0	Cluster_08832
477.07000	35.0	Cluster_26275
477.20000	35.0	Cluster_08843
477.27000	35.0	Cluster_08840
477.55000	35.0	Cluster_08851
477.61000	35.0	Cluster_08856
477.93000	35.0	Cluster_08861
478.22000	35.0	Cluster_26309
478.25000	35.0	Cluster_26313
478.76000	35.0	Cluster_26329
478.77000	35.0	Cluster_26332
478.93000	35.0	Cluster_08880
478.99000	35.0	Cluster_01780
479.57000	35.0	Cluster_08888
479.71000	35.0	Cluster_26429
479.76000	35.0	Cluster_26423
480.22000	35.0	Cluster_08900
480.70000	35.0	Cluster_26476
481.22000	35.0	Cluster_26493

481.27000	35.0	Cluster_26504
481.28000	35.0	Cluster_08920
481.56000	35.0	Cluster_08922
481.58000	35.0	Cluster_08924
481.72000	35.0	Cluster_26523
481.73000	35.0	Cluster_01804
481.79000	35.0	Cluster_26518
482.20000	35.0	Cluster_01810
482.28000	35.0	Cluster_26547
482.49000	35.0	Cluster_26549
483.24000	35.0	Cluster_26594
483.30000	35.0	Cluster_26603
483.76000	35.0	Cluster_26625
483.84000	35.0	Cluster_26627
484.09000	35.0	Cluster_26635
484.73000	35.0	Cluster_26685
484.76000	35.0	Cluster_26681
485.06000	35.0	Cluster_00166
485.54000	35.0	Cluster_08981
488.29000	35.0	Cluster_26837
488.61000	35.0	Cluster_09032
488.79000	35.0	Cluster_26859
488.95000	35.0	Cluster_09038
489.74000	35.0	Cluster_26904
489.93000	35.0	Cluster_09048
490.50000	35.0	Cluster_26931
490.70000	35.0	Cluster_26935
490.77000	35.0	Cluster_26939
492.55000	35.0	Cluster_09097
492.60000	35.0	Cluster_09099
493.27000	35.0	Cluster_27041
494.26000	35.0	Cluster_27100
495.19000	35.0	Cluster_27149
495.71000	35.0	Cluster_27170
495.95000	35.0	Cluster_09160
496.24000	35.0	Cluster_01895
496.95000	35.0	Cluster_09176
497.35000	35.0	Cluster_27258
497.74000	35.0	Cluster_27265
497.80000	35.0	Cluster_27275
497.94000	35.0	Cluster_09191
498.01000	35.0	Cluster_01929
498.50000	35.0	Cluster_27299
498.73000	35.0	Cluster_27302
499.23000	35.0	Cluster_09211
499.33000	35.0	Cluster_27324
499.76000	35.0	Cluster_01941
499.92000	35.0	Cluster_09218
499.96000	35.0	Cluster_09219
500.02000	35.0	Cluster_27355
500.63000	35.0	Cluster_09231

500.70000	35.0	Cluster_27401
500.96000	35.0	Cluster_01956
501.22000	35.0	Cluster_27436
501.59000	35.0	Cluster_09244
501.76000	35.0	Cluster_27459
502.21000	35.0	Cluster_01970
502.22000	35.0	Cluster_27473
502.59000	35.0	Cluster_09268
502.76000	35.0	Cluster_27485
503.25000	35.0	Cluster_27512
503.28000	35.0	Cluster_27518
504.21000	35.0	Cluster_27547
504.56000	35.0	Cluster_09288
504.98000	35.0	Cluster_01984
505.01000	35.0	Cluster_27567
505.24000	35.0	Cluster_09298
505.52000	35.0	Cluster_01990
505.60000	35.0	Cluster_09305
505.79000	35.0	Cluster_27593
506.73000	35.0	Cluster_27611
506.74000	35.0	Cluster_27621
506.76000	35.0	Cluster_27614
507.25000	35.0	Cluster_27638
507.97000	35.0	Cluster_09350
508.62000	35.0	Cluster_09366
508.70000	35.0	Cluster_27686
508.71000	35.0	Cluster_27692
509.20000	35.0	Cluster_27715
509.26000	35.0	Cluster_27718
509.87000	35.0	Cluster_09377
509.91000	35.0	Cluster_09383
511.23000	35.0	Cluster_27820
511.79000	35.0	Cluster_27852
511.91000	35.0	Cluster_09420
512.25000	35.0	Cluster_27867
512.27000	35.0	Cluster_27865
513.09000	35.0	Cluster_27898
513.77000	35.0	Cluster_27929
514.72000	35.0	Cluster_27964
514.74000	35.0	Cluster_27971
515.25000	35.0	Cluster_02053
515.48000	35.0	Cluster_02061
515.90000	35.0	Cluster_09478
516.74000	35.0	Cluster_28062
517.92000	35.0	Cluster_09503
518.00000	35.0	Cluster_28115
518.26000	35.0	Cluster_09508
518.31000	35.0	Cluster_28130
518.51000	35.0	Cluster_28138
518.59000	35.0	Cluster_09521
518.78000	35.0	Cluster_28152

519.59000	35.0	Cluster_09531
519.81000	35.0	Cluster_28200
520.04000	35.0	Cluster_28202
520.51000	35.0	Cluster_28225
520.80000	35.0	Cluster_28240
520.81000	35.0	Cluster_28232
521.02000	35.0	Cluster_02095
521.26000	35.0	Cluster_09553
521.66000	35.0	Cluster_28271
522.22000	35.0	Cluster_28286
522.45000	35.0	Cluster_02102
522.50000	35.0	Cluster_28303
522.62000	35.0	Cluster_09582
522.75000	35.0	Cluster_28315
523.22000	35.0	Cluster_28350
523.27000	35.0	Cluster_28334
523.35000	35.0	Cluster_28353
523.92000	35.0	Cluster_09603
523.97000	35.0	Cluster_28374
524.28000	35.0	Cluster_28386
526.18000	35.0	Cluster_28467
526.20000	35.0	Cluster_09656
526.93000	35.0	Cluster_09676
526.95000	35.0	Cluster_09677
527.76000	35.0	Cluster_28507
528.23000	35.0	Cluster_28536
528.27000	35.0	Cluster_00184
528.32000	35.0	Cluster_28539
528.36000	35.0	Cluster_28552
528.97000	35.0	Cluster_09710
529.25000	35.0	Cluster_09712
529.79000	35.0	Cluster_28615
530.20000	35.0	Cluster_28629
530.26000	35.0	Cluster_02153
530.51000	35.0	Cluster_28657
530.81000	35.0	Cluster_28677
530.94000	35.0	Cluster_09741
531.75000	35.0	Cluster_28707
531.77000	35.0	Cluster_28708
532.24000	35.0	Cluster_09763
532.27000	35.0	Cluster_09766
532.62000	35.0	Cluster_09768
532.97000	35.0	Cluster_09773
533.15000	35.0	Cluster_28752
533.16000	35.0	Cluster_28753
533.19000	35.0	Cluster_09774
533.32000	35.0	Cluster_09778
533.70000	35.0	Cluster_28783
533.82000	35.0	Cluster_28799
533.91000	35.0	Cluster_09792
534.28000	35.0	Cluster_28807

534.59000	35.0	Cluster_09803
535.32000	35.0	Cluster_09812
535.80000	35.0	Cluster_28867
535.94000	35.0	Cluster_09822
536.06000	35.0	Cluster_28878
536.26000	35.0	Cluster_09823
536.27000	35.0	Cluster_28888
536.48000	35.0	Cluster_28899
536.60000	35.0	Cluster_09834
537.23000	35.0	Cluster_09849
537.56000	35.0	Cluster_09853
537.69000	35.0	Cluster_28943
537.91000	35.0	Cluster_09869
538.84000	35.0	Cluster_28980
538.97000	35.0	Cluster_09886
538.98000	35.0	Cluster_09885
539.01000	35.0	Cluster_28985
539.28000	35.0	Cluster_28993
539.52000	35.0	Cluster_29001
540.02000	35.0	Cluster_09899
540.27000	35.0	Cluster_29031
540.79000	35.0	Cluster_29052
541.34000	35.0	Cluster_29085
541.44000	35.0	Cluster_29098
541.82000	35.0	Cluster_29115
542.26000	35.0	Cluster_02236
542.28000	35.0	Cluster_02229
542.52000	35.0	Cluster_02233
542.77000	35.0	Cluster_29162
543.27000	35.0	Cluster_29193
544.95000	35.0	Cluster_09982
545.77000	35.0	Cluster_29280
545.92000	35.0	Cluster_09992
546.31000	35.0	Cluster_29312
547.32000	35.0	Cluster_29345
548.27000	35.0	Cluster_29389
548.46000	35.0	Cluster_00194
548.61000	35.0	Cluster_10035
548.64000	35.0	Cluster_10037
549.25000	35.0	Cluster_10044
549.28000	35.0	Cluster_29418
549.90000	35.0	Cluster_10052
549.99000	35.0	Cluster_10058
550.22000	35.0	Cluster_29451
550.31000	35.0	Cluster_29461
550.55000	35.0	Cluster_10069
550.61000	35.0	Cluster_10068
550.78000	35.0	Cluster_29465
550.84000	35.0	Cluster_29476
551.01000	35.0	Cluster_29483
551.29000	35.0	Cluster_29499

551.57000	35.0	Cluster_10082
551.97000	35.0	Cluster_10089
552.36000	35.0	Cluster_29545
552.74000	35.0	Cluster_29553
552.75000	35.0	Cluster_29548
552.96000	35.0	Cluster_10104
553.55000	35.0	Cluster_29579
553.63000	35.0	Cluster_10116
554.58000	35.0	Cluster_10130
554.60000	35.0	Cluster_10136
554.97000	35.0	Cluster_10144
556.25000	35.0	Cluster_29692
556.68000	35.0	Cluster_10177
556.96000	35.0	Cluster_10184
557.73000	35.0	Cluster_29750
558.23000	35.0	Cluster_10209
558.65000	35.0	Cluster_10215
558.81000	35.0	Cluster_29792
559.29000	35.0	Cluster_29811
560.35000	35.0	Cluster_29845
560.65000	35.0	Cluster_10245
560.83000	35.0	Cluster_29862
561.28000	35.0	Cluster_29879
561.64000	35.0	Cluster_10262
561.70000	35.0	Cluster_29892
561.81000	35.0	Cluster_29901
561.98000	35.0	Cluster_10266
562.07000	35.0	Cluster_29906
562.29000	35.0	Cluster_29915
563.32000	35.0	Cluster_10288
563.50000	35.0	Cluster_02355
563.75000	35.0	Cluster_29975
563.76000	35.0	Cluster_29965
564.78000	35.0	Cluster_30003
564.79000	35.0	Cluster_02366
565.56000	35.0	Cluster_30039
565.60000	35.0	Cluster_10318
566.33000	35.0	Cluster_30080
566.74000	35.0	Cluster_02376
566.79000	35.0	Cluster_30088
566.90000	35.0	Cluster_30092
566.98000	35.0	Cluster_10333
567.07000	35.0	Cluster_30105
567.24000	35.0	Cluster_30110
567.30000	35.0	Cluster_30123
567.31000	35.0	Cluster_30114
568.56000	35.0	Cluster_02384
568.73000	35.0	Cluster_30166
568.79000	35.0	Cluster_30177
568.80000	35.0	Cluster_30176
568.85000	35.0	Cluster_30178

569.06000	35.0	Cluster_02386
569.57000	35.0	Cluster_02390
569.82000	35.0	Cluster_30204
570.30000	35.0	Cluster_02396
570.36000	35.0	Cluster_30219
570.73000	35.0	Cluster_02397
570.77000	35.0	Cluster_30236
571.22000	35.0	Cluster_30252
571.34000	35.0	Cluster_30256
571.64000	35.0	Cluster_10409
571.77000	35.0	Cluster_30264
572.13000	35.0	Cluster_00213
572.30000	35.0	Cluster_10420
572.79000	35.0	Cluster_30301
573.35000	35.0	Cluster_30317
574.26000	35.0	Cluster_10456
574.80000	35.0	Cluster_30355
575.25000	35.0	Cluster_02433
575.33000	35.0	Cluster_02435
576.55000	35.0	Cluster_02439
576.77000	35.0	Cluster_30417
576.98000	35.0	Cluster_10494
577.30000	35.0	Cluster_10500
577.81000	35.0	Cluster_30446
578.32000	35.0	Cluster_30460
578.55000	35.0	Cluster_02454
578.86000	35.0	Cluster_30486
578.98000	35.0	Cluster_10523
579.33000	35.0	Cluster_30494
579.55000	35.0	Cluster_02460
579.61000	35.0	Cluster_10531
579.63000	35.0	Cluster_10533
579.77000	35.0	Cluster_30512
579.81000	35.0	Cluster_30517
579.83000	35.0	Cluster_30515
580.36000	35.0	Cluster_30555
580.86000	35.0	Cluster_02466
581.28000	35.0	Cluster_02468
581.31000	35.0	Cluster_30577
581.96000	35.0	Cluster_10563
582.31000	35.0	Cluster_30648
582.54000	35.0	Cluster_02478
582.65000	35.0	Cluster_10574
582.70000	35.0	Cluster_00226
582.74000	35.0	Cluster_30654
584.05000	35.0	Cluster_30718
584.31000	35.0	Cluster_30724
584.54000	35.0	Cluster_30733
585.02000	35.0	Cluster_30763
585.29000	35.0	Cluster_10612
585.35000	35.0	Cluster_02501

585.80000	35.0	Cluster_30791
585.84000	35.0	Cluster_30792
586.62000	35.0	Cluster_10630
586.75000	35.0	Cluster_30815
587.30000	35.0	Cluster_10642
587.33000	35.0	Cluster_30848
587.34000	35.0	Cluster_30837
587.65000	35.0	Cluster_10646
588.69000	35.0	Cluster_30892
588.82000	35.0	Cluster_30900
589.55000	35.0	Cluster_30932
589.74000	35.0	Cluster_30933
589.96000	35.0	Cluster_10677
590.22000	35.0	Cluster_30952
590.30000	35.0	Cluster_30953
591.04000	35.0	Cluster_30998
591.44000	35.0	Cluster_31008
591.52000	35.0	Cluster_02538
591.66000	35.0	Cluster_10703
591.81000	35.0	Cluster_31027
592.01000	35.0	Cluster_10706
592.79000	35.0	Cluster_31056
592.82000	35.0	Cluster_31060
592.91000	35.0	Cluster_31063
593.06000	35.0	Cluster_02547
593.07000	35.0	Cluster_02548
593.33000	35.0	Cluster_10724
593.34000	35.0	Cluster_31073
593.93000	35.0	Cluster_10743
594.32000	35.0	Cluster_10753
595.59000	35.0	Cluster_31146
595.62000	35.0	Cluster_10769
596.75000	35.0	Cluster_31177
597.67000	35.0	Cluster_10808
597.81000	35.0	Cluster_31212
597.82000	35.0	Cluster_31208
598.29000	35.0	Cluster_10814
598.36000	35.0	Cluster_10818
599.36000	35.0	Cluster_31267
599.66000	35.0	Cluster_10840
599.95000	35.0	Cluster_10848
600.81000	35.0	Cluster_31316
600.97000	35.0	Cluster_10858
601.36000	35.0	Cluster_31339
602.32000	35.0	Cluster_31390
602.83000	35.0	Cluster_31406
603.30000	35.0	Cluster_31409
603.51000	35.0	Cluster_02598
603.53000	35.0	Cluster_31419
603.96000	35.0	Cluster_10888
604.80000	35.0	Cluster_31463

605.31000	35.0	Cluster_31473
605.67000	35.0	Cluster_10910
606.07000	35.0	Cluster_31501
606.27000	35.0	Cluster_31514
606.87000	35.0	Cluster_31531
606.89000	35.0	Cluster_10941
607.32000	35.0	Cluster_31536
608.09000	35.0	Cluster_02626
608.32000	35.0	Cluster_31587
608.49000	35.0	Cluster_31590
608.78000	35.0	Cluster_31595
608.79000	35.0	Cluster_31601
609.06000	35.0	Cluster_31605
609.81000	35.0	Cluster_31625
610.31000	35.0	Cluster_31655
611.34000	35.0	Cluster_31685
612.32000	35.0	Cluster_11010
613.83000	35.0	Cluster_31794
614.06000	35.0	Cluster_31796
614.07000	35.0	Cluster_02670
614.19000	35.0	Cluster_31797
614.26000	35.0	Cluster_11035
615.83000	35.0	Cluster_31852
616.08000	35.0	Cluster_02680
616.32000	35.0	Cluster_11075
616.55000	35.0	Cluster_31873
616.83000	35.0	Cluster_31882
617.02000	35.0	Cluster_31892
617.35000	35.0	Cluster_31911
617.93000	35.0	Cluster_31935
618.05000	35.0	Cluster_02693
618.79000	35.0	Cluster_31952
619.29000	35.0	Cluster_02701
619.33000	35.0	Cluster_31985
619.62000	35.0	Cluster_11116
620.53000	35.0	Cluster_32028
620.60000	35.0	Cluster_11128
621.30000	35.0	Cluster_32049
621.36000	35.0	Cluster_32053
621.39000	35.0	Cluster_32052
621.79000	35.0	Cluster_32057
621.98000	35.0	Cluster_11154
622.78000	35.0	Cluster_32095
622.98000	35.0	Cluster_11175
623.28000	35.0	Cluster_32112
623.36000	35.0	Cluster_32126
623.65000	35.0	Cluster_11191
623.98000	35.0	Cluster_11189
624.20000	35.0	Cluster_32153
624.28000	35.0	Cluster_32154
624.63000	35.0	Cluster_11202

625.04000	35.0	Cluster_02732
625.32000	35.0	Cluster_11203
625.58000	35.0	Cluster_02735
626.00000	35.0	Cluster_11225
626.07000	35.0	Cluster_32208
626.27000	35.0	Cluster_32218
626.28000	35.0	Cluster_32212
626.83000	35.0	Cluster_32244
627.30000	35.0	Cluster_32266
627.31000	35.0	Cluster_32263
627.50000	35.0	Cluster_32275
627.79000	35.0	Cluster_32283
628.07000	35.0	Cluster_32292
628.86000	35.0	Cluster_32321
629.63000	35.0	Cluster_11289
629.83000	35.0	Cluster_32344
629.96000	35.0	Cluster_11292
630.84000	35.0	Cluster_32375
631.37000	35.0	Cluster_11312
631.80000	35.0	Cluster_32410
632.05000	35.0	Cluster_11324
632.35000	35.0	Cluster_11329
632.78000	35.0	Cluster_32445
632.84000	35.0	Cluster_32450
633.00000	35.0	Cluster_11339
633.32000	35.0	Cluster_32465
633.36000	35.0	Cluster_32472
635.07000	35.0	Cluster_02772
635.82000	35.0	Cluster_02775
636.32000	35.0	Cluster_32584
636.84000	35.0	Cluster_32596
637.62000	35.0	Cluster_11403
637.83000	35.0	Cluster_32632
638.35000	35.0	Cluster_32655
638.36000	35.0	Cluster_11416
638.39000	35.0	Cluster_32659
638.79000	35.0	Cluster_32669
638.85000	35.0	Cluster_32673
639.85000	35.0	Cluster_32724
639.99000	35.0	Cluster_11446
640.32000	35.0	Cluster_32732
641.64000	35.0	Cluster_11472
641.84000	35.0	Cluster_32794
642.02000	35.0	Cluster_11476
643.07000	35.0	Cluster_32838
643.83000	35.0	Cluster_32863
644.00000	35.0	Cluster_11510
644.30000	35.0	Cluster_02836
644.36000	35.0	Cluster_32878
644.37000	35.0	Cluster_11511
645.00000	35.0	Cluster_11529

645.85000	35.0	Cluster_32926
646.30000	35.0	Cluster_32945
648.34000	35.0	Cluster_33005
648.67000	35.0	Cluster_11566
648.88000	35.0	Cluster_33029
649.01000	35.0	Cluster_11574
649.77000	35.0	Cluster_33054
650.31000	35.0	Cluster_33085
651.01000	35.0	Cluster_11595
651.08000	35.0	Cluster_33114
651.32000	35.0	Cluster_33124
651.36000	35.0	Cluster_11600
651.86000	35.0	Cluster_33158
651.99000	35.0	Cluster_11614
652.06000	35.0	Cluster_33159
652.35000	35.0	Cluster_33162
652.86000	35.0	Cluster_33187
653.33000	35.0	Cluster_33199
653.57000	35.0	Cluster_33207
653.60000	35.0	Cluster_02883
654.05000	35.0	Cluster_33221
654.68000	35.0	Cluster_11655
654.78000	35.0	Cluster_33237
655.99000	35.0	Cluster_11675
656.63000	35.0	Cluster_11676
657.90000	35.0	Cluster_33331
657.95000	35.0	Cluster_33350
658.07000	35.0	Cluster_33340
658.66000	35.0	Cluster_11699
659.28000	35.0	Cluster_33386
659.60000	35.0	Cluster_33396
659.63000	35.0	Cluster_02907
659.98000	35.0	Cluster_11725
660.33000	35.0	Cluster_33416
660.46000	35.0	Cluster_33423
660.84000	35.0	Cluster_33430
661.01000	35.0	Cluster_11741
661.31000	35.0	Cluster_33436
662.02000	35.0	Cluster_11748
662.12000	35.0	Cluster_02928
662.30000	35.0	Cluster_33472
663.37000	35.0	Cluster_33509
663.55000	35.0	Cluster_02941
663.60000	35.0	Cluster_02940
665.32000	35.0	Cluster_33570
666.35000	35.0	Cluster_02964
666.68000	35.0	Cluster_11814
666.95000	35.0	Cluster_33617
667.82000	35.0	Cluster_33644
668.33000	35.0	Cluster_33651
668.35000	35.0	Cluster_11848

669.10000	35.0	Cluster_02977
669.33000	35.0	Cluster_33680
669.78000	35.0	Cluster_33695
670.01000	35.0	Cluster_11876
670.40000	35.0	Cluster_33725
670.80000	35.0	Cluster_33734
670.82000	35.0	Cluster_33742
670.87000	35.0	Cluster_02986
671.44000	35.0	Cluster_33760
671.82000	35.0	Cluster_33770
671.97000	35.0	Cluster_11894
672.35000	35.0	Cluster_33781
672.81000	35.0	Cluster_33789
673.09000	35.0	Cluster_03005
673.37000	35.0	Cluster_33802
673.62000	35.0	Cluster_33824
673.65000	35.0	Cluster_11927
674.11000	35.0	Cluster_03014
674.32000	35.0	Cluster_11933
674.95000	35.0	Cluster_00274
675.37000	35.0	Cluster_33861
675.60000	35.0	Cluster_33864
675.64000	35.0	Cluster_03025
675.84000	35.0	Cluster_03026
676.00000	35.0	Cluster_11956
676.32000	35.0	Cluster_11959
677.20000	35.0	Cluster_33905
677.89000	35.0	Cluster_33946
678.81000	35.0	Cluster_33981
678.84000	35.0	Cluster_33980
678.85000	35.0	Cluster_03050
679.38000	35.0	Cluster_33996
679.79000	35.0	Cluster_34007
679.86000	35.0	Cluster_34018
680.08000	35.0	Cluster_03060
680.09000	35.0	Cluster_34023
680.87000	35.0	Cluster_34054
681.08000	35.0	Cluster_03066
681.32000	35.0	Cluster_12020
681.35000	35.0	Cluster_34074
682.09000	35.0	Cluster_34089
682.31000	35.0	Cluster_34106
682.38000	35.0	Cluster_03074
683.61000	35.0	Cluster_12059
683.81000	35.0	Cluster_34134
683.99000	35.0	Cluster_12062
684.08000	35.0	Cluster_03088
684.82000	35.0	Cluster_34171
684.83000	35.0	Cluster_34174
685.09000	35.0	Cluster_03095
685.10000	35.0	Cluster_03102

685.35000	35.0	Cluster_34201
686.04000	35.0	Cluster_12086
686.50000	35.0	Cluster_00285
686.75000	35.0	Cluster_00286
687.66000	35.0	Cluster_34255
689.34000	35.0	Cluster_34298
689.36000	35.0	Cluster_34295
689.57000	35.0	Cluster_03142
690.82000	35.0	Cluster_34324
690.84000	35.0	Cluster_34327
691.59000	35.0	Cluster_03162
691.79000	35.0	Cluster_34357
691.83000	35.0	Cluster_34369
692.26000	35.0	Cluster_34373
692.32000	35.0	Cluster_03167
692.35000	35.0	Cluster_03168
692.60000	35.0	Cluster_03182
692.84000	35.0	Cluster_34402
693.56000	35.0	Cluster_03192
693.60000	35.0	Cluster_34413
693.88000	35.0	Cluster_34418
694.36000	35.0	Cluster_34424
694.84000	35.0	Cluster_34436
694.87000	35.0	Cluster_34443
695.01000	35.0	Cluster_12183
695.36000	35.0	Cluster_34458
695.70000	35.0	Cluster_12190
695.87000	35.0	Cluster_34468
696.32000	35.0	Cluster_34489
696.34000	35.0	Cluster_03210
696.39000	35.0	Cluster_34483
696.58000	35.0	Cluster_03211
697.31000	35.0	Cluster_03215
697.37000	35.0	Cluster_34515
697.85000	35.0	Cluster_34520
697.87000	35.0	Cluster_34523
698.82000	35.0	Cluster_34541
698.83000	35.0	Cluster_34546
699.70000	35.0	Cluster_12244
699.85000	35.0	Cluster_03235
700.26000	35.0	Cluster_34580
700.36000	35.0	Cluster_34589
700.65000	35.0	Cluster_34590
700.85000	35.0	Cluster_34594
701.36000	35.0	Cluster_34614
702.34000	35.0	Cluster_34653
702.62000	35.0	Cluster_12286
703.04000	35.0	Cluster_12291
703.05000	35.0	Cluster_03266
703.33000	35.0	Cluster_34677
703.69000	35.0	Cluster_12310

703.91000	35.0	Cluster_03274
704.17000	35.0	Cluster_34698
704.21000	35.0	Cluster_34700
704.31000	35.0	Cluster_34706
704.81000	35.0	Cluster_34728
704.85000	35.0	Cluster_34734
705.04000	35.0	Cluster_12319
705.37000	35.0	Cluster_34742
705.83000	35.0	Cluster_34756
705.88000	35.0	Cluster_34757
706.86000	35.0	Cluster_03301
707.02000	35.0	Cluster_12346
707.80000	35.0	Cluster_34791
708.51000	35.0	Cluster_00309
709.28000	35.0	Cluster_34840
709.39000	35.0	Cluster_34846
710.88000	35.0	Cluster_03335
711.06000	35.0	Cluster_12397
711.85000	35.0	Cluster_34925
712.39000	35.0	Cluster_34948
712.84000	35.0	Cluster_34967
713.08000	35.0	Cluster_03351
713.29000	35.0	Cluster_03356
713.38000	35.0	Cluster_12431
713.72000	35.0	Cluster_12437
713.77000	35.0	Cluster_34987
713.92000	35.0	Cluster_34998
714.32000	35.0	Cluster_35004
714.36000	35.0	Cluster_35010
715.38000	35.0	Cluster_35041
715.54000	35.0	Cluster_00320
715.71000	35.0	Cluster_12456
715.85000	35.0	Cluster_35046
715.99000	35.0	Cluster_12465
716.39000	35.0	Cluster_35061
717.36000	35.0	Cluster_12478
717.90000	35.0	Cluster_35102
718.36000	35.0	Cluster_35122
718.85000	35.0	Cluster_35127
718.89000	35.0	Cluster_35137
718.91000	35.0	Cluster_35135
718.96000	35.0	Cluster_12511
719.39000	35.0	Cluster_35145
719.83000	35.0	Cluster_35161
719.85000	35.0	Cluster_03411
719.98000	35.0	Cluster_12519
720.12000	35.0	Cluster_35168
720.13000	35.0	Cluster_35170
720.35000	35.0	Cluster_03417
720.58000	35.0	Cluster_03421
720.83000	35.0	Cluster_35178

721.32000	35.0	Cluster_35195
721.41000	35.0	Cluster_35191
721.72000	35.0	Cluster_12548
721.88000	35.0	Cluster_35209
721.90000	35.0	Cluster_35213
722.02000	35.0	Cluster_12553
722.09000	35.0	Cluster_03436
722.34000	35.0	Cluster_03433
722.68000	35.0	Cluster_12565
723.40000	35.0	Cluster_12568
723.42000	35.0	Cluster_12570
723.95000	35.0	Cluster_00326
724.04000	35.0	Cluster_12575
724.90000	35.0	Cluster_03465
725.06000	35.0	Cluster_12593
725.32000	35.0	Cluster_03469
725.41000	35.0	Cluster_35286
725.42000	35.0	Cluster_35293
727.38000	35.0	Cluster_35317
728.10000	35.0	Cluster_03487
728.13000	35.0	Cluster_03484
728.42000	35.0	Cluster_35338
728.98000	35.0	Cluster_12653
729.15000	35.0	Cluster_03497
729.84000	35.0	Cluster_35373
729.89000	35.0	Cluster_03507
730.91000	35.0	Cluster_35408
731.10000	35.0	Cluster_35410
731.83000	35.0	Cluster_03518
732.11000	35.0	Cluster_03522
732.64000	35.0	Cluster_03534
733.03000	35.0	Cluster_12708
733.13000	35.0	Cluster_03548
733.83000	35.0	Cluster_35471
733.85000	35.0	Cluster_35474
734.58000	35.0	Cluster_35493
735.04000	35.0	Cluster_12729
735.74000	35.0	Cluster_12741
736.37000	35.0	Cluster_35541
737.04000	35.0	Cluster_12754
737.16000	35.0	Cluster_35560
737.82000	35.0	Cluster_35593
738.39000	35.0	Cluster_35611
738.63000	35.0	Cluster_03590
739.38000	35.0	Cluster_12791
739.39000	35.0	Cluster_12789
739.84000	35.0	Cluster_35645
740.37000	35.0	Cluster_35657
740.38000	35.0	Cluster_35661
740.93000	35.0	Cluster_35676
741.85000	35.0	Cluster_35683

742.37000	35.0	Cluster_35697
742.67000	35.0	Cluster_12831
742.84000	35.0	Cluster_35707
744.37000	35.0	Cluster_35750
745.38000	35.0	Cluster_35777
745.68000	35.0	Cluster_12857
746.06000	35.0	Cluster_12863
746.15000	35.0	Cluster_35792
746.30000	35.0	Cluster_35797
747.12000	35.0	Cluster_35817
747.37000	35.0	Cluster_35818
748.00000	35.0	Cluster_12903
748.86000	35.0	Cluster_03654
748.88000	35.0	Cluster_35857
749.07000	35.0	Cluster_12920
749.69000	35.0	Cluster_12929
749.98000	35.0	Cluster_12936
750.01000	35.0	Cluster_12941
750.38000	35.0	Cluster_35883
750.44000	35.0	Cluster_35886
750.94000	35.0	Cluster_35903
751.06000	35.0	Cluster_12961
751.15000	35.0	Cluster_03662
751.36000	35.0	Cluster_35908
752.37000	35.0	Cluster_35932
753.05000	35.0	Cluster_12988
753.06000	35.0	Cluster_12984
753.39000	35.0	Cluster_35964
753.91000	35.0	Cluster_35981
754.06000	35.0	Cluster_13009
754.90000	35.0	Cluster_36004
755.06000	35.0	Cluster_13021
755.30000	35.0	Cluster_13019
755.33000	35.0	Cluster_13020
755.42000	35.0	Cluster_36013
755.75000	35.0	Cluster_13034
755.92000	35.0	Cluster_36043
756.13000	35.0	Cluster_03688
757.31000	35.0	Cluster_36071
757.73000	35.0	Cluster_13076
758.41000	35.0	Cluster_36102
758.70000	35.0	Cluster_13088
759.63000	35.0	Cluster_03704
759.90000	35.0	Cluster_36129
759.91000	35.0	Cluster_36132
760.35000	35.0	Cluster_36146
761.05000	35.0	Cluster_00024
761.41000	35.0	Cluster_36182
762.38000	35.0	Cluster_13130
763.03000	35.0	Cluster_13141
763.08000	35.0	Cluster_13137

763.35000	35.0	Cluster_03726
763.71000	35.0	Cluster_13147
764.09000	35.0	Cluster_13148
764.36000	35.0	Cluster_36253
765.15000	35.0	Cluster_03741
765.79000	35.0	Cluster_36274
765.80000	35.0	Cluster_36275
766.37000	35.0	Cluster_36292
766.55000	35.0	Cluster_00339
767.70000	35.0	Cluster_13203
767.85000	35.0	Cluster_36325
768.07000	35.0	Cluster_13209
768.08000	35.0	Cluster_13205
768.38000	35.0	Cluster_36351
769.33000	35.0	Cluster_36370
769.59000	35.0	Cluster_36368
769.82000	35.0	Cluster_36385
772.41000	35.0	Cluster_36441
772.74000	35.0	Cluster_13265
772.80000	35.0	Cluster_13273
772.88000	35.0	Cluster_36459
772.92000	35.0	Cluster_36460
773.08000	35.0	Cluster_03785
773.09000	35.0	Cluster_03786
773.13000	35.0	Cluster_13272
773.35000	35.0	Cluster_13283
773.41000	35.0	Cluster_13286
774.12000	35.0	Cluster_36481
774.73000	35.0	Cluster_13306
775.06000	35.0	Cluster_13312
775.07000	35.0	Cluster_13311
775.42000	35.0	Cluster_36510
776.37000	35.0	Cluster_03803
777.15000	35.0	Cluster_03806
777.35000	35.0	Cluster_36561
777.38000	35.0	Cluster_36567
777.39000	35.0	Cluster_36564
778.37000	35.0	Cluster_36591
778.77000	35.0	Cluster_13350
778.87000	35.0	Cluster_36605
780.73000	35.0	Cluster_13379
780.81000	35.0	Cluster_36636
780.88000	35.0	Cluster_03821
781.40000	35.0	Cluster_36661
782.03000	35.0	Cluster_13403
783.13000	35.0	Cluster_03834
783.46000	35.0	Cluster_36708
783.72000	35.0	Cluster_13422
784.06000	35.0	Cluster_13433
784.89000	35.0	Cluster_36733
784.93000	35.0	Cluster_36732

785.45000	35.0	Cluster_36744
785.83000	35.0	Cluster_36763
787.06000	35.0	Cluster_13459
787.12000	35.0	Cluster_03868
787.35000	35.0	Cluster_03870
788.87000	35.0	Cluster_36840
789.06000	35.0	Cluster_13483
790.87000	35.0	Cluster_36906
791.12000	35.0	Cluster_36909
791.38000	35.0	Cluster_36914
791.90000	35.0	Cluster_36919
792.38000	35.0	Cluster_36933
792.64000	35.0	Cluster_36941
792.86000	35.0	Cluster_03914
792.87000	35.0	Cluster_36948
792.97000	35.0	Cluster_13541
793.78000	35.0	Cluster_13547
794.36000	35.0	Cluster_36984
794.37000	35.0	Cluster_36982
794.45000	35.0	Cluster_36985
794.65000	35.0	Cluster_36986
795.07000	35.0	Cluster_13571
795.42000	35.0	Cluster_37004
795.44000	35.0	Cluster_37000
795.68000	35.0	Cluster_13585
795.87000	35.0	Cluster_03947
795.90000	35.0	Cluster_37017
795.96000	35.0	Cluster_37020
796.41000	35.0	Cluster_13605
796.72000	35.0	Cluster_13597
796.91000	35.0	Cluster_37044
797.86000	35.0	Cluster_37077
797.87000	35.0	Cluster_37073
797.88000	35.0	Cluster_37072
797.89000	35.0	Cluster_37068
799.38000	35.0	Cluster_37115
799.40000	35.0	Cluster_37112
799.89000	35.0	Cluster_03975
800.35000	35.0	Cluster_37146
800.41000	35.0	Cluster_13643
800.84000	35.0	Cluster_37149
800.86000	35.0	Cluster_03991
801.06000	35.0	Cluster_00029
801.37000	35.0	Cluster_03995
801.42000	35.0	Cluster_37163
801.44000	35.0	Cluster_13660
802.43000	35.0	Cluster_37188
802.74000	35.0	Cluster_13681
803.41000	35.0	Cluster_13684
803.88000	35.0	Cluster_37226
804.43000	35.0	Cluster_37243

804.91000	35.0	Cluster_04015
805.16000	35.0	Cluster_37269
805.39000	35.0	Cluster_37283
805.94000	35.0	Cluster_37284
806.39000	35.0	Cluster_37304
807.01000	35.0	Cluster_13723
807.46000	35.0	Cluster_37330
807.92000	35.0	Cluster_37352
808.85000	35.0	Cluster_37370
808.91000	35.0	Cluster_37377
809.40000	35.0	Cluster_04037
809.91000	35.0	Cluster_37401
810.39000	35.0	Cluster_37407
810.44000	35.0	Cluster_13774
811.74000	35.0	Cluster_00036
811.86000	35.0	Cluster_37446
811.91000	35.0	Cluster_37441
812.39000	35.0	Cluster_37458
812.41000	35.0	Cluster_13796
813.82000	35.0	Cluster_00352
813.90000	35.0	Cluster_37483
814.10000	35.0	Cluster_13827
814.16000	35.0	Cluster_37492
814.17000	35.0	Cluster_04067
814.90000	35.0	Cluster_04074
815.06000	35.0	Cluster_13832
815.79000	35.0	Cluster_13844
815.92000	35.0	Cluster_37530
816.38000	35.0	Cluster_37540
816.43000	35.0	Cluster_04088
816.72000	35.0	Cluster_13857
817.34000	35.0	Cluster_37561
817.88000	35.0	Cluster_37570
818.42000	35.0	Cluster_13883
819.05000	35.0	Cluster_13897
819.42000	35.0	Cluster_13906
820.17000	35.0	Cluster_04104
821.23000	35.0	Cluster_04111
821.38000	35.0	Cluster_37637
822.06000	35.0	Cluster_13934
823.42000	35.0	Cluster_37669
823.91000	35.0	Cluster_37674
823.94000	35.0	Cluster_37688
825.32000	35.0	Cluster_37717
825.39000	35.0	Cluster_04137
826.38000	35.0	Cluster_13981
826.86000	35.0	Cluster_37765
827.04000	35.0	Cluster_13986
827.06000	35.0	Cluster_13988
827.09000	35.0	Cluster_13989
827.88000	35.0	Cluster_37794

828.80000	35.0	Cluster_14000
828.98000	35.0	Cluster_14004
829.16000	35.0	Cluster_04165
829.36000	35.0	Cluster_37821
829.43000	35.0	Cluster_37825
829.91000	35.0	Cluster_04169
830.74000	35.0	Cluster_14020
830.89000	35.0	Cluster_00042
831.45000	35.0	Cluster_37853
832.41000	35.0	Cluster_37873
832.68000	35.0	Cluster_37879
833.49000	35.0	Cluster_37894
833.99000	35.0	Cluster_37902
834.10000	35.0	Cluster_14054
834.94000	35.0	Cluster_37918
835.05000	35.0	Cluster_14062
836.39000	35.0	Cluster_14076
836.42000	35.0	Cluster_14077
836.76000	35.0	Cluster_14083
836.86000	35.0	Cluster_37959
836.94000	35.0	Cluster_04234
837.41000	35.0	Cluster_37970
837.95000	35.0	Cluster_37976
839.10000	35.0	Cluster_14102
840.66000	35.0	Cluster_04260
840.78000	35.0	Cluster_14117
841.08000	35.0	Cluster_14128
841.10000	35.0	Cluster_14126
841.67000	35.0	Cluster_00364
841.76000	35.0	Cluster_38066
842.73000	35.0	Cluster_14140
843.87000	35.0	Cluster_38114
844.18000	35.0	Cluster_04298
844.44000	35.0	Cluster_14157
846.98000	35.0	Cluster_38180
847.48000	35.0	Cluster_38200
847.75000	35.0	Cluster_14185
847.88000	35.0	Cluster_38203
848.40000	35.0	Cluster_04350
848.91000	35.0	Cluster_04352
849.46000	35.0	Cluster_38235
849.64000	35.0	Cluster_04364
849.97000	35.0	Cluster_38245
849.99000	35.0	Cluster_38248
850.33000	35.0	Cluster_38250
850.40000	35.0	Cluster_04376
850.76000	35.0	Cluster_14213
851.36000	35.0	Cluster_14215
851.77000	35.0	Cluster_38298
852.03000	35.0	Cluster_38307
852.91000	35.0	Cluster_04400

852.93000	35.0	Cluster_04401
853.45000	35.0	Cluster_38334
854.44000	35.0	Cluster_38377
854.47000	35.0	Cluster_38375
854.88000	35.0	Cluster_38385
855.41000	35.0	Cluster_38392
855.73000	35.0	Cluster_14240
856.43000	35.0	Cluster_14249
856.91000	35.0	Cluster_04444
857.06000	35.0	Cluster_14257
857.89000	35.0	Cluster_38459
857.91000	35.0	Cluster_04449
858.07000	35.0	Cluster_14269
858.77000	35.0	Cluster_14275
859.45000	35.0	Cluster_38500
859.47000	35.0	Cluster_38497
860.47000	35.0	Cluster_14284
861.39000	35.0	Cluster_04493
862.39000	35.0	Cluster_04505
862.49000	35.0	Cluster_38591
863.05000	35.0	Cluster_14302
863.76000	35.0	Cluster_14307
863.94000	35.0	Cluster_38615
864.07000	35.0	Cluster_14314
865.03000	35.0	Cluster_38646
865.75000	35.0	Cluster_14326
865.92000	35.0	Cluster_38659
865.93000	35.0	Cluster_04555
866.41000	35.0	Cluster_14334
869.07000	35.0	Cluster_14353
869.28000	35.0	Cluster_38719
869.41000	35.0	Cluster_04599
869.78000	35.0	Cluster_14363
871.17000	35.0	Cluster_38758
871.27000	35.0	Cluster_38759
871.44000	35.0	Cluster_38769
871.47000	35.0	Cluster_14388
872.27000	35.0	Cluster_38802
874.09000	35.0	Cluster_14414
874.26000	35.0	Cluster_38844
874.78000	35.0	Cluster_14424
875.28000	35.0	Cluster_38860
875.66000	35.0	Cluster_04698
876.08000	35.0	Cluster_14437
876.53000	35.0	Cluster_38895
877.38000	35.0	Cluster_14453
877.44000	35.0	Cluster_38909
878.48000	35.0	Cluster_38928
880.46000	35.0	Cluster_14483
881.96000	35.0	Cluster_38993
882.44000	35.0	Cluster_39008

882.94000	35.0	Cluster_39018
884.40000	35.0	Cluster_14528
884.66000	35.0	Cluster_39047
885.12000	35.0	Cluster_14544
885.68000	35.0	Cluster_04765
885.95000	35.0	Cluster_39078
886.43000	35.0	Cluster_39095
886.96000	35.0	Cluster_39099
887.52000	35.0	Cluster_39107
887.68000	35.0	Cluster_04781
887.74000	35.0	Cluster_14571
888.13000	35.0	Cluster_14577
888.40000	35.0	Cluster_14578
888.91000	35.0	Cluster_39135
888.92000	35.0	Cluster_04786
889.11000	35.0	Cluster_14581
889.44000	35.0	Cluster_39147
890.17000	35.0	Cluster_04796
890.38000	35.0	Cluster_39161
890.39000	35.0	Cluster_39160
890.46000	35.0	Cluster_39173
890.99000	35.0	Cluster_39180
891.46000	35.0	Cluster_39186
891.81000	35.0	Cluster_14598
892.21000	35.0	Cluster_04824
893.43000	35.0	Cluster_39231
893.78000	35.0	Cluster_14615
894.12000	35.0	Cluster_14617
894.71000	35.0	Cluster_04845
895.21000	35.0	Cluster_04851
895.45000	35.0	Cluster_14622
895.81000	35.0	Cluster_14625
896.15000	35.0	Cluster_14634
897.04000	35.0	Cluster_39292
898.77000	35.0	Cluster_14664
899.43000	35.0	Cluster_39337
899.51000	35.0	Cluster_39345
899.96000	35.0	Cluster_04891
900.94000	35.0	Cluster_39368
900.97000	35.0	Cluster_39380
900.99000	35.0	Cluster_39377
902.46000	35.0	Cluster_39412
904.46000	35.0	Cluster_39443
905.43000	35.0	Cluster_39461
906.77000	35.0	Cluster_14739
907.80000	35.0	Cluster_14749
908.46000	35.0	Cluster_39500
908.48000	35.0	Cluster_14756
908.94000	35.0	Cluster_04958
908.95000	35.0	Cluster_39504
909.44000	35.0	Cluster_14760

909.50000	35.0	Cluster_14765
909.89000	35.0	Cluster_39520
910.11000	35.0	Cluster_14769
910.19000	35.0	Cluster_04966
911.96000	35.0	Cluster_39564
914.56000	35.0	Cluster_39615
915.13000	35.0	Cluster_14823
915.93000	35.0	Cluster_39626
916.44000	35.0	Cluster_39640
916.49000	35.0	Cluster_39633
917.45000	35.0	Cluster_14854
917.97000	35.0	Cluster_39674
918.66000	35.0	Cluster_39698
920.45000	35.0	Cluster_39721
920.76000	35.0	Cluster_14891
920.79000	35.0	Cluster_39725
920.88000	35.0	Cluster_39726
920.95000	35.0	Cluster_39731
922.42000	35.0	Cluster_14907
922.98000	35.0	Cluster_39772
923.30000	35.0	Cluster_14924
923.45000	35.0	Cluster_14932
923.92000	35.0	Cluster_39782
924.49000	35.0	Cluster_39788
925.11000	35.0	Cluster_14961
926.42000	35.0	Cluster_14975
927.48000	35.0	Cluster_39834
928.47000	35.0	Cluster_39858
928.96000	35.0	Cluster_39861
929.12000	35.0	Cluster_15009
929.43000	35.0	Cluster_39880
929.90000	35.0	Cluster_39897
930.47000	35.0	Cluster_15020
931.73000	35.0	Cluster_15043
931.90000	35.0	Cluster_39924
932.64000	35.0	Cluster_15062
932.95000	35.0	Cluster_39941
933.14000	35.0	Cluster_15072
933.33000	35.0	Cluster_05038
934.00000	35.0	Cluster_39963
934.13000	35.0	Cluster_15084
934.43000	35.0	Cluster_15083
934.78000	35.0	Cluster_15089
935.78000	35.0	Cluster_15100
936.45000	35.0	Cluster_15116
936.80000	35.0	Cluster_15129
937.00000	35.0	Cluster_39997
937.44000	35.0	Cluster_15144
937.49000	35.0	Cluster_05051
938.07000	35.0	Cluster_15154
938.13000	35.0	Cluster_15156

938.21000	35.0	Cluster_15160
939.69000	35.0	Cluster_05058
940.46000	35.0	Cluster_15180
941.72000	35.0	Cluster_40098
942.00000	35.0	Cluster_40094
943.45000	35.0	Cluster_15229
945.13000	35.0	Cluster_15244
945.39000	35.0	Cluster_15242
945.72000	35.0	Cluster_05075
945.73000	35.0	Cluster_05078
945.78000	35.0	Cluster_15250
945.98000	35.0	Cluster_40152
947.16000	35.0	Cluster_15264
947.22000	35.0	Cluster_05084
947.79000	35.0	Cluster_15273
948.19000	35.0	Cluster_15277
948.52000	35.0	Cluster_40180
949.14000	35.0	Cluster_15301
949.44000	35.0	Cluster_40190
949.46000	35.0	Cluster_15302
950.44000	35.0	Cluster_15314
951.17000	35.0	Cluster_15324
951.96000	35.0	Cluster_40227
952.14000	35.0	Cluster_15337
952.15000	35.0	Cluster_15339
952.44000	35.0	Cluster_15342
952.53000	35.0	Cluster_40233
953.98000	35.0	Cluster_40255
954.82000	35.0	Cluster_15379
954.99000	35.0	Cluster_40262
955.13000	35.0	Cluster_15394
955.47000	35.0	Cluster_15404
956.48000	35.0	Cluster_15426
957.07000	35.0	Cluster_40290
957.48000	35.0	Cluster_40291
960.15000	35.0	Cluster_15462
960.98000	35.0	Cluster_40317
961.48000	35.0	Cluster_15478
962.14000	35.0	Cluster_15497
962.38000	35.0	Cluster_40344
962.79000	35.0	Cluster_15505
964.13000	35.0	Cluster_15523
964.43000	35.0	Cluster_40365
965.29000	35.0	Cluster_40377
966.15000	35.0	Cluster_15549
966.49000	35.0	Cluster_40387
966.50000	35.0	Cluster_05172
966.85000	35.0	Cluster_15552
967.00000	35.0	Cluster_40392
967.56000	35.0	Cluster_15565
968.03000	35.0	Cluster_40402

968.25000	35.0	Cluster_05187
971.97000	35.0	Cluster_40451
972.01000	35.0	Cluster_40467
972.46000	35.0	Cluster_15627
972.49000	35.0	Cluster_15626
973.51000	35.0	Cluster_40476
974.47000	35.0	Cluster_15651
974.98000	35.0	Cluster_40495
975.82000	35.0	Cluster_15672
975.98000	35.0	Cluster_05210
976.00000	35.0	Cluster_05212
976.03000	35.0	Cluster_40504
977.14000	35.0	Cluster_15696
977.62000	35.0	Cluster_40522
979.45000	35.0	Cluster_40546
979.80000	35.0	Cluster_15720
979.83000	35.0	Cluster_15721
979.98000	35.0	Cluster_40568
980.00000	35.0	Cluster_40562
980.50000	35.0	Cluster_40565
980.70000	35.0	Cluster_40578
981.45000	35.0	Cluster_15750
983.25000	35.0	Cluster_05237
983.95000	35.0	Cluster_40617
985.14000	35.0	Cluster_40628
986.02000	35.0	Cluster_40630
986.52000	35.0	Cluster_40639
986.83000	35.0	Cluster_15810
987.24000	35.0	Cluster_40654
987.80000	35.0	Cluster_15825
988.44000	35.0	Cluster_15835
991.93000	35.0	Cluster_40717
993.50000	35.0	Cluster_15895
997.19000	35.0	Cluster_15954
1000.06000	35.0	Cluster_40851
1001.03000	35.0	Cluster_40878
1001.16000	35.0	Cluster_15996
1001.49000	35.0	Cluster_40884
1003.52000	35.0	Cluster_05294
1004.00000	35.0	Cluster_40937
1004.31000	35.0	Cluster_05296
1004.57000	35.0	Cluster_40940
1007.81000	35.0	Cluster_16072
1008.78000	35.0	Cluster_16080
1012.26000	35.0	Cluster_05329
1012.27000	35.0	Cluster_41044
1012.93000	35.0	Cluster_41054
1013.01000	35.0	Cluster_05341
1013.47000	35.0	Cluster_16138
1013.49000	35.0	Cluster_16137
1013.76000	35.0	Cluster_05344

1013.90000	35.0	Cluster_41056
1015.92000	35.0	Cluster_41088
1016.78000	35.0	Cluster_05351
1017.27000	35.0	Cluster_41099
1019.25000	35.0	Cluster_05360
1020.15000	35.0	Cluster_16210
1022.50000	35.0	Cluster_16231
1023.82000	35.0	Cluster_41185
1024.48000	35.0	Cluster_16254
1024.50000	35.0	Cluster_16256
1024.93000	35.0	Cluster_41195
1025.46000	35.0	Cluster_16264
1025.81000	35.0	Cluster_41211
1026.83000	35.0	Cluster_16274
1027.13000	35.0	Cluster_16285
1027.14000	35.0	Cluster_16284
1027.88000	35.0	Cluster_16291
1028.18000	35.0	Cluster_16295
1028.84000	35.0	Cluster_16300
1029.99000	35.0	Cluster_41262
1031.20000	35.0	Cluster_16323
1032.82000	35.0	Cluster_16335
1033.20000	35.0	Cluster_16342
1036.52000	35.0	Cluster_16382
1037.50000	35.0	Cluster_16400
1039.05000	35.0	Cluster_41371
1039.52000	35.0	Cluster_41374
1039.81000	35.0	Cluster_16429
1040.83000	35.0	Cluster_05437
1041.01000	35.0	Cluster_41401
1042.17000	35.0	Cluster_16457
1044.49000	35.0	Cluster_41432
1046.13000	35.0	Cluster_16488
1046.22000	35.0	Cluster_16495
1047.16000	35.0	Cluster_16507
1047.83000	35.0	Cluster_41470
1053.50000	35.0	Cluster_16581
1053.98000	35.0	Cluster_41534
1054.99000	35.0	Cluster_05476
1055.22000	35.0	Cluster_16595
1055.50000	35.0	Cluster_16606
1056.52000	35.0	Cluster_16616
1057.06000	35.0	Cluster_41567
1057.33000	35.0	Cluster_05486
1057.34000	35.0	Cluster_05487
1057.58000	35.0	Cluster_41574
1057.99000	35.0	Cluster_41578
1058.02000	35.0	Cluster_41582
1058.81000	35.0	Cluster_16658
1058.85000	35.0	Cluster_16649
1058.96000	35.0	Cluster_41595

1058.97000	35.0	Cluster_41597
1059.01000	35.0	Cluster_41596
1060.32000	35.0	Cluster_41612
1060.75000	35.0	Cluster_05502
1060.82000	35.0	Cluster_16679
1061.26000	35.0	Cluster_05510
1061.33000	35.0	Cluster_05506
1061.82000	35.0	Cluster_41625
1062.83000	35.0	Cluster_16700
1063.27000	35.0	Cluster_05516
1063.82000	35.0	Cluster_41655
1063.99000	35.0	Cluster_41663
1064.72000	35.0	Cluster_05521
1064.73000	35.0	Cluster_41675
1064.99000	35.0	Cluster_41673
1066.26000	35.0	Cluster_41702
1067.52000	35.0	Cluster_41715
1068.05000	35.0	Cluster_41719
1068.75000	35.0	Cluster_41734
1069.51000	35.0	Cluster_41748
1070.16000	35.0	Cluster_16781
1070.51000	35.0	Cluster_05546
1072.55000	35.0	Cluster_16804
1072.76000	35.0	Cluster_05555
1074.19000	35.0	Cluster_16831
1074.51000	35.0	Cluster_41810
1075.53000	35.0	Cluster_41816
1076.75000	35.0	Cluster_05574
1078.01000	35.0	Cluster_05578
1079.49000	35.0	Cluster_05582
1080.75000	35.0	Cluster_00593
1082.00000	35.0	Cluster_05588
1083.50000	35.0	Cluster_05592
1086.59000	35.0	Cluster_41940
1091.58000	35.0	Cluster_41998
1092.90000	35.0	Cluster_17025
1093.21000	35.0	Cluster_17031
1093.55000	35.0	Cluster_17035
1093.58000	35.0	Cluster_42024
1093.99000	35.0	Cluster_42029
1094.92000	35.0	Cluster_00625
1096.55000	35.0	Cluster_42051
1100.23000	35.0	Cluster_17091
1100.56000	35.0	Cluster_17097
1101.96000	35.0	Cluster_17105
1102.54000	35.0	Cluster_17111
1103.12000	35.0	Cluster_42104
1108.23000	35.0	Cluster_17176
1108.37000	35.0	Cluster_42148
1108.88000	35.0	Cluster_17175
1109.86000	35.0	Cluster_17199

1113.50000	35.0	Cluster_42184
1113.53000	35.0	Cluster_17241
1115.18000	35.0	Cluster_17258
1115.77000	35.0	Cluster_00660
1117.22000	35.0	Cluster_17276
1118.21000	35.0	Cluster_17277
1118.22000	35.0	Cluster_17280
1119.87000	35.0	Cluster_42234
1121.54000	35.0	Cluster_17322
1123.04000	35.0	Cluster_42270
1123.97000	35.0	Cluster_42271
1124.25000	35.0	Cluster_17359
1125.57000	35.0	Cluster_42283
1126.07000	35.0	Cluster_42291
1127.88000	35.0	Cluster_17398
1128.57000	35.0	Cluster_17409
1129.21000	35.0	Cluster_17418
1129.55000	35.0	Cluster_17428
1129.90000	35.0	Cluster_17433
1130.23000	35.0	Cluster_17434
1131.58000	35.0	Cluster_17448
1133.13000	35.0	Cluster_42337
1133.20000	35.0	Cluster_17479
1133.22000	35.0	Cluster_17484
1134.90000	35.0	Cluster_17503
1135.23000	35.0	Cluster_17509
1135.51000	35.0	Cluster_17513
1135.54000	35.0	Cluster_17519
1135.55000	35.0	Cluster_17517
1136.11000	35.0	Cluster_42363
1136.88000	35.0	Cluster_17525
1136.89000	35.0	Cluster_17538
1138.13000	35.0	Cluster_42377
1140.22000	35.0	Cluster_17567
1143.18000	35.0	Cluster_00674
1143.56000	35.0	Cluster_17607
1143.60000	35.0	Cluster_42409
1144.20000	35.0	Cluster_17626
1144.24000	35.0	Cluster_17620
1145.21000	35.0	Cluster_17645
1146.57000	35.0	Cluster_00680
1149.29000	35.0	Cluster_42461
1151.05000	35.0	Cluster_42474
1152.54000	35.0	Cluster_17735
1152.88000	35.0	Cluster_17750
1153.03000	35.0	Cluster_05790
1153.76000	35.0	Cluster_00695
1154.35000	35.0	Cluster_42485
1155.34000	35.0	Cluster_42501
1156.84000	35.0	Cluster_42511
1158.84000	35.0	Cluster_42519

1159.61000	35.0	Cluster_42529
1160.34000	35.0	Cluster_42534
1162.11000	35.0	Cluster_42551
1162.64000	35.0	Cluster_42555
1163.09000	35.0	Cluster_05817
1166.08000	35.0	Cluster_05827
1166.14000	35.0	Cluster_42592
1166.21000	35.0	Cluster_17894
1167.19000	35.0	Cluster_17908
1167.57000	35.0	Cluster_17921
1168.21000	35.0	Cluster_17931
1168.33000	35.0	Cluster_42608
1168.58000	35.0	Cluster_05832
1173.07000	35.0	Cluster_42645
1180.80000	35.0	Cluster_05888
1183.23000	35.0	Cluster_18040
1187.59000	35.0	Cluster_18075
1190.56000	35.0	Cluster_18091
1192.81000	35.0	Cluster_42765
1192.92000	35.0	Cluster_18117
1195.88000	35.0	Cluster_18133
1196.63000	35.0	Cluster_42787
1197.59000	35.0	Cluster_18145
1197.90000	35.0	Cluster_18140
1198.64000	35.0	Cluster_42803
1199.32000	35.0	Cluster_05982
1199.57000	35.0	Cluster_18161
1200.26000	35.0	Cluster_18168
1200.56000	35.0	Cluster_18177
1201.89000	35.0	Cluster_18192
1203.44000	35.0	Cluster_42830
1206.62000	35.0	Cluster_42853
1208.56000	35.0	Cluster_18237
1209.51000	35.0	Cluster_42887
1213.12000	35.0	Cluster_42910
1215.15000	35.0	Cluster_42924
1218.11000	35.0	Cluster_42950
1219.09000	35.0	Cluster_42956
1221.25000	35.0	Cluster_18299
1222.10000	35.0	Cluster_42968
1224.08000	35.0	Cluster_42977
1224.60000	35.0	Cluster_42983
1225.59000	35.0	Cluster_42994
1228.07000	35.0	Cluster_43008
1228.11000	35.0	Cluster_43007
1230.63000	35.0	Cluster_43026
1232.57000	35.0	Cluster_43045
1234.58000	35.0	Cluster_43065
1238.57000	35.0	Cluster_43079
1245.28000	35.0	Cluster_18390
1249.16000	35.0	Cluster_43135

1251.87000	35.0	Cluster_43149
1253.87000	35.0	Cluster_43162
1255.86000	35.0	Cluster_43176
1257.08000	35.0	Cluster_06282
1264.27000	35.0	Cluster_18463
1267.20000	35.0	Cluster_43224
1267.68000	35.0	Cluster_43228
1274.29000	35.0	Cluster_18502
1284.15000	35.0	Cluster_43287
1300.69000	35.0	Cluster_43366
1300.70000	35.0	Cluster_43360
1300.73000	35.0	Cluster_43361
1300.89000	35.0	Cluster_43362
1301.68000	35.0	Cluster_43365
1320.21000	35.0	Cluster_43449
1329.73000	35.0	Cluster_43484
1330.45000	35.0	Cluster_06499
1331.69000	35.0	Cluster_43492
1334.68000	35.0	Cluster_43505
1349.89000	35.0	Cluster_43562
1353.71000	35.0	Cluster_43578
1354.21000	35.0	Cluster_43579
1354.66000	35.0	Cluster_06571
1361.03000	35.0	Cluster_18681
1367.16000	35.0	Cluster_43645
1372.75000	35.0	Cluster_43667
1376.18000	35.0	Cluster_43680
1376.34000	35.0	Cluster_18707
1383.67000	35.0	Cluster_18716
1394.65000	35.0	Cluster_43739
1396.44000	35.0	Cluster_18732
1402.12000	35.0	Cluster_18741
1406.24000	35.0	Cluster_43793
1407.03000	35.0	Cluster_18752
1414.35000	35.0	Cluster_18768
1417.37000	35.0	Cluster_18772
1420.01000	35.0	Cluster_18783
1422.96000	35.0	Cluster_43843
1423.47000	35.0	Cluster_43844
1424.69000	35.0	Cluster_18789
1424.79000	35.0	Cluster_43851
1425.27000	35.0	Cluster_43853
1427.03000	35.0	Cluster_18794
1428.82000	35.0	Cluster_43862
1429.02000	35.0	Cluster_18795
1430.01000	35.0	Cluster_18796
1431.68000	35.0	Cluster_18799
1437.03000	35.0	Cluster_18803
1439.35000	35.0	Cluster_18807
1459.27000	35.0	Cluster_43959
1477.30000	35.0	Cluster_44015

1489.22000	35.0	Cluster_44043
1492.94000	35.0	Cluster_44054
1495.14000	35.0	Cluster_44065
1511.80000	35.0	Cluster_44102
1518.72000	35.0	Cluster_44112
1530.33000	35.0	Cluster_44125
1560.76000	35.0	Cluster_44175
1564.32000	35.0	Cluster_44184
1571.76000	35.0	Cluster_44194
1582.85000	35.0	Cluster_44218
1616.85000	35.0	Cluster_44250
1633.97000	35.0	Cluster_44257
1635.40000	35.0	Cluster_44260
1676.82000	35.0	Cluster_44320
1683.84000	35.0	Cluster_44341
1687.92000	35.0	Cluster_44353
1739.94000	35.0	Cluster_44403
1792.99000	35.0	Cluster_44425

Reject Mass List: (none)
Neutral Loss Mass List: (none)
Product Mass List: (none)
Neutral loss in top: 3
Product in top: 3
Most intense if no parent masses found enabled
Add/subtract mass not enabled
FT master scan preview mode enabled
Charge state screening enabled
Charge state dependent ETD time not enabled
Monoisotopic precursor selection enabled
Charge state rejection enabled
Unassigned charge states : rejected
Charge state 1 : rejected
Charge state 2 : not rejected
Charge state 3 : not rejected
Charge states 4+ : not rejected
Chromatography mode is disabled

Global Data Dependent Settings:
Predict ion injection time enabled
Use global parent and reject mass lists not enabled
Exclude parent mass from data dependent selection not enabled
Exclusion mass width by mass
Exclusion mass width low: 0.50000
Exclusion mass width high: 0.50000
Parent mass width by mass
Parent mass width low: 0.50000
Parent mass width high: 0.50000
Reject mass width by mass
Reject mass width low: 0.50000
Reject mass width high: 0.50000
Zoom/UltraZoom scan mass width by mass

Zoom/UltraZoom scan mass width low: 5.00
Zoom/UltraZoom scan mass width high: 5.00
FT SIM scan mass width low: 5.00
FT SIM scan mass width high: 5.00
Neutral Loss candidates processed by decreasing intensity
Neutral Loss mass width by mass
Neutral Loss mass width low: 0.50000
Neutral Loss mass width high: 0.50000
Product candidates processed by decreasing intensity
Product mass width by mass
Product mass width low: 0.50000
Product mass width high: 0.50000
MS mass range: 0.00-1000000.00
MSn mass range by mass
MSn mass range: 0.00-1000000.00
Use m/z values as masses not enabled
Analog UV data dep. not enabled
Dynamic exclusion not enabled
Isotopic data dependence not enabled
Mass Tags data dependence not enabled

Custom Data Dependent Settings:

Not enabled

4. Galaxy workflow to create a wheat non redundant FASTA database with contaminant and decoy sequences

To use the workflow below, simply copy/paste the command line into a text editor and save it with a .ga extension. Galaxy will then be able to recognise and import it.


```
{
    "a_galaxy_workflow": "true",
    "annotation": "",
    "format-version": "0.1",
    "name": "Wheat - creating non redundant FASTA DBs with decoy",
    "steps": {
        "0": {
            "annotation": "",
            "content_id": null,
            "errors": null,
            "id": 0,
            "input_connections": {},
            "inputs": [
                {
                    "description": "",
                    "name": "from-GKG_alpha-amylase-blast_2022-03-
08.fasta"
                }
            ],
            "label": "from-GKG_alpha-amylase-blast_2022-03-
08.fasta",
            "name": "Input dataset",
            "outputs": [],
            "position": {
                "bottom": 207.96875,
                "height": 102.5625,
                "left": 360.25,
                "right": 560.25,
                "top": 105.40625,
                "width": 200,
                "x": 360.25,
                "y": 105.40625
            },
            "tool_id": null,
            "tool_state": "{\"optional\": false}",
            "tool_version": null,
            "type": "data_input",
            "uuid": "b4d34c10-e494-478e-b73a-e0672b3431f6",
            "workflow_outputs": []
        },
        "1": {
            "annotation": "",
            "content_id": null,
            "errors": null,
            "id": 1,
            "input_connections": {},
            "inputs": [
                {
                    "description": "",
```

```
                    "name": "Triticum_aestivum.IWGSC.pep.all.fa"
                }
            ],
            "label": "Triticum_aestivum.IWGSC.pep.all.fa",
            "name": "Input dataset",
            "outputs": [],
            "position": {
                "bottom": 318.96875,
                "height": 102.5625,
                "left": 358.25,
                "right": 558.25,
                "top": 216.40625,
                "width": 200,
                "x": 358.25,
                "y": 216.40625
            },
            "tool_id": null,
            "tool_state": "{\"optional\": false}",
            "tool_version": null,
            "type": "data_input",
            "uuid": "7152c491-219f-47f0-ad77-cad307e81d00",
            "workflow_outputs": []
        },
        "2": {
            "annotation": "",
            "content_id": null,
            "errors": null,
            "id": 2,
            "input_connections": {},
            "inputs": [
                {
                    "description": "",
                    "name": "uniprot_Triticum-
aestivum_142969entries_2020-02-26.fasta"
                }
            ],
            "label": "uniprot_Triticum-aestivum_142969entries_2020-
02-26.fasta",
            "name": "Input dataset",
            "outputs": [],
            "position": {
                "bottom": 452.359375,
                "height": 122.953125,
                "left": 355.25,
                "right": 555.25,
                "top": 329.40625,
                "width": 200,
                "x": 355.25,
                "y": 329.40625
            },
            "tool_id": null,
            "tool_state": "{\"optional\": false}",
            "tool_version": null,
            "type": "data_input",
            "uuid": "fda351ea-f08d-426d-a946-4eaefa86dd0a",
            "workflow_outputs": []
```

```
        },
        "3": {
            "annotation": "",
            "content_id": null,
            "errors": null,
            "id": 3,
            "input_connections": {},
            "inputs": [
                {
                    "description": "",
                    "name": "1-uniprot_Triticum-
aestivum_142969entries_2020-02-26.fasta"
                }
            ],
            "label": "1-uniprot_Triticum-
aestivum_142969entries_2020-02-26.fasta",
            "name": "Input dataset",
            "outputs": [],
            "position": {
                "bottom": 633.359375,
                "height": 122.953125,
                "left": 353.25,
                "right": 553.25,
                "top": 510.40625,
                "width": 200,
                "x": 353.25,
                "y": 510.40625
            },
            "tool_id": null,
            "tool_state": "{\"optional\": false}",
            "tool_version": null,
            "type": "data_input",
            "uuid": "f2f13af5-a14a-4d43-9ef1-e26b0eb88597",
            "workflow_outputs": []
        },
        "4": {
            "annotation": "",
            "content_id": null,
            "errors": null,
            "id": 4,
            "input_connections": {},
            "inputs": [
                {
                    "description": "",
                    "name": "2-
Triticum_aestivum.IWGSC.pep.all.fasta"
                }
            ],
            "label": "2-Triticum_aestivum.IWGSC.pep.all.fasta",
            "name": "Input dataset",
            "outputs": [],
            "position": {
                "bottom": 741.96875,
                "height": 102.5625,
                "left": 349.25,
                "right": 549.25,
```

```
                "top": 639.40625,
                "width": 200,
                "x": 349.25,
                "y": 639.40625
            },
            "tool_id": null,
            "tool_state": "{\"optional\": false}",
            "tool_version": null,
            "type": "data_input",
            "uuid": "274c7c93-e1f0-428d-9fce-75e7d69ef118",
            "workflow_outputs": []
        },
        "5": {
            "annotation": "",
            "content_id":
"toolshed.g2.bx.psu.edu/repos/galaxyp/dbbuilder/dbbuilder/0.3.1",
            "errors": null,
            "id": 5,
            "input_connections": {},
            "inputs": [],
            "label": null,
            "name": "Protein Database Downloader",
            "outputs": [
                {
                    "name": "output_database",
                    "type": "fasta"
                }
            ],
            "position": {
                "bottom": 557.96875,
                "height": 102.5625,
                "left": 607.25,
                "right": 807.25,
                "top": 455.40625,
                "width": 200,
                "x": 607.25,
                "y": 455.40625
            },
            "post_job_actions": {},
            "tool_id":
"toolshed.g2.bx.psu.edu/repos/galaxyp/dbbuilder/dbbuilder/0.3.1",
            "tool_shed_repository": {
                "changeset_revision": "c1b437242fee",
                "name": "dbbuilder",
                "owner": "galaxyp",
                "tool_shed": "toolshed.g2.bx.psu.edu"
            },
            "tool_state": "{\"source\": {\"from\": \"cRAP\",
\"__current_case__\": 1}, \"__page__\": null,
\"__rerun_remap_job_id__\": null}",
            "tool_version": "0.3.1",
            "type": "tool",
            "uuid": "0720d479-9fbe-4ba0-9535-c4d97cddcc23",
            "workflow_outputs": [
                {
                    "label": null,
```

```
                    "output_name": "output_database",
                    "uuid": "0d4d9480-f55e-40be-9f17-d5b63eb3f719"
                }
            ]
        },
        "6": {
            "annotation": "",
            "content_id": null,
            "errors": null,
            "id": 6,
            "input_connections": {},
            "inputs": [
                {
                    "description": "",
                    "name": "3-from-GKG_alpha-amylase-blast_2022-03-
08.fasta"
                }
            ],
            "label": "3-from-GKG_alpha-amylase-blast_2022-03-
08.fasta",
            "name": "Input dataset",
            "outputs": [],
            "position": {
                "bottom": 850.96875,
                "height": 102.5625,
                "left": 352.25,
                "right": 552.25,
                "top": 748.40625,
                "width": 200,
                "x": 352.25,
                "y": 748.40625
            },
            "tool_id": null,
            "tool_state": "{\"optional\": false}",
            "tool_version": null,
            "type": "data_input",
            "uuid": "ac70a284-a0f1-469f-bd75-899a0d3e9905",
            "workflow_outputs": []
        },
        "7": {
            "annotation": "",
            "content_id":
"toolshed.g2.bx.psu.edu/repos/galaxyp/fasta_merge_files_and_filter_u
nique_sequences/fasta_merge_files_and_filter_unique_sequences/1.2.0"
,
            "errors": null,
            "id": 7,
            "input_connections": {
                "batchmode|input_fastas": [
                    {
                        "id": 0,
                        "output_name": "output"
                    },
                    {
                        "id": 1,
                        "output_name": "output"
```

```
                    },
                    {
                        "id": 2,
                        "output_name": "output"
                    },
                    {
                        "id": 5,
                        "output_name": "output_database"
                    }
                ]
            },
            "inputs": [],
            "label": null,
            "name": "FASTA Merge Files and Filter Unique Sequences",
            "outputs": [
                {
                    "name": "output",
                    "type": "fasta"
                }
            ],
            "position": {
                "bottom": 455.140625,
                "height": 174.734375,
                "left": 938.25,
                "right": 1138.25,
                "top": 280.40625,
                "width": 200,
                "x": 938.25,
                "y": 280.40625
            },
            "post_job_actions": {},
            "tool_id":
"toolshed.g2.bx.psu.edu/repos/galaxyp/fasta_merge_files_and_filter_u
nique_sequences/fasta_merge_files_and_filter_unique_sequences/1.2.0"
,
            "tool_shed_repository": {
                "changeset_revision": "f546e7278f04",
                "name":
"fasta_merge_files_and_filter_unique_sequences",
                "owner": "galaxyp",
                "tool_shed": "toolshed.g2.bx.psu.edu"
            },
            "tool_state": "{\"accession_parser\": \"^>([^ ]+).*$\",
\"batchmode\": {\"processmode\": \"merge\", \"__current_case__\": 1,
\"input_fastas\": {\"__class__\": \"ConnectedValue\"}},
\"uniqueness_criterion\": \"sequence\", \"__page__\": null,
\"__rerun_remap_job_id__\": null}",
            "tool_version": "1.2.0",
            "type": "tool",
            "uuid": "acf11599-e033-46b5-8ca7-ab97ae8be58b",
            "workflow_outputs": [
                {
                    "label": null,
                    "output_name": "output",
                    "uuid": "848c5bf4-290e-4608-a129-dc565c7692d6"
                }
```

```
            ]
        },
        "8": {
            "annotation": "",
            "content_id":
"toolshed.g2.bx.psu.edu/repos/galaxyp/fasta_merge_files_and_filter_u
nique_sequences/fasta_merge_files_and_filter_unique_sequences/1.2.0"
,
            "errors": null,
            "id": 8,
            "input_connections": {
                "batchmode|input_fastas": [
                    {
                        "id": 3,
                        "output_name": "output"
                    },
                    {
                        "id": 4,
                        "output_name": "output"
                    },
                    {
                        "id": 6,
                        "output_name": "output"
                    },
                    {
                        "id": 5,
                        "output_name": "output_database"
                    }
                ]
            },
            "inputs": [],
            "label": null,
            "name": "FASTA Merge Files and Filter Unique Sequences",
            "outputs": [
                {
                    "name": "output",
                    "type": "fasta"
                }
            ],
            "position": {
                "bottom": 704.140625,
                "height": 174.734375,
                "left": 939.25,
                "right": 1139.25,
                "top": 529.40625,
                "width": 200,
                "x": 939.25,
                "y": 529.40625
            },
            "post_job_actions": {},
            "tool_id":
"toolshed.g2.bx.psu.edu/repos/galaxyp/fasta_merge_files_and_filter_u
nique_sequences/fasta_merge_files_and_filter_unique_sequences/1.2.0"
,
            "tool_shed_repository": {
                "changeset_revision": "f546e7278f04",
```

```
                "name": 
"fasta_merge_files_and_filter_unique_sequences",
                "owner": "galaxyp",
                "tool_shed": "toolshed.g2.bx.psu.edu"
            },
            "tool_state": "{\"accession_parser\": \"^>([^ ]+).*$\", 
\"batchmode\": {\"processmode\": \"merge\", \"__current_case__\": 1, 
\"input_fastas\": {\"__class__\": \"ConnectedValue\"}}, 
\"uniqueness_criterion\": \"sequence\", \"__page__\": null, 
\"__rerun_remap_job_id__\": null}",
            "tool_version": "1.2.0",
            "type": "tool",
            "uuid": "1f78efc3-aeaf-44e3-9400-97da2feaee9c",
            "workflow_outputs": [
                {
                    "label": null,
                    "output_name": "output",
                    "uuid": "08d15055-fb91-42ff-b946-e979934c55f9"
                }
            ]
        },
        "9": {
            "annotation": "",
            "content_id": 
"toolshed.g2.bx.psu.edu/repos/galaxyp/openms_decoydatabase/DecoyData
base/2.6+galaxy0",
            "errors": null,
            "id": 9,
            "input_connections": {
                "in": {
                    "id": 7,
                    "output_name": "output"
                }
            },
            "inputs": [],
            "label": null,
            "name": "DecoyDatabase",
            "outputs": [
                {
                    "name": "out",
                    "type": "fasta"
                }
            ],
            "position": {
                "bottom": 445.75,
                "height": 154.34375,
                "left": 1210.25,
                "right": 1410.25,
                "top": 291.40625,
                "width": 200,
                "x": 1210.25,
                "y": 291.40625
            },
            "post_job_actions": {},
```

```
            "tool_id":
"toolshed.g2.bx.psu.edu/repos/galaxyp/openms_decoydatabase/DecoyData
base/2.6+galaxy0",
            "tool_shed_repository": {
                "changeset_revision": "370141bc0da3",
                "name": "openms_decoydatabase",
                "owner": "galaxyp",
                "tool_shed": "toolshed.g2.bx.psu.edu"
            },
            "tool_state": "{\"Decoy\": {\"non_shuffle_pattern\":
\"\", \"keepPeptideNTerm\": \"true\", \"keepPeptideCTerm\":
\"true\"}, \"OPTIONAL_OUTPUTS\": null, \"adv_opts_cond\":
{\"adv_opts_selector\": \"basic\", \"__current_case__\": 0},
\"decoy_string\": \"DECOY_\", \"decoy_string_position\": \"prefix\",
\"enzyme\": \"Trypsin\", \"in\": {\"__class__\":
\"ConnectedValue\"}, \"method\": \"reverse\", \"only_decoy\":
\"false\", \"type\": \"protein\", \"__page__\": null,
\"__rerun_remap_job_id__\": null}",
            "tool_version": "2.6+galaxy0",
            "type": "tool",
            "uuid": "68e37f73-0773-4ff3-895b-5286dea2efe1",
            "workflow_outputs": [
                {
                    "label": null,
                    "output_name": "out",
                    "uuid": "5ee1eec0-9464-4428-81ea-263b09602f36"
                }
            ]
        },
        "10": {
            "annotation": "",
            "content_id":
"toolshed.g2.bx.psu.edu/repos/galaxyp/openms_decoydatabase/DecoyData
base/2.6+galaxy0",
            "errors": null,
            "id": 10,
            "input_connections": {
                "in": {
                    "id": 8,
                    "output_name": "output"
                }
            },
            "inputs": [],
            "label": null,
            "name": "DecoyDatabase",
            "outputs": [
                {
                    "name": "out",
                    "type": "fasta"
                }
            ],
            "position": {
                "bottom": 695.75,
                "height": 154.34375,
                "left": 1216.25,
                "right": 1416.25,
```

```
                "top": 541.40625,
                "width": 200,
                "x": 1216.25,
                "y": 541.40625
            },
            "post_job_actions": {},
            "tool_id":
"toolshed.g2.bx.psu.edu/repos/galaxyp/openms_decoydatabase/DecoyData
base/2.6+galaxy0",
            "tool_shed_repository": {
                "changeset_revision": "370141bc0da3",
                "name": "openms_decoydatabase",
                "owner": "galaxyp",
                "tool_shed": "toolshed.g2.bx.psu.edu"
            },
            "tool_state": "{\"Decoy\": {\"non_shuffle_pattern\":
\"\", \"keepPeptideNTerm\": \"true\", \"keepPeptideCTerm\":
\"true\"}, \"OPTIONAL_OUTPUTS\": null, \"adv_opts_cond\":
{\"adv_opts_selector\": \"basic\", \"__current_case__\": 0},
\"decoy_string\": \"DECOY_\", \"decoy_string_position\": \"prefix\",
\"enzyme\": \"Trypsin\", \"in\": {\"__class__\":
\"ConnectedValue\"}, \"method\": \"reverse\", \"only_decoy\":
\"false\", \"type\": \"protein\", \"__page__\": null,
\"__rerun_remap_job_id__\": null}",
            "tool_version": "2.6+galaxy0",
            "type": "tool",
            "uuid": "0c5ec050-869a-4657-b1f7-e8827236f495",
            "workflow_outputs": [
                {
                    "label": null,
                    "output_name": "out",
                    "uuid": "9bf5c5ff-cb8b-4dc7-809d-53f987794688"
                }
            ]
        }
    },
    "tags": [],
    "uuid": "53b7f604-35b5-41ec-b665-bedbd5c6a8f7",
    "version": 2
}
```

5. R script to link LC-MS1 clusters to LC-MS2 clusters

In this study, the 32,336 peptide clusters from the corrected dataset produced by the LCMS1 analyses were matched in R to the 29,908 peptide clusters generated by the LC-MS2 analyses using their respective retention time in min (RT), mass-to-charge ratio (*m/z*) and mass values with ±0.1 accuracy. The identification results of the peptide clusters whose RT shifted by more than 1 min were not included. LCMS1 and LCMS2 data files were formatted as described in sections 5.1 and 5.2. The R script detailed in section 5.3 was executed to link both cluster sets.

5.1 LCMS1 data

The LCMS1-clusters.csv file should have the following format:

Name	charge	mass	m/z	RT
LCMS1_Cluster_00004	7	5987.184499	856.3193477	31.20762132
LCMS1_Cluster_00005	6	1963.608341	328.2753334	8.964887201
LCMS1_Cluster_00006	6	2158.705017	360.791446	22.90218307
LCMS1_Cluster_00007	6	2517.239683	420.5472236	18.88168878
LCMS1_Cluster_00008	6	2798.408952	467.4087684	6.213851091
LCMS1_Cluster_00010	6	2817.332146	470.5626342	7.808784963
LCMS1_Cluster_00011	6	2954.506446	493.4250174	20.51954376
LCMS1_Cluster_00012	6	3022.959234	504.8338155	23.97602643
LCMS1_Cluster_00013	6	3049.690826	509.2890808	28.52867505
LCMS1_Cluster_00017	6	3511.51342	586.2595132	16.50431069
LCMS1_Cluster_00018	6	3746.027423	625.3451804	23.61489309

Etc…

5.2 LCMS2 data

The LCMS2-clusters.csv file should have the following format:

Name	charge	mass	m/z	RT
LCMS2_Cluster_00001	9	4213.044711	469.1233555	24.80944814
LCMS2_Cluster_00002	6	1960.035197	327.6798093	3.481241834
LCMS2_Cluster_00003	6	1972.015064	329.6764537	12.70818926
LCMS2_Cluster_00004	6	2006.144019	335.364613	6.831586735
LCMS2_Cluster_00005	6	2024.875246	338.4864841	3.82342595
LCMS2_Cluster_00006	6	2096.240041	350.3806166	10.51081511
LCMS2_Cluster_00007	6	2126.132939	355.3627663	4.848375927
LCMS2_Cluster_00008	6	2208.04959	369.0155414	5.641878778
LCMS2_Cluster_00009	6	2209.056864	369.1834204	16.34450342
LCMS2_Cluster_00010	6	2324.245956	388.3816025	10.32717327
LCMS2_Cluster_00011	6	2388.349998	399.0656095	8.474850487

Etc…

5.3 R script

Both csv files were imported into R environment. The R script indicated below matches both LCMS1 and LCMS2 cluster lists based on their charge, mass, m/z and RT in min.
To run the script, simply copy/paste the command line into R or a compatible environment such as RStudio and execute it.
If you'd like to use different tolerances, simply modify the numbers indicated in the script.

```
LCMS1<-read.csv(".\\LCMS1-clusters.csv", header=T, sep=",")
LCMS2<-read.csv(".\\LCMS2-clusters.csv", header=T, sep=",")

LCMS1<-LCMS1[order(LCMS1$mass),]
LCMS2<-LCMS2[order(LCMS2$mass),]

merged<-NULL
for (i in 1:nrow(LCMS2)){
    n_mass<-LCMS2[i, 'mass']
    o_mass_l<-n_mass-0.1
    o_mass_u<-n_mass+0.1

    n_mz<-LCMS2[i, 'm.z']
    o_mz_l<-n_mz-0.01
    o_mz_u<-n_mz+0.01

    n_rt<-LCMS2[i, 'RT']
    o_rt_l<-n_rt-0.1
    o_rt_u<-n_rt+0.1

    tmp<-LCMS1[which(LCMS1$mass>=o_mass_l & LCMS1$mass<o_mass_u),]
      if (nrow(tmp)==0){
        o_mass_l<-n_mass-1.99
        o_mass_u<-n_mass+1.99
        tmp<-LCMS1[which(LCMS1$mass>=o_mass_l &
LCMS1$mass<o_mass_u),]
      }

      if (nrow(tmp)==0){
        next
        }

      tmp2<-tmp[which(tmp$m.z>=o_mz_l & tmp$m.z<o_mz_u),]
      if (nrow(tmp2)==0){
        o_mz_l<-n_mz-1.99
        o_mz_u<-n_mz+1.99
        tmp2<-tmp[which(tmp$m.z>=o_mz_l & tmp$m.z<o_mz_u),]
      }

      if (nrow(tmp2)==0){
        next
        }

      dist=1000
    for(j in 1:nrow(tmp2)){
        d<-sum(abs(n_mass-tmp2[j, 'mass'])+(abs(n_mz-tmp2[j,
'm.z']))+ (abs(n_rt-tmp2[j, 'RT'])))
          if (d<dist){
            dist<-d
            o_tmp<-tmp2[j,]
            }#if
      }#j

  merged<-rbind(merged, cbind(o_tmp, LCMS2[i,]))
```

```
}#i

write.csv(merged, file="./merged_Mass_MZ.csv", row.names=F,
col.names = T, sep=",")
```

5.4 LCMS1-LCMS2 linked output

Matched files are merged into a single tabular file with the following format:

Name	charge	mass	m.z	RT	Name	charge	mass	m.z	RT
LCMS1_Cluster_13784	2	598.1675	300.0910	31.5988	LCMS2_Cluster_03834	2	598.1692	300.0919	31.9680
LCMS1_Cluster_13785	2	598.1886	300.1016	6.6407	LCMS2_Cluster_03835	2	598.1898	300.1022	7.3814
LCMS1_Cluster_13787	2	598.2042	300.1094	18.4285	LCMS2_Cluster_03837	2	598.2080	300.1113	18.8839
LCMS1_Cluster_13787	2	598.2042	300.1094	18.4285	LCMS2_Cluster_03836	2	598.2087	300.1116	18.6304
LCMS1_Cluster_13792	2	598.2639	300.1392	12.8899	LCMS2_Cluster_03838	2	598.2443	300.1294	39.4478
LCMS1_Cluster_13798	2	598.3208	300.1677	7.9535	LCMS2_Cluster_03841	2	598.2909	300.1527	9.5876
LCMS1_Cluster_13794	2	598.3551	300.1848	6.1704	LCMS2_Cluster_03839	2	598.3581	300.1863	7.0153
