# Supplementary figures and images for: A community resource to mass explore the wheat grain proteome and its application to the late-maturity alpha-amylase (LMA) problem
Source: Gigascience. 2023 Nov 1;12:giad084. doi: 10.1093/gigascience/giad084 (PMC10627334; doi:10.1093/gigascience/giad084)

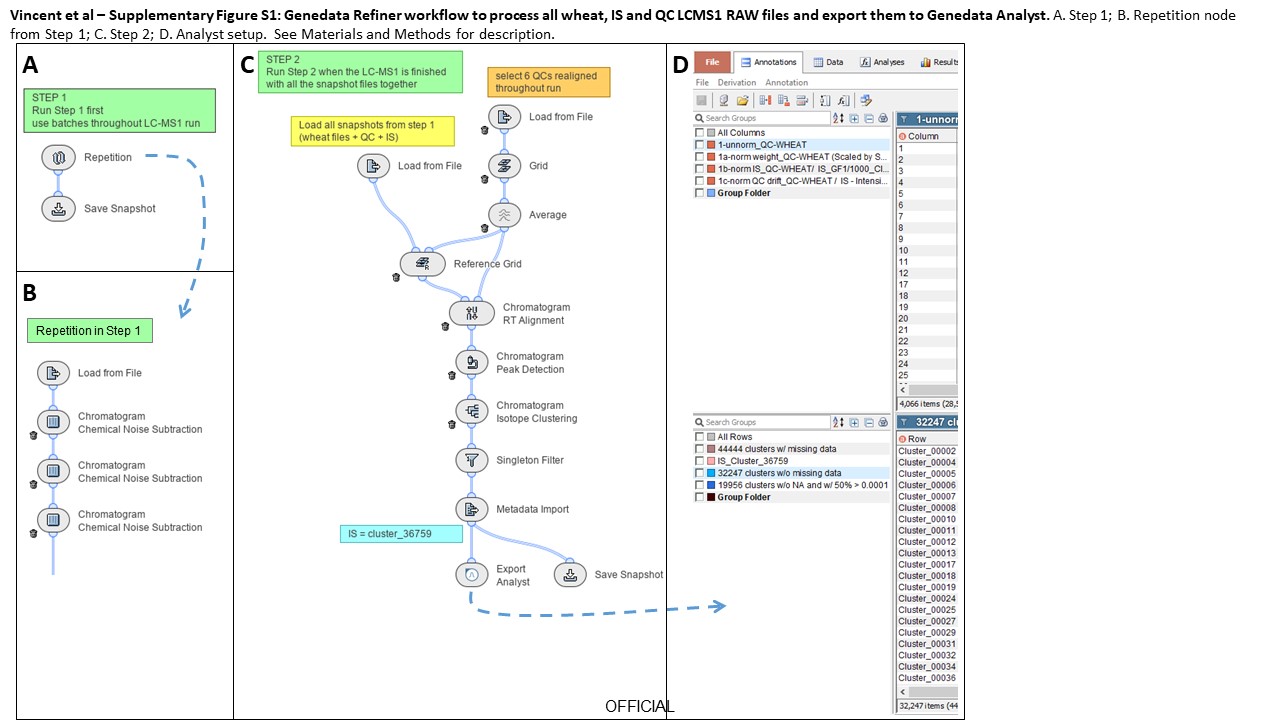

Supplement: giad084_Supplemental_Files [file giad084_supplemental_files.zip › Vincent_Suppl-Fig-S01.JPG]

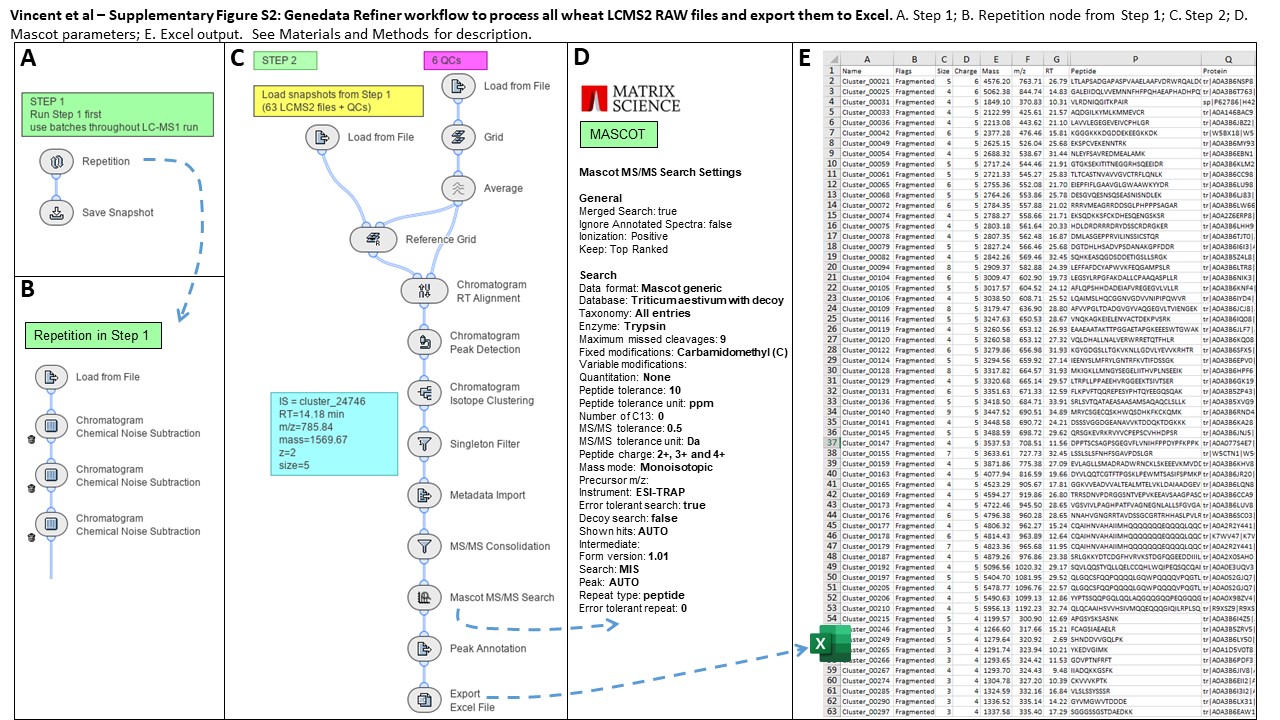

Supplement: giad084_Supplemental_Files [file giad084_supplemental_files.zip › Vincent_Suppl-Fig-S02.JPG]

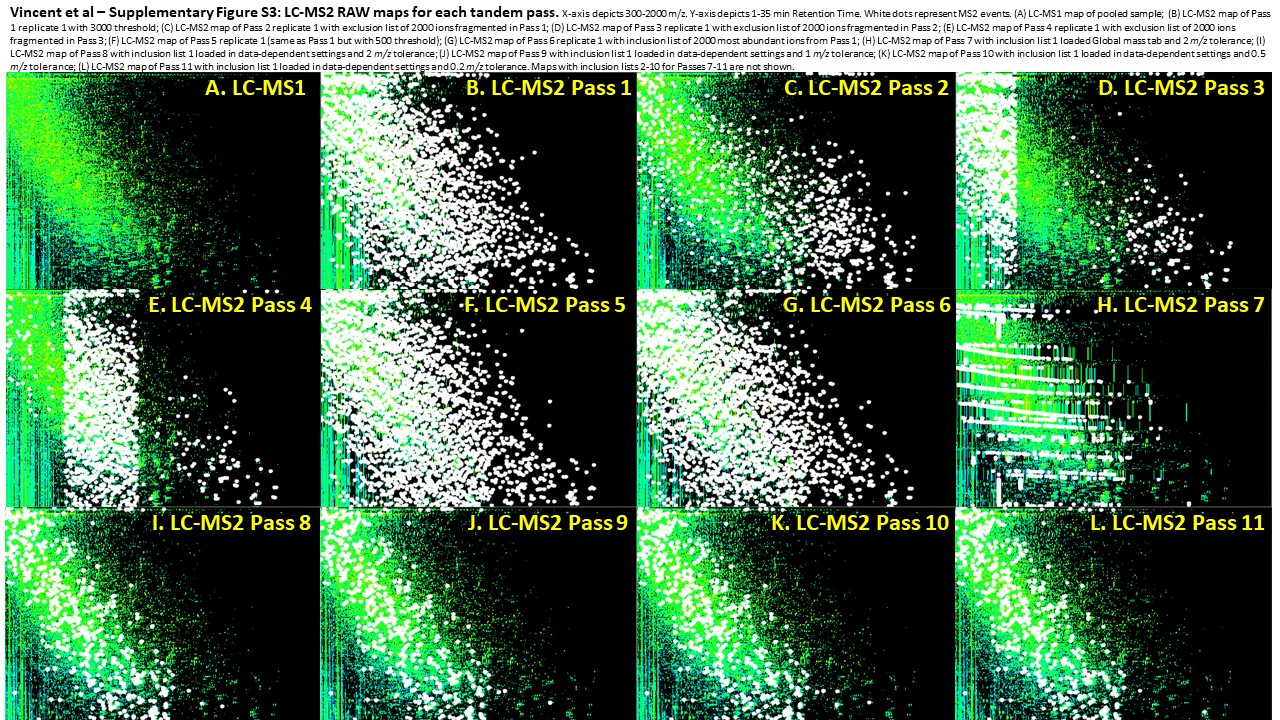

Supplement: giad084_Supplemental_Files [file giad084_supplemental_files.zip › Vincent_Suppl-Fig-S03.JPG]

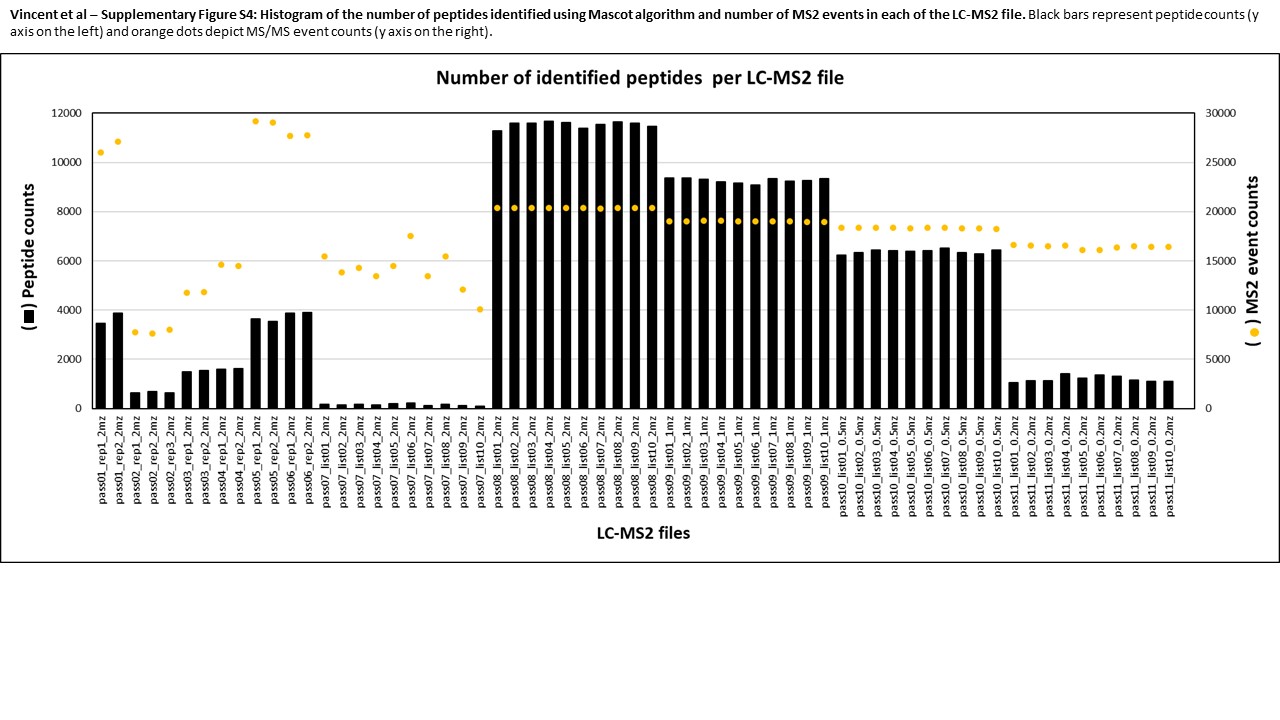

Supplement: giad084_Supplemental_Files [file giad084_supplemental_files.zip › Vincent_Suppl-Fig-S04.JPG]

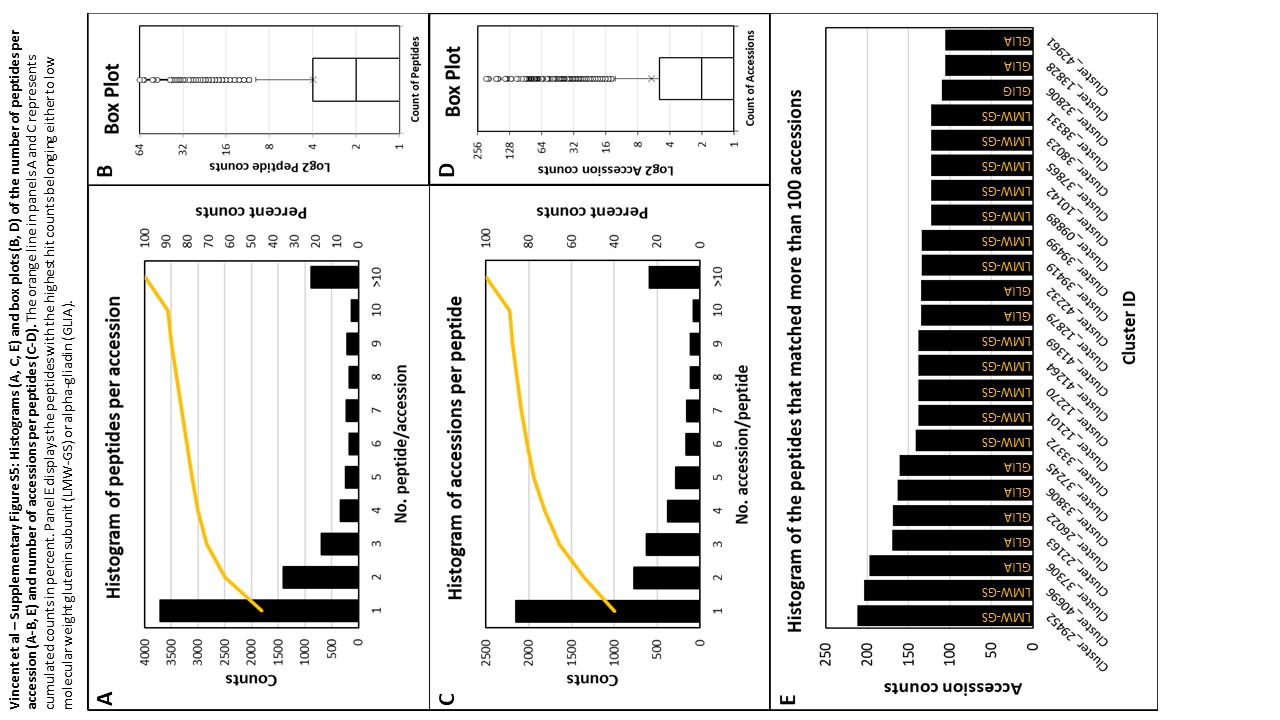

Supplement: giad084_Supplemental_Files [file giad084_supplemental_files.zip › Vincent_Suppl-Fig-S05.JPG]

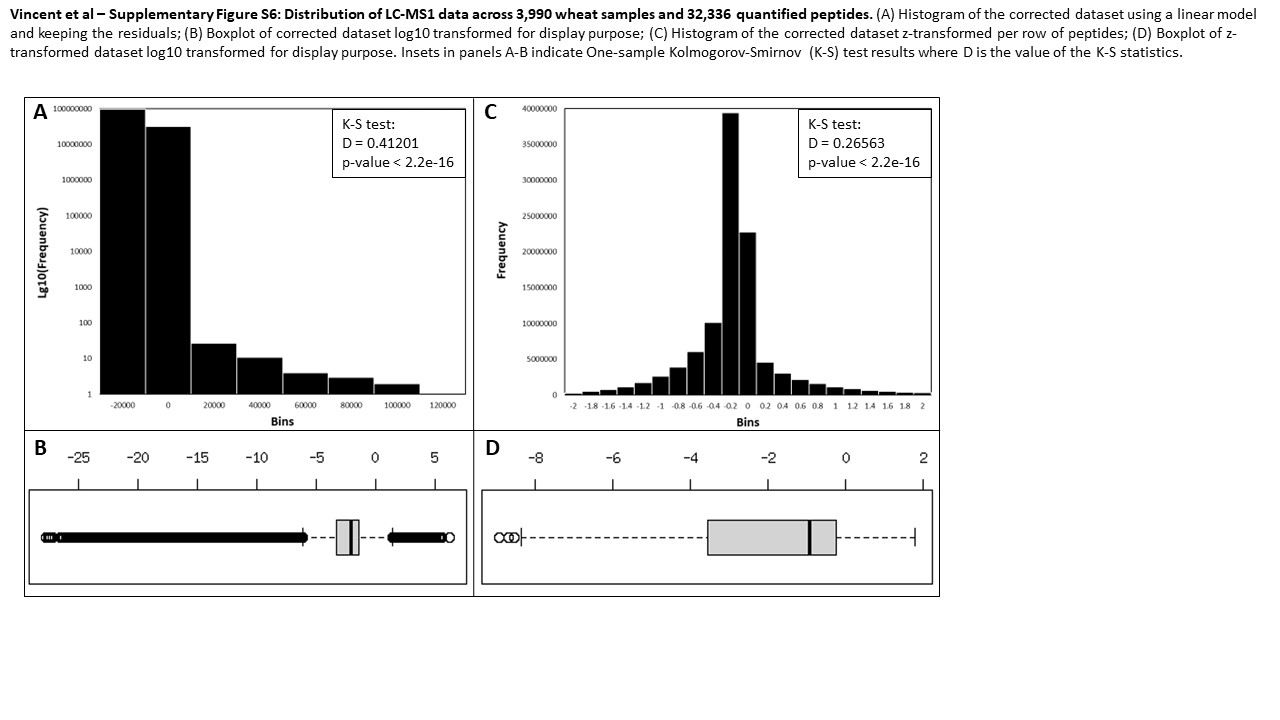

Supplement: giad084_Supplemental_Files [file giad084_supplemental_files.zip › Vincent_Suppl-Fig-S06.JPG]

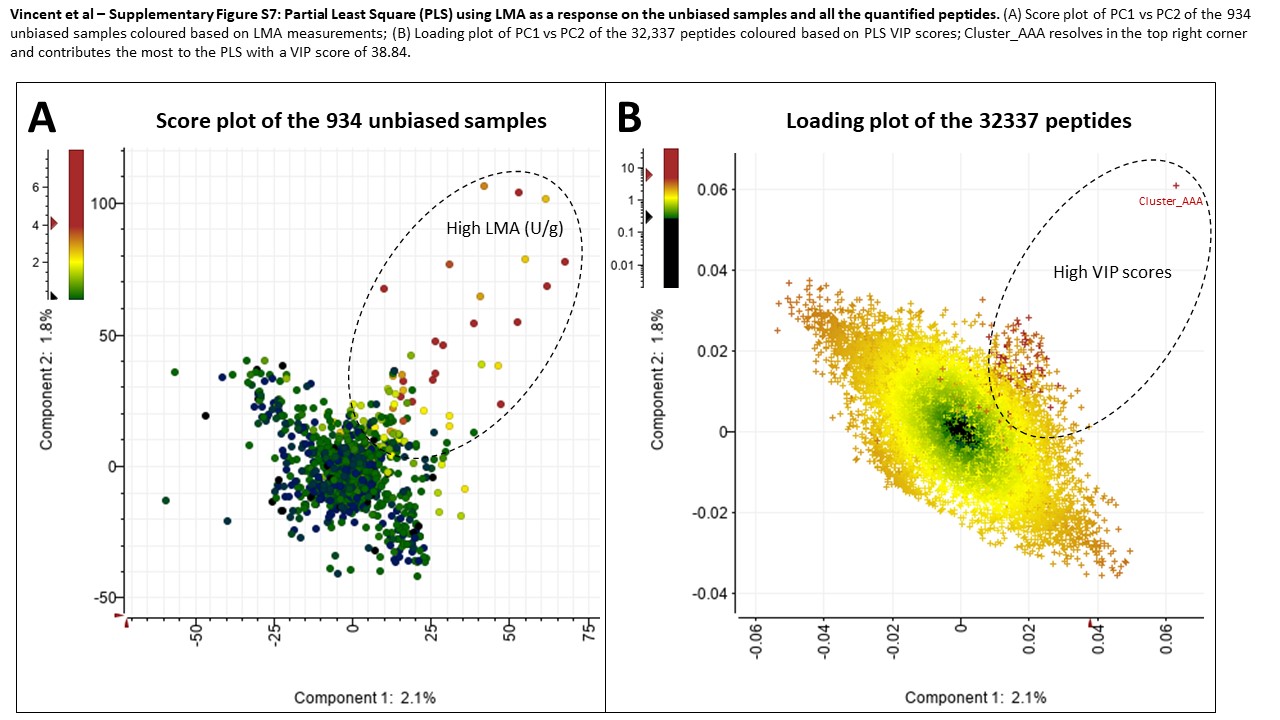

Supplement: giad084_Supplemental_Files [file giad084_supplemental_files.zip › Vincent_Suppl-Fig-S07.JPG]

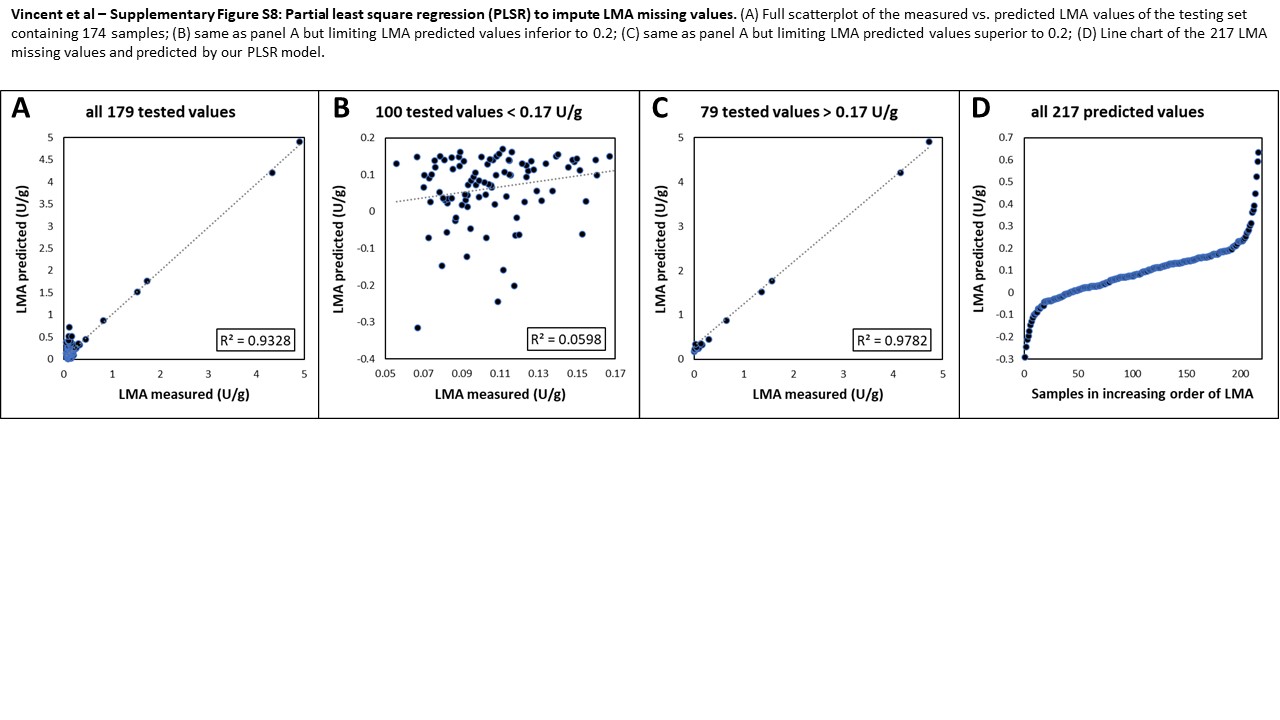

Supplement: giad084_Supplemental_Files [file giad084_supplemental_files.zip › Vincent_Suppl-Fig-S08.JPG]

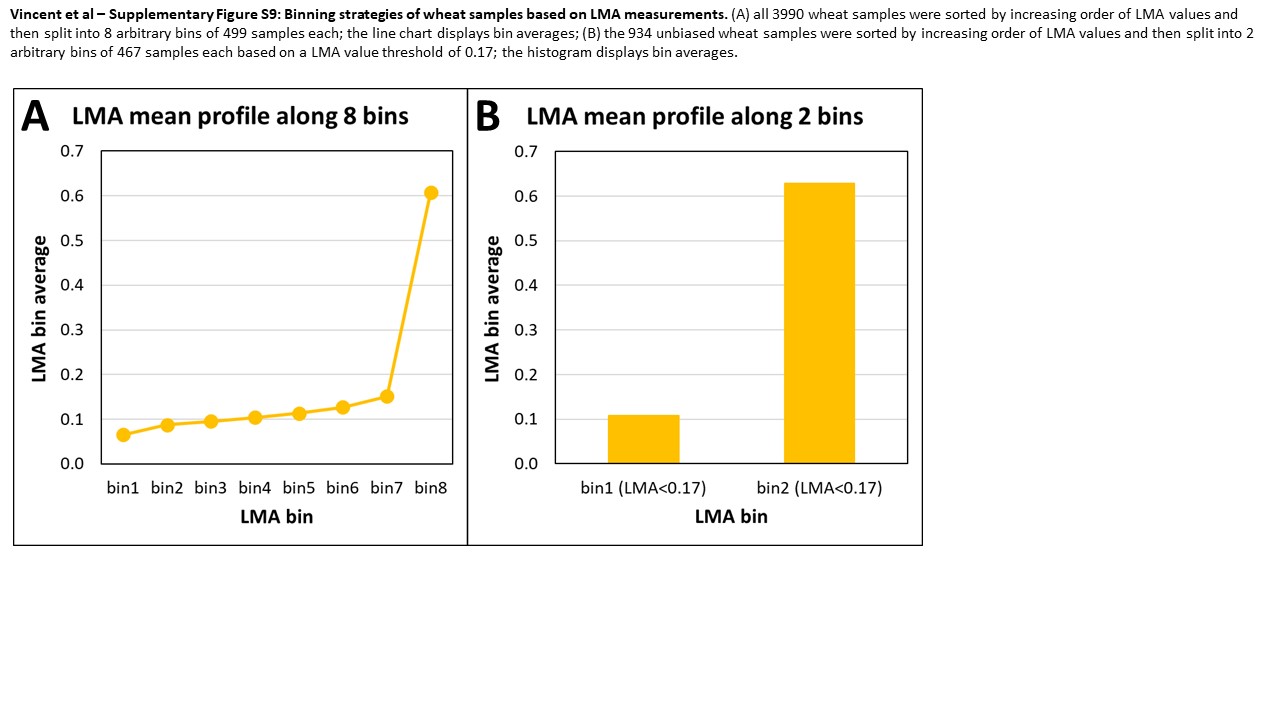

Supplement: giad084_Supplemental_Files [file giad084_supplemental_files.zip › Vincent_Suppl-Fig-S09.JPG]

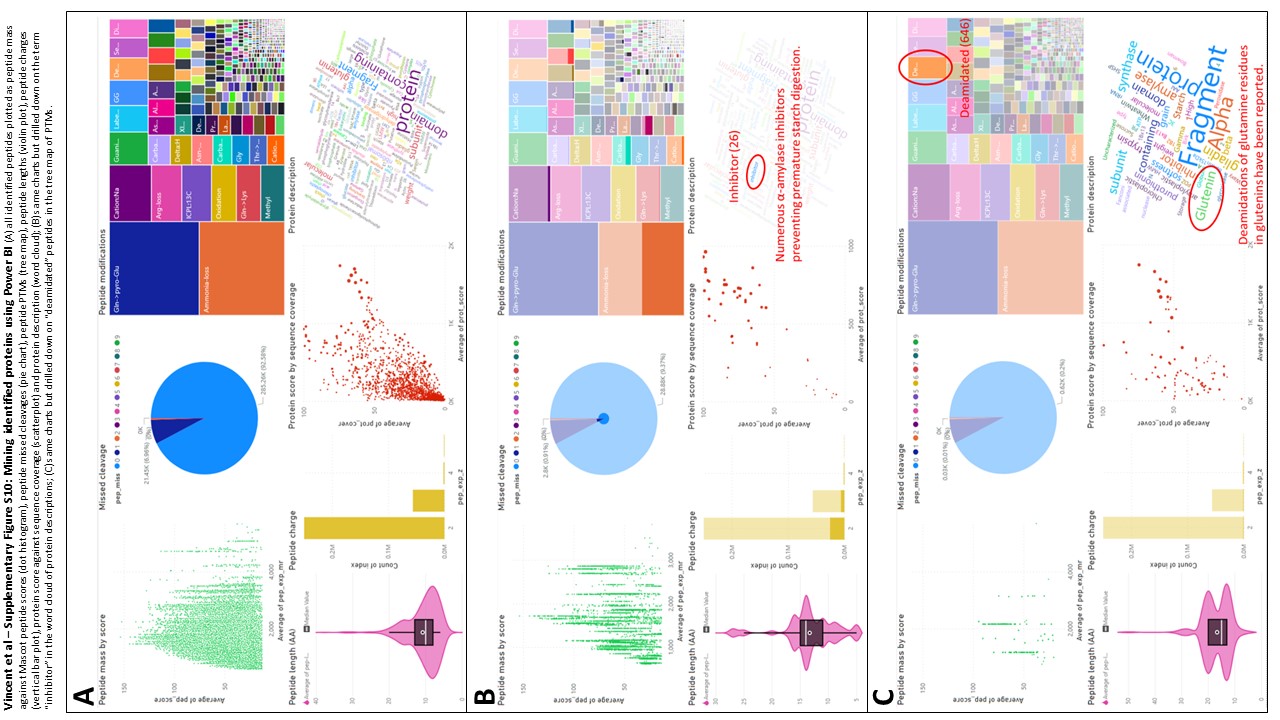

Supplement: giad084_Supplemental_Files [file giad084_supplemental_files.zip › Vincent_Suppl-Fig-S10.JPG]

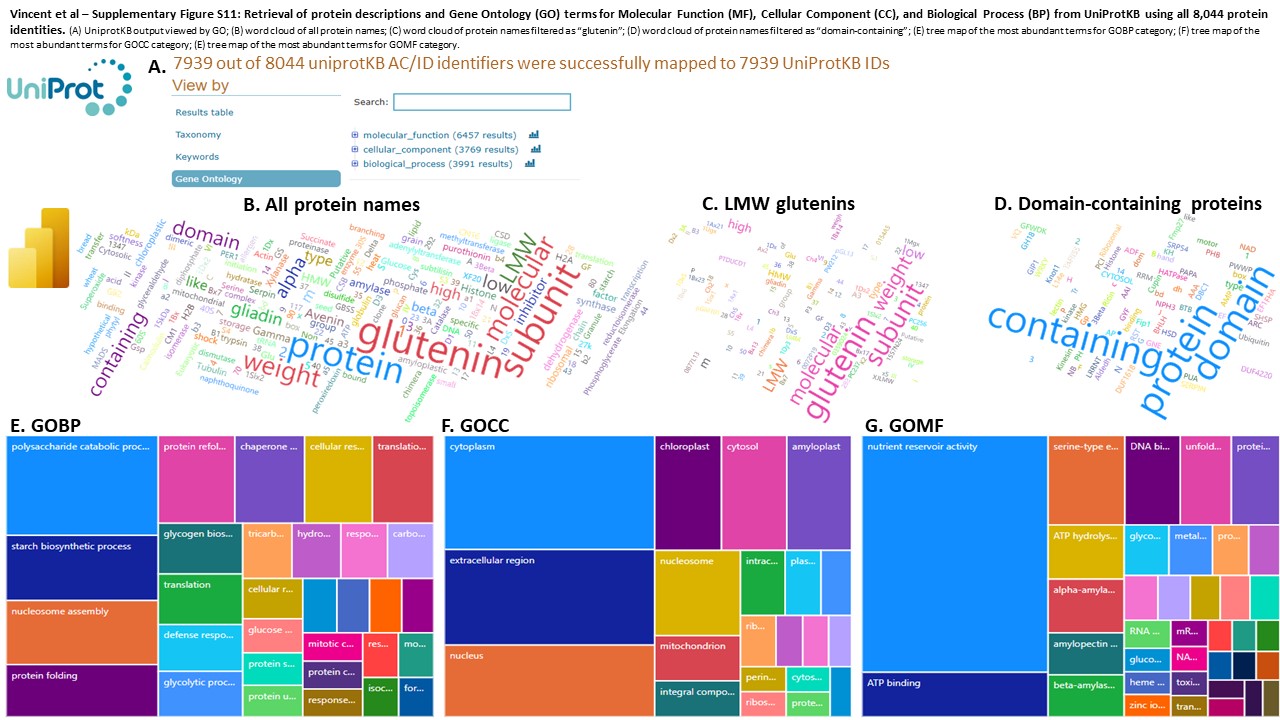

Supplement: giad084_Supplemental_Files [file giad084_supplemental_files.zip › Vincent_Suppl-Fig-S11.JPG]

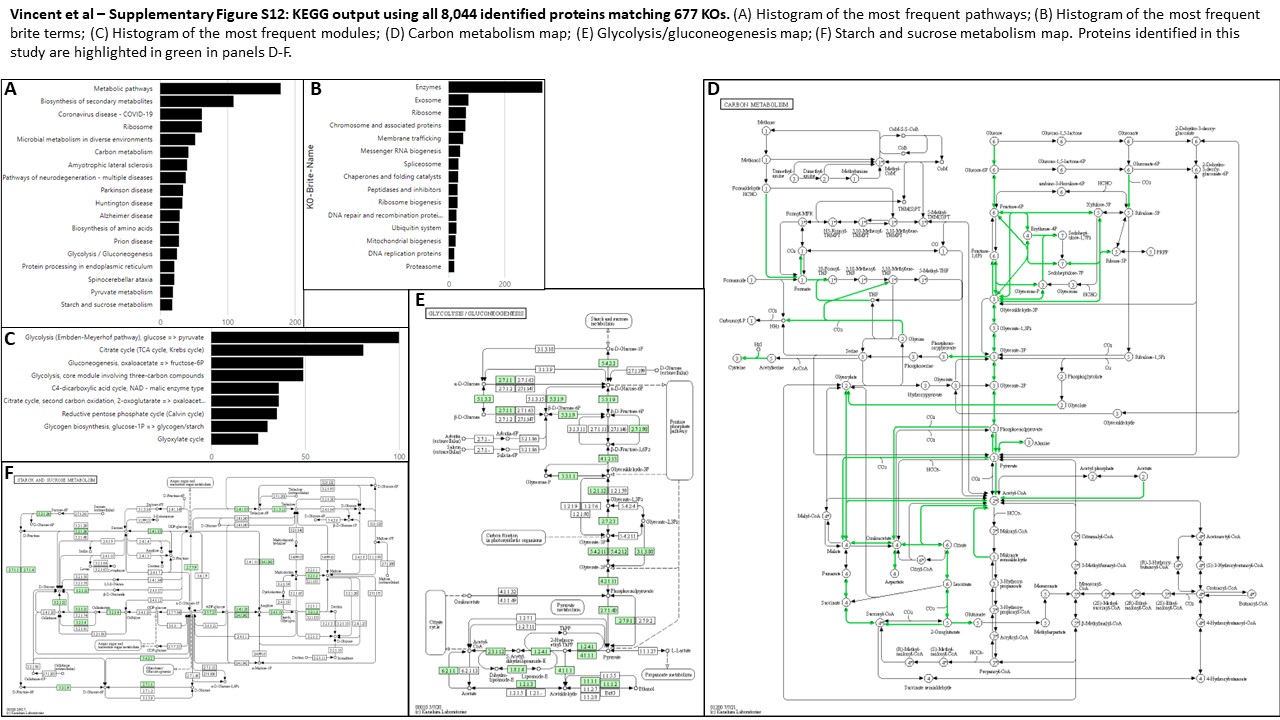

Supplement: giad084_Supplemental_Files [file giad084_supplemental_files.zip › Vincent_Suppl-Fig-S12.JPG]

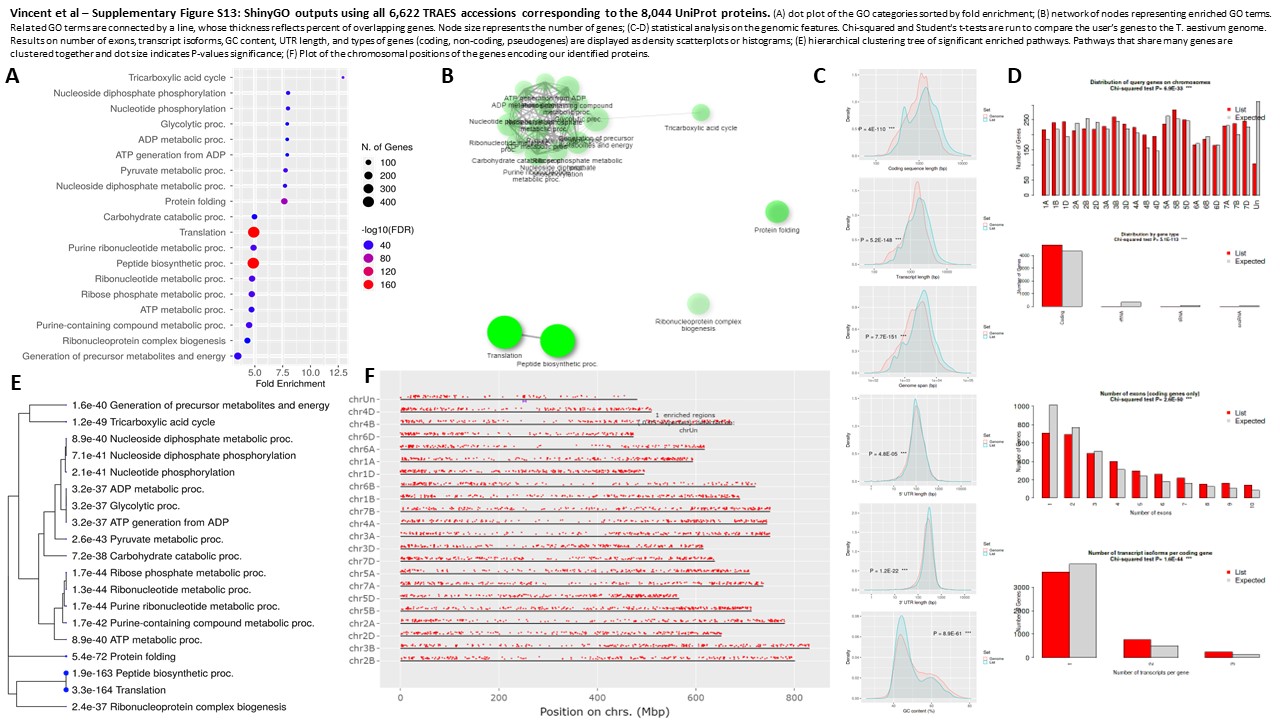

Supplement: giad084_Supplemental_Files [file giad084_supplemental_files.zip › Vincent_Suppl-Fig-S13.JPG]

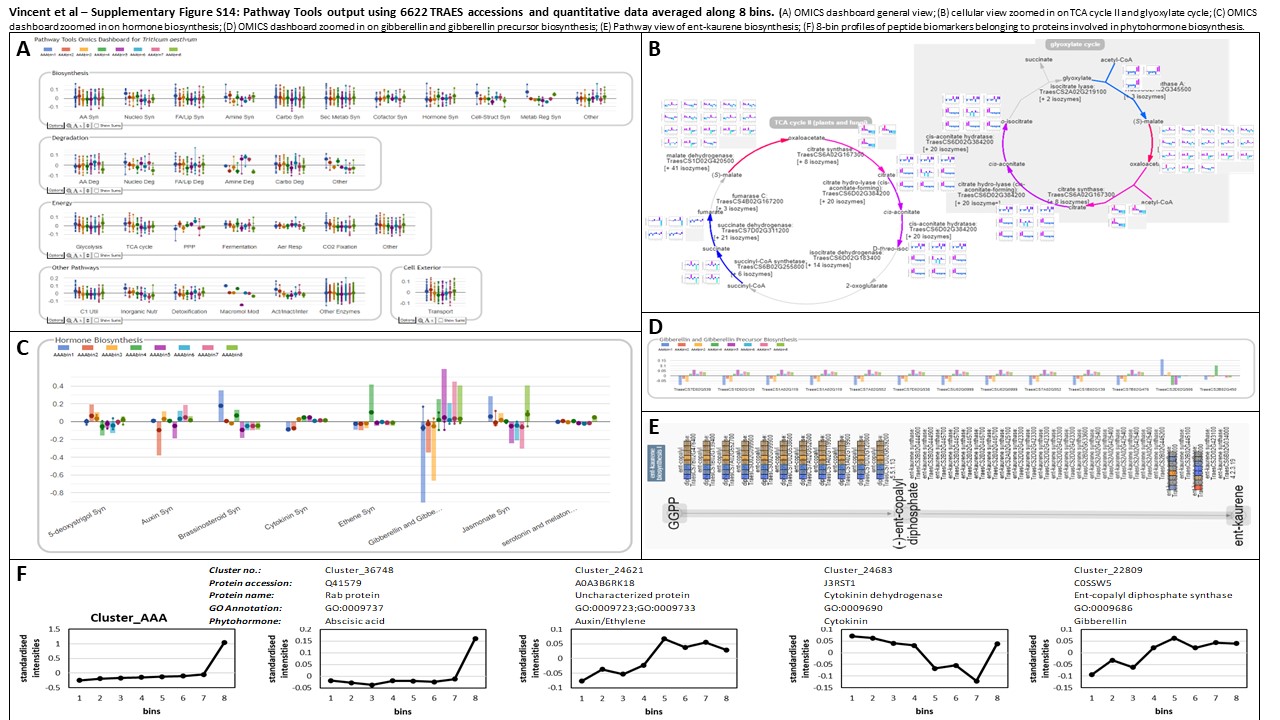

Supplement: giad084_Supplemental_Files [file giad084_supplemental_files.zip › Vincent_Suppl-Fig-S14.JPG]
