# Supplementary material for: A community resource to mass explore the wheat grain proteome and its application to the late-maturity alpha-amylase (LMA) problem
Source: Gigascience. 2023 Nov 1;12:giad084. doi: 10.1093/gigascience/giad084 (PMC10627334; doi:10.1093/gigascience/giad084)
Supplement: giad084_Response_to_Reviewer_Comments_Original_Submission [file giad084_response_to_reviewer_comments_original_submission.pdf]

Author's Response To Reviewer Comments

1/ Authors' responses to the Editor:

Your manuscript "Finding the LMA needle in the wheat proteome haystack." (GIGA-D-23-00108) has been assessed by our re
pleased to inform you that it is potentially acceptable for publication in GigaScience, once you have carried out some minor r
Authors' reply: thank you for processing our submission in due course and for granting us a generous deadline to revise our r
revisions using the Track Change tool in Word and we have included line numbers which were unfortunately omitted in the or

Their reports are below.

Editor's comments:
Reviewer #1 has mainly stylistic suggestions and believes the manuscript could be presented in a more concise way. I hope y
helpful to improve the clarity of your paper. However, with respect to this reviewer's comments on text length and style etc,
these issues are ultimately the choice of the authors. Making these more stylistic changes is not mandatory, in case you disag
Authors' reply: we thank the Editor for giving us the option to refute aspects pertaining to manuscript length. We have abridg
it was where we felt the article would lose its substance and its impact to the community. In our response to Reviewed #1, w

In particular, at GigaScience we put great emphasis on reproducibility and transparency of methods, and in this light I would
information to the supplement, contrary to the suggestion of the reviewer. It could be useful though to use the protocols-io p
https://urldefense.com/v3/__https://www.protocols.io/__;!!C5rN6bSF!Cm_QLxfqELRu5fy34r5ghXNsedGuFc3NcMeRGYz_Y6Q
gF2DzliYcKnV0-prUEWZfUhlmSKF-gA9iLwA$ to share detailed methods, which can then be cited via their doi in the manuscrip
Authors' reply: thank you for the suggestion. The protocol.io platform is something we'll keep in mind for the future.

Once you have made the necessary corrections, please submit a revised manuscript online at:
https://urldefense.com/v3/__https://www.editorialmanager.com/giga/__;!!C5rN6bSF!Cm_QLxfqELRu5fy34r5ghXNsedGuFc3N
gF2DzliYcKnV0-prUEWZfUhlmSKF-zN2s-A8$
Authors' reply: we have complied to all of Reviewer #1 suggestions and most of the reviewer #2 recommendations.
We offer rebuttals where we felt complying with Reviewer #2 would dilute the content of the article and reduce its impact on
Briefly, we disagreed with removing the photographs in Figure 1 as we strongly feel it helps understand our workflow. We hav
We were also against moving parts of method sections 2.6-2.9 to Supplementary Files, as it covers aspects essential in proce
analysing the data, running the statistics and mining the results.
We attempted to reduced Result section 3.1.3 and its associated Figure 3 but failed to do so as each normalisation deserves b
discussed.
Finally, we also decided against moving parts of Figure 6 to the Supplementary Files as it would imply that some analyses are
mining tools we employed are equally useful and informative, therefore either we'd move the whole Figure 6 to Supplementa
latter as it encapsulates the data mining tools used and illustrates how LMA biomarkers behave, which supports our statemen
us to move Figure 6 in its entirety to Supplementary File, we will do so. Please, instruct us accordingly.
Further to the reviewers' requirements, we have also updated Figure 2 (Gantt chart) to include the final actions (revisions, pu
complete.
We have added introductory tabs to the Excel supplementary files that lists the various tabs they contain.
We have reworded the Concluding remarks.
Finally, we have carefully read through the manuscript and corrected grammar and typographical mistakes.

2/ Authors' responses to Reviewer #1:

The large proteome dataset for wheat, a representative grain, presented in this manuscript is valuable not only for agricultur
science, but unfortunately, the manuscript is too wordy in its description and informative. Of course, a detailed description of
generation process is an important component in obtaining reproducibility, but excessive information in the main text may ha
the reader's understanding of the manuscript. The volume of the main text in this manuscript should be reduced to 1/2 or ev
the following suggested revisions.
Authors' reply: we appreciate Reviewer #1's candour for their feedback and assessment of our article. We understand their p
manuscript however this manuscript shouldn't be viewed as a research paper but rather as a community paper (i.e. with a br
just the wheat LMA one) which authorises more content. We agree it is a very long document and we tried making it as conci

description of the methods, workflows and strategies so that others can emulate them elsewhere. We appreciate Reviewer #1 parts that could be shrunk down and we have done our best to abide to their suggestion as indicated below. We apologise for original version as it would have made the reviewing process far easier. We have remedied this in the revised version.

Title: It looks rather like the title of a review article and is not appropriate for the title of an original research paper. An abbre to understand. It should be changed to a title that more specifically and pragmatically reflects the content of the paper.
Authors' reply: thank you for having noted this. We were after a memorable title that would elicit interest in the community b strategy. We have changed the title to "A community resource to mass explore the wheat grain proteome and its application (LMA) problem". We have updated the Supplementary Files accordingly. We hope Reviewer #1 will approve.

Materials and Methods 2.3: The sample pretreatment used in this experiment has already been described in Ref. 41, so detai unnecessary.
Authors' reply: thank you for the feedback. Indeed, this section was largely redundant. We now only detail novel aspects that such a large volume of samples. We have rephrased as follows (P7 L206-207) "Detailed hereafter are technical considerations a large volume of samples. The overall workflow is it is schematised in Figure 1.". Following that we have removed redundant section from 47 lines down to 23 lines (P7-8 L218-260). We hope Reviewer #1 will be pleased with this.

Also, Figure 1, which visualizes the experimental process, is too packed with information and is difficult to read in its small fo photographs of LC-MS instruments and other common equipment are included.
Authors' reply: we appreciate Reviewer #1's viewpoint however we disagree with the fact that Figure 1 is too busy. It faithful technical workflow which could be hard to follow and emulate. The photographs and pictograms are needed to illustrate what equipment we used as detailed in the methods. Of course, scientists wishing to reproduce our method do not have to use the like to equipment themselves the same way we did, this figure 1 will help them do so. Therefore, for all these reasons we thi relevant and not extraneous. However, we agree font size was too small; consequently, we have increased the size of the tex the figure to accommodate for it. We have also added consecutive numbers to each step text box to make the order unequivo the arrows to emphasise the sequence of the steps.

Sample pretreatment should be described very briefly in the text, and only those areas where there are differences from prev the author wishes to describe the details of the experiment to assure reproducibility, it is recommended to describe it in the f include it in the Supplementary Information.
Authors' reply: we are assuming Reviewer #2 refers to Section 2.4 (LC-MS analyses). Indeed, some details are available from eliminated redundant parts (P8-9 L254-295). In doing so, 11 lines were removed thus reducing this section by 30%. We hope

Materials and Methods 2.5: The 11 different paths the authors have set up for LC-MS/MS analysis are difficult to understand summarized in a table or visualized using a flowchart.
Authors' reply: thank you for the excellent suggestion. We have added a table to Supplementary File SF2 in the 1st tab summ entitled "Summary of MS parameters across all 11 passes". We have consequently slightly reduced the description of these p thus eliminating 11 lines. We hope Reviewer #1 will find this acceptable.

Materials and Methods 2.6 to 2.9: It is recommended that only the essentials be described in the text and the minute details Information.
Authors' reply: We thank reviewer #2 for this feedback, however we have already shrunk this section to the bare minimum w of the paper as these aspects were essential in processing all those LC-MS1 and LC-MS2 files, analysing the data, running the those sections to the supplementary file would reduce the impact of the article and its relevance to the scientific community. them. We hope Reviewer #1 will accept our rebuttal.

Results 3.2.(p 26, line 11-20): The description should be moved to the introduction.
Authors' reply: It's a valid point however we feel it introduces the application of our strategy to LMA nicely, so we'd rather lea Introduction section would disjoint it from the results/discussion where we address this. Nonetheless, thanks to Reviewer #1' first 3 sentences (P27 L840-845=6 lines) to reduce the text.

Results 3.1.3-3.1.4 & 3.2.1.-3.2.3 : Too detailed and too long. Only the main points should be mentioned. It would be effectiv possible.
Authors' reply: thank you for the feedback. We attempted to reduce Section 3.1.3 but failed to do so as each normalisation d and briefly discussed. This holds true for Figure 3 which depicts what each normalisation step does to the data. We managed 772) to some extent by eliminating 12 lines (25%). We have created a separate section for PTMs by inserting the section title modifications (PTMs)" (P23 L773)

Figure 6: Too much information; A, B, F, and G should be supplemental information.
Authors' reply: We concede Figure 6 is busy however we do not understand why we'd consign some parts of it to the Suppler [illegible] stay in the main article. That would imply that some analyses are more relevant than others. All data mining tools we employ [illegible] therefore either we'd move the whole figure to Supplement File or leave it as it is. We chose the latter as it encapsulates the [illegible] how LMA biomarkers behave which supports our statements in the text. We hope Reviewer #1 will accept our decision howev [illegible] Figure 6 in its entirety and as it is to the Supplementary Files, we'd comply.

Figure 8: Wheat cartoon is unnecessary. The font is too small. This information should be in a Table.
Authors' reply: Thank you for the great suggestion. Yes, indeed we agree that a table would be an adequate substitute. We h [illegible] into Supplementary Table S7 entitled "List of mechanisms involved in LMA response" and changed the text accordingly (P41 L [illegible] find this satisfactory.

3/ Authors' responses to Reviewer #2:

General comments
This paper is a very thorough report on large-scale proteomics mapping of ca. 4000 wheat samples and several challenges re [illegible] measurement and data analysis. It is the first paper reporting such an extensive dataset and tools for analysis. Overall, I thir [illegible] work and it is also described in a way that can be understood well. The descriptions of how the authors arrived at the final wo [illegible] groups attempting to do proteomics of wheat or other grains. I have only few comments for improvement.
Note: line numbers would have been helpful
Authors' reply: we appreciate Reviewer #2's positive feedback and assessment of our article. We apologise for omitting to inc [illegible] version as it would have made the reviewing process far easier. We have remedied this in the revised version.

Specific comments
Abstract - Results: "LMA expression greatly impacted grain starch and other carbohydrates …" and then alpha-gliadins and LM [illegible] these are proteins and their relation to starch/carbohydrates is not clear Introduction overall:
Authors' reply: thank you for raising this; indeed, this required correcting as gliadins and glutenins are storage proteins. We [illegible] that now reads "Finally, and unsurprisingly, LMA expression greatly impacted grain storage proteins, as well as starch and oth [illegible] regulation of alpha-gliadins and starch metabolism, whereas LMW glutenin, stachyose, sucrose, UDP-galactose and UDP-gluc [illegible] Reviewer #1 will find this acceptable.

Please harmonize the use of alpha-amylase and a-amylase; alpha-amylase is recommended, or else the Greek letter.
Authors' reply: thank you for noting this. Conventionally the Greek letter is used but as it can lead to formatting issue upon c [illegible] favour of using the fully spelled name "alpha-amylase". We have checked throughout the text, table, legends, figures, and su [illegible] needed (P4 L123 and P5 L148). We hope Reviewer #1 will find this satisfactory.

p3, L1: "great source of protein": In terms of quantity, this is true. However, you should also include a brief statement about [illegible] especially when considering gluten proteins
Authors' reply: this is very true and particularly topical with the rise of gluten intolerance and coeliac disease reports. Thank [illegible] thorough review by Shewry that we cite (reference [5]) also covers adverse reactions to wheat products, so we drew upon it. [illegible] which now states on P3 L71-74: "Wheat grains are not only a major source of carbohydrate in the form of starch which can r [illegible] but also a substantial source of protein, representing up to 15% of grain dry weight [5]. Wheat proteins can however trigger [illegible] intolerance, or food and respiratory allergies [5]." We hope Reviewer #2 will find this change satisfactory.

section 2.1: Please include if all samples were grown together at the same place in one year (or not); i.e. include the informa [illegible]
Authors' reply: yes, indeed this information is missing from the Materials section. Thank you for noting this. We have now am [illegible] 194) "The wheat collection of 858 genotypes used in this study represents a diverse range of cultivars and germplasm source [illegible] Genebank and representing worldwide genetic diversity. Wheat was grown in a single location in field trials at Horsham Victor [illegible] using a mechanical small-plot harvester. We hope Reviewer #2 will find this description suitable.
