# Supplementary material for: A community resource to mass explore the wheat grain proteome and its application to the late-maturity alpha-amylase (LMA) problem
Source: Gigascience. 2023 Nov 1;12:giad084. doi: 10.1093/gigascience/giad084 (PMC10627334; doi:10.1093/gigascience/giad084)
Supplement: giad084_Response_to_Reviewer_Comments_Revision_1 [file giad084_response_to_reviewer_comments_revision_1.pdf]

Author's Response To Reviewer Comments

Tuesday 22 August 2023
Dear Hans Zauner,
Thank you for your instructions.
Please find below my replies to your comments.
I have now re-opened the submission system for you, to make the changes and additions you mentioned in your email. To upload, please follow the link below.
We have made the necessary changes as you'll see below and have resubmitted the manuscript, including Supplementary File SF1 which now contains the R script linking LCMS1 to LCMS2.
We have also resubmitted Supplementary File SF2 which was removed by mistake during the revisions.
This SF2 file will have to be added to our GigaDB dataset and linked to the dataset current DOI.
I will contact the curator and explain what to do.

In addition, please address the following editorial points:

1) Please include a numbered citation to your upcoming GigaDB dataset (including the DOI link) to your reference list, and cite this in the data availability section.
You will get the exact citation details and doi from our curators.
In the data availability section, please write something along the lines, "Supporting data [including x,y,z] is also available via the GigaScience repository, GigaDB [xx]".
Curators have contacted me enjoining me to complete my dataset submission in GigaDB. I now have a doi and citation which I have both included to the manuscript as follows.

To the section "Data Availability", we have included the following statement:
"Supporting data [including Supplementary Figures S1-S14, Supplementary Tables S1-S7, and Supplementary Files SF1-SF2] is also available via the GigaScience repository, GigaDB [156]."

To the section "References", I have added as the last reference:
"156. Vincent D, Bui A, Ezernieks V, Shahinfar S, Luke T, Ram D, et al. Supporting data for "A community resource to mass explore the wheat grain proteome and its application to the Late Maturity Alpha-Amylase (LMA) problem". GigaScience Database. 2023; http://dx.doi.org/10.5524/102436."

2) Please add ORCIDs for all authors to the title page, if available.
We have included existing ORCIDs.

3) Please remove any tracking of changes in the document that was made for the purpose of peer review.
We have accepted all changes and stopped the tracking.

Please also ensure that your revised manuscript conforms to the journal style, which can be found in the Instructions for Authors on the journal homepage.
We have formatted the manuscript according to GigaScience's instructions to authors.
We have also used latex overleaf to convert our manuscript to the correct template ensuring that the manuscript structure (with 4 levels of sections and subsections) is appropriate.
We have uploaded all those new and missing files to the system, including the overleaf code and bibliography.

The final list of files needed for this submission is:

1. Vincent_2023-08-21.tex [overleaf latex code]
2. Vincent_gigascience_2023-08-21.bib [bibliography]
3. Vincent_wheat_4000lines_article_2023-08-16.docx [article with figures, docx format]
4. Vincent_wheat_4000lines_article_2023-08-16.pdf [article with figures, docx converted to pdf format]
5. Vincent_wheat_4000lines_article_2023-08-16_overleaf-latex.pdf [article with figures, formatted using overleaf latex]

6. Vincent_graphical-abstract_2023-08-21 [summary figure]
7. Vincent_wheat_4000lines_figures_2023-07-20.pptx [figures]
8. Vincent_wheat_4000lines_Suppl-figures_2023-07-20.pdf [supplementary figures]
9. Vincent_wheat_4000lines_Suppl-tables_2023-07-20.xlsx [supplementary tables]
10. Vincent_wheat_4000lines_Suppl-File-SF1_2023-08-16.pdf [supplementary method text]
11. Vincent_wheat_4000lines_Suppl-File-SF2_2023-07-20.xlsx [supplementary method tables]
12. Vincent_wheat_4000lines_Suppl-Video-SV1_2023-07-20.mp4 [supplementary video]
13. Vincent-et-al_cover-letter_GigaScience_2023-08-22 [cover letter]

Hopefully, none were omitted.

If the data and code has been modified in the revision process please be sure to update the public versions of this too.
We have not changed any data or code since the initial submission of the manuscript.

I hope that all is in order.
If not, please do not hesitate to contact me again.

Best regards,
Delphine Vincent.
