# Supplementary material for: A community resource to mass explore the wheat grain proteome and its application to the late-maturity alpha-amylase (LMA) problem
Source: Gigascience. 2023 Nov 1;12:giad084. doi: 10.1093/gigascience/giad084 (PMC10627334; doi:10.1093/gigascience/giad084)
Supplement: giad084_Response_to_Reviewer_Comments_Revision_2 [file giad084_response_to_reviewer_comments_revision_2.pdf]

Author's Response To Reviewer Comments

Dear Hans Zauner,
Thank you for finalising the processing and for recommending more minor revisions to ensure the manuscript format is corre[illegible]
addressed all your points in our 3rd revision and explained below in green what we did.
- In the main text, the GigaScience format does not allow URLs and other hyperlinks such as ftp addresses. All URLs and othe[illegible]
need to be deleted from the main text, but you can refer to online sources by giving any online source their own, numbered i[illegible]
bibliography. For example, line 1260:
The authors: Thank you. All the hyperlinks have been removed. Those not covered by citations were converted to citations th[illegible]

"KO identifiers were then mapped using the KEGG Mapper Reconstruct tool
(https://urldefense.com/v3/__https://www.genome.jp/kegg/mapper/reconstruct.html__;!!C5rN6bSF!F0nNSbByCFH4pyYl6rd[illegible]
ye2zwC5G2eNCSs3-fDD1mezbA_Z8lXKBGjG_d2aEt6rRiXE50zcRXGQQVszjbfQEemlM$, accessed on May 2022) " should be re[illegible]
identifiers were then mapped using the KEGG Mapper Reconstruct tool [citation number]".
Please refer to the citation guidelines on our homepage for details.
The authors: Thank you, this has been done.

- In the data availability section, please just mention the MASSIVE accession number, which is a unique and stable identifier, [illegible]
the ftp link.
The authors: Thank you. This has been done and we've added the citation with the link to the repository.

- Some of the supplemental files are now included in the GigaDB repository. I'm not sure if you want to keep them in the arti[illegible]
supplement as well - if it's redunant, that's probably not necessary, but it's up to you. (To explain, supplemental files that are[illegible]
via Editorial Manager will be hosted/linked on our publisher's homepage together with the article, independent of our databas[illegible]
The GigaDB entry has the advantage that it has its own unique identifier (doi) and is citable independently of the article).
The authors: Thank you. The GigaDB team has indeed uploaded all my supplementary files therefore we do not need to keep [illegible]
article supplement. In the data availability statement, we indicate the citation referring to our GigaDB DOI. In the additional f[illegible]
statement, we list all the supplements with a short description.

We hope you will find these changes satisfactory. Please note that I'm attending a conference from 19-23 Sept with limited in[illegible]
access, and then I'm on leave from 24 Sept – 10 Oct during which I will not check my emails at all.
Kind regards,
Delphine Vincent.
