# Supplementary material for: A community resource to mass explore the wheat grain proteome and its application to the late-maturity alpha-amylase (LMA) problem
Source: Gigascience. 2023 Nov 1;12:giad084. doi: 10.1093/gigascience/giad084 (PMC10627334; doi:10.1093/gigascience/giad084)
Supplement: giad084_Reviewer_1_Report_Original_Submission — Nobuaki Takemori, Ph.D. -- 5/22/2023 Reviewed [file giad084_reviewer_1_report_original_submission.pdf]

Reviewer Report

Title: A community resource to mass explore the wheat grain proteome and its application to the Late Maturity Alpha-Amylase (LMA) problem.

Version: Original Submission **Date:** 5/22/2023

Reviewer name: Nobuaki Takemori, Ph.D.

Reviewer Comments to Author:

The large proteome dataset for wheat, a representative grain, presented in this manuscript is valuable not only for agriculture science but also for basic plant science, but unfortunately, the manuscript is too wordy in its description and informative. Of course, a detailed description of the experimental methods and data generation process is an important component in obtaining reproducibility, but excessive information in the main text may have the unintended effect of hindering the reader's understanding of the manuscript. The volume of the main text in this manuscript should be reduced to 1/2 or even 1/3 of the original by referring to the following suggested revisions.
Title: It looks rather like the title of a review article and is not appropriate for the title of an original research paper. An abbreviation is also used, making it difficult to understand. It should be changed to a title that more specifically and pragmatically reflects the content of the paper.
Materials and Methods 2.3: The sample pretreatment used in this experiment has already been described in Ref. 41, so detailed description in this text is unnecessary. Also, Figure 1, which visualizes the experimental process, is too packed with information and is difficult to read in its small font. In addition, many extraneous photographs of LC-MS instruments and other common equipment are included. Sample pretreatment should be described very briefly in the text, and only those areas where there are differences from previous reports should be mentioned. If the author wishes to describe the details of the experiment to assure reproducibility, it is recommended to describe it in the form of an experimental protocol and include it in the Supplementary Information.
Materials and Methods 2.5: The 11 different paths the authors have set up for LC-MS/MS analysis are difficult to understand in text. Maybe they could be summarized in a table or visualized using a flowchart.
Materials and Methods 2.6 to 2.9: It is recommended that only the essentials be described in the text and the minute details be moved to the Supplementary Information.
Results 3.2.(p 26, line 11-20): The description should be moved to the introduction.
Results 3.1.3-3.1.4 & 3.2.1.-3.2.3ï¼šToo detailed and too long. Only the main points should be mentioned. It would be effective to use concise Figures where possible.
Figure 6: Too much information; A, B, F, and G should be supplemental information.
Figure 8: Wheat cartoon is unnecessary. The font is too small. This information should be in a Table.

Methods

Are the methods appropriate to the aims of the study, are they well described, and are necessary controls included? Choose an item.

Conclusions

Are the conclusions adequately supported by the data shown? Choose an item.

Reporting Standards

Does the manuscript adhere to the journal's guidelines on minimum standards of reporting? Choose an item.

Choose an item.

Statistics

Are you able to assess all statistics in the manuscript, including the appropriateness of statistical tests used? Choose an item.

Quality of Written English

Please indicate the quality of language in the manuscript: Choose an item.

Declaration of Competing Interests

Please complete a declaration of competing interests, considering the following questions:

- Have you in the past five years received reimbursements, fees, funding, or salary from an organisation that may in any way gain or lose financially from the publication of this manuscript, either now or in the future?
- Do you hold any stocks or shares in an organisation that may in any way gain or lose financially from the publication of this manuscript, either now or in the future?
- Do you hold or are you currently applying for any patents relating to the content of the manuscript?
- Have you received reimbursements, fees, funding, or salary from an organization that holds or has applied for patents relating to the content of the manuscript?
- Do you have any other financial competing interests?
- Do you have any non-financial competing interests in relation to this paper?

If you can answer no to all of the above, write 'I declare that I have no competing interests' below. If your reply is yes to any, please give details below.

I declare that I have no competing interests.

I agree to the open peer review policy of the journal. I understand that my name will be included on my report to the authors and, if the manuscript is accepted for publication, my named report including any attachments I upload will be posted on the website along with the authors' responses. I agree for my

report to be made available under an Open Access Creative Commons CC-BY license (http://creativecommons.org/licenses/by/4.0/). I understand that any comments which I do not wish to be included in my named report can be included as confidential comments to the editors, which will not be published.

Choose an item.

To further support our reviewers, we have joined with Publons, where you can gain additional credit to further highlight your hard work (see: https://publons.com/journal/530/gigascience). On publication of this paper, your review will be automatically added to Publons, you can then choose whether or not to claim your Publons credit. I understand this statement.

Yes Choose an item.
