# Supplementary material for: A community resource to mass explore the wheat grain proteome and its application to the late-maturity alpha-amylase (LMA) problem
Source: Gigascience. 2023 Nov 1;12:giad084. doi: 10.1093/gigascience/giad084 (PMC10627334; doi:10.1093/gigascience/giad084)
Supplement: giad084_Reviewer_2_Report_Original_Submission — Katharina Scherf -- 6/15/2023 Reviewed [file giad084_reviewer_2_report_original_submission.pdf]

Reviewer Report

Title: A community resource to mass explore the wheat grain proteome and its application to the Late Maturity Alpha-Amylase (LMA) problem.

Version: Original Submission **Date:** 6/15/2023

Reviewer name: Katharina Scherf

Reviewer Comments to Author:

General comments
This paper is a very thorough report on large-scale proteomics mapping of ca. 4000 wheat samples and several challenges related to sample preparation, measurement and data analysis. It is the first paper reporting such an extensive dataset and tools for analysis. Overall, I think that the authors have done in-depth work and it is also described in a way that can be understood well. The descriptions of how the authors arrived at the final workflow will also be useful to other groups attempting to do proteomics of wheat or other grains. I have only few comments for improvement.
Note: line numbers would have been helpful
Specific comments
Abstract - Results: "LMA expression greatly impacted grain starch and other carbohydrates …" and then alpha-gliadins and LMW glutenin is mentioned. However, these are proteins and their relation to starch/carbohydrates is not clear
Introduction overall: Please harmonize the use of alpha-amylase and a-amylase; alpha-amylase is recommended, or else the Greek letter.
p3, L1: "great source of protein": In terms of quantity, this is true. However, you should also include a brief statement about protein quality, which is not ideal, especially when considering gluten proteins
section 2.1: Please include if all samples were grown together at the same place in one year (or not); i.e. include the information from section 3.1.1 already here

Methods

Are the methods appropriate to the aims of the study, are they well described, and are necessary controls included? Choose an item.

Conclusions

Are the conclusions adequately supported by the data shown? Choose an item.

Reporting Standards

Does the manuscript adhere to the journal’s guidelines on minimum standards of reporting? Choose an item.

Choose an item.

Statistics

Are you able to assess all statistics in the manuscript, including the appropriateness of statistical tests used? Choose an item.

Quality of Written English

Please indicate the quality of language in the manuscript: Choose an item.

Declaration of Competing Interests

Please complete a declaration of competing interests, considering the following questions:

- Have you in the past five years received reimbursements, fees, funding, or salary from an organisation that may in any way gain or lose financially from the publication of this manuscript, either now or in the future?
- Do you hold any stocks or shares in an organisation that may in any way gain or lose financially from the publication of this manuscript, either now or in the future?
- Do you hold or are you currently applying for any patents relating to the content of the manuscript?
- Have you received reimbursements, fees, funding, or salary from an organization that holds or has applied for patents relating to the content of the manuscript?
- Do you have any other financial competing interests?
- Do you have any non-financial competing interests in relation to this paper?

If you can answer no to all of the above, write 'I declare that I have no competing interests' below. If your reply is yes to any, please give details below.

I declare that I have no competing interests

I agree to the open peer review policy of the journal. I understand that my name will be included on my report to the authors and, if the manuscript is accepted for publication, my named report including any attachments I upload will be posted on the website along with the authors' responses. I agree for my report to be made available under an Open Access Creative Commons CC-BY license (http://creativecommons.org/licenses/by/4.0/). I understand that any comments which I do not wish to be included in my named report can be included as confidential comments to the editors, which will not be published.

Choose an item.

To further support our reviewers, we have joined with Publons, where you can gain additional credit to further highlight your hard work (see: https://publons.com/journal/530/gigascience). On publication of this paper, your review will be automatically added to Publons, you can then choose whether or not to claim your Publons credit. I understand this statement.

Yes Choose an item.
